# A network meta-analysis of efficacy, acceptability, and tolerability of antipsychotics in treatment-resistant schizophrenia

**DOI:** 10.1007/s00406-023-01654-2

**Published:** 2023-08-01

**Authors:** Shimeng Dong, Johannes Schneider-Thoma, Irene Bighelli, Spyridon Siafis, Dongfang Wang, Angelika Burschinski, Kristina Schestag, Myrto Samara, Stefan Leucht

**Affiliations:** 1 Department of Psychiatry and Psychotherapy, School of Medicine, Technical University of Munich, Klinikum rechts der Isar, Ismaningerstr. 22, 81675 Munich, Germany; 2 Department of Psychiatry, Faculty of Medicine, University of Thessaly, Larisa, Greece

**Keywords:** Schizophrenia, Non-response, Clozapine, Olanzapine, Antipsychotics

## Abstract

**Objective:**

Clozapine is considered as the standard treatment for this subgroup, but the evidence is not unequivocal. There are several potential alternatives being used because of the possible adverse effects of clozapine. We aimed to examine the efficacy and adverse events of different antipsychotics in treatment-resistant schizophrenia by performing a network meta-analysis.

**Methods:**

We searched the Cochrane Schizophrenia Group register for randomized-controlled trials (up to March 06, 2022) and MEDLINE (up to January 20, 2023). We included blinded and open studies and participants with a broad definition of treatment resistance. The primary outcome was overall symptoms of schizophrenia; secondary outcomes were response to treatment, positive and negative symptoms of schizophrenia, discontinuation, side effects, quality of life, and functioning. The study was registered in Open Science Framework (https://osf.io/9nf2y/).

**Results:**

We included 60 studies involving 6838 participants in the network meta-analysis. In the primary outcome, clozapine and olanzapine were more efficacious than risperidone, haloperidol, fluphenazine, sertindole, chlorpromazine, and quetiapine (range of mean SMDs, − 0.11 to − 0.48). The difference between clozapine and olanzapine was trivial and uncertain (SMD − 0.05, 95% CI, − 0.21 to 0.11). The result of other efficacy outcomes as well as subgroup and sensitivity analyses were consistent with the primary analysis. Clozapine and olanzapine were associated with more weight gain, and clozapine was associated with more sedation events compared to many other antipsychotics.

**Conclusions:**

Clozapine remains the gold standard for patients with treatment-resistant schizophrenia. Olanzapine seems to be second-best and could be tried before switching to clozapine.

**Supplementary Information:**

The online version contains supplementary material available at 10.1007/s00406-023-01654-2.

## Introduction

In short-term trials, as many as 40% of patients with schizophrenia experienced no or little benefit from antipsychotic treatment [1]. In addition, as many as 20% to 30% patients who initially responded to antipsychotic drugs relapsed within one or 2 years despite maintenance treatment [2]. Therefore, managing patients with schizophrenia who are non-responsive to antipsychotics is a major challenge for doctors and health care systems worldwide [3]. Clozapine is considered as the standard antipsychotic for treatment-resistant schizophrenia [4–6], but the evidence is actually not as clear as one may believe. The superiority of clozapine was established by a landmark study that presented an impressive effect size of clozapine compared with chlorpromazine [2]. Subsequent clinical trials done in the 1990s which compared clozapine with first-generation antipsychotics (FGAs) for treatment-resistant schizophrenia provided more robust evidence of clozapine’s effectiveness [7, 8]. In the following decade, the superiority of clozapine compared with FGAs was corroborated again by a meta-analysis and a Cochrane review [9, 10].

However, whether clozapine is more efficacious than other second-generation antipsychotics (SGAs) for treatment-resistant schizophrenia is not undisputed. A meta-analysis by Siskind et al. that compared clozapine with both first- and second-generation antipsychotics indicated a positive answer for the superiority of clozapine [11]. In contrast, in the same time period, a network meta-analysis [12] including nine antipsychotics showed that all included blinded randomized-controlled trials (RCTs) provided little evidence to draw that conclusion. Which antipsychotic drug was more efficacious for treatment-resistant schizophrenia remained unclear. We therefore broadened the inclusion criteria of previous review [12] with an attempt to perform a comprehensive network meta-analysis of both FGAs and SGAs, including all age groups (from children and adolescents to elderly) and both blinded and open-label RCTs in treatment-resistant schizophrenia.

## Methods

### Participants and interventions

We included all RCTs of patients with a treatment-resistant form of schizophrenia, schizoaffective disorder, or schizophreniform disorder. To conduct a comprehensive network meta-analysis, patients according to study-defined definition of treatment resistance were included without any age limit or other restrictions; however, in a subgroup analysis, we addressed different levels of resistance (see below). We included all SGAs available in Europe or the US, and a selection of FGAs based on a survey of international schizophrenia experts [13] (amisulpride, aripiprazole, asenapine, benperidol, brexpiprazole, cariprazine, chlorpromazine, clopenthixol, clozapine, flupentixol, fluphenazine, fluspirilene, haloperidol, iloperidone, levomepromazine, loxapine, lumateperone, lurasidone, molindone, olanzapine, paliperidone, penfluridol, perazine, perphenazine, pimozide, quetiapine, risperidone, sertindole, sulpiride, thioridazine, tiotixene, trifluoperazine, ziprasidone, zotepine, and zuclopenthixol). We included all forms of administration, including long-acting injectables (LAIs) at any dose. Antipsychotic drugs being used as an augmentation or combination strategy were excluded. The minimum duration of trials was set at three weeks.

### Search strategy

We searched the Cochrane Schizophrenia Group’s (CSzG) Study-Based Register of Trials on April 27, 2020. We made two update searches of CSzG on September 19, 2021, and March 06, 2022, respectively. The last update search in MEDLINE was on January 20, 2023. A detailed search strategy can be found in the appendix (pp 9–11). Additionally, we inspected the reference lists of the included studies and previous reviews on treatment-resistant schizophrenia [11, 12, 14].

### Study selection

All published and unpublished RCTs, as well as blinded and open-label trials, were included. We excluded studies with a high risk of bias in the randomization process and studies from mainland China due to quality concerns [15].

At least two reviewers among AB, KS, SD, and DW screened all identified studies independently at the title and abstract level. Studies that met the inclusion criteria on the title and abstract review, or that could not be excluded due to insufficient information at this level, were reviewed in full texts. In case of discrepancies, a third reviewer (SL) was involved.

### Outcomes

The primary outcome was the overall symptoms of schizophrenia as measured by rating scales such as the Positive and Negative Syndrome Scale (PANSS) [16], the Brief Psychiatric Rating Scale (BPRS) [17], or of any other validated scale (e.g., the Manchester Scale [18]). Changes from baseline to endpoint data were preferred to endpoint data, if available.

Secondary outcomes were response to treatment (detailed definitions are presented in the appendix [p 42]), positive and negative symptoms of schizophrenia (measured by PANSS subscale, BPRS subscale, or any other rating scale), dropouts due to any reason (all-cause discontinuation), dropouts due to inefficacy of treatment, dropouts due to adverse events (if possible, only side-effect related dropouts due to adverse events were used), the occurrence of specific adverse events (use of antiparkinsonian medication, weight gain, sedation, prolactin levels, and QTc prolongation), and quality of life and functioning.

We also extracted age, diagnosis, duration of illness, number of previous episode, and baseline severity as patient characteristics; sample size, number of male and female participants, publication year, study duration, masking type, definition of treatment resistance, intervention, application, dose, and funding source as study characteristics.

### Data extraction

Two reviewers among SD, DW, and MS extracted the data independently, and entered them in electronic form in Microsoft Access 2009. Differences were discussed, and if a consensus was not reached, we contacted a third reviewer (SL) for a final decision. Attempts were made to contact first or corresponding authors for missing information about their studies.

### Data analysis

We conducted random-effects pairwise meta-analyses and network meta-analyses in a frequentist framework using the package *netmeta* in R (version 4.1.2) [19]. We calculated standardized mean differences (SMDs) for efficacy-related continuous outcomes and odds ratios (ORs) for binary outcomes, both presented with their 95% credible intervals (CIs). Nevertheless, we used mean differences (MDs) for clinically palpable results as weight gain, prolactin, and QTc prolongation. We transformed ORs to more interpretable relative risks (RR). Additionally, we calculated the relative ranking for each intervention within the frequentist framework (as P-scores) and used them to present the results according to this order [20].

The transitivity assumption was evaluated by comparing the distribution of potential effect modifiers (baseline severity, blinding of outcome assessor, treatment-resistant definition, publication year, sample size, mean age, and study duration) across studies grouped by interventions.

We assumed a common heterogeneity parameter across the various treatment comparisons and presented the between-study variance tau-squared (*τ*^2^) for each outcome. We characterized the amount of heterogeneity as low, moderate, or high using the first and third quantiles of their empirical distributions [21, 22].

We evaluated inconsistency statistically using the design-by-treatment interaction model [23] that assesses inconsistency globally in the network and by separating indirect from direct evidence and testing the agreement of these two parts of evidence (SIDE test) [24].

We explored the potential sources of heterogeneity or inconsistency using a priori planned subgroup analyses for the primary outcome on the following potential effect modifiers: the criteria of treatment-resistant definitions classified as low, intermediate, and high cut-off in terms of stringency (Table 1), mean age, dose of the antipsychotics in chlorpromazine-equivalents [25], publication year, baseline severity, and study duration.

**Table 1:** Criteria of treatment-resistant definitions

Criteria of treatment-resistant definitions	
Low cut-off	Non-response or intolerant to antipsychotics without a specification and studies that do not meet intermediate or high cut-off criteria
Intermediate cut-off	Failure of response to at least 2 trials with antipsychotics at dosage in the therapeutic range and adequate duration and persistent at least moderate symptoms assessed with standardized rating scales
High cut-off	Failure of response to at least two antipsychotic drug trials at dosage in the therapeutic range and adequate duration and at least one of them was prospective, also persistent at least moderate symptoms assessed with standardized rating scales at an assessment at the end of the prospective trial

Sensitivity analyses were conducted excluding studies that were not double-blind, studies that presented only completer analyses, studies that did not use operationalized criteria to diagnose schizophrenia, studies with high overall bias, and studies that only included children and/or adolescents. In post-hoc analyses, we excluded studies that included intolerant patients, studies that used low clozapine doses (< 400 mg/d), studies from clozapine’s manufacturer, studies from olanzapine’s manufacturer, and studies that used off-label dose olanzapine (> 20 mg/d). In a most extreme sensitivity analysis, we included only situations in which clozapine may be most superior; namely, double-blind studies with high-dose clozapine, and non-intolerant patients with high cut-off treatment-resistance criteria. Additionally, three old studies [2, 7, 26] comparing clozapine with chlorpromazine were excluded in the sensitivity analysis of the primary outcome, because they led to significant inconsistency.

Risk of bias was independently assessed by two reviewers (SD and DW) using the Cochrane Risk of Bias tool 2 for the primary outcome overall symptoms of schizophrenia [27]. We assessed the presence of small-study effects and publication bias for the primary outcome with funnel plots of pairwise meta-analysis if ten or more studies were included, as well as comparison-adjusted funnel plots [28, 29] ordering the antipsychotics from the newest to the oldest and from the best to the worst in efficacy [30]. Following this, we tested for the asymmetry using the Egger ’s test.

We evaluated the confidence in the relative treatment effect estimated in the network meta-analysis for the primary outcome using the Confidence in Network Meta-Analysis framework [31], implemented in the web application CINeMA [32], which allows confidence in the results to be graded as high, moderate, low, and very low. The reporting bias domain of CINeMA was evaluated by ROB-MEN (Risk of Bias due to Missing Evidence in Network meta-analysis) [33], and details are presented in Appendix 15 and 16. The study was registered in the Open Science Framework (https://osf.io/9nf2y/).

## Results

### Description of included studies

We identified 14,135 citations and included 73 studies in the qualitative synthesis. Of these, 62 studies had usable data and 60 studies involving 6838 participants were included in network meta-analysis (appendix p 12). Two studies [34, 35] were only included in pairwise meta-analysis because the interventions (paliperidone, LAI-paliperidone, LAI-clopenthixol, and LAI-perphenazine) were not connected to the network. Of 6241 participants with sex indicated, 28.54% were female. The mean age (SD) of participants was 38.07 (6.98) years; the mean (SD) duration of illness was 14.53 (5.56) years; and the mean (SD) number of previous episodes was 6.97 (3.41). The median trial duration was 12 weeks (range 4–52). One study did not provide the masking type, two studies were open-label, six studies were single-blind, and the remaining were double-blind (appendix pp 13–21). We found no clear evidence of violations of the transitivity assumption when comparing characteristics of studies across interventions (appendix pp 22–26).

According to the risk-of-bias assessment for the studies with data for the primary outcome, 23 studies had a moderate overall risk of bias, and 22 studies had a high overall risk. No study had a low overall risk (appendix pp 191–92).

### Primary outcome

**Fig. 1:**
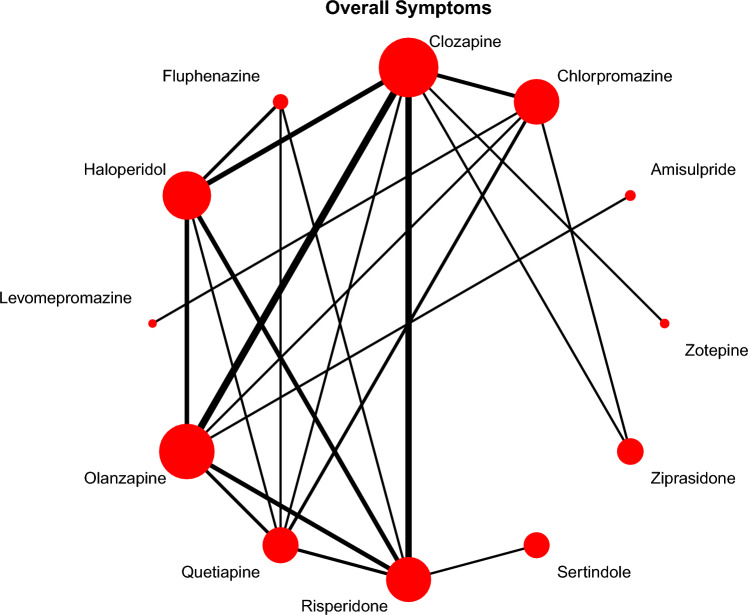
Network plot overall symptoms (primary outcome). Lines link treatments with direct comparisons in trials; thickness of lines corresponds to the number of trials evaluating the comparison; size of the nodes corresponds to the number of participants assigned to the treatment.

**Fig. 2:**
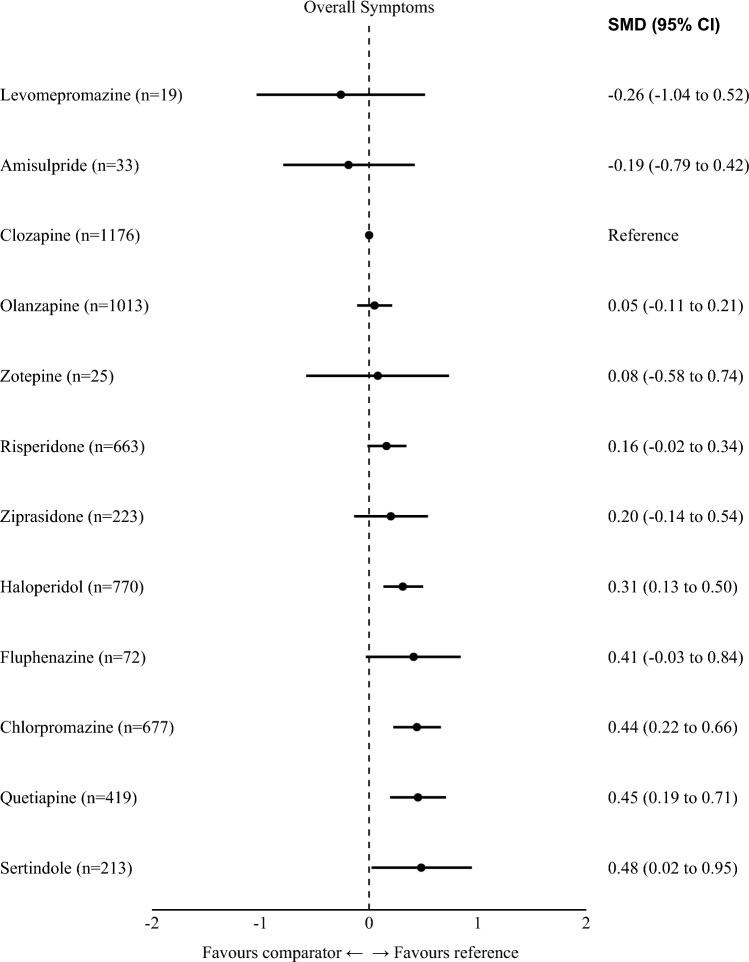
Forest plot overall symptoms (primary outcome). Numbers in parentheses are number of participants, *SMD*  standardized mean difference. *CI*  confidence interval. Reference is clozapine.

Forty-five studies with 12 interventions, involving 5303 participants, contributed to the network meta-analysis of the primary outcome of overall symptoms of schizophrenia (Fig. 1). The efficacy of clozapine and olanzapine was virtually identical. They were more effective than haloperidol, sertindole, chlorpromazine, and quetiapine, with mean SMDs ranging between small (− 0.26, olanzapine vs. haloperidol) and moderate (− 0.48, clozapine vs. sertindole, Table 2). There were no clear differences compared to zotepine, risperidone, ziprasidone, and fluphenazine, because their point estimates compared to clozapine/olanzapine were small and/or 95% CIs broadly overlapped (Fig. 2, Table 2).

**Table 2:**
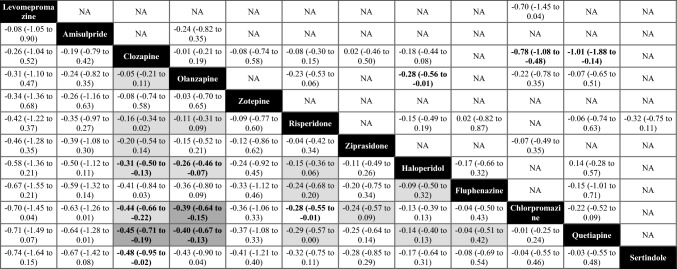
League table overall symptoms (primary outcome)

Treatments are presented in order of efficacy ranking. Results of the network meta-analysis are reported in the left lower half and results of pairwise meta-analyses in the right upper half. Each cell provides the effect estimate and the corresponding 95% credible interval (95% CI) of a comparison (left lower half: treatment in column versus treatment in row; right upper half: treatment in row versus treatment in column). The type of effect size measure is standardized mean difference (SMD). Bold results indicate 95% CI excluding no effect. For the results of the network meta-analysis, the background colors of the cells reflect confidence in the estimates, with dark gray representing moderate confidence, light gray representing low confidence, and white representing very low confidence.

*NA*  not available

In terms of rankings, levomepromazine was ranked first, followed by amisulpride, clozapine, olanzapine, and zotepine (Fig. 2). However, levomepromazine, amisulpride, and zotepine were only examined by three small sample-size studies [36–38] and did not form a single closed triangular loop in the network (Fig. 1), meaning that their rankings were almost entirely based on indirect evidence (see discussion).

Aripiprazole, perphenazine, LAI-fluphenazine, LAI-pipotiazine, and placebo were disconnected from the network. Pairwise meta-analyses of these drugs are presented in the appendix (pp 27–30).

Heterogeneity in estimates between studies of the same comparison was low to moderate (appendix p 106). Significant inconsistency was detected between direct and indirect evidence (appendix p 107). Inspection of the data showed that inconsistency was mainly owing to the chlorpromazine–clozapine comparison. We conducted a sensitivity analysis without chlorpromazine–clozapine studies [2, 7, 26] and presented the outcomes below. The confidence in the evidence of each comparison was between moderate and very low (Table 2, appendix pp 197–200).

### Positive symptoms

The results based on 39 studies with 4649 participants were similar to those based on overall symptoms (appendix pp 32–36). The noteworthy difference is risperidone, which showed no clear difference to clozapine and olanzapine and was more effective than haloperidol, chlorpromazine, and quetiapine, with 95% CIs excluding a possibility of opposite effects (mean SMDs between − 0.51 and − 0.25; appendix p 34).

### Negative symptoms

Forty-two studies with 4863 participants contributed to the results. Most negative symptom results were uncertain according to 95% CIs, except for chlorpromazine being clearly the worst and inferior to clozapine, olanzapine, quetiapine, risperidone, and haloperidol, with 95% CIs making opposite effects unlikely (appendix pp 37–41).

### Response to treatment

The network based on 46 studies (involving 20 interventions) with 6043 participants was inconsistent (12.9% inconsistent comparisons, design-by-treatment interaction test *p* = 0.02), and pairwise meta-analyses are presented in the appendix (pp 42–52).

### Study discontinuation

Based on 54 studies with 6228 participants, the ranking of all-cause discontinuation was comparable to that for overall symptoms (appendix pp 53–60). A few more interventions were included, but they had been studied with only a few participants. Clozapine, showing no difference to olanzapine, was better than chlorpromazine (RR 0.77, 95% CI 0.57 to 1.01), quetiapine (RR 0.77, 95% CI 0.53 to 1.05), haloperidol (RR 0.69, 95% CI 0.54 to 0.86), and fluphenazine (RR 0.52, 95% CI 0.22 to 0.99, appendix p 57).

Based on 48 studies with 5142 participants, clozapine ranked first and outperformed seven other interventions in decreasing the discontinuation due to inefficacy: olanzapine, risperidone, sertindole, quetiapine, haloperidol, placebo, and fluphenazine (range of mean RRs 0.14 to 0.53, 95% CIs excluding opposite effects, appendix p 65). In contrast to overall and positive symptoms, chlorpromazine was in the middle of the ranking and was equal to olanzapine (appendix p 65).

Fifty studies and 5103 participants were available for network meta-analysis of discontinuation due to adverse events. Most differences were uncertain. Only chlorpromazine was less tolerable than a number of other antipsychotics (appendix pp 69–76).

### Adverse events

In 24 studies with 2061 participants, clozapine and olanzapine were associated with less use of antiparkinsonian medication compared with risperidone, fluphenazine, and haloperidol (the worst), with 95% CIs excluding no effect (range of mean RRs 0.23 and 0.46; appendix p 81). The differences to other second-generation antipsychotics and low-potency first-generation antipsychotics were uncertain (appendix pp 77–83). Likewise, clozapine was associated with less prolactin increase than risperidone (MD− -27.41 ng/ml, 95% CI − 34.41 to − 20.42), haloperidol (MD − 17.13 ng/ml, 95% CI − 29.67 to − 4.60), chlorpromazine (MD − 13.74 ng/ml, 95% CI − 27.56 to 0.07), and olanzapine (MD − 6.37 ng/ml, 95% CI − 10.59 to − 2.15; appendix p 86).

In 30 studies with 4079 participants, clozapine was associated with more sedation than several other antipsychotics, including olanzapine, with 95% CIs making opposite effects unlikely (appendix pp 89–92).

Twenty-eight studies with 3393 participants were available for network meta-analysis of weight gain. Clozapine and olanzapine were worse than all other antipsychotics, followed by sertindole and risperidone (appendix pp 96–97).

Data on QTc prolongation were scarce and disconnected. Pairwise meta-analyses are presented in the appendix (p 100).

### Other outcomes

Seven studies with 561 participants yielded no clear differences in functioning (appendix pp 101–04). There were no inconsistent comparisons according to the SIDE test, but the design-by-treatment interaction test suggested some inconsistency of the overall network (*p* = 0.01). Heterogeneity was high (common tau = 0.77).

Eight studies provided data on quality of life, whereas conducting a network meta-analysis was not feasible. The pairwise meta-analysis indicated no clear differences among antipsychotics, with the exception of clozapine being better than haloperidol based on one study (appendix p 105).

### Heterogeneity and inconsistency

Heterogeneity and inconsistency assessments for secondary outcomes are presented in the appendix (pp 106–07). For many secondary outcomes, the networks were thin and the power was low; therefore, there might be an inconsistency that we were not able to detect.

### Sensitivity and subgroup analyses of the primary outcome

**Fig. 3:**
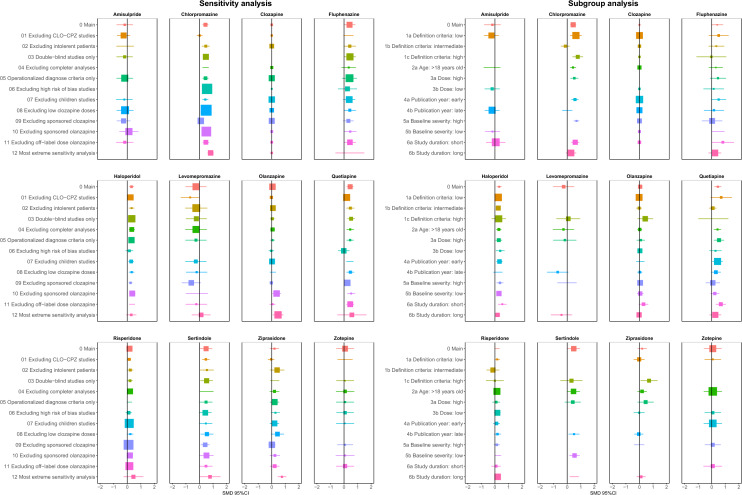
Forest plots of sensitivity and subgroup analyses. SMDs more than 0 are in favor of clozapine. *SMD*  standardized mean difference. *CI*  confidence interval. Reference is clozapine. *0 Main*  main analysis

The most important sensitivity analysis was the exclusion of the three old studies [2, 7, 26] that led to significant inconsistency. The result indicated no clear evidence of inconsistency after exclusion according to the SIDE test (5.88%) and the design-by-treatment interaction test (*p* = 0.30). However, excluding these studies did not change the ranking much, with the exception of chlorpromazine—ranking sixth here, whereas ranking last in the main analysis (appendix pp 109–10, Fig. 2). In almost all remaining sensitivity analyses, SMDs and rankings did not change considerably (Fig. 3, appendix pp 111–40). Only in the most extreme sensitivity analysis, including only situations in which clozapine may be most superior (double-blind studies with high-dose clozapine and in very refractory patients), was clozapine clearly superior to olanzapine (appendix pp 142–43).

Moreover, clozapine conserved its superiority when studies from its manufacturer were excluded (appendix pp 133–34). In contrast, olanzapine was less efficacious than clozapine and several other drugs when studies from its manufacturer were excluded (appendix pp 136–37). However, the remaining olanzapine sample size was small (*n* = 157).

In the subgroup analyses, the results of the overall symptoms were similar to the main analysis (Fig. 3, appendix pp 144–90), with the exception of olanzapine, which failed to show any clear superiority to other antipsychotics in stringently treatment-resistant patients (appendix p 153).

### Small-study effects

The comparison-adjusted funnel plots including all studies revealed no small-study effects, but the pairwise meta-analysis funnel plot for clozapine versus olanzapine suggested that some small studies favoring olanzapine could be missing (appendix pp 193–96).

### Confidence in the evidence according to CINeMA

Judgements about confidence in the estimates (CINeMA) ranged from moderate to very low, meaning that further research is likely to change the estimates (see color code in Table 2; details of the CINeMA assessment are presented in appendix pp 197–206).

## Discussion

To our knowledge, we provide the most comprehensive meta-analysis of all randomized-controlled trials evaluating the efficacy, acceptability, and tolerability of available antipsychotics in treatment-resistant schizophrenia. In particular, compared to a previous network meta-analysis by Samara et al. [12], 20 more RCTs were included. We found that clozapine and olanzapine were more efficacious than some other antipsychotics. However, in tolerability outcomes, clozapine and olanzapine were associated with more weight gain, and clozapine was associated with more sedation events compared to many other antipsychotics.

Clozapine has been regarded as the most efficacious antipsychotic in treatment-resistant schizophrenia patients since a pivotal publication in 1988 [2]. Clozapine was much more efficacious than the FGA chlorpromazine, with a large effect size (− 0.88). Haloperidol was subsequently shown to be inferior to clozapine as well [39–41]. However, the superiority of clozapine to other FGAs had only been demonstrated among patients with non-resistant schizophrenia [10, 42]. No direct evidence comparing clozapine and other FGAs in treatment-resistant schizophrenia patients was found. Our network meta-analysis provides indirect evidence to fill this gap, and we found that clozapine was better than all included FGAs but levomepromazine in symptoms reduction. It should be noted that levomepromazine and amisulpride ranked higher than clozapine in efficacy, but their results were based on minimal, unreliable, indirect evidence (19 and 33 participants, respectively), and their 95% CIs overlapped broadly with those of clozapine and olanzapine (Fig. 2).

Siskind et al. found in a pairwise meta-analysis that clozapine was more efficacious than SGAs in reducing psychotic symptoms in the short-term in treatment-resistant schizophrenia patients, but not in the longer term [11]. Similarly, Mizuno et al. found clozapine superior to other antipsychotics irrespectively of treatment resistance [43]. It should be noted that these reviews focused on the comparison of clozapine with groups of other antipsychotics. However, in practice, clinicians do not choose between either clozapine or just any other antipsychotic, and previous meta-analyses had shown that there are certain efficacy differences between individual antipsychotics [30]. In our analysis which focused on individual drugs, clozapine was more efficacious than quetiapine and sertindole. The superiority of clozapine compared to other SGAs, namely, zotepine, risperidone, and ziprasidone, was uncertain, because 95% confidence intervals suggested a certain possibility of opposite effects.

Most noteworthy is olanzapine, which in various efficacy outcomes performed similarly to clozapine according to both pairwise and network meta-analysis. The only exceptional outcome was study discontinuation due to inefficacy in which clozapine was clearly better than olanzapine. The exceptions in sensitivity analyses were excluding studies sponsored by olanzapine’s manufacturer (few trials remained), and the most extreme sensitivity analysis including only double-blind studies using high clozapine doses in very resistant patients. Here, clozapine ranked first and was clearly better than olanzapine, but again the remaining evidence was relatively scarce. Therefore, one important research agenda is a large trial comparing olanzapine and clozapine in patients with clear treatment-resistant schizophrenia (i.e., conforming to the high cut-off criterion). Moreover, given the current overall superiority of clozapine, another important research agenda is the use of clozapine after only one previously failed antipsychotic (see, for example, the ongoing clinical trial by Hasan et al. [44]). Such a study could eventually provide evidence that the early use of clozapine may avoid treatment resistance in at least a proportion of patients.

Apart from its promise in efficacy, clozapine is controversial due to its propensity for adverse effects. The most severe adverse drug reaction is agranulocytosis, which caused its withdrawal from the market in 1975 [45, 46]. Although the mandatory hematological monitoring made this fatal incident manageable [47], other adverse effects of clozapine, including weight gain, constipation, sedation, myocarditis, pancreatitis, and orthostatic hypotension [48], should also be considered. Clozapine was associated with most sedation in our meta-analysis. The same result was also found in patients with non-resistant schizophrenia [10, 42]. Similarly, in weight gain, clozapine and olanzapine were found to be inferior to both FGAs and SGAs, with most 95% CIs excluding no effect.

Another important issue for patients with treatment-resistant schizophrenia is functioning. Clozapine and olanzapine had better efficacy in various outcomes. However, this trend was not clear in the functioning outcome. One possible explanation is that only a few studies reported functioning (7 studies, 11.7% of all studies included in network meta-analysis) and the sample sizes in individual studies were small, resulting relatively wide confidence intervals of effect sizes and less noticeable differences between each antipsychotic in the network. Moreover, most studies were relatively short (median duration 17 weeks), and longer trials may be necessary for efficacy differences translating in better functioning. Finally, the outcome should be interpreted with caution, given the high heterogeneity and inconsistency in the network.

Our findings have several limitations. First, there was significant inconsistency in the primary outcome. The design-by-treatment test and SIDE tests suggested that this inconsistency was mainly due to a disagreement between direct and indirect evidence in the clozapine-chlorpromazine comparison. To assess this assumption, we excluded three old clozapine–chlorpromazine studies in sensitivity analysis and found that the data synthesis was free of inconsistency, but the ranking was similar except for chlorpromazine. We speculate that a cohort effect related to the old studies in this comparison may in part explain the inconsistency. Psychopharmacologic studies conducted before 1990 were found to have lower quality compared with later studies [49].

Second, we included definitions of treatment-resistant schizophrenia, which varied from partial nonresponse to very strict treatment resistance, and the patients also varied in baseline severity. Although the subgroup analyses across different criteria of treatment-resistant definitions did not reveal significant differences to the main analysis, individual antipsychotics could still perform variably in different stringency degrees of treatment resistance. Therefore, homogeneous definitions of treatment-resistant schizophrenia as they have recently been recommended [14] in future studies could improve relative consistency and transitivity in subsequent reviews. Including patients with similar treatment-resistant characteristics makes study results comparable and contributes higher feasibility to replicate findings. Once clinical trials achieve homogeneity on patients with “real” treatment resistance, clinical guidelines could recommend more precise and specific treatment for the group.

Third, although our search was comprehensive and included both published and unpublished studies, the pairwise meta-analysis funnel plot indicated a potential publication bias.

Fourth, the available evidence for many comparisons was based on a few trials, or only one study in some cases, which resulted in thinly connected networks and low statistical power to detect possible differences. Additionally, interventions that are not connected to the network in closed loops are prone to outlying results. This concern, for example, relates to levomepromazine and amisulpride, which were on top of the hierarchy but almost entirely based on indirect evidence. Therefore, the interpretation of results must be made cautiously and must consider the number of trials and participants for each antipsychotic and outcome.

Fifth, due to judgements as “some concerns” or “major concerns” in within-study domain and incoherence domain, the confidence in the primary outcome was mainly low and very low in the estimates according to CINeMA. This means that the results can potentially change if more evidence becomes available.

The final limitation of this study is that we only evaluated the effects of antipsychotic drugs in patients with treatment-resistant schizophrenia. It should be noted that psychological interventions, in particular cognitive behavioral therapy, are also effective for such patients [50, 51], and are also recommended by guidelines [4, 52].

## Conclusions

The results of the current network meta-analysis, together with those of observational and register-based studies [53–56] and their meta-analysis [57] in which clozapine was associated with lowest treatment failure rates and the highest effectiveness, still make clozapine the drug of choice for treatment-resistant patients. The observational data bear the limitation that clozapine-treated patients might have had superior effectiveness outcomes due to stringent monitoring to avoid agranulocytosis. Nevertheless, a trial with olanzapine, which has a very similar receptor-binding profile and was as efficacious as clozapine and more efficacious than a number of other drugs in most outcomes, might be considered before switching to clozapine and its multiple side effects. The great risk for weight gain and associated problems of both drugs must always be considered.

### Supplementary Information

Below is the link to the electronic supplementary material.

Supplementary file1 (PDF 18622 KB)

## Data Availability

The data that support the findings of this study are available from the corresponding author, Prof. Stefan Leucht, upon reasonable request.
