# Supplementary material for: A network meta-analysis of efficacy, acceptability, and tolerability of antipsychotics in treatment-resistant schizophrenia
Source: Eur Arch Psychiatry Clin Neurosci. 2023 Aug 1;274(4):917–28. doi: 10.1007/s00406-023-01654-2 (PMC11127860; doi:10.1007/s00406-023-01654-2)
Supplement: Supplementary file 1 — Supplementary file1 (PDF 18622 KB) [file 406_2023_1654_MOESM1_ESM.pdf]

APPENDIX

Appendix

1 PRISMA checklist 1

2 Protocol 4

3 Search strategy 9

4 Flow chart 12

5 Characteristics and references of included studies 13

5.1 Characteristics and references of specific studies with usable data 13

5.2 Characteristics and references of specific studies without usable data 20

6 Transitivity assessment 22

6.1 Baseline severity 22

6.2 Blinding of outcome assessor 23

6.3 Treatment-resistance definition 24

6.4 Publication year 25

6.5 Sample size 25

6.6 Mean age 26

6.7 Study duration 26

7 Forest plots of pairwise meta-analyses of the primary outcome 27

7.1 Pairwise meta-analysis of all antipsychotics 27

7.2 Post-hoc pairwise meta-analysis of first-generation antipsychotics (FGAs) vs. clozapine 29

7.3 Post-hoc pairwise meta-analysis of second-generation antipsychotics (SGAs) vs. clozapine 30

8 Results of the network meta-analyses of the secondary outcomes 31

8.1 Positive symptoms 32

8.2 Negative symptoms 37

8.3 Response 42

8.4 Discontinuation due to any reason 53

8.5 Discontinuation due to inefficacy 61

8.6 Discontinuation due to adverse events 69

8.7 Use of antiparkinsonian medication 77

8.8 Prolactin levels 84

8.9 Sedation 88

8.10 Weight gain 95

8.11 QTc prolongation 100

8.12 Functioning 101
8.13 Quality of life 105
9 Evaluation of heterogeneity 106
10 Evaluation of inconsistency 107
11 Results of the sensitivity analyses of the primary outcome 108
11.1 Excluding 3 old clozapine-chlorpromazine studies 108
11.2 Excluding studies that included intolerant patients 111
11.3 Double-blind studies only 114
11.4 Excluding studies that presented only completer analyses 117
11.5 Excluding studies that did not use operationalized criteria to diagnose schizophrenia 120
11.6 Excluding studies at high risk of bias 123
11.7 Excluding studies that only included children and/or adolescents 126
11.8 Excluding low clozapine doses (<400 mg) 129
11.9 Excluding studies from clozapine's manufacturer 132
11.10 Excluding studies from olanzapine's manufacturer 135
11.11 Excluding off-label dose olanzapine (>20mg/d) 138
11.12 A most extreme sensitivity analysis including only situations in which clozapine may be most superior 141
12 Results of the subgroup analyses of the primary outcome 144
12.1 The criteria of treatment-resistant definitions 144
12.1.1 The criteria of treatment-resistant definitions 144
12.1.2 The criteria from Samara et al[81] 154
12.2 Mean age 163
12.3 Dose of the antipsychotics in chlorpromazine-equivalents 167
12.4 Publication year 173
12.5 Baseline severity 179
12.6 Study duration 185
13 Risk of bias Assessment 191
14 Investigation of the small-study effect and publication bias 193
15 Evaluating the confidence in NMA: CINeMA 197
16 ROB-MEN 201
17 References 207

1 PRISMA checklist

Section and Topic	Item #	Checklist item	Location where item is reported
**TITLE**			
Title	1	Identify the report as a systematic review.	Page 1
**ABSTRACT**			
Abstract	2	See the PRISMA 2020 for Abstracts checklist.	Page 2
**INTRODUCTION**			
Rationale	3	Describe the rationale for the review in the context of existing knowledge.	Page 4
Objectives	4	Provide an explicit statement of the objective(s) or question(s) the review addresses.	Page 4
**METHODS**			
Eligibility criteria	5	Specify the inclusion and exclusion criteria for the review and how studies were grouped for the syntheses.	Page 5
Information sources	6	Specify all databases, registers, websites, organisations, reference lists and other sources searched or consulted to identify studies. Specify the date when each source was last searched or consulted.	Page 5
Search strategy	7	Present the full search strategies for all databases, registers and websites, including any filters and limits used.	Appendix3
Selection process	8	Specify the methods used to decide whether a study met the inclusion criteria of the review, including how many reviewers screened each record and each report retrieved, whether they worked independently, and if applicable, details of automation tools used in the process.	Page 5-6
Data collection process	9	Specify the methods used to collect data from reports, including how many reviewers collected data from each report, whether they worked independently, any processes for obtaining or confirming data from study investigators, and if applicable, details of automation tools used in the process.	Page 6
Data items	10a	List and define all outcomes for which data were sought. Specify whether all results that were compatible with each outcome domain in each study were sought (e.g. for all measures, time points, analyses), and if not, the methods used to decide which results to collect.	Page 6
	10b	List and define all other variables for which data were sought (e.g. participant and intervention characteristics, funding sources). Describe any assumptions made about any missing or unclear information.	Page 6
Study risk of bias assessment	11	Specify the methods used to assess risk of bias in the included studies, including details of the tool(s) used, how many reviewers assessed each study and whether they worked independently, and if applicable, details of automation tools used in the process.	Page 8
Effect measures	12	Specify for each outcome the effect measure(s) (e.g. risk ratio, mean difference) used in the synthesis or presentation of results.	Page 7
Synthesis methods	13a	Describe the processes used to decide which studies were eligible for each synthesis (e.g. tabulating the study intervention characteristics and comparing against the planned groups for each synthesis (item #5)).	Appendix5
	13b	Describe any methods required to prepare the data for presentation or synthesis, such as handling of missing summary statistics, or data conversions.	Page 7, Appendix6.1

Section and Topic	Item #	Checklist item	Location where item is reported
	13c	Describe any methods used to tabulate or visually display results of individual studies and syntheses.	Page 7
	13d	Describe any methods used to synthesize results and provide a rationale for the choice(s). If meta-analysis was performed, describe the model(s), method(s) to identify the presence and extent of statistical heterogeneity, and software package(s) used.	Page 7
	13e	Describe any methods used to explore possible causes of heterogeneity among study results (e.g. subgroup analysis, meta-regression).	Page 7
	13f	Describe any sensitivity analyses conducted to assess robustness of the synthesized results.	Page 8
Reporting bias assessment	14	Describe any methods used to assess risk of bias due to missing results in a synthesis (arising from reporting biases).	Page 8
Certainty assessment	15	Describe any methods used to assess certainty (or confidence) in the body of evidence for an outcome.	Page 8
**RESULTS**			
Study selection	16a	Describe the results of the search and selection process, from the number of records identified in the search to the number of studies included in the review, ideally using a flow diagram.	Page 9, Appendix4
	16b	Cite studies that might appear to meet the inclusion criteria, but which were excluded, and explain why they were excluded.	Appendix4
Study characteristics	17	Cite each included study and present its characteristics.	Appendix5
Risk of bias in studies	18	Present assessments of risk of bias for each included study.	Appendix13
Results of individual studies	19	For all outcomes, present, for each study: (a) summary statistics for each group (where appropriate) and (b) an effect estimate and its precision (e.g. confidence/credible interval), ideally using structured tables or plots.	Appendix7-8
Results of syntheses	20a	For each synthesis, briefly summarise the characteristics and risk of bias among contributing studies.	Page 9-12
	20b	Present results of all statistical syntheses conducted. If meta-analysis was done, present for each the summary estimate and its precision (e.g. confidence/credible interval) and measures of statistical heterogeneity. If comparing groups, describe the direction of the effect.	Page 9-12 and Appendix7-8
	20c	Present results of all investigations of possible causes of heterogeneity among study results.	Page 12 and Appendix9
	20d	Present results of all sensitivity analyses conducted to assess the robustness of the synthesized results.	Page 13 and Appendix11
Reporting biases	21	Present assessments of risk of bias due to missing results (arising from reporting biases) for each synthesis assessed.	Page 13 and

Section and Topic	Item #	Checklist item	Location where item is reported
			Appendix14
Certainty of evidence	22	Present assessments of certainty (or confidence) in the body of evidence for each outcome assessed.	Page 13-14 and Appendix15
**DISCUSSION**			
Discussion	23a	Provide a general interpretation of the results in the context of other evidence.	Page 14
	23b	Discuss any limitations of the evidence included in the review.	Page 17
	23c	Discuss any limitations of the review processes used.	Page 16
	23d	Discuss implications of the results for practice, policy, and future research.	Page 14-16
**OTHER INFORMATION**			
Registration and protocol	24a	Provide registration information for the review, including register name and registration number, or state that the review was not registered.	Page 8
	24b	Indicate where the review protocol can be accessed, or state that a protocol was not prepared.	Appendix2
	24c	Describe and explain any amendments to information provided at registration or in the protocol.	Page 8
Support	25	Describe sources of financial or non-financial support for the review, and the role of the funders or sponsors in the review.	Page 3
Competing interests	26	Declare any competing interests of review authors.	Page 3
Availability of data, code and other materials	27	Report which of the following are publicly available and where they can be found: template data collection forms; data extracted from included studies; data used for all analyses; analytic code; any other materials used in the review.	Page 18

2 Protocol

A Network Meta-Analysis of Efficacy, Acceptability, and Tolerability of Antipsychotics in Treatment-Resistant Schizophrenia

Review question
To examine the efficacy, acceptability and tolerability of both first- and second-generation antipsychotic drugs for treatment-resistant schizophrenia patients of all ages by applying a network meta-analysis approach.

Searches
1. Electronic databases: We will search the Cochrane Schizophrenia Group's Study-Based Register of Trials with no date/time, language, document type, and publication status limitations. This register is compiled of regular searches in multiple electronic databases, ClinicalTrials.gov, WHO register of clinical trials and more. Details on the register can be found in (Shokraneh and Adams 2017, 2019, 2020, 2021).
2. Hand searching: The Cochrane Schizophrenia Group's Study-Based Register of Trials also includes hand searches. We will additionally inspect references in previous reviews on treatment-resistant schizophrenia, as well as previous reviews from our team [1-3].
3. Personal contact: We will contact via email the first and/or corresponding authors of each included study published in the last 20 years for missing information about their studies.

Types of study to be included
We will include all randomized trials (RCTs) comparing one antipsychotic drug with another antipsychotic agent or placebo in treatment-resistant schizophrenia, but open and single-blind RCTs will be excluded in a sensitivity analysis (see below the Analysis of subgroups or subsets). Trials in which antipsychotic drugs were used as an augmentation- or combination strategy will be excluded as well as studies with a high risk of bias in the randomization process, and studies from mainland China [4]. In the case of cross-over studies we will only use the first cross-over phase to avoid the problem of carry-over effects which are very likely in schizophrenia. We will include cluster-RCTs applying the appropriate correction of the unit of analysis, or if such a correction can be applied post-hoc. But we anticipate it could be a very rare case. The minimum duration of trials will be 3 weeks.

Condition or domain being studied
Schizophrenia

Participants/population
We will include participants with a treatment-resistant form of schizophrenia, schizophreniform disorder, or schizoaffective disorder (no age limit, any definition of treatment resistance, no restriction in diagnostic criteria, setting, gender, and ethnicity).

Intervention(s), exposure(s)
We will include all antipsychotic drugs that are available in at least one country, including first-generation ("typical", "conventional") antipsychotics as well as second-generation ("atypical") antipsychotics and placebo. We will include all these compounds at any dose and in any oral forms of administration (for example tablets, liquid) or as intramuscular depot formulations. If an antipsychotic is available in both oral and depot forms, both formulations will be used as separate interventions in the network.

Comparator(s)/control
In a network meta-analysis, any treatment can be a comparator as all antipsychotics and placebo will be used to compare with each other. Placebo will be used as reference for presentation.

Context
There are no restrictions in terms of setting, for example, we will include in- and outpatients.

Main outcome(s)

The primary outcome will be the overall symptoms of schizophrenia as measured by rating scales such as the Positive and Negative Syndrome Scale (PANSS) [5], the Brief Psychiatric Rating Scale (BPRS) [6] or of any other validated scale (e.g. the Manchester Scale [7]) for the assessment of overall schizophrenic symptomatology. As not all studies will have used the same scale, we will apply the following hierarchy: mean change of the PANSS total score from baseline to endpoint, if not available mean change of the BPRS, or if again not available the mean values at endpoint of the PANSS/ BPRS. The results of other rating scales will only be used if the instrument has been published in a peer-reviewed journal, because it has been shown that unvalidated schizophrenia scales exaggerate differences [8].

Measures of effect
The effect size for the primary outcome will be the standardized mean difference (SMD), presented with their 95% CIs, because we expect different rating scales of schizophrenia symptomatology can be used in studies. We will give preference to the estimates based on imputation methods to handle missing data (used by the original authors) over completers' data.

Additional outcome(s)
1. Response to treatment. The following hierarchy of the response definitions will be applied: at least 20% reduction of the baseline score of the PANSS, 20% reduction of the BPRS or 20% reduction of any other global schizophrenia rating scale, at least "minimally improved" (score of 3) on the Clinical Global-Impressions-Improvement Scale (CGI). We choose this cutoff because even minimal improvement can be clinically important for treatment-resistant patients. If none of these definitions is available, we will use the original authors' primary definition [9].
2. Positive symptoms, measured by published rating scales.
3. Negative symptoms, measured by published rating scales.
4. Dropout due to any reason. Premature discontinuation due to any reason combines efficacy, tolerability, and other factors and can therefore be considered as a measure of "overall acceptability of treatment".
5. Dropout due to specific reasons. Dropout due to inefficacy of treatment will be considered as an additional outcome of the efficacy of treatment. Dropout due to the occurrence of adverse events will be used as a measure of overall tolerability.
6. Specific adverse events. We will focus on five most common adverse events, use of antiparkinson medication (dichotomous outcome), weight gain (kg, continuous outcome), sedation (dichotomous outcome), prolactin levels (ng/mL, continuous outcome) and QTc prolongation (ms, continuous outcome).
7. Quality of life. We will accept any published rating scale such as Heinrichs quality of life scale, Quality of Life Scale (QOLS), or any other published rating scale.
8. Functioning. Functioning will be measured by rating scales such as the Global Assessment of Functioning, the Psychosocial Performance Scale, or any other published rating scale.

Measures of effect
The effect size for continuous outcomes will be the standardized mean difference (SMD), presented with their 95% CIs, since we expect different rating scales of schizophrenia symptomatology, quality of life and functioning can be used in studies. Nevertheless, we will use mean differences (MD) for weight gain (kg), prolactin levels (ng/ml) and QTc prolongation (ms), since we can convert values of these outcomes into the same metric. We will give preference to the estimates based on imputation methods to handle missing data (used by the original authors) over completers' data.
The effect size for dichotomous outcomes will be the odds ratio (OR) and its 95% confidence intervals (CIs), because odds ratio has better mathematical properties. But we will convert back to relative risks (RRs) and percentages in treatment and control groups for presentation of the results.

Data extraction (selection and coding)
1. Selection of trials: Two reviewers will independently inspect all abstracts identified in the searches. Disagreement will be resolved by discussion, and where doubt still remains, we will acquire the full article for further inspection. Once the full articles are obtained, at least two reviewers will independently decide whether the studies meet the review criteria. If disagreement cannot be resolved by discussion, we will resolve it with a third reviewer or seek further information from the study authors.

2. Data extraction: At least two reviewers will independently extract data from all selected trials on specifically customized digital forms in the Microsoft Access database. Disagreement will be resolved by discussion with a third reviewer or by contacting the study authors.

Risk of bias (quality) assessment
Two independent reviewers will assess risk of bias of individual studies using the Cochrane Risk of Bias tool, RoB 2.0 [10].

Strategy for data synthesis
1. Two-step procedure. In a first step we will perform series of conventional pair-wise meta-analyses by combining studies that compared the same interventions. In a second step we will then perform network meta-analysis within a frequentist framework [11]. The analysis and presentation of results will be performed using R (meta and netmeta packages).
2. The heterogeneity (variability in relative treatment effects within the same treatment comparison) will be measured with the tau-squared (the variance of the random effects distribution). The heterogeneity variance will be assumed common across the various treatment comparisons and we will compare the empirical distribution with predictive distributions [12, 13]. Potential reasons for heterogeneity will be explored by subgroup analysis (see below the Analysis of subgroups or subsets).
3. Assessment of the transitivity. Intransitive networks can lead to misleading estimates. Therefore, we will assess the transitivity assumption by investigating the distribution of clinical and methodological variables that can act as effect modifiers across treatment comparisons. Potential effect modifiers are listed under “Analysis of subgroups or subsets “below. We will investigate if these variables are similarly distributed across studies grouped by comparison.
4. Network meta-analysis**.** We will conduct a random effects network meta-analysis to synthesize all evidence for each outcome, and obtain a comprehensive ranking of all treatments. Treatments will be ranked for each outcome using P-scores [14]. A key assumption is coherence, meaning the agreement between direct and indirect evidence. This will be assessed locally for each closed loop using the separating indirect from direct evidence approach, and globally for the whole network using a design-by-treatment interaction model. In case incoherence, we will investigate possible sources of it (mistakes in data entry, clear differences in study characteristics) and utilize analytical approaches [15]. If the requirements of network meta-analysis are not met (low likelihood of transitivity and/or large unexplained inconsistency) we will use pairwise meta-analysis for data synthesis.
5. Assessment of the confidence in the evidence. The confidence in the relative treatment effects for the primary outcome will be evaluated using the Confidence in Network Meta-Analysis framework [16, 17], implemented in the web application (http://cinema.ispm.ch/model/CINeMA_paper.pdf). This tool evaluates the credibility of the findings across the domains of within-study bias, across-study bias, indirectness, imprecision, heterogeneity and incoherence.

Analysis of subgroups or subsets
The following potential effect moderators of the primary outcome will be explored by subgroup analyses:
1. The criteria of treatment-resistant definitions
2. Mean participant age
3. Dose of the antipsychotics in chlorpromazine-equivalents according to Gardner et al. [18]
4. Publication date (to address the effect of possibly generally decreasing effect sizes over time)
5. Severity of illness at baseline (PANSS or BPRS score at baseline)
6. Study duration

Sensitivity analyses will be performed as follow:
1. Exclusion of non-double-blind studies (open and single-blind studies)
2. Exclusion of studies that presented only completer analyses
3. Exclusion of studies that did not use operationalized criteria to diagnose schizophrenia
4. Exclusion of studies with an overall assessment of high risk of bias
5. Exclusion of studies only included children and/or adolescents

Contact details for further information
Stefan Leucht

stefan.leucht@tum.de

Organisational affiliation of the review

Department of Psychiatry and Psychotherapy, School of Medicine, Technical University of Munich, Munich, Germany
https://www.tum.de/

Review team members and their organisational affiliations

Ms Shimeng Dong. Department of Psychiatry and Psychotherapy, School of Medicine, Technical University of Munich, Munich, Germany
Dr Johannes Schneider-Thoma. Department of Psychiatry and Psychotherapy, School of Medicine, Technical University of Munich, Munich, Germany
Mr Spyridon Siafis. Department of Psychiatry and Psychotherapy, School of Medicine, Technical University of Munich, Munich, Germany
Mr Dongfang Wang. Department of Psychiatry and Psychotherapy, School of Medicine, Technical University of Munich, Munich, Germany
Professor Stefan Leucht. Department of Psychiatry and Psychotherapy, School of Medicine, Technical University of Munich, Munich, Germany

Type and method of review
Network meta-analysis

Anticipated or actual start date
01 June 2021

Anticipated completion date
31 June 2022

Funding sources/sponsors

China Scholarship Council (CSC)

Language
English

Country
Germany

References

1. Huhn, M., et al., *Comparative efficacy and tolerability of 32 oral antipsychotics for the acute treatment of adults with multi-episode schizophrenia: a systematic review and network meta-analysis.* Lancet, 2019. **394**(10202): p. 939-951.
2. Samara, M.T., et al., *Efficacy, Acceptability, and Tolerability of Antipsychotics in Treatment-Resistant Schizophrenia: A Network Meta-analysis.* JAMA Psychiatry, 2016. **73**(3): p. 199-210.
3. Krause, M., et al., *Efficacy, acceptability, and tolerability of antipsychotics in children and adolescents with schizophrenia: A network meta-analysis.* Eur Neuropsychopharmacol, 2018. **28**(6): p. 659-674.
4. Wu, T., et al., *Randomized trials published in some Chinese journals: how many are randomized?* Trials, 2009. **10**: p. 46.
5. Kay, S.R., A. Fiszbein, and L.A. Opler, *The positive and negative syndrome scale (PANSS) for schizophrenia.* Schizophr Bull, 1987. **13**(2): p. 261-76.
6. Overall, J.E. and D.R. Gorham, *The Brief Psychiatric Rating Scale.* Psychological Reports, 1962. **10**(3): p. 799-812.
7. Krawiecka, M., D. Goldberg, and M. Vaughan, *A standardized psychiatric assessment scale for rating chronic psychotic patients.* Acta Psychiatr Scand, 1977. **55**(4): p. 299-308.

8. Marshall, M., et al., *Unpublished rating scales: a major source of bias in randomised controlled trials of treatments for schizophrenia.* Br J Psychiatry, 2000. **176**: p. 249-52.
9. Leucht, S., et al., *Linking the PANSS, BPRS, and CGI: clinical implications.* Neuropsychopharmacology, 2006. **31**(10): p. 2318-25.
10. Sterne, J.A.C., et al., *RoB 2: a revised tool for assessing risk of bias in randomised trials.* BMJ, 2019. **366**: p. l4898.
11. Rücker, G., *Network meta-analysis, electrical networks and graph theory.* Res Synth Methods, 2012. **3**(4): p. 312-24.
12. Turner, R.M., et al., *Predicting the extent of heterogeneity in meta-analysis, using empirical data from the Cochrane Database of Systematic Reviews.* Int J Epidemiol, 2012. **41**(3): p. 818-27.
13. Rhodes, K.M., R.M. Turner, and J.P. Higgins, *Predictive distributions were developed for the extent of heterogeneity in meta-analyses of continuous outcome data.* J Clin Epidemiol, 2015. **68**(1): p. 52-60.
14. Rücker, G. and G. Schwarzer, *Ranking treatments in frequentist network meta-analysis works without resampling methods.* BMC Med Res Methodol, 2015. **15**: p. 58.
15. Salanti, G., *Indirect and mixed-treatment comparison, network, or multiple-treatments meta-analysis: many names, many benefits, many concerns for the next generation evidence synthesis tool.* Res Synth Methods, 2012. **3**(2): p. 80-97.
16. Nikolakopoulou, A., et al., *CINeMA: An approach for assessing confidence in the results of a network meta-analysis.* PLoS Med, 2020. **17**(4): p. e1003082.
17. Salanti, G., et al., *Evaluating the quality of evidence from a network meta-analysis.* PLoS One, 2014. **9**(7): p. e99682.
18. Gardner, D.M., et al., *International consensus study of antipsychotic dosing.* Am J Psychiatry, 2010. **167**(6): p. 686-93.

3 Search strategy

We searched the register of the Cochrane Schizophrenia Group.

Following the methods from Cochrane,[1] the Information Specialist compiles this register from systematic searches of major resources and their monthly updates (unless otherwise specified):

1. MEDLINE;
2. Embase;
3. Allied and Complementary Medicine (AMED);
4. Cumulative Index to Nursing and Allied Health Literature (CINAHL);
5. PsycINFO;
6. PubMed;
7. US National Institute of Health Ongoing Trials Register ClinicalTrials.gov;
8. World Health Organization International Clinical Trials Registry Platform (www.who.int/ictrp);
9. ProQuest Dissertations and Theses A&I and its quarterly update;
10. Chinese databases (Chinese Biomedical Literature Database, China Knowledge Resource Integrated Database, and Wanfang) and their annual updates until the end of 2016.

The register also includes hand searches and conference proceedings (see Group's website: http://schizophrenia.cochrane.org/register-trials). It does not place any limitations on language, date, document type or publication status. Further information about the register has been published by Shokraneh et al.[2–5]

The search strategy of the first search (27/04/2020) was:

((*Amisulpride* OR *Aripiprazole* OR *Asenapine* OR *Benperidol* OR *Brexpiprazole* OR *Cariprazine* OR *Chlorpromazine* OR *Clopenthixol* OR *Clozapine* OR *Flupentixol* OR *Fluphenazine* OR *Fluspirilene* OR *Haloperidol* OR *Iloperidone* OR *Levomepromazine* OR *Loxapine* OR *Lumateperone* OR *Lurasidone* OR *Molindone* OR *Olanzapine* OR *Paliperidone* OR *Penfluridol* OR *Perazine* OR *Perphenazine* OR *Pimozide* OR *Quetiapine* OR *Risperidone* OR *Sertindole* OR *Sulpiride* OR *Thioridazine* OR *Tiotixene* OR *Trifluoperazine* OR *Ziprasidone* OR *Zotepine* OR *Zuclopenthixol*) in Intervention Field of Study)

The second and the third update searches of the Cochrane Schizophrenia Group Register were made on September 19, 2021 and March 06, 2022, respectively. Both searches were not restricted to the antipsychotic drugs above, all controlled trials of schizophrenia were inspected. Apart from this all descriptions of the register etc. above applied.

The last update search was conducted in MEDLINE (20/01/2023) with the following search strategy:

Database: Ovid MEDLINE(R) and Epub Ahead of Print, In-Process, In-Data-Review & Other Non-Indexed Citations and Daily <1946 to January 20, 2023>

Search Strategy:
1 randomized controlled trial.pt. (584874)
2 controlled clinical trial.pt. (95158)
3 randomized.ab. (590240)
4 placebo.ab. (235122)
5 clinical trials as topic.sh. (200778)
6 randomly.ab. (400293)
7 trial.ab. (632640)
8 or/1-7 (1692527)
9 exp animals/ not humans.sh. (5084723)
10 8 not 9 (1552536)
11 exp SCHIZOPHRENIA/ (113837)
12 exp Paranoid Disorders/ (4248)
13 schizo$.mp. (187748)
14 hebephreni$.mp. (287)
15 oligophreni$.mp. (1146)
16 psychotic$.mp. (77111)
17 psychosis.mp. (45087)
18 psychoses.mp. (21437)
19 ((chronic$ or sever$) adj2 mental$ adj2 (ill$ or disorder$)).mp. (10531)
20 exp dyskinesia, drug-induced/ (7370)
21 exp psychomotor agitation/ (6916)
22 exp neuroleptic malignant syndrome/ (2143)
23 exp "diagnosis, dual (psychiatry)"/ (3725)
24 (tardiv$ adj dyskine$).mp. (4583)
25 akathisi$.mp. (3065)
26 acathisi$.mp. (14)
27 (neuroleptic$ and (malignant adj2 syndrome)).mp. (2883)
28 (neuroleptic$ and (movement and disorder$)).mp. (983)
29 parkinsoni$.mp. (34289)
30 neuroleptic-induc$.mp. (1276)
31 or/24-30 (44295)
32 31 not (parkinson's adj1 disease).ti. (36572)
33 or/11-23 (273796)
34 or/32-33 (300026)
35 and/10,34 (23251)

36 Amisulpride/ (792)
37 Aripiprazole/ (2818)
38 Asenapine.mp. (486)
39 Benperidol/ (799)
40 Brexpiprazole.mp. (316)
41 Cariprazine.mp. (360)
42 Chlorpromazine/ (17378)
43 Clopenthixol/ (408)
44 Clopenthixol/ (408)
45 Flupenthixol/ (927)
46 Fluphenazine/ (2427)
47 Fluspirilene/ (113)
48 Haloperidol/ (16016)
49 Iloperidone.mp. (234)

50 Methotrimeprazine/ or Levomepromazine.mp. (1031)
51 Loxapine/ (319)
52 Lumateperone.mp. (54)
53 Lurasidone Hydrochloride/ (344)
54 Molindone/ (145)
55 Olanzapine/ (6157)
56 Paliperidone Palmitate/ (1016)
57 Penfluridol/ (179)
58 Perazine/ (158)
59 Perphenazine/ (1593)
60 Pimozide/ (1741)
61 Quetiapine Fumarate/ (3107)
62 Risperidone/ (6709)
63 Sertindole.mp. (491)
64 Sulpiride/ (4029)
65 Thioridazine/ (2393)
66 Thiothixene/ (335)
67 Trifluoperazine/ (3614)
68 Ziprasidone.mp. (2135)
69 Zotepine.mp. (303)
70 Zuclopenthixol.mp. (313)
71 or/36-70 (64470)

72 limit 71 to yr="2022 - 2023" (928)
73 and/35,72 (100)

Although Pipotiazine was not part of our initial search strategy, we decided to include it in this study because it was a comparator in a three-armed trial.[6]

4 Flow chart

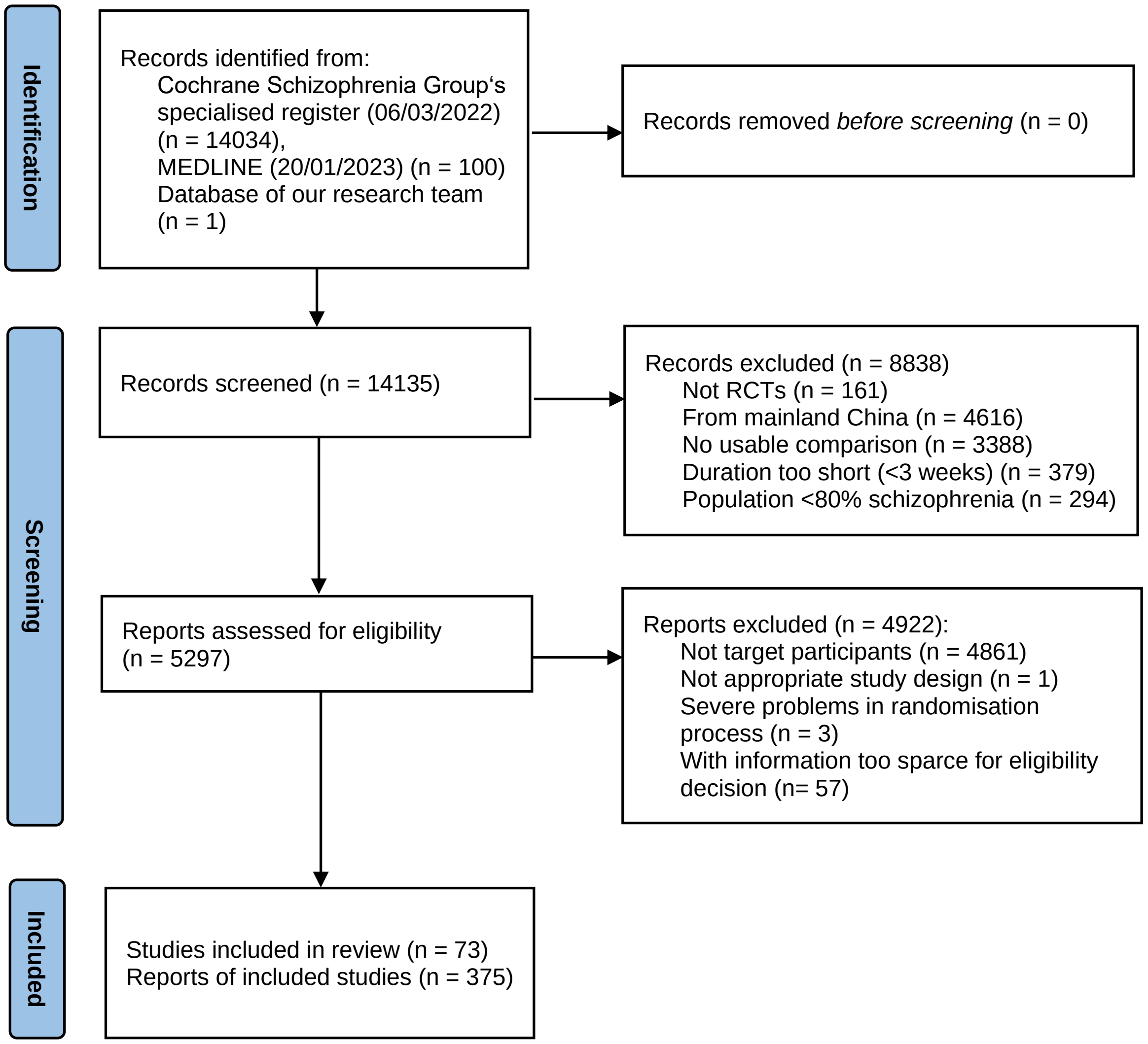

Reference from the database of our research team.[7] RCTs = randomized controlled trials.

5 Characteristics and references of included studies

5.1 Characteristics and references of specific studies with usable data

Study	Duration (weeks)	Study Design	Diagnosis	Definition of treatment-resistance	Intervention	Application, interval, mean dose in mg (range)	Randomized N	Mean age
Actrn1261800 1113246[8]	26	OL-RCT	Schizophrenia (DSM-V)	Unsuccessful treatment with 1 oral antipsychotic	Paliperidone	Depot, 4 weeks, n.i. (50-150)	36	46.4
					Paliperidone	Oral, daily, n.i. (6-12)	36	46.4
Ahlfors 1980[9]	26	DB-RCT	Schizophrenia (clinical diagnosis)	Unsatisfactory response to present neuroleptic treatment	Clopenthixol	Depot, 2 weeks, 280 (50-280)	87	n.i.
					Perphenazine	Depot, 2 weeks, 141 (20-600)	85	n.i.
Altamura 2002[10]	14	DB-RCT	Paranoid schizophrenia (DSM-IV)	partial response to previous treatments, at least 2 retrospective trials	Haloperidol	Oral, daily, 12.3 (5-20)	15	38.3
					Olanzapine	Oral, daily, 12.4 (10-20)	13	39.3
Alvarez 2006[11]	52	OL-RCT	Schizophrenia (DSM-IV)	Prominent negative symptoms and previously treated with conventional antipsychotics	Olanzapine	Oral, daily, 12.2 (10-n.i.)	124	37
					Risperidone	Oral, daily, 4.9 (3-n.i.)	123	35.5
AstraZeneca 5077IL/0031[12]	10	DB-RCT	Schizophrenia (DSM-IV)	Historical and prospective criteria of treatment-resistance	Quetiapine	Oral, daily, 571.2 (n.i. -750)	130	41
					Chlorpromazine	Oral, daily, 1040.4 (n.i. -1500)	130	40.8
AstraZeneca 5077IL/0054[13]	10	DB-RCT	Schizophrenia (DSM-IV)	Treatment-resistance, at least 2 retrospective trials	Quetiapine	Oral, daily, 600 (600-600)	117	39
					Chlorpromazine	Oral, daily, 900 (900-900)	119	39
Azorin 2001[14]	12	DB-RCT	Schizophrenia (DSM-IV)	Poor response to previous treatment, at least 2 retrospective trials	Clozapine	Oral, daily, 642 (200-900)	138	38.2
					Risperidone	Oral, daily, 9 (2-15)	135	39.4
Bitter 2004[15]	18	DB-RCT	Schizophrenia (DSM-IV)	Treatment-resistance or intolerance, at least 1 retrospective trial	Clozapine	Oral, daily, 216.2 (100-500)	72	37.4
					Olanzapine	Oral, daily, 17.2 (5-25)	75	37.6
Bondolfi 1998[16]	8	DB-RCT	Schizophrenia (DSM-III-R)	Treatment-resistance or intolerance, at least 2	Clozapine	Oral, daily, 291.2 (150-400)	43	36.2
					Risperidone	Oral, daily, 6.4 (3-10)	43	38.3

				retrospective trials				
Breier 1999a[17]	6	DB-RCT	Schizophrenia (DSM-IV)	Partial response to neuroleptics, at least 1 retrospective and 1 prospective trials	Clozapine	Oral, daily, 403.6 (200-600)	14	37.7
					Risperidone	Oral, daily, 5.9 (2-9)	15	32.4
Breier 1999b[18]	6	DB-RCT	Schizophrenia, schizophreniform disorder, or schizoaffective disorder (DSM-III-R)	Treatment-resistance, at least 1 retrospective trial	Olanzapine	Oral, daily, 11.1 (5-20)	352	38.3
					Haloperidol	Oral, daily, 10 (5-20)	174	38.2
Browne 1988[19]	20	DB-RCT	Schizophrenia (Feighner's criteria, 1972)	Treatment-resistance, at least 1 retrospective trial	Haloperidol	Oral, daily, n.i. (10-160)	6	40.88
					Placebo	Oral, daily	5	40.88
Buchanan 1998[20]	10	DB-RCT	Schizophrenia or schizoaffective disorder (DSM-III-R)	Partial response to conventional neuroleptics, at least 2 retrospective and 1 prospective trials	Clozapine	Oral, daily, 415 (200-600)	38	41
					Haloperidol	Oral, daily, 25.2 (10-30)	37	40.1
Buchanan 2005[21]	16	DB-RCT	Schizophrenia or schizoaffective disorder (DSM-IV)	Partial response to conventional neuroleptics, at least 2 retrospective and 1 prospective trials	Haloperidol	Oral, daily, 18.3 (10-30)	34	46.4
					Olanzapine	Oral, daily, 20.3 (10-30)	29	41.9
Chen 2012[22]	8	SB-RCT	Schizophrenia (DSM-IV)	Inadequate response to only 1 line of first-generation antipsychotics, 1 retrospective trial	Olanzapine	Oral, daily, 17.8 (n.i. -20)	16	37.9
					Risperidone	Oral, daily, 4.3 (n.i. -6)	16	36.7
Chowdhury 1999[23]	16	n.i.	Schizophrenia and it subtypes (ICD-10)	Inadequate response, at least 1 retrospective trial	Clozapine	Oral, daily, 342.86 (200-500)	30	30.3
					Risperidone	Oral, daily, 5.8 (4-8)	30	32.43
Claus 1992[24]	12	DB-RCT	Schizophrenia (DSM-III-R)	Treatment-resistance, at least 1 retrospective trial	Haloperidol	Oral, daily, 10.3 (10.3-10.3)	22	39
					Risperidone	Oral, daily, 12 (12-12)	22	37.4
Conley 1998[25]	8	DB-RCT	Schizophrenia (DSM-III-R)	Treatment-resistance, at least 2 retrospective and 1 prospective trials	Chlorpromazine	Oral, daily, 1200 (1200-1200)	42	42.63
					Olanzapine	Oral, daily, 25 (25-25)	42	42.91

Conley 2003[26]	8	DB-RCT	Schizophrenia (DSM-IV)	Treatment-resistance, at least 2 retrospective trials	Clozapine	Oral, daily, 390 (300-450)	5	40.26
					Olanzapine	Oral, daily, 50 (30-50)	8	35.91
Conley 2005[27]	12	DB-RCT	Schizophrenia (DSM-IV)	Treatment-resistance, at least 2 retrospective and 1 prospective trials	Fluphenazine	Oral, daily, 13.2 (10-15)	13	44.2
					Quetiapine	Oral, daily, 463.6 (300-500)	12	43.7
					Risperidone	Oral, daily, 4.31 (3-5)	13	46.3
Daniel 1996[28]	6	SB-RCT	Schizophrenia or schizoaffective disorder (DSM-III-R)	Multiple past treatment failures or intolerance of conventional antipsychotic side effects	Clozapine	Oral, daily, n.i. (n.i. - n.i.)	10	33.8
					Risperidone	Oral, daily, n.i. (n.i. - n.i.)	10	33.8
Dean 1958[29]	15	DB-RCT	Schizophrenia (clinical diagnosis)	Fail to respond to previous treatment	Chlorpromazine	Oral, daily, n.i. (2000-3000)	9	37
					Placebo	Oral, daily	9	37
Emsley 2000[30]	8	DB-RCT	Schizophrenia (DSM-IV)	History of unsuccessful antipsychotic therapy, at least 1 retrospective and 1 prospective trials	Haloperidol	Oral, daily, 20 (20-20)	145	38.8
					Quetiapine	Oral, daily, 600 (600-600)	143	37.7
Hall 1968[31]	12	DB-RCT	Schizophrenia (clinical diagnosis)	Treatment-resistance, at least 2 retrospective trials	Haloperidol	Oral, daily, 6.95 (2-10)	25	45
					Fluphenazine	Oral, daily, 17.86 (5-25)	25	45
Hong 1997[32]	12	DB-RCT	Schizophrenia (DSM-IV)	Treatment-resistance, at least 2 retrospective and 1 prospective trials	Clozapine	Oral, daily, 543 (100-900)	21	39.7
					Chlorpromazine	Oral, daily, 1163 (200-1800)	19	37.1
Honigfeld 1984[33]	4	DB-RCT	Schizophrenia (DSM-II)	Poor response to previous therapy or sensitivity to extra-pyramidal side effects, at least 2 retrospective trials	Clozapine	Oral, daily, 417 (150-900)	75	30
					Chlorpromazine	Oral, daily, 795 (300-1800)	76	30
Howard 1974[34]	12	DB-RCT	Chronic psychosis (clinical diagnosis)	Treatment-resistance	Haloperidol	Oral, daily, 61.57 (10-133.33)	17	46.6
					Placebo	Oral, daily	16	47.8
					Tiotixene	Oral, daily, 184.7 (30-400)	16	45.3
Kahn 2018[35]	6	DB-RCT	Schizophrenia, schizophreniform disorder, or schizoaffective disorder	Not in remission from 1 prospective trial	Amisulpride	Oral, daily, 590.9 (200-800)	47	24.9
					Olanzapine	Oral, daily, 15.6 (5-20)	46	24.6

			(DSM-IV)					
Kane 1988[36]	6	DB-RCT	Schizophrenia (DSM-III)	Treatment-resistance, at least 3 retrospective and 1 prospective trials	Clozapine	Oral, daily, 600 (500-900)	126	36.2
					Chlorpromazine	Oral, daily, 1200 (1000-1800)	142	35.6
Kane 2001[37]	29	DB-RCT	Schizophrenia or schizoaffective disorder (DSM-III-R)	Partial or poor response, at least 3 retrospective trials or 2 retrospective and 1 prospective trials	Clozapine	Oral, daily, 523 (200-800)	37	41
					Haloperidol	Oral, daily, 18.9 (4-16)	34	40
Kane 2006[38]	12	DB-RCT	Schizophrenia (DSM-III-R)	Treatment-resistance, at least 3 retrospective and 1 prospective trials	Chlorpromazine	Oral, daily, 743.6 (200-1200)	154	34.4
					Ziprasidone	Oral, daily, 153.8 (80-160)	152	35.6
Kane 2007[39]	6	DB-RCT	Schizophrenia (DSM-IV)	Treatment-resistance, at least 2 retrospective and 1 prospective trials	Aripiprazole	Oral, daily, 28.8 (15-30)	154	42.6
					Perphenazine	Oral, daily, 39.1 (8-64)	146	41.6
Kane 2011[40]	12	DB-RCT	Schizophrenia (DSM-IV)	Treatment-resistance, at least 1 retrospective and 1 prospective trials	Risperidone	Oral, daily, 9 (6-12)	105	38.8
					Sertindole	Oral, daily, 18.1 (12-24)	216	38.9
Kinon 1993b[41]	4	DB-RCT	Schizophrenia, schizoaffective disorder, or schizophreniform disorder (DSM-III-R)	Treatment-resistance, non-response to 1 prospective trial	Haloperidol	Oral, daily, 20 (20-20)	13	n.i.
					Fluphenazine	Oral, daily, 48.24 (20-80)	34	n.i.
Kinon 2009[42]	12	DB-RCT	Schizophrenia, schizoaffective disorder, or schizophreniform disorder (DSM-IV)	Treatment-resistance, non-response to 1 prospective trial	Risperidone	Oral, daily, n.i. (2-6)	192	41.87
					Olanzapine	Oral, daily, n.i. (10-20)	186	41.84
Kumra 1996[43]	6	DB-RCT	Schizophrenia (DSM-III-R)	Non-response or/and intolerance, at least 2 retrospective trials	Clozapine	Oral, daily, 176 (6.25-525)	10	14.4
					Haloperidol	Oral, daily, 16 (0.25-27)	11	13.73
Kumra 2007[44]	12	DB-RCT	Schizophrenia or	Treatment-resistance, at least 2 retrospective trials	Clozapine	Oral, daily, 403.1 (50-700)	18	15.8

			schizoaffective disorder (DSM-IV)		Olanzapine	Oral, daily, 26.2 (10-30)	21	15.5
Lal 2006[45]	15	DB-RCT	Schizophrenia (DSM-III-R)	Treatment-resistance, at least 3 retrospective and 1 prospective trials	Chlorpromazine	Oral, daily, 762 (600-1000)	19	39.4
					Levomepromazine	Oral, daily, 813 (600-1000)	19	38.5
Marjerrison 1964[46]	30	DB-RCT	Chronic psychosis (clinical diagnosis)	Treatment-resistance	Placebo	Oral, daily	34	47
					Trifluoperazine	Oral, daily, 28 (n.i. – n.i.)	16	49
McCreadie 1977[47]	12	DB-RCT	Schizophrenia (clinical diagnosis)	Treatment-resistance	Chlorpromazine	Oral, daily, 350 (100-600)	10	52
					Haloperidol	Oral, daily, 57.5 (15-100)	10	52
McEvoy 2006[48]	26	DB-RCT (Clozapine OL)	Schizophrenia (DSM-IV)	Non-response to prior atypical antipsychotic treatment	Olanzapine	Oral, daily, 23.4 (7.5-30)	19	44.3
					Quetiapine	Oral, daily, 642.9 (200-800)	15	37.1
					Risperidone	Oral, daily, 4.8 (1.5-6)	16	37.7
					Clozapine	Oral, daily, 332.1 (n.i. – n.i.)	49	39.4
Meltzer 2008[49]	26	DB-RCT	Schizophrenia or schizoaffective disorder (DSM-IV)	Treatment-resistance, at least 2 retrospective trials	Clozapine	Oral, daily, 564 (300-900)	21	37.2
					Olanzapine	Oral, daily, 33.6 (25-45)	19	36.4
Mercer 1997[50]	9	SB-RCT	Schizophrenia (DSM-III-R)	Treatment-resistance	Chlorpromazine	Oral, daily, 500 (50-850)	12	37.3
					Risperidone	Oral, daily, 8 (4-16)	15	42.5
Meyer-Lindenberg 1997[51]	6	DB-RCT	Schizophrenia (DSM-III-R)	Treatment-resistance or intolerance, at least 2 retrospective trials	Clozapine	Oral, daily, 300 (150-450)	25	33.2
					Zotepine	Oral, daily, 300 (150-450)	25	33.7
Moresco 2004[52]	8	DB-RCT	Schizophrenia (DSM-IV)	Treatment-resistance, at least 2 retrospective trials	Clozapine	Oral, daily, 325.4 (300-400)	12	38.3
					Olanzapine	Oral, daily, 18.3 (15-20)	11	34.1
Naber 2005[53]	26	DB-RCT	Schizophrenia (DSM-IV)	Non-response or intolerance, at least 1 retrospective trial	Clozapine	Oral, daily, 209.4 (100-400)	57	35.2
					Olanzapine	Oral, daily, 16.2 (5-25)	57	32.9
Rosenheck 1997[54]	52	DB-RCT	Schizophrenia (DSM-III-R)	Treatment-resistance, at least 2 retrospective trials	Clozapine	Oral, daily, 552 (100-900)	205	43.2
					Haloperidol	Oral, daily, 28 (5-30)	218	43.9
Sacchetti 2009[55]	18	DB-RCT	Schizophrenia (DSM-IV)	Treatment-resistance or/and intolerance, at least 3	Clozapine	Oral, daily, 345.7 (250-600)	74	38.3
					Ziprasidone	Oral, daily, 130.4 (80-160)	73	41.6

				retrospective trials				
Schiele 1961, 06602[56]	16	DB-RCT	Schizophrenia (clinical diagnosis)	Treatment-resistance	Chlorpromazine	Oral, daily, 894 (200-1000)	20	40.6
					Placebo	Oral, daily	20	40.6
					Thioridazine	Oral, daily, 958 (200-1000)	20	40.6
					Trifluoperazine	Oral, daily, 35 (10-50)	20	40.6
Schlosberg 1978[6]	52	DB-RCT	Schizophrenia (clinical diagnosis)	Treatment-resistance	Fluphenazine	Depot, 4 weeks, 27.5 (6.25-50)	30	43
					Pipotiazine	Depot, 4 weeks, 27.5 (6.25-50)	30	45
					Placebo	Depot	15	39.87
Schooler 2016[57]	29	DB-RCT	Schizophrenia or schizoaffective disorder (DSM-IV)	Partial or poor response, at least 2 retrospective trials	Clozapine	Oral, daily, 456.7 (500-800)	53	42
					Risperidone	Oral, daily, 6.8 (6-16)	54	42
See 1999[58]	5	DB-RCT	Schizophrenia (DSM-IV)	Partial response to typical antipsychotics, at least 1 retrospective and 1 prospective trials	Haloperidol	Oral, daily, n.i. (15-30)	10	37.7
					Risperidone	Oral, daily, n.i. (4-6)	10	33.5
Shalev 1993[59]	4	SB-RCT	Schizophrenia (DSM-III)	Treatment-resistance	Haloperidol	Oral, daily, 29.3 (n.i. – n.i.)	18	33
					Perphenazine	Oral, daily, 35.8 (n.i. – n.i.)	21	33
					Levomepromazine	Oral, daily, 379 (n.i. – n.i.)	21	33
Shaw 2006[60]	8	DB-RCT	Schizophrenia (DSM-IV)	Insufficient response, at least 2 retrospective trials	Clozapine	Oral, daily, 327 (150-500)	12	11.7
					Olanzapine	Oral, daily, 18.1 (5-20)	13	12.8
Sirota 2006[61]	12	SB-RCT	Schizophrenia (DSM-IV)	Inadequate response, at least 2 retrospective trials	Olanzapine	Oral, daily, 16 (5-20)	21	36.2
					Quetiapine	Oral, daily, 637.2 (200-800)	19	38.3
Smith 2001[62]	8	DB-RCT	Schizophrenia or schizoaffective psychosis (criteria n.i.)	Poor clinical response, at least 2 retrospective trials	Haloperidol	Oral, daily, 37.9 (5-40)	18	43
					Olanzapine	Oral, daily, 19.9 (5-20)	20	43
Suzuki 2007[63]	4	OL-RCT	Schizophrenia (DSM-IV)	Non-response to 1 prospective trial	Olanzapine	Oral, daily, n.i. (n.i. - 20)	15	44.9
					Quetiapine	Oral, daily, n.i. (n.i. - 750)	10	44.9
					Risperidone	Oral, daily, n.i. (n.i. - 12)	12	44.9

Tollefson 2001[64]	18	DB-RCT	Schizophrenia (DSM-IV)	Treatment-resistance, at least 2 retrospective trials	Clozapine	Oral, daily, 303.6 (200-600)	90	38.6
					Olanzapine	Oral, daily, 20.5 (15-25)	90	38.6
Toru 1972[65]	8	DB-RCT	Schizophrenia (clinical diagnosis)	Non-response to conventional psychotropic	Chlorpromazine	Oral, daily, n.i. (150-600)	37	35
					Sulpiride	Oral, daily, n.i. (300-1200)	38	35
Volavka 2002[66]	14	DB-RCT	Schizophrenia or schizoaffective disorder (DSM-IV)	Suboptimal response, at least 1 retrospective trial	Clozapine	Oral, daily, 526.6 (200-800)	40	42.6
					Haloperidol	Oral, daily, 25.7 (10-30)	37	37.3
					Olanzapine	Oral, daily, 30.4 (10-40)	39	41
					Risperidone	Oral, daily, 11.6 (4-16)	41	42.9
Wahlbeck 2000[67]	10	SB-RCT	Schizophrenia (DSM-IV)	Treatment-resistance, at least 2 retrospective and 1 prospective trials	Clozapine	Oral, daily, 385 (25-600)	11	35.7
					Risperidone	Oral, daily, 7.8 (2-10)	9	36.8
Wirshing 1999[68]	8	DB-RCT	Schizophrenia (DSM-III-R)	Treatment-resistance or intolerance, at least 3 retrospective trials	Haloperidol	Oral, daily, 19.4 (5-30)	33	40
					Risperidone	Oral, daily, 7.5 (3-15)	34	41

5.2 Characteristics and references of specific studies without usable data

Study	Duration (weeks)	Study Design	Diagnosis	Definition of treatment-resistant	Intervention	Application, interval, mean dose in mg (range)	Randomized N	Mean age
Addington 1996[69]	6	DB-RCT	Schizophrenia (DSM-III-R)	Treatment-resistance	Risperidone	Oral, daily, n.i. (6-10)	n.i.	n.i.
					Haloperidol	Oral, daily, n.i. (10-15)	n.i.	n.i.
Byerly 1999[70]	12	SB-RCT	Schizophrenia (criteria n.i.)	Non-response to olanzapine or risperidone	Clozapine	Oral, daily, 300 (300-300)	n.i.	n.i.
					Quetiapine	Oral, daily, 400 (400-400)	n.i.	n.i.
Estrella 1996[71]	104	OL-RCT	schizophrenic patients	Treatment-resistance	Clozapine	Oral, n.i., n.i. (n.i. - n.i.)	n.i.	n.i.
					Risperidone	Oral, n.i., n.i. (n.i. - n.i.)	n.i.	n.i.
Green 2004[72]	104	OL-RCT	Schizophrenia or schizoaffective disorder (criteria n.i.)	Treatment-resistance to 2 trials of typical neuroleptics or 1 trial of atypical antipsychotic	Clozapine	Oral, n.i., n.i. (n.i. - n.i.)	n.i.	n.i.
					Olanzapine	Oral, n.i., n.i. (n.i. - n.i.)	n.i.	n.i.
Hamilton 1960[73]	8	DB-RCT	Schizophrenia (clinical diagnosis)	Treatment-resistance	Chlorpromazine	Oral, daily, 300 (300-300)	18	38
					Placebo	Oral, daily	18	38
Heres 2022[74]	6	DB-RCT	schizophrenia, schizoaffective disorder or schizophreniform disorder (DSM-IV)	Not in remission from 1 prospective trial	Amisulpride	Oral, daily, n.i. (600-800)	n.i.	n.i.
					Olanzapine	Oral, daily, n.i. (15-20)	n.i.	n.i.
Heylen 1988[75]	8	DB-RCT	Chronic psychosis (clinical diagnosis)	Treatment-resistance	Risperidone	Oral, daily, n.i. (2-20)	n.i.	n.i.
					Haloperidol	Oral, daily, n.i. (2-20)	n.i.	n.i.
Mergl 1999[76]	6	DB-RCT	Schizophrenia (criteria n.i.)	Non-response to typical neuroleptics	Olanzapine	Oral, n.i., n.i. (n.i. - n.i.)	n.i.	n.i.
					Clozapine	Oral, n.i., n.i. (n.i. - n.i.)	n.i.	n.i.
Oliemeulen 2000[77]	8	n.i.	Schizophrenia (DSM-IV)	Treatment-resistance or intolerance	Olanzapine	Oral, n.i., n.i. (n.i. - n.i.)	21	n.i.
					Clozapine	Oral, n.i., n.i. (n.i. - n.i.)	15	n.i.
Salganik 1998[78]	10	DB-RCT	Schizophrenia (DSM-III-R)	Treatment-resistance	Clozapine	Oral, n.i., n.i. (n.i. - n.i.)	n.i.	66.6
					Haloperidol	Oral, n.i., n.i. (n.i. - n.i.)	n.i.	66.6

Teja 1975[7]	20	DB-RCT	Schizophrenia (DSM-II)	Non-response to the various treatments	Chlorpromazine	Oral, daily, n.i. (100-1080)	14	38.01
					Haloperidol	Oral, daily, n.i. (2-45)	13	38.01
					Placebo	Oral, daily	10	38.01

DB-RCT: double blind randomized controlled trial; DSM: Diagnostic and Statistical Manual of Mental Disorders; ICD: International Statistical Classification of Diseases and Related Health Problems; n.i.: not indicated; OL-RCT: open label randomized controlled trial; SB-RCT: single blind randomized controlled trial.

6 Transitivity assessment

Following the recommendations of Cochrane handbook,[79] we visually inspected the distribution of potential effect modifiers across antipsychotics based on all included studies with at least one available data.

6.1 Baseline severity

We converted BPRS-18 baseline scores of studies to PANSS equivalents based on Leucht 2013[80]. Baseline severity ranges from a minimal of 66 in PANSS equivalents to a maximum of 148. However, the PANSS equivalents could be problematic as BPRS versions were not indicated clearly in studies when converting from BPRS to PANSS.

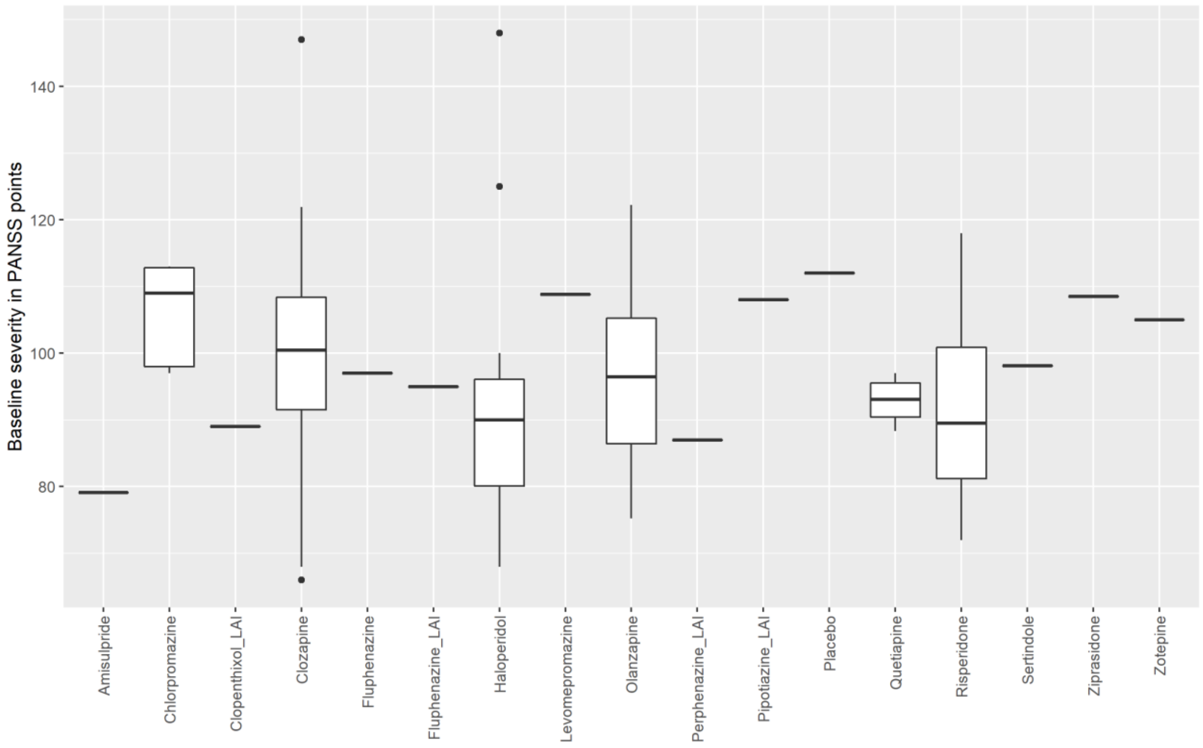

6.2 Blinding of outcome assessor

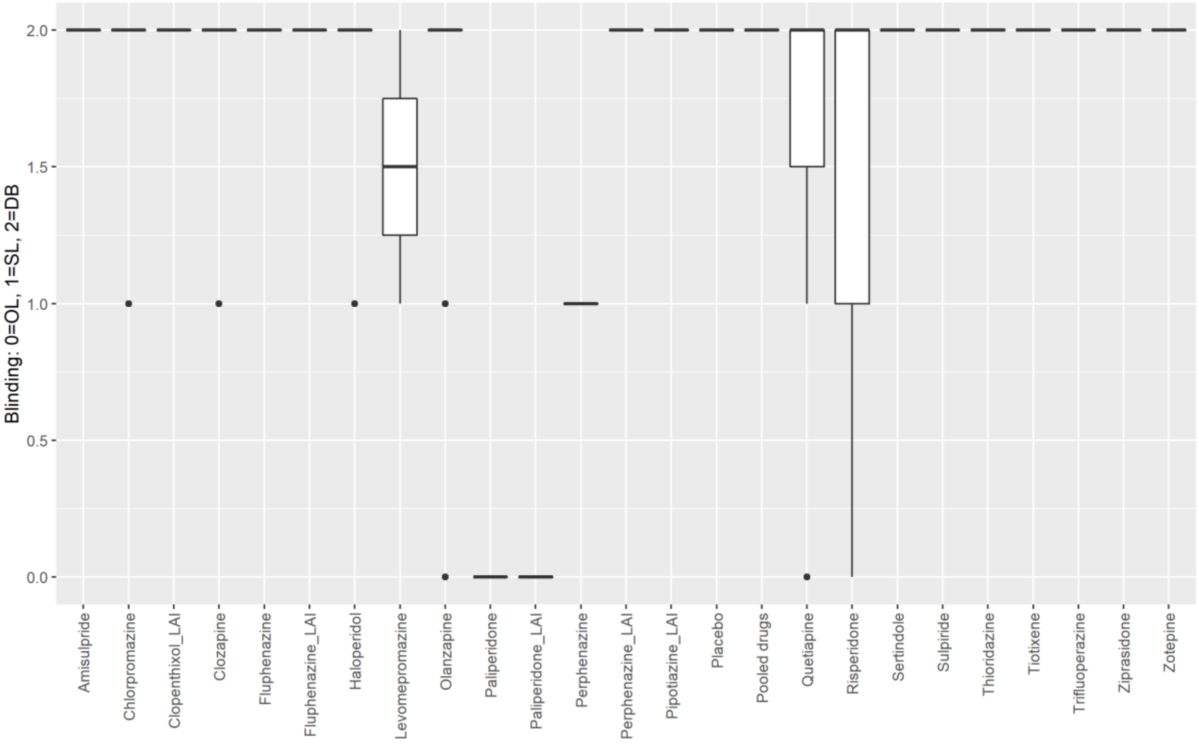

OL=open-label study; SL=single-blind study; DB=double-blind study

6.3 Treatment-resistance definition

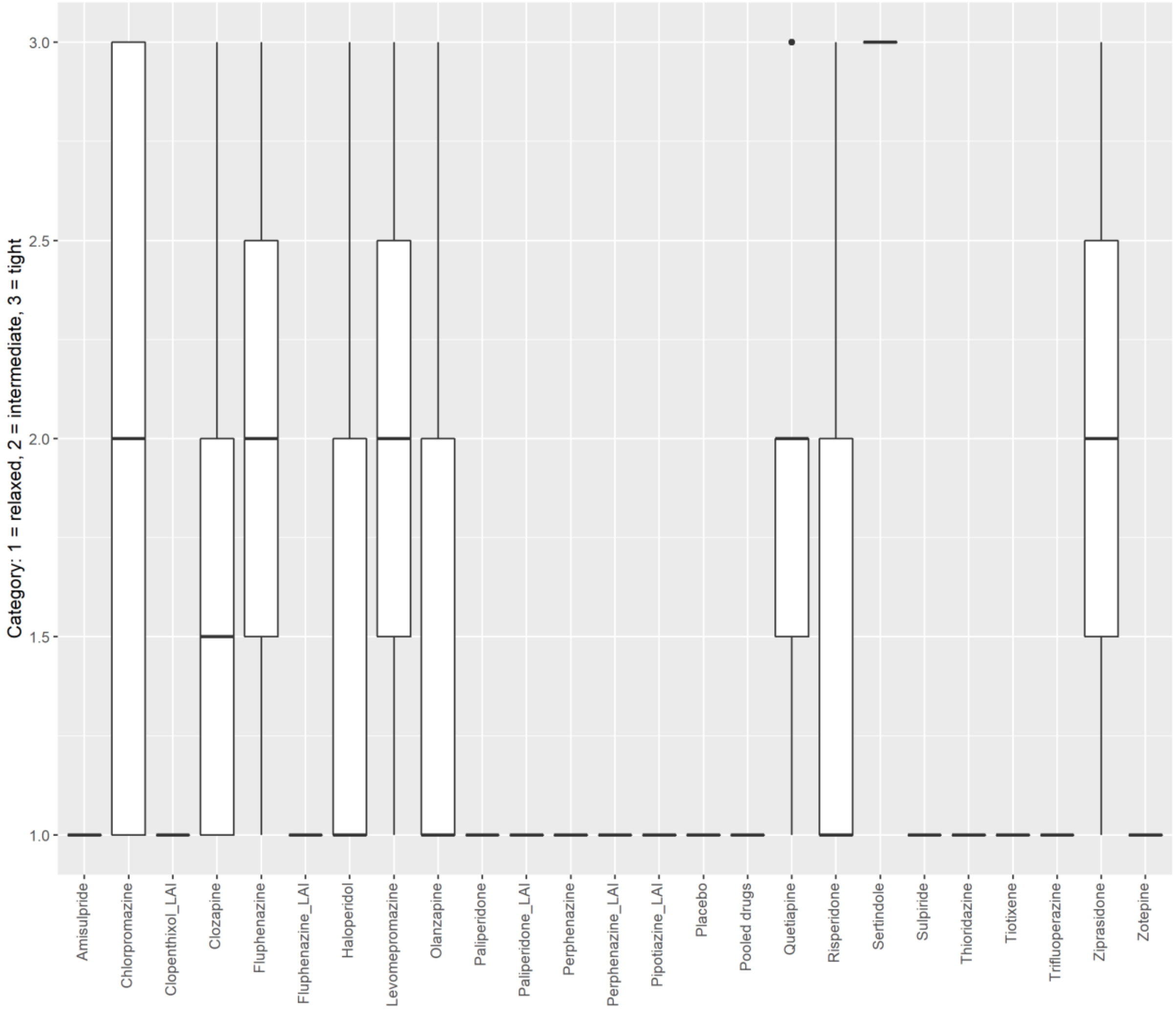

Criteria of treatment-resistance definition is presented in Appendix 12.1.1.

6.4 Publication year

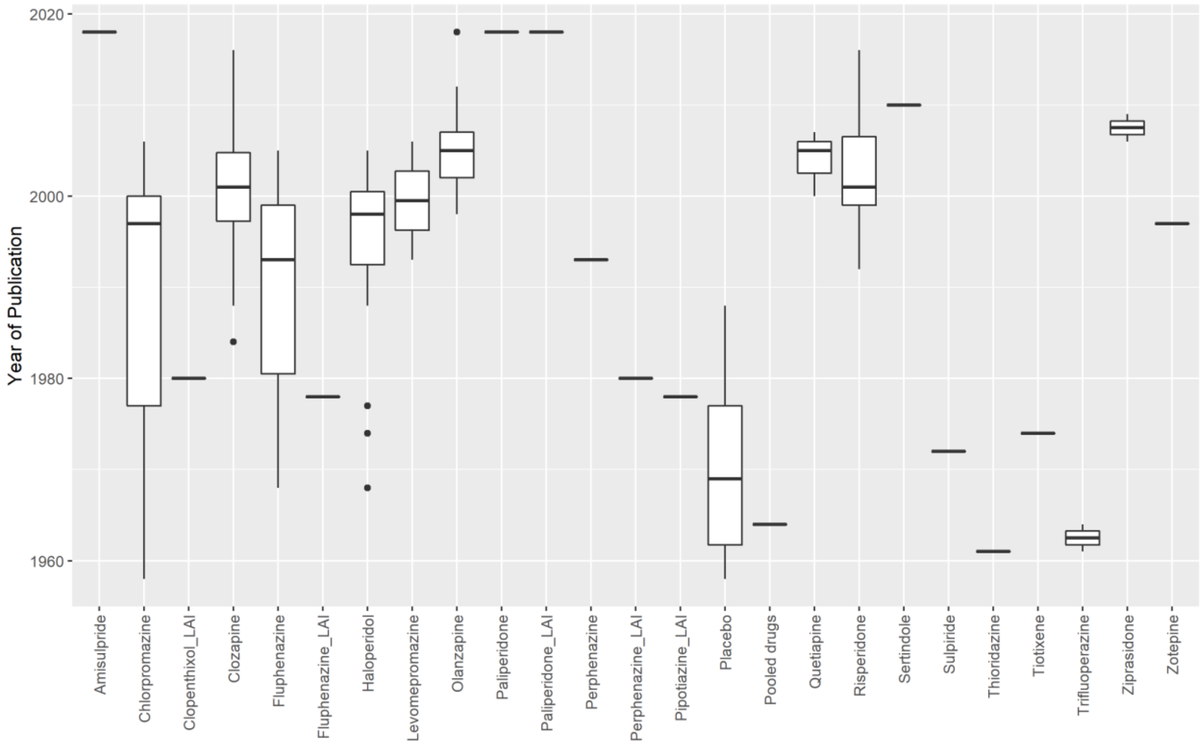

6.5 Sample size

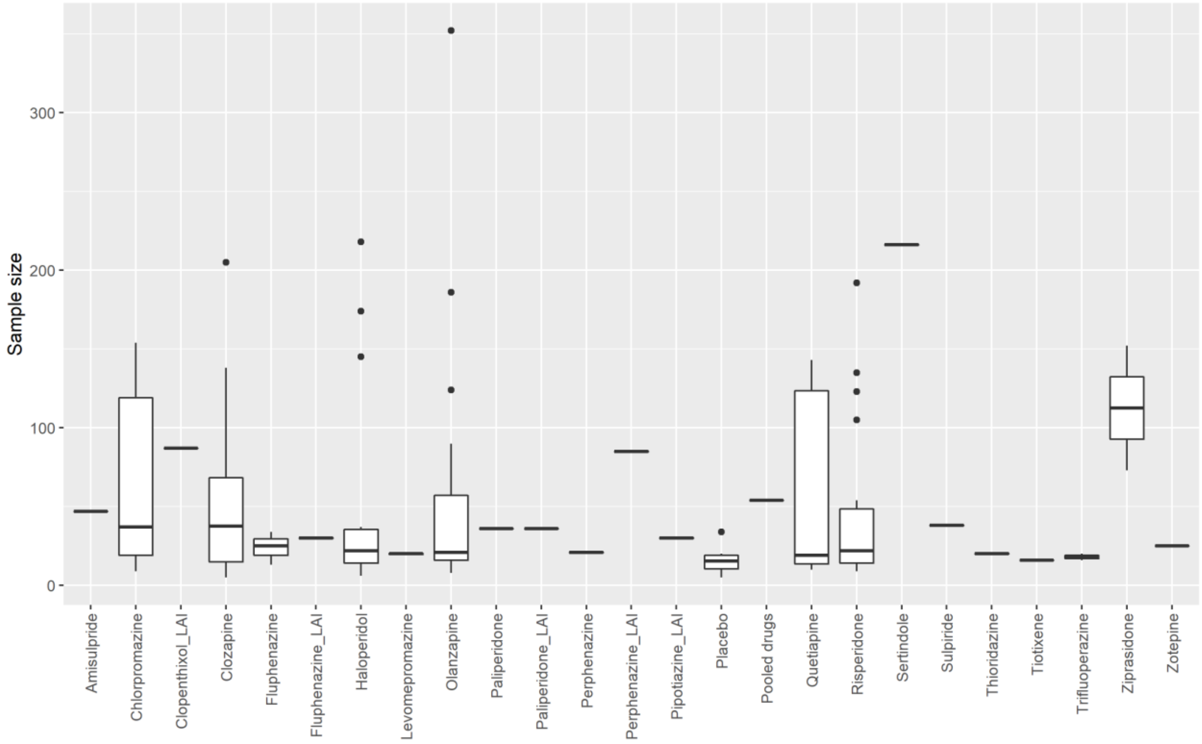

6.6 Mean age

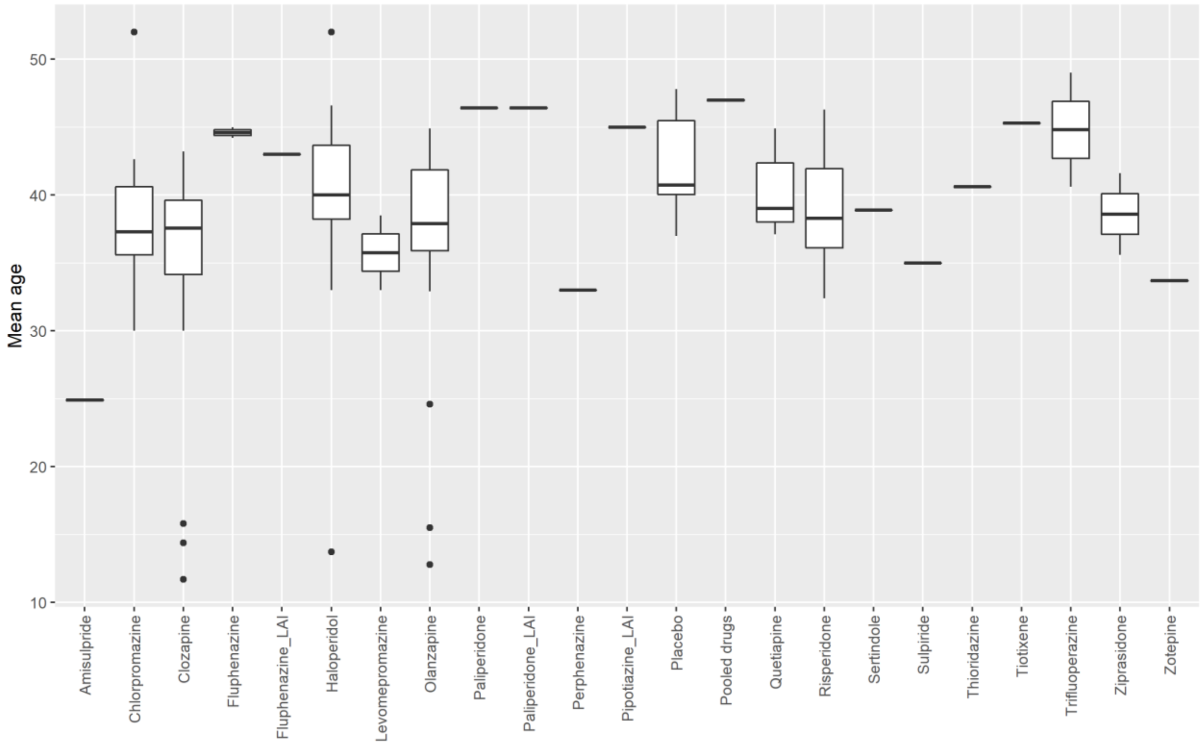

6.7 Study duration

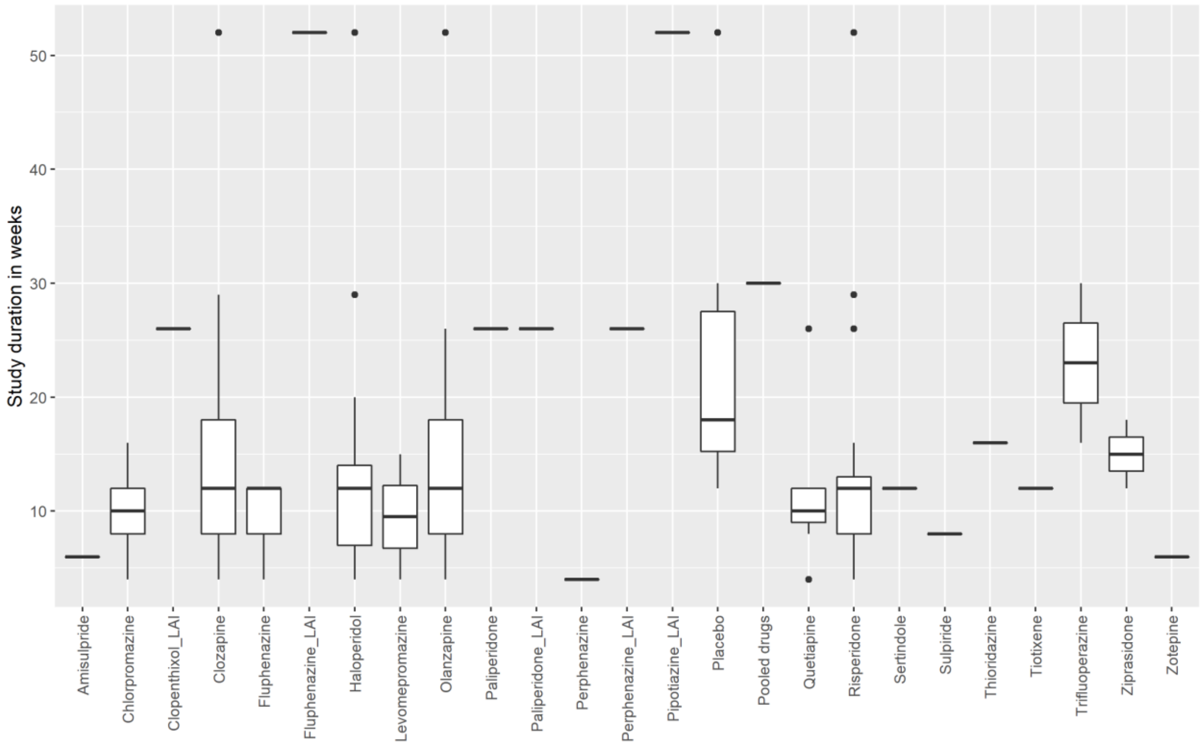

7 Forest plots of pairwise meta-analyses of the primary outcome

7.1 Pairwise meta-analysis of all antipsychotics

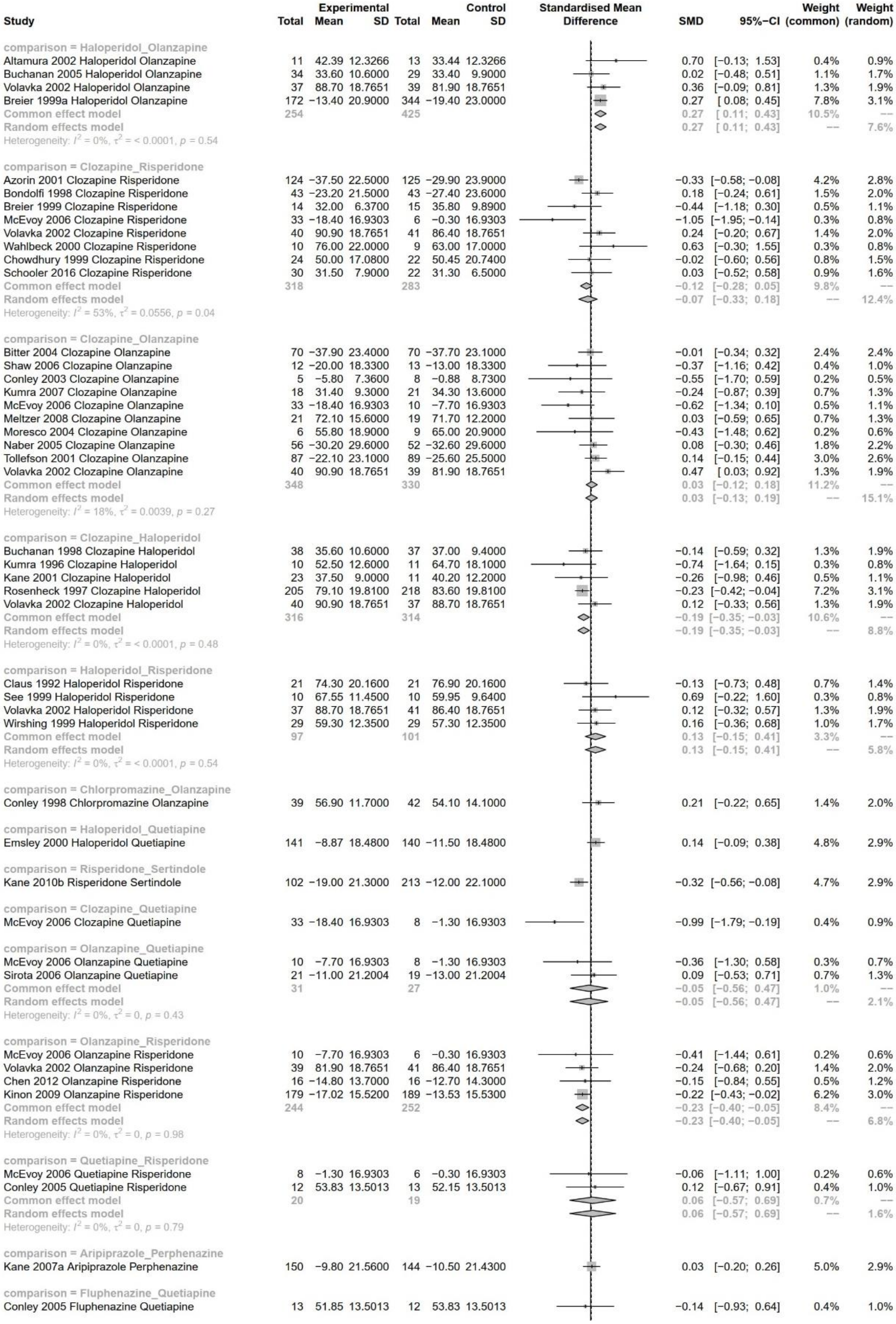

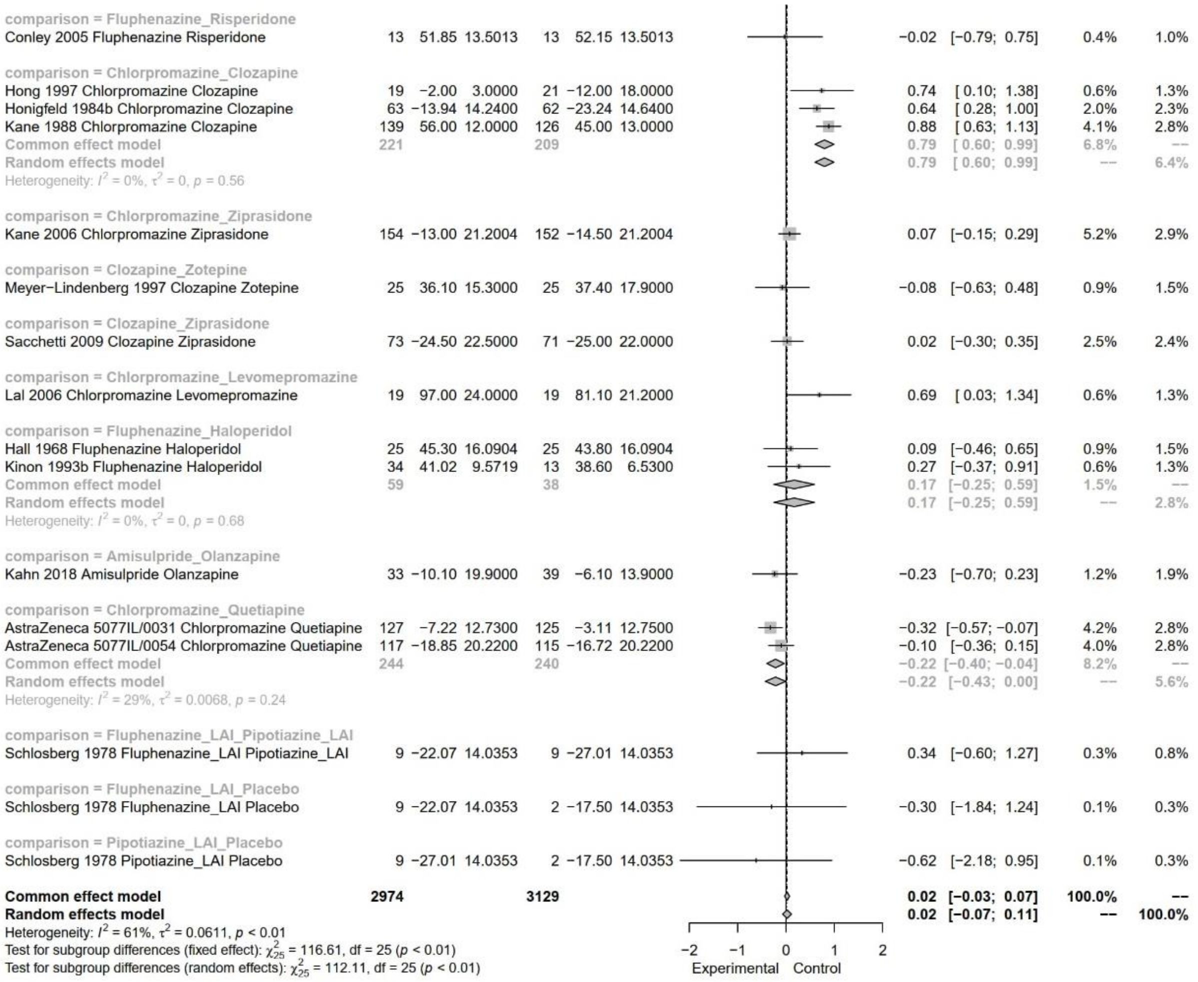

A summary effect size is calculated by pairwise meta-analyses of all studies of a specific comparison. The type of effect size measure is standardized mean difference (SMD).

7.2 Post-hoc pairwise meta-analysis of first-generation antipsychotics (FGAs) vs. clozapine

Clozapine is better only as FGAs as a group but not as SGAs as a group.

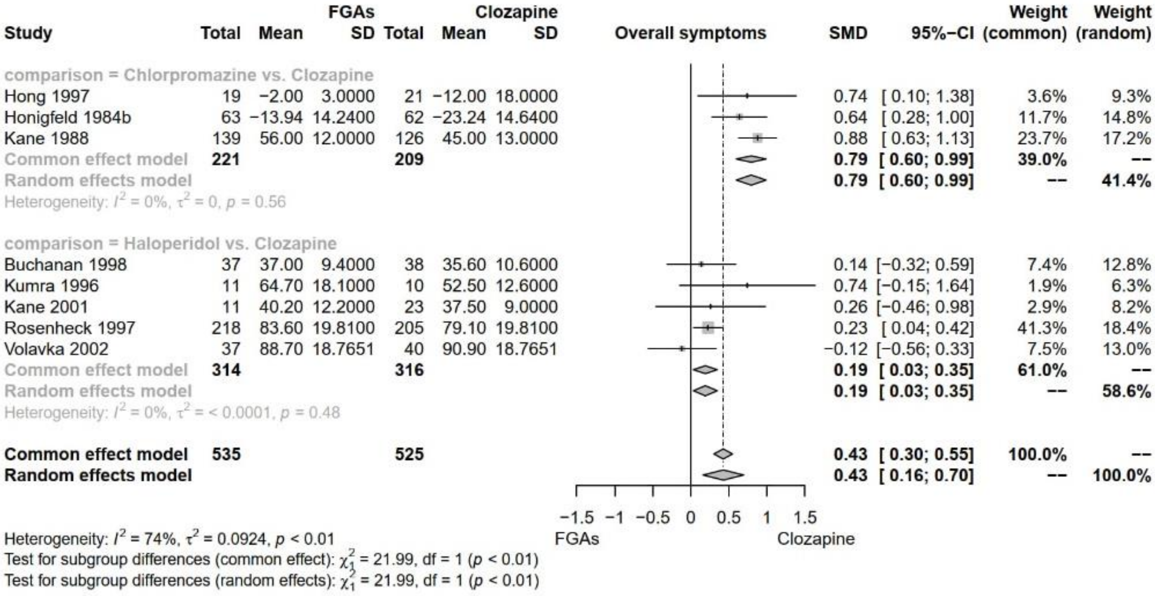

7.3 Post-hoc pairwise meta-analysis of second-generation antipsychotics (SGAs) vs. clozapine

Clozapine is better only as FGAs as a group but not as SGAs as a group.

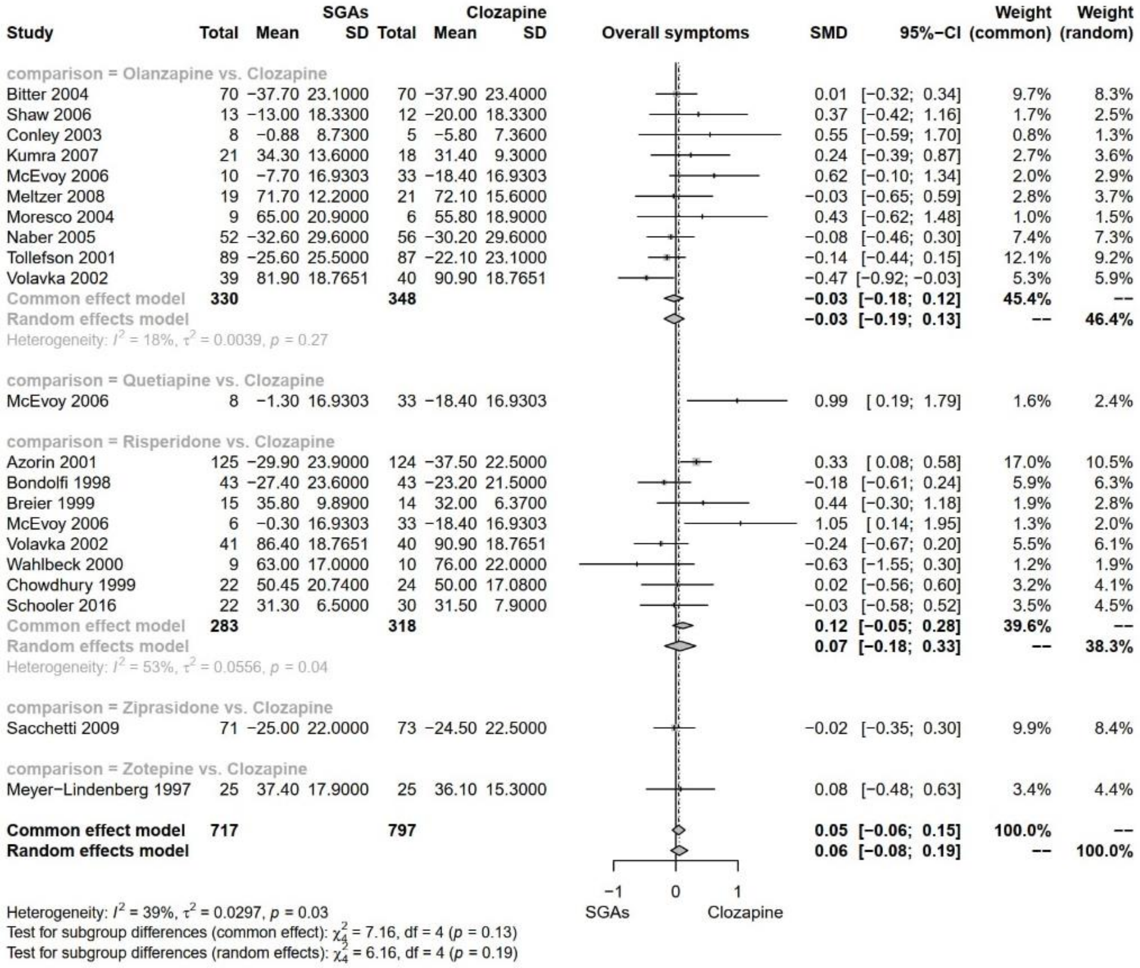

8 Results of the network meta-analyses of the secondary outcomes

Each outcome is presented in the order:

- Network plot
- Forest plot of results of network meta-analysis (clozapine as reference)
- League table
- Forest plot of results of pairwise meta-analysis

For the effect size of dichotomous outcomes, we performed the statistical analysis using odds ratios (OR) and not risk ratios (RR), because OR provide more stable estimates of treatment effects across study-conditions with different baseline risks. Therefore, it is recommended for (network-)meta-analysis. However, OR are more difficult to interpret than RR. For specific situations/patients with specific (assumed or known) baseline risks, OR can be transformed to RR or presented as exposure-event-rates (EER) versus control-event-rates (CER).

Formula for the transformations:

RR= OR/[(1-CER) +(OR*CER)]

EER= RR*CER

We present forests plots and league tables with the original results in OR, RR and EER vs. CER below (EER vs. CER in the forest plot only).

8.1 Positive symptoms

Network plot

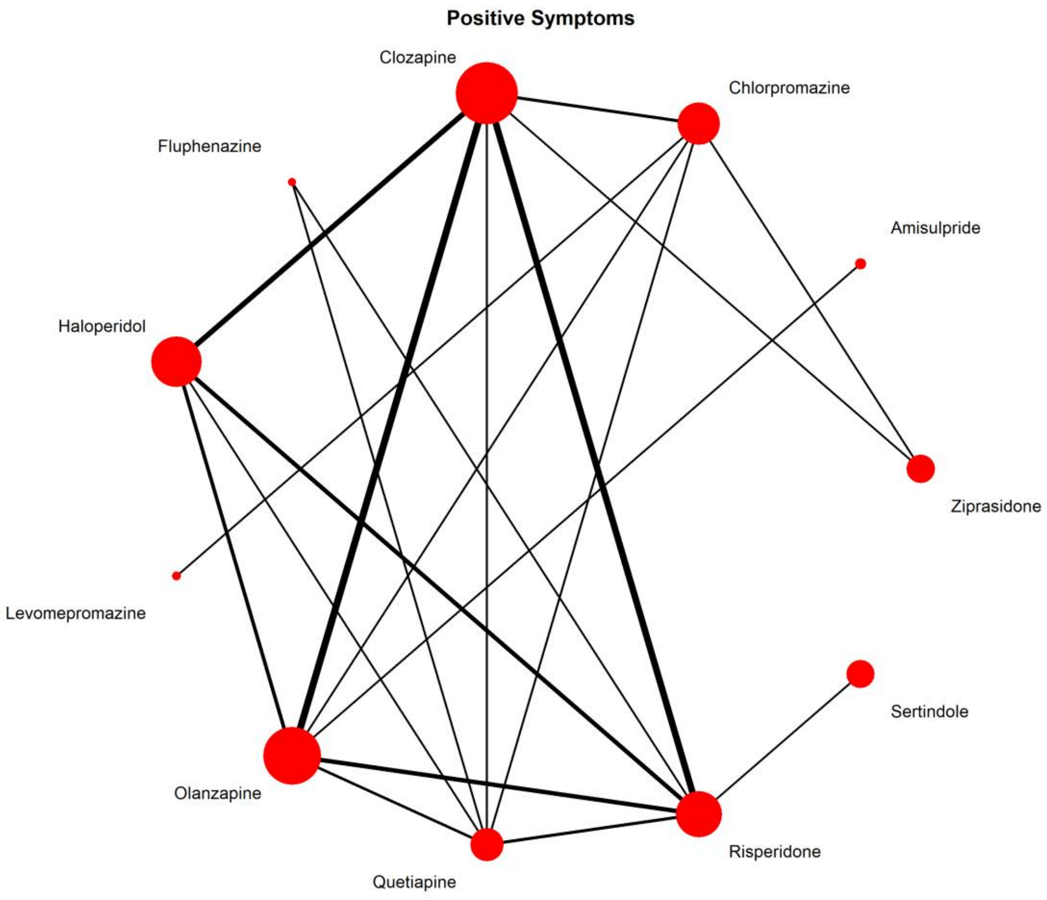

Lines link treatments with direct comparisons in trials; thickness of lines corresponds to the number of trials evaluating the comparison; size of the nodes corresponds to the number of participants assigned to the treatment.

Forest-plot of results of network meta-analysis for antipsychotic drugs versus clozapine

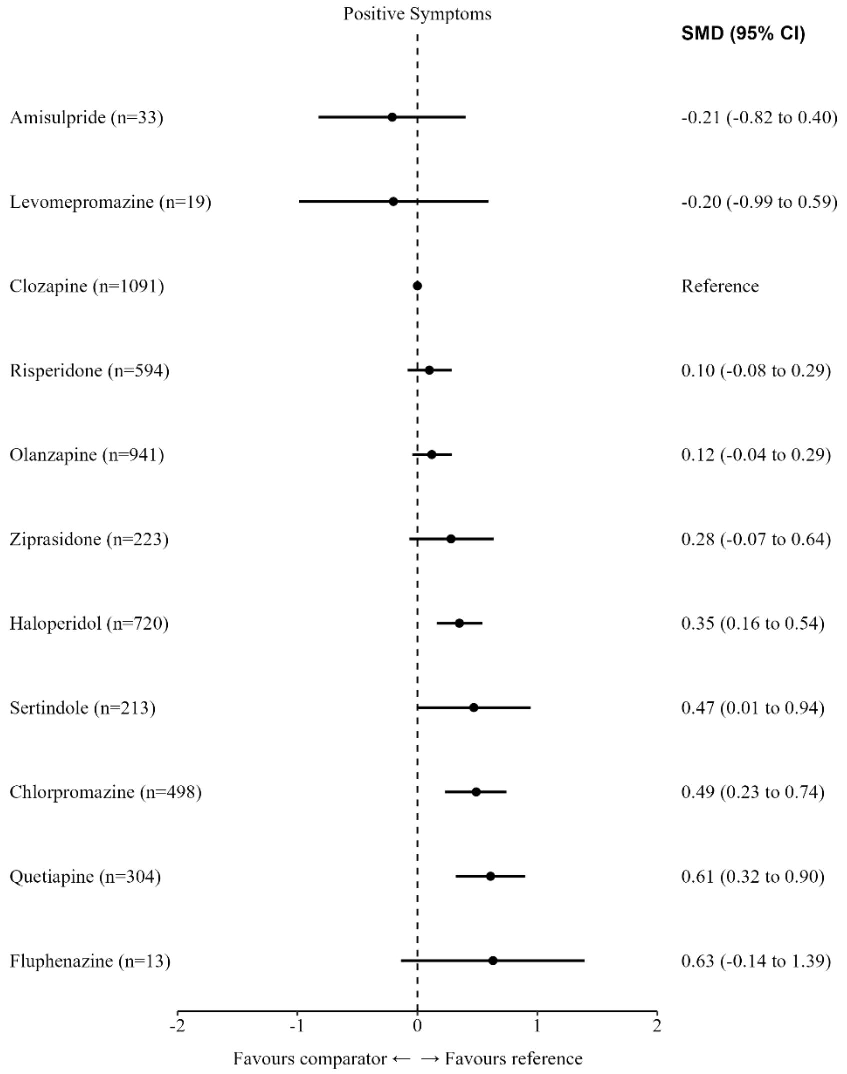

Effect sizes, measured as standardized mean difference (SMD), are from the network meta-analysis. Order of treatments is according to the mean effect size. Reference is clozapine. The direction of the effect is indicated below the x-axis.

League table for the outcome: Positive symptoms

**Amisulpride**	NA	NA	NA	-0.33 (-0.92 to 0.26)	NA	NA	NA	NA	NA	NA
-0.01 (-1.01 to 0.98)	**Levomepromazine**	NA	NA	NA	NA	NA	NA	-0.68 (-1.43 to 0.06)	NA	NA
-0.21 (-0.82 to 0.40)	-0.20 (-0.99 to 0.59)	**Clozapine**	-0.06 (-0.28 to 0.17)	-0.11 (-0.31 to 0.10)	-0.13 (-0.62 to 0.35)	-0.25 (-0.51 to 0.02)	NA	**-0.72 (-1.10 to -0.34)**	NA	**-0.97 (-1.84 to -0.10)**
-0.31 (-0.94 to 0.31)	-0.30 (-1.10 to 0.51)	-0.10 (-0.29 to 0.08)	**Risperidone**	0.10 (-0.21 to 0.40)	NA	-0.24 (-0.58 to 0.10)	-0.37 (-0.80 to 0.06)	NA	-0.77 (-1.64 to 0.09)	-0.69 (-1.38 to 0.01)
-0.33 (-0.92 to 0.26)	-0.32 (-1.12 to 0.48)	-0.12 (-0.29 to 0.04)	-0.02 (-0.22 to 0.19)	**Olanzapine**	NA	-0.14 (-0.43 to 0.15)	NA	-0.35 (-0.92 to 0.22)	NA	-0.54 (-1.13 to 0.04)
-0.50 (-1.20 to 0.20)	-0.48 (-1.30 to 0.34)	-0.28 (-0.64 to 0.07)	-0.18 (-0.57 to 0.21)	-0.16 (-0.54 to 0.21)	**Ziprasidone**	NA	NA	-0.09 (-0.51 to 0.34)	NA	NA
-0.56 (-1.19 to 0.06)	-0.55 (-1.35 to 0.25)	**-0.35 (-0.54 to -0.16)**	**-0.25 (-0.47 to -0.03)**	**-0.23 (-0.43 to -0.03)**	-0.07 (-0.46 to 0.32)	**Haloperidol**	NA	NA	NA	0.09 (-0.34 to 0.52)
-0.69 (-1.45 to 0.07)	-0.67 (-1.59 to 0.24)	**-0.47 (-0.94 to -0.01)**	-0.37 (-0.80 to 0.06)	-0.35 (-0.83 to 0.13)	-0.19 (-0.77 to 0.39)	-0.12 (-0.61 to 0.36)	**Sertindole**	NA	NA	NA
**-0.70 (-1.35 to -0.05)**	-0.68 (-1.43 to 0.06)	**-0.49 (-0.74 to -0.23)**	**-0.38 (-0.68 to -0.08)**	**-0.36 (-0.64 to -0.09)**	-0.20 (-0.54 to 0.14)	-0.13 (-0.43 to 0.16)	-0.01 (-0.54 to 0.51)	**Chlorpromazine**	NA	-0.31 (-0.75 to 0.13)
-0.84 (-1.81 to 0.13)	-0.82 (-1.91 to 0.26)	-0.63 (-1.39 to 0.14)	-0.53 (-1.28 to 0.23)	-0.51 (-1.27 to 0.26)	-0.34 (-1.17 to 0.49)	-0.28 (-1.05 to 0.49)	-0.15 (-1.03 to 0.72)	-0.14 (-0.93 to 0.65)	**Fluphenazine**	-0.23 (-1.09 to 0.64)
**-0.82 (-1.48 to -0.16)**	-0.81 (-1.62 to 0.00)	**-0.61 (-0.90 to -0.32)**	**-0.51 (-0.82 to -0.20)**	**-0.49 (-0.78 to -0.19)**	-0.33 (-0.75 to 0.10)	-0.26 (-0.55 to 0.03)	-0.13 (-0.67 to 0.40)	-0.12 (-0.43 to 0.19)	0.02 (-0.74 to 0.78)	**Quetiapine**

Treatments are presented in order of efficacy ranking. Results of the network meta-analysis are reported in the left lower half and results of pairwise meta-analyses in the right upper half. Each cell provides the effect estimate and the corresponding 95% credible interval (95% CI) of a comparison (left lower half: treatment in column versus treatment in row; right upper half: treatment in row versus treatment in column). The type of effect size measure is standardized mean difference (SMD). Bold results indicate 95% CI excluding no effect. NA=not available.

Forest plot of results of pairwise meta-analyses

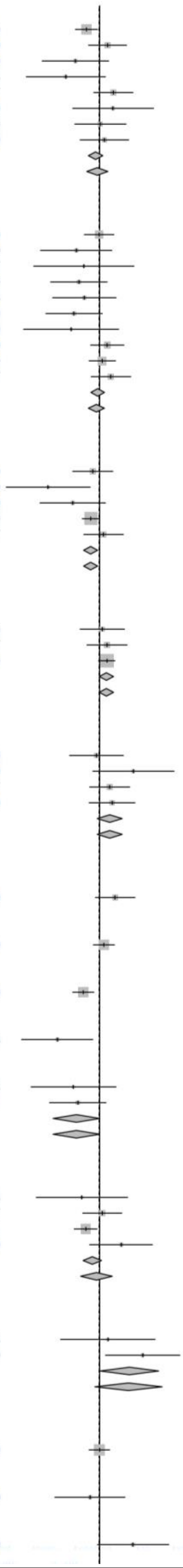

Study	Experimental Total	Experimental Mean	Experimental SD	Control Total	Control Mean	Control SD	SMD	95%-CI	Weight (common)	Weight (random)
comparison = Clozapine_Risperidone										
Azorin 2001 Clozapine Risperidone	126	−10.40	6.6100	130	−8.30	7.3900	−0.30	[−0.54; −0.05]	4.8%	3.2%
Bondolfi 1998 Clozapine Risperidone	43	−6.70	7.1000	43	−8.30	10.7000	0.17	[−0.25; 0.60]	1.6%	2.3%
Breier 1999 Clozapine Risperidone	14	7.43	2.2100	15	9.53	4.7300	−0.55	[−1.29; 0.20]	0.5%	1.2%
McEvoy 2006 Clozapine Risperidone	33	−4.90	5.6498	6	−0.50	5.6498	−0.76	[−1.65; 0.13]	0.4%	0.9%
Volavka 2002 Clozapine Risperidone	40	23.40	7.1257	41	21.20	7.1257	0.31	[−0.13; 0.74]	1.5%	2.2%
Wahlbeck 2000 Clozapine Risperidone	10	17.00	6.0000	9	15.00	7.0000	0.29	[−0.61; 1.20]	0.4%	0.9%
Chowdhury 1999 Clozapine Risperidone	24	10.08	3.0600	22	10.04	3.2600	0.01	[−0.57; 0.59]	0.9%	1.6%
Schooler 2016 Clozapine Risperidone	30	2.50	0.9000	22	2.40	1.0000	0.10	[−0.45; 0.65]	1.0%	1.7%
Common effect model	320			288			−0.09	[−0.26; 0.07]	11.1%	--
Random effects model							−0.05	[−0.29; 0.18]	--	14.0%
Heterogeneity: $I^2 = 43\%$, $\tau^2 = 0.0431$, $p = 0.09$										
comparison = Clozapine_Olanzapine										
Bitter 2004 Clozapine Olanzapine	70	−11.80	7.9000	70	−11.70	7.3000	−0.01	[−0.34; 0.32]	2.7%	2.7%
Shaw 2006 Clozapine Olanzapine	12	−22.00	16.5739	13	−13.00	16.5739	−0.53	[−1.33; 0.28]	0.5%	1.1%
Conley 2003 Clozapine Olanzapine	5	−0.80	2.8707	8	0.31	2.8707	−0.36	[−1.49; 0.77]	0.2%	0.6%
Kumra 2007 Clozapine Olanzapine	18	6.30	2.6000	21	8.10	4.5000	−0.47	[−1.11; 0.17]	0.7%	1.5%
McEvoy 2006 Clozapine Olanzapine	33	−4.90	5.6498	10	−2.90	5.6498	−0.35	[−1.06; 0.36]	0.6%	1.3%
Meltzer 2008 Clozapine Olanzapine	21	15.10	5.0400	19	17.80	3.9200	−0.58	[−1.22; 0.05]	0.7%	1.5%
Moresco 2004 Clozapine Olanzapine	6	9.50	3.3000	9	11.20	1.8000	−0.64	[−1.71; 0.42]	0.3%	0.7%
Naber 2005 Clozapine Olanzapine	56	−7.60	8.0000	52	−9.00	8.5000	0.17	[−0.21; 0.55]	2.0%	2.5%
Tollefson 2001 Clozapine Olanzapine	87	−6.40	7.2000	89	−6.80	7.6000	0.05	[−0.24; 0.35]	3.4%	2.9%
Volavka 2002 Clozapine Olanzapine	40	23.40	7.1257	39	21.60	7.1257	0.25	[−0.19; 0.69]	1.5%	2.2%
Common effect model	348			330			−0.05	[−0.20; 0.11]	12.5%	--
Random effects model							−0.08	[−0.26; 0.10]	--	16.8%
Heterogeneity: $I^2 = 21\%$, $\tau^2 = 0.0158$, $p = 0.25$										
comparison = Clozapine_Haloperidol										
Buchanan 1998 Clozapine Haloperidol	38	11.00	5.2000	37	11.80	4.8000	−0.16	[−0.61; 0.30]	1.4%	2.1%
Kumra 1996 Clozapine Haloperidol	10	19.10	11.7000	11	35.90	15.6000	−1.16	[−2.10; −0.22]	0.3%	0.8%
Kane 2001 Clozapine Haloperidol	23	12.10	3.5000	11	14.80	5.7000	−0.61	[−1.35; 0.12]	0.5%	1.2%
Rosenheck 1997 Clozapine Haloperidol	205	19.50	8.4800	218	21.20	8.4800	−0.20	[−0.39; −0.01]	8.0%	3.4%
Volavka 2002 Clozapine Haloperidol	40	23.40	7.1257	37	22.80	7.1257	0.08	[−0.36; 0.53]	1.5%	2.1%
Common effect model	316			314			−0.21	[−0.36; −0.05]	11.8%	--
Random effects model							−0.21	[−0.36; −0.05]	--	9.7%
Heterogeneity: $I^2 = 41\%$, $\tau^2 = < 0.0001$, $p = 0.15$										
comparison = Haloperidol_Olanzapine										
Buchanan 2005 Haloperidol Olanzapine	34	10.10	5.3000	29	9.80	5.3000	0.06	[−0.44; 0.55]	1.2%	1.9%
Volavka 2002 Haloperidol Olanzapine	37	22.80	7.1257	39	21.60	7.1257	0.17	[−0.28; 0.62]	1.4%	2.1%
Breier 1999a Haloperidol Olanzapine	172	−4.00	6.2000	344	−5.10	7.3000	0.16	[−0.03; 0.34]	8.7%	3.5%
Common effect model	243			412			0.15	[−0.01; 0.31]	11.3%	--
Random effects model							0.15	[−0.01; 0.31]	--	7.5%
Heterogeneity: $I^2 = 0\%$, $\tau^2 = 0$, $p = 0.93$										
comparison = Haloperidol_Risperidone										
Claus 1992 Haloperidol Risperidone	21	15.40	5.9600	21	15.90	6.4200	−0.08	[−0.68; 0.53]	0.8%	1.6%
See 1999 Haloperidol Risperidone	10	13.05	2.9400	10	10.90	2.5000	0.75	[−0.16; 1.67]	0.4%	0.9%
Volavka 2002 Haloperidol Risperidone	37	22.80	7.1257	41	21.20	7.1257	0.22	[−0.22; 0.67]	1.5%	2.2%
Wirshing 1999 Haloperidol Risperidone	29	14.40	4.6300	29	13.10	4.6300	0.28	[−0.24; 0.79]	1.1%	1.9%
Common effect model	97			101			0.22	[−0.06; 0.50]	3.7%	--
Random effects model							0.22	[−0.06; 0.50]	--	6.4%
Heterogeneity: $I^2 = 0\%$, $\tau^2 = < 0.0001$, $p = 0.51$										
comparison = Chlorpromazine_Olanzapine										
Conley 1998 Chlorpromazine Olanzapine	39	19.40	3.1000	42	18.10	4.2000	0.35	[−0.09; 0.79]	1.5%	2.2%
comparison = Haloperidol_Quetiapine										
Emsley 2000 Haloperidol Quetiapine	140	−2.85	6.1800	140	−3.43	6.1800	0.09	[−0.14; 0.33]	5.3%	3.2%
comparison = Risperidone_Sertindole										
Kane 2010b Risperidone Sertindole	102	−5.80	6.3000	213	−3.30	6.9000	−0.37	[−0.61; −0.13]	5.2%	3.2%
comparison = Clozapine_Quetiapine										
McEvoy 2006 Clozapine Quetiapine	33	−4.90	5.6498	8	0.60	5.6498	−0.95	[−1.76; −0.15]	0.5%	1.1%
comparison = Olanzapine_Quetiapine										
McEvoy 2006 Olanzapine Quetiapine	10	−2.90	5.6498	8	0.60	5.6498	−0.59	[−1.54; 0.36]	0.3%	0.8%
Sirota 2006 Olanzapine Quetiapine	21	−4.00	5.9421	19	−1.00	5.9421	−0.49	[−1.13; 0.14]	0.7%	1.5%
Common effect model	31			27			−0.52	[−1.05; 0.00]	1.1%	--
Random effects model							−0.52	[−1.05; 0.00]	--	2.3%
Heterogeneity: $I^2 = 0\%$, $\tau^2 = 0$, $p = 0.87$										
comparison = Olanzapine_Risperidone										
McEvoy 2006 Olanzapine Risperidone	10	−2.90	5.6498	6	−0.50	5.6498	−0.40	[−1.43; 0.62]	0.3%	0.7%
Volavka 2002 Olanzapine Risperidone	39	21.60	7.1257	41	21.20	7.1257	0.06	[−0.38; 0.49]	1.5%	2.2%
Alvarez 2006 Olanzapine Risperidone	120	−18.98	16.4317	115	−13.64	17.1581	−0.32	[−0.57; −0.06]	4.4%	3.1%
Chen 2012 Olanzapine Risperidone	16	−3.50	3.3000	16	−5.80	5.7000	0.48	[−0.22; 1.19]	0.6%	1.3%
Common effect model	185			178			−0.17	[−0.38; 0.04]	6.8%	--
Random effects model							−0.07	[−0.43; 0.28]	--	7.3%
Heterogeneity: $I^2 = 48\%$, $\tau^2 = 0.0600$, $p = 0.12$										
comparison = Quetiapine_Risperidone										
McEvoy 2006 Quetiapine Risperidone	8	0.60	5.6498	6	−0.50	5.6498	0.18	[−0.88; 1.24]	0.3%	0.7%
Conley 2005 Quetiapine Risperidone	12	−0.67	1.1009	13	−1.77	1.1009	0.97	[0.13; 1.80]	0.4%	1.0%
Common effect model	20			19			0.67	[0.01; 1.32]	0.7%	--
Random effects model							0.65	[−0.11; 1.40]	--	1.7%
Heterogeneity: $I^2 = 23\%$, $\tau^2 = 0.0696$, $p = 0.26$										
comparison = Aripiprazole_Perphenazine										
Kane 2007a Aripiprazole Perphenazine	150	−2.70	6.4000	144	−2.65	6.4900	−0.01	[−0.24; 0.22]	5.6%	3.2%
comparison = Fluphenazine_Quetiapine										
Conley 2005 Fluphenazine Quetiapine	13	−0.92	1.1009	12	−0.67	1.1009	−0.22	[−1.01; 0.57]	0.5%	1.1%
comparison = Fluphenazine_Risperidone										
Conley 2005 Fluphenazine Risperidone	13	−0.92	1.1009	13	−1.77	1.1009	0.75	[−0.05; 1.55]	0.5%	1.1%

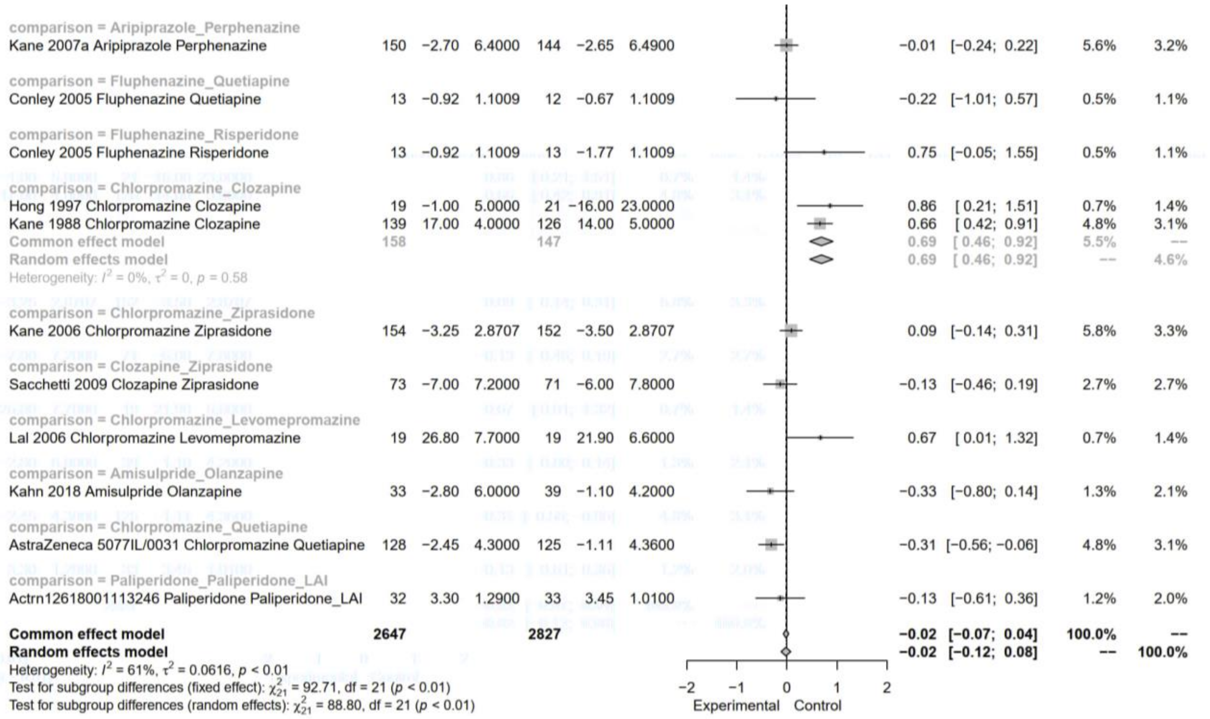

A summary effect size is calculated by pairwise meta-analyses of all studies of a specific comparison. The type of effect size measure is standardized mean difference (SMD).

8.2 Negative symptoms

Network plot

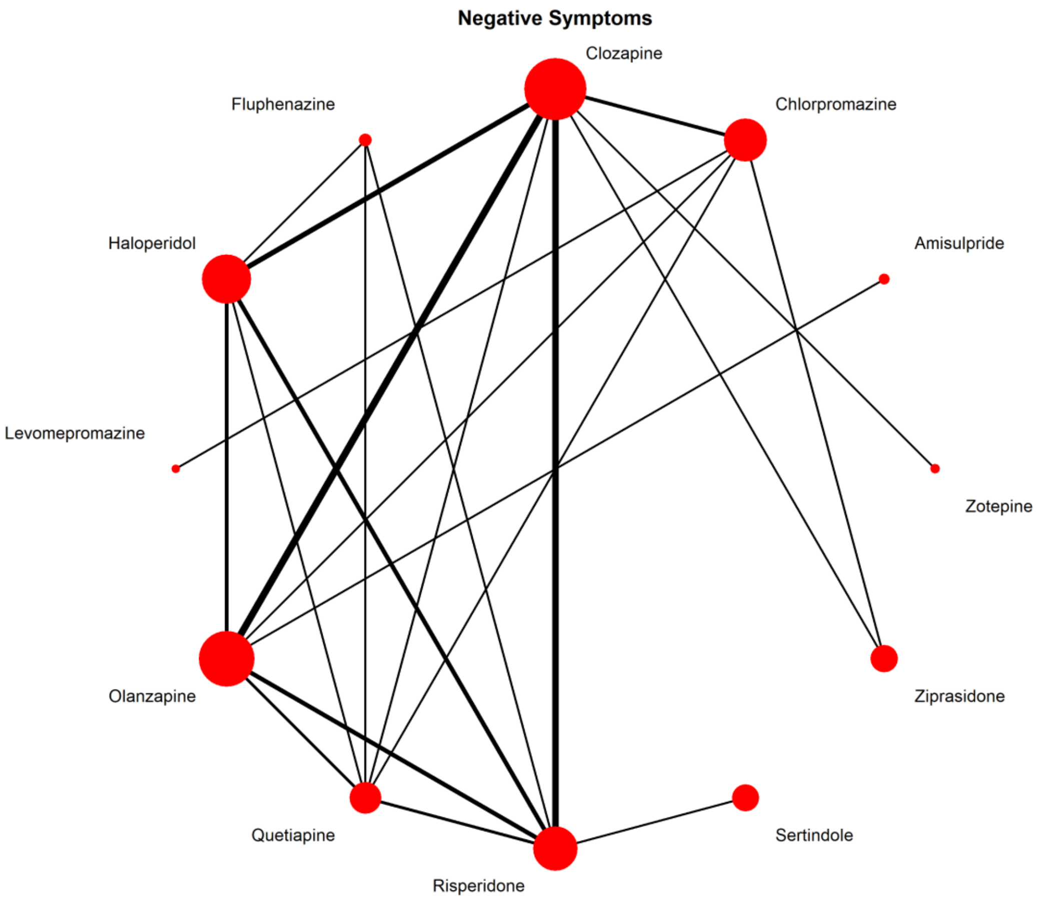

Lines link treatments with direct comparisons in trials; thickness of lines corresponds to the number of trials evaluating the comparison; size of the nodes corresponds to the number of participants assigned to the treatment.

Forest-plot of results of network meta-analysis for antipsychotic drugs versus clozapine

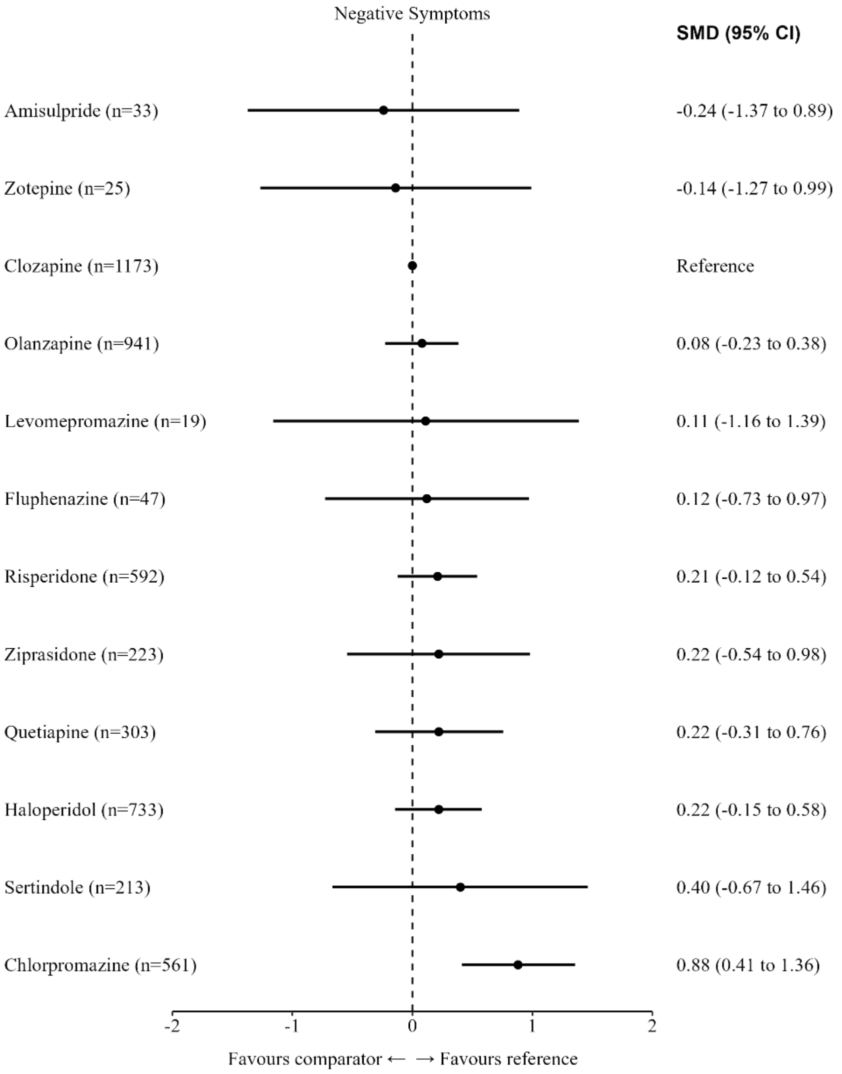

Effect sizes, measured as standardized mean difference (SMD), are from the network meta-analysis. Order of treatments is according to the mean effect size. Reference is clozapine. The direction of the effect is indicated below the x-axis.

League table for the outcome: Negative symptoms

**Amisulpride**	NA	NA	-0.32 (-1.41 to 0.77)	NA	NA	NA	NA	NA	NA	NA	NA
-0.24 (-1.37 to 0.89)	**Clozapine**	0.14 (-0.99 to 1.27)	-0.06 (-0.43 to 0.31)	NA	NA	0.23 (-0.81 to 1.26)	-0.75 (-2.00 to 0.51)	-0.02 (-0.43 to 0.39)	-0.19 (-0.70 to 0.31)	NA	**-1.43 (-2.05 to -0.80)**
-0.10 (-1.70 to 1.49)	0.14 (-0.99 to 1.27)	**Zotepine**	NA	NA	NA	NA	NA	NA	NA	NA	NA
-0.32 (-1.41 to 0.77)	-0.08 (-0.38 to 0.23)	-0.22 (-1.39 to 0.95)	**Olanzapine**	NA	NA	NA	0.03 (-0.85 to 0.91)	-0.36 (-0.93 to 0.22)	-0.25 (-0.86 to 0.36)	NA	-0.10 (-1.18 to 0.97)
-0.36 (-1.75 to 1.02)	-0.12 (-0.97 to 0.73)	-0.26 (-1.67 to 1.15)	-0.04 (-0.90 to 0.82)	**Fluphenazine**	NA	NA	-0.04 (-1.29 to 1.22)	-0.51 (-1.76 to 0.75)	0.12 (-1.06 to 1.29)	NA	NA
-0.36 (-2.04 to 1.33)	-0.11 (-1.39 to 1.16)	-0.25 (-1.95 to 1.45)	-0.04 (-1.33 to 1.25)	0.01 (-1.50 to 1.52)	**Levomepromazine**	NA	NA	NA	NA	NA	-0.77 (-1.95 to 0.41)
-0.46 (-1.81 to 0.89)	-0.22 (-0.98 to 0.54)	-0.36 (-1.72 to 1.01)	-0.14 (-0.94 to 0.67)	-0.10 (-1.22 to 1.03)	-0.10 (-1.51 to 1.30)	**Ziprasidone**	NA	NA	NA	NA	-0.24 (-1.25 to 0.76)
-0.46 (-1.68 to 0.75)	-0.22 (-0.76 to 0.31)	-0.36 (-1.61 to 0.89)	-0.14 (-0.68 to 0.40)	-0.10 (-0.98 to 0.78)	-0.11 (-1.44 to 1.22)	-0.01 (-0.90 to 0.89)	**Quetiapine**	-0.35 (-1.30 to 0.60)	-0.12 (-1.13 to 0.89)	NA	-0.28 (-1.29 to 0.74)
-0.45 (-1.60 to 0.70)	-0.21 (-0.54 to 0.12)	-0.35 (-1.52 to 0.83)	-0.13 (-0.50 to 0.25)	-0.09 (-0.93 to 0.76)	-0.09 (-1.40 to 1.21)	0.01 (-0.81 to 0.83)	0.02 (-0.54 to 0.57)	**Risperidone**	0.08 (-0.50 to 0.66)	-0.19 (-1.20 to 0.82)	NA
-0.46 (-1.61 to 0.70)	-0.22 (-0.58 to 0.15)	-0.35 (-1.54 to 0.83)	-0.14 (-0.53 to 0.25)	-0.09 (-0.92 to 0.73)	-0.10 (-1.41 to 1.21)	0.00 (-0.83 to 0.83)	0.01 (-0.54 to 0.56)	-0.01 (-0.40 to 0.38)	**Haloperidol**	NA	NA
-0.64 (-2.17 to 0.89)	-0.40 (-1.46 to 0.67)	-0.54 (-2.09 to 1.02)	-0.32 (-1.40 to 0.76)	-0.28 (-1.59 to 1.04)	-0.28 (-1.93 to 1.37)	-0.18 (-1.48 to 1.12)	-0.17 (-1.33 to 0.98)	-0.19 (-1.20 to 0.82)	-0.18 (-1.27 to 0.90)	**Sertindole**	NA
-1.13 (-2.33 to 0.08)	**-0.88 (-1.36 to -0.41)**	-1.02 (-2.25 to 0.20)	**-0.81 (-1.32 to -0.29)**	-0.76 (-1.70 to 0.18)	-0.77 (-1.95 to 0.41)	-0.67 (-1.42 to 0.09)	**-0.66 (-1.28 to -0.05)**	**-0.68 (-1.23 to -0.12)**	**-0.67 (-1.23 to -0.10)**	-0.49 (-1.64 to 0.67)	**Chlorpromazine**

Treatments are presented in order of efficacy ranking. Results of the network meta-analysis are reported in the left lower half and results of pairwise meta-analyses in the right upper half. Each cell provides the effect estimate and the corresponding 95% credible interval (95% CI) of a comparison (left lower half: treatment in column versus treatment in row; right upper half: treatment in row versus treatment in column). The type of effect size measure is standardized mean difference (SMD). Bold results indicate 95% CI excluding no effect. NA=not available.

Forest plot of results of pairwise meta-analyses

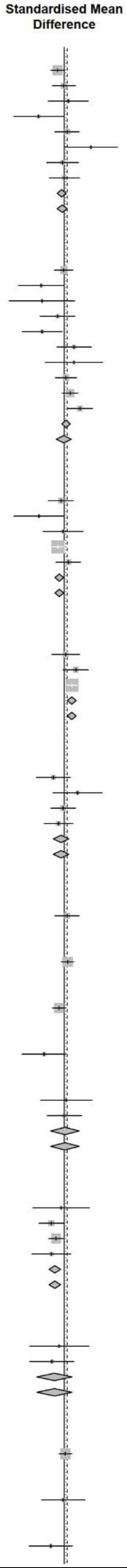

Study	Experimental Total	Experimental Mean	Experimental SD	Control Total	Control Mean	Control SD	SMD	95%-CI	Weight (common)	Weight (random)
comparison = Clozapine_Risperidone										
Azorin 2001 Clozapine Risperidone	124	−8.80	6.7500	128	−7.10	7.2400	−0.24	[−0.49; 0.01]	4.7%	2.2%
Bondolfi 1998 Clozapine Risperidone	43	−6.10	6.1000	43	−6.00	6.5000	−0.02	[−0.44; 0.41]	1.6%	2.0%
Breier 1999 Clozapine Risperidone	14	48.93	8.4300	15	47.60	9.4200	0.14	[−0.59; 0.87]	0.5%	1.6%
McEvoy 2006 Clozapine Risperidone	33	−5.30	5.6292	6	0.00	5.6292	−0.92	[−1.82; −0.03]	0.4%	1.3%
Volavka 2002 Clozapine Risperidone	40	23.50	5.9836	41	22.90	5.9836	0.10	[−0.34; 0.54]	1.5%	2.0%
Wahlbeck 2000 Clozapine Risperidone	10	21.00	4.0000	9	17.00	4.0000	0.96	[−0.01; 1.92]	0.3%	1.2%
Chowdhury 1999 Clozapine Risperidone	24	14.08	6.6600	22	14.55	8.3300	−0.06	[−0.64; 0.52]	0.9%	1.8%
Schooler 2016 Clozapine Risperidone	30	1.80	0.7000	22	1.80	0.6000	0.00	[−0.55; 0.55]	1.0%	1.8%
Common effect model	318			286			−0.09	[−0.26; 0.07]	10.9%	--
Random effects model							−0.07	[−0.26; 0.11]	--	13.9%
Heterogeneity: $I^2 = 34\%$, $\tau^2 = 0.0092$, $p = 0.16$										
comparison = Clozapine_Olanzapine										
Bitter 2004 Clozapine Olanzapine	70	−7.70	6.1000	70	−7.60	6.0000	−0.02	[−0.35; 0.31]	2.6%	2.1%
Shaw 2006 Clozapine Olanzapine	12	−25.00	12.7426	13	−14.00	12.7426	−0.83	[−1.66; −0.01]	0.4%	1.4%
Conley 2003 Clozapine Olanzapine	5	0.30	0.9008	8	1.08	0.9008	−0.81	[−1.98; 0.37]	0.2%	1.0%
Kumra 2007 Clozapine Olanzapine	18	6.60	4.4000	21	7.60	3.8000	−0.24	[−0.87; 0.39]	0.7%	1.7%
McEvoy 2006 Clozapine Olanzapine	33	−5.30	5.6292	10	−0.70	5.6292	−0.80	[−1.53; −0.07]	0.5%	1.6%
Meltzer 2008 Clozapine Olanzapine	21	20.90	5.5000	19	19.10	4.3600	0.35	[−0.27; 0.98]	0.7%	1.7%
Moresco 2004 Clozapine Olanzapine	6	18.90	5.6000	9	17.30	3.4000	0.34	[−0.70; 1.39]	0.3%	1.1%
Naber 2005 Clozapine Olanzapine	56	−8.00	8.5000	52	−8.50	8.6000	0.06	[−0.32; 0.44]	2.0%	2.1%
Tollefson 2001 Clozapine Olanzapine	87	−5.60	6.9000	89	−7.10	7.4000	0.21	[−0.09; 0.50]	3.3%	2.2%
Volavka 2002 Clozapine Olanzapine	40	23.50	5.9836	39	20.10	5.9836	0.56	[0.11; 1.01]	1.4%	2.0%
Common effect model	348			330			0.06	[−0.09; 0.22]	12.3%	--
Random effects model							−0.02	[−0.29; 0.25]	--	16.8%
Heterogeneity: $I^2 = 55\%$, $\tau^2 = 0.0995$, $p = 0.02$										
comparison = Clozapine_Haloperidol										
Buchanan 1998 Clozapine Haloperidol	38	27.00	11.5000	37	28.40	11.1000	−0.12	[−0.58; 0.33]	1.4%	2.0%
Kumra 1996 Clozapine Haloperidol	10	46.00	30.3000	11	72.20	24.7000	−0.91	[−1.82; −0.01]	0.3%	1.3%
Kane 2001 Clozapine Haloperidol	23	9.70	15.5955	11	10.40	15.5955	−0.04	[−0.76; 0.67]	0.6%	1.6%
Rosenheck 1997 Clozapine Haloperidol	205	20.90	1.3200	218	21.20	1.3200	−0.23	[−0.42; −0.04]	7.9%	2.3%
Volavka 2002 Clozapine Haloperidol	40	23.50	5.9836	37	22.60	5.9836	0.15	[−0.30; 0.60]	1.4%	2.0%
Common effect model	316			314			−0.18	[−0.34; −0.02]	11.6%	--
Random effects model							−0.18	[−0.34; −0.02]	--	9.1%
Heterogeneity: $I^2 = 20\%$, $\tau^2 = < 0.0001$, $p = 0.29$										
comparison = Haloperidol_Olanzapine										
Buchanan 2005 Haloperidol Olanzapine	34	30.20	11.6000	29	29.60	12.4000	0.05	[−0.45; 0.54]	1.2%	1.9%
Volavka 2002 Haloperidol Olanzapine	37	22.60	5.9836	39	20.10	5.9836	0.41	[−0.04; 0.87]	1.4%	2.0%
Breier 1999a Haloperidol Olanzapine	172	−2.90	6.4000	344	−4.70	6.6000	0.28	[0.09; 0.46]	8.5%	2.3%
Common effect model	243			412			0.27	[0.11; 0.43]	11.1%	--
Random effects model							0.27	[0.11; 0.43]	--	6.1%
Heterogeneity: $I^2 = 0\%$, $\tau^2 = 0$, $p = 0.56$										
comparison = Haloperidol_Risperidone										
Claus 1992 Haloperidol Risperidone	21	22.00	7.7900	21	25.20	8.2500	−0.39	[−1.00; 0.22]	0.8%	1.7%
See 1999 Haloperidol Risperidone	10	21.90	5.8500	10	19.20	4.9600	0.48	[−0.41; 1.37]	0.4%	1.3%
Volavka 2002 Haloperidol Risperidone	37	22.60	5.9836	41	22.90	5.9836	−0.05	[−0.49; 0.39]	1.5%	2.0%
Wirshing 1999 Haloperidol Risperidone	29	8.70	3.2800	29	9.40	3.2800	−0.21	[−0.73; 0.31]	1.1%	1.9%
Common effect model	97			101			−0.12	[−0.40; 0.16]	3.7%	--
Random effects model							−0.12	[−0.40; 0.16]	--	6.9%
Heterogeneity: $I^2 = 0\%$, $\tau^2 = < 0.0001$, $p = 0.44$										
comparison = Chlorpromazine_Olanzapine										
Conley 1998 Chlorpromazine Olanzapine	39	14.00	9.5000	42	13.10	8.2000	0.10	[−0.34; 0.54]	1.5%	2.0%
comparison = Haloperidol_Quetiapine										
Emsley 2000 Haloperidol Quetiapine	140	−2.39	4.9900	139	−3.00	4.9900	0.12	[−0.11; 0.36]	5.2%	2.2%
comparison = Risperidone_Sertindole										
Kane 2010b Risperidone Sertindole	102	−3.70	5.9000	213	−2.50	6.5000	−0.19	[−0.43; 0.05]	5.2%	2.2%
comparison = Clozapine_Quetiapine										
McEvoy 2006 Clozapine Quetiapine	33	−5.30	5.6292	8	−1.10	5.6292	−0.73	[−1.52; 0.06]	0.5%	1.5%
comparison = Olanzapine_Quetiapine										
McEvoy 2006 Olanzapine Quetiapine	10	−0.70	5.6292	8	−1.10	5.6292	0.07	[−0.86; 1.00]	0.3%	1.3%
Sirota 2006 Olanzapine Quetiapine	21	−5.00	9.6957	19	−5.00	9.6957	0.00	[−0.62; 0.62]	0.7%	1.7%
Common effect model	31			27			0.02	[−0.50; 0.54]	1.1%	--
Random effects model							0.02	[−0.50; 0.54]	--	3.0%
Heterogeneity: $I^2 = 0\%$, $\tau^2 = 0$, $p = 0.91$										
comparison = Olanzapine_Risperidone										
McEvoy 2006 Olanzapine Risperidone	10	−0.70	5.6292	6	0.00	5.6292	−0.12	[−1.13; 0.90]	0.3%	1.2%
Volavka 2002 Olanzapine Risperidone	39	20.10	5.9836	41	22.90	5.9836	−0.46	[−0.91; −0.02]	1.5%	2.0%
Alvarez 2006 Olanzapine Risperidone	120	−32.59	25.1952	115	−24.97	25.7371	−0.30	[−0.56; −0.04]	4.4%	2.2%
Chen 2012 Olanzapine Risperidone	16	−4.10	6.3000	16	−1.20	6.0000	−0.46	[−1.16; 0.24]	0.6%	1.6%
Common effect model	185			178			−0.34	[−0.55; −0.13]	6.7%	--
Random effects model							−0.34	[−0.55; −0.13]	--	7.0%
Heterogeneity: $I^2 = 0\%$, $\tau^2 = 0$, $p = 0.87$										
comparison = Quetiapine_Risperidone										
McEvoy 2006 Quetiapine Risperidone	8	−1.10	5.6292	6	0.00	5.6292	−0.18	[−1.24; 0.88]	0.3%	1.1%
Conley 2005 Quetiapine Risperidone	12	6.42	3.0428	13	7.85	3.0428	−0.45	[−1.25; 0.34]	0.5%	1.5%
Common effect model	20			19			−0.36	[−0.99; 0.28]	0.7%	--
Random effects model							−0.36	[−0.99; 0.28]	--	2.6%
Heterogeneity: $I^2 = 0\%$, $\tau^2 = 0$, $p = 0.69$										
comparison = Aripiprazole_Perphenazine										
Kane 2007a Aripiprazole Perphenazine	150	−2.35	5.9400	144	−2.59	6.0000	0.04	[−0.19; 0.27]	5.5%	2.2%
comparison = Fluphenazine_Quetiapine										
Conley 2005 Fluphenazine Quetiapine	13	6.31	3.0428	12	6.42	3.0428	−0.03	[−0.82; 0.75]	0.5%	1.5%
comparison = Fluphenazine_Risperidone										
Conley 2005 Fluphenazine Risperidone	13	6.31	3.0428	13	7.85	3.0428	−0.49	[−1.27; 0.29]	0.5%	1.5%

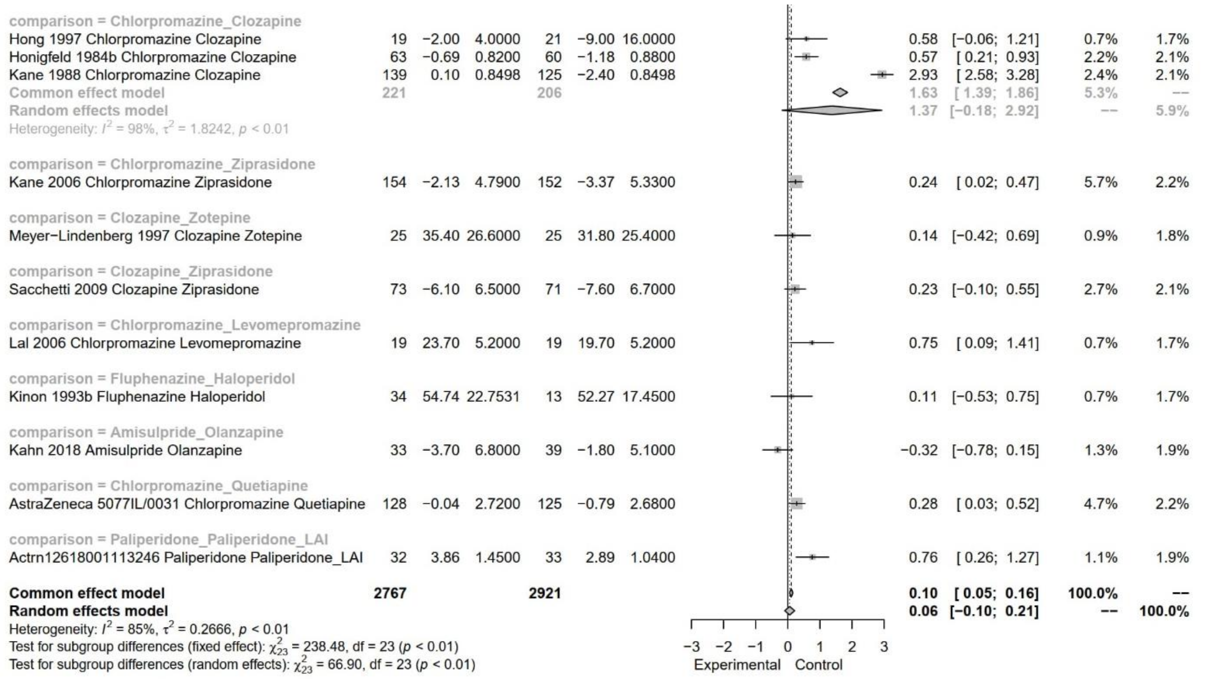

A summary effect size is calculated by pairwise meta-analyses of all studies of a specific comparison. The type of effect size measure is standardized mean difference (SMD).

8.3 Response

The following hierarchy of the response definitions were applied: at least 20% reduction of the baseline score of the PANSS, 20% reduction of the BPRS or 20% reduction of any other global schizophrenia rating scale, at least “minimally improved” (score of 3) on the Clinical Global-Impressions-Improvement Scale (CGI). We chose this cutoff because even minimal improvement can be clinically important for patients with treatment-resistant schizophrenia. If none of these definitions was available, we used the original authors’ primary definition.

We transformed the original ORs to RRs and EERs vs. CER assuming the baseline response rate with clozapine of 49% (namely CER). 49% was the average response rate with clozapine across all clozapine-arms in the network meta-analysis, as estimated by a single-arm meta-analysis of proportions (see below).

<u>Forest plot of the single arm meta-analysis of response in the clozapine-arm</u>

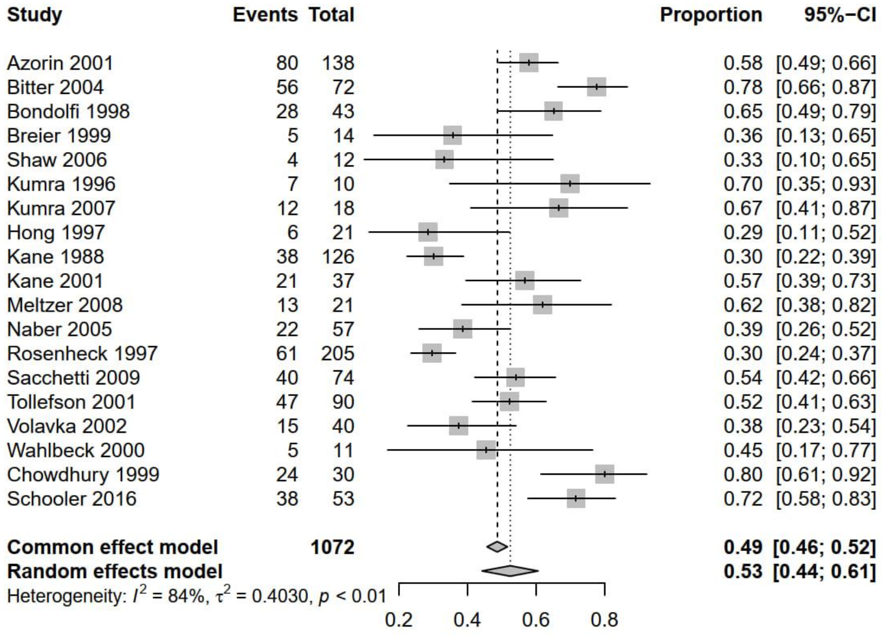

Forest plot of response in all clozapine-arms included in the network meta-analysis. The summary effect size is from a single-arm meta-analysis of proportions.

Because inconsistency of the network was high for response to treatment (12.9% inconsistent comparisons, design-by-treatment interaction test p=0.0194), we conducted a pairwise meta-analysis of active antipsychotics versus clozapine and present the results below.

Forest-plot of results of pairwise meta-analysis for antipsychotic drugs versus clozapine with OR

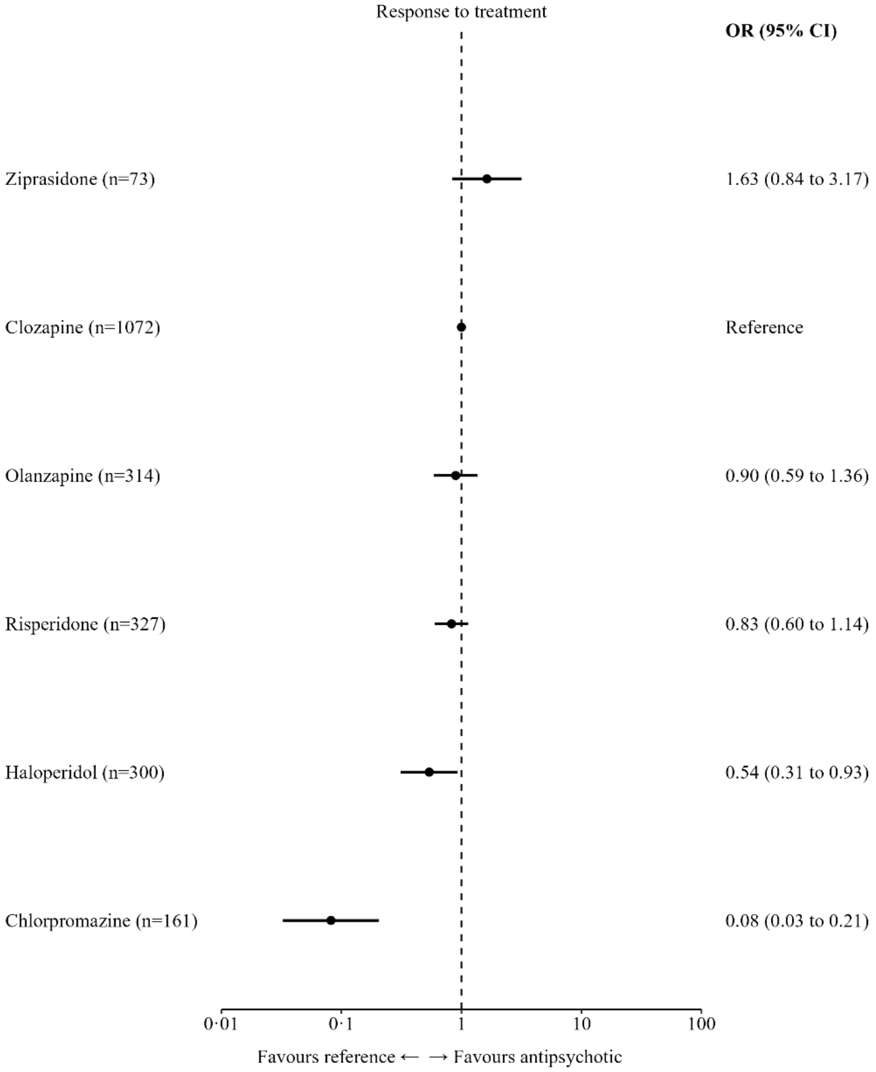

Effect sizes, measured as odds ratio (OR), are from the network meta-analysis. Order of treatments is according to the mean effect size. Reference is clozapine. The direction of the effect is indicated below the x-axis.

Forest-plot of results of pairwise meta-analysis for antipsychotic drugs versus clozapine with RR

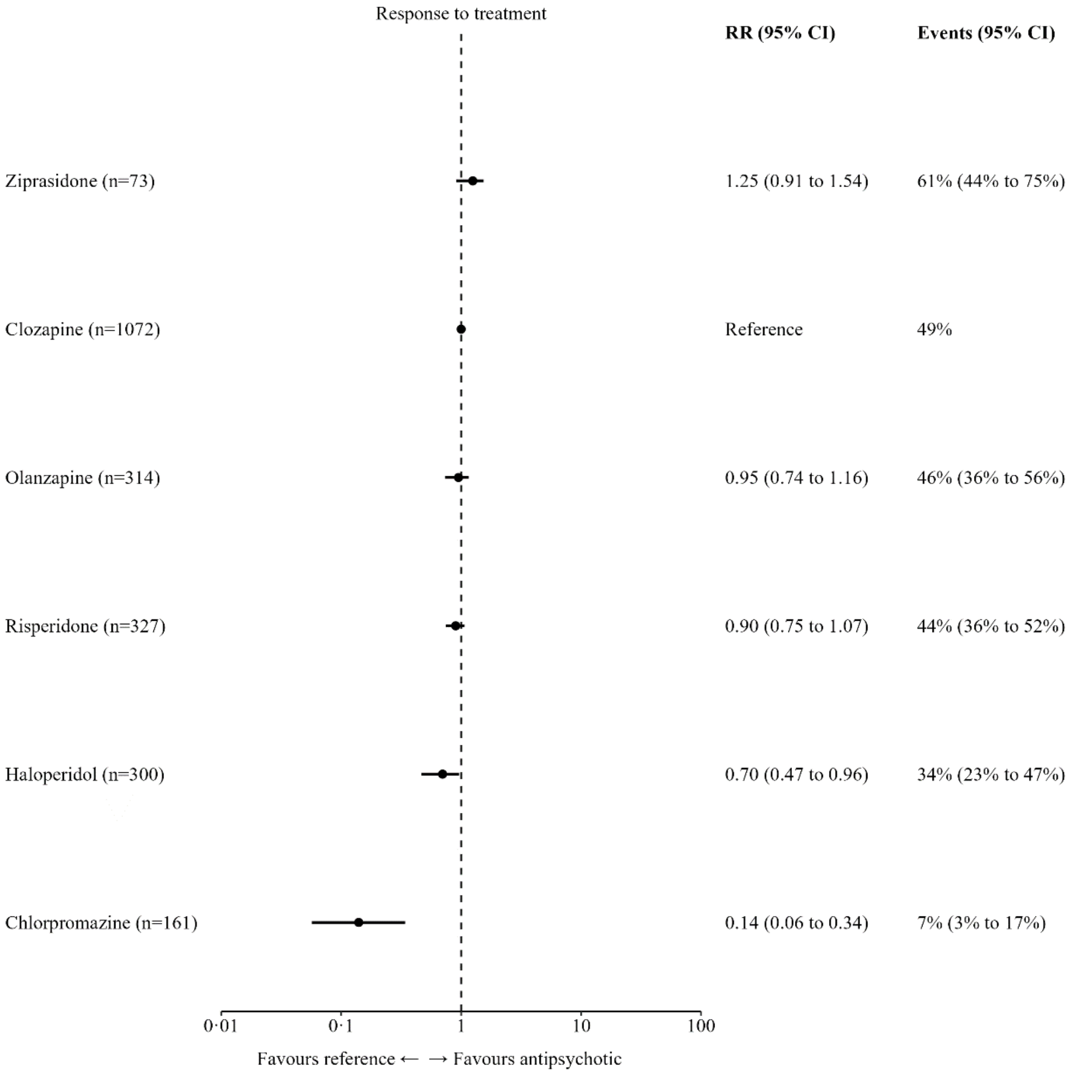

For this forest plot, we transformed the original OR to RR and exposure events rates (EER, called "events" in the forest plot) using the formula indicated above. Therefore, we used an average response rate with clozapine of 49% as the control event rate (CER).
Effect sizes are from the pairwise meta-analysis. Order of treatments is according to the mean effect size. Reference is clozapine. The direction of the effect is indicated below the x-axis.

We additionally present the results of the network meta-analysis below, which agree with the results of the pairwise meta-analysis, but it should be emphasized that differences between antipsychotics need to be interpreted with caution due to the observed inconsistency.

Network plot

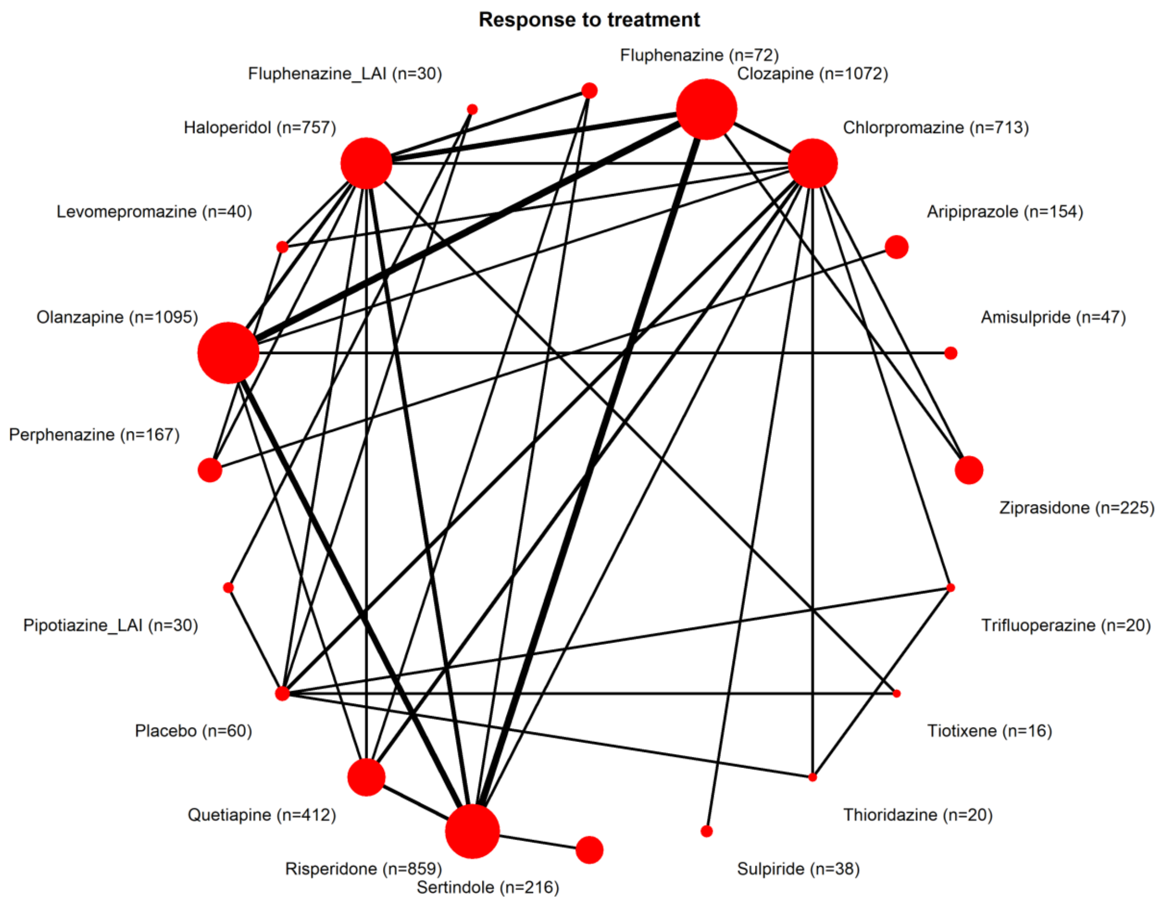

Lines link treatments with direct comparisons in trials; thickness of lines corresponds to the number of trials evaluating the comparison; size of the nodes corresponds to the number of participants assigned to the treatment.

Forest-plot of results of network meta-analysis for antipsychotic drugs versus clozapine

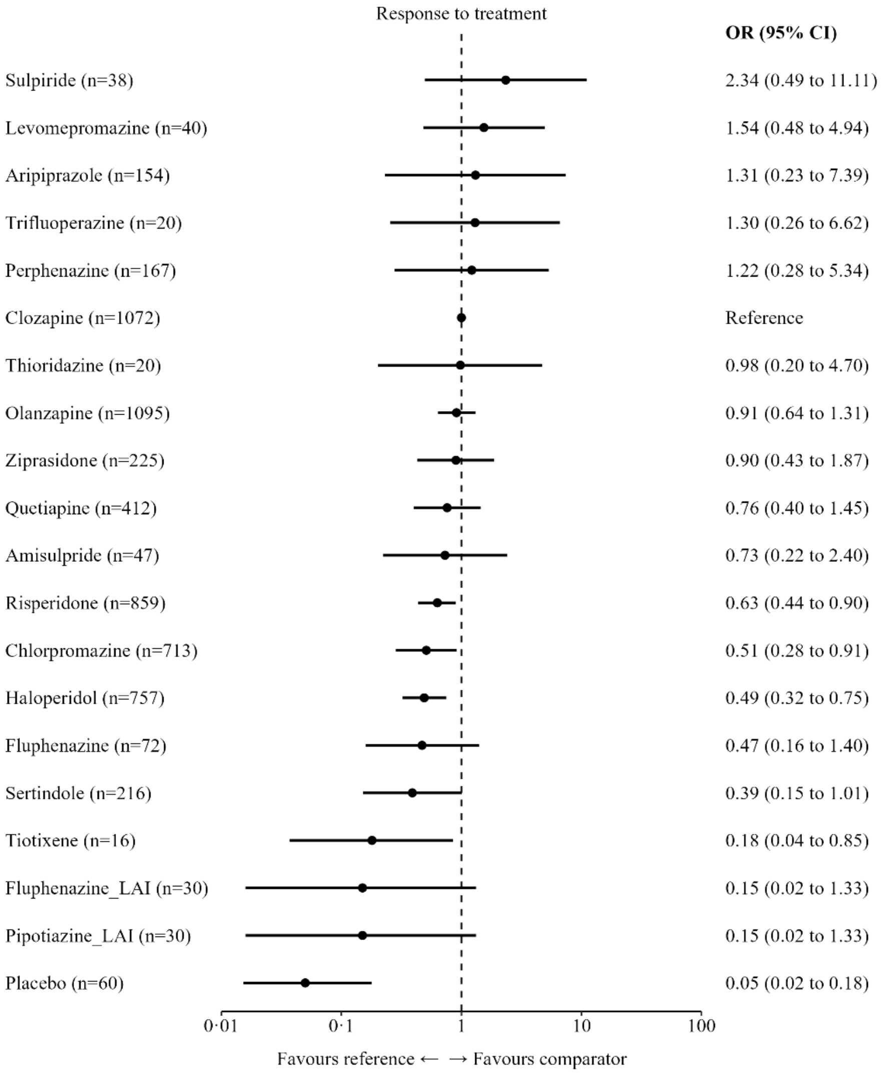

Effect sizes, measured as odds ratio (OR), are from the network meta-analysis. Order of treatments is according to the mean effect size. Reference is clozapine. The direction of the effect is indicated below the x-axis.

Forest plot with RR and EER versus CER

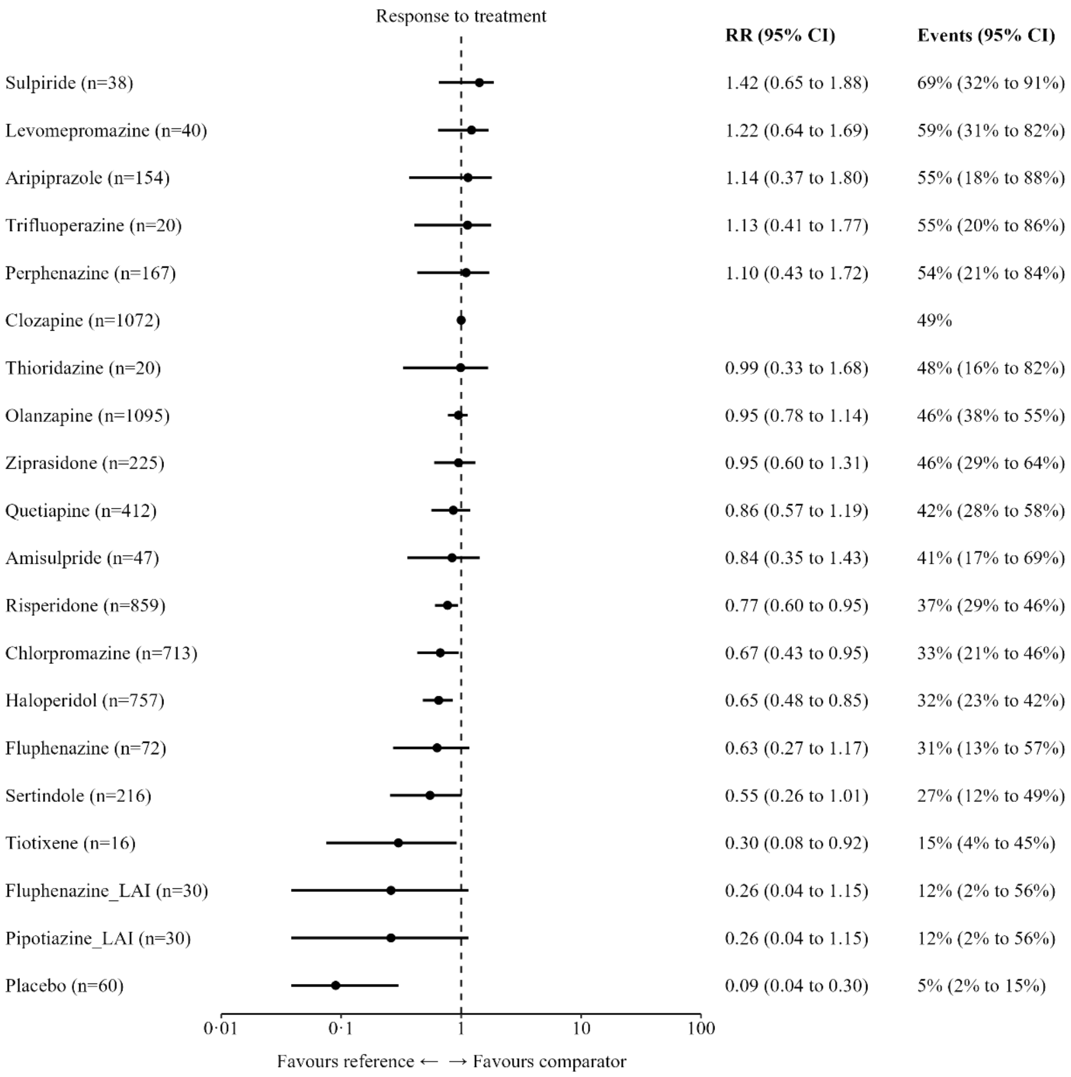

For this forest plot, we transformed the original OR to RR and exposure events rates (EER, called "events" in the forest plot) using the formula indicated above. Therefore, we used an average response rate with clozapine of 49% as the control event rate (CER).
Effect sizes are from the network meta-analysis. Order of treatments is according to the mean effect size. Reference is clozapine. The direction of the effect is indicated below the x-axis.

League table for the outcome: Response to treatment (OR)

**Sulpiride**	NA	NA	NA	NA	NA	NA	NA	NA	NA	NA	NA	**4.60 (1.09 to 19.47)**	NA	NA	NA	NA	NA	NA	NA
1.52 (0.24 to 9.46)	**Levomepromazine**	NA	NA	1.28 (0.27 to 6.13)	NA	NA	NA	NA	NA	NA	NA	2.98 (0.65 to 13.64)	3.20 (0.68 to 15.12)	NA	NA	NA	NA	NA	NA
1.79 (0.19 to 17.31)	1.18 (0.21 to 6.59)	**Aripiprazole**	NA	1.07 (0.43 to 2.65)	NA	NA	NA	NA	NA	NA	NA	NA	NA	NA	NA	NA	NA	NA	NA
1.80 (0.22 to 14.90)	1.19 (0.18 to 7.92)	1.01 (0.10 to 10.19)	**Trifluoperazine**	NA	NA	1.33 (0.25 to 7.05)	NA	NA	NA	NA	NA	2.15 (0.43 to 10.78)	NA	NA	NA	NA	NA	NA	**36.00 (5.02 to 258.37)**
1.92 (0.24 to 15.37)	1.27 (0.29 to 5.46)	1.07 (0.43 to 2.65)	1.07 (0.13 to 8.99)	**Perphenazine**	NA	NA	NA	NA	NA	NA	NA	NA	2.50 (0.55 to 11.39)	NA	NA	NA	NA	NA	NA
2.34 (0.49 to 11.11)	1.54 (0.48 to 4.94)	1.31 (0.23 to 7.39)	1.30 (0.26 to 6.62)	1.22 (0.28 to 5.34)	**Clozapine**	NA	1.14 (0.72 to 1.80)	0.61 (0.23 to 1.66)	NA	NA	1.25 (0.79 to 1.99)	**12.37 (3.98 to 38.43)**	**1.88 (1.05 to 3.39)**	NA	NA	NA	NA	NA	NA
2.40 (0.30 to 19.04)	1.58 (0.25 to 10.08)	1.34 (0.14 to 13.08)	1.33 (0.25 to 7.05)	1.25 (0.15 to 10.11)	1.03 (0.21 to 4.94)	**Thioridazine**	NA	NA	NA	NA	NA	1.62 (0.34 to 7.65)	NA	NA	NA	NA	NA	NA	**27.00 (3.94 to 185.22)**
2.57 (0.53 to 12.40)	1.69 (0.52 to 5.51)	1.43 (0.25 to 8.18)	1.42 (0.28 to 7.37)	1.34 (0.30 to 5.92)	1.10 (0.76 to 1.57)	1.07 (0.22 to 5.24)	**Olanzapine**	NA	0.67 (0.11 to 3.94)	1.25 (0.40 to 3.88)	**1.68 (1.02 to 2.78)**	7.53 (0.34 to 164.72)	**2.04 (1.02 to 4.06)**	NA	NA	NA	NA	NA	NA
2.62 (0.53 to 13.00)	1.72 (0.48 to 6.22)	1.46 (0.23 to 9.17)	1.45 (0.27 to 7.83)	1.36 (0.27 to 6.74)	1.12 (0.54 to 2.33)	1.09 (0.21 to 5.57)	1.02 (0.46 to 2.25)	**Ziprasidone**	NA	NA	NA	1.12 (0.47 to 2.66)	NA	NA	NA	NA	NA	NA	NA
3.07 (0.64 to 14.65)	2.03 (0.61 to 6.76)	1.71 (0.29 to 10.11)	1.70 (0.33 to 8.79)	1.60 (0.35 to 7.35)	1.31 (0.69 to 2.49)	1.28 (0.26 to 6.24)	1.20 (0.62 to 2.32)	1.18 (0.50 to 2.76)	**Quetiapine**	NA	1.84 (0.46 to 7.38)	1.17 (0.55 to 2.49)	1.76 (0.73 to 4.23)	1.83 (0.22 to 15.39)	NA	NA	NA	NA	NA
3.21 (0.46 to 22.35)	2.12 (0.41 to 10.87)	1.79 (0.22 to 14.31)	1.78 (0.24 to 13.12)	1.67 (0.26 to 10.84)	1.37 (0.42 to 4.50)	1.34 (0.19 to 9.41)	1.25 (0.40 to 3.88)	1.23 (0.31 to 4.89)	1.05 (0.28 to 3.89)	**Amisulpride**	NA	NA	NA	NA	NA	NA	NA	NA	NA
3.75 (0.78 to 18.04)	2.47 (0.76 to 8.04)	2.09 (0.37 to 11.94)	2.08 (0.40 to 10.73)	1.95 (0.44 to 8.64)	**1.60 (1.11 to 2.30)**	1.56 (0.32 to 7.62)	1.46 (0.99 to 2.14)	1.43 (0.65 to 3.16)	1.22 (0.63 to 2.35)	1.17 (0.35 to 3.86)	**Risperidone**	0.18 (0.03 to 1.15)	1.30 (0.59 to 2.87)	1.65 (0.20 to 13.71)	1.60 (0.67 to 3.86)	NA	NA	NA	NA
**4.60 (1.09 to 19.47)**	3.03 (0.98 to 9.35)	2.57 (0.45 to 14.77)	2.55 (0.55 to 11.95)	2.39 (0.54 to 10.69)	**1.96 (1.09 to 3.53)**	1.92 (0.43 to 8.45)	1.79 (0.95 to 3.37)	1.76 (0.87 to 3.55)	1.50 (0.82 to 2.73)	1.43 (0.39 to 5.25)	1.23 (0.66 to 2.30)	**Chlorpromazine**	0.25 (0.03 to 2.08)	NA	NA	NA	NA	NA	**19.76 (4.22 to 92.52)**
4.77 (1.00 to 22.73)	**3.14 (1.01 to 9.71)**	2.66 (0.49 to 14.47)	2.64 (0.52 to 13.43)	2.48 (0.59 to 10.38)	**2.03 (1.33 to 3.10)**	1.98 (0.41 to 9.53)	**1.86 (1.18 to 2.91)**	1.82 (0.83 to 4.02)	1.55 (0.85 to 2.84)	1.48 (0.44 to 5.02)	1.27 (0.80 to 2.01)	1.03 (0.57 to 1.89)	**Haloperidol**	0.96 (0.29 to 3.26)	NA	1.96 (0.39 to 9.73)	NA	NA	3.85 (0.68 to 21.98)
4.95 (0.78 to 31.46)	3.26 (0.72 to 14.84)	2.76 (0.38 to 19.95)	2.75 (0.41 to 18.47)	2.57 (0.44 to 14.94)	2.11 (0.71 to 6.26)	2.06 (0.32 to 13.22)	1.93 (0.64 to 5.78)	1.89 (0.53 to 6.75)	1.61 (0.52 to 5.03)	1.54 (0.32 to 7.47)	1.32 (0.44 to 3.93)	1.08 (0.34 to 3.42)	1.04 (0.37 to 2.91)	**Fluphenazine**	NA	NA	NA	NA	NA
6.00 (0.99 to 36.34)	3.96 (0.91 to 17.23)	3.35 (0.48 to 23.57)	3.33 (0.52 to 21.44)	3.12 (0.55 to 17.59)	2.56 (0.99 to 6.62)	2.50 (0.41 to 15.33)	2.34 (0.90 to 6.10)	2.30 (0.70 to 7.48)	1.95 (0.65 to 5.85)	1.87 (0.42 to 8.25)	1.60 (0.67 to 3.86)	1.30 (0.44 to 3.83)	1.26 (0.47 to 3.39)	1.21 (0.30 to 4.92)	**Sertindole**	NA	NA	NA	NA
**13.22 (1.54 to 113.29)**	**8.71 (1.33 to 56.98)**	7.37 (0.76 to 71.55)	7.33 (0.88 to 61.29)	6.87 (0.85 to 55.28)	**5.64 (1.17 to 27.13)**	5.50 (0.69 to 44.08)	**5.15 (1.06 to 25.01)**	5.05 (0.93 to 27.35)	4.30 (0.85 to 21.69)	4.11 (0.59 to 28.79)	3.53 (0.72 to 17.18)	2.87 (0.58 to 14.12)	2.77 (0.60 to 12.78)	2.67 (0.43 to 16.77)	2.20 (0.36 to 13.45)	**Tiotixene**	NA	NA	1.97 (0.33 to 11.93)
**16.15 (1.18 to 221.59)**	10.64 (0.94 to 120.56)	9.00 (0.57 to 141.77)	8.96 (0.73 to 109.56)	8.40 (0.62 to 113.45)	6.89 (0.75 to 62.90)	6.72 (0.57 to 79.31)	6.29 (0.68 to 57.96)	6.17 (0.64 to 60.02)	5.25 (0.56 to 48.97)	5.03 (0.42 to 60.86)	4.31 (0.47 to 39.74)	3.51 (0.39 to 31.23)	3.39 (0.38 to 30.47)	3.26 (0.29 to 36.56)	2.69 (0.25 to 29.30)	1.22 (0.10 to 14.24)	**Fluphenazine_LAI**	1.00 (0.26 to 3.78)	2.79 (0.44 to 17.49)
**16.15 (1.18 to 221.59)**	10.64 (0.94 to 120.56)	9.00 (0.57 to 141.77)	8.96 (0.73 to 109.56)	8.40 (0.62 to 113.45)	6.89 (0.75 to 62.90)	6.72 (0.57 to 79.31)	6.29 (0.68 to 57.96)	6.17 (0.64 to 60.02)	5.25 (0.56 to 48.97)	5.03 (0.42 to 60.86)	4.31 (0.47 to 39.74)	3.51 (0.39 to 31.23)	3.39 (0.38 to 30.47)	3.26 (0.29 to 36.56)	2.69 (0.25 to 29.30)	1.22 (0.10 to 14.24)	1.00 (0.26 to 3.78)	**Pipotiazine_LAI**	2.79 (0.44 to 17.49)
**44.99 (6.96 to 290.82)**	**29.64 (6.07 to 144.83)**	**25.08 (3.21 to 195.81)**	**24.95 (4.55 to 136.76)**	**23.39 (3.70 to 147.96)**	**19.19 (5.61 to 65.72)**	**18.71 (3.60 to 97.32)**	**17.51 (5.03 to 61.03)**	**17.20 (4.50 to 65.72)**	**14.64 (4.12 to 52.01)**	**14.01 (2.59 to 75.60)**	**12.01 (3.45 to 41.84)**	**9.77 (2.99 to 31.96)**	**9.44 (2.83 to 31.45)**	**9.09 (1.89 to 43.67)**	**7.49 (1.63 to 34.45)**	3.40 (0.67 to 17.35)	2.79 (0.44 to 17.49)	2.79 (0.44 to 17.49)	**Placebo**

Treatments are presented in order of efficacy ranking. Results of the network meta-analysis are reported in the left lower half and results of pairwise meta-analyses in the right upper half. Each cell provides the effect estimate and the corresponding 95% credible interval (95% CI) of a comparison (left lower half: treatment in column versus treatment in row; right upper half: treatment in row versus treatment in column). The type of effect size measure is odd ratio (OR). Bold results indicate 95% CI excluding no effect. NA=not available.

League table for the outcome: Response to treatment (RR)

Sulpiride	NA	NA	NA	NA	NA	NA	NA	NA	NA	NA	NA	**2.16 (1.06 to 2.87)**	NA	NA	NA	NA	NA	NA	NA
1.17 (0.43 to 1.6)	Levomepromazine	NA	NA	1.12 (0.44 to 1.66)	NA	NA	NA	NA	NA	NA	NA	1.84 (0.73 to 2.75)	1.91 (0.75 to 2.84)	NA	NA	NA	NA	NA	NA
1.25 (0.34 to 1.76)	1.08 (0.37 to 1.64)	Aripiprazole	NA	1.03 (0.61 to 1.42)	NA	NA	NA	NA	NA	NA	NA	NA	NA	NA	NA	NA	NA	NA	NA
1.26 (0.38 to 1.76)	1.08 (0.32 to 1.68)	1 (0.19 to 1.71)	Trifluoperazine	NA	NA	1.15 (0.38 to 1.84)	NA	NA	NA	NA	NA	1.58 (0.52 to 2.66)	NA	NA	NA	NA	NA	NA	**14.03 (4.26 to 20.65)**
1.3 (0.4 to 1.81)	1.11 (0.46 to 1.64)	1.03 (0.61 to 1.42)	1.03 (0.24 to 1.74)	Perphenazine	NA	NA	NA	NA	NA	NA	NA	NA	1.71 (0.64 to 2.72)	NA	NA	NA	NA	NA	NA
1.43 (0.65 to 1.92)	1.23 (0.64 to 1.73)	1.14 (0.36 to 1.84)	1.14 (0.4 to 1.81)	1.11 (0.42 to 1.75)	Clozapine	NA	1.07 (0.82 to 1.32)	0.74 (0.35 to 1.28)	NA	NA	1.15 (0.85 to 1.47)	**2.71 (2.06 to 3.02)**	**1.48 (1.03 to 1.96)**	NA	NA	NA	NA	NA	NA
1.45 (0.45 to 2.02)	1.24 (0.38 to 1.92)	1.16 (0.23 to 1.97)	1.15 (0.38 to 1.84)	1.12 (0.25 to 1.93)	1.02 (0.33 to 1.74)	Thioridazine	NA	NA	NA	NA	NA	1.36 (0.43 to 2.48)	NA	NA	NA	NA	NA	NA	**12.48 (3.48 to 20.05)**
1.5 (0.67 to 2.02)	1.29 (0.66 to 1.82)	1.2 (0.38 to 1.93)	1.19 (0.41 to 1.9)	1.16 (0.44 to 1.84)	1.05 (0.85 to 1.25)	1.04 (0.34 to 1.8)	Olanzapine	NA	0.77 (0.17 to 1.79)	1.14 (0.52 to 1.81)	**1.35 (1.01 to 1.7)**	2.47 (0.43 to 3.15)	**1.55 (1.01 to 2.1)**	NA	NA	NA	NA	NA	NA
1.52 (0.67 to 2.05)	1.3 (0.62 to 1.87)	1.21 (0.35 to 1.98)	1.21 (0.4 to 1.94)	1.17 (0.4 to 1.89)	1.06 (0.68 to 1.46)	1.05 (0.32 to 1.83)	1.01 (0.61 to 1.44)	Ziprasidone	NA	NA	NA	1.08 (0.56 to 1.75)	NA	NA	NA	NA	NA	NA	NA
1.67 (0.75 to 2.24)	1.43 (0.72 to 2.02)	1.33 (0.41 to 2.15)	1.32 (0.45 to 2.11)	1.29 (0.48 to 2.05)	1.16 (0.79 to 1.55)	1.15 (0.37 to 1.99)	1.11 (0.73 to 1.51)	1.1 (0.63 to 1.61)	Quetiapine	NA	1.41 (0.57 to 2.24)	1.11 (0.64 to 1.7)	1.43 (0.8 to 2.13)	1.47 (0.29 to 2.91)	NA	NA	NA	NA	NA
1.71 (0.58 to 2.37)	1.47 (0.53 to 2.22)	1.36 (0.32 to 2.28)	1.36 (0.34 to 2.27)	1.32 (0.37 to 2.22)	1.2 (0.54 to 1.89)	1.18 (0.28 to 2.18)	1.14 (0.52 to 1.81)	1.13 (0.43 to 1.93)	1.03 (0.39 to 1.82)	Amisulpride	NA	NA	NA	NA	NA	NA	NA	NA	NA
1.89 (0.85 to 2.53)	1.62 (0.83 to 2.28)	1.5 (0.48 to 2.42)	1.5 (0.51 to 2.39)	1.45 (0.55 to 2.31)	**1.32 (1.07 to 1.57)**	1.3 (0.42 to 2.25)	1.25 (0.99 to 1.52)	1.24 (0.74 to 1.78)	1.13 (0.73 to 1.58)	1.1 (0.46 to 1.9)	Risperidone	0.24 (0.04 to 1.1)	1.19 (0.67 to 1.82)	1.38 (0.26 to 2.86)	1.38 (0.73 to 2.22)	NA	NA	NA	NA
**2.16 (1.06 to 2.87)**	1.85 (0.99 to 2.59)	1.72 (0.54 to 2.78)	1.72 (0.64 to 2.7)	1.67 (0.63 to 2.65)	**1.51 (1.06 to 1.97)**	1.49 (0.52 to 2.54)	1.44 (0.97 to 1.94)	1.42 (0.91 to 1.97)	1.3 (0.87 to 1.77)	1.26 (0.48 to 2.25)	1.15 (0.74 to 1.64)	Chlorpromazine	0.32 (0.04 to 1.56)	NA	NA	NA	NA	NA	**10.75 (3.69 to 18.17)**
2.21 (1 to 2.97)	**1.9 (1.01 to 2.65)**	1.76 (0.58 to 2.82)	1.76 (0.61 to 2.79)	1.71 (0.67 to 2.68)	**1.54 (1.21 to 1.89)**	1.52 (0.5 to 2.64)	**1.47 (1.12 to 1.84)**	1.45 (0.88 to 2.09)	1.33 (0.89 to 1.82)	1.29 (0.53 to 2.25)	1.17 (0.85 to 1.53)	1.02 (0.66 to 1.48)	Haloperidol	0.97 (0.37 to 1.95)	NA	1.73 (0.43 to 4.42)	NA	NA	3.41 (0.69 to 11.34)
2.27 (0.83 to 3.12)	1.95 (0.79 to 2.89)	1.81 (0.47 to 3)	1.81 (0.5 to 2.97)	1.75 (0.53 to 2.9)	1.59 (0.78 to 2.44)	1.57 (0.4 to 2.85)	1.51 (0.72 to 2.38)	1.49 (0.62 to 2.49)	1.36 (0.61 to 2.28)	1.33 (0.4 to 2.55)	1.21 (0.53 to 2.1)	1.05 (0.42 to 1.99)	1.03 (0.46 to 1.85)	Fluphenazine	NA	NA	NA	NA	NA
2.61 (0.99 to 3.57)	2.24 (0.93 to 3.3)	2.08 (0.55 to 3.44)	2.07 (0.59 to 3.4)	2.01 (0.62 to 3.31)	1.82 (0.99 to 2.69)	1.8 (0.48 to 3.25)	1.74 (0.92 to 2.62)	1.72 (0.76 to 2.79)	1.56 (0.71 to 2.59)	1.53 (0.49 to 2.86)	1.38 (0.73 to 2.22)	1.21 (0.51 to 2.21)	1.18 (0.55 to 2.09)	1.15 (0.37 to 2.44)	Sertindole	NA	NA	NA	NA
**4.93 (1.43 to 6.89)**	**4.23 (1.27 to 6.55)**	3.93 (0.79 to 6.68)	3.92 (0.89 to 6.59)	3.8 (0.87 to 6.53)	**3.44 (1.14 to 5.9)**	3.4 (0.72 to 6.36)	**3.28 (1.05 to 5.81)**	3.24 (0.94 to 5.91)	2.96 (0.87 to 5.64)	2.88 (0.63 to 5.97)	2.62 (0.75 to 5.33)	2.28 (0.62 to 5.03)	2.23 (0.63 to 4.88)	2.17 (0.47 to 5.29)	1.89 (0.39 to 4.96)	Tiotixene	NA	NA	1.89 (0.34 to 8.01)
**5.88 (1.16 to 8.38)**	5.04 (0.95 to 8.15)	4.68 (0.6 to 8.23)	4.67 (0.75 to 8.1)	4.53 (0.65 to 8.12)	4.1 (0.77 to 7.73)	4.05 (0.6 to 7.91)	3.91 (0.71 to 7.66)	3.87 (0.67 to 7.69)	3.52 (0.59 to 7.5)	3.43 (0.45 to 7.7)	3.12 (0.5 to 7.27)	2.72 (0.42 to 6.96)	2.66 (0.41 to 6.93)	2.59 (0.32 to 7.17)	2.25 (0.27 to 6.87)	1.19 (0.11 to 5.64)	Fluphenazine_LAI	1 (0.28 to 2.86)	2.58 (0.45 to 10.07)
**5.88 (1.16 to 8.38)**	5.04 (0.95 to 8.15)	4.68 (0.6 to 8.23)	4.67 (0.75 to 8.1)	4.53 (0.65 to 8.12)	4.1 (0.77 to 7.73)	4.05 (0.6 to 7.91)	3.91 (0.71 to 7.66)	3.87 (0.67 to 7.69)	3.52 (0.59 to 7.5)	3.43 (0.45 to 7.7)	3.12 (0.5 to 7.27)	2.72 (0.42 to 6.96)	2.66 (0.41 to 6.93)	2.59 (0.32 to 7.17)	2.25 (0.27 to 6.87)	1.19 (0.11 to 5.64)	1 (0.28 to 2.86)	Pipotiazine_LAI	2.58 (0.45 to 10.07)
**15.16 (5.5 to 20.83)**	**13 (4.95 to 19.49)**	**12.08 (2.92 to 20.16)**	**12.05 (3.93 to 19.34)**	**11.69 (3.3 to 19.54)**	**10.58 (4.65 to 16.88)**	**10.44 (3.23 to 18.34)**	**10.07 (4.26 to 16.56)**	**9.97 (3.89 to 16.88)**	**9.09 (3.62 to 15.85)**	**8.86 (2.42 to 17.44)**	**8.05 (3.11 to 14.8)**	**7.02 (2.75 to 13.4)**	**6.85 (2.62 to 13.32)**	**6.68 (1.82 to 15.02)**	**5.81 (1.59 to 13.8)**	3.07 (0.68 to 10.02)	2.58 (0.45 to 10.07)	2.58 (0.45 to 10.07)	Placebo

The original results given in OR (and their 95% CI) are transformed to RR (left lower half: treatment in column versus treatment in row; right upper half: treatment in row versus treatment in column) using the formula described above. For this transformation, we assumed a response rate with clozapine of 49% as the control event rate (CER) for all comparisons of active antipsychotic versus clozapine.

Treatments are presented in order of efficacy ranking. Results of the network meta-analysis are reported in the left lower half and results of pairwise meta-analyses in the right upper half. Bold results indicate 95% CI excluding no effect. NA=not available.

Forest plot of results of pairwise meta-analyses

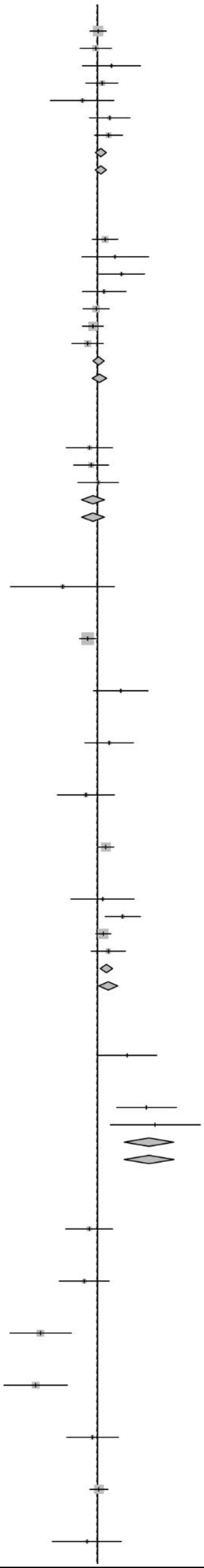

Study	Experimental Events	Experimental Total	Control Events	Control Total	OR	95%-CI	Weight (common)	Weight (random)
comparison = Clozapine_Risperidone								
Azorin 2001 Clozapine Risperidone	80	138	77	135	1.04	[0.64; 1.68]	4.3%	2.5%
Bondolfi 1998 Clozapine Risperidone	28	43	29	43	0.90	[0.37; 2.20]	1.3%	1.9%
Breier 1999 Clozapine Risperidone	5	14	3	15	2.22	[0.42; 11.83]	0.2%	1.0%
Volavka 2002 Clozapine Risperidone	15	40	13	41	1.29	[0.52; 3.24]	1.1%	1.8%
Wahlbeck 2000 Clozapine Risperidone	5	11	6	9	0.42	[0.07; 2.58]	0.5%	0.9%
Chowdhury 1999 Clozapine Risperidone	24	30	20	30	2.00	[0.62; 6.46]	0.5%	1.5%
Schooler 2016 Clozapine Risperidone	38	53	31	54	1.88	[0.84; 4.20]	1.1%	2.0%
Common effect model		329		327	1.21	[0.88; 1.66]	9.1%	--
Random effects model					1.21	[0.88; 1.67]	--	11.6%
Heterogeneity: $I^2 = 0\%$, $\tau^2 = 0$, $p = 0.61$								
comparison = Clozapine_Olanzapine								
Bitter 2004 Clozapine Olanzapine	56	72	52	75	1.55	[0.74; 3.25]	1.5%	2.1%
Shaw 2006 Clozapine Olanzapine	4	12	2	13	2.75	[0.40; 18.88]	0.2%	0.8%
Kumra 2007 Clozapine Olanzapine	12	18	7	21	4.00	[1.05; 15.21]	0.3%	1.3%
Meltzer 2008 Clozapine Olanzapine	13	21	10	19	1.46	[0.41; 5.15]	0.5%	1.4%
Naber 2005 Clozapine Olanzapine	22	57	23	57	0.93	[0.44; 1.97]	1.9%	2.1%
Tollefson 2001 Clozapine Olanzapine	47	90	53	90	0.76	[0.42; 1.38]	3.3%	2.3%
Volavka 2002 Clozapine Olanzapine	15	40	20	39	0.57	[0.23; 1.40]	1.7%	1.9%
Common effect model		310		314	1.06	[0.77; 1.47]	9.3%	--
Random effects model					1.12	[0.73; 1.70]	--	11.9%
Heterogeneity: $I^2 = 34\%$, $\tau^2 = 0.0922$, $p = 0.17$								
comparison = Haloperidol_Risperidone								
Claus 1992 Haloperidol Risperidone	5	22	7	22	0.63	[0.16; 2.41]	0.7%	1.3%
Volavka 2002 Haloperidol Risperidone	9	37	13	41	0.69	[0.26; 1.88]	1.2%	1.7%
Wirshing 1999 Haloperidol Risperidone	7	33	7	34	1.04	[0.32; 3.37]	0.7%	1.5%
Common effect model		92		97	0.77	[0.40; 1.49]	2.6%	--
Random effects model					0.77	[0.40; 1.49]	--	4.5%
Heterogeneity: $I^2 = 0\%$, $\tau^2 = 0$, $p = 0.83$								
comparison = Chlorpromazine_Olanzapine								
Conley 1998 Chlorpromazine Olanzapine	0	42	3	42	0.13	[0.01; 2.65]	0.5%	0.4%
comparison = Haloperidol_Quetiapine								
Emsley 2000 Haloperidol Quetiapine	54	145	73	143	0.57	[0.36; 0.91]	6.1%	2.5%
comparison = Haloperidol_Placebo								
Howard 1974 Haloperidol Placebo	8	17	3	16	3.85	[0.80; 18.62]	0.2%	1.1%
comparison = Haloperidol_Tiotixene								
Howard 1974 Haloperidol Tiotixene	8	17	5	16	1.96	[0.47; 8.11]	0.4%	1.2%
comparison = Placebo_Tiotixene								
Howard 1974 Placebo Tiotixene	3	16	5	16	0.51	[0.10; 2.62]	0.5%	1.0%
comparison = Risperidone_Sertindole								
Kane 2010b Risperidone Sertindole	59	105	96	216	1.60	[1.00; 2.56]	3.6%	2.5%
comparison = Clozapine_Haloperidol								
Kumra 1996 Clozapine Haloperidol	7	10	7	11	1.33	[0.21; 8.29]	0.3%	0.9%
Kane 2001 Clozapine Haloperidol	21	37	8	34	4.27	[1.53; 11.89]	0.5%	1.7%
Rosenheck 1997 Clozapine Haloperidol	61	205	51	218	1.39	[0.90; 2.14]	4.6%	2.5%
Volavka 2002 Clozapine Haloperidol	15	40	9	37	1.87	[0.70; 5.01]	0.8%	1.7%
Common effect model		292		300	1.67	[1.17; 2.39]	6.1%	--
Random effects model					1.85	[1.07; 3.20]	--	6.8%
Heterogeneity: $I^2 = 25\%$, $\tau^2 = 0.1059$, $p = 0.26$								
comparison = Chlorpromazine_Risperidone								
Mercer 1997 Chlorpromazine Risperidone	7	12	3	15	5.60	[1.01; 30.90]	0.1%	1.0%
comparison = Chlorpromazine_Placebo								
Schiele 1961, 06602 Chlorpromazine Placebo	13	20	2	20	16.71	[2.98; 93.89]	0.1%	1.0%
Dean 1958 Chlorpromazine Placebo	8	9	2	9	28.00	[2.07; 379.25]	0.0%	0.5%
Common effect model		29		29	19.43	[4.61; 81.95]	0.1%	--
Random effects model					19.56	[4.64; 82.47]	--	1.5%
Heterogeneity: $I^2 = 0\%$, $\tau^2 = 0$, $p = 0.75$								
comparison = Chlorpromazine_Thioridazine								
Schiele 1961, 06602 Chlorpromazine Thioridazine	13	20	15	20	0.62	[0.16; 2.43]	0.7%	1.3%
comparison = Chlorpromazine_Trifluoperazine								
Schiele 1961, 06602 Chlorpromazine Trifluoperazine	13	20	16	20	0.46	[0.11; 1.94]	0.7%	1.2%
comparison = Placebo_Thioridazine								
Schiele 1961, 06602 Placebo Thioridazine	2	20	15	20	0.04	[0.01; 0.22]	1.8%	0.9%
comparison = Placebo_Trifluoperazine								
Schiele 1961, 06602 Placebo Trifluoperazine	2	20	16	20	0.03	[0.00; 0.17]	1.9%	0.9%
comparison = Thioridazine_Trifluoperazine								
Schiele 1961, 06602 Thioridazine Trifluoperazine	15	20	16	20	0.75	[0.17; 3.33]	0.5%	1.2%
comparison = Aripiprazole_Perphenazine								
Kane 2007a Aripiprazole Perphenazine	40	154	36	146	1.07	[0.64; 1.81]	3.6%	2.4%
comparison = Fluphenazine_Quetiapine								
Conley 2005 Fluphenazine Quetiapine	2	13	3	12	0.55	[0.07; 4.01]	0.3%	0.8%

comparison = Fluphenazine_Risperidone									
Conley 2005 Fluphenazine Risperidone	2	13	3	13	0.61	[0.08;	4.41]	0.3%	0.8%
comparison = Quetiapine_Risperidone									
Conley 2005 Quetiapine Risperidone	3	12	3	13	1.11	[0.18;	6.97]	0.3%	0.9%
Suzuki 2007 Quetiapine Risperidone	5	10	3	12	3.00	[0.50;	18.17]	0.2%	0.9%
Common effect model		22		25	1.84	[0.52;	6.53]	0.5%	--
Random effects model					1.84	[0.51;	6.67]	--	1.8%
Heterogeneity: $I^2 = 0\%$, $\tau^2 = 0$, $p = 0.45$									
comparison = Chlorpromazine_Clozapine									
Hong 1997 Chlorpromazine Clozapine	0	19	6	21	0.06	[0.00;	1.17]	0.8%	0.4%
Kane 1988 Chlorpromazine Clozapine	5	142	38	126	0.08	[0.03;	0.22]	5.1%	1.8%
Common effect model		161		147	0.08	[0.03;	0.20]	5.9%	--
Random effects model					0.08	[0.03;	0.21]	--	2.2%
Heterogeneity: $I^2 = 0\%$, $\tau^2 = 0$, $p = 0.84$									
comparison = Chlorpromazine_Ziprasidone									
Kane 2006 Chlorpromazine Ziprasidone	85	154	88	152	0.90	[0.57;	1.41]	5.2%	2.5%
comparison = Chlorpromazine_Haloperidol									
McCreadie 1977 Chlorpromazine Haloperidol	2	10	5	10	0.25	[0.03;	1.82]	0.5%	0.8%
comparison = Clozapine_Ziprasidone									
Sacchetti 2009 Clozapine Ziprasidone	40	74	48	73	0.61	[0.32;	1.19]	2.9%	2.2%
comparison = Haloperidol_Olanzapine									
Volavka 2002 Haloperidol Olanzapine	9	37	20	39	0.31	[0.11;	0.81]	1.9%	1.7%
Breier 1999a Haloperidol Olanzapine	60	174	163	352	0.61	[0.42;	0.89]	9.3%	2.6%
Common effect model		211		391	0.56	[0.39;	0.79]	11.2%	--
Random effects model					0.50	[0.27;	0.92]	--	4.3%
Heterogeneity: $I^2 = 40\%$, $\tau^2 = 0.0966$, $p = 0.20$									
comparison = Olanzapine_Risperidone									
Volavka 2002 Olanzapine Risperidone	20	39	13	41	2.27	[0.91;	5.63]	0.8%	1.8%
Alvarez 2006 Olanzapine Risperidone	83	124	56	123	2.42	[1.45;	4.06]	2.4%	2.4%
Chen 2012 Olanzapine Risperidone	5	16	4	16	1.36	[0.29;	6.42]	0.4%	1.1%
Suzuki 2007 Olanzapine Risperidone	6	15	3	12	2.00	[0.38;	10.58]	0.3%	1.0%
Kinon 2009 Olanzapine Risperidone	94	186	94	192	1.07	[0.71;	1.59]	6.0%	2.6%
Common effect model		380		384	1.53	[1.15;	2.05]	9.9%	--
Random effects model					1.68	[1.04;	2.70]	--	8.9%
Heterogeneity: $I^2 = 43\%$, $\tau^2 = 0.1217$, $p = 0.14$									
comparison = Chlorpromazine_Levomepromazine									
Lal 2006 Chlorpromazine Levomepromazine	6	19	11	19	0.34	[0.09;	1.27]	1.0%	1.3%
comparison = Fluphenazine_Haloperidol									
Hall 1968 Fluphenazine Haloperidol	8	25	8	25	1.00	[0.30;	3.28]	0.7%	1.5%
Kinon 1993b Fluphenazine Haloperidol	3	34	1	13	1.16	[0.11;	12.29]	0.2%	0.6%
Common effect model		59		38	1.03	[0.36;	2.97]	0.9%	--
Random effects model					1.03	[0.36;	2.98]	--	2.1%
Heterogeneity: $I^2 = 0\%$, $\tau^2 = 0$, $p = 0.91$									
comparison = Chlorpromazine_Sulpiride									
Toru 1972 Chlorpromazine Sulpiride	24	37	34	38	0.22	[0.06;	0.75]	1.5%	1.4%
comparison = Amisulpride_Olanzapine									
Kahn 2018 Amisulpride Olanzapine	15	47	17	46	0.80	[0.34;	1.88]	1.5%	1.9%
comparison = Chlorpromazine_Quetiapine									
AstraZeneca 5077IL/0031 Chlorpromazine Quetiapine	9	130	10	130	0.89	[0.35;	2.27]	1.2%	1.8%
AstraZeneca 5077IL/0054 Chlorpromazine Quetiapine	22	119	25	117	0.83	[0.44;	1.58]	2.7%	2.2%
Common effect model		249		247	0.85	[0.50;	1.45]	3.9%	--
Random effects model					0.85	[0.50;	1.45]	--	4.0%
Heterogeneity: $I^2 = 0\%$, $\tau^2 = 0$, $p = 0.91$									
comparison = Haloperidol_Levomepromazine									
Shalev 1993 Haloperidol Levomepromazine	9	18	16	21	0.31	[0.08;	1.22]	1.0%	1.3%
comparison = Haloperidol_Perphenazine									
Shalev 1993 Haloperidol Perphenazine	9	18	15	21	0.40	[0.11;	1.50]	0.9%	1.3%
comparison = Levomepromazine_Perphenazine									
Shalev 1993 Levomepromazine Perphenazine	16	21	15	21	1.28	[0.32;	5.09]	0.5%	1.3%
comparison = Olanzapine_Quetiapine									
Suzuki 2007 Olanzapine Quetiapine	6	15	5	10	0.67	[0.13;	3.35]	0.5%	1.1%
comparison = Fluphenazine_LAI_Pipotiazine_LAI									
Schlosberg 1978 Fluphenazine_LAI Pipotiazine_LAI	9	30	9	30	1.00	[0.33;	3.02]	0.8%	1.6%
comparison = Fluphenazine_LAI_Placebo									
Schlosberg 1978 Fluphenazine_LAI Placebo	9	30	2	15	2.79	[0.52;	14.96]	0.2%	1.0%
comparison = Pipotiazine_LAI_Placebo									
Schlosberg 1978 Pipotiazine_LAI Placebo	9	30	2	15	2.79	[0.52;	14.96]	0.2%	1.0%
comparison = Clopenthixol_LAI_Perphenazine_LAI									
Ahlfors 1980 Clopenthixol_LAI Perphenazine_LAI	41	87	35	85	1.27	[0.70;	2.33]	2.5%	2.3%
**Common effect model**		**3358**		**3590**	**0.95**	**[0.86;**	**1.05]**	**100.0%**	**--**
**Random effects model**					**0.96**	**[0.78;**	**1.19]**	**--**	**100.0%**

Heterogeneity: $I^2 = 62\%$, $\tau^2 = 0.4082$, $p < 0.01$
Test for subgroup differences (fixed effect): $\chi^2_{39} = 146.25$, df = 39 ($p < 0.01$)
Test for subgroup differences (random effects): $\chi^2_{39} = 132.96$, df = 39 ($p < 0.01$)

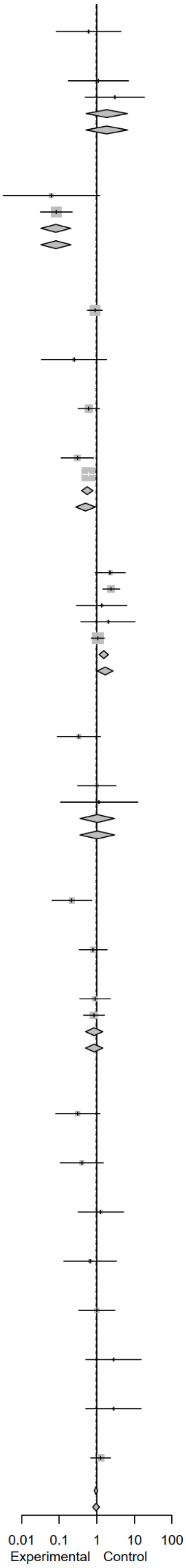

A summary effect size is calculated by pairwise meta-analyses of all studies of a specific comparison. The type of effect size measure is OR.

8.4 Discontinuation due to any reason

We transformed the original ORs to RRs and EERs vs. CER assuming the baseline discontinuation rate due to any reason with clozapine of 35% (namely CER). 35% was the average discontinuation rate due to any reason with clozapine across all clozapine-arms in the network meta-analysis, as estimated by a single-arm meta-analysis of proportions.

Network plot

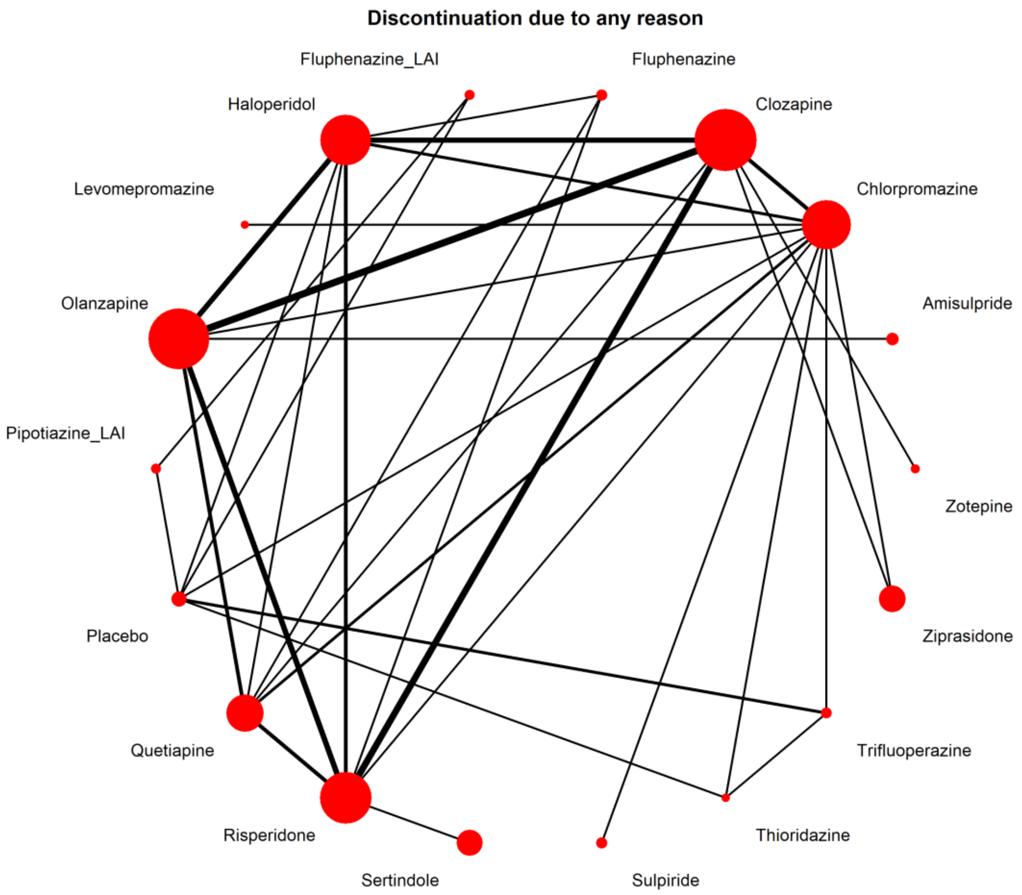

Lines link treatments with direct comparisons in trials; thickness of lines corresponds to the number of trials evaluating the comparison; size of the nodes corresponds to the number of participants assigned to the treatment.

Forest-plot of results of network meta-analysis for antipsychotic drugs versus clozapine

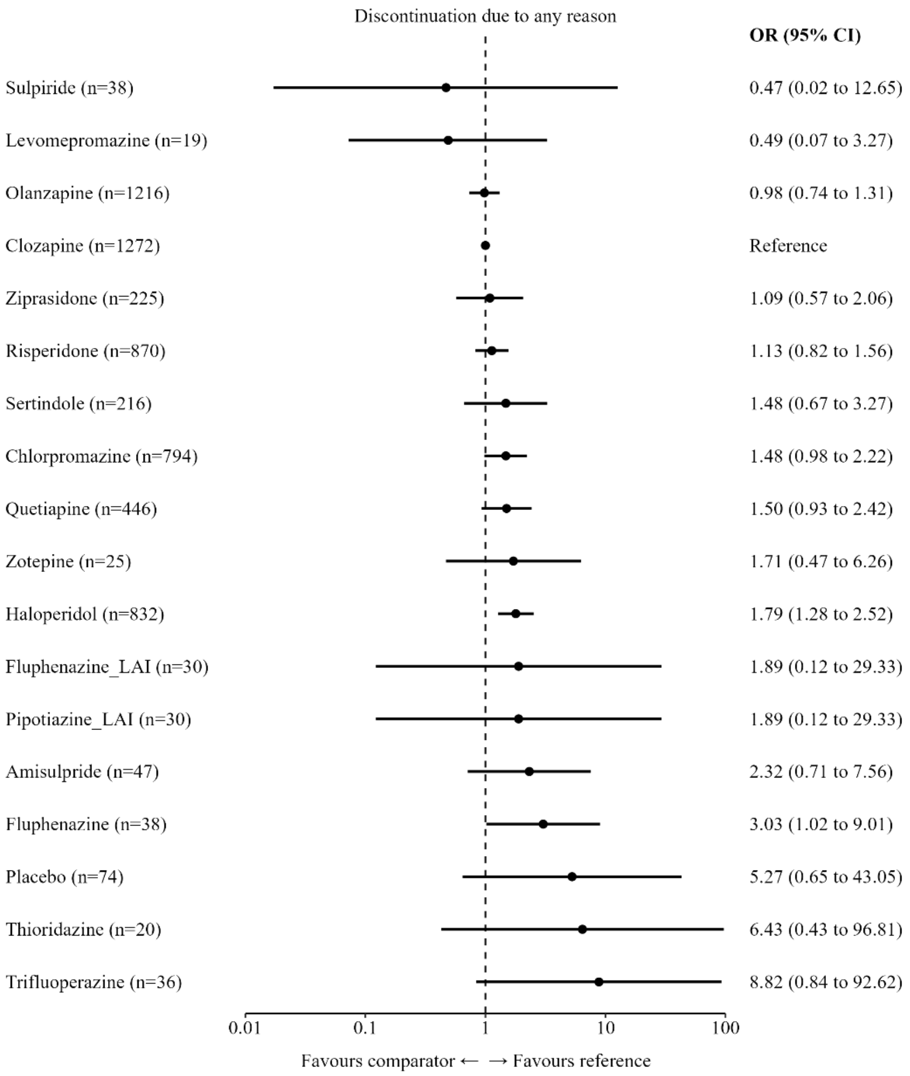

Effect sizes, measured as odds ratio (OR), are from the network meta-analysis. Order of treatments is according to the mean effect size. Reference is clozapine. The direction of the effect is indicated below the x-axis.

Forest plot with RR and EER versus CER

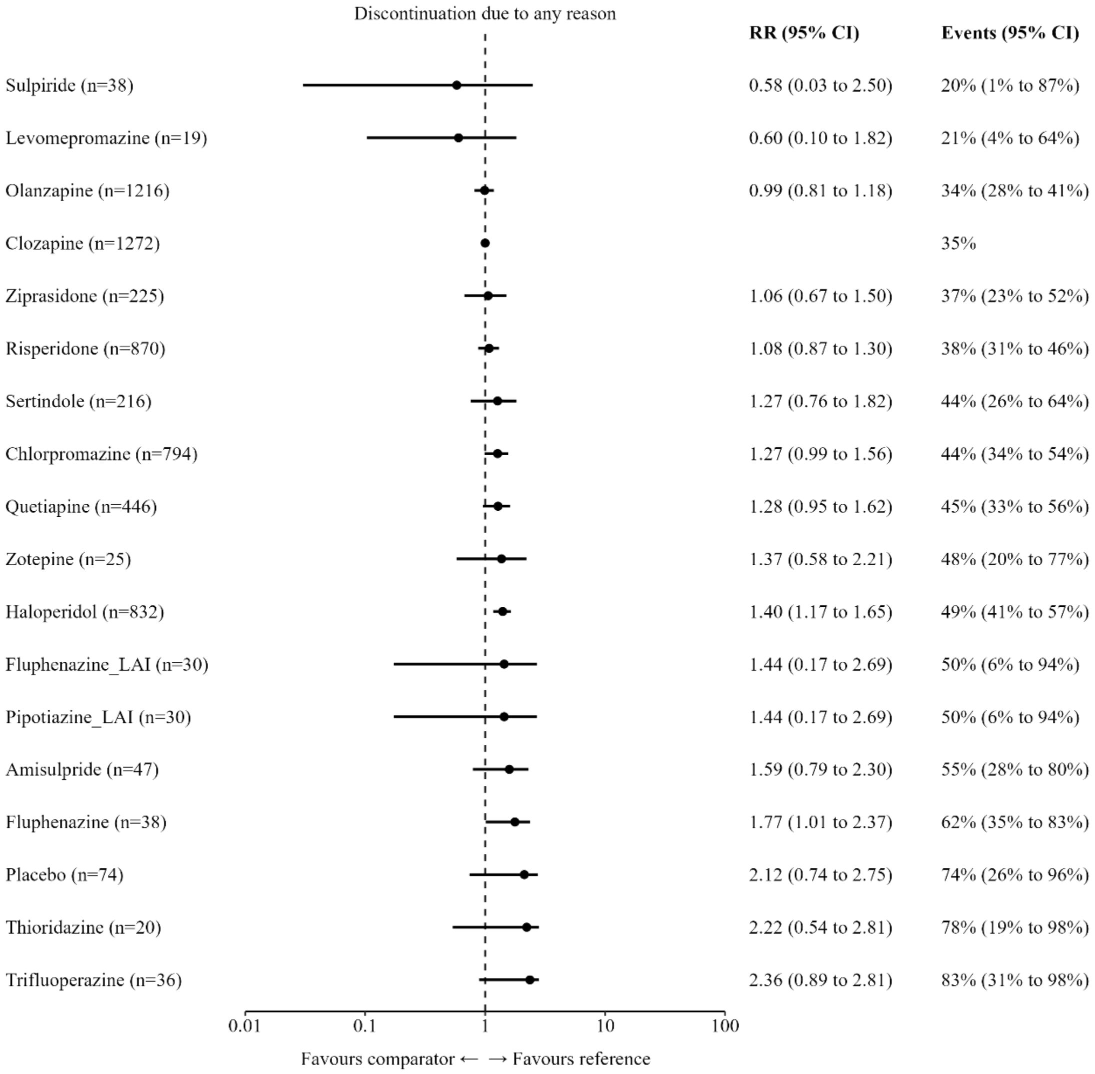

For this forest plot, we transformed the original OR to RR and exposure events rates (EER, called "events" in the forest plot) using the formula indicated above. Therefore, we used an average discontinuation rate (due to any reason) with clozapine of 35% as the control event rate (CER).
Effect sizes are from the network meta-analysis. Order of treatments is according to the mean effect size. Reference is clozapine. The direction of the effect is indicated below the x-axis.

League table for the outcome: Discontinuation due to any reason (OR)

**Sulpiride**	NA	NA	NA	NA	NA	NA	0.32 (0.01 to 8.35)	NA	NA	NA	NA	NA	NA	NA	NA	NA	NA
0.96 (0.02 to 41.48)	**Levomepromazine**	NA	NA	NA	NA	NA	0.33 (0.05 to 2.12)	NA	NA	NA	NA	NA	NA	NA	NA	NA	NA
0.48 (0.02 to 12.96)	0.50 (0.07 to 3.36)	**Olanzapine**	0.90 (0.62 to 1.31)	NA	0.87 (0.57 to 1.33)	NA	0.89 (0.31 to 2.61)	0.79 (0.25 to 2.46)	NA	**0.51 (0.31 to 0.85)**	NA	NA	0.42 (0.13 to 1.33)	NA	NA	NA	NA
0.47 (0.02 to 12.65)	0.49 (0.07 to 3.27)	0.98 (0.74 to 1.31)	**Clozapine**	1.04 (0.44 to 2.41)	0.85 (0.57 to 1.28)	NA	0.76 (0.42 to 1.37)	**0.16 (0.03 to 0.86)**	0.58 (0.16 to 2.13)	**0.52 (0.33 to 0.83)**	NA	NA	NA	NA	NA	NA	NA
0.43 (0.02 to 12.09)	0.45 (0.06 to 3.21)	0.90 (0.46 to 1.79)	0.92 (0.49 to 1.75)	**Ziprasidone**	NA	NA	0.84 (0.35 to 2.01)	NA	NA	NA	NA	NA	NA	NA	NA	NA	NA
0.41 (0.02 to 11.28)	0.43 (0.06 to 2.94)	0.87 (0.62 to 1.20)	0.88 (0.64 to 1.22)	0.96 (0.47 to 1.94)	**Risperidone**	0.77 (0.37 to 1.59)	1.69 (0.13 to 22.44)	0.49 (0.15 to 1.65)	NA	0.89 (0.42 to 1.91)	NA	NA	NA	0.20 (0.03 to 1.13)	NA	NA	NA
0.32 (0.01 to 9.38)	0.33 (0.04 to 2.58)	0.67 (0.30 to 1.48)	0.68 (0.31 to 1.50)	0.74 (0.27 to 2.03)	0.77 (0.37 to 1.59)	**Sertindole**	NA	NA	NA	NA	NA	NA	NA	NA	NA	NA	NA
0.32 (0.01 to 8.35)	0.33 (0.05 to 2.12)	0.66 (0.42 to 1.04)	0.68 (0.45 to 1.02)	0.74 (0.39 to 1.40)	0.77 (0.47 to 1.24)	1.00 (0.42 to 2.40)	**Chlorpromazine**	1.18 (0.69 to 2.01)	NA	2.96 (0.24 to 36.39)	NA	NA	NA	NA	0.32 (0.01 to 8.61)	0.32 (0.01 to 8.61)	0.32 (0.01 to 8.61)
0.31 (0.01 to 8.46)	0.32 (0.05 to 2.19)	0.65 (0.40 to 1.08)	0.67 (0.41 to 1.08)	0.72 (0.35 to 1.51)	0.75 (0.45 to 1.28)	0.98 (0.40 to 2.42)	0.98 (0.63 to 1.53)	**Quetiapine**	NA	1.20 (0.56 to 2.61)	NA	NA	NA	0.32 (0.06 to 1.78)	NA	NA	NA
0.27 (0.01 to 9.43)	0.28 (0.03 to 2.84)	0.57 (0.15 to 2.16)	0.58 (0.16 to 2.13)	0.63 (0.15 to 2.69)	0.66 (0.17 to 2.51)	0.86 (0.19 to 3.94)	0.86 (0.22 to 3.35)	0.88 (0.22 to 3.48)	**Zotepine**	NA	NA	NA	NA	NA	NA	NA	NA
0.26 (0.01 to 7.11)	0.27 (0.04 to 1.85)	**0.55 (0.38 to 0.78)**	**0.56 (0.40 to 0.78)**	0.61 (0.30 to 1.23)	**0.63 (0.42 to 0.94)**	0.82 (0.36 to 1.89)	0.82 (0.51 to 1.32)	0.84 (0.51 to 1.36)	0.96 (0.25 to 3.65)	**Haloperidol**	NA	NA	NA	1.00 (0.23 to 4.40)	0.25 (0.02 to 3.96)	NA	NA
0.25 (0.00 to 17.61)	0.26 (0.01 to 7.04)	0.52 (0.03 to 8.07)	0.53 (0.03 to 8.18)	0.57 (0.03 to 9.43)	0.60 (0.04 to 9.36)	0.78 (0.05 to 13.41)	0.78 (0.05 to 12.06)	0.79 (0.05 to 12.46)	0.91 (0.04 to 18.76)	0.95 (0.06 to 14.57)	**Fluphenazine_LAI**	1.00 (0.29 to 3.40)	NA	NA	0.36 (0.06 to 2.09)	NA	NA
0.25 (0.00 to 17.61)	0.26 (0.01 to 7.04)	0.52 (0.03 to 8.07)	0.53 (0.03 to 8.18)	0.57 (0.03 to 9.43)	0.60 (0.04 to 9.36)	0.78 (0.05 to 13.41)	0.78 (0.05 to 12.06)	0.79 (0.05 to 12.46)	0.91 (0.04 to 18.76)	0.95 (0.06 to 14.57)	1.00 (0.29 to 3.40)	**Pipotiazine_LAI**	NA	NA	0.36 (0.06 to 2.09)	NA	NA
0.20 (0.01 to 6.65)	0.21 (0.02 to 1.95)	0.42 (0.13 to 1.33)	0.43 (0.13 to 1.40)	0.47 (0.12 to 1.78)	0.49 (0.15 to 1.61)	0.64 (0.16 to 2.57)	0.64 (0.19 to 2.18)	0.65 (0.19 to 2.26)	0.74 (0.13 to 4.26)	0.77 (0.23 to 2.56)	0.82 (0.04 to 15.96)	0.82 (0.04 to 15.96)	**Amisulpride**	NA	NA	NA	NA
0.15 (0.00 to 4.92)	0.16 (0.02 to 1.41)	**0.32 (0.11 to 0.97)**	**0.33 (0.11 to 0.98)**	0.36 (0.10 to 1.25)	0.37 (0.13 to 1.12)	0.49 (0.13 to 1.82)	0.49 (0.16 to 1.50)	0.50 (0.16 to 1.51)	0.57 (0.10 to 3.08)	0.59 (0.20 to 1.73)	0.63 (0.03 to 11.69)	0.63 (0.03 to 11.69)	0.77 (0.16 to 3.74)	**Fluphenazine**	NA	NA	NA
0.09 (0.00 to 4.32)	0.09 (0.01 to 1.52)	0.19 (0.02 to 1.53)	0.19 (0.02 to 1.55)	0.21 (0.02 to 1.81)	0.21 (0.03 to 1.78)	0.28 (0.03 to 2.62)	0.28 (0.03 to 2.28)	0.28 (0.03 to 2.37)	0.33 (0.03 to 3.83)	0.34 (0.04 to 2.75)	0.36 (0.06 to 2.09)	0.36 (0.06 to 2.09)	0.44 (0.04 to 4.83)	0.57 (0.06 to 5.95)	**Placebo**	1.00 (0.09 to 11.09)	0.57 (0.14 to 2.43)
0.07 (0.00 to 5.07)	0.08 (0.00 to 2.01)	0.15 (0.01 to 2.31)	0.16 (0.01 to 2.34)	0.17 (0.01 to 2.69)	0.18 (0.01 to 2.68)	0.23 (0.01 to 3.84)	0.23 (0.02 to 3.43)	0.23 (0.02 to 3.56)	0.27 (0.01 to 5.38)	0.28 (0.02 to 4.18)	0.29 (0.02 to 4.76)	0.29 (0.02 to 4.76)	0.36 (0.02 to 6.87)	0.47 (0.03 to 8.56)	0.82 (0.09 to 7.08)	**Thioridazine**	1.00 (0.09 to 11.09)
0.05 (0.00 to 2.97)	0.06 (0.00 to 1.10)	0.11 (0.01 to 1.17)	0.11 (0.01 to 1.19)	0.12 (0.01 to 1.38)	0.13 (0.01 to 1.36)	0.17 (0.01 to 1.98)	0.17 (0.02 to 1.75)	0.17 (0.02 to 1.82)	0.19 (0.01 to 2.85)	0.20 (0.02 to 2.12)	0.21 (0.02 to 2.06)	0.21 (0.02 to 2.06)	0.26 (0.02 to 3.61)	0.34 (0.03 to 4.47)	0.60 (0.14 to 2.48)	0.73 (0.08 to 6.55)	**Trifluoperazine**

Treatments are presented in order of efficacy ranking. Results of the network meta-analysis are reported in the left lower half and results of pairwise meta-analyses in the right upper half. Each cell provides the effect estimate and the corresponding 95% credible interval (95% CI) of a comparison (left lower half: treatment in column versus treatment in row; right upper half: treatment in row versus treatment in column). The type of effect size measure is odd ratio (OR). Bold results indicate 95% CI excluding no effect. NA=not available.

League table for the outcome: Discontinuation due to any reason (RR)

**Sulpiride**	NA	NA	NA	NA	NA	NA	0.43 (0.02 to 2.23)	NA	NA	NA	NA	NA	NA	NA	NA	NA	NA
0.97 (0.02 to 5.43)	**Levomepromazine**	NA	NA	NA	NA	NA	0.44 (0.08 to 1.5)	NA	NA	NA	NA	NA	NA	NA	NA	NA	NA
0.56 (0.03 to 2.94)	0.58 (0.1 to 2.01)	**Olanzapine**	0.93 (0.7 to 1.2)	NA	0.91 (0.66 to 1.21)	NA	0.93 (0.42 to 1.63)	0.86 (0.35 to 1.58)	NA	**0.64 (0.44 to 0.91)**	NA	NA	0.58 (0.23 to 1.15)	NA	NA	NA	NA
0.56 (0.03 to 2.9)	0.57 (0.1 to 1.97)	0.99 (0.8 to 1.2)	**Clozapine**	1.03 (0.53 to 1.68)	0.89 (0.66 to 1.18)	NA	0.83 (0.54 to 1.2)	**0.23 (0.05 to 0.91)**	0.7 (0.24 to 1.45)	**0.65 (0.46 to 0.89)**	NA	NA	NA	NA	NA	NA	NA
0.52 (0.03 to 2.75)	0.54 (0.08 to 1.91)	0.93 (0.55 to 1.44)	0.94 (0.58 to 1.42)	**Ziprasidone**	NA	NA	0.89 (0.46 to 1.46)	NA	NA	NA	NA	NA	NA	NA	NA	NA	NA
0.5 (0.03 to 2.67)	0.52 (0.09 to 1.83)	0.91 (0.7 to 1.13)	0.91 (0.72 to 1.14)	0.97 (0.56 to 1.5)	**Risperidone**	0.84 (0.48 to 1.3)	1.34 (0.19 to 2.49)	0.61 (0.22 to 1.32)	NA	0.93 (0.56 to 1.38)	NA	NA	NA	0.36 (0.06 to 1.05)	NA	NA	NA
0.43 (0.02 to 2.27)	0.44 (0.06 to 1.62)	0.76 (0.41 to 1.26)	0.77 (0.42 to 1.26)	0.82 (0.37 to 1.47)	0.84 (0.48 to 1.3)	**Sertindole**	NA	NA	NA	NA	NA	NA	NA	NA	NA	NA	NA
0.43 (0.02 to 2.23)	0.44 (0.08 to 1.5)	0.76 (0.54 to 1.02)	0.77 (0.57 to 1.01)	0.82 (0.5 to 1.22)	0.84 (0.59 to 1.14)	1 (0.54 to 1.58)	**Chlorpromazine**	1.1 (0.78 to 1.45)	NA	1.62 (0.35 to 2.29)	NA	NA	NA	NA	0.6 (0.03 to 1.39)	0.63 (0.04 to 1.33)	0.69 (0.04 to 1.24)
0.42 (0.02 to 2.21)	0.43 (0.08 to 1.51)	0.75 (0.52 to 1.05)	0.77 (0.53 to 1.05)	0.81 (0.46 to 1.27)	0.83 (0.57 to 1.16)	0.99 (0.52 to 1.57)	0.99 (0.73 to 1.27)	**Quetiapine**	NA	1.11 (0.69 to 1.56)	NA	NA	NA	0.51 (0.12 to 1.24)	NA	NA	NA
0.39 (0.02 to 2.11)	0.4 (0.05 to 1.62)	0.69 (0.23 to 1.46)	0.7 (0.24 to 1.45)	0.74 (0.23 to 1.59)	0.77 (0.26 to 1.55)	0.91 (0.28 to 1.78)	0.91 (0.32 to 1.7)	0.93 (0.32 to 1.72)	**Zotepine**	NA	NA	NA	NA	NA	NA	NA	NA
0.38 (0.02 to 1.99)	0.39 (0.07 to 1.36)	**0.68 (0.51 to 0.86)**	**0.69 (0.54 to 0.86)**	0.73 (0.43 to 1.12)	**0.75 (0.56 to 0.96)**	0.89 (0.49 to 1.38)	0.89 (0.64 to 1.16)	0.9 (0.64 to 1.18)	0.98 (0.37 to 1.73)	**Haloperidol**	NA	NA	NA	1 (0.4 to 1.53)	0.51 (0.06 to 1.31)	NA	NA
0.37 (0 to 2.15)	0.38 (0.02 to 1.95)	0.66 (0.05 to 1.99)	0.67 (0.05 to 1.99)	0.7 (0.05 to 2.03)	0.73 (0.07 to 2.03)	0.86 (0.08 to 2.1)	0.86 (0.08 to 2.08)	0.87 (0.08 to 2.09)	0.95 (0.07 to 2.16)	0.97 (0.1 to 2.12)	**Fluphenazine_LAI**	1 (0.42 to 1.67)	NA	NA	0.64 (0.17 to 1.2)	NA	NA
0.37 (0 to 2.15)	0.38 (0.02 to 1.95)	0.66 (0.05 to 1.99)	0.67 (0.05 to 1.99)	0.7 (0.05 to 2.03)	0.73 (0.07 to 2.03)	0.86 (0.08 to 2.1)	0.86 (0.08 to 2.08)	0.87 (0.08 to 2.09)	0.95 (0.07 to 2.16)	0.97 (0.1 to 2.12)	1 (0.42 to 1.67)	**Pipotiazine_LAI**	NA	NA	0.64 (0.17 to 1.2)	NA	NA
0.33 (0.02 to 1.78)	0.34 (0.04 to 1.33)	0.58 (0.23 to 1.15)	0.59 (0.23 to 1.17)	0.63 (0.21 to 1.29)	0.65 (0.26 to 1.24)	0.78 (0.27 to 1.46)	0.78 (0.31 to 1.39)	0.78 (0.31 to 1.4)	0.85 (0.23 to 1.65)	0.87 (0.37 to 1.46)	0.9 (0.07 to 1.93)	0.9 (0.07 to 1.93)	**Amisulpride**	NA	NA	NA	NA
0.28 (0 to 1.56)	0.3 (0.04 to 1.15)	**0.51 (0.22 to 0.99)**	**0.52 (0.22 to 0.99)**	0.56 (0.2 to 1.1)	0.57 (0.25 to 1.05)	0.68 (0.25 to 1.25)	0.68 (0.3 to 1.18)	0.69 (0.3 to 1.18)	0.75 (0.2 to 1.43)	0.76 (0.36 to 1.23)	0.79 (0.06 to 1.69)	0.79 (0.06 to 1.69)	0.88 (0.3 to 1.49)	**Fluphenazine**	NA	NA	NA
0.24 (0 to 1.32)	0.24 (0.03 to 1.12)	0.42 (0.06 to 1.12)	0.42 (0.06 to 1.13)	0.45 (0.06 to 1.17)	0.45 (0.09 to 1.16)	0.55 (0.09 to 1.25)	0.55 (0.09 to 1.22)	0.55 (0.09 to 1.23)	0.61 (0.09 to 1.31)	0.62 (0.12 to 1.25)	0.64 (0.17 to 1.2)	0.64 (0.17 to 1.2)	0.71 (0.12 to 1.34)	0.81 (0.17 to 1.36)	**Placebo**	1 (0.26 to 1.34)	0.86 (0.43 to 1.15)
0.21 (0 to 1.29)	0.24 (0 to 1.16)	0.39 (0.04 to 1.19)	0.41 (0.04 to 1.19)	0.42 (0.04 to 1.21)	0.44 (0.04 to 1.21)	0.52 (0.04 to 1.26)	0.52 (0.07 to 1.25)	0.52 (0.07 to 1.25)	0.57 (0.04 to 1.29)	0.58 (0.07 to 1.27)	0.59 (0.07 to 1.28)	0.59 (0.07 to 1.28)	0.67 (0.07 to 1.31)	0.76 (0.1 to 1.33)	0.94 (0.26 to 1.31)	**Thioridazine**	1 (0.31 to 1.24)
0.2 (0 to 1.17)	0.23 (0 to 1.02)	0.36 (0.04 to 1.03)	0.36 (0.04 to 1.04)	0.39 (0.04 to 1.06)	0.41 (0.04 to 1.06)	0.49 (0.04 to 1.12)	0.49 (0.09 to 1.1)	0.49 (0.09 to 1.11)	0.52 (0.04 to 1.16)	0.54 (0.09 to 1.13)	0.55 (0.09 to 1.13)	0.55 (0.09 to 1.13)	0.62 (0.09 to 1.19)	0.7 (0.13 to 1.2)	0.87 (0.43 to 1.15)	0.93 (0.29 to 1.22)	**Trifluoperazine**

The original results given in OR (and their 95% CI) are transformed to RR (left lower half: treatment in column versus treatment in row; right upper half: treatment in row versus treatment in column) using the formula described above. For this transformation, we assumed a discontinuation rate (due to any reason) with clozapine of 35% as the control event rate (CER) for all comparisons of active antipsychotic versus clozapine.
Treatments are presented in order of efficacy ranking. Results of the network meta-analysis are reported in the left lower half and results of pairwise meta-analyses in the right upper half. Bold results indicate 95% CI excluding no effect. NA=not available.

Forest plot of results of pairwise meta-analyses

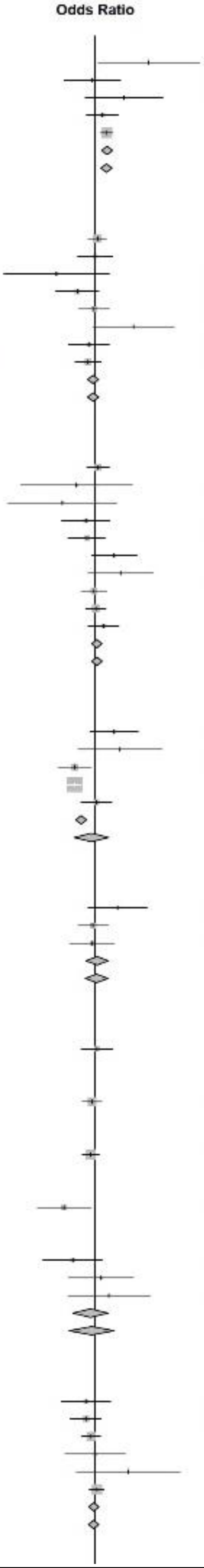

Study	Experimental		Control		OR	95%-CI	Weight	Weight
	Events	Total	Events	Total			(common)	(random)
comparison = Haloperidol_Olanzapine								
Altamura 2002 Haloperidol Olanzapine	7	15	0	13	23.82	[1.20; 473.07]	0.0%	0.2%
Buchanan 2005 Haloperidol Olanzapine	3	34	3	29	0.84	[0.16; 4.51]	0.4%	0.7%
Smith 2001 Haloperidol Olanzapine	4	18	1	20	5.43	[0.55; 54.01]	0.1%	0.4%
Volavka 2002 Haloperidol Olanzapine	16	37	13	39	1.52	[0.60; 3.86]	1.0%	1.7%
Breier 1999a Haloperidol Olanzapine	90	174	124	352	1.97	[1.36; 2.85]	5.4%	3.7%
Common effect model		278		453	2.01	[1.45; 2.79]	7.0%	--
Random effects model					1.94	[1.40; 2.70]	--	6.8%
Heterogeneity: $I^2 = 15\%$, $\tau^2 = < 0.0001$, $p = 0.32$								
comparison = Clozapine_Risperidone								
Azorin 2001 Clozapine Risperidone	38	138	34	135	1.13	[0.66; 1.94]	3.4%	3.0%
Bondolfi 1998 Clozapine Risperidone	9	43	9	43	1.00	[0.35; 2.83]	1.0%	1.5%
Daniel 1996 Clozapine Risperidone	0	10	3	10	0.10	[0.00; 2.28]	0.5%	0.2%
McEvoy 2006 Clozapine Risperidone	25	49	12	16	0.35	[0.10; 1.23]	1.2%	1.1%
Volavka 2002 Clozapine Risperidone	18	40	19	41	0.95	[0.40; 2.27]	1.4%	1.9%
Wahlbeck 2000 Clozapine Risperidone	6	11	1	9	9.60	[0.88; 105.17]	0.1%	0.4%
Chowdhury 1999 Clozapine Risperidone	6	30	8	30	0.69	[0.21; 2.30]	0.9%	1.2%
Schooler 2016 Clozapine Risperidone	25	53	31	54	0.66	[0.31; 1.42]	2.2%	2.2%
Common effect model		374		338	0.88	[0.64; 1.22]	10.6%	--
Random effects model					0.88	[0.63; 1.23]	--	11.5%
Heterogeneity: $I^2 = 25\%$, $\tau^2 = < 0.0001$, $p = 0.23$								
comparison = Clozapine_Olanzapine								
Bitter 2004 Clozapine Olanzapine	32	72	30	75	1.20	[0.62; 2.31]	2.2%	2.5%
Shaw 2006 Clozapine Olanzapine	0	12	1	13	0.33	[0.01; 8.99]	0.2%	0.2%
Conley 2003 Clozapine Olanzapine	0	5	3	8	0.14	[0.01; 3.47]	0.4%	0.2%
Kumra 2007 Clozapine Olanzapine	4	18	7	21	0.57	[0.14; 2.40]	0.7%	0.9%
McEvoy 2006 Clozapine Olanzapine	25	49	12	19	0.61	[0.20; 1.80]	1.2%	1.4%
Meltzer 2008 Clozapine Olanzapine	11	21	5	19	3.08	[0.81; 11.68]	0.3%	1.0%
Moresco 2004 Clozapine Olanzapine	6	12	2	11	4.50	[0.67; 30.23]	0.1%	0.6%
Naber 2005 Clozapine Olanzapine	35	57	36	57	0.93	[0.44; 1.98]	1.9%	2.2%
Tollefson 2001 Clozapine Olanzapine	37	90	36	90	1.05	[0.58; 1.90]	2.9%	2.8%
Volavka 2002 Clozapine Olanzapine	18	40	13	39	1.64	[0.66; 4.07]	1.0%	1.8%
Common effect model		376		352	1.10	[0.81; 1.49]	10.9%	--
Random effects model					1.11	[0.81; 1.51]	--	13.6%
Heterogeneity: $I^2 = 5\%$, $\tau^2 = < 0.0001$, $p = 0.40$								
comparison = Clozapine_Haloperidol								
Buchanan 1998 Clozapine Haloperidol	8	38	3	37	3.02	[0.73; 12.44]	0.3%	0.9%
Kumra 1996 Clozapine Haloperidol	3	10	1	11	4.29	[0.37; 50.20]	0.1%	0.4%
Kane 2001 Clozapine Haloperidol	13	37	22	34	0.30	[0.11; 0.78]	2.0%	1.6%
Rosenheck 1997 Clozapine Haloperidol	88	205	157	218	0.29	[0.19; 0.44]	11.9%	3.6%
Volavka 2002 Clozapine Haloperidol	18	40	16	37	1.07	[0.44; 2.64]	1.3%	1.8%
Common effect model		330		337	0.44	[0.32; 0.60]	15.6%	--
Random effects model					0.80	[0.29; 2.22]	--	8.3%
Heterogeneity: $I^2 = 79\%$, $\tau^2 = 0.9824$, $p < 0.01$								
comparison = Haloperidol_Risperidone								
Claus 1992 Haloperidol Risperidone	6	22	2	22	3.75	[0.66; 21.15]	0.2%	0.7%
Volavka 2002 Haloperidol Risperidone	16	37	19	41	0.88	[0.36; 2.16]	1.4%	1.8%
Wirshing 1999 Haloperidol Risperidone	5	33	6	34	0.83	[0.23; 3.05]	0.7%	1.1%
Common effect model		92		97	1.12	[0.58; 2.16]	2.3%	--
Random effects model					1.08	[0.55; 2.14]	--	3.6%
Heterogeneity: $I^2 = 14\%$, $\tau^2 = < 0.0001$, $p = 0.31$								
comparison = Chlorpromazine_Olanzapine								
Conley 1998 Chlorpromazine Olanzapine	13	42	12	42	1.12	[0.44; 2.86]	1.1%	1.7%
comparison = Haloperidol_Quetiapine								
Emsley 2000 Haloperidol Quetiapine	28	145	32	143	0.83	[0.47; 1.47]	3.6%	2.9%
comparison = Risperidone_Sertindole								
Kane 2010b Risperidone Sertindole	30	105	74	216	0.77	[0.46; 1.28]	4.7%	3.1%
comparison = Clozapine_Quetiapine								
McEvoy 2006 Clozapine Quetiapine	25	49	13	15	0.16	[0.03; 0.79]	1.3%	0.8%
comparison = Olanzapine_Quetiapine								
McEvoy 2006 Olanzapine Quetiapine	12	19	13	15	0.26	[0.05; 1.53]	0.7%	0.7%
Sirota 2006 Olanzapine Quetiapine	3	21	2	19	1.42	[0.21; 9.55]	0.2%	0.6%
Suzuki 2007 Olanzapine Quetiapine	3	15	1	10	2.25	[0.20; 25.37]	0.1%	0.4%
Common effect model		55		44	0.75	[0.27; 2.14]	1.1%	--
Random effects model					0.81	[0.21; 3.08]	--	1.6%
Heterogeneity: $I^2 = 22\%$, $\tau^2 = 0.3581$, $p = 0.28$								
comparison = Olanzapine_Risperidone								
McEvoy 2006 Olanzapine Risperidone	12	19	12	16	0.57	[0.13; 2.48]	0.7%	0.9%
Volavka 2002 Olanzapine Risperidone	13	39	19	41	0.58	[0.23; 1.43]	1.7%	1.8%
Alvarez 2006 Olanzapine Risperidone	33	124	39	123	0.78	[0.45; 1.35]	3.9%	3.0%
Chen 2012 Olanzapine Risperidone	3	16	3	16	1.00	[0.17; 5.90]	0.3%	0.6%
Suzuki 2007 Olanzapine Risperidone	3	15	0	12	7.00	[0.33; 150.06]	0.1%	0.2%
Kinon 2009 Olanzapine Risperidone	60	186	58	192	1.10	[0.71; 1.70]	5.3%	3.4%
Common effect model		399		400	0.92	[0.68; 1.24]	12.0%	--
Random effects model					0.91	[0.67; 1.23]	--	10.0%
Heterogeneity: $I^2 = 0\%$, $\tau^2 = < 0.0001$, $p = 0.54$								

comparison = Quetiapine_Risperidone								
McEvoy 2006 Quetiapine Risperidone	13	15	12	16	2.17	[0.33; 14.06]	0.2%	0.6%
Conley 2005 Quetiapine Risperidone	5	12	4	13	1.61	[0.31; 8.32]	0.3%	0.7%
Suzuki 2007 Quetiapine Risperidone	1	10	0	12	3.95	[0.14; 108.09]	0.1%	0.2%
Common effect model		37		41	2.04	[0.65; 6.41]	0.6%	--
Random effects model					2.01	[0.63; 6.40]	--	1.5%
Heterogeneity: $I^2 = 0\%$, $\tau^2 = 0$, $p = 0.89$								
comparison = Chlorpromazine_Risperidone								
Mercer 1997 Chlorpromazine Risperidone	1	12	2	15	0.59	[0.05; 7.43]	0.2%	0.3%
comparison = Chlorpromazine_Placebo								
Schiele 1961, 06602 Chlorpromazine Placebo	0	20	1	20	0.32	[0.01; 8.26]	0.2%	0.2%
comparison = Chlorpromazine_Thioridazine								
Schiele 1961, 06602 Chlorpromazine Thioridazine	0	20	1	20	0.32	[0.01; 8.26]	0.2%	0.2%
comparison = Chlorpromazine_Trifluoperazine								
Schiele 1961, 06602 Chlorpromazine Trifluoperazine	0	20	1	20	0.32	[0.01; 8.26]	0.2%	0.2%
comparison = Placebo_Thioridazine								
Schiele 1961, 06602 Placebo Thioridazine	1	20	1	20	1.00	[0.06; 17.18]	0.1%	0.3%
comparison = Placebo_Trifluoperazine								
Schiele 1961, 06602 Placebo Trifluoperazine	1	20	1	20	1.00	[0.06; 17.18]	0.1%	0.3%
Marjerrison 1964 Placebo Trifluoperazine	3	34	3	16	0.42	[0.07; 2.36]	0.5%	0.7%
Common effect model		54		36	0.54	[0.12; 2.35]	0.6%	--
Random effects model					0.53	[0.12; 2.32]	--	0.9%
Heterogeneity: $I^2 = 0\%$, $\tau^2 = 0$, $p = 0.61$								
comparison = Thioridazine_Trifluoperazine								
Schiele 1961, 06602 Thioridazine Trifluoperazine	1	20	1	20	1.00	[0.06; 17.18]	0.1%	0.3%
comparison = Aripiprazole_Perphenazine								
Kane 2007a Aripiprazole Perphenazine	44	154	31	146	1.48	[0.87; 2.52]	3.1%	3.0%
comparison = Chlorpromazine_Haloperidol								
Teja 1975 Chlorpromazine Haloperidol	0	14	0	13	0.93	[0.02; 50.30]	0.1%	0.1%
McCreadie 1977 Chlorpromazine Haloperidol	2	10	0	10	6.18	[0.26; 146.78]	0.1%	0.2%
Common effect model		24		23	3.22	[0.32; 32.67]	0.1%	--
Random effects model					2.97	[0.25; 35.51]	--	0.4%
Heterogeneity: $I^2 = 0\%$, $\tau^2 = 0$, $p = 0.47$								
comparison = Fluphenazine_Quetiapine								
Conley 2005 Fluphenazine Quetiapine	9	13	5	12	3.15	[0.61; 16.31]	0.2%	0.7%
comparison = Fluphenazine_Risperidone								
Conley 2005 Fluphenazine Risperidone	9	13	4	13	5.06	[0.96; 26.78]	0.2%	0.7%
comparison = Chlorpromazine_Clozapine								
Hong 1997 Chlorpromazine Clozapine	2	19	2	21	1.12	[0.14; 8.82]	0.2%	0.5%
Honigfeld 1984b Chlorpromazine Clozapine	36	76	27	75	1.60	[0.83; 3.07]	2.0%	2.6%
Kane 1988 Chlorpromazine Clozapine	18	142	15	126	1.07	[0.52; 2.23]	1.9%	2.3%
Common effect model		237		222	1.33	[0.83; 2.13]	4.1%	--
Random effects model					1.33	[0.83; 2.13]	--	5.3%
Heterogeneity: $I^2 = 0\%$, $\tau^2 = 0$, $p = 0.72$								
comparison = Chlorpromazine_Ziprasidone								
Kane 2006 Chlorpromazine Ziprasidone	19	154	16	152	1.20	[0.59; 2.42]	1.9%	2.4%
comparison = Clozapine_Zotepine								
Meyer−Lindenberg 1997 Clozapine Zotepine	7	25	10	25	0.58	[0.18; 1.91]	1.0%	1.2%
comparison = Clozapine_Ziprasidone								
Sacchetti 2009 Clozapine Ziprasidone	29	74	28	73	1.04	[0.53; 2.01]	2.3%	2.5%
comparison = Chlorpromazine_Levomepromazine								
Lal 2006 Chlorpromazine Levomepromazine	5	19	2	19	3.04	[0.51; 18.11]	0.2%	0.6%
comparison = Fluphenazine_Haloperidol								
Hall 1968 Fluphenazine Haloperidol	5	25	5	25	1.00	[0.25; 4.00]	0.5%	1.0%
comparison = Chlorpromazine_Sulpiride								
Toru 1972 Chlorpromazine Sulpiride	1	37	0	38	3.16	[0.12; 80.19]	0.1%	0.2%
comparison = Amisulpride_Olanzapine								
Kahn 2018 Amisulpride Olanzapine	14	47	7	46	2.36	[0.85; 6.55]	0.7%	1.5%
comparison = Chlorpromazine_Quetiapine								
AstraZeneca 5077IL/0031 Chlorpromazine Quetiapine	41	130	41	130	1.00	[0.59; 1.69]	3.8%	3.1%
AstraZeneca 5077IL/0054 Chlorpromazine Quetiapine	40	119	31	117	1.40	[0.80; 2.46]	2.8%	2.9%
Common effect model		249		247	1.17	[0.80; 1.72]	6.7%	--
Random effects model					1.17	[0.80; 1.72]	--	6.0%
Heterogeneity: $I^2 = 0\%$, $\tau^2 = 0$, $p = 0.38$								
comparison = Haloperidol_Placebo								
Browne 1988 Haloperidol Placebo	3	6	4	5	0.25	[0.02; 3.77]	0.3%	0.3%
comparison = Paliperidone_Paliperidone_LAI								
Actrn12618001113246 Paliperidone Paliperidone_LAI	4	36	3	36	1.38	[0.28; 6.64]	0.4%	0.8%
comparison = Fluphenazine_LAI_Pipotiazine_LAI								
Schlosberg 1978 Fluphenazine_LAI Pipotiazine_LAI	21	30	21	30	1.00	[0.33; 3.02]	0.9%	1.4%
comparison = Fluphenazine_LAI_Placebo								
Schlosberg 1978 Fluphenazine_LAI Placebo	21	30	13	15	0.36	[0.07; 1.93]	0.7%	0.7%

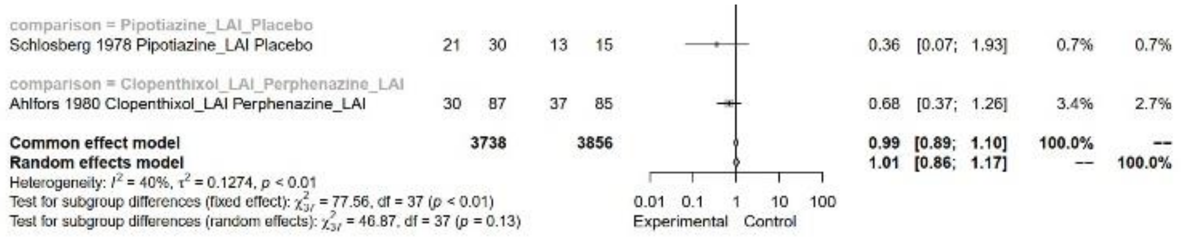

A summary effect size is calculated by pairwise meta-analyses of all studies of a specific comparison. The type of effect size measure is OR.

8.5 Discontinuation due to inefficacy

We transformed the original ORs to RRs and EERs vs. CER assuming the baseline discontinuation rate due to inefficacy with clozapine of 6% (namely CER). 6% was the average discontinuation rate due to efficacy with clozapine across all clozapine -arms in the network meta-analysis, as estimated by a single-arm meta-analysis of proportions.

Network plot

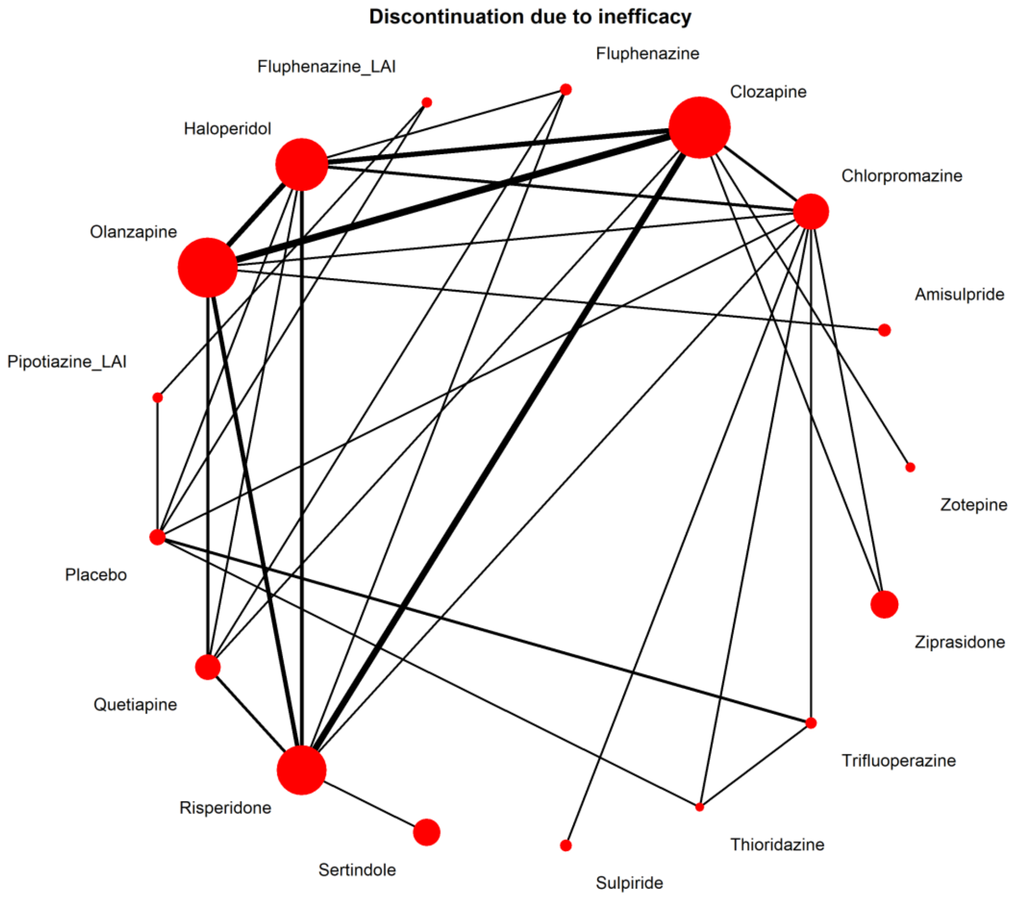

Lines link treatments with direct comparisons in trials; thickness of lines corresponds to the number of trials evaluating the comparison; size of the nodes corresponds to the number of participants assigned to the treatment.

Forest-plot of results of network meta-analysis for antipsychotic drugs versus clozapine

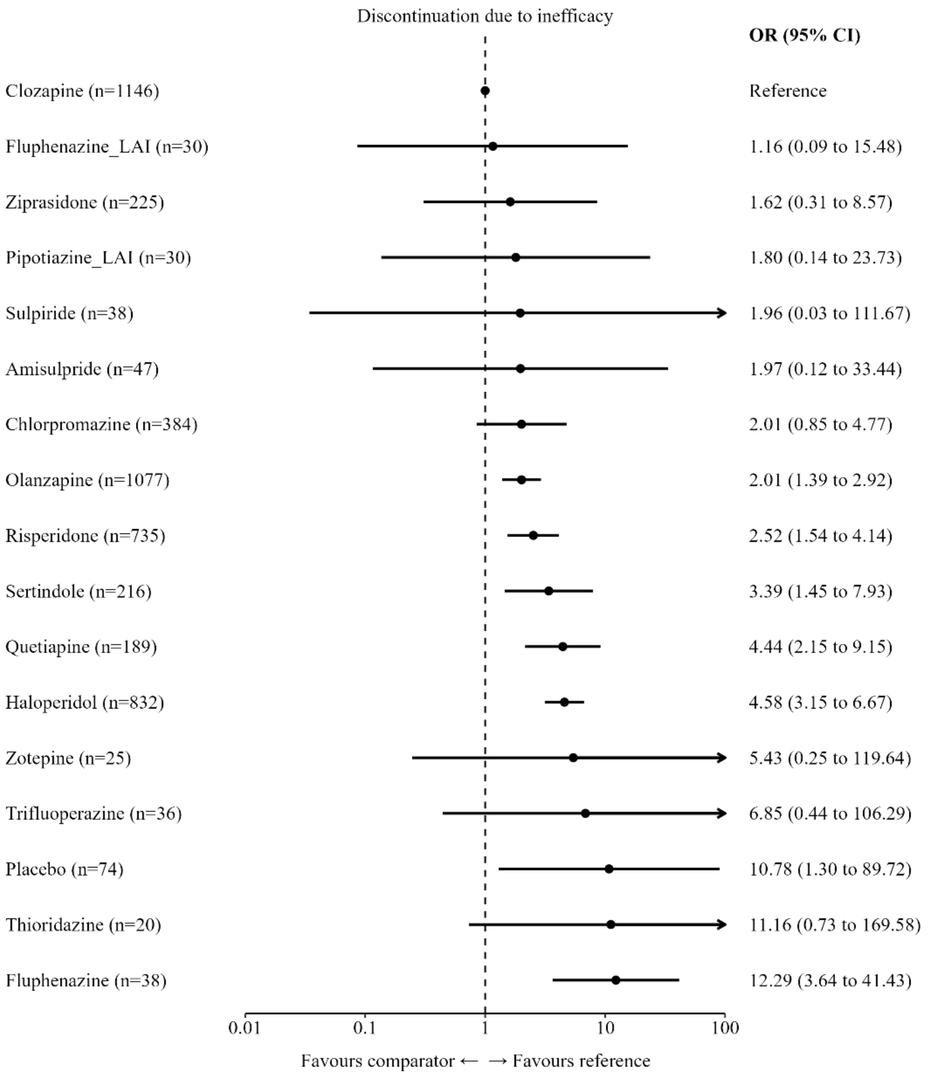

Effect sizes, measured as odds ratio (OR), are from the network meta-analysis. Order of treatments is according to the mean effect size. Reference is clozapine. The direction of the effect is indicated below the x-axis.

Forest plot with RR and EER versus CER

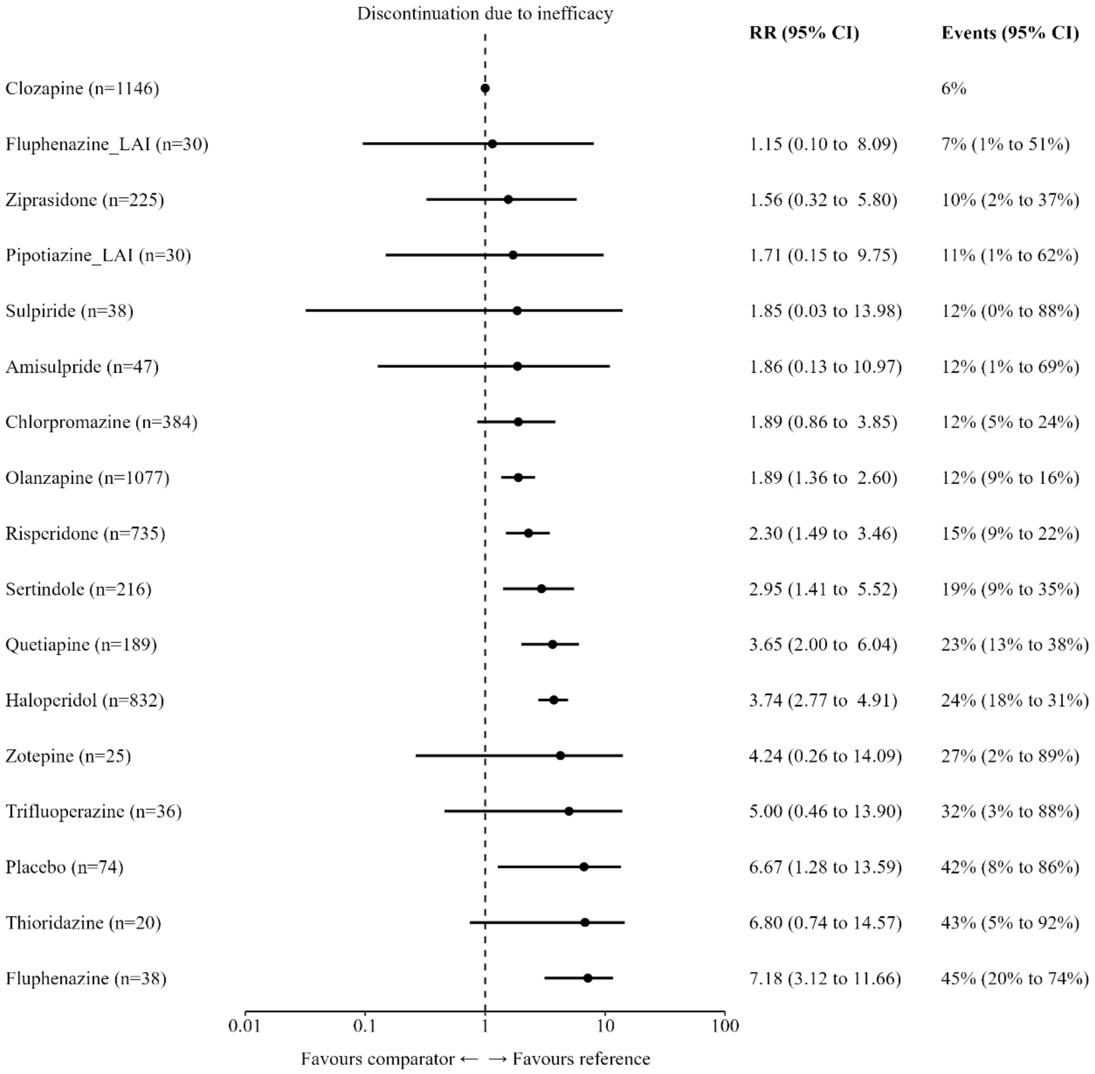

For this forest plot, we transformed the original OR to RR and exposure events rates (EER, called "events" in the forest plot) using the formula indicated above. Therefore, we used an average discontinuation rate (due to inefficacy) with clozapine of 6% as the control event rate (CER).
Effect sizes are from the network meta-analysis. Order of treatments is according to the mean effect size. Reference is clozapine. The direction of the effect is indicated below the x-axis.

League table for the outcome: Discontinuation due to inefficacy (OR)

**Clozapine**	NA	0.65 (0.10 to 4.04)	NA	NA	NA	0.40 (0.12 to 1.29)	0.77 (0.47 to 1.25)	**0.31 (0.17 to 0.57)**	NA	**0.17 (0.04 to 0.69)**	**0.16 (0.10 to 0.27)**	0.18 (0.01 to 4.06)	NA	NA	NA	NA
0.87 (0.06 to 11.59)	**Fluphenazine_LAI**	NA	0.64 (0.22 to 1.90)	NA	NA	NA	NA	NA	NA	NA	NA	NA	NA	**0.11 (0.02 to 0.48)**	NA	NA
0.62 (0.12 to 3.26)	0.71 (0.03 to 15.26)	**Ziprasidone**	NA	NA	NA	1.01 (0.02 to 51.61)	NA	NA	NA	NA	NA	NA	NA	NA	NA	NA
0.56 (0.04 to 7.34)	0.64 (0.22 to 1.90)	0.90 (0.04 to 19.07)	**Pipotiazine_LAI**	NA	NA	NA	NA	NA	NA	NA	NA	NA	NA	**0.17 (0.04 to 0.73)**	NA	NA
0.51 (0.01 to 29.12)	0.59 (0.01 to 67.10)	0.83 (0.01 to 63.66)	0.92 (0.01 to 103.54)	**Sulpiride**	NA	0.97 (0.02 to 50.59)	NA	NA	NA	NA	NA	NA	NA	NA	NA	NA
0.51 (0.03 to 8.64)	0.59 (0.01 to 26.88)	0.82 (0.03 to 21.97)	0.91 (0.02 to 41.40)	1.00 (0.01 to 137.34)	**Amisulpride**	NA	0.98 (0.06 to 16.22)	NA	NA	NA	NA	NA	NA	NA	NA	NA
0.50 (0.21 to 1.18)	0.57 (0.04 to 7.80)	0.81 (0.13 to 4.90)	0.89 (0.07 to 11.96)	0.97 (0.02 to 50.59)	0.98 (0.05 to 18.62)	**Chlorpromazine**	0.37 (0.07 to 2.05)	1.24 (0.02 to 67.33)	NA	NA	0.96 (0.06 to 16.38)	NA	1.00 (0.02 to 53.08)	0.32 (0.01 to 8.30)	0.32 (0.01 to 8.30)	NA
**0.50 (0.34 to 0.72)**	0.57 (0.04 to 7.70)	0.81 (0.15 to 4.42)	0.89 (0.07 to 11.80)	0.97 (0.02 to 55.81)	0.98 (0.06 to 16.22)	1.00 (0.41 to 2.43)	**Olanzapine**	1.19 (0.51 to 2.76)	NA	0.73 (0.20 to 2.61)	**0.53 (0.35 to 0.78)**	NA	NA	NA	NA	NA
**0.40 (0.24 to 0.65)**	0.46 (0.03 to 6.32)	0.64 (0.11 to 3.64)	0.71 (0.05 to 9.68)	0.78 (0.01 to 45.24)	0.78 (0.04 to 13.57)	0.80 (0.30 to 2.09)	0.80 (0.47 to 1.35)	**Risperidone**	0.74 (0.37 to 1.48)	0.90 (0.29 to 2.83)	**0.33 (0.12 to 0.90)**	NA	NA	NA	NA	0.19 (0.03 to 1.04)
**0.29 (0.13 to 0.69)**	0.34 (0.02 to 5.14)	0.48 (0.07 to 3.09)	0.53 (0.04 to 7.88)	0.58 (0.01 to 35.69)	0.58 (0.03 to 10.97)	0.59 (0.18 to 1.94)	0.59 (0.25 to 1.41)	0.74 (0.37 to 1.48)	**Sertindole**	NA	NA	NA	NA	NA	NA	NA
**0.23 (0.11 to 0.46)**	0.26 (0.02 to 3.75)	0.37 (0.06 to 2.24)	0.41 (0.03 to 5.75)	0.44 (0.01 to 26.61)	0.44 (0.02 to 8.04)	0.45 (0.15 to 1.35)	**0.45 (0.22 to 0.93)**	0.57 (0.26 to 1.23)	0.76 (0.27 to 2.15)	**Quetiapine**	1.43 (0.55 to 3.73)	NA	NA	NA	NA	0.21 (0.04 to 1.17)
**0.22 (0.15 to 0.32)**	0.25 (0.02 to 3.34)	0.35 (0.06 to 1.94)	0.39 (0.03 to 5.12)	0.43 (0.01 to 24.55)	0.43 (0.03 to 7.27)	0.44 (0.18 to 1.08)	**0.44 (0.31 to 0.61)**	**0.55 (0.33 to 0.93)**	0.74 (0.31 to 1.76)	0.97 (0.49 to 1.92)	**Haloperidol**	NA	NA	0.25 (0.02 to 3.79)	NA	0.64 (0.10 to 4.23)
0.18 (0.01 to 4.06)	0.21 (0.00 to 12.07)	0.30 (0.01 to 10.02)	0.33 (0.01 to 18.60)	0.36 (0.00 to 58.66)	0.36 (0.01 to 24.05)	0.37 (0.01 to 9.20)	0.37 (0.02 to 8.36)	0.47 (0.02 to 10.67)	0.62 (0.03 to 15.45)	0.82 (0.03 to 19.60)	0.84 (0.04 to 19.04)	**Zotepine**	NA	NA	NA	NA
0.15 (0.01 to 2.26)	0.17 (0.01 to 1.99)	0.24 (0.01 to 5.72)	0.26 (0.02 to 3.05)	0.29 (0.00 to 34.95)	0.29 (0.01 to 14.54)	0.29 (0.02 to 4.54)	0.29 (0.02 to 4.55)	0.37 (0.02 to 5.87)	0.49 (0.03 to 8.59)	0.65 (0.04 to 10.76)	0.67 (0.04 to 10.27)	0.79 (0.01 to 49.37)	**Trifluoperazine**	0.68 (0.09 to 4.94)	0.32 (0.01 to 8.30)	NA
**0.09 (0.01 to 0.77)**	**0.11 (0.02 to 0.48)**	0.15 (0.01 to 2.18)	**0.17 (0.04 to 0.73)**	0.18 (0.00 to 16.18)	0.18 (0.01 to 6.15)	0.19 (0.02 to 1.58)	0.19 (0.02 to 1.55)	0.23 (0.03 to 2.02)	0.31 (0.03 to 3.02)	0.41 (0.05 to 3.74)	0.42 (0.05 to 3.49)	0.50 (0.01 to 21.38)	0.64 (0.09 to 4.53)	**Placebo**	1.00 (0.09 to 10.55)	NA
0.09 (0.01 to 1.36)	0.10 (0.01 to 1.47)	0.15 (0.01 to 3.45)	0.16 (0.01 to 2.26)	0.18 (0.00 to 21.00)	0.18 (0.00 to 8.82)	0.18 (0.01 to 2.68)	0.18 (0.01 to 2.75)	0.23 (0.01 to 3.54)	0.30 (0.02 to 5.18)	0.40 (0.02 to 6.49)	0.41 (0.03 to 6.20)	0.49 (0.01 to 29.94)	0.61 (0.04 to 8.46)	0.97 (0.11 to 8.65)	**Thioridazine**	NA
**0.08 (0.02 to 0.27)**	0.09 (0.01 to 1.61)	0.13 (0.02 to 1.03)	0.15 (0.01 to 2.47)	0.16 (0.00 to 10.77)	0.16 (0.01 to 3.41)	**0.16 (0.04 to 0.71)**	**0.16 (0.05 to 0.55)**	**0.21 (0.06 to 0.69)**	0.28 (0.07 to 1.11)	0.36 (0.10 to 1.25)	0.37 (0.11 to 1.23)	0.44 (0.02 to 12.26)	0.56 (0.03 to 10.93)	0.88 (0.08 to 9.79)	0.91 (0.05 to 17.50)	**Fluphenazine**

Treatments are presented in order of efficacy ranking. Results of the network meta-analysis are reported in the left lower half and results of pairwise meta-analyses in the right upper half. Each cell provides the effect estimate and the corresponding 95% credible interval (95% CI) of a comparison (left lower half: treatment in column versus treatment in row; right upper half: treatment in row versus treatment in column). The type of effect size measure is odd ratio (OR). Bold results indicate 95% CI excluding no effect. NA=not available.

League table for the outcome: Discontinuation due to inefficacy (RR)

**Clozapine**	NA	0.67 (0.11 to 3.1)	NA	NA	NA	0.43 (0.13 to 1.25)	0.79 (0.5 to 1.21)	**0.35 (0.19 to 0.61)**	NA	**0.21 (0.05 to 0.74)**	**0.2 (0.13 to 0.33)**	0.23 (0.01 to 2.2)	NA	NA	NA	NA
0.88 (0.06 to 6.51)	**Fluphenazine_LAI**	NA	0.67 (0.24 to 1.73)	NA	NA	NA	NA	NA	NA	NA	NA	NA	NA	**0.18 (0.03 to 0.62)**	NA	NA
0.64 (0.13 to 2.66)	0.73 (0.03 to 6.28)	**Ziprasidone**	NA	NA	NA	1.01 (0.02 to 7.15)	NA	NA	NA	NA	NA	NA	NA	NA	NA	NA
0.59 (0.04 to 4.32)	0.67 (0.24 to 1.73)	0.91 (0.04 to 6.36)	**Pipotiazine_LAI**	NA	NA	NA	NA	NA	NA	NA	NA	NA	NA	**0.26 (0.07 to 0.83)**	NA	NA
0.54 (0.01 to 6.64)	0.62 (0.01 to 7.49)	0.85 (0.01 to 7.45)	0.93 (0.01 to 7.75)	**Sulpiride**	NA	0.97 (0.02 to 7.13)	NA	NA	NA	NA	NA	NA	NA	NA	NA	NA
0.54 (0.03 to 4.5)	0.62 (0.01 to 6.53)	0.84 (0.03 to 6.23)	0.92 (0.02 to 7.06)	1 (0.01 to 7.88)	**Amisulpride**	NA	0.98 (0.07 to 5.65)	NA	NA	NA	NA	NA	NA	NA	NA	NA
0.53 (0.23 to 1.15)	0.6 (0.05 to 4.25)	0.83 (0.15 to 3.31)	0.9 (0.08 to 5.1)	0.97 (0.02 to 7.13)	0.98 (0.06 to 5.88)	**Chlorpromazine**	0.4 (0.08 to 1.82)	1.2 (0.02 to 6.18)	NA	NA	0.97 (0.08 to 3.47)	NA	1 (0.03 to 2.99)	0.45 (0.02 to 2)	0.46 (0.02 to 1.98)	NA
**0.53 (0.37 to 0.75)**	0.6 (0.05 to 4.22)	0.83 (0.17 to 3.11)	0.9 (0.08 to 5.07)	0.97 (0.02 to 7.21)	0.98 (0.07 to 5.65)	1 (0.44 to 2.07)	**Olanzapine**	1.16 (0.55 to 2.19)	NA	0.78 (0.25 to 1.89)	**0.6 (0.42 to 0.82)**	NA	NA	NA	NA	NA
**0.44 (0.27 to 0.69)**	0.5 (0.04 to 3.52)	0.68 (0.13 to 2.61)	0.74 (0.06 to 4.22)	0.81 (0.01 to 5.96)	0.81 (0.05 to 4.72)	0.82 (0.33 to 1.8)	0.82 (0.51 to 1.28)	**Risperidone**	0.78 (0.42 to 1.36)	0.92 (0.35 to 1.98)	**0.39 (0.15 to 0.92)**	NA	NA	NA	NA	0.3 (0.05 to 1.02)
**0.34 (0.16 to 0.73)**	0.39 (0.02 to 2.87)	0.53 (0.09 to 2.21)	0.58 (0.05 to 3.41)	0.63 (0.01 to 4.69)	0.63 (0.04 to 3.78)	0.64 (0.21 to 1.65)	0.64 (0.29 to 1.31)	0.78 (0.42 to 1.36)	**Sertindole**	NA	NA	NA	NA	NA	NA	NA
**0.28 (0.14 to 0.53)**	0.32 (0.03 to 2.27)	0.43 (0.08 to 1.73)	0.48 (0.04 to 2.71)	0.51 (0.01 to 3.78)	0.51 (0.03 to 3.02)	0.52 (0.19 to 1.25)	**0.52 (0.27 to 0.95)**	0.63 (0.32 to 1.17)	0.81 (0.33 to 1.69)	**Quetiapine**	1.3 (0.62 to 2.25)	NA	NA	NA	NA	0.33 (0.07 to 1.08)
**0.27 (0.19 to 0.38)**	0.31 (0.03 to 2.13)	0.42 (0.08 to 1.58)	0.46 (0.04 to 2.57)	0.5 (0.01 to 3.67)	0.5 (0.04 to 2.89)	0.51 (0.22 to 1.06)	**0.51 (0.37 to 0.67)**	**0.62 (0.39 to 0.95)**	0.79 (0.37 to 1.49)	0.98 (0.56 to 1.57)	**Haloperidol**	NA	NA	0.37 (0.03 to 1.72)	NA	0.77 (0.17 to 1.7)
0.23 (0.01 to 2.2)	0.27 (0 to 2.98)	0.37 (0.01 to 2.88)	0.4 (0.01 to 3.19)	0.44 (0 to 3.48)	0.44 (0.01 to 3.28)	0.45 (0.01 to 2.83)	0.45 (0.03 to 2.76)	0.55 (0.03 to 2.92)	0.69 (0.04 to 3.11)	0.86 (0.04 to 3.21)	0.88 (0.05 to 3.19)	**Zotepine**	NA	NA	NA	NA
0.21 (0.01 to 1.61)	0.23 (0.01 to 1.51)	0.32 (0.01 to 2.27)	0.34 (0.03 to 1.84)	0.38 (0 to 2.93)	0.38 (0.01 to 2.71)	0.38 (0.03 to 2.12)	0.38 (0.03 to 2.12)	0.46 (0.03 to 2.28)	0.59 (0.04 to 2.49)	0.73 (0.06 to 2.6)	0.75 (0.06 to 2.58)	0.85 (0.01 to 2.98)	**Trifluoperazine**	0.79 (0.15 to 1.83)	0.46 (0.02 to 1.98)	NA
**0.15 (0.02 to 0.85)**	**0.18 (0.03 to 0.62)**	0.24 (0.02 to 1.44)	**0.26 (0.07 to 0.83)**	0.28 (0 to 2.14)	0.28 (0.02 to 1.91)	0.29 (0.03 to 1.26)	0.29 (0.03 to 1.25)	0.34 (0.05 to 1.4)	0.44 (0.05 to 1.61)	0.55 (0.08 to 1.71)	0.56 (0.08 to 1.68)	0.64 (0.02 to 2.18)	0.76 (0.15 to 1.8)	**Placebo**	1 (0.15 to 2.04)	NA
0.15 (0.02 to 1.18)	0.16 (0.02 to 1.22)	0.24 (0.02 to 1.67)	0.25 (0.02 to 1.46)	0.28 (0 to 2.16)	0.28 (0 to 2)	0.28 (0.02 to 1.55)	0.28 (0.02 to 1.56)	0.35 (0.02 to 1.68)	0.43 (0.03 to 1.83)	0.54 (0.03 to 1.91)	0.55 (0.05 to 1.89)	0.63 (0.02 to 2.19)	0.74 (0.07 to 1.99)	0.98 (0.18 to 1.99)	**Thioridazine**	NA
**0.14 (0.04 to 0.41)**	0.16 (0.02 to 1.26)	0.22 (0.04 to 1.02)	0.25 (0.02 to 1.47)	0.26 (0 to 1.95)	0.26 (0.02 to 1.61)	**0.26 (0.07 to 0.82)**	**0.26 (0.09 to 0.69)**	**0.33 (0.11 to 0.81)**	0.42 (0.12 to 1.06)	0.51 (0.17 to 1.12)	0.52 (0.19 to 1.11)	0.59 (0.04 to 1.97)	0.7 (0.05 to 1.95)	0.93 (0.14 to 1.93)	0.95 (0.09 to 2.03)	**Fluphenazine**

The original results given in OR (and their 95% CI) are transformed to RR (left lower half: treatment in column versus treatment in row; right upper half: treatment in row versus treatment in column) using the formula described above. For this transformation, we assumed a discontinuation rate (due to inefficacy) with clozapine of 6% as the control event rate (CER) for all comparisons of active antipsychotic versus clozapine.
Treatments are presented in order of efficacy ranking. Results of the network meta-analysis are reported in the left lower half and results of pairwise meta-analyses in the right upper half. Bold results indicate 95% CI excluding no effect. NA=not available.

Forest plot of results of pairwise meta-analyses

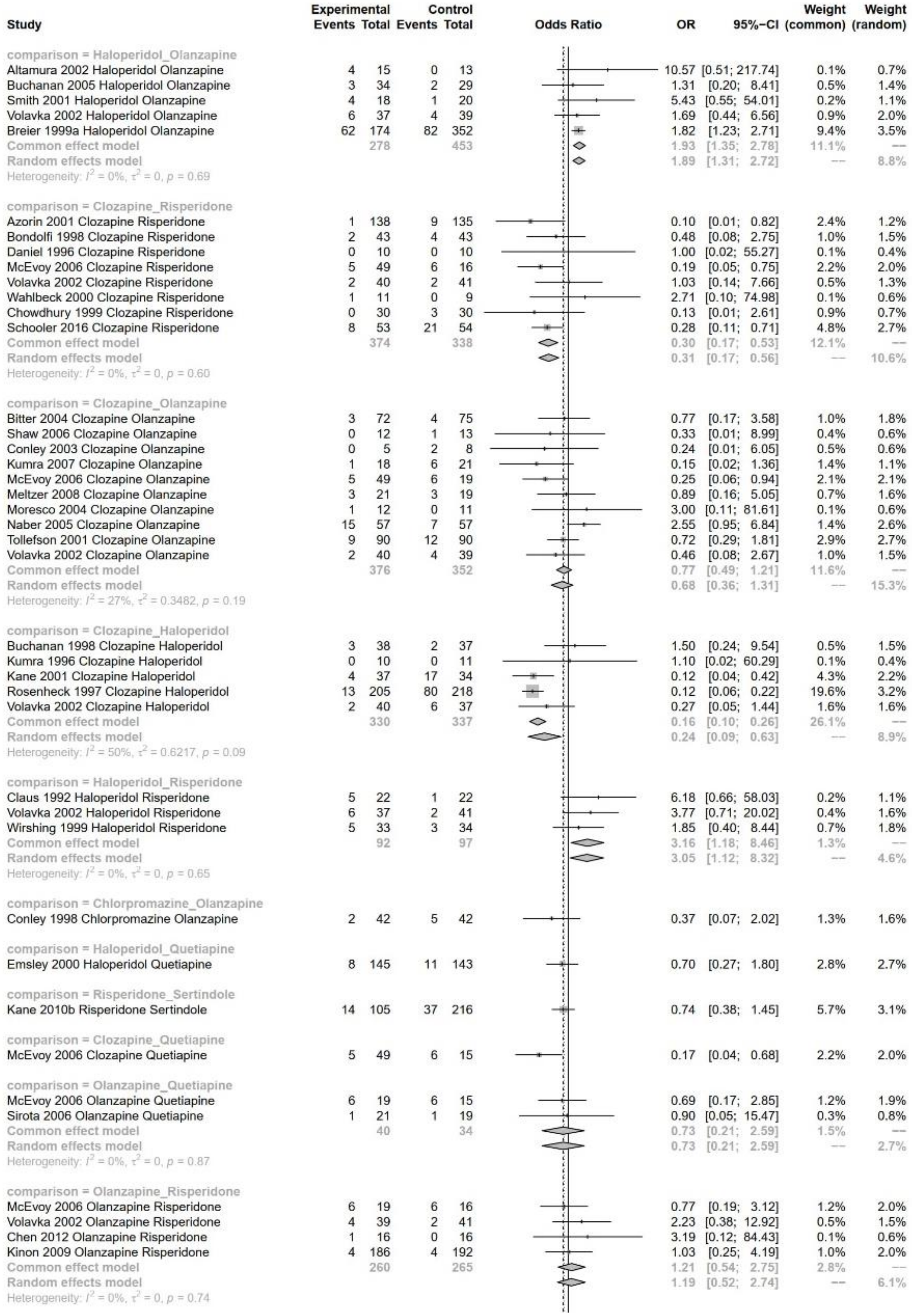

comparison = Quetiapine_Risperidone								
McEvoy 2006 Quetiapine Risperidone	6	15	6	16	1.11	[0.26; 4.72]	0.9%	1.9%
Conley 2005 Quetiapine Risperidone	3	12	3	13	1.11	[0.18; 6.97]	0.6%	1.5%
Common effect model		27		29	1.11	[0.36; 3.46]	1.5%	--
Random effects model					1.11	[0.36; 3.46]	--	3.4%
Heterogeneity: $I^2 = 0\%$, $\tau^2 = 0$, $p = 1.00$								
comparison = Chlorpromazine_Risperidone								
Mercer 1997 Chlorpromazine Risperidone	0	12	0	15	1.24	[0.02; 67.04]	0.1%	0.4%
comparison = Chlorpromazine_Placebo								
Schiele 1961, 06602 Chlorpromazine Placebo	0	20	1	20	0.32	[0.01; 8.26]	0.4%	0.6%
comparison = Chlorpromazine_Thioridazine								
Schiele 1961, 06602 Chlorpromazine Thioridazine	0	20	1	20	0.32	[0.01; 8.26]	0.4%	0.6%
comparison = Chlorpromazine_Trifluoperazine								
Schiele 1961, 06602 Chlorpromazine Trifluoperazine	0	20	0	20	1.00	[0.02; 52.85]	0.1%	0.5%
comparison = Placebo_Thioridazine								
Schiele 1961, 06602 Placebo Thioridazine	1	20	1	20	1.00	[0.06; 17.18]	0.3%	0.8%
comparison = Placebo_Trifluoperazine								
Schiele 1961, 06602 Placebo Trifluoperazine	1	20	0	20	3.15	[0.12; 82.16]	0.1%	0.6%
Marjerrison 1964 Placebo Trifluoperazine	2	34	1	16	0.94	[0.08; 11.17]	0.3%	1.0%
Common effect model		54		36	1.53	[0.22; 10.66]	0.5%	--
Random effects model					1.46	[0.20; 10.51]	--	1.6%
Heterogeneity: $I^2 = 0\%$, $\tau^2 = 0$, $p = 0.56$								
comparison = Thioridazine_Trifluoperazine								
Schiele 1961, 06602 Thioridazine Trifluoperazine	1	20	0	20	3.15	[0.12; 82.16]	0.1%	0.6%
comparison = Aripiprazole_Perphenazine								
Kane 2007a Aripiprazole Perphenazine	10	154	8	146	1.20	[0.46; 3.12]	2.1%	2.6%
comparison = Chlorpromazine_Haloperidol								
Teja 1975 Chlorpromazine Haloperidol	0	14	0	13	0.93	[0.02; 50.30]	0.1%	0.4%
McCreadie 1977 Chlorpromazine Haloperidol	0	10	0	10	1.00	[0.02; 55.27]	0.1%	0.4%
Common effect model		24		23	0.96	[0.06; 16.33]	0.3%	--
Random effects model					0.96	[0.06; 16.33]	--	0.9%
Heterogeneity: $I^2 = 0\%$, $\tau^2 = 0$, $p = 0.98$								
comparison = Fluphenazine_Quetiapine								
Conley 2005 Fluphenazine Quetiapine	8	13	3	12	4.80	[0.86; 26.79]	0.3%	1.6%
comparison = Fluphenazine_Risperidone								
Conley 2005 Fluphenazine Risperidone	8	13	3	13	5.33	[0.97; 29.39]	0.3%	1.6%
comparison = Chlorpromazine_Clozapine								
Hong 1997 Chlorpromazine Clozapine	0	19	0	21	1.10	[0.02; 58.28]	0.1%	0.5%
Honigfeld 1984b Chlorpromazine Clozapine	10	76	4	75	2.69	[0.80; 8.99]	0.9%	2.2%
Common effect model		95		96	2.50	[0.79; 7.88]	1.1%	--
Random effects model					2.49	[0.79; 7.91]	--	2.7%
Heterogeneity: $I^2 = 0\%$, $\tau^2 = 0$, $p = 0.67$								
comparison = Chlorpromazine_Ziprasidone								
Kane 2006 Chlorpromazine Ziprasidone	0	154	0	152	0.99	[0.02; 50.06]	0.1%	0.5%
comparison = Clozapine_Zotepine								
Meyer−Lindenberg 1997 Clozapine Zotepine	0	25	2	25	0.18	[0.01; 4.04]	0.7%	0.7%
comparison = Clozapine_Ziprasidone								
Sacchetti 2009 Clozapine Ziprasidone	2	74	3	73	0.65	[0.11; 4.00]	0.8%	1.5%
comparison = Fluphenazine_Haloperidol								
Hall 1968 Fluphenazine Haloperidol	3	25	2	25	1.57	[0.24; 10.30]	0.5%	1.4%
comparison = Chlorpromazine_Sulpiride								
Toru 1972 Chlorpromazine Sulpiride	0	37	0	38	1.03	[0.02; 53.09]	0.1%	0.5%
comparison = Amisulpride_Olanzapine								
Kahn 2018 Amisulpride Olanzapine	1	47	1	46	0.98	[0.06; 16.12]	0.3%	0.8%
comparison = Haloperidol_Placebo								
Browne 1988 Haloperidol Placebo	3	6	4	5	0.25	[0.02; 3.77]	0.6%	0.9%
comparison = Paliperidone_Paliperidone_LAI								
Actrn12618001113246 Paliperidone Paliperidone_LAI	0	36	0	36	1.00	[0.02; 51.76]	0.1%	0.5%
comparison = Fluphenazine_LAI_Pipotiazine_LAI								
Schlosberg 1978 Fluphenazine_LAI Pipotiazine_LAI	9	30	12	30	0.64	[0.22; 1.87]	2.3%	2.5%
comparison = Fluphenazine_LAI_Placebo								
Schlosberg 1978 Fluphenazine_LAI Placebo	9	30	12	15	0.11	[0.02; 0.47]	3.0%	1.9%
comparison = Pipotiazine_LAI_Placebo								
Schlosberg 1978 Pipotiazine_LAI Placebo	12	30	12	15	0.17	[0.04; 0.72]	2.6%	1.9%
comparison = Clopenthixol_LAI_Perphenazine_LAI								
Ahlfors 1980 Clopenthixol_LAI Perphenazine_LAI	13	87	13	85	0.97	[0.42; 2.24]	3.0%	2.8%
**Common effect model**		**3164**		**3307**	**0.73**	**[0.63; 0.86]**	**100.0%**	**--**
**Random effects model**					**0.76**	**[0.57; 1.01]**	**--**	**100.0%**

Heterogeneity: $I^2 = 52\%$, $\tau^2 = 0.5612$, $p < 0.01$
Test for subgroup differences (fixed effect): $\chi^2_{35} = 120.27$, df = 35 ($p < 0.01$)
Test for subgroup differences (random effects): $\chi^2_{35} = 77.80$, df = 35 ($p < 0.01$)

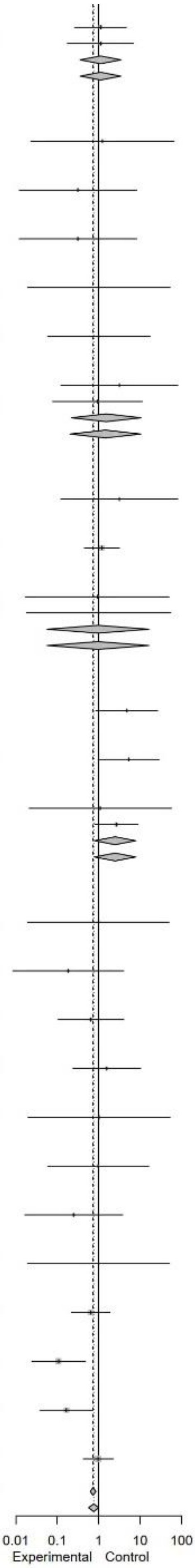

A summary effect size is calculated by pairwise meta-analyses of all studies of a specific comparison. The type of effect size measure is OR.

8.6 Discontinuation due to adverse events

We transformed the original ORs to RRs and EERs vs. CER assuming the baseline discontinuation rate due to adverse events with clozapine of 12% (namely CER). 12% was the average discontinuation rate due to adverse events with clozapine across all clozapine -arms in the network meta-analysis, as estimated by a single-arm meta-analysis of proportions.

Network plot

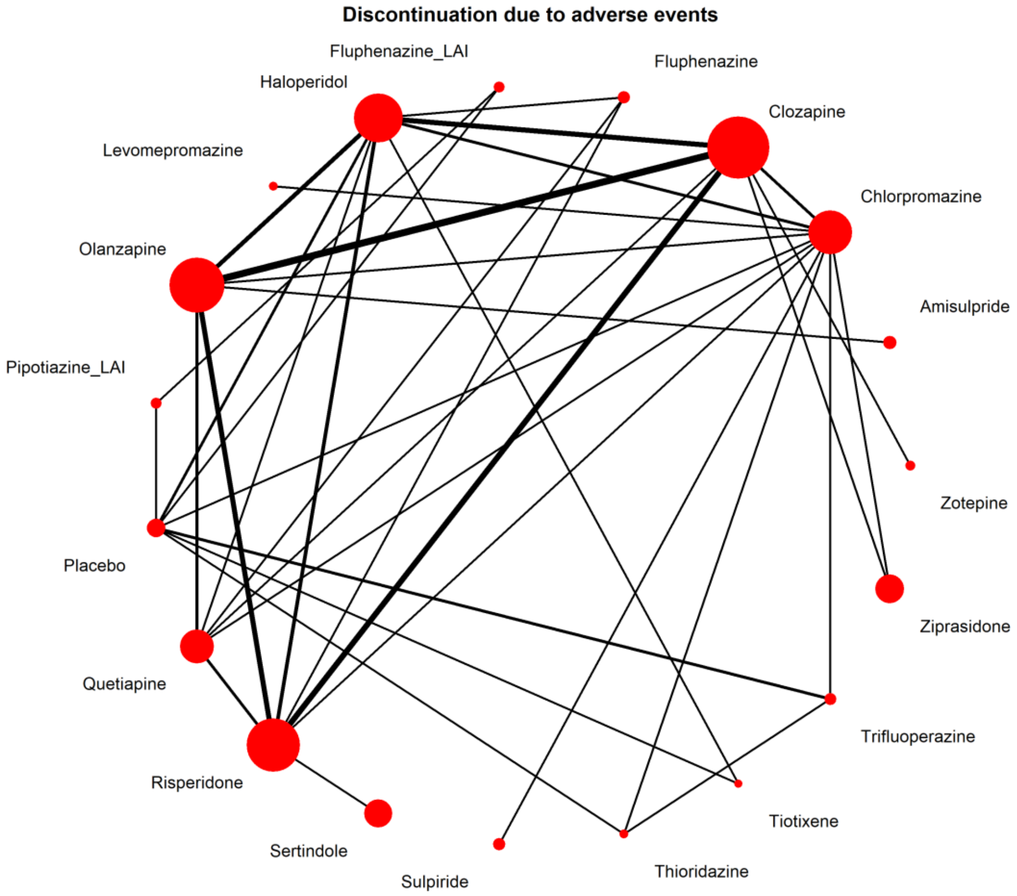

Lines link treatments with direct comparisons in trials; thickness of lines corresponds to the number of trials evaluating the comparison; size of the nodes corresponds to the number of participants assigned to the treatment.

Forest-plot of results of network meta-analysis for antipsychotic drugs versus clozapine

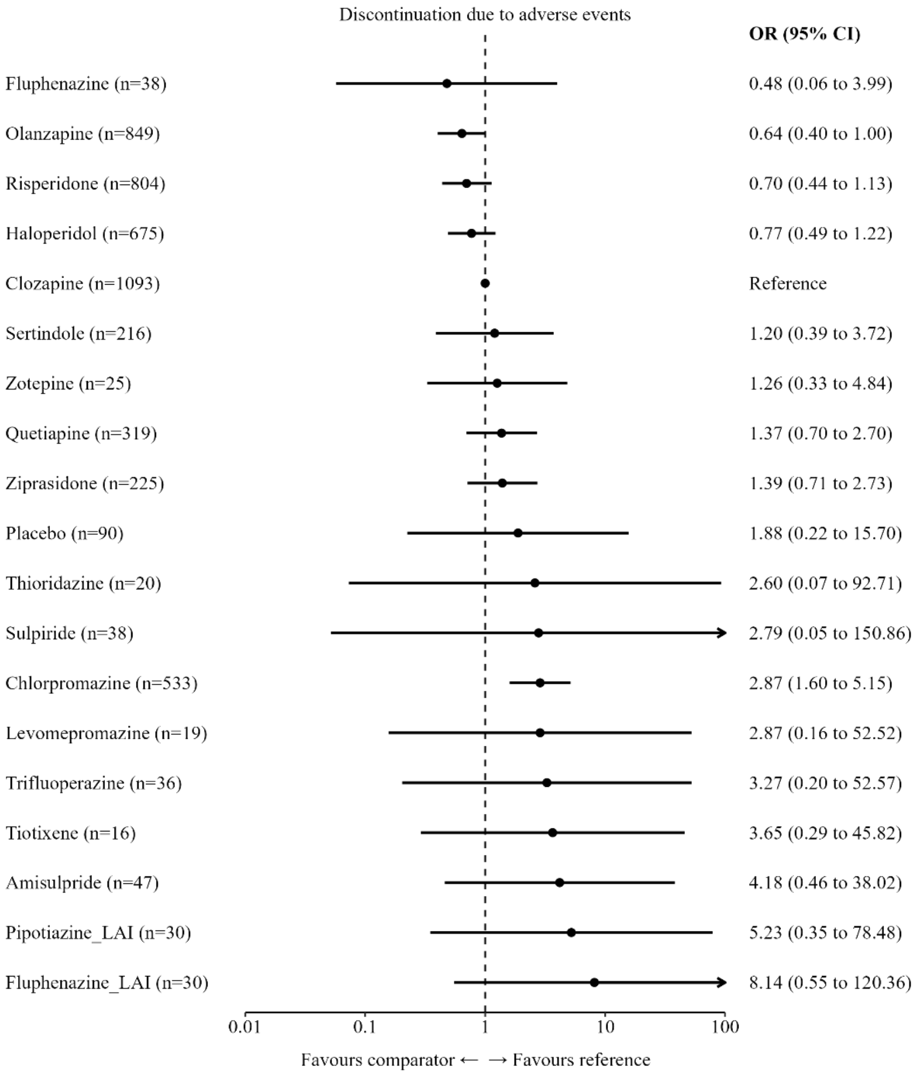

Effect sizes, measured as odds ratio (OR), are from the network meta-analysis. Order of treatments is according to the mean effect size. Reference is clozapine. The direction of the effect is indicated below the x-axis.

Forest plot with RR and EER versus CER

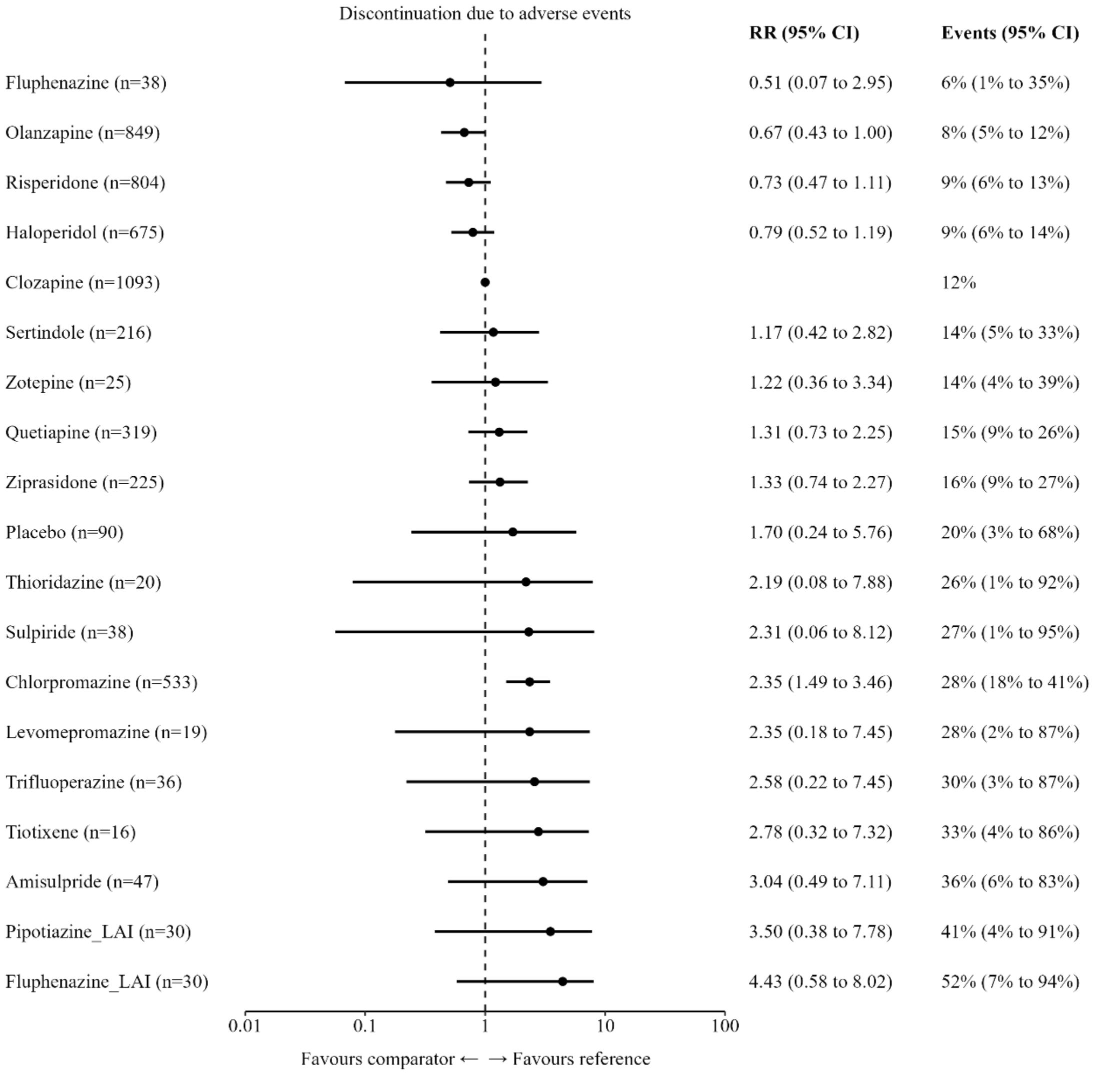

For this forest plot, we transformed the original OR to RR and exposure events rates (EER, called "events" in the forest plot) using the formula indicated above. Therefore, we used an average discontinuation rate (due to adverse events) with clozapine of 12% as the control event rate (CER).
Effect sizes are from the network meta-analysis. Order of treatments is according to the mean effect size. Reference is clozapine. The direction of the effect is indicated below the x-axis.

League table for the outcome: Discontinuation due to adverse events (OR)

**Fluphenazine**	NA	1.00 (0.02 to 54.16)	1.00 (0.06 to 16.93)	NA	NA	NA	0.16 (0.01 to 3.60)	NA	NA	NA	NA	NA	NA	NA	NA	NA	NA	NA
0.75 (0.09 to 6.49)	**Olanzapine**	1.04 (0.52 to 2.10)	0.72 (0.11 to 4.67)	**0.57 (0.32 to 0.99)**	NA	NA	0.55 (0.10 to 3.12)	NA	NA	NA	NA	0.15 (0.02 to 1.27)	NA	NA	NA	0.15 (0.02 to 1.32)	NA	NA
0.68 (0.08 to 5.85)	0.90 (0.53 to 1.53)	**Risperidone**	2.46 (0.41 to 14.74)	0.70 (0.39 to 1.26)	0.59 (0.21 to 1.63)	NA	0.13 (0.01 to 1.15)	NA	NA	NA	NA	2.59 (0.10 to 69.34)	NA	NA	NA	NA	NA	NA
0.62 (0.08 to 5.06)	0.82 (0.45 to 1.52)	0.91 (0.49 to 1.71)	**Haloperidol**	0.84 (0.50 to 1.42)	NA	NA	0.39 (0.13 to 1.14)	NA	0.45 (0.03 to 5.73)	NA	NA	0.51 (0.04 to 6.50)	NA	NA	0.17 (0.01 to 3.73)	NA	NA	NA
0.48 (0.06 to 3.99)	0.64 (0.40 to 1.00)	0.70 (0.44 to 1.13)	0.77 (0.49 to 1.22)	**Clozapine**	NA	0.79 (0.21 to 3.03)	0.44 (0.10 to 1.94)	0.91 (0.41 to 2.00)	NA	NA	NA	**0.35 (0.14 to 0.85)**	NA	NA	NA	NA	NA	NA
0.40 (0.04 to 4.32)	0.53 (0.17 to 1.67)	0.59 (0.21 to 1.63)	0.64 (0.19 to 2.14)	0.83 (0.27 to 2.57)	**Sertindole**	NA	NA	NA	NA	NA	NA	NA	NA	NA	NA	NA	NA	NA
0.38 (0.03 to 4.66)	0.50 (0.12 to 2.08)	0.56 (0.13 to 2.31)	0.61 (0.15 to 2.53)	0.79 (0.21 to 3.03)	0.95 (0.16 to 5.51)	**Zotepine**	NA	NA	NA	NA	NA	NA	NA	NA	NA	NA	NA	NA
0.35 (0.04 to 2.91)	0.46 (0.21 to 1.00)	0.51 (0.23 to 1.12)	0.56 (0.28 to 1.13)	0.73 (0.37 to 1.43)	0.88 (0.24 to 3.18)	0.92 (0.20 to 4.13)	**Quetiapine**	NA	NA	NA	NA	**0.30 (0.12 to 0.72)**	NA	NA	NA	NA	NA	NA
0.34 (0.04 to 3.13)	0.46 (0.21 to 1.01)	0.51 (0.22 to 1.14)	0.55 (0.25 to 1.22)	0.72 (0.37 to 1.40)	0.86 (0.23 to 3.19)	0.91 (0.20 to 4.07)	0.99 (0.41 to 2.40)	**Ziprasidone**	NA	NA	NA	0.75 (0.25 to 2.22)	NA	NA	NA	NA	NA	NA
0.25 (0.01 to 4.90)	0.34 (0.04 to 2.94)	0.37 (0.04 to 3.27)	0.41 (0.05 to 3.36)	0.53 (0.06 to 4.45)	0.64 (0.06 to 7.03)	0.67 (0.05 to 8.30)	0.73 (0.08 to 6.44)	0.74 (0.08 to 6.73)	**Placebo**	1.00 (0.02 to 52.85)	NA	1.00 (0.02 to 52.85)	NA	0.59 (0.06 to 5.97)	0.56 (0.07 to 4.80)	NA	0.36 (0.07 to 1.93)	0.23 (0.04 to 1.21)
0.18 (0.00 to 11.39)	0.24 (0.01 to 8.90)	0.27 (0.01 to 9.89)	0.30 (0.01 to 10.56)	0.38 (0.01 to 13.70)	0.46 (0.01 to 19.49)	0.49 (0.01 to 22.08)	0.53 (0.01 to 19.30)	0.54 (0.01 to 19.88)	0.72 (0.02 to 22.27)	**Thioridazine**	NA	1.00 (0.02 to 52.85)	NA	1.00 (0.02 to 52.85)	NA	NA	NA	NA
0.17 (0.00 to 15.35)	0.23 (0.00 to 12.52)	0.25 (0.00 to 13.91)	0.28 (0.01 to 15.15)	0.36 (0.01 to 19.33)	0.43 (0.01 to 27.04)	0.45 (0.01 to 30.42)	0.49 (0.01 to 26.96)	0.50 (0.01 to 27.65)	0.67 (0.01 to 59.71)	0.93 (0.00 to 189.06)	**Sulpiride**	0.97 (0.02 to 50.37)	NA	NA	NA	NA	NA	NA
0.17 (0.02 to 1.44)	**0.22 (0.11 to 0.45)**	**0.25 (0.12 to 0.51)**	**0.27 (0.14 to 0.53)**	**0.35 (0.19 to 0.63)**	0.42 (0.12 to 1.47)	0.44 (0.10 to 1.91)	**0.48 (0.24 to 0.95)**	0.49 (0.23 to 1.02)	0.65 (0.08 to 5.54)	0.91 (0.03 to 31.85)	0.97 (0.02 to 50.37)	**Chlorpromazine**	1.00 (0.06 to 17.25)	1.00 (0.02 to 52.85)	NA	NA	NA	NA
0.17 (0.00 to 5.94)	0.22 (0.01 to 4.16)	0.25 (0.01 to 4.63)	0.27 (0.01 to 5.03)	0.35 (0.02 to 6.38)	0.42 (0.02 to 9.42)	0.44 (0.02 to 10.83)	0.48 (0.03 to 8.95)	0.49 (0.03 to 9.22)	0.65 (0.02 to 23.02)	0.91 (0.01 to 86.50)	0.97 (0.01 to 126.43)	1.00 (0.06 to 17.25)	**Levomepromazine**	NA	NA	NA	NA	NA
0.15 (0.00 to 4.65)	0.19 (0.01 to 3.22)	0.22 (0.01 to 3.58)	0.24 (0.01 to 3.76)	0.31 (0.02 to 4.92)	0.37 (0.02 to 7.32)	0.39 (0.02 to 8.45)	0.42 (0.03 to 7.01)	0.43 (0.03 to 7.26)	0.57 (0.06 to 5.34)	0.80 (0.02 to 28.48)	0.85 (0.01 to 106.19)	0.88 (0.05 to 14.04)	0.88 (0.02 to 46.70)	**Trifluoperazine**	NA	NA	NA	NA
0.13 (0.01 to 3.41)	0.17 (0.01 to 2.26)	0.19 (0.01 to 2.51)	0.21 (0.02 to 2.59)	0.27 (0.02 to 3.44)	0.33 (0.02 to 5.22)	0.35 (0.02 to 6.07)	0.38 (0.03 to 4.95)	0.38 (0.03 to 5.15)	0.51 (0.07 to 3.97)	0.71 (0.01 to 34.47)	0.77 (0.01 to 84.07)	0.79 (0.06 to 10.08)	0.79 (0.02 to 35.97)	0.90 (0.05 to 17.11)	**Tiotixene**	NA	NA	NA
0.11 (0.01 to 2.41)	0.15 (0.02 to 1.32)	0.17 (0.02 to 1.55)	0.18 (0.02 to 1.74)	0.24 (0.03 to 2.17)	0.29 (0.02 to 3.32)	0.30 (0.02 to 3.99)	0.33 (0.03 to 3.25)	0.33 (0.03 to 3.33)	0.45 (0.02 to 9.52)	0.62 (0.01 to 41.19)	0.67 (0.01 to 63.32)	0.69 (0.07 to 6.64)	0.69 (0.02 to 26.16)	0.78 (0.02 to 26.94)	0.87 (0.03 to 24.86)	**Amisulpride**	NA	NA
0.09 (0.00 to 2.74)	0.12 (0.01 to 1.88)	0.13 (0.01 to 2.09)	0.15 (0.01 to 2.17)	0.19 (0.01 to 2.87)	0.23 (0.01 to 4.29)	0.24 (0.01 to 4.96)	0.26 (0.02 to 4.11)	0.27 (0.02 to 4.26)	0.36 (0.07 to 1.93)	0.50 (0.01 to 22.66)	0.53 (0.00 to 64.34)	0.55 (0.04 to 8.31)	0.55 (0.01 to 28.11)	0.63 (0.04 to 10.21)	0.70 (0.05 to 9.84)	0.80 (0.02 to 26.14)	**Pipotiazine_LAI**	0.64 (0.22 to 1.87)
0.06 (0.00 to 1.74)	0.08 (0.01 to 1.19)	0.09 (0.01 to 1.32)	0.09 (0.01 to 1.38)	0.12 (0.01 to 1.82)	0.15 (0.01 to 2.72)	0.16 (0.01 to 3.15)	0.17 (0.01 to 2.60)	0.17 (0.01 to 2.70)	0.23 (0.04 to 1.21)	0.32 (0.01 to 14.42)	0.34 (0.00 to 41.04)	0.35 (0.02 to 5.27)	0.35 (0.01 to 17.90)	0.40 (0.02 to 6.47)	0.45 (0.03 to 6.23)	0.51 (0.02 to 16.62)	0.64 (0.22 to 1.87)	**Fluphenazine_LAI**

Treatments are presented in order of efficacy ranking. Results of the network meta-analysis are reported in the left lower half and results of pairwise meta-analyses in the right upper half. Each cell provides the effect estimate and the corresponding 95% credible interval (95% CI) of a comparison (left lower half: treatment in column versus treatment in row; right upper half: treatment in row versus treatment in column). The type of effect size measure is odd ratio (OR). Bold results indicate 95% CI excluding no effect. NA=not available.

League table for the outcome: Discontinuation due to adverse events (RR)

**Fluphenazine**	NA	1 (0.02 to 22.16)	1 (0.06 to 11.48)	NA	NA	NA	0.17 (0.01 to 3.17)	NA	NA	NA	NA	NA	NA	NA	NA	NA	NA	NA
0.75 (0.09 to 5.72)	**Olanzapine**	1.04 (0.53 to 2.04)	0.73 (0.11 to 4.21)	**0.58 (0.33 to 0.99)**	NA	NA	0.56 (0.1 to 2.81)	NA	NA	NA	NA	0.16 (0.02 to 1.24)	NA	NA	NA	0.17 (0.02 to 1.26)	NA	NA
0.69 (0.08 to 5.17)	0.9 (0.54 to 1.51)	**Risperidone**	2.36 (0.42 to 10.46)	0.71 (0.4 to 1.25)	0.6 (0.22 to 1.58)	NA	0.14 (0.01 to 1.14)	NA	NA	NA	NA	2.23 (0.11 to 8.69)	NA	NA	NA	NA	NA	NA
0.63 (0.08 to 4.51)	0.82 (0.46 to 1.5)	0.91 (0.5 to 1.67)	**Haloperidol**	0.85 (0.51 to 1.4)	NA	NA	0.4 (0.14 to 1.13)	NA	0.47 (0.03 to 4.31)	NA	NA	0.54 (0.04 to 4.16)	NA	NA	0.19 (0.01 to 2.77)	NA	NA	NA
0.49 (0.06 to 3.58)	0.65 (0.41 to 1)	0.71 (0.45 to 1.12)	0.78 (0.5 to 1.21)	**Clozapine**	NA	0.8 (0.22 to 2.76)	0.45 (0.1 to 1.85)	0.91 (0.42 to 1.9)	NA	NA	NA	**0.37 (0.15 to 0.86)**	NA	NA	NA	NA	NA	NA
0.41 (0.04 to 3.75)	0.54 (0.18 to 1.62)	0.6 (0.22 to 1.58)	0.65 (0.2 to 2.03)	0.84 (0.28 to 2.4)	**Sertindole**	NA	NA	NA	NA	NA	NA	NA	NA	NA	NA	NA	NA	NA
0.39 (0.03 to 3.96)	0.51 (0.13 to 1.98)	0.57 (0.14 to 2.17)	0.62 (0.16 to 2.36)	0.8 (0.22 to 2.76)	0.95 (0.17 to 4.53)	**Zotepine**	NA	NA	NA	NA	NA	NA	NA	NA	NA	NA	NA	NA
0.36 (0.04 to 2.65)	0.47 (0.22 to 1)	0.52 (0.24 to 1.11)	0.57 (0.29 to 1.12)	0.74 (0.38 to 1.4)	0.89 (0.25 to 2.86)	0.92 (0.21 to 3.55)	**Quetiapine**	NA	NA	NA	NA	**0.32 (0.13 to 0.74)**	NA	NA	NA	NA	NA	NA
0.35 (0.04 to 2.81)	0.47 (0.22 to 1.01)	0.52 (0.23 to 1.13)	0.56 (0.26 to 1.21)	0.73 (0.38 to 1.37)	0.87 (0.24 to 2.86)	0.91 (0.21 to 3.5)	0.99 (0.42 to 2.23)	**Ziprasidone**	NA	NA	NA	0.77 (0.27 to 1.97)	NA	NA	NA	NA	NA	NA
0.26 (0.01 to 3.85)	0.36 (0.04 to 2.59)	0.39 (0.04 to 2.82)	0.43 (0.05 to 2.89)	0.55 (0.06 to 3.59)	0.66 (0.06 to 4.95)	0.69 (0.05 to 5.5)	0.74 (0.09 to 4.67)	0.75 (0.09 to 4.81)	**Placebo**	1 (0.02 to 9.09)	NA	1 (0.02 to 8.4)	NA	0.62 (0.07 to 3.82)	0.59 (0.08 to 3.23)	NA	0.4 (0.08 to 1.67)	0.29 (0.05 to 1.15)
0.19 (0 to 5.8)	0.26 (0.01 to 5.14)	0.29 (0.01 to 5.42)	0.32 (0.01 to 5.6)	0.4 (0.01 to 6.29)	0.48 (0.01 to 7.18)	0.51 (0.01 to 7.47)	0.55 (0.01 to 7.15)	0.56 (0.01 to 7.22)	0.74 (0.02 to 7.49)	**Thioridazine**	NA	1 (0.02 to 8.4)	NA	1 (0.02 to 7.68)	NA	NA	NA	NA
0.19 (0 to 6.35)	0.25 (0 to 5.86)	0.27 (0 to 6.11)	0.3 (0.01 to 6.32)	0.38 (0.01 to 6.88)	0.46 (0.01 to 7.57)	0.48 (0.01 to 7.79)	0.52 (0.01 to 7.56)	0.53 (0.01 to 7.61)	0.69 (0.01 to 8.78)	0.94 (0 to 9.66)	**Sulpiride**	0.97 (0.02 to 8.34)	NA	NA	NA	NA	NA	NA
0.19 (0.02 to 1.38)	**0.24 (0.12 to 0.48)**	**0.27 (0.13 to 0.54)**	**0.29 (0.15 to 0.56)**	**0.37 (0.21 to 0.65)**	0.45 (0.13 to 1.4)	0.47 (0.11 to 1.75)	**0.51 (0.26 to 0.95)**	0.52 (0.25 to 1.02)	0.67 (0.09 to 3.79)	0.92 (0.03 to 7.68)	0.97 (0.02 to 8.34)	**Chlorpromazine**	1 (0.07 to 6.49)	1 (0.02 to 7.68)	NA	NA	NA	NA
0.19 (0 to 3.95)	0.24 (0.01 to 3.15)	0.27 (0.01 to 3.38)	0.29 (0.01 to 3.56)	0.37 (0.02 to 4.12)	0.45 (0.02 to 5.07)	0.47 (0.02 to 5.41)	0.51 (0.03 to 4.94)	0.52 (0.03 to 5.01)	0.67 (0.02 to 7.09)	0.92 (0.01 to 8.89)	0.97 (0.01 to 9.16)	1 (0.07 to 6.49)	**Levomepromazine**	NA	NA	NA	NA	NA
0.17 (0 to 3.29)	0.21 (0.01 to 2.57)	0.24 (0.01 to 2.77)	0.26 (0.01 to 2.86)	0.34 (0.02 to 3.41)	0.4 (0.02 to 4.26)	0.42 (0.02 to 4.58)	0.45 (0.03 to 4.17)	0.46 (0.03 to 4.25)	0.6 (0.07 to 3.58)	0.82 (0.02 to 6.92)	0.86 (0.01 to 8.22)	0.89 (0.06 to 5.66)	0.89 (0.02 to 7.55)	**Trifluoperazine**	NA	NA	NA	NA
0.15 (0.01 to 2.61)	0.19 (0.01 to 1.95)	0.21 (0.01 to 2.1)	0.23 (0.02 to 2.15)	0.3 (0.02 to 2.62)	0.36 (0.02 to 3.39)	0.38 (0.02 to 3.69)	0.41 (0.03 to 3.29)	0.41 (0.03 to 3.37)	0.54 (0.08 to 2.88)	0.74 (0.01 to 6.54)	0.79 (0.01 to 7.25)	0.81 (0.07 to 4.67)	0.81 (0.02 to 6.59)	0.91 (0.06 to 5.6)	**Tiotixene**	NA	NA	NA
0.13 (0.01 to 2)	0.17 (0.02 to 1.26)	0.19 (0.02 to 1.44)	0.2 (0.02 to 1.57)	0.27 (0.03 to 1.85)	0.32 (0.02 to 2.48)	0.33 (0.02 to 2.78)	0.37 (0.03 to 2.45)	0.37 (0.03 to 2.49)	0.49 (0.02 to 4.25)	0.66 (0.01 to 6.01)	0.7 (0.01 to 6.28)	0.72 (0.08 to 3.65)	0.72 (0.02 to 5.61)	0.81 (0.02 to 5.64)	0.89 (0.03 to 5.55)	**Amisulpride**	NA	NA
0.11 (0 to 2.11)	0.14 (0.01 to 1.64)	0.15 (0.01 to 1.76)	0.18 (0.01 to 1.81)	0.22 (0.01 to 2.18)	0.26 (0.01 to 2.75)	0.28 (0.01 to 2.97)	0.3 (0.02 to 2.69)	0.31 (0.02 to 2.74)	0.4 (0.08 to 1.67)	0.55 (0.01 to 4.84)	0.58 (0 to 5.47)	0.6 (0.05 to 3.71)	0.6 (0.01 to 5.02)	0.67 (0.05 to 3.98)	0.74 (0.06 to 3.93)	0.83 (0.02 to 4.96)	**Pipotiazine_LAI**	0.7 (0.27 to 1.53)
0.08 (0 to 1.46)	0.1 (0.01 to 1.14)	0.12 (0.01 to 1.22)	0.12 (0.01 to 1.26)	0.15 (0.01 to 1.51)	0.19 (0.01 to 1.89)	0.2 (0.01 to 2.04)	0.22 (0.01 to 1.85)	0.22 (0.01 to 1.89)	0.29 (0.05 to 1.15)	0.39 (0.01 to 3.27)	0.41 (0 to 3.67)	0.42 (0.03 to 2.53)	0.42 (0.01 to 3.38)	0.47 (0.03 to 2.71)	0.52 (0.04 to 2.67)	0.58 (0.03 to 3.34)	0.7 (0.27 to 1.53)	**Fluphenazine_LAI**

The original results given in OR (and their 95% CI) are transformed to RR (left lower half: treatment in column versus treatment in row; right upper half: treatment in row versus treatment in column) using the formula described above. For this transformation, we assumed a discontinuation rate (due to adverse events) with clozapine of 12% as the control event rate (CER) for all comparisons of active antipsychotic versus clozapine.
Treatments are presented in order of efficacy ranking. Results of the network meta-analysis are reported in the left lower half and results of pairwise meta-analyses in the right upper half. Bold results indicate 95% CI excluding no effect. NA=not available.

Forest plot of results of pairwise meta-analyses

Study	Experimental Events	Experimental Total	Control Events	Control Total	OR	95%-CI	Weight (common)	Weight (random)
comparison = Haloperidol_Olanzapine								
Altamura 2002 Haloperidol Olanzapine	1	15	0	13	2.79	[0.10; 74.63]	0.2%	0.4%
Buchanan 2005 Haloperidol Olanzapine	0	34	0	29	0.86	[0.02; 44.44]	0.3%	0.3%
Smith 2001 Haloperidol Olanzapine	0	18	0	20	1.11	[0.02; 58.71]	0.2%	0.3%
Volavka 2002 Haloperidol Olanzapine	0	37	0	39	1.05	[0.02; 54.45]	0.2%	0.3%
Common effect model		104		101	1.44	[0.23; 8.93]	0.9%	--
Random effects model					1.40	[0.21; 9.11]	--	1.4%
Heterogeneity: $I^2 = 0\%$, $\tau^2 = 0$, $p = 0.97$								
comparison = Clozapine_Risperidone								
Azorin 2001 Clozapine Risperidone	16	138	12	135	1.34	[0.61; 2.96]	5.2%	5.1%
Bondolfi 1998 Clozapine Risperidone	1	43	1	43	1.00	[0.06; 16.52]	0.5%	0.6%
Daniel 1996 Clozapine Risperidone	0	10	2	10	0.16	[0.01; 3.85]	1.2%	0.5%
McEvoy 2006 Clozapine Risperidone	5	49	0	16	4.08	[0.21; 77.90]	0.3%	0.5%
Volavka 2002 Clozapine Risperidone	5	40	2	41	2.79	[0.51; 15.28]	0.8%	1.5%
Wahlbeck 2000 Clozapine Risperidone	2	11	1	9	1.78	[0.13; 23.52]	0.4%	0.7%
Chowdhury 1999 Clozapine Risperidone	4	30	3	30	1.38	[0.28; 6.80]	1.3%	1.7%
Common effect model		321		284	1.43	[0.80; 2.53]	9.7%	--
Random effects model					1.43	[0.79; 2.60]	--	10.6%
Heterogeneity: $I^2 = 0\%$, $\tau^2 = 0$, $p = 0.81$								
comparison = Clozapine_Olanzapine								
Bitter 2004 Clozapine Olanzapine	7	72	7	75	1.05	[0.35; 3.15]	3.0%	3.1%
Shaw 2006 Clozapine Olanzapine	0	12	0	13	1.08	[0.02; 58.65]	0.2%	0.3%
Conley 2003 Clozapine Olanzapine	0	5	1	8	0.45	[0.02; 13.41]	0.5%	0.4%
Kumra 2007 Clozapine Olanzapine	2	18	1	21	2.50	[0.21; 30.12]	0.4%	0.7%
McEvoy 2006 Clozapine Olanzapine	5	49	1	19	2.05	[0.22; 18.76]	0.6%	0.9%
Meltzer 2008 Clozapine Olanzapine	0	21	0	19	0.91	[0.02; 47.94]	0.2%	0.3%
Moresco 2004 Clozapine Olanzapine	5	12	1	11	7.14	[0.68; 75.22]	0.3%	0.8%
Naber 2005 Clozapine Olanzapine	6	57	6	57	1.00	[0.30; 3.31]	2.6%	2.8%
Tollefson 2001 Clozapine Olanzapine	13	90	4	90	3.63	[1.14; 11.60]	1.7%	2.9%
Volavka 2002 Clozapine Olanzapine	5	40	0	39	12.24	[0.65; 229.29]	0.2%	0.5%
Common effect model		376		352	1.99	[1.17; 3.37]	9.8%	--
Random effects model					1.83	[1.01; 3.34]	--	12.9%
Heterogeneity: $I^2 = 0\%$, $\tau^2 = 0.0591$, $p = 0.63$								
comparison = Clozapine_Haloperidol								
Buchanan 1998 Clozapine Haloperidol	2	38	0	37	5.14	[0.24; 110.70]	0.2%	0.5%
Kumra 1996 Clozapine Haloperidol	3	10	1	11	4.29	[0.37; 50.20]	0.3%	0.8%
Kane 2001 Clozapine Haloperidol	2	37	3	34	0.59	[0.09; 3.77]	1.4%	1.3%
Rosenheck 1997 Clozapine Haloperidol	26	205	27	218	1.03	[0.58; 1.83]	11.1%	7.3%
Volavka 2002 Clozapine Haloperidol	5	40	0	37	11.62	[0.62; 217.87]	0.2%	0.5%
Common effect model		330		337	1.30	[0.79; 2.14]	13.3%	--
Random effects model					1.41	[0.64; 3.13]	--	10.4%
Heterogeneity: $I^2 = 21\%$, $\tau^2 = 0.1801$, $p = 0.28$								
comparison = Haloperidol_Risperidone								
Claus 1992 Haloperidol Risperidone	1	22	0	22	3.14	[0.12; 81.35]	0.2%	0.4%
Volavka 2002 Haloperidol Risperidone	0	37	2	41	0.21	[0.01; 4.53]	1.1%	0.5%
Wirshing 1999 Haloperidol Risperidone	0	33	3	34	0.13	[0.01; 2.71]	1.6%	0.5%
Common effect model		92		97	0.39	[0.09; 1.71]	3.0%	--
Random effects model					0.41	[0.06; 2.60]	--	1.5%
Heterogeneity: $I^2 = 10\%$, $\tau^2 = 0.1662$, $p = 0.33$								
comparison = Chlorpromazine_Olanzapine								
Conley 1998 Chlorpromazine Olanzapine	6	42	1	42	6.83	[0.79; 59.48]	0.4%	1.0%
comparison = Haloperidol_Quetiapine								
Emsley 2000 Haloperidol Quetiapine	5	145	12	143	0.39	[0.13; 1.14]	5.6%	3.3%
comparison = Haloperidol_Placebo								
Howard 1974 Haloperidol Placebo	0	17	1	16	0.30	[0.01; 7.79]	0.7%	0.4%
Browne 1988 Haloperidol Placebo	0	6	0	5	0.85	[0.01; 50.10]	0.2%	0.3%
Common effect model		23		21	0.43	[0.04; 5.17]	1.0%	--
Random effects model					0.45	[0.03; 5.73]	--	0.7%
Heterogeneity: $I^2 = 0\%$, $\tau^2 = 0$, $p = 0.69$								
comparison = Haloperidol_Tiotixene								
Howard 1974 Haloperidol Tiotixene	0	17	2	16	0.17	[0.01; 3.73]	1.2%	0.5%
comparison = Placebo_Tiotixene								
Howard 1974 Placebo Tiotixene	1	16	2	16	0.47	[0.04; 5.73]	0.9%	0.7%
comparison = Risperidone_Sertindole								
Kane 2010b Risperidone Sertindole	5	105	17	216	0.59	[0.21; 1.63]	5.1%	3.5%
comparison = Clozapine_Quetiapine								
McEvoy 2006 Clozapine Quetiapine	5	49	3	15	0.45	[0.09; 2.18]	2.0%	1.7%
comparison = Olanzapine_Quetiapine								
McEvoy 2006 Olanzapine Quetiapine	1	19	3	15	0.22	[0.02; 2.40]	1.5%	0.8%
Sirota 2006 Olanzapine Quetiapine	1	21	0	19	2.85	[0.11; 74.34]	0.2%	0.4%
Common effect model		40		34	0.57	[0.11; 3.02]	1.8%	--
Random effects model					0.62	[0.05; 7.17]	--	1.3%
Heterogeneity: $I^2 = 35\%$, $\tau^2 = 1.1386$, $p = 0.22$								

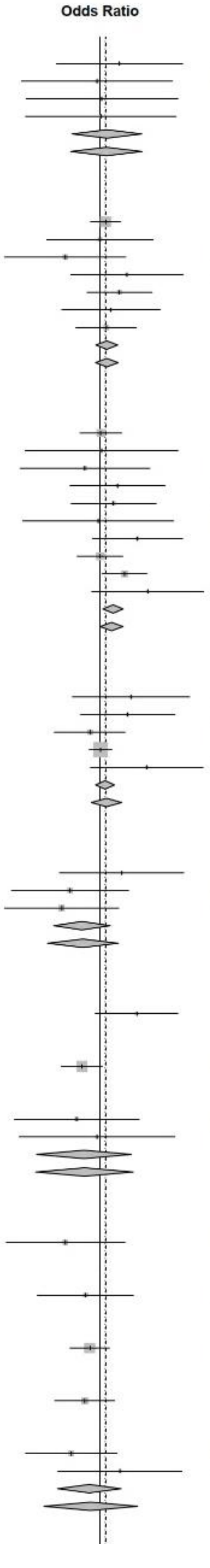

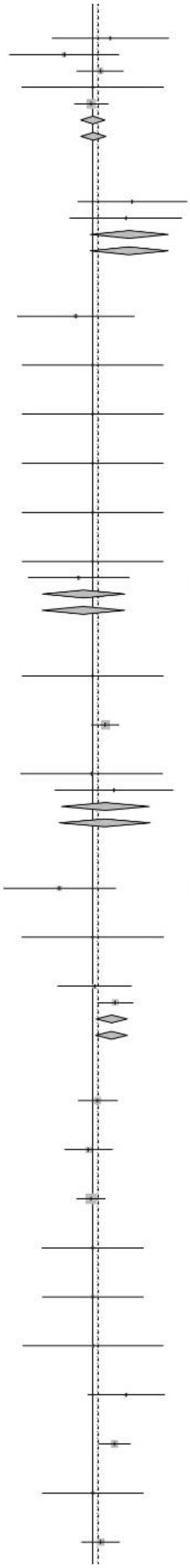

comparison = Olanzapine_Risperidone							
McEvoy 2006 Olanzapine Risperidone	1	19	0	16	2.68 [0.10; 70.31]	0.2%	0.4%
Volavka 2002 Olanzapine Risperidone	0	39	2	41	0.20 [0.01; 4.30]	1.2%	0.5%
Alvarez 2006 Olanzapine Risperidone	6	124	4	123	1.51 [0.42; 5.50]	1.8%	2.4%
Chen 2012 Olanzapine Risperidone	0	16	0	16	1.00 [0.02; 53.46]	0.2%	0.3%
Kinon 2009 Olanzapine Risperidone	9	186	10	192	0.93 [0.37; 2.33]	4.5%	4.1%
Common effect model		384		388	1.01 [0.51; 1.99]	8.0%	--
Random effects model					1.04 [0.52; 2.10]	--	7.8%
Heterogeneity: $I^2 = 0\%$, $\tau^2 = 0$, $p = 0.77$							
comparison = Quetiapine_Risperidone							
McEvoy 2006 Quetiapine Risperidone	3	15	0	16	9.24 [0.44; 195.69]	0.2%	0.5%
Conley 2005 Quetiapine Risperidone	2	12	0	13	6.43 [0.28; 148.77]	0.2%	0.5%
Common effect model		27		29	7.82 [0.88; 69.47]	0.4%	--
Random effects model					7.75 [0.87; 69.19]	--	1.0%
Heterogeneity: $I^2 = 0\%$, $\tau^2 = 0$, $p = 0.87$							
comparison = Chlorpromazine_Risperidone							
Mercer 1997 Chlorpromazine Risperidone	0	12	1	15	0.39 [0.01; 10.37]	0.6%	0.4%
comparison = Chlorpromazine_Placebo							
Schiele 1961, 06602 Chlorpromazine Placebo	0	20	0	20	1.00 [0.02; 52.85]	0.2%	0.3%
comparison = Chlorpromazine_Thioridazine							
Schiele 1961, 06602 Chlorpromazine Thioridazine	0	20	0	20	1.00 [0.02; 52.85]	0.2%	0.3%
comparison = Chlorpromazine_Trifluoperazine							
Schiele 1961, 06602 Chlorpromazine Trifluoperazine	0	20	0	20	1.00 [0.02; 52.85]	0.2%	0.3%
comparison = Placebo_Thioridazine							
Schiele 1961, 06602 Placebo Thioridazine	0	20	0	20	1.00 [0.02; 52.85]	0.2%	0.3%
comparison = Placebo_Trifluoperazine							
Schiele 1961, 06602 Placebo Trifluoperazine	0	20	0	20	1.00 [0.02; 52.85]	0.2%	0.3%
Marjerrison 1964 Placebo Trifluoperazine	1	34	1	16	0.45 [0.03; 7.77]	0.6%	0.6%
Common effect model		54		36	0.60 [0.06; 6.06]	0.9%	--
Random effects model					0.59 [0.06; 5.97]	--	0.9%
Heterogeneity: $I^2 = 0\%$, $\tau^2 = 0$, $p = 0.75$							
comparison = Thioridazine_Trifluoperazine							
Schiele 1961, 06602 Thioridazine Trifluoperazine	0	20	0	20	1.00 [0.02; 52.85]	0.2%	0.3%
comparison = Aripiprazole_Perphenazine							
Kane 2007a Aripiprazole Perphenazine	22	154	11	146	2.05 [0.95; 4.38]	4.7%	5.3%
comparison = Chlorpromazine_Haloperidol							
Teja 1975 Chlorpromazine Haloperidol	0	14	0	13	0.93 [0.02; 50.30]	0.2%	0.3%
McCreadie 1977 Chlorpromazine Haloperidol	1	10	0	10	3.32 [0.12; 91.60]	0.2%	0.4%
Common effect model		24		23	2.04 [0.17; 23.87]	0.5%	--
Random effects model					1.97 [0.15; 25.29]	--	0.7%
Heterogeneity: $I^2 = 0\%$, $\tau^2 = 0$, $p = 0.63$							
comparison = Fluphenazine_Quetiapine							
Conley 2005 Fluphenazine Quetiapine	0	13	2	12	0.16 [0.01; 3.60]	1.2%	0.5%
comparison = Fluphenazine_Risperidone							
Conley 2005 Fluphenazine Risperidone	0	13	0	13	1.00 [0.02; 54.16]	0.2%	0.3%
comparison = Chlorpromazine_Clozapine							
Hong 1997 Chlorpromazine Clozapine	2	19	2	21	1.12 [0.14; 8.82]	0.8%	1.1%
Honigfeld 1984b Chlorpromazine Clozapine	18	76	6	75	3.57 [1.33; 9.58]	2.2%	3.7%
Common effect model		95		96	2.91 [1.21; 6.97]	3.1%	--
Random effects model					2.88 [1.18; 7.01]	--	4.8%
Heterogeneity: $I^2 = 0\%$, $\tau^2 = 0$, $p = 0.32$							
comparison = Chlorpromazine_Ziprasidone							
Kane 2006 Chlorpromazine Ziprasidone	8	154	6	152	1.33 [0.45; 3.94]	2.8%	3.2%
comparison = Clozapine_Zotepine							
Meyer−Lindenberg 1997 Clozapine Zotepine	5	25	6	25	0.79 [0.21; 3.03]	2.3%	2.3%
comparison = Clozapine_Ziprasidone							
Sacchetti 2009 Clozapine Ziprasidone	15	74	16	73	0.91 [0.41; 2.00]	6.2%	5.0%
comparison = Chlorpromazine_Levomepromazine							
Lal 2006 Chlorpromazine Levomepromazine	1	19	1	19	1.00 [0.06; 17.25]	0.5%	0.6%
comparison = Fluphenazine_Haloperidol							
Hall 1968 Fluphenazine Haloperidol	1	25	1	25	1.00 [0.06; 16.93]	0.5%	0.6%
comparison = Chlorpromazine_Sulpiride							
Toru 1972 Chlorpromazine Sulpiride	0	37	0	38	1.03 [0.02; 53.09]	0.2%	0.3%
comparison = Amisulpride_Olanzapine							
Kahn 2018 Amisulpride Olanzapine	6	47	1	46	6.59 [0.76; 57.04]	0.4%	1.0%
comparison = Chlorpromazine_Quetiapine							
AstraZeneca 5077IL/0031 Chlorpromazine Quetiapine	21	130	7	130	3.39 [1.39; 8.27]	2.8%	4.3%
comparison = Paliperidone_Paliperidone_LAI							
Actrn12618001113246 Paliperidone Paliperidone_LAI	1	36	1	36	1.00 [0.06; 16.63]	0.5%	0.6%
comparison = Fluphenazine_LAI_Pipotiazine_LAI							
Schlosberg 1978 Fluphenazine_LAI Pipotiazine_LAI	12	30	9	30	1.56 [0.53; 4.53]	2.6%	3.3%

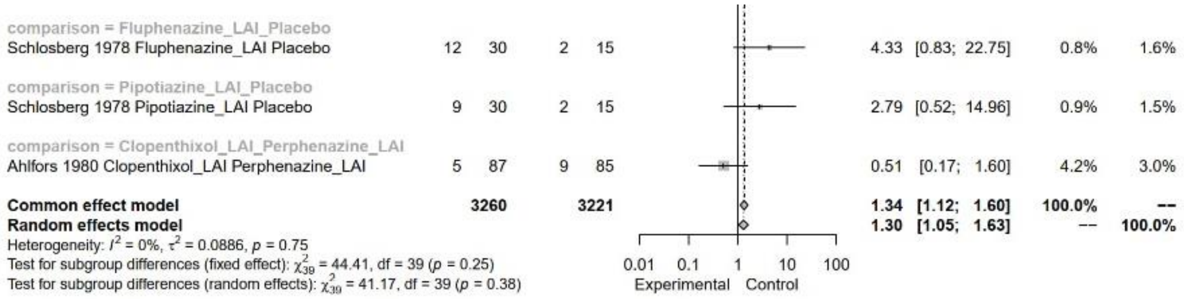

A summary effect size is calculated by pairwise meta-analyses of all studies of a specific comparison. The type of effect size measure is OR.

8.7 Use of antiparkinsonian medication

We transformed the original ORs to RRs and EERs vs. CER assuming the baseline risk of using antiparkinsonian medication with clozapine of 24% (namely CER). 24% was the average risk of using antiparkinsonian medication with clozapine across all clozapine-arms in the network meta-analysis, as estimated by a single-arm meta-analysis of proportions.

Network plot

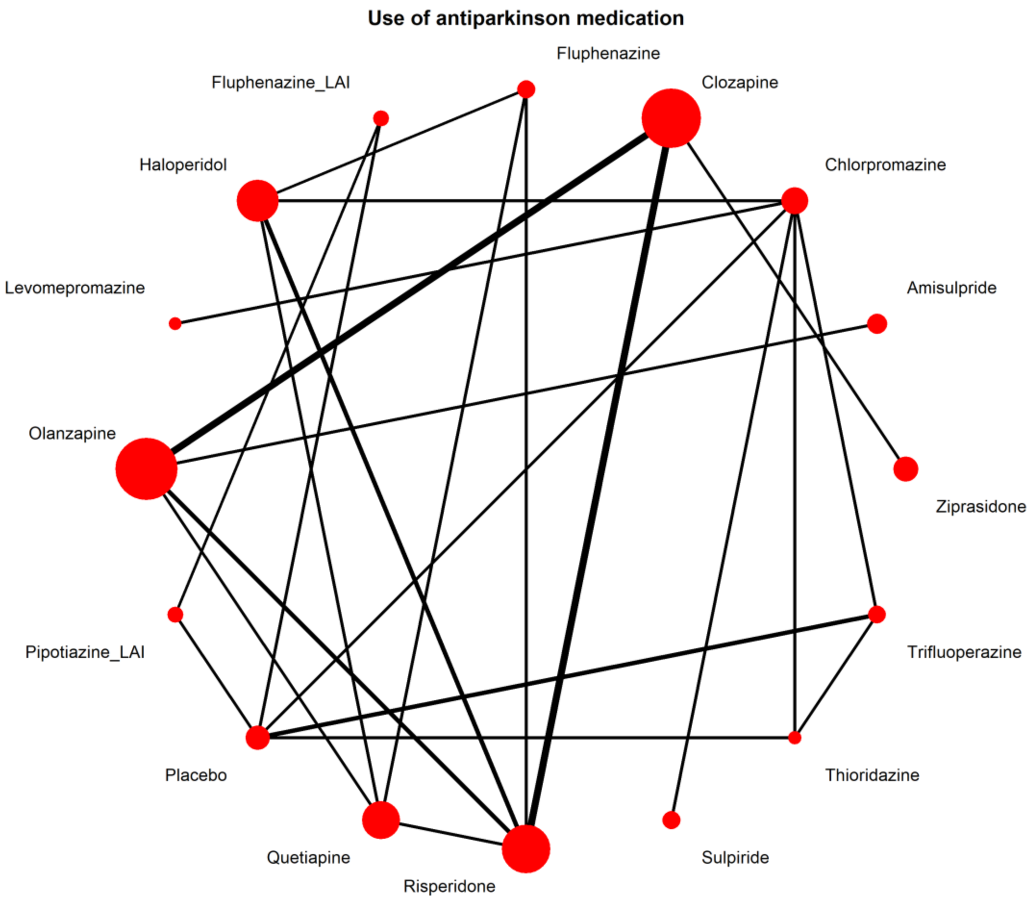

Lines link treatments with direct comparisons in trials; thickness of lines corresponds to the number of trials evaluating the comparison; size of the nodes corresponds to the number of participants assigned to the treatment.

Forest-plot of results of network meta-analysis for antipsychotic drugs versus clozapine

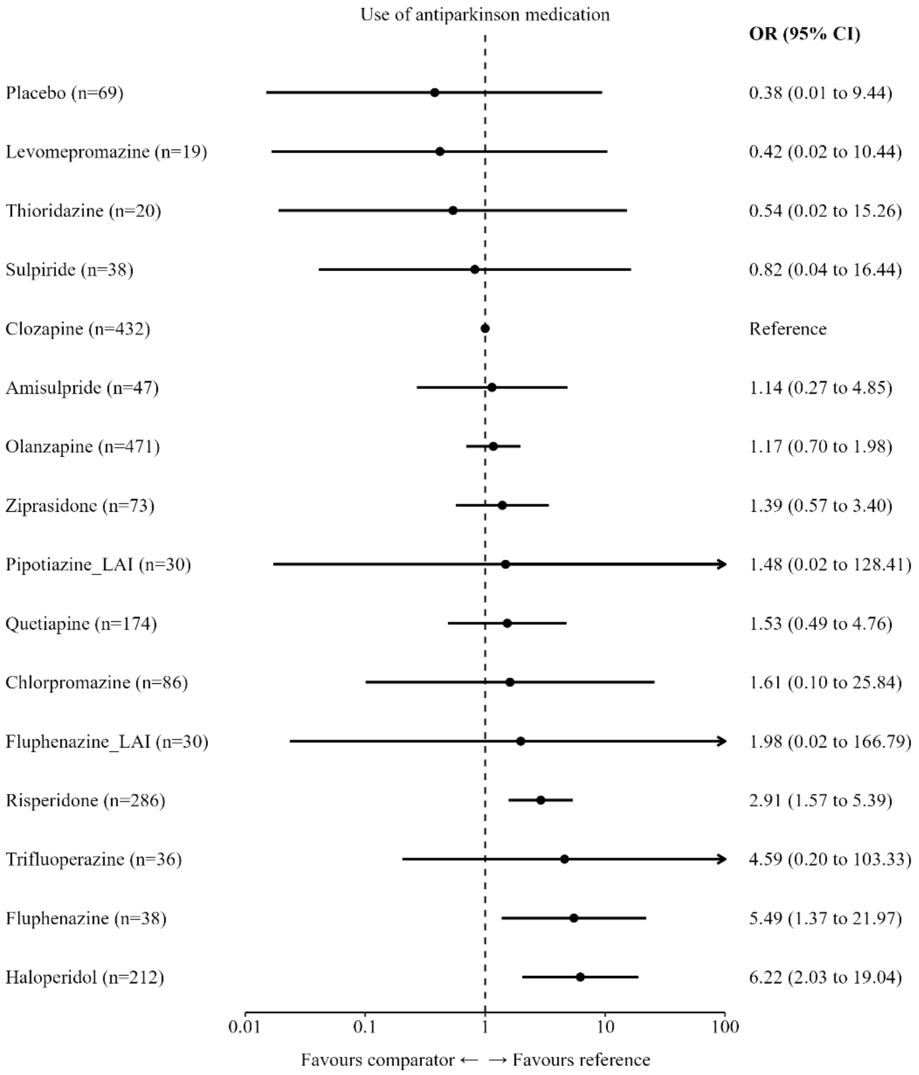

Effect sizes, measured as odds ratio (OR), are from the network meta-analysis. Order of treatments is according to the mean effect size. Reference is clozapine. The direction of the effect is indicated below the x-axis.

<u>Forest plot with RR and EER versus CER</u>

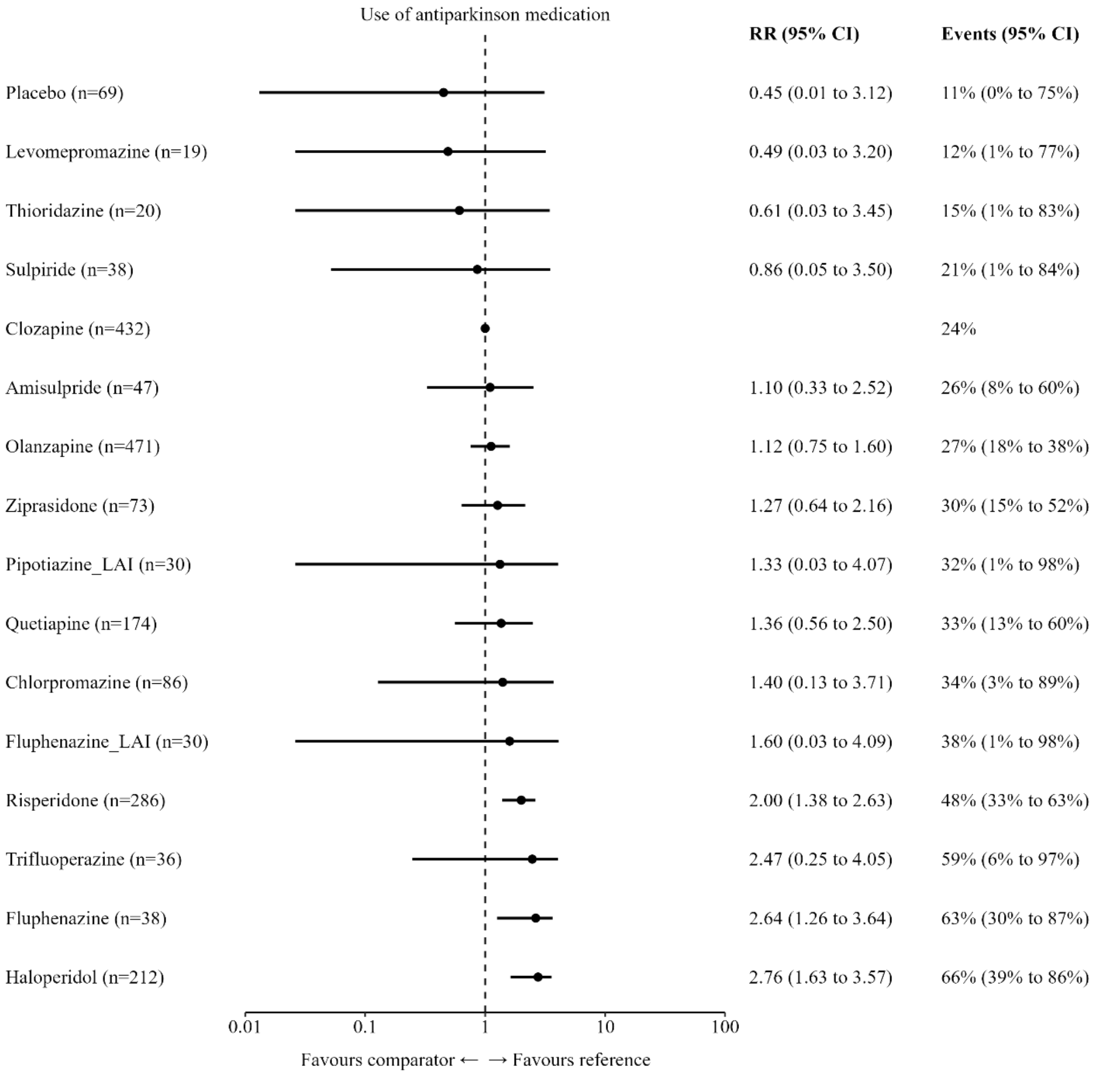

For this forest plot, we transformed the original OR to RR and exposure events rates (EER, called "events" in the forest plot) using the formula indicated above. Therefore, we used an average rate of using antiparkinsonian medication with clozapine of 24% as the control event rate (CER).
Effect sizes are from the network meta-analysis. Order of treatments is according to the mean effect size. Reference is clozapine. The direction of the effect is indicated below the x-axis.

<u>League table for the outcome: Use of antiparkinsonian medication (OR)</u>

**Placebo**	NA	1.00 (0.12 to 8.56)	NA	NA	NA	NA	NA	0.25 (0.01 to 5.54)	NA	0.33 (0.05 to 2.17)	0.19 (0.01 to 3.99)	NA	**0.08 (0.02 to 0.30)**	NA	NA
0.90 (0.09 to 9.21)	**Levomepromazine**	NA	NA	NA	NA	NA	NA	NA	NA	0.26 (0.05 to 1.33)	NA	NA	NA	NA	NA
0.70 (0.10 to 4.92)	0.77 (0.06 to 9.32)	**Thioridazine**	NA	NA	NA	NA	NA	NA	NA	0.33 (0.05 to 2.17)	NA	NA	**0.14 (0.02 to 0.82)**	NA	NA
0.46 (0.06 to 3.37)	0.51 (0.07 to 3.72)	0.65 (0.07 to 5.84)	**Sulpiride**	NA	NA	NA	NA	NA	NA	0.51 (0.16 to 1.58)	NA	NA	NA	NA	NA
0.38 (0.01 to 9.44)	0.42 (0.02 to 10.44)	0.54 (0.02 to 15.26)	0.82 (0.04 to 16.44)	**Clozapine**	NA	1.02 (0.57 to 1.81)	0.72 (0.29 to 1.75)	NA	NA	NA	NA	**0.24 (0.11 to 0.55)**	NA	NA	NA
0.33 (0.01 to 10.64)	0.36 (0.01 to 11.76)	0.47 (0.01 to 17.05)	0.72 (0.03 to 18.86)	0.87 (0.21 to 3.71)	**Amisulpride**	0.98 (0.25 to 3.75)	NA	NA	NA	NA	NA	NA	NA	NA	NA
0.32 (0.01 to 7.91)	0.35 (0.01 to 8.75)	0.46 (0.02 to 12.80)	0.70 (0.04 to 13.76)	0.85 (0.51 to 1.44)	0.98 (0.25 to 3.75)	**Olanzapine**	NA	NA	1.12 (0.25 to 5.07)	NA	NA	**0.46 (0.24 to 0.92)**	NA	NA	NA
0.27 (0.01 to 7.66)	0.30 (0.01 to 8.47)	0.39 (0.01 to 12.33)	0.59 (0.03 to 13.45)	0.72 (0.29 to 1.75)	0.82 (0.15 to 4.49)	0.84 (0.30 to 2.37)	**Ziprasidone**	NA	NA	NA	NA	NA	NA	NA	NA
0.25 (0.01 to 5.54)	0.28 (0.01 to 13.32)	0.36 (0.01 to 13.95)	0.55 (0.01 to 21.82)	0.67 (0.01 to 58.37)	0.77 (0.01 to 80.54)	0.79 (0.01 to 67.63)	0.94 (0.01 to 88.77)	**Pipotiazine_LAI**	NA	NA	0.75 (0.15 to 3.73)	NA	NA	NA	NA
0.25 (0.01 to 5.97)	0.27 (0.01 to 6.60)	0.35 (0.01 to 9.67)	0.54 (0.03 to 10.37)	0.65 (0.21 to 2.04)	0.75 (0.13 to 4.17)	0.77 (0.26 to 2.23)	0.91 (0.21 to 3.87)	0.97 (0.01 to 82.01)	**Quetiapine**	NA	NA	1.83 (0.23 to 14.65)	NA	0.75 (0.12 to 4.79)	**0.16 (0.04 to 0.64)**
0.23 (0.05 to 1.20)	0.26 (0.05 to 1.33)	0.33 (0.05 to 2.17)	0.51 (0.16 to 1.58)	0.62 (0.04 to 9.92)	0.71 (0.03 to 15.20)	0.73 (0.05 to 11.41)	0.86 (0.05 to 15.89)	0.92 (0.03 to 30.26)	0.95 (0.06 to 14.58)	**Chlorpromazine**	NA	NA	0.41 (0.09 to 1.76)	NA	0.26 (0.02 to 3.28)
0.19 (0.01 to 3.99)	0.21 (0.00 to 9.68)	0.27 (0.01 to 10.11)	0.42 (0.01 to 15.83)	0.51 (0.01 to 42.59)	0.58 (0.01 to 58.83)	0.59 (0.01 to 49.34)	0.70 (0.01 to 64.80)	0.75 (0.15 to 3.73)	0.77 (0.01 to 63.51)	0.82 (0.03 to 25.94)	**Fluphenazine_LAI**	NA	NA	NA	NA
0.13 (0.01 to 3.10)	0.14 (0.01 to 3.43)	0.19 (0.01 to 5.03)	0.28 (0.01 to 5.39)	**0.34 (0.19 to 0.64)**	0.39 (0.09 to 1.69)	**0.40 (0.23 to 0.71)**	0.48 (0.16 to 1.42)	0.51 (0.01 to 42.77)	0.53 (0.18 to 1.50)	0.56 (0.04 to 8.43)	0.68 (0.01 to 55.54)	**Risperidone**	NA	0.41 (0.06 to 3.02)	0.50 (0.15 to 1.75)
**0.08 (0.02 to 0.30)**	**0.09 (0.01 to 0.79)**	**0.12 (0.02 to 0.69)**	0.18 (0.03 to 1.10)	0.22 (0.01 to 4.91)	0.25 (0.01 to 7.30)	0.26 (0.01 to 5.66)	0.30 (0.01 to 7.74)	0.32 (0.01 to 9.13)	0.33 (0.02 to 7.24)	0.35 (0.09 to 1.45)	0.43 (0.02 to 11.76)	0.63 (0.03 to 13.60)	**Trifluoperazine**	NA	NA
0.07 (0.00 to 1.72)	0.08 (0.00 to 1.90)	0.10 (0.00 to 2.78)	0.15 (0.01 to 2.99)	**0.18 (0.05 to 0.73)**	0.21 (0.03 to 1.40)	**0.21 (0.06 to 0.82)**	0.25 (0.05 to 1.32)	0.27 (0.00 to 23.38)	**0.28 (0.08 to 0.99)**	0.29 (0.02 to 4.70)	0.36 (0.00 to 30.37)	0.53 (0.15 to 1.90)	0.84 (0.04 to 18.82)	**Fluphenazine**	1.23 (0.30 to 4.97)
0.06 (0.00 to 1.24)	0.07 (0.00 to 1.37)	0.09 (0.00 to 2.02)	0.13 (0.01 to 2.13)	**0.16 (0.05 to 0.49)**	0.18 (0.03 to 1.03)	**0.19 (0.06 to 0.55)**	**0.22 (0.05 to 0.94)**	0.24 (0.00 to 17.89)	**0.25 (0.09 to 0.68)**	0.26 (0.02 to 3.28)	0.32 (0.00 to 23.22)	0.47 (0.18 to 1.24)	0.74 (0.04 to 13.49)	0.88 (0.29 to 2.70)	**Haloperidol**

Treatments are presented in order of efficacy ranking. Results of the network meta-analysis are reported in the left lower half and results of pairwise meta-analyses in the right upper half. Each cell provides the effect estimate and the corresponding 95% credible interval (95% CI) of a comparison (left lower half: treatment in column versus treatment in row; right upper half: treatment in row versus treatment in column). The type of effect size measure is odd ratio (OR). Bold results indicate 95% CI excluding no effect. NA=not available.

League table for the outcome: Use of antiparkinsonian medication (RR)

**Placebo**	NA	1 (0.13 to 6.29)	NA	NA	NA	NA	NA	0.27 (0.01 to 3.61)	NA	0.36 (0.06 to 1.89)	0.22 (0.01 to 2.75)	NA	**0.11 (0.03 to 0.38)**	NA	NA
0.9 (0.09 to 7.04)	**Levomepromazine**	NA	NA	NA	NA	NA	NA	NA	NA	0.29 (0.06 to 1.28)	NA	NA	NA	NA	NA
0.71 (0.1 to 4.15)	0.78 (0.06 to 6.67)	**Thioridazine**	NA	NA	NA	NA	NA	NA	NA	0.36 (0.06 to 1.89)	NA	NA	**0.19 (0.03 to 0.87)**	NA	NA
0.48 (0.06 to 2.91)	0.53 (0.07 to 3.14)	0.67 (0.07 to 4.4)	**Sulpiride**	NA	NA	NA	NA	NA	NA	0.54 (0.18 to 1.47)	NA	NA	NA	NA	NA
0.4 (0.01 to 5.59)	0.44 (0.02 to 5.89)	0.56 (0.02 to 7.05)	0.83 (0.04 to 7.27)	**Clozapine**	NA	1.02 (0.59 to 1.68)	0.74 (0.31 to 1.62)	NA	NA	NA	NA	**0.28 (0.13 to 0.61)**	NA	NA	NA
0.35 (0.01 to 5.67)	0.38 (0.01 to 5.94)	0.49 (0.01 to 6.93)	0.74 (0.03 to 7.18)	0.88 (0.23 to 2.98)	**Amisulpride**	0.98 (0.27 to 2.97)	NA	NA	NA	NA	NA	NA	NA	NA	NA
0.34 (0.01 to 4.76)	0.37 (0.01 to 5.03)	0.49 (0.02 to 6.02)	0.72 (0.04 to 6.2)	0.86 (0.54 to 1.38)	0.98 (0.27 to 2.97)	**Olanzapine**	NA	NA	1.1 (0.28 to 3.39)	NA	NA	**0.52 (0.28 to 0.94)**	NA	NA	NA
0.29 (0.01 to 4.44)	0.32 (0.01 to 4.67)	0.42 (0.01 to 5.52)	0.62 (0.03 to 5.71)	0.74 (0.31 to 1.62)	0.84 (0.17 to 3.25)	0.85 (0.32 to 2.06)	**Ziprasidone**	NA	NA	NA	NA	NA	NA	NA	NA
0.27 (0.01 to 3.61)	0.31 (0.01 to 5.43)	0.39 (0.01 to 5.52)	0.58 (0.01 to 6.32)	0.7 (0.01 to 7.52)	0.79 (0.01 to 7.77)	0.81 (0.01 to 7.64)	0.95 (0.01 to 7.83)	**Pipotiazine_LAI**	NA	NA	0.78 (0.17 to 2.64)	NA	NA	NA	NA
0.28 (0.01 to 3.72)	0.3 (0.01 to 3.92)	0.38 (0.01 to 4.7)	0.57 (0.03 to 4.84)	0.68 (0.23 to 1.81)	0.77 (0.15 to 3.01)	0.79 (0.29 to 1.94)	0.92 (0.23 to 2.87)	0.97 (0.01 to 7.53)	**Quetiapine**	NA	NA	1.56 (0.27 to 3.83)	NA	0.82 (0.17 to 2.13)	**0.23 (0.06 to 0.73)**
0.25 (0.06 to 1.17)	0.29 (0.06 to 1.28)	0.36 (0.06 to 1.89)	0.54 (0.18 to 1.47)	0.65 (0.05 to 4.66)	0.74 (0.03 to 5.44)	0.76 (0.06 to 4.93)	0.88 (0.06 to 5.51)	0.93 (0.03 to 6.44)	0.96 (0.07 to 5.37)	**Chlorpromazine**	NA	NA	0.5 (0.12 to 1.44)	NA	0.35 (0.03 to 1.81)
0.22 (0.01 to 2.75)	0.24 (0 to 4.19)	0.3 (0.01 to 4.25)	0.46 (0.01 to 4.88)	0.55 (0.01 to 5.85)	0.62 (0.01 to 6.04)	0.63 (0.01 to 5.94)	0.73 (0.01 to 6.09)	0.78 (0.17 to 2.64)	0.8 (0.01 to 6.08)	0.84 (0.04 to 5.44)	**Fluphenazine_LAI**	NA	NA	NA	NA
0.16 (0.01 to 2.16)	0.17 (0.01 to 2.28)	0.23 (0.01 to 2.74)	0.33 (0.01 to 2.82)	**0.39 (0.23 to 0.69)**	0.45 (0.11 to 1.48)	**0.46 (0.27 to 0.76)**	0.54 (0.19 to 1.31)	0.57 (0.01 to 4.43)	0.59 (0.22 to 1.36)	0.62 (0.05 to 3.32)	0.73 (0.01 to 4.51)	**Risperidone**	NA	0.51 (0.09 to 1.82)	0.61 (0.22 to 1.38)
**0.11 (0.03 to 0.38)**	**0.12 (0.01 to 0.84)**	**0.16 (0.03 to 0.76)**	0.24 (0.04 to 1.07)	0.28 (0.01 to 2.29)	0.32 (0.01 to 2.57)	0.33 (0.01 to 2.4)	0.38 (0.01 to 2.61)	0.4 (0.01 to 2.71)	0.41 (0.03 to 2.57)	0.43 (0.12 to 1.28)	0.52 (0.03 to 2.84)	0.71 (0.04 to 2.91)	**Trifluoperazine**	NA	NA
0.1 (0 to 1.39)	0.11 (0 to 1.47)	0.14 (0 to 1.75)	0.21 (0.01 to 1.81)	**0.25 (0.07 to 0.8)**	0.28 (0.04 to 1.24)	**0.28 (0.09 to 0.87)**	0.33 (0.07 to 1.19)	0.36 (0 to 2.8)	**0.37 (0.11 to 0.99)**	0.38 (0.03 to 2.12)	0.46 (0 to 2.85)	0.63 (0.21 to 1.47)	0.89 (0.06 to 2.74)	**Fluphenazine**	1.14 (0.4 to 2.05)
0.09 (0 to 1.14)	0.1 (0 to 1.21)	0.13 (0 to 1.48)	0.19 (0.02 to 1.52)	**0.23 (0.08 to 0.6)**	0.25 (0.05 to 1.02)	**0.27 (0.09 to 0.66)**	**0.31 (0.08 to 0.96)**	0.33 (0 to 2.54)	**0.34 (0.13 to 0.77)**	0.35 (0.03 to 1.81)	0.42 (0 to 2.6)	0.58 (0.25 to 1.14)	0.82 (0.06 to 2.47)	0.92 (0.39 to 1.68)	**Haloperidol**

The original results given in OR (and their 95% CI) are transformed to RR (left lower half: treatment in column versus treatment in row; right upper half: treatment in row versus treatment in column) using the formula described above. For this transformation, we assumed a rate of using antiparkinsonian medication with clozapine of 24% as the control event rate (CER) for all comparisons of active antipsychotic versus clozapine.

Treatments are presented in order of efficacy ranking. Results of the network meta-analysis are reported in the left lower half and results of pairwise meta-analyses in the right upper half. Bold results indicate 95% CI excluding no effect. NA=not available.

Forest plot of results of pairwise meta-analyses

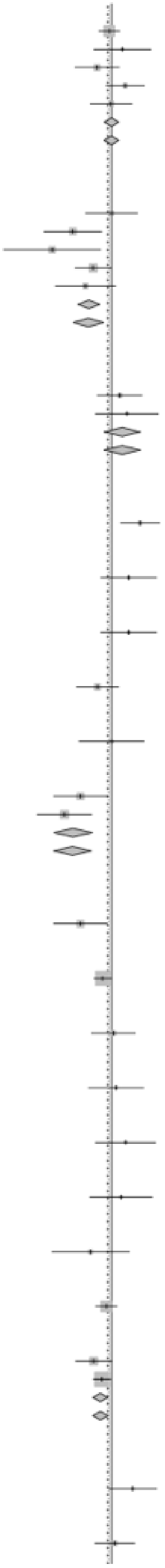

Study	Experimental Events	Experimental Total	Control Events	Control Total	OR	95%-CI	Weight (common)	Weight (random)
comparison = Clozapine_Olanzapine								
Bitter 2004 Clozapine Olanzapine	29	72	33	75	0.86	[0.45; 1.65]	7.8%	4.7%
Meltzer 2008 Clozapine Olanzapine	4	21	2	19	2.00	[0.32; 12.41]	0.7%	2.3%
Naber 2005 Clozapine Olanzapine	3	57	7	57	0.40	[0.10; 1.62]	2.7%	3.0%
Tollefson 2001 Clozapine Olanzapine	9	90	4	90	2.39	[0.71; 8.06]	1.4%	3.4%
Volavka 2002 Clozapine Olanzapine	5	40	5	39	0.97	[0.26; 3.66]	1.8%	3.1%
Common effect model		280		280	1.00	[0.63; 1.58]	14.3%	--
Random effects model					0.99	[0.61; 1.60]	--	16.4%
Heterogeneity: $I^2 = 9\%$, $\tau^2 = < 0.0001$, $p = 0.36$								
comparison = Clozapine_Risperidone								
Bondolfi 1998 Clozapine Risperidone	3	43	3	43	1.00	[0.19; 5.26]	1.1%	2.5%
Breier 1999 Clozapine Risperidone	2	14	10	15	0.08	[0.01; 0.53]	3.3%	2.2%
Daniel 1996 Clozapine Risperidone	0	10	7	10	0.02	[0.00; 0.50]	2.9%	1.1%
Volavka 2002 Clozapine Risperidone	5	40	13	41	0.31	[0.10; 0.97]	4.5%	3.5%
Wahlbeck 2000 Clozapine Risperidone	3	11	6	9	0.19	[0.03; 1.28]	1.9%	2.1%
Common effect model		118		118	0.23	[0.12; 0.47]	13.8%	--
Random effects model					0.23	[0.09; 0.60]	--	11.4%
Heterogeneity: $I^2 = 39\%$, $\tau^2 = 0.4183$, $p = 0.16$								
comparison = Haloperidol_Risperidone								
Claus 1992 Haloperidol Risperidone	6	22	4	22	1.69	[0.40; 7.07]	1.2%	2.9%
See 1999 Haloperidol Risperidone	8	10	6	10	2.67	[0.36; 19.71]	0.5%	2.0%
Common effect model		32		32	1.97	[0.62; 6.31]	1.7%	--
Random effects model					1.97	[0.61; 6.32]	--	4.9%
Heterogeneity: $I^2 = 0\%$, $\tau^2 = 0$, $p = 0.72$								
comparison = Haloperidol_Quetiapine								
Emsley 2000 Haloperidol Quetiapine	17	145	3	143	6.20	[1.77; 21.65]	1.1%	3.3%
comparison = Chlorpromazine_Placebo								
Schiele 1961, 06602 Chlorpromazine Placebo	5	20	2	20	3.00	[0.51; 17.74]	0.6%	2.3%
comparison = Chlorpromazine_Thioridazine								
Schiele 1961, 06602 Chlorpromazine Thioridazine	5	20	2	20	3.00	[0.51; 17.74]	0.6%	2.3%
comparison = Chlorpromazine_Trifluoperazine								
Schiele 1961, 06602 Chlorpromazine Trifluoperazine	5	20	9	20	0.41	[0.11; 1.56]	2.7%	3.1%
comparison = Placebo_Thioridazine								
Schiele 1961, 06602 Placebo Thioridazine	2	20	2	20	1.00	[0.13; 7.89]	0.7%	1.9%
comparison = Placebo_Trifluoperazine								
Schiele 1961, 06602 Placebo Trifluoperazine	2	20	9	20	0.14	[0.02; 0.75]	3.3%	2.4%
Marjerrison 1964 Placebo Trifluoperazine	2	34	9	16	0.05	[0.01; 0.28]	4.6%	2.4%
Common effect model		54		36	0.08	[0.03; 0.29]	7.9%	--
Random effects model					0.08	[0.02; 0.28]	--	4.8%
Heterogeneity: $I^2 = 0\%$, $\tau^2 = 0$, $p = 0.41$								
comparison = Thioridazine_Trifluoperazine								
Schiele 1961, 06602 Thioridazine Trifluoperazine	2	20	9	20	0.14	[0.02; 0.75]	3.3%	2.4%
comparison = Aripiprazole_Perphenazine								
Kane 2007a Aripiprazole Perphenazine	27	154	40	146	0.56	[0.32; 0.98]	13.6%	4.9%
comparison = Olanzapine_Quetiapine								
Sirota 2006 Olanzapine Quetiapine	6	21	5	19	1.12	[0.28; 4.51]	1.5%	3.0%
comparison = Fluphenazine_Quetiapine								
Conley 2005 Fluphenazine Quetiapine	4	13	3	12	1.33	[0.23; 7.74]	0.9%	2.4%
comparison = Fluphenazine_Risperidone								
Conley 2005 Fluphenazine Risperidone	4	13	2	13	2.44	[0.36; 16.55]	0.6%	2.1%
comparison = Quetiapine_Risperidone								
Conley 2005 Quetiapine Risperidone	3	12	2	13	1.83	[0.25; 13.47]	0.6%	2.0%
comparison = Chlorpromazine_Haloperidol								
McCreadie 1977 Chlorpromazine Haloperidol	1	10	3	10	0.26	[0.02; 3.06]	1.1%	1.5%
comparison = Clozapine_Ziprasidone								
Sacchetti 2009 Clozapine Ziprasidone	44	74	49	73	0.72	[0.37; 1.41]	8.0%	4.6%
comparison = Olanzapine_Risperidone								
Volavka 2002 Olanzapine Risperidone	5	39	13	41	0.32	[0.10; 1.00]	4.4%	3.5%
Alvarez 2006 Olanzapine Risperidone	31	124	47	123	0.54	[0.31; 0.93]	14.2%	4.9%
Common effect model		163		164	0.49	[0.30; 0.79]	18.7%	--
Random effects model					0.49	[0.30; 0.80]	--	8.4%
Heterogeneity: $I^2 = 0\%$, $\tau^2 = 0$, $p = 0.41$								
comparison = Chlorpromazine_Levomepromazine								
Lal 2006 Chlorpromazine Levomepromazine	8	19	3	19	3.88	[0.84; 17.97]	0.7%	2.7%
comparison = Fluphenazine_Haloperidol								
Hall 1968 Fluphenazine Haloperidol	19	25	18	25	1.23	[0.35; 4.37]	1.7%	3.3%

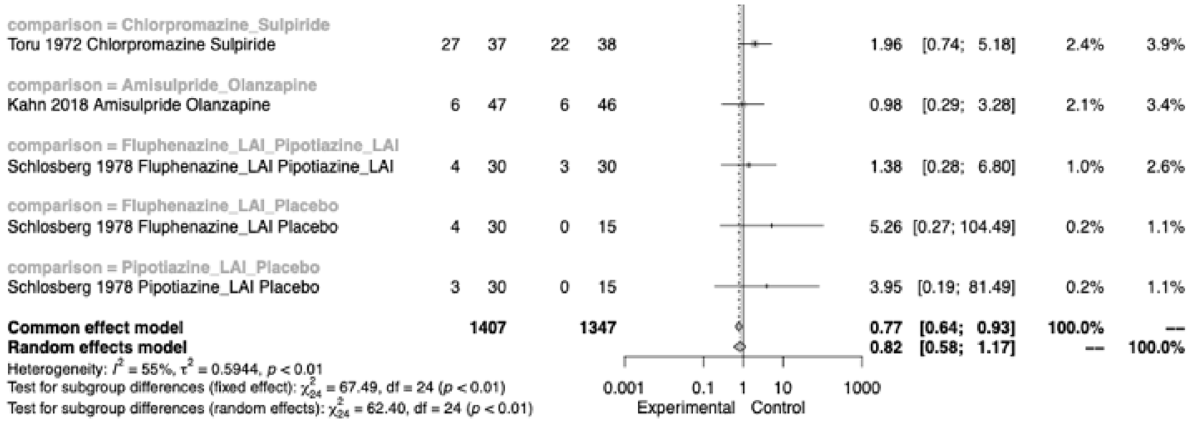

A summary effect size is calculated by pairwise meta-analyses of all studies of a specific comparison. The type of effect size measure is OR.

8.8 Prolactin levels

Network plot

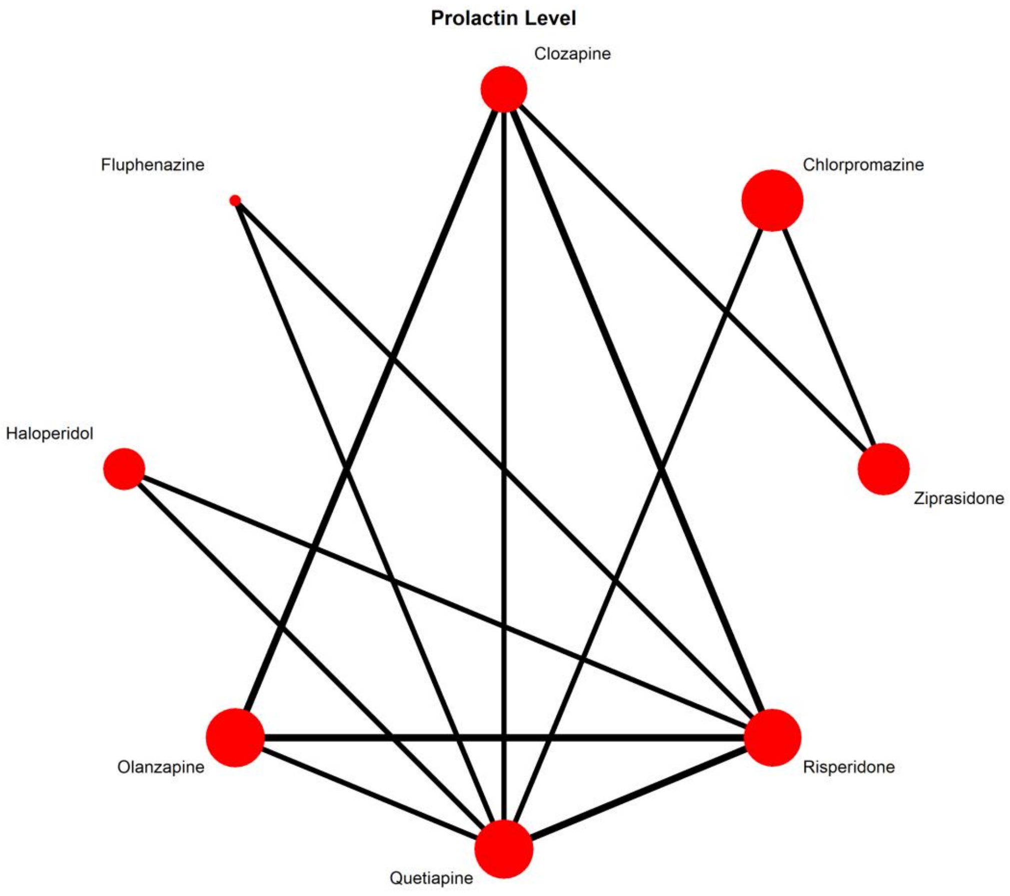

Lines link treatments with direct comparisons in trials; thickness of lines corresponds to the number of trials evaluating the comparison; size of the nodes corresponds to the number of participants assigned to the treatment.

Forest-plot of results of network meta-analysis for antipsychotic drugs versus clozapine

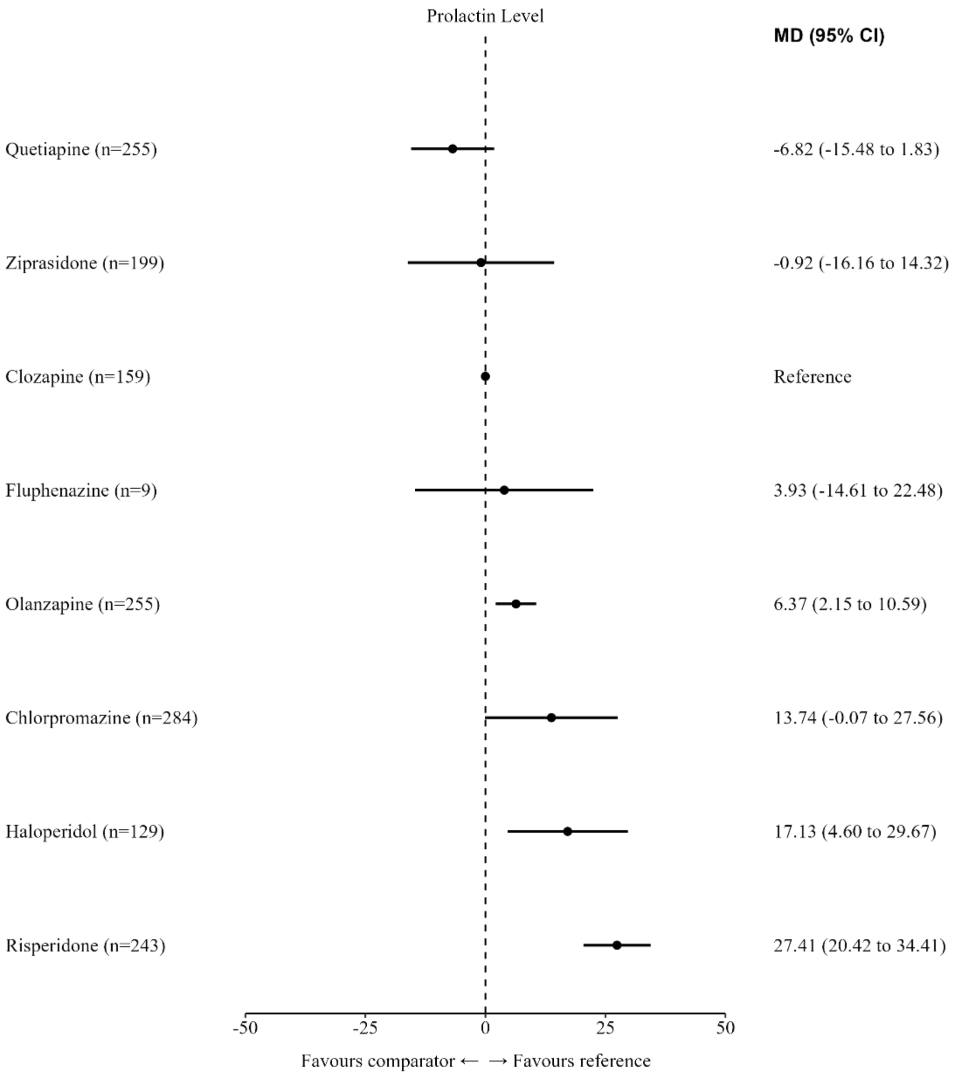

Effect sizes, measured as mean difference (MD), are from the network meta-analysis. Order of treatments is according to the mean effect size. Reference is clozapine. The direction of the effect is indicated below the x-axis.

League table for the outcome: Prolactin levels

**Quetiapine**	NA	-5.60 (-16.21 to 5.01)	-16.20 (-38.76 to 6.36)	-9.10 (-19.87 to 1.67)	**-19.70 (-33.28 to -6.12)**	**-27.40 (-43.45 to -11.35)**	**-32.90 (-44.86 to -20.95)**
-5.90 (-20.63 to 8.83)	**Ziprasidone**	1.50 (-21.21 to 24.21)	NA	NA	**-15.40 (-27.90 to -2.90)**	NA	NA
-6.82 (-15.48 to 1.83)	-0.92 (-16.16 to 14.32)	**Clozapine**	NA	**-5.38 (-9.73 to -1.04)**	NA	NA	**-28.55 (-37.64 to -19.45)**
-10.76 (-29.29 to 7.78)	-4.86 (-27.90 to 18.19)	-3.93 (-22.48 to 14.61)	**Fluphenazine**	NA	NA	NA	**-26.20 (-45.08 to -7.32)**
**-13.19 (-21.95 to -4.43)**	-7.29 (-22.80 to 8.22)	**-6.37 (-10.59 to -2.15)**	-2.43 (-20.96 to 16.10)	**Olanzapine**	NA	NA	**-16.90 (-25.00 to -8.79)**
**-20.57 (-32.74 to -8.39)**	**-14.67 (-26.08 to -3.26)**	-13.74 (-27.56 to 0.07)	-9.81 (-31.62 to 12.00)	-7.38 (-21.38 to 6.62)	**Chlorpromazine**	NA	NA
**-23.96 (-35.92 to -12.00)**	-18.06 (-36.38 to 0.27)	**-17.13 (-29.67 to -4.60)**	-13.20 (-33.80 to 7.40)	-10.77 (-23.30 to 1.76)	-3.39 (-20.07 to 13.28)	**Haloperidol**	-13.17 (-27.88 to 1.54)
**-34.24 (-43.51 to -24.96)**	**-28.34 (-44.41 to -12.26)**	**-27.41 (-34.41 to -20.42)**	**-23.48 (-41.23 to -5.73)**	**-21.05 (-27.93 to -14.16)**	-13.67 (-28.16 to 0.82)	-10.28 (-21.92 to 1.36)	**Risperidone**

Treatments are presented in order of efficacy ranking. Results of the network meta-analysis are reported in the left lower half and results of pairwise meta-analyses in the right upper half. Each cell provides the effect estimate and the corresponding 95% credible interval (95% CI) of a comparison (left lower half: treatment in column versus treatment in row; right upper half: treatment in row versus treatment in column). The type of effect size measure is mean difference (MD). Bold results indicate 95% CI excluding no effect. NA=not available.

Forest plot of results of pairwise meta-analyses

Study	Experimental Total	Experimental Mean	Experimental SD	Control Total	Control Mean	Control SD	MD	95%-CI	Weight (common)	Weight (random)
comparison = Clozapine_Risperidone										
Breier 1999 Clozapine Risperidone	13	12.20	7.7000	14	50.70	27.9000	−38.50	[−53.70; −23.30]	3.2%	5.5%
McEvoy 2006 Clozapine Risperidone	39	−7.60	13.1100	11	15.40	17.9000	−23.00	[−34.35; −11.65]	5.7%	5.9%
Common effect model	52			25			−28.55	[−37.64; −19.45]	8.9%	--
Random effects model							−29.89	[−44.99; −14.79]	--	11.4%
Heterogeneity: $I^2 = 61\%$, $\tau^2 = 73.2767$, $p = 0.11$										
comparison = Haloperidol_Risperidone										
Claus 1992 Haloperidol Risperidone	16	13.20	14.5700	18	26.37	27.8400	−13.17	[−27.88; 1.54]	3.4%	5.5%
comparison = Haloperidol_Quetiapine										
Emsley 2000 Haloperidol Quetiapine	113	−0.97	60.5600	106	−28.37	60.5600	27.40	[11.35; 43.45]	2.9%	5.4%
comparison = Clozapine_Olanzapine										
McEvoy 2006 Clozapine Olanzapine	39	−7.60	13.1100	16	−4.10	9.2000	−3.50	[−9.60; 2.60]	19.8%	6.3%
Tollefson 2001 Clozapine Olanzapine	60	−3.20	17.3000	60	4.12	17.3000	−7.32	[−13.51; −1.13]	19.3%	6.3%
Common effect model	99			76			−5.38	[−9.73; −1.04]	39.1%	--
Random effects model							−5.38	[−9.73; −1.04]	--	12.5%
Heterogeneity: $I^2 = 0\%$, $\tau^2 = 0$, $p = 0.39$										
comparison = Clozapine_Quetiapine										
McEvoy 2006 Clozapine Quetiapine	39	−7.60	13.1100	13	−13.20	18.0000	5.60	[−5.01; 16.21]	6.5%	5.9%
comparison = Olanzapine_Quetiapine										
McEvoy 2006 Olanzapine Quetiapine	16	−4.10	9.2000	13	−13.20	18.0000	9.10	[−1.67; 19.87]	6.4%	5.9%
comparison = Olanzapine_Risperidone										
McEvoy 2006 Olanzapine Risperidone	16	−4.10	9.2000	11	15.40	17.9000	−19.50	[−31.00; −8.00]	5.6%	5.9%
Kinon 2009 Olanzapine Risperidone	179	−26.46	55.8653	188	−12.14	55.8653	−14.32	[−25.75; −2.89]	5.6%	5.9%
Common effect model	195			199			−16.90	[−25.00; −8.79]	11.2%	--
Random effects model							−16.90	[−25.00; −8.79]	--	11.7%
Heterogeneity: $I^2 = 0\%$, $\tau^2 = 0$, $p = 0.53$										
comparison = Quetiapine_Risperidone										
McEvoy 2006 Quetiapine Risperidone	13	−13.20	18.0000	11	15.40	17.9000	−28.60	[−43.01; −14.19]	3.6%	5.6%
Conley 2005 Quetiapine Risperidone	6	8.20	21.8412	12	50.60	21.8412	−42.40	[−63.80; −21.00]	1.6%	4.8%
Common effect model	19			23			−32.90	[−44.86; −20.95]	5.2%	--
Random effects model							−33.14	[−45.84; −20.43]	--	10.4%
Heterogeneity: $I^2 = 9\%$, $\tau^2 = 8.5645$, $p = 0.29$										
comparison = Aripiprazole_Perphenazine										
Kane 2007a Aripiprazole Perphenazine	135	−28.20	68.7500	137	−0.30	68.7500	−27.90	[−44.24; −11.56]	2.8%	5.4%
comparison = Fluphenazine_Quetiapine										
Conley 2005 Fluphenazine Quetiapine	9	24.40	21.8412	6	8.20	21.8412	16.20	[−6.36; 38.76]	1.4%	4.7%
comparison = Fluphenazine_Risperidone										
Conley 2005 Fluphenazine Risperidone	9	24.40	21.8412	12	50.60	21.8412	−26.20	[−45.08; −7.32]	2.1%	5.1%
comparison = Chlorpromazine_Ziprasidone										
Kane 2006 Chlorpromazine Ziprasidone	154	−1.60	55.8653	153	−17.00	55.8653	15.40	[2.90; 27.90]	4.7%	5.8%
comparison = Clozapine_Ziprasidone										
Sacchetti 2009 Clozapine Ziprasidone	47	−6.50	55.8653	46	−5.00	55.8653	−1.50	[−24.21; 21.21]	1.4%	4.7%
comparison = Chlorpromazine_Quetiapine										
AstraZeneca 5077IL/0031 Chlorpromazine Quetiapine	130	−3.80	55.8653	130	−23.50	55.8653	19.70	[6.12; 33.28]	4.0%	5.7%
**Common effect model**	**1033**			**957**			**−6.55**	**[−9.27; −3.83]**	**100.0%**	**--**
**Random effects model**							**−8.28**	**[−17.60; 1.05]**	**--**	**100.0%**

Heterogeneity: $I^2 = 86\%$, $\tau^2 = 352.0106$, $p < 0.01$
Test for subgroup differences (fixed effect): $\chi^2_{13} = 119.80$, df = 13 ($p < 0.01$)
Test for subgroup differences (random effects): $\chi^2_{13} = 103.64$, df = 13 ($p < 0.01$)

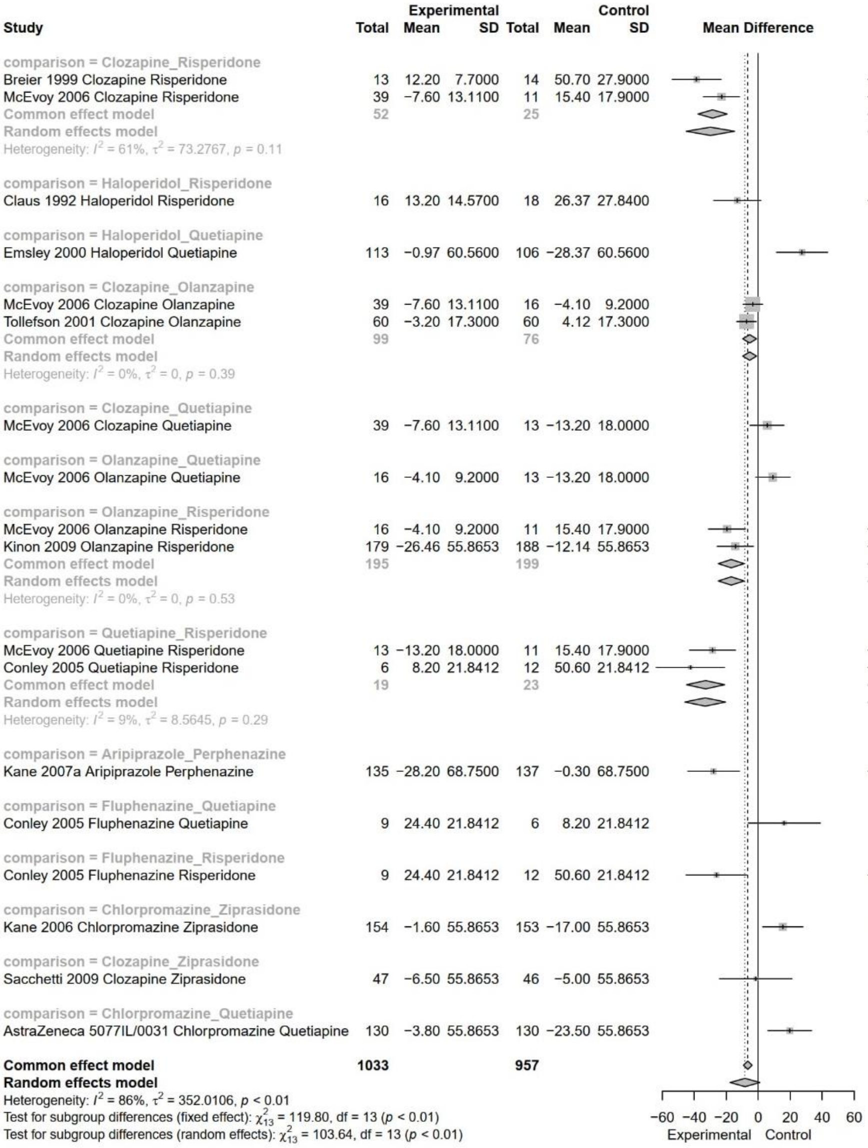

A summary effect size is calculated by pairwise meta-analyses of all studies of a specific comparison. The type of effect size measure is mean difference (MD).

8.9 Sedation

We transformed the original ORs to RRs and EERs vs. CER assuming the baseline risk of sedation with clozapine of 47% (namely CER). 47% was the average risk of sedation with clozapine across all clozapine-arms in the network meta-analysis, as estimated by a single-arm meta-analysis of proportions.

Network plot

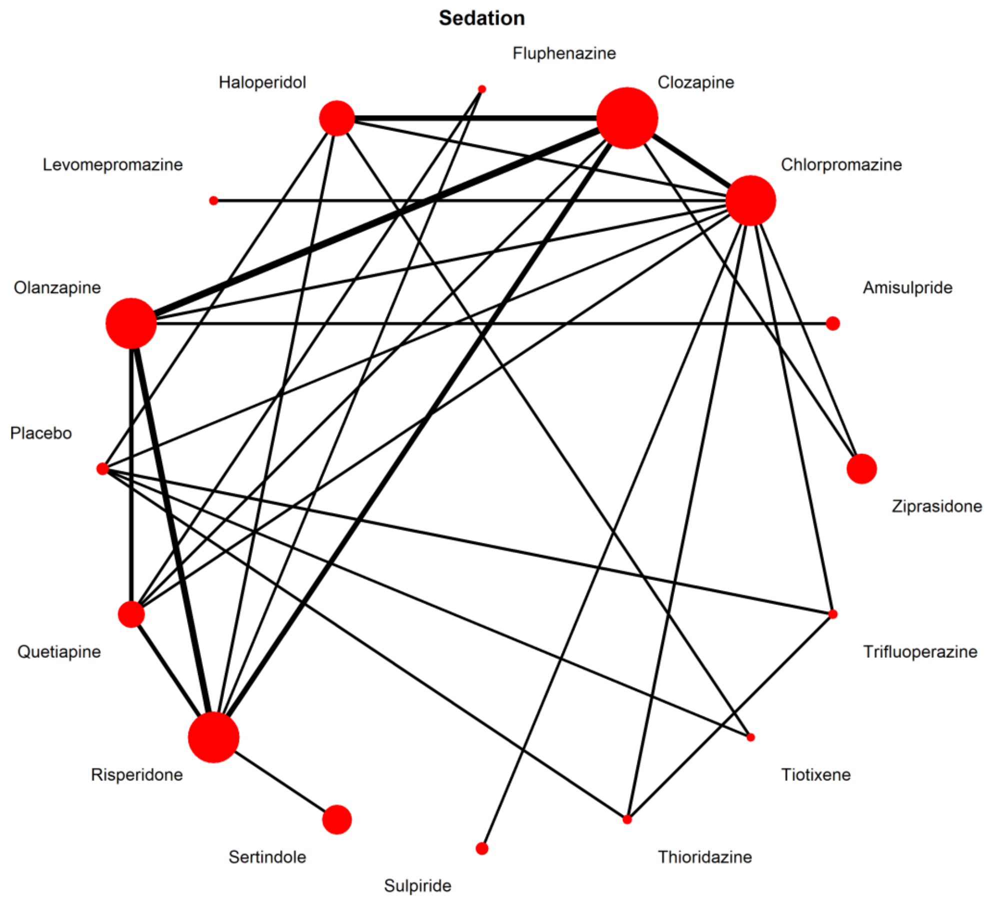

Lines link treatments with direct comparisons in trials; thickness of lines corresponds to the number of trials evaluating the comparison; size of the nodes corresponds to the number of participants assigned to the treatment.

<u>Forest-plot of results of network meta-analysis for antipsychotic drugs versus clozapine</u>

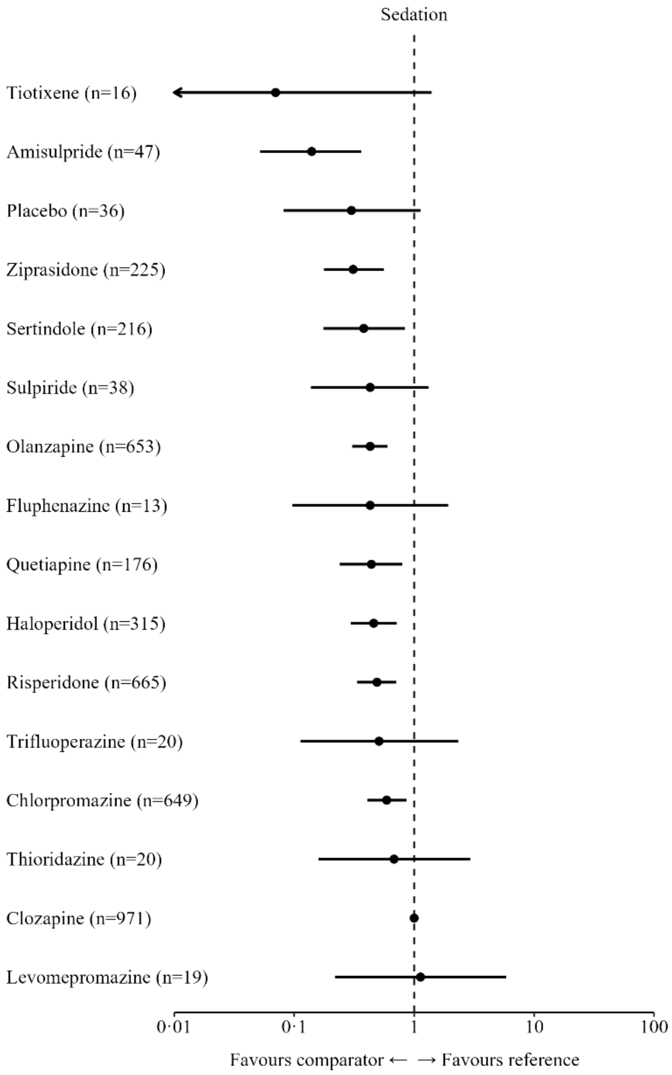

Effect sizes, measured as odds ratio (OR), are from the network meta-analysis. Order of treatments is according to the mean effect size. Reference is clozapine. The direction of the effect is indicated below the x-axis.

Forest plot with RR and EER versus CER

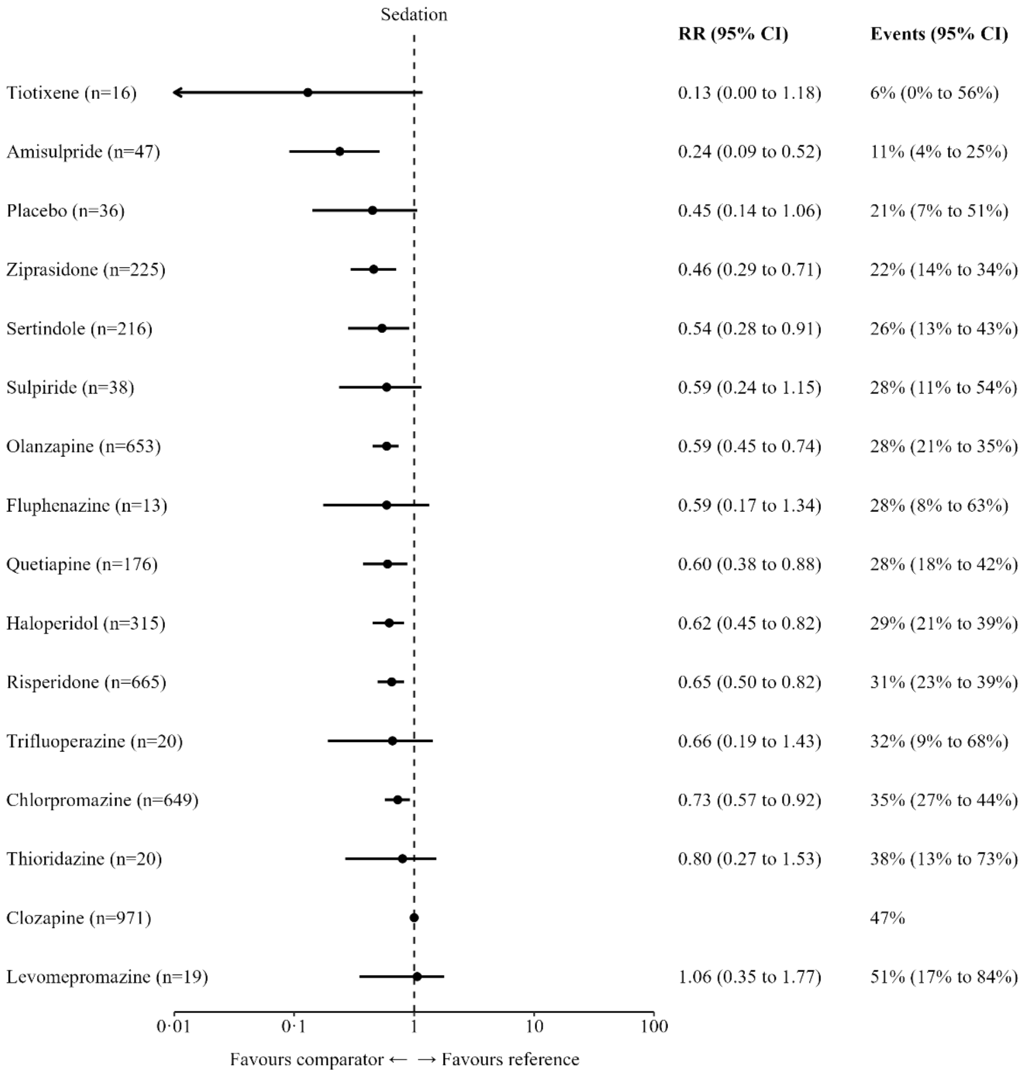

For this forest plot, we transformed the original OR to RR and exposure events rates (EER, called "events" in the forest plot) using the formula indicated above. Therefore, we used an average sedation rate with clozapine of 47% as the control event rate (CER).
Effect sizes are from the network meta-analysis. Order of treatments is according to the mean effect size. Reference is clozapine. The direction of the effect is indicated below the x-axis

League table for the outcome: Sedation (OR)

**Tiotixene**	NA	0.31 (0.01 to 8.28)	NA	NA	NA	NA	NA	NA	0.13 (0.01 to 2.64)	NA	NA	NA	NA	NA	NA
0.49 (0.02 to 11.78)	**Amisulpride**	NA	NA	NA	NA	**0.32 (0.13 to 0.80)**	NA	NA	NA	NA	NA	NA	NA	NA	NA
0.22 (0.01 to 4.99)	0.45 (0.09 to 2.30)	**Placebo**	NA	NA	NA	NA	NA	NA	0.40 (0.05 to 3.09)	NA	0.71 (0.14 to 3.66)	0.71 (0.14 to 3.66)	0.53 (0.11 to 2.60)	NA	NA
0.22 (0.01 to 4.64)	0.44 (0.14 to 1.32)	0.96 (0.24 to 3.84)	**Ziprasidone**	NA	NA	NA	NA	NA	NA	NA	NA	0.59 (0.35 to 1.01)	NA	**0.19 (0.06 to 0.61)**	NA
0.18 (0.01 to 4.01)	0.36 (0.11 to 1.21)	0.79 (0.17 to 3.62)	0.82 (0.32 to 2.14)	**Sertindole**	NA	NA	NA	NA	NA	0.79 (0.39 to 1.56)	NA	NA	NA	NA	NA
0.16 (0.01 to 3.96)	0.32 (0.07 to 1.40)	0.71 (0.13 to 3.81)	0.74 (0.23 to 2.38)	0.90 (0.23 to 3.51)	**Sulpiride**	NA	NA	NA	NA	NA	NA	0.72 (0.25 to 2.09)	NA	NA	NA
0.16 (0.01 to 3.32)	**0.32 (0.13 to 0.80)**	0.71 (0.18 to 2.72)	0.74 (0.39 to 1.39)	0.90 (0.40 to 2.00)	1.00 (0.31 to 3.18)	**Olanzapine**	NA	0.92 (0.24 to 3.58)	NA	1.02 (0.58 to 1.81)	NA	0.51 (0.21 to 1.21)	NA	**0.44 (0.29 to 0.67)**	NA
0.16 (0.01 to 4.56)	0.32 (0.05 to 1.85)	0.70 (0.10 to 5.05)	0.73 (0.15 to 3.52)	0.89 (0.17 to 4.53)	0.99 (0.16 to 6.27)	0.99 (0.22 to 4.48)	**Fluphenazine**	1.33 (0.23 to 7.74)	NA	0.71 (0.14 to 3.61)	NA	NA	NA	NA	NA
0.16 (0.01 to 3.36)	**0.31 (0.10 to 0.96)**	0.69 (0.17 to 2.81)	0.72 (0.35 to 1.49)	0.88 (0.34 to 2.28)	0.98 (0.29 to 3.24)	0.98 (0.51 to 1.86)	0.99 (0.22 to 4.44)	**Quetiapine**	NA	0.94 (0.30 to 2.99)	NA	0.72 (0.37 to 1.38)	NA	0.61 (0.18 to 2.06)	NA
0.15 (0.01 to 2.98)	**0.30 (0.10 to 0.86)**	0.66 (0.18 to 2.48)	0.68 (0.34 to 1.39)	0.83 (0.35 to 2.01)	0.93 (0.28 to 3.09)	0.93 (0.54 to 1.60)	0.94 (0.20 to 4.42)	0.95 (0.46 to 1.97)	**Haloperidol**	0.82 (0.23 to 2.85)	NA	**9.33 (1.19 to 72.99)**	NA	**0.40 (0.24 to 0.65)**	NA
0.14 (0.01 to 2.91)	**0.28 (0.10 to 0.77)**	0.62 (0.16 to 2.41)	0.65 (0.33 to 1.26)	0.79 (0.39 to 1.56)	0.88 (0.27 to 2.84)	0.88 (0.58 to 1.33)	0.89 (0.20 to 3.88)	0.90 (0.46 to 1.74)	0.94 (0.55 to 1.63)	**Risperidone**	NA	NA	NA	**0.50 (0.31 to 0.80)**	NA
0.13 (0.00 to 3.56)	0.27 (0.04 to 1.60)	0.59 (0.13 to 2.77)	0.61 (0.13 to 2.91)	0.75 (0.14 to 4.07)	0.83 (0.13 to 5.16)	0.83 (0.18 to 3.88)	0.84 (0.10 to 6.91)	0.85 (0.18 to 4.12)	0.89 (0.19 to 4.21)	0.95 (0.20 to 4.48)	**Trifluoperazine**	1.00 (0.21 to 4.71)	0.75 (0.17 to 3.33)	NA	NA
0.11 (0.01 to 2.38)	**0.23 (0.08 to 0.64)**	0.51 (0.14 to 1.88)	**0.53 (0.33 to 0.87)**	0.65 (0.28 to 1.51)	0.72 (0.25 to 2.09)	0.72 (0.46 to 1.14)	0.73 (0.16 to 3.29)	0.74 (0.42 to 1.28)	0.77 (0.44 to 1.35)	0.82 (0.50 to 1.36)	0.87 (0.20 to 3.82)	**Chlorpromazine**	0.75 (0.17 to 3.33)	0.67 (0.42 to 1.09)	0.52 (0.11 to 2.60)
0.10 (0.00 to 2.60)	0.20 (0.04 to 1.14)	0.44 (0.10 to 1.96)	0.46 (0.10 to 2.06)	0.56 (0.11 to 2.89)	0.62 (0.11 to 3.69)	0.62 (0.14 to 2.74)	0.63 (0.08 to 4.97)	0.64 (0.14 to 2.92)	0.67 (0.15 to 2.98)	0.71 (0.16 to 3.17)	0.75 (0.17 to 3.33)	0.87 (0.21 to 3.59)	**Thioridazine**	NA	NA
0.07 (0.00 to 1.40)	**0.14 (0.05 to 0.36)**	0.30 (0.08 to 1.13)	**0.31 (0.18 to 0.56)**	**0.38 (0.17 to 0.84)**	0.43 (0.14 to 1.32)	**0.43 (0.30 to 0.60)**	0.43 (0.10 to 1.92)	**0.44 (0.24 to 0.79)**	**0.46 (0.30 to 0.71)**	**0.49 (0.34 to 0.71)**	0.51 (0.11 to 2.34)	**0.59 (0.41 to 0.86)**	0.68 (0.16 to 2.94)	**Clozapine**	NA
0.06 (0.00 to 1.86)	**0.12 (0.02 to 0.81)**	0.27 (0.03 to 2.11)	0.28 (0.05 to 1.49)	0.34 (0.06 to 2.08)	0.38 (0.06 to 2.59)	0.38 (0.07 to 2.00)	0.38 (0.04 to 3.45)	0.39 (0.07 to 2.10)	0.41 (0.07 to 2.22)	0.43 (0.08 to 2.31)	0.45 (0.05 to 4.04)	0.52 (0.11 to 2.60)	0.61 (0.07 to 5.17)	0.89 (0.17 to 4.59)	**Levomepromazine**

Treatments are presented in order of efficacy ranking. Results of the network meta-analysis are reported in the left lower half and results of pairwise meta-analyses in the right upper half. Each cell provides the effect estimate and the corresponding 95% credible interval (95% CI) of a comparison (left lower half: treatment in column versus treatment in row; right upper half: treatment in row versus treatment in column). The type of effect size measure is odd ratio (OR). Bold results indicate 95% CI excluding no effect. NA=not available.

__League table for the outcome: Sedation (RR)__

**Tiotixene**	NA	0.53 (0.02 to 1.55)	NA	NA	NA	NA	NA	NA	0.33 (0.03 to 1.24)	NA	NA	NA	NA	NA	NA
0.62 (0.03 to 2.21)	**Amisulpride**	NA	NA	NA	NA	**0.59 (0.32 to 0.93)**	NA	NA	NA	NA	NA	NA	NA	NA	NA
0.41 (0.02 to 1.47)	0.67 (0.2 to 1.29)	**Placebo**	NA	NA	NA	NA	NA	NA	0.68 (0.15 to 1.26)	NA	0.9 (0.36 to 1.26)	0.91 (0.39 to 1.23)	0.83 (0.35 to 1.16)	NA	NA
0.42 (0.02 to 1.45)	0.67 (0.29 to 1.11)	0.98 (0.44 to 1.41)	**Ziprasidone**	NA	NA	NA	NA	NA	NA	NA	NA	0.85 (0.68 to 1)	NA	**0.58 (0.27 to 0.9)**	NA
0.39 (0.03 to 1.35)	0.62 (0.26 to 1.06)	0.92 (0.37 to 1.34)	0.93 (0.57 to 1.23)	**Sertindole**	NA	NA	NA	NA	NA	0.93 (0.68 to 1.12)	NA	NA	NA	NA	NA
0.37 (0.03 to 1.32)	0.59 (0.19 to 1.1)	0.88 (0.32 to 1.31)	0.9 (0.48 to 1.23)	0.97 (0.48 to 1.3)	**Sulpiride**	NA	NA	NA	NA	NA	NA	0.91 (0.57 to 1.15)	NA	NA	NA
0.37 (0.03 to 1.29)	**0.59 (0.32 to 0.93)**	0.88 (0.4 to 1.26)	0.9 (0.66 to 1.1)	0.97 (0.67 to 1.19)	1 (0.58 to 1.29)	**Olanzapine**	NA	0.97 (0.5 to 1.3)	NA	1.01 (0.82 to 1.15)	NA	0.8 (0.51 to 1.05)	NA	**0.82 (0.71 to 0.92)**	NA
0.37 (0.03 to 1.33)	0.59 (0.14 to 1.17)	0.88 (0.26 to 1.35)	0.89 (0.35 to 1.3)	0.96 (0.39 to 1.33)	1 (0.37 to 1.37)	1 (0.47 to 1.33)	**Fluphenazine**	1.09 (0.48 to 1.38)	NA	0.89 (0.36 to 1.27)	NA	NA	NA	NA	NA
0.37 (0.03 to 1.29)	**0.58 (0.26 to 0.99)**	0.87 (0.39 to 1.26)	0.89 (0.63 to 1.12)	0.96 (0.62 to 1.22)	0.99 (0.56 to 1.28)	0.99 (0.77 to 1.17)	1 (0.47 to 1.33)	**Quetiapine**	NA	0.98 (0.59 to 1.24)	NA	0.91 (0.7 to 1.08)	NA	0.9 (0.56 to 1.1)	NA
0.36 (0.03 to 1.26)	**0.58 (0.27 to 0.95)**	0.86 (0.42 to 1.23)	0.87 (0.63 to 1.09)	0.94 (0.64 to 1.18)	0.98 (0.56 to 1.26)	0.98 (0.79 to 1.13)	0.98 (0.45 to 1.31)	0.98 (0.73 to 1.18)	**Haloperidol**	0.94 (0.5 to 1.24)	NA	**1.3 (1.04 to 1.34)**	NA	**0.8 (0.65 to 0.92)**	NA
0.36 (0.03 to 1.24)	**0.57 (0.27 to 0.92)**	0.85 (0.39 to 1.21)	0.86 (0.63 to 1.06)	0.93 (0.68 to 1.12)	0.96 (0.56 to 1.24)	0.96 (0.82 to 1.08)	0.96 (0.46 to 1.28)	0.97 (0.74 to 1.14)	0.98 (0.81 to 1.13)	**Risperidone**	NA	NA	NA	**0.85 (0.73 to 0.96)**	NA
0.35 (0 to 1.26)	0.57 (0.13 to 1.12)	0.84 (0.35 to 1.22)	0.85 (0.35 to 1.23)	0.91 (0.36 to 1.27)	0.95 (0.35 to 1.3)	0.95 (0.44 to 1.27)	0.95 (0.28 to 1.32)	0.95 (0.44 to 1.27)	0.97 (0.45 to 1.28)	0.99 (0.47 to 1.28)	**Trifluoperazine**	1 (0.51 to 1.25)	0.93 (0.47 to 1.19)	NA	NA
0.33 (0.04 to 1.17)	**0.54 (0.25 to 0.87)**	0.8 (0.39 to 1.14)	**0.82 (0.66 to 0.96)**	0.88 (0.6 to 1.09)	0.91 (0.57 to 1.15)	0.91 (0.77 to 1.03)	0.91 (0.43 to 1.22)	0.92 (0.74 to 1.06)	0.93 (0.75 to 1.07)	0.95 (0.8 to 1.07)	0.96 (0.49 to 1.23)	**Chlorpromazine**	0.93 (0.47 to 1.19)	0.92 (0.81 to 1.01)	0.88 (0.44 to 1.1)
0.33 (0 to 1.16)	0.52 (0.15 to 1.03)	0.77 (0.33 to 1.13)	0.79 (0.33 to 1.13)	0.85 (0.35 to 1.18)	0.88 (0.35 to 1.2)	0.88 (0.41 to 1.17)	0.88 (0.27 to 1.22)	0.89 (0.41 to 1.18)	0.9 (0.43 to 1.18)	0.91 (0.45 to 1.19)	0.93 (0.47 to 1.19)	0.97 (0.54 to 1.2)	**Thioridazine**	NA	NA
0.31 (0 to 1.05)	**0.49 (0.24 to 0.77)**	0.72 (0.34 to 1.02)	**0.73 (0.56 to 0.88)**	**0.78 (0.55 to 0.97)**	0.82 (0.49 to 1.04)	**0.82 (0.72 to 0.9)**	0.82 (0.4 to 1.09)	**0.82 (0.65 to 0.96)**	**0.83 (0.72 to 0.94)**	**0.85 (0.75 to 0.94)**	0.86 (0.42 to 1.11)	**0.89 (0.8 to 0.97)**	0.93 (0.53 to 1.13)	**Clozapine**	NA
0.29 (0 to 1.08)	**0.47 (0.12 to 0.97)**	0.71 (0.17 to 1.09)	0.72 (0.25 to 1.05)	0.77 (0.29 to 1.09)	0.8 (0.29 to 1.1)	0.8 (0.33 to 1.08)	0.8 (0.21 to 1.12)	0.81 (0.33 to 1.09)	0.82 (0.33 to 1.09)	0.83 (0.36 to 1.1)	0.84 (0.25 to 1.13)	0.88 (0.44 to 1.1)	0.91 (0.33 to 1.14)	0.98 (0.57 to 1.14)	**Levomepromazine**

The original results given in OR (and their 95% CI) are transformed to RR (left lower half: treatment in column versus treatment in row; right upper half: treatment in row versus treatment in column) using the formula described above. For this transformation, we assumed a sedation rate with clozapine of 47% as the control event rate (CER) for all comparisons of active antipsychotic versus clozapine.
Treatments are presented in order of efficacy ranking. Results of the network meta-analysis are reported in the left lower half and results of pairwise meta-analyses in the right upper half. Bold results indicate 95% CI excluding no effect. NA=not available.

Forest plot of results of pairwise meta-analyses

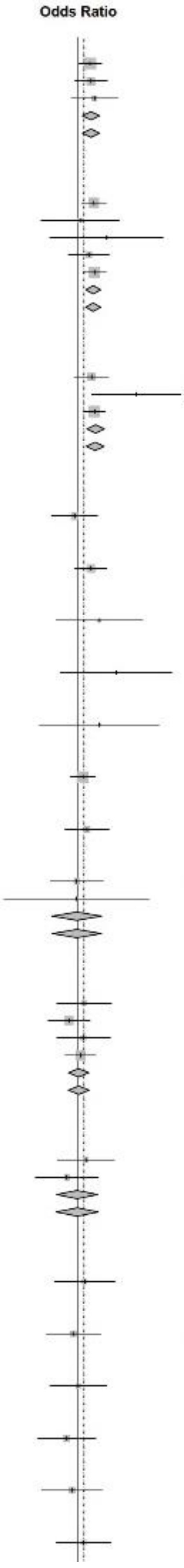

Study	Experimental Events	Experimental Total	Control Events	Control Total	OR	95%-CI	Weight (common)	Weight (random)
comparison = Clozapine_Risperidone								
Azorin 2001 Clozapine Risperidone	33	138	19	135	1.92	[1.03; 3.58]	5.0%	4.5%
Bondolfi 1998 Clozapine Risperidone	20	43	13	43	2.01	[0.83; 4.86]	2.4%	3.2%
McEvoy 2006 Clozapine Risperidone	22	49	4	16	2.44	[0.69; 8.65]	1.1%	2.0%
Common effect model		230		194	2.01	[1.26; 3.23]	8.5%	--
Random effects model					2.01	[1.25; 3.22]	--	9.8%
Heterogeneity: $I^2 = 0\%$, $\tau^2 = 0$, $p = 0.94$								
comparison = Clozapine_Olanzapine								
Bitter 2004 Clozapine Olanzapine	41	72	27	75	2.35	[1.21; 4.56]	3.9%	4.3%
Shaw 2006 Clozapine Olanzapine	2	12	2	13	1.10	[0.13; 9.34]	0.5%	0.9%
Kumra 2007 Clozapine Olanzapine	18	18	19	21	4.74	[0.21; 105.54]	0.2%	0.4%
McEvoy 2006 Clozapine Olanzapine	22	49	6	19	1.77	[0.58; 5.41]	1.6%	2.4%
Tollefson 2001 Clozapine Olanzapine	41	90	23	90	2.44	[1.30; 4.57]	4.3%	4.5%
Common effect model		241		218	2.27	[1.50; 3.42]	10.5%	--
Random effects model					2.26	[1.50; 3.41]	--	12.5%
Heterogeneity: $I^2 = 0\%$, $\tau^2 = 0$, $p = 0.92$								
comparison = Clozapine_Haloperidol								
Buchanan 1998 Clozapine Haloperidol	20	38	13	37	2.05	[0.81; 5.19]	2.1%	3.0%
Kumra 1996 Clozapine Haloperidol	9	10	3	11	24.00	[2.06; 279.62]	0.1%	0.7%
Rosenheck 1997 Clozapine Haloperidol	187	205	177	218	2.41	[1.33; 4.35]	5.1%	4.7%
Common effect model		253		266	2.59	[1.60; 4.19]	7.4%	--
Random effects model					2.52	[1.55; 4.11]	--	8.4%
Heterogeneity: $I^2 = 42\%$, $\tau^2 = < 0.0001$, $p = 0.18$								
comparison = Haloperidol_Risperidone								
Claus 1992 Haloperidol Risperidone	14	22	15	22	0.82	[0.23; 2.85]	1.9%	2.1%
comparison = Chlorpromazine_Olanzapine								
Conley 1998 Chlorpromazine Olanzapine	22	42	15	42	1.98	[0.83; 4.75]	2.4%	3.3%
comparison = Haloperidol_Placebo								
Howard 1974 Haloperidol Placebo	3	17	1	16	3.21	[0.30; 34.64]	0.3%	0.7%
comparison = Haloperidol_Tiotixene								
Howard 1974 Haloperidol Tiotixene	3	17	0	16	7.97	[0.38; 167.53]	0.1%	0.4%
comparison = Placebo_Tiotixene								
Howard 1974 Placebo Tiotixene	1	16	0	16	3.19	[0.12; 84.43]	0.2%	0.4%
comparison = Risperidone_Sertindole								
Kane 2010b Risperidone Sertindole	15	105	25	216	1.27	[0.64; 2.53]	4.8%	4.2%
comparison = Clozapine_Quetiapine								
McEvoy 2006 Clozapine Quetiapine	22	49	5	15	1.63	[0.48; 5.48]	1.4%	2.1%
comparison = Olanzapine_Quetiapine								
McEvoy 2006 Olanzapine Quetiapine	6	19	5	15	0.92	[0.22; 3.92]	1.3%	1.6%
Sirota 2006 Olanzapine Quetiapine	0	21	0	19	0.91	[0.02; 47.94]	0.2%	0.3%
Common effect model		40		34	0.92	[0.24; 3.58]	1.5%	--
Random effects model					0.92	[0.24; 3.58]	--	1.9%
Heterogeneity: $I^2 = 0\%$, $\tau^2 = 0$, $p = 0.99$								
comparison = Olanzapine_Risperidone								
McEvoy 2006 Olanzapine Risperidone	6	19	4	16	1.38	[0.31; 6.14]	1.0%	1.6%
Alvarez 2006 Olanzapine Risperidone	5	124	8	123	0.60	[0.19; 1.90]	2.6%	2.3%
Chen 2012 Olanzapine Risperidone	11	16	10	16	1.32	[0.31; 5.70]	1.1%	1.6%
Kinon 2009 Olanzapine Risperidone	12	186	11	192	1.13	[0.49; 2.64]	3.5%	3.4%
Common effect model		345		347	1.02	[0.58; 1.79]	8.2%	--
Random effects model					1.02	[0.58; 1.81]	--	8.9%
Heterogeneity: $I^2 = 0\%$, $\tau^2 = 0$, $p = 0.77$								
comparison = Quetiapine_Risperidone								
McEvoy 2006 Quetiapine Risperidone	5	15	4	16	1.50	[0.32; 7.14]	0.9%	1.5%
Conley 2005 Quetiapine Risperidone	3	12	5	13	0.53	[0.10; 2.98]	1.2%	1.2%
Common effect model		27		29	0.94	[0.30; 2.91]	2.1%	--
Random effects model					0.94	[0.30; 2.99]	--	2.7%
Heterogeneity: $I^2 = 0\%$, $\tau^2 = 0$, $p = 0.38$								
comparison = Chlorpromazine_Placebo								
Schiele 1961, 06602 Chlorpromazine Placebo	4	20	3	20	1.42	[0.27; 7.34]	0.8%	1.3%
comparison = Chlorpromazine_Thioridazine								
Schiele 1961, 06602 Chlorpromazine Thioridazine	4	20	5	20	0.75	[0.17; 3.33]	1.4%	1.6%
comparison = Chlorpromazine_Trifluoperazine								
Schiele 1961, 06602 Chlorpromazine Trifluoperazine	4	20	4	20	1.00	[0.21; 4.71]	1.1%	1.5%
comparison = Placebo_Thioridazine								
Schiele 1961, 06602 Placebo Thioridazine	3	20	5	20	0.53	[0.11; 2.60]	1.5%	1.4%
comparison = Placebo_Trifluoperazine								
Schiele 1961, 06602 Placebo Trifluoperazine	3	20	4	20	0.71	[0.14; 3.66]	1.2%	1.3%
comparison = Thioridazine_Trifluoperazine								
Schiele 1961, 06602 Thioridazine Trifluoperazine	5	20	4	20	1.33	[0.30; 5.93]	1.0%	1.6%

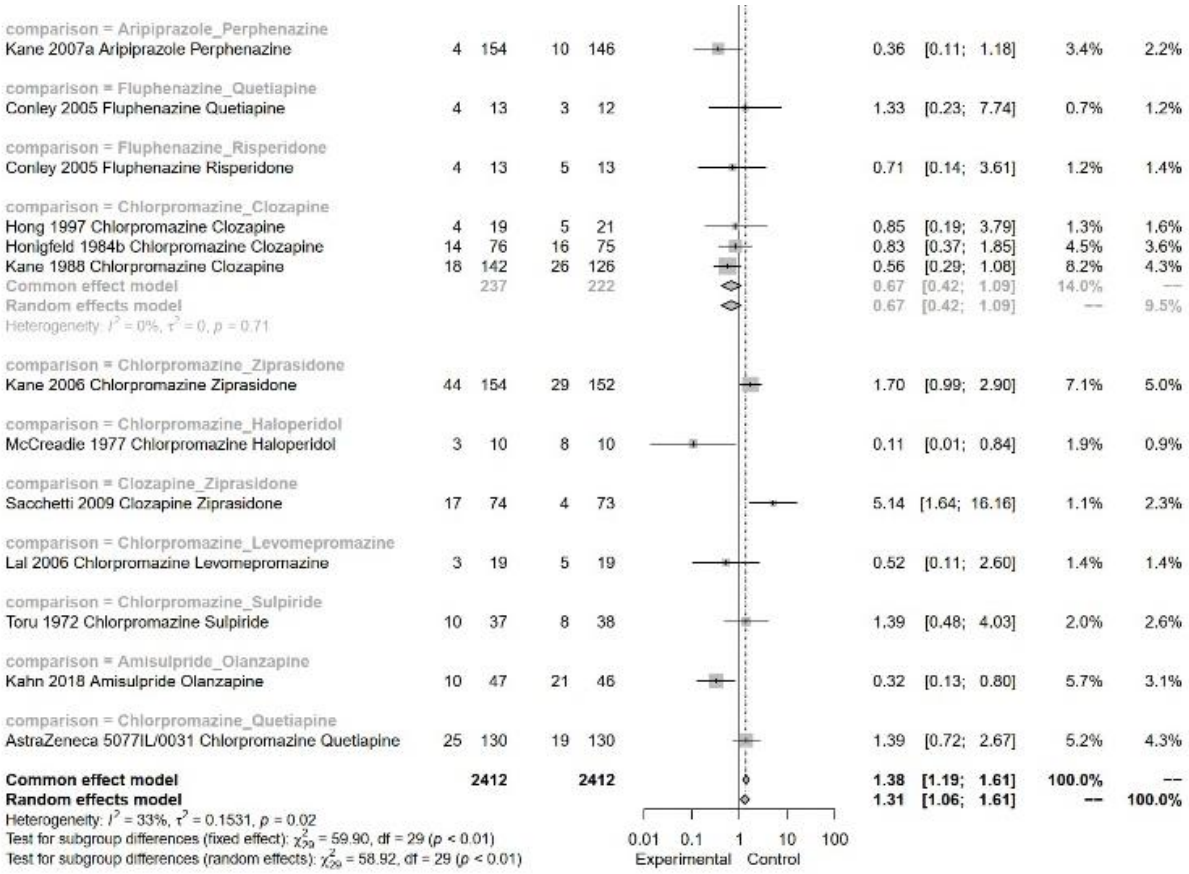

A summary effect size is calculated by pairwise meta-analyses of all studies of a specific comparison. The type of effect size measure is OR.

8.10 Weight gain

Network plot

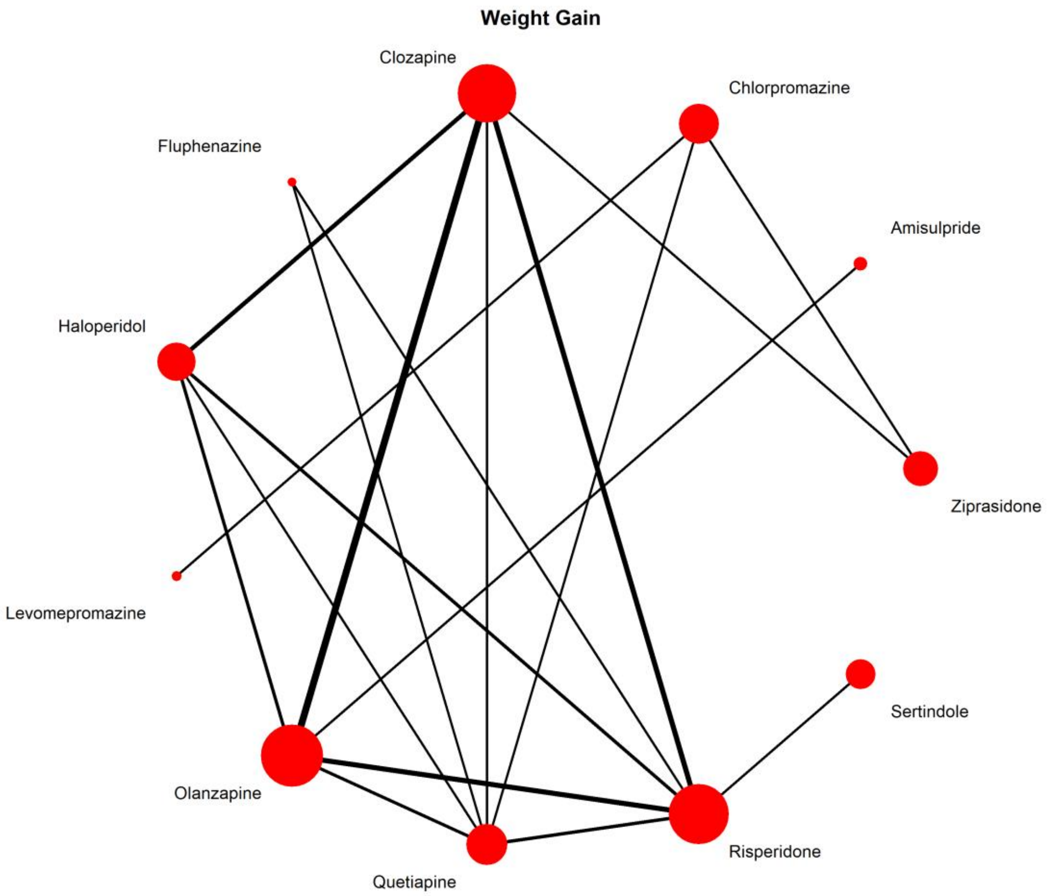

Lines link treatments with direct comparisons in trials; thickness of lines corresponds to the number of trials evaluating the comparison; size of the nodes corresponds to the number of participants assigned to the treatment.

Forest-plot of results of network meta-analysis for antipsychotic drugs versus clozapine

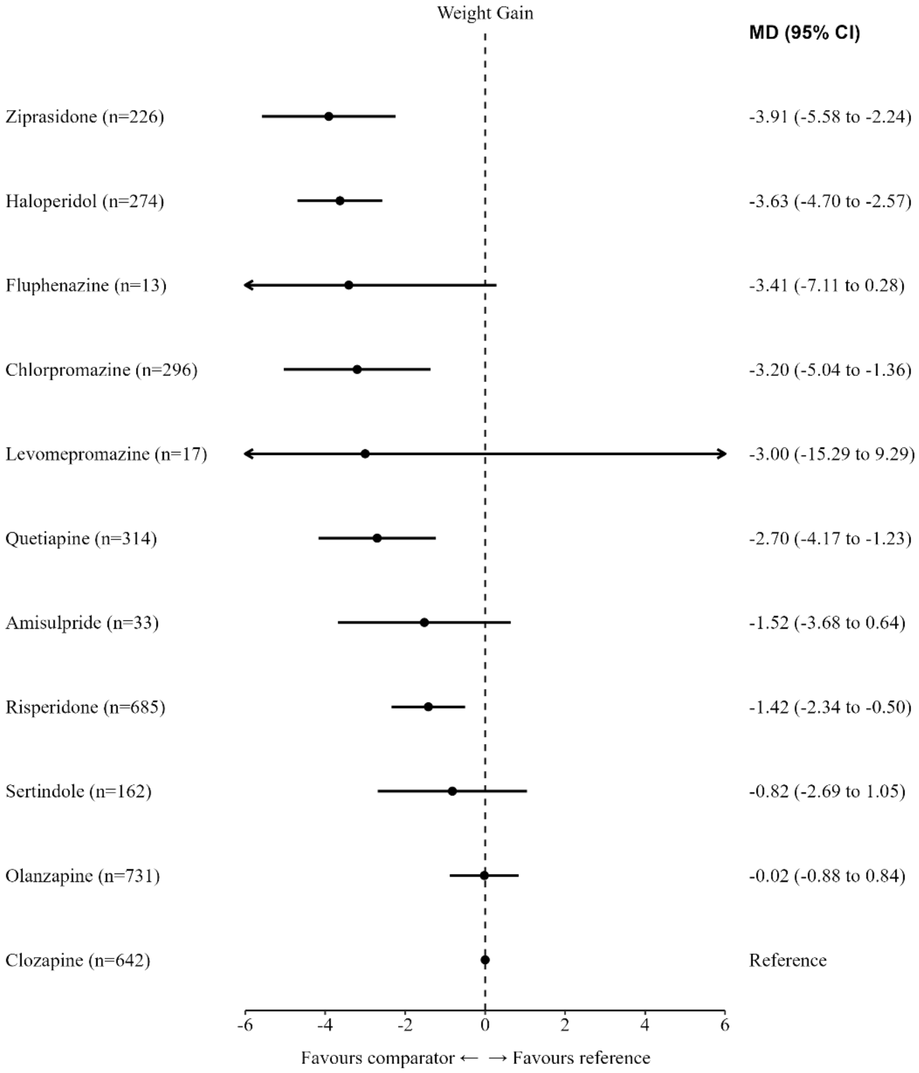

Effect sizes, measured as mean difference (MD), are from the network meta-analysis. Order of treatments is according to the mean effect size. Reference is clozapine. The direction of the effect is indicated below the x-axis.

League table for the outcome: Weight gain

**Ziprasidone**	NA	NA	-1.09 (-2.91 to 0.73)	NA	NA	NA	NA	NA	NA	**-3.46 (-5.45 to -1.47)**
-0.28 (-2.13 to 1.58)	**Haloperidol**	NA	NA	-0.70 (-2.56 to 1.16)	NA	NA	**-1.99 (-3.52 to -0.45)**	NA	**-5.06 (-6.97 to -3.14)**	**-3.55 (-4.92 to -2.17)**
-0.50 (-4.51 to 3.52)	-0.22 (-3.97 to 3.52)	**Fluphenazine**	NA	-1.40 (-8.58 to 5.78)	NA	NA	-1.95 (-5.56 to 1.66)	NA	NA	NA
-0.71 (-2.29 to 0.87)	-0.44 (-2.34 to 1.47)	-0.22 (-4.27 to 3.84)	**Chlorpromazine**	-0.91 (-2.81 to 0.99)	-0.20 (-12.35 to 11.95)	NA	NA	NA	NA	NA
-1.21 (-3.05 to 0.63)	-0.93 (-2.33 to 0.46)	-0.71 (-4.57 to 3.15)	-0.50 (-2.13 to 1.13)	**Quetiapine**	NA	NA	-0.99 (-5.14 to 3.16)	NA	**-3.11 (-5.60 to -0.62)**	-0.90 (-8.14 to 6.34)
-0.91 (-13.17 to 11.34)	-0.64 (-12.94 to 11.66)	-0.42 (-13.22 to 12.39)	-0.20 (-12.35 to 11.95)	0.30 (-11.96 to 12.56)	**Levomepromazine**	NA	NA	NA	NA	NA
-2.39 (-5.07 to 0.29)	-2.11 (-4.39 to 0.17)	-1.89 (-6.08 to 2.29)	-1.68 (-4.42 to 1.07)	-1.18 (-3.64 to 1.29)	-1.48 (-13.93 to 10.98)	**Amisulpride**	NA	NA	-1.50 (-3.48 to 0.48)	NA
**-2.49 (-4.33 to -0.65)**	**-2.21 (-3.33 to -1.10)**	-1.99 (-5.58 to 1.60)	-1.78 (-3.71 to 0.16)	-1.28 (-2.79 to 0.23)	-1.58 (-13.88 to 10.73)	-0.10 (-2.27 to 2.06)	**Risperidone**	-0.60 (-2.23 to 1.03)	**-1.26 (-2.28 to -0.24)**	**-1.86 (-3.06 to -0.66)**
**-3.09 (-5.54 to -0.63)**	**-2.81 (-4.78 to -0.84)**	-2.59 (-6.53 to 1.35)	-2.38 (-4.90 to 0.15)	-1.88 (-4.10 to 0.34)	-2.18 (-14.59 to 10.23)	-0.70 (-3.41 to 2.00)	-0.60 (-2.23 to 1.03)	**Sertindole**	NA	NA
**-3.89 (-5.70 to -2.08)**	**-3.61 (-4.73 to -2.49)**	-3.39 (-7.08 to 0.29)	**-3.18 (-5.08 to -1.27)**	**-2.68 (-4.14 to -1.21)**	-2.98 (-15.28 to 9.32)	-1.50 (-3.48 to 0.48)	**-1.40 (-2.27 to -0.53)**	-0.80 (-2.64 to 1.05)	**Olanzapine**	0.20 (-0.83 to 1.22)
**-3.91 (-5.58 to -2.24)**	**-3.63 (-4.70 to -2.57)**	-3.41 (-7.11 to 0.28)	**-3.20 (-5.04 to -1.36)**	**-2.70 (-4.17 to -1.23)**	-3.00 (-15.29 to 9.29)	-1.52 (-3.68 to 0.64)	**-1.42 (-2.34 to -0.50)**	-0.82 (-2.69 to 1.05)	-0.02 (-0.88 to 0.84)	**Clozapine**

Treatments are presented in order of efficacy ranking. Results of the network meta-analysis are reported in the left lower half and results of pairwise meta-analyses in the right upper half. Each cell provides the effect estimate and the corresponding 95% credible interval (95% CI) of a comparison (left lower half: treatment in column versus treatment in row; right upper half: treatment in row versus treatment in column). The type of effect size measure is mean difference (MD). Bold results indicate 95% CI excluding no effect. NA=not available.

Forest plot of results of pairwise meta-analyses

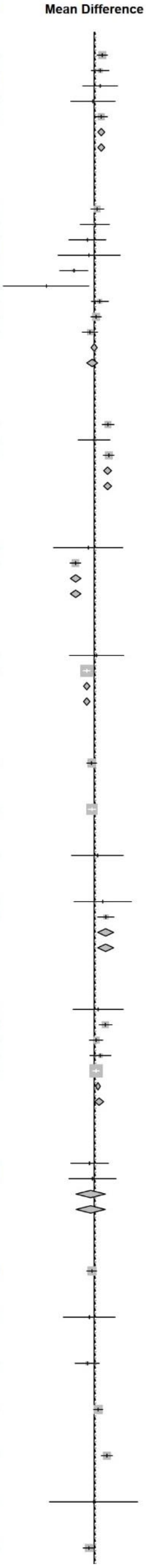

Study	Experimental Total	Experimental Mean	Experimental SD	Control Total	Control Mean	Control SD	MD	95%-CI	Weight (common)	Weight (random)
comparison = Clozapine_Risperidone										
Azorin 2001 Clozapine Risperidone	136	2.40	5.7600	134	0.20	5.7600	2.20	[0.83; 3.57]	4.4%	3.6%
Bondolfi 1998 Clozapine Risperidone	43	2.70	5.7269	43	1.10	5.7269	1.60	[−0.82; 4.02]	1.4%	2.9%
Daniel 1996 Clozapine Risperidone	10	80.56	5.8100	10	78.97	5.1200	1.59	[−3.21; 6.39]	0.4%	1.6%
McEvoy 2006 Clozapine Risperidone	41	1.40	17.9300	11	1.77	4.2100	−0.37	[−6.40; 5.66]	0.2%	1.2%
Volavka 2002 Clozapine Risperidone	38	4.20	4.7000	39	2.30	2.8000	1.90	[0.17; 3.63]	2.8%	3.4%
Common effect model	268			237			1.93	[0.98; 2.88]	9.2%	--
Random effects model							1.93	[0.98; 2.88]	--	12.7%
Heterogeneity: I^2 = 0%, τ^2 = 0, p = 0.94										
comparison = Clozapine_Olanzapine										
Bitter 2004 Clozapine Olanzapine	72	4.10	5.6000	75	3.30	5.3000	0.80	[−0.96; 2.56]	2.7%	3.3%
Shaw 2006 Clozapine Olanzapine	12	3.80	6.0000	13	3.60	4.0000	0.20	[−3.83; 4.23]	0.5%	1.9%
Conley 2003 Clozapine Olanzapine	5	1.54	4.0000	8	3.40	5.3500	−1.86	[−6.96; 3.24]	0.3%	1.5%
McEvoy 2006 Clozapine Olanzapine	41	1.40	17.9300	16	2.81	13.2300	−1.41	[−9.90; 7.08]	0.1%	0.7%
Meltzer 2008 Clozapine Olanzapine	21	92.80	6.8600	19	98.40	5.7300	−5.60	[−9.50; −1.70]	0.5%	2.0%
Moresco 2004 Clozapine Olanzapine	6	66.80	9.4000	9	80.00	13.9000	−13.20	[−24.99; −1.41]	0.1%	0.4%
Naber 2005 Clozapine Olanzapine	57	5.00	6.8000	57	3.50	5.9000	1.50	[−0.84; 3.84]	1.5%	3.0%
Tollefson 2001 Clozapine Olanzapine	90	2.30	4.9000	90	1.80	5.0000	0.50	[−0.95; 1.95]	4.0%	3.5%
Volavka 2002 Clozapine Olanzapine	38	4.20	4.7000	38	5.40	4.6000	−1.20	[−3.29; 0.89]	1.9%	3.1%
Common effect model	342			325			−0.03	[−0.88; 0.81]	11.7%	--
Random effects model							−0.57	[−2.06; 0.92]	--	19.5%
Heterogeneity: I^2 = 54%, τ^2 = 2.2854, p = 0.03										
comparison = Clozapine_Haloperidol										
Buchanan 1998 Clozapine Haloperidol	28	4.20	3.4000	33	0.40	3.2000	3.80	[2.13; 5.47]	3.0%	3.4%
Kumra 1996 Clozapine Haloperidol	10	0.90	6.4700	11	0.94	2.8900	−0.04	[−4.40; 4.32]	0.4%	1.8%
Volavka 2002 Clozapine Haloperidol	38	4.20	4.7000	36	0.20	0.2000	4.00	[2.50; 5.50]	3.7%	3.5%
Common effect model	76			80			3.67	[2.59; 4.75]	7.2%	--
Random effects model							3.67	[2.59; 4.75]	--	8.7%
Heterogeneity: I^2 = 33%, τ^2 = < 0.0001, p = 0.22										
comparison = Haloperidol_Olanzapine										
Buchanan 2005 Haloperidol Olanzapine	31	87.27	20.0000	27	88.95	16.7800	−1.68	[−11.15; 7.79]	0.1%	0.6%
Volavka 2002 Haloperidol Olanzapine	36	0.20	0.2000	38	5.40	4.6000	−5.20	[−6.66; −3.74]	3.9%	3.5%
Common effect model	67			65			−5.12	[−6.56; −3.67]	4.0%	--
Random effects model							−5.12	[−6.56; −3.67]	--	4.1%
Heterogeneity: I^2 = 0%, τ^2 = 0, p = 0.47										
comparison = Haloperidol_Risperidone										
Claus 1992 Haloperidol Risperidone	22	73.90	13.1300	22	73.20	12.2000	0.70	[−6.79; 8.19]	0.1%	0.9%
Volavka 2002 Haloperidol Risperidone	36	0.20	0.2000	39	2.30	2.8000	−2.10	[−2.98; −1.22]	10.8%	3.8%
Common effect model	58			61			−2.06	[−2.94; −1.19]	10.9%	--
Random effects model							−2.06	[−2.94; −1.19]	--	4.7%
Heterogeneity: I^2 = 0%, τ^2 = 0, p = 0.47										
comparison = Haloperidol_Quetiapine										
Emsley 2000 Haloperidol Quetiapine	141	0.70	5.7269	140	1.40	5.7269	−0.70	[−2.04; 0.64]	4.7%	3.6%
comparison = Risperidone_Sertindole										
Kane 2010b Risperidone Sertindole	86	2.50	3.3000	162	3.10	4.5000	−0.60	[−1.58; 0.38]	8.7%	3.8%
comparison = Clozapine_Quetiapine										
McEvoy 2006 Clozapine Quetiapine	41	1.40	17.9300	13	0.50	8.3400	0.90	[−6.22; 8.02]	0.2%	0.9%
comparison = Olanzapine_Quetiapine										
McEvoy 2006 Olanzapine Quetiapine	16	2.81	13.2300	13	0.50	8.3400	2.31	[−5.60; 10.22]	0.1%	0.8%
Sirota 2006 Olanzapine Quetiapine	21	2.30	3.6700	19	−0.90	3.6700	3.20	[0.92; 5.48]	1.6%	3.0%
Common effect model	37			32			3.13	[0.94; 5.32]	1.7%	--
Random effects model							3.13	[0.94; 5.32]	--	3.8%
Heterogeneity: I^2 = 0%, τ^2 = 0, p = 0.83										
comparison = Olanzapine_Risperidone										
McEvoy 2006 Olanzapine Risperidone	16	2.81	13.2300	11	1.77	4.2100	1.04	[−5.90; 7.98]	0.2%	1.0%
Volavka 2002 Olanzapine Risperidone	38	5.40	4.6000	39	2.30	2.8000	3.10	[1.39; 4.81]	2.9%	3.4%
Alvarez 2006 Olanzapine Risperidone	124	3.50	6.9000	123	2.97	7.6500	0.53	[−1.29; 2.35]	2.5%	3.3%
Chen 2012 Olanzapine Risperidone	16	2.70	4.0000	16	1.00	4.2000	1.70	[−1.14; 4.54]	1.0%	2.6%
Kinon 2009 Olanzapine Risperidone	179	1.85	3.9700	188	1.29	4.2500	0.56	[−0.28; 1.40]	11.8%	3.9%
Common effect model	373			377			1.02	[0.35; 1.69]	18.5%	--
Random effects model							1.33	[0.09; 2.58]	--	14.2%
Heterogeneity: I^2 = 46%, τ^2 = 0.9202, p = 0.12										
comparison = Quetiapine_Risperidone										
McEvoy 2006 Quetiapine Risperidone	13	0.50	8.3400	11	1.77	4.2100	−1.27	[−6.44; 3.90]	0.3%	1.5%
Conley 2005 Quetiapine Risperidone	12	−1.20	11.2200	13	−0.65	2.4300	−0.55	[−7.03; 5.93]	0.2%	1.1%
Common effect model	25			24			−0.99	[−5.03; 3.05]	0.5%	--
Random effects model							−0.99	[−5.03; 3.05]	--	2.5%
Heterogeneity: I^2 = 0%, τ^2 = 0, p = 0.86										
comparison = Aripiprazole_Perphenazine										
Kane 2007a Aripiprazole Perphenazine	154	−2.19	5.7269	146	−1.52	5.7269	−0.67	[−1.97; 0.63]	5.0%	3.6%
comparison = Fluphenazine_Quetiapine										
Conley 2005 Fluphenazine Quetiapine	13	−2.60	5.7000	12	−1.20	11.2200	−1.40	[−8.46; 5.66]	0.2%	0.9%
comparison = Fluphenazine_Risperidone										
Conley 2005 Fluphenazine Risperidone	13	−2.60	5.7000	13	−0.65	2.4300	−1.95	[−5.32; 1.42]	0.7%	2.3%
comparison = Chlorpromazine_Ziprasidone										
Kane 2006 Chlorpromazine Ziprasidone	154	1.32	5.7269	153	0.23	5.7269	1.09	[−0.19; 2.37]	5.1%	3.6%
comparison = Clozapine_Ziprasidone										
Sacchetti 2009 Clozapine Ziprasidone	73	0.84	4.6000	73	−2.62	4.7000	3.46	[1.95; 4.97]	3.7%	3.5%
comparison = Chlorpromazine_Levomepromazine										
Lal 2006 Chlorpromazine Levomepromazine	12	75.40	18.4000	17	75.60	12.9000	−0.20	[−12.28; 11.88]	0.1%	0.4%
comparison = Amisulpride_Olanzapine										
Kahn 2018 Amisulpride Olanzapine	33	2.70	2.9000	39	4.20	3.6000	−1.50	[−3.00; 0.00]	3.7%	3.5%

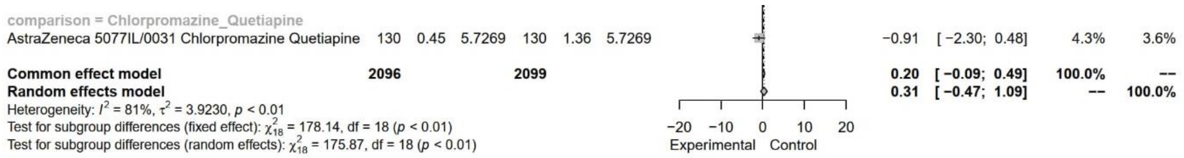

A summary effect size is calculated by pairwise meta-analyses of all studies of a specific comparison. The type of effect size measure is mean difference (MD).

8.11 QTc prolongation

Forest plot of results of pairwise meta-analyses

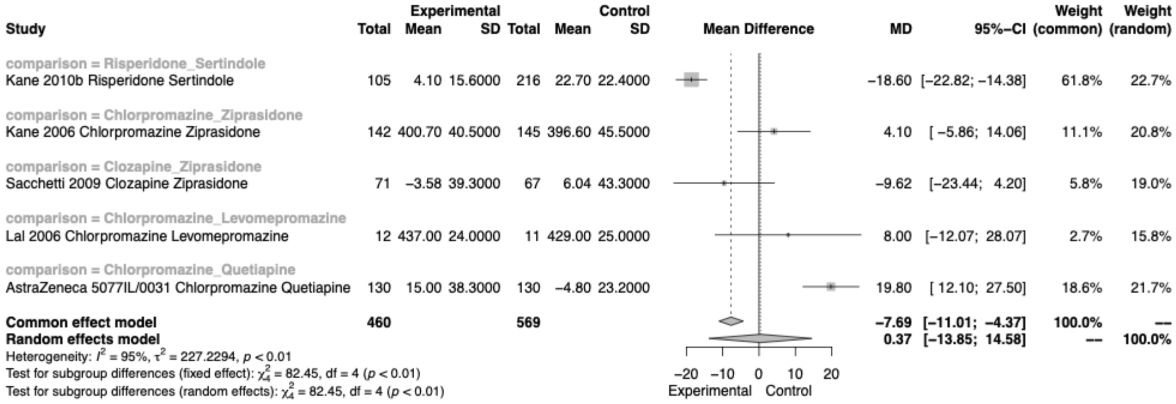

A summary effect size is calculated by pairwise meta-analyses of all studies of a specific comparison. The type of effect size measure is mean difference (MD).

8.12 Functioning

Network plot

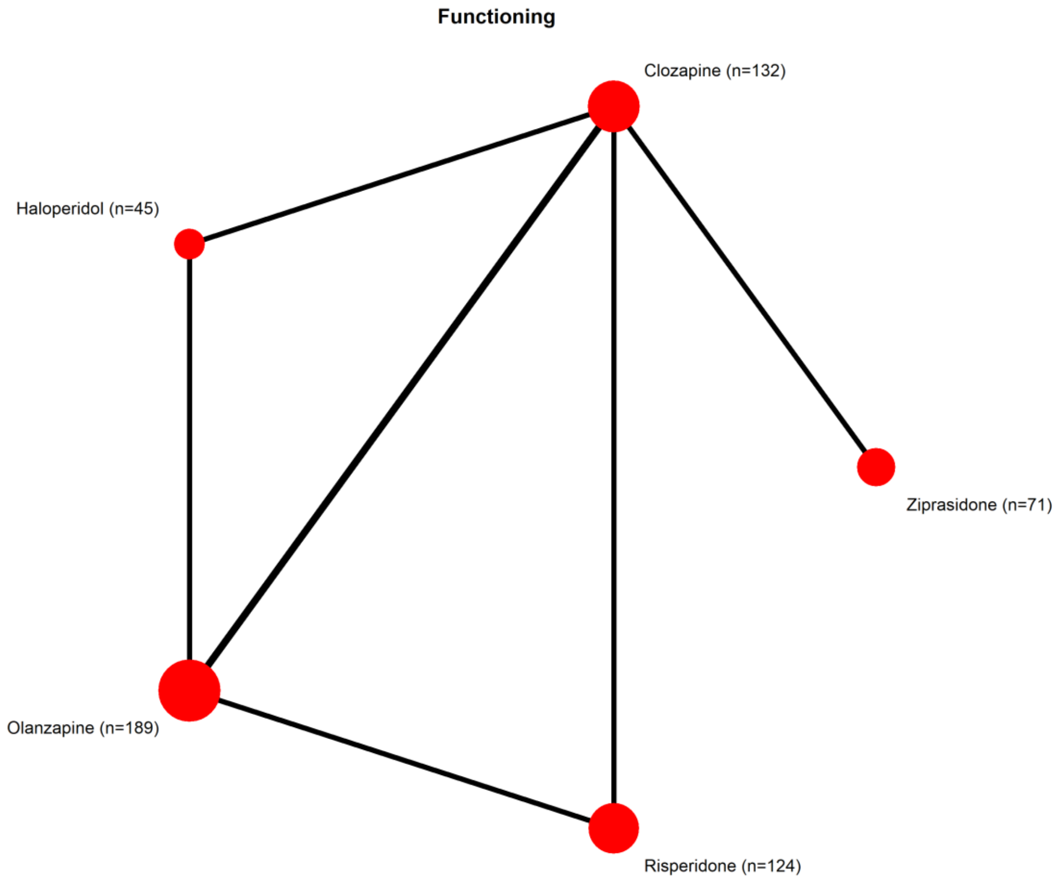

Lines link treatments with direct comparisons in trials; thickness of lines corresponds to the number of trials evaluating the comparison; size of the nodes corresponds to the number of participants assigned to the treatment.

Forest-plot of results of network meta-analysis for antipsychotic drugs versus clozapine

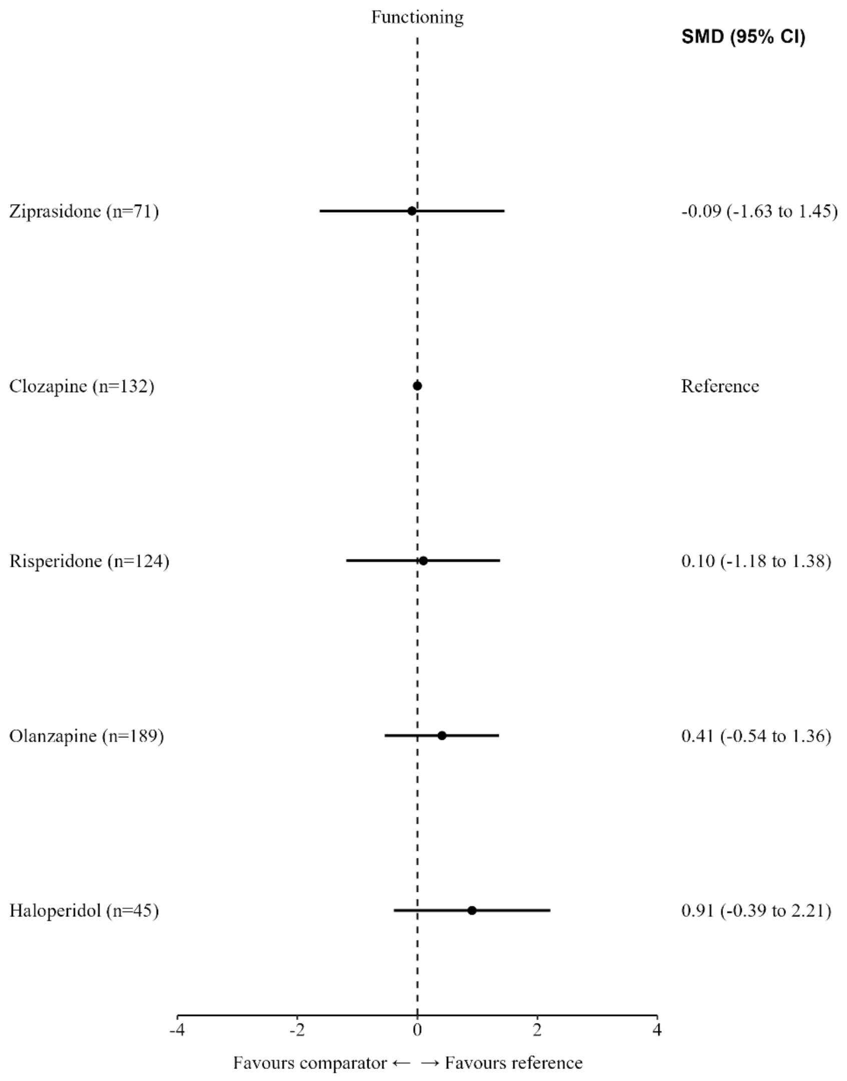

Effect sizes, measured as standardized mean difference (SMD), are from the network meta-analysis. Order of treatments is according to the mean effect size. Reference is clozapine. The direction of the effect is indicated below the x-axis.

League table for the outcome: Functioning

**Clozapine**	0.09 (-1.45 to 1.63)	0.85 (-0.92 to 2.63)	-0.55 (-1.70 to 0.61)	-1.55 (-3.35 to 0.24)
0.09 (-1.45 to 1.63)	**Ziprasidone**	NA	NA	NA
-0.10 (-1.38 to 1.18)	-0.19 (-2.19 to 1.82)	**Risperidone**	0.39 (-1.13 to 1.92)	NA
-0.41 (-1.36 to 0.54)	-0.50 (-2.31 to 1.31)	-0.31 (-1.54 to 0.92)	**Olanzapine**	-0.00 (-1.59 to 1.59)
-0.91 (-2.21 to 0.39)	-1.00 (-3.02 to 1.02)	-0.81 (-2.47 to 0.85)	-0.50 (-1.76 to 0.76)	**Haloperidol**

Treatments are presented in order of efficacy ranking. Results of the network meta-analysis are reported in the left lower half and results of pairwise meta-analyses in the right upper half. Each cell provides the effect estimate and the corresponding 95% credible interval (95% CI) of a comparison (left lower half: treatment in column versus treatment in row; right upper half: treatment in row versus treatment in column). The type of effect size measure is standardized mean difference (SMD). Bold results indicate 95% CI excluding no effect. NA=not available.

Forest plot of results of pairwise meta-analyses

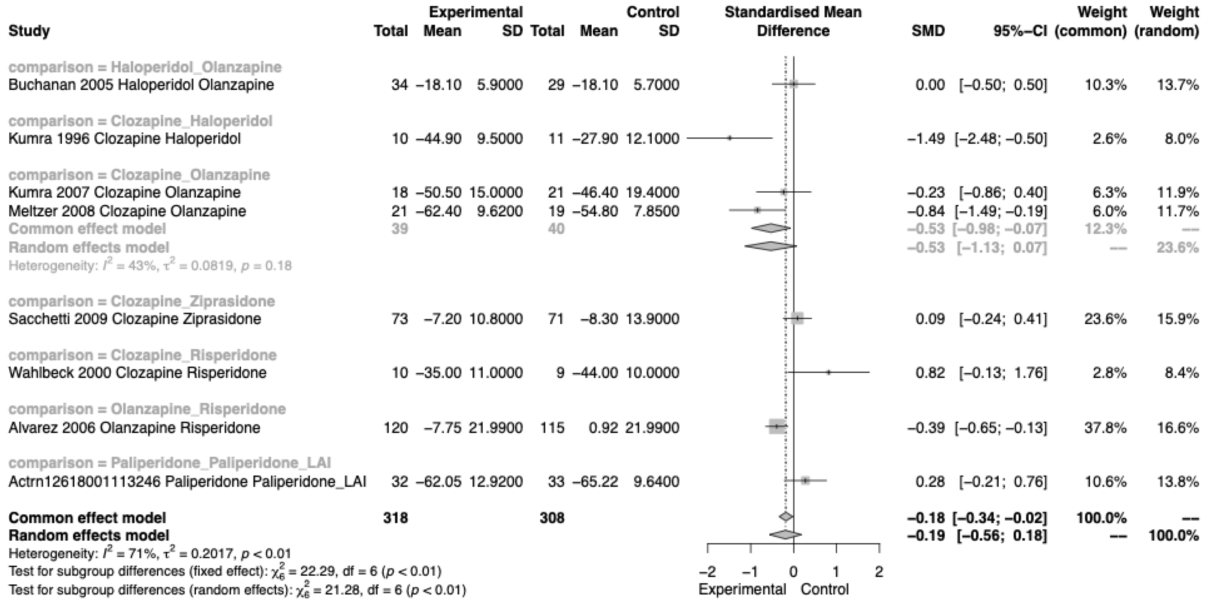

Study	Experimental Total	Experimental Mean	Experimental SD	Control Total	Control Mean	Control SD	SMD	95%-CI	Weight (common)	Weight (random)
comparison = Haloperidol_Olanzapine										
Buchanan 2005 Haloperidol Olanzapine	34	−18.10	5.9000	29	−18.10	5.7000	0.00	[−0.50; 0.50]	10.3%	13.7%
comparison = Clozapine_Haloperidol										
Kumra 1996 Clozapine Haloperidol	10	−44.90	9.5000	11	−27.90	12.1000	−1.49	[−2.48; −0.50]	2.6%	8.0%
comparison = Clozapine_Olanzapine										
Kumra 2007 Clozapine Olanzapine	18	−50.50	15.0000	21	−46.40	19.4000	−0.23	[−0.86; 0.40]	6.3%	11.9%
Meltzer 2008 Clozapine Olanzapine	21	−62.40	9.6200	19	−54.80	7.8500	−0.84	[−1.49; −0.19]	6.0%	11.7%
Common effect model	39			40			−0.53	[−0.98; −0.07]	12.3%	--
Random effects model							−0.53	[−1.13; 0.07]	--	23.6%
Heterogeneity: $I^2 = 43\%$, $\tau^2 = 0.0819$, $p = 0.18$										
comparison = Clozapine_Ziprasidone										
Sacchetti 2009 Clozapine Ziprasidone	73	−7.20	10.8000	71	−8.30	13.9000	0.09	[−0.24; 0.41]	23.6%	15.9%
comparison = Clozapine_Risperidone										
Wahlbeck 2000 Clozapine Risperidone	10	−35.00	11.0000	9	−44.00	10.0000	0.82	[−0.13; 1.76]	2.8%	8.4%
comparison = Olanzapine_Risperidone										
Alvarez 2006 Olanzapine Risperidone	120	−7.75	21.9900	115	0.92	21.9900	−0.39	[−0.65; −0.13]	37.8%	16.6%
comparison = Paliperidone_Paliperidone_LAI										
Actrn12618001113246 Paliperidone Paliperidone_LAI	32	−62.05	12.9200	33	−65.22	9.6400	0.28	[−0.21; 0.76]	10.6%	13.8%
**Common effect model**	**318**			**308**			**−0.18**	**[−0.34; −0.02]**	**100.0%**	**--**
**Random effects model**							**−0.19**	**[−0.56; 0.18]**	**--**	**100.0%**

Heterogeneity: $I^2 = 71\%$, $\tau^2 = 0.2017$, $p < 0.01$
Test for subgroup differences (fixed effect): $\chi^2_6 = 22.29$, df = 6 ($p < 0.01$)
Test for subgroup differences (random effects): $\chi^2_6 = 21.28$, df = 6 ($p < 0.01$)

A summary effect size is calculated by pairwise meta-analyses of all studies of a specific comparison. The type of effect size measure is standardized mean difference (SMD).

8.13 Quality of life

Forest plot of results of pairwise meta-analyses

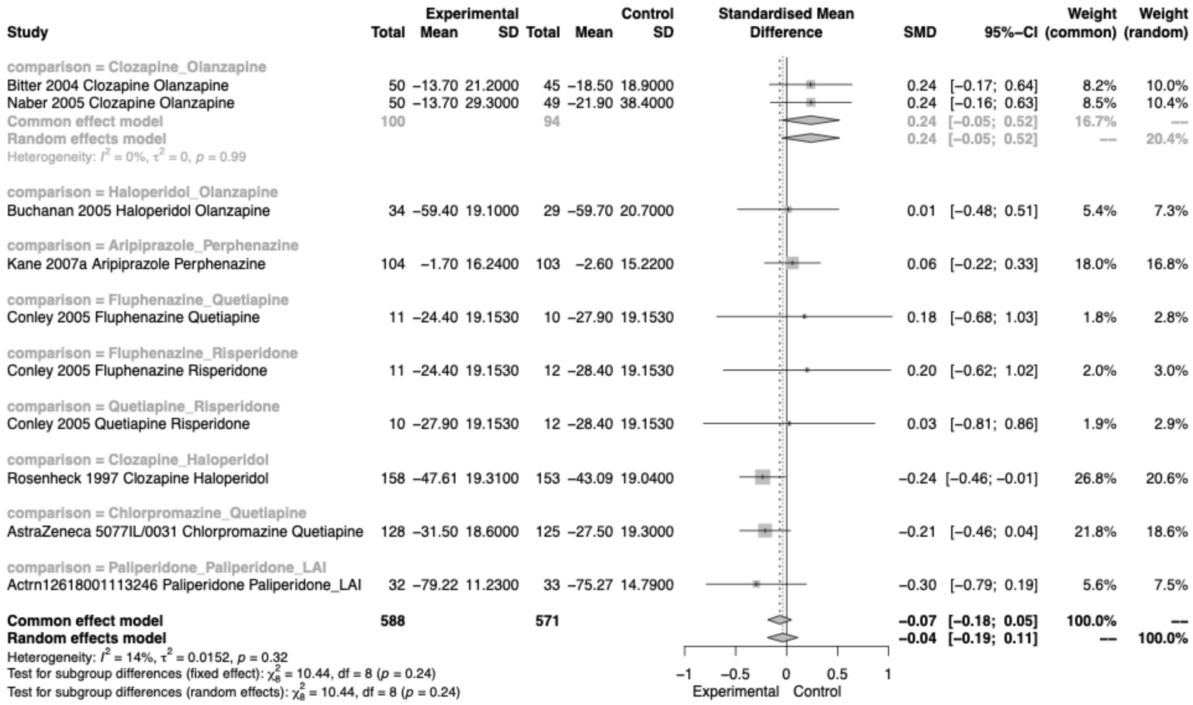

A summary effect size is calculated by pairwise meta-analyses of all studies of a specific comparison. The type of effect size measure is standardized mean difference (SMD).

9 Evaluation of heterogeneity

Outcome	Between study variance (tau)	Outcome type used as comparator	Predictive distribution of tau	Location of the estimated common tau compared to the quartiles of the empirical predictive distribution	Judgement on heterogeneity
Continuous		from Rhodes et al 2015	Median (IQR)		
Overall symptoms (SMD)	0.1804	Mental health outcome	0.19 (0.09, 0.42)	Between 25%-and 50%-quantile	low-moderate
Positive symptoms (SMD)	0.1840	Mental health outcome	0.19 (0.09, 0.42)	Between 25%-and 50%-quantile	low-moderate
Negative symptoms (SMD)	0.5014	Mental health outcome	0.19 (0.09, 0.42)	Above 75%-quantile	high
Weight gain (MD)	0.6602	General physical health and adverse event and pain and quality of life/functioning	0.18 (0.07, 0.43)	Above 75%-quantile	high
Prolactin (MD)	0	General physical health and adverse event and pain and quality of life/functioning	0.18 (0.07, 0.43)	Below 25%-quantile	low
Functioning (SMD)	0.7686	Mental health outcome	0.19 (0.09, 0.42)	Above 75%-quantile	high
Quality of life (SMD)	0	Mental health outcome	0.19 (0.09, 0.42)	Below 25%-quantile	low
Dichotomous		from Turner et al 2015	Median (IQR)		
Response (OR)	0.3783	Mental health indicators	0.31 (0.18, 0.53)	Between 50%-and 75%-quantile	moderate-high
Dropout for any reason (OR)	0.2674	Withdrawals/drop-outs	0.2 (0.11, 0.36)	Between 50%-and 75%-quantile	moderate-high
Dropout for inefficacy (OR)	0.0951	Withdrawals/drop-outs	0.2 (0.11, 0.36)	Below 25%-quantile	low
Dropout for adverse events (OR)	0	Withdrawals/drop-outs	0.2 (0.11, 0.36)	Below 25%-quantile	low
Use of antiparkinsonian medication (OR)	0.2990	Adverse events	0.35 (0.21, 0.6)	Between 50%-and 75%-quantile	moderate-high
Sedation (OR)	0	Adverse events	0.35 (0.21, 0.6)	Below 25%-quantile	low

10 Evaluation of inconsistency

Outcome	Number of studies (comparisons) [interventions]	P value of design-by-treatment interaction test	Inconsistent comparisons of detachable comparisons (%) (SIDE-test p<0.10)	Judgement
Continuous				
Overall symptoms	45 (57) [12]	< 0.0001	2 / 18 (11.11%)	Important evidence of inconsistency
Positive symptoms	39 (51) [11]	0.4937	2 / 17 (11.76%)	Little evidence of inconsistency
Negative symptoms	42 (54) [12]	0.7478	1 / 18 (5.56%)	Little evidence of inconsistency
Weight gain	28 (40) [11]	0.9701	1 / 15 (6.67%)	Little evidence of inconsistency
Prolactin	10 (17) [8]	0.4395	2 / 13 (15.38%)	Little evidence of inconsistency
QTc prolongation	5 (5) [7]	No closed loops	No closed loops	Inconsistency not estimable
Functioning	7 (7) [5]	0.0097	0 / 5 (0%)	Some evidence of inconsistency
Quality of life	8 (9) [11]	No closed loops	No closed loops	Inconsistency not estimable
Dichotomous				
Response	46 (66) [20]	0.0194	4 / 31 (12.90%)	Important evidence of inconsistency
Dropout for any reason	54 (75) [18]	0.9155	1 / 27 (3.70%)	Little evidence of inconsistency
Dropout for inefficacy	48 (67) [17]	0.7432	2 / 26 (7.69%)	Little evidence of inconsistency
Dropout for adverse events	50 (71) [19]	0.8684	0 / 29 (0%)	No evidence of inconsistency
Use of antiparkinsonian medication	24 (35) [16]	0.7994	0 / 14 (0%)	No evidence of inconsistency
Sedation	30 (44) [16]	0.5939	1 / 24 (4.17%)	Little evidence of inconsistency

11 Results of the sensitivity analyses of the primary outcome

11.1 Excluding 3 old clozapine-chlorpromazine studies

Number of studies: k = 42

Number of pairwise comparisons: m = 54

Number of treatments: n = 12

Number of designs: d = 20

Network plot

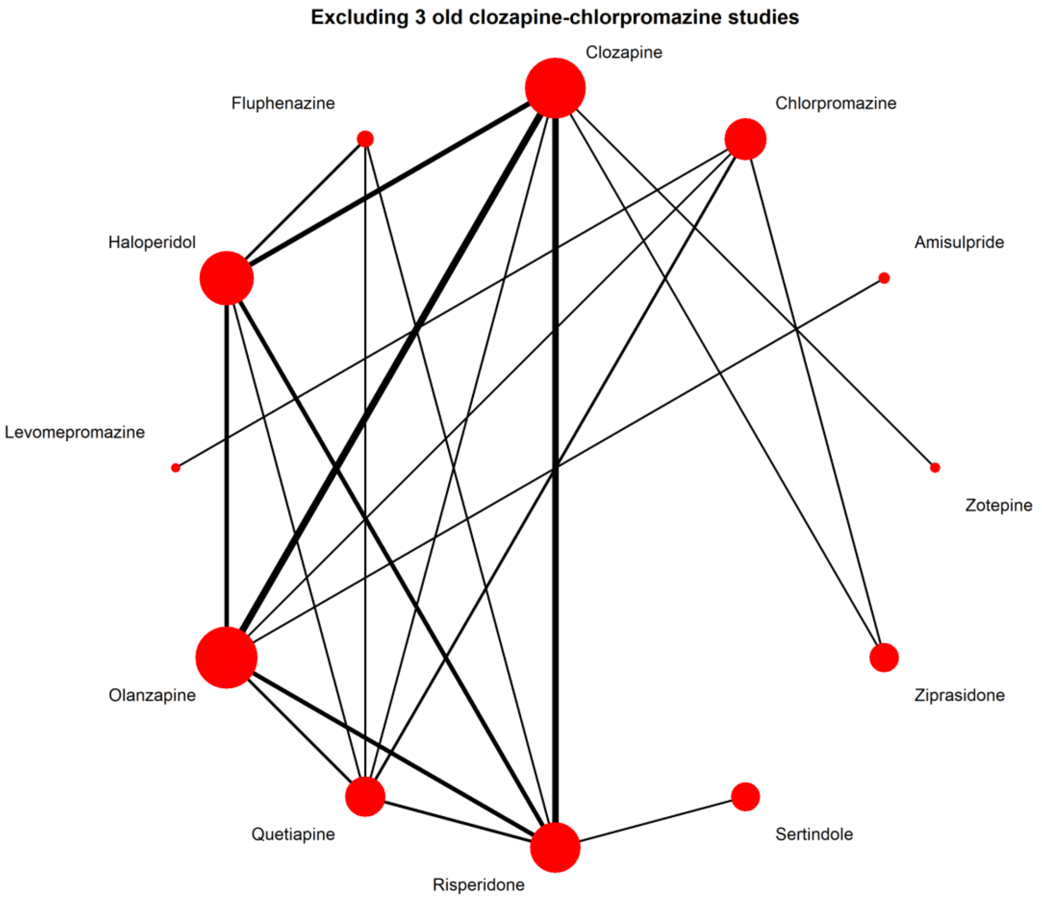

Lines link treatments with direct comparisons in trials; thickness of lines corresponds to the number of trials evaluating the comparison; size of the nodes corresponds to the number of participants assigned to the treatment.

τ	P-value	Inconsistency loop (%)
**0.0325**	**0.2979**	**5.88**

<u>Forest-plot of results of network meta-analysis for antipsychotic drugs versus clozapine</u>

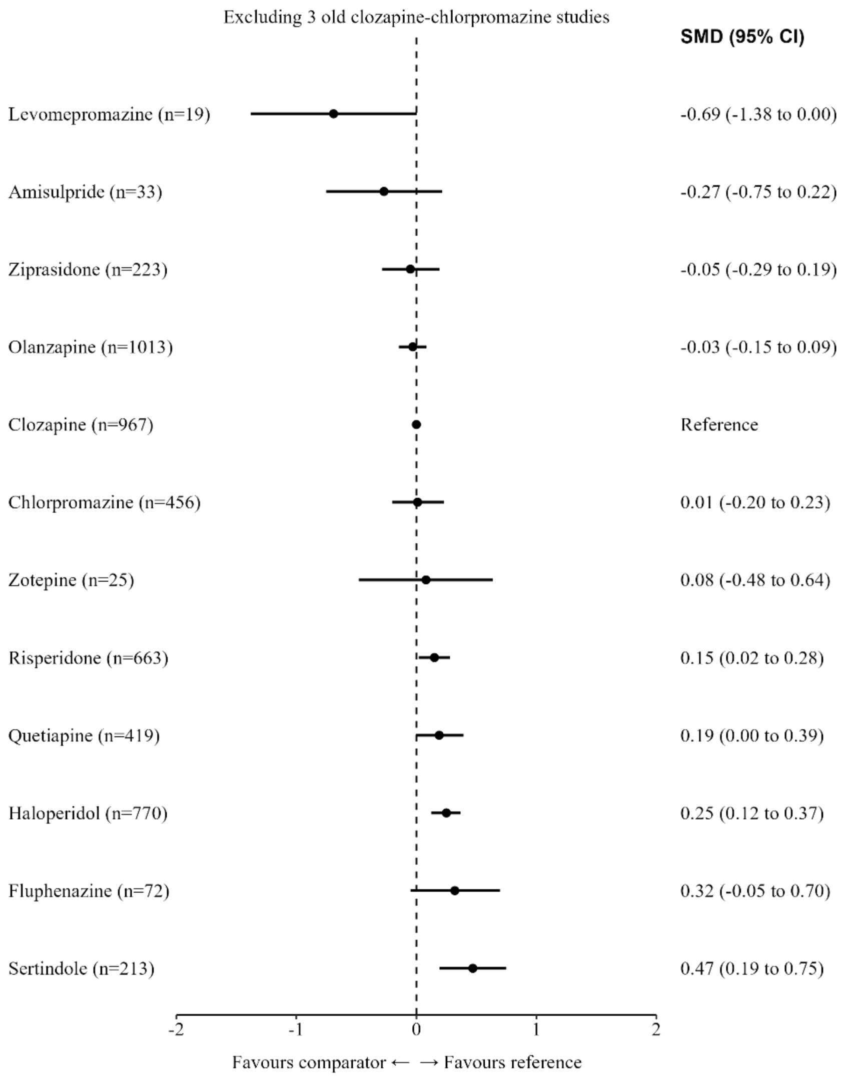

Effect sizes, measured as standardized mean difference (SMD), are from the network meta-analysis. Order of treatments is according to the mean effect size. Reference is clozapine. The direction of the effect is indicated below the x-axis.

League table

**Levomepromazine**	NA	NA	NA	NA	**-0.70 (-1.36 to -0.04)**	NA	NA	NA	NA	NA	NA
-0.42 (-1.26 to 0.42)	**Amisulpride**	NA	-0.24 (-0.71 to 0.23)	NA	NA	NA	NA	NA	NA	NA	NA
-0.64 (-1.33 to 0.05)	-0.22 (-0.75 to 0.31)	**Ziprasidone**	NA	-0.02 (-0.36 to 0.31)	-0.07 (-0.30 to 0.16)	NA	NA	NA	NA	NA	NA
-0.66 (-1.35 to 0.03)	-0.24 (-0.71 to 0.23)	-0.02 (-0.26 to 0.23)	**Olanzapine**	-0.03 (-0.18 to 0.13)	-0.22 (-0.66 to 0.23)	NA	**-0.23 (-0.41 to -0.05)**	-0.05 (-0.57 to 0.47)	**-0.27 (-0.44 to -0.11)**	NA	NA
-0.69 (-1.38 to 0.00)	-0.27 (-0.75 to 0.22)	-0.05 (-0.29 to 0.19)	-0.03 (-0.15 to 0.09)	**Clozapine**	NA	-0.08 (-0.64 to 0.48)	-0.11 (-0.28 to 0.05)	**-1.01 (-1.81 to -0.21)**	**-0.19 (-0.35 to -0.03)**	NA	NA
**-0.70 (-1.36 to -0.04)**	-0.28 (-0.80 to 0.24)	-0.06 (-0.26 to 0.14)	-0.04 (-0.26 to 0.17)	-0.01 (-0.23 to 0.20)	**Chlorpromazine**	NA	NA	**-0.22 (-0.40 to -0.03)**	NA	NA	NA
-0.77 (-1.66 to 0.12)	-0.35 (-1.08 to 0.39)	-0.12 (-0.73 to 0.48)	-0.11 (-0.68 to 0.46)	-0.08 (-0.64 to 0.48)	-0.06 (-0.66 to 0.53)	**Zotepine**	NA	NA	NA	NA	NA
**-0.84 (-1.54 to -0.14)**	-0.42 (-0.91 to 0.07)	-0.20 (-0.46 to 0.07)	**-0.18 (-0.32 to -0.05)**	**-0.15 (-0.28 to -0.02)**	-0.14 (-0.37 to 0.10)	-0.07 (-0.65 to 0.50)	**Risperidone**	-0.06 (-0.69 to 0.57)	-0.14 (-0.42 to 0.14)	0.02 (-0.75 to 0.79)	**-0.32 (-0.57 to -0.07)**
**-0.88 (-1.56 to -0.20)**	-0.46 (-0.97 to 0.05)	-0.24 (-0.48 to 0.00)	**-0.22 (-0.42 to -0.03)**	-0.19 (-0.39 to 0.00)	**-0.18 (-0.35 to -0.02)**	-0.12 (-0.71 to 0.48)	-0.04 (-0.26 to 0.17)	**Quetiapine**	-0.14 (-0.38 to 0.10)	0.15 (-0.64 to 0.93)	NA
**-0.93 (-1.63 to -0.24)**	**-0.51 (-1.00 to -0.03)**	**-0.29 (-0.54 to -0.04)**	**-0.28 (-0.40 to -0.15)**	**-0.25 (-0.37 to -0.12)**	**-0.23 (-0.44 to -0.02)**	-0.17 (-0.74 to 0.40)	-0.10 (-0.24 to 0.05)	-0.05 (-0.24 to 0.13)	**Haloperidol**	-0.17 (-0.59 to 0.25)	NA
**-1.01 (-1.79 to -0.24)**	-0.59 (-1.19 to 0.01)	-0.37 (-0.80 to 0.06)	-0.35 (-0.73 to 0.02)	-0.32 (-0.70 to 0.05)	-0.31 (-0.72 to 0.10)	-0.25 (-0.92 to 0.43)	-0.17 (-0.55 to 0.21)	-0.13 (-0.52 to 0.26)	-0.08 (-0.44 to 0.28)	**Fluphenazine**	NA
**-1.16 (-1.90 to -0.42)**	**-0.74 (-1.28 to -0.19)**	**-0.52 (-0.88 to -0.16)**	**-0.50 (-0.78 to -0.22)**	**-0.47 (-0.75 to -0.19)**	**-0.46 (-0.80 to -0.12)**	-0.39 (-1.02 to 0.23)	**-0.32 (-0.57 to -0.07)**	-0.28 (-0.60 to 0.05)	-0.23 (-0.51 to 0.06)	-0.15 (-0.60 to 0.30)	**Sertindole**

Treatments are presented in order of efficacy ranking. Results of the network meta-analysis are reported in the left lower half and results of pairwise meta-analyses in the right upper half. Each cell provides the effect estimate and the corresponding 95% credible interval (95% CI) of a comparison (left lower half: treatment in column versus treatment in row; right upper half: treatment in row versus treatment in column). The type of effect size measure is standardized mean difference (SMD). Bold results indicate 95% CI excluding no effect. NA=not available.

11.2 Excluding studies that included intolerant patients

Number of studies: k = 35

Number of pairwise comparisons: m = 47

Number of treatments: n = 11

Number of designs: d = 19

Network plot

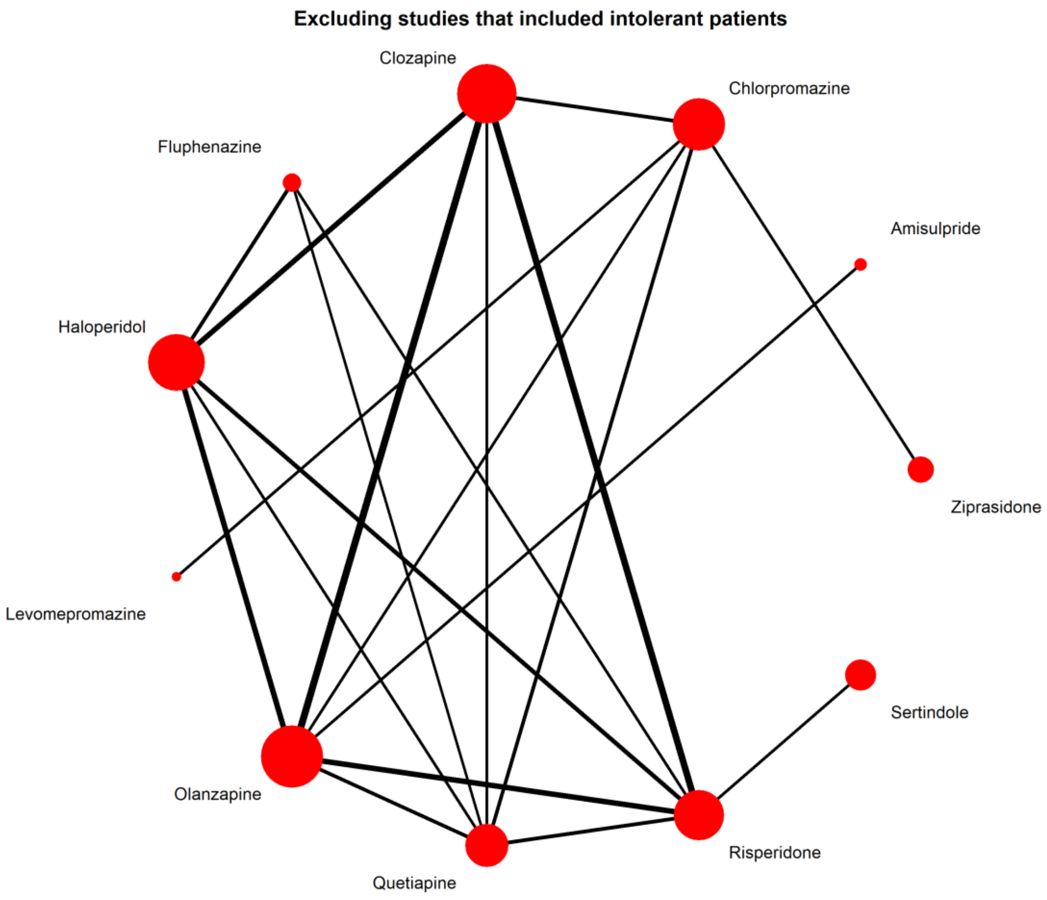

Lines link treatments with direct comparisons in trials; thickness of lines corresponds to the number of trials evaluating the comparison; size of the nodes corresponds to the number of participants assigned to the treatment.

τ	P-value	Inconsistency loop (%)
**0.2007**	**0.0002**	**18.75**

Forest-plot of results of network meta-analysis for antipsychotic drugs versus clozapine

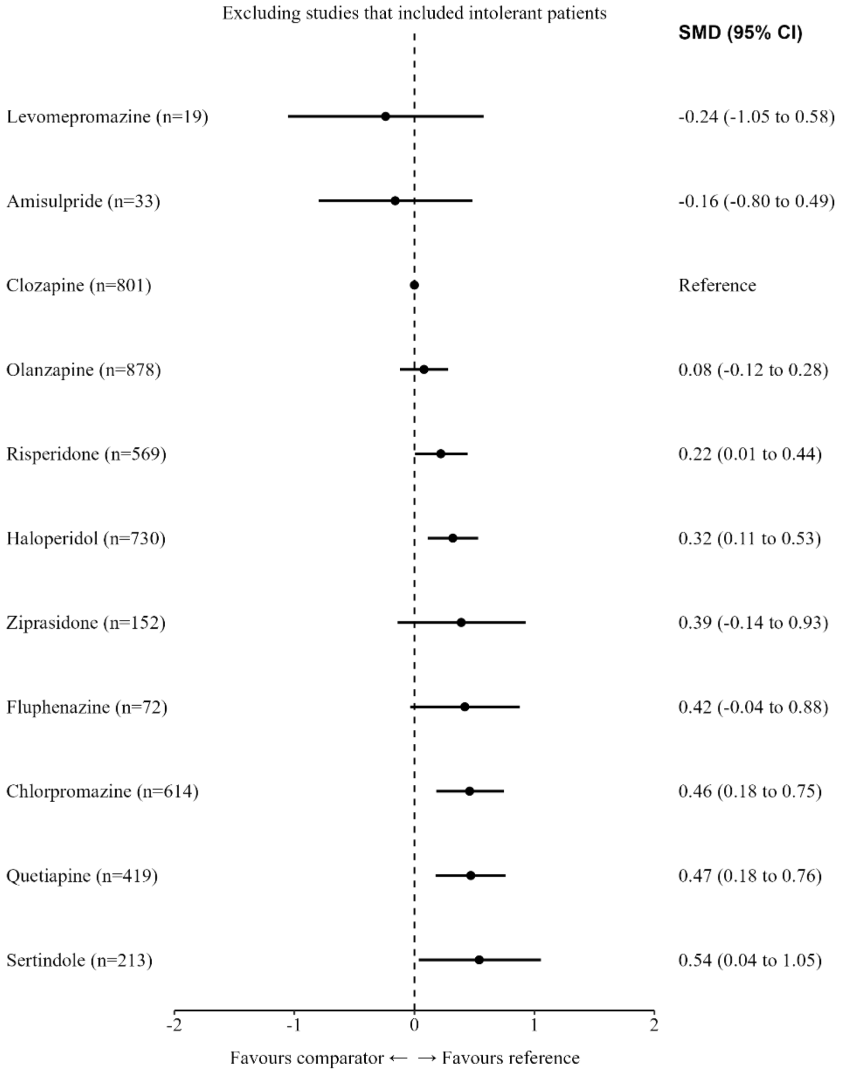

Effect sizes, measured as standardized mean difference (SMD), are from the network meta-analysis. Order of treatments is according to the mean effect size. Reference is clozapine. The direction of the effect is indicated below the x-axis.

League table

**Levomepromazine**	NA	NA	NA	NA	NA	NA	NA	-0.70 (-1.47 to 0.06)	NA	NA
-0.08 (-1.10 to 0.94)	**Amisulpride**	NA	-0.24 (-0.85 to 0.37)	NA	NA	NA	NA	NA	NA	NA
-0.24 (-1.05 to 0.58)	-0.16 (-0.80 to 0.49)	**Clozapine**	-0.01 (-0.28 to 0.26)	-0.14 (-0.42 to 0.13)	-0.13 (-0.42 to 0.15)	NA	NA	**-0.85 (-1.24 to -0.45)**	**-1.01 (-1.90 to -0.12)**	NA
-0.32 (-1.14 to 0.50)	-0.24 (-0.85 to 0.37)	-0.08 (-0.28 to 0.12)	**Olanzapine**	-0.23 (-0.55 to 0.08)	-0.28 (-0.58 to 0.01)	NA	NA	-0.22 (-0.80 to 0.37)	-0.07 (-0.66 to 0.53)	NA
-0.46 (-1.29 to 0.37)	-0.38 (-1.03 to 0.27)	**-0.22 (-0.44 to -0.01)**	-0.14 (-0.37 to 0.09)	**Risperidone**	-0.15 (-0.56 to 0.27)	NA	0.02 (-0.84 to 0.89)	NA	-0.06 (-0.75 to 0.64)	-0.32 (-0.78 to 0.14)
-0.56 (-1.38 to 0.27)	-0.48 (-1.12 to 0.17)	**-0.32 (-0.53 to -0.11)**	**-0.24 (-0.46 to -0.03)**	-0.10 (-0.34 to 0.15)	**Haloperidol**	NA	-0.17 (-0.68 to 0.33)	NA	0.14 (-0.32 to 0.60)	NA
-0.63 (-1.52 to 0.26)	-0.55 (-1.36 to 0.27)	-0.39 (-0.93 to 0.14)	-0.31 (-0.85 to 0.23)	-0.17 (-0.73 to 0.39)	-0.07 (-0.62 to 0.47)	**Ziprasidone**	NA	-0.07 (-0.52 to 0.38)	NA	NA
-0.66 (-1.57 to 0.25)	-0.58 (-1.34 to 0.18)	-0.42 (-0.88 to 0.04)	-0.34 (-0.80 to 0.12)	-0.20 (-0.66 to 0.27)	-0.10 (-0.53 to 0.32)	-0.03 (-0.70 to 0.65)	**Fluphenazine**	NA	-0.15 (-1.02 to 0.73)	NA
-0.70 (-1.47 to 0.06)	-0.62 (-1.30 to 0.06)	**-0.46 (-0.75 to -0.18)**	**-0.38 (-0.68 to -0.09)**	-0.24 (-0.57 to 0.09)	-0.14 (-0.45 to 0.16)	-0.07 (-0.52 to 0.38)	-0.04 (-0.54 to 0.46)	**Chlorpromazine**	-0.22 (-0.55 to 0.12)	NA
-0.71 (-1.52 to 0.11)	-0.62 (-1.30 to 0.05)	**-0.47 (-0.76 to -0.18)**	**-0.39 (-0.68 to -0.09)**	-0.24 (-0.57 to 0.08)	-0.15 (-0.44 to 0.14)	-0.07 (-0.60 to 0.45)	-0.05 (-0.53 to 0.44)	-0.00 (-0.28 to 0.27)	**Quetiapine**	NA
-0.78 (-1.73 to 0.17)	-0.70 (-1.50 to 0.10)	**-0.54 (-1.05 to -0.04)**	-0.46 (-0.98 to 0.05)	-0.32 (-0.78 to 0.14)	-0.22 (-0.75 to 0.30)	-0.15 (-0.87 to 0.57)	-0.12 (-0.78 to 0.53)	-0.08 (-0.64 to 0.48)	-0.08 (-0.64 to 0.48)	**Sertindole**

Treatments are presented in order of efficacy ranking. Results of the network meta-analysis are reported in the left lower half and results of pairwise meta-analyses in the right upper half. Each cell provides the effect estimate and the corresponding 95% credible interval (95% CI) of a comparison (left lower half: treatment in column versus treatment in row; right upper half: treatment in row versus treatment in column). The type of effect size measure is standardized mean difference (SMD). Bold results indicate 95% CI excluding no effect. NA=not available.

11.3 Double-blind studies only

Number of studies: k = 41

Number of pairwise comparisons: m = 53

Number of treatments: n = 12

Number of designs: d = 20

Network plot

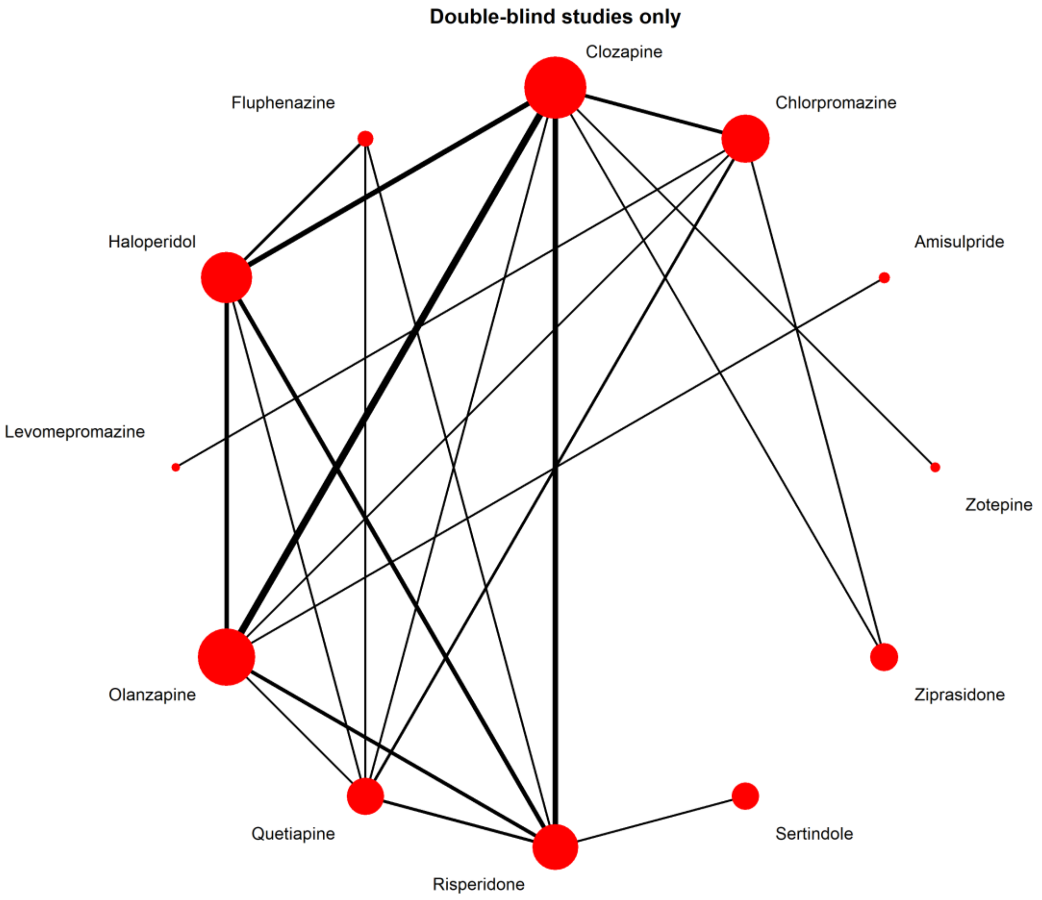

Lines link treatments with direct comparisons in trials; thickness of lines corresponds to the number of trials evaluating the comparison; size of the nodes corresponds to the number of participants assigned to the treatment.

τ	P-value	Inconsistency loop (%)
0.1759	0.0001	16.67

Forest-plot of results of network meta-analysis for antipsychotic drugs versus clozapine

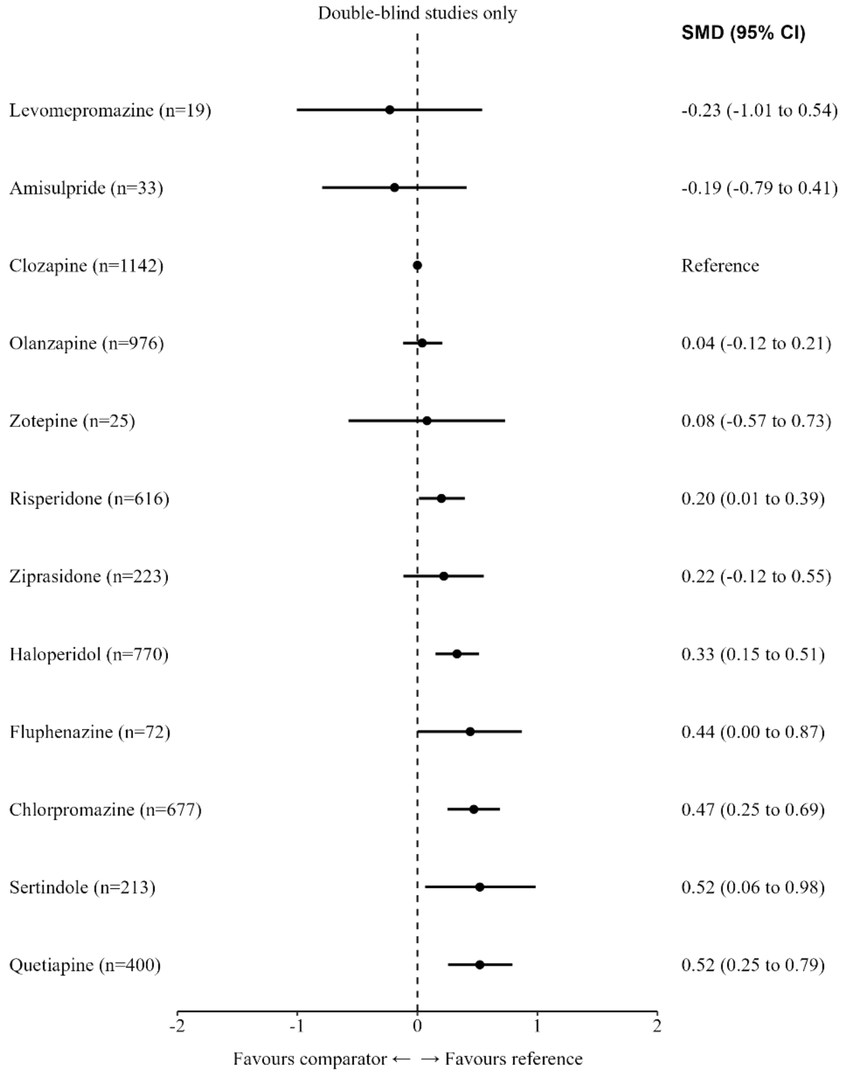

Effect sizes, measured as standardized mean difference (SMD), are from the network meta-analysis. Order of treatments is according to the mean effect size. Reference is clozapine. The direction of the effect is indicated below the x-axis.

League table

**Amisulpride**	NA	NA	-0.24 (-0.82 to 0.34)	NA	NA	NA	NA	NA	NA	NA	NA
0.04 (-0.93 to 1.01)	**Levomepromazine**	NA	NA	NA	NA	NA	NA	NA	-0.70 (-1.44 to 0.04)	NA	NA
-0.19 (-0.79 to 0.41)	-0.23 (-1.01 to 0.54)	**Clozapine**	-0.01 (-0.21 to 0.19)	-0.08 (-0.73 to 0.57)	-0.13 (-0.37 to 0.11)	0.02 (-0.45 to 0.50)	-0.18 (-0.44 to 0.07)	NA	**-0.78 (-1.07 to -0.48)**	NA	**-1.01 (-1.88 to -0.14)**
-0.24 (-0.82 to 0.34)	-0.28 (-1.06 to 0.50)	-0.04 (-0.21 to 0.12)	**Olanzapine**	NA	-0.25 (-0.56 to 0.06)	NA	**-0.28 (-0.55 to -0.01)**	NA	-0.22 (-0.77 to 0.34)	NA	-0.38 (-1.37 to 0.62)
-0.27 (-1.16 to 0.62)	-0.31 (-1.32 to 0.70)	-0.08 (-0.73 to 0.57)	-0.03 (-0.71 to 0.64)	**Zotepine**	NA	NA	NA	NA	NA	NA	NA
-0.40 (-1.01 to 0.22)	-0.44 (-1.23 to 0.35)	**-0.20 (-0.39 to -0.01)**	-0.16 (-0.37 to 0.05)	-0.13 (-0.81 to 0.55)	**Risperidone**	NA	-0.15 (-0.48 to 0.19)	0.02 (-0.82 to 0.86)	NA	-0.32 (-0.74 to 0.10)	-0.06 (-0.74 to 0.62)
-0.41 (-1.09 to 0.27)	-0.45 (-1.26 to 0.36)	-0.22 (-0.55 to 0.12)	-0.17 (-0.54 to 0.19)	-0.14 (-0.87 to 0.59)	-0.01 (-0.39 to 0.36)	**Ziprasidone**	NA	NA	-0.07 (-0.48 to 0.34)	NA	NA
-0.52 (-1.14 to 0.09)	-0.57 (-1.35 to 0.22)	**-0.33 (-0.51 to -0.15)**	**-0.29 (-0.48 to -0.09)**	-0.25 (-0.93 to 0.42)	-0.13 (-0.34 to 0.09)	-0.11 (-0.48 to 0.26)	**Haloperidol**	-0.17 (-0.66 to 0.31)	NA	NA	0.14 (-0.27 to 0.56)
-0.63 (-1.36 to 0.10)	-0.67 (-1.54 to 0.21)	-0.44 (-0.87 to 0.00)	-0.39 (-0.83 to 0.05)	-0.36 (-1.14 to 0.43)	-0.23 (-0.67 to 0.21)	-0.22 (-0.76 to 0.32)	-0.10 (-0.51 to 0.31)	**Fluphenazine**	NA	NA	-0.15 (-1.00 to 0.71)
**-0.66 (-1.29 to -0.03)**	-0.70 (-1.44 to 0.04)	**-0.47 (-0.69 to -0.25)**	**-0.42 (-0.67 to -0.18)**	-0.39 (-1.08 to 0.30)	-0.27 (-0.54 to 0.01)	-0.25 (-0.58 to 0.07)	-0.14 (-0.39 to 0.12)	-0.03 (-0.50 to 0.43)	**Chlorpromazine**	NA	-0.22 (-0.52 to 0.09)
-0.72 (-1.46 to 0.03)	-0.76 (-1.65 to 0.14)	**-0.52 (-0.98 to -0.06)**	**-0.48 (-0.95 to -0.01)**	-0.45 (-1.25 to 0.35)	-0.32 (-0.74 to 0.10)	-0.31 (-0.87 to 0.26)	-0.19 (-0.66 to 0.28)	-0.09 (-0.70 to 0.52)	-0.06 (-0.56 to 0.44)	**Sertindole**	NA
**-0.72 (-1.36 to -0.07)**	-0.76 (-1.54 to 0.02)	**-0.52 (-0.79 to -0.25)**	**-0.48 (-0.77 to -0.19)**	-0.45 (-1.15 to 0.26)	**-0.32 (-0.62 to -0.02)**	-0.31 (-0.69 to 0.08)	-0.19 (-0.46 to 0.08)	-0.09 (-0.55 to 0.38)	-0.05 (-0.30 to 0.19)	0.00 (-0.51 to 0.52)	**Quetiapine**

Treatments are presented in order of efficacy ranking. Results of the network meta-analysis are reported in the left lower half and results of pairwise meta-analyses in the right upper half. Each cell provides the effect estimate and the corresponding 95% credible interval (95% CI) of a comparison (left lower half: treatment in column versus treatment in row; right upper half: treatment in row versus treatment in column). The type of effect size measure is standardized mean difference (SMD). Bold results indicate 95% CI excluding no effect. NA=not available

11.4 Excluding studies that presented only completer analyses

Number of studies: k = 37

Number of pairwise comparisons: m = 49

Number of treatments: n = 11

Number of designs: d = 20

Network plot

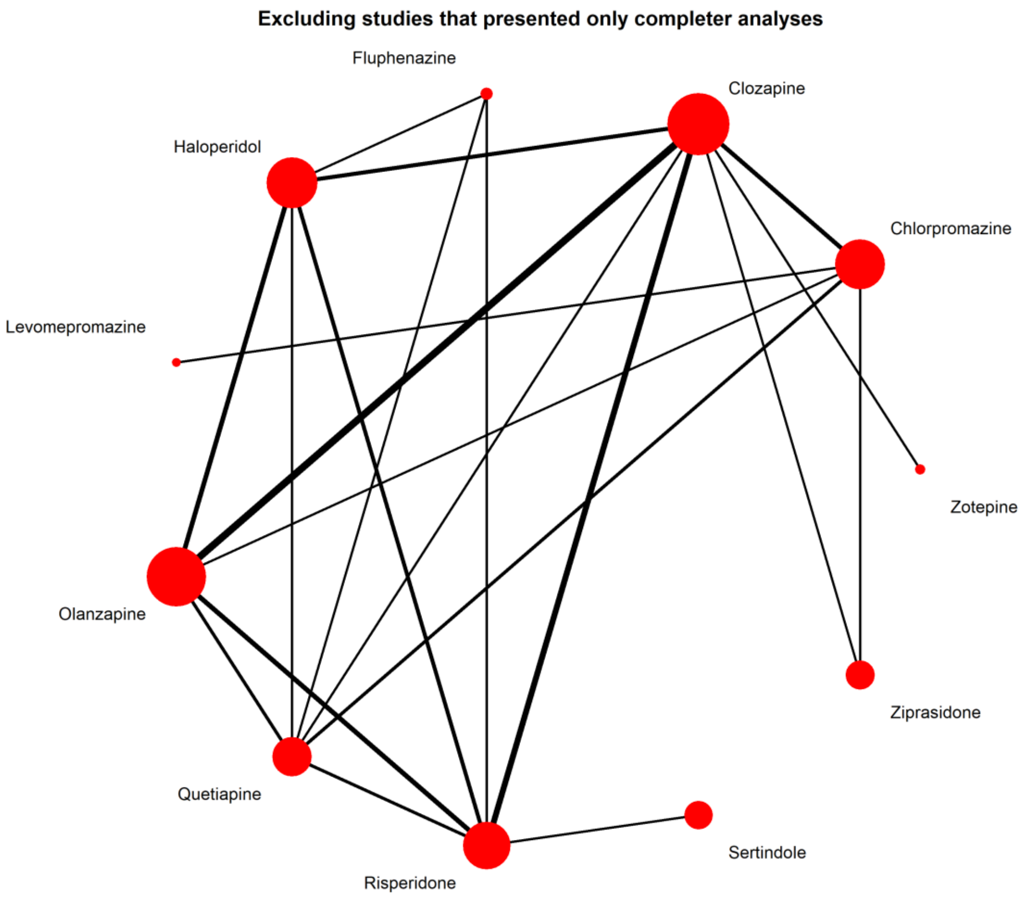

Lines link treatments with direct comparisons in trials; thickness of lines corresponds to the number of trials evaluating the comparison; size of the nodes corresponds to the number of participants assigned to the treatment.

τ	P-value	Inconsistency loop (%)
0.1979	0.0007	11.11

Forest-plot of results of network meta-analysis for antipsychotic drugs versus clozapine

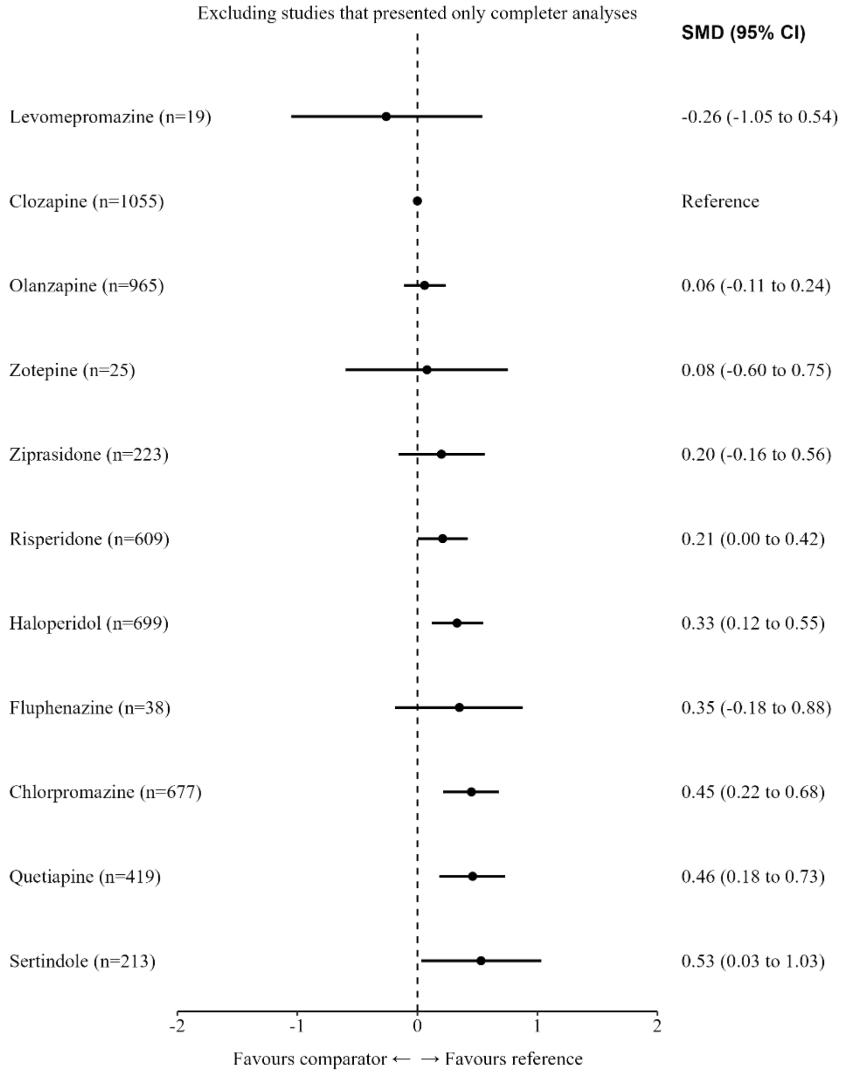

Effect sizes, measured as standardized mean difference (SMD), are from the network meta-analysis. Order of treatments is according to the mean effect size. Reference is clozapine. The direction of the effect is indicated below the x-axis.

League table

**Levomepromazine**	NA	NA	NA	NA	NA	NA	NA	-0.70 (-1.46 to 0.06)	NA	NA
-0.26 (-1.05 to 0.54)	**Clozapine**	-0.00 (-0.22 to 0.21)	-0.08 (-0.75 to 0.60)	0.02 (-0.48 to 0.53)	-0.10 (-0.36 to 0.17)	NA	-0.18 (-0.51 to 0.14)	**-0.77 (-1.09 to -0.46)**	**-1.01 (-1.89 to -0.13)**	NA
-0.32 (-1.12 to 0.49)	-0.06 (-0.24 to 0.11)	**Olanzapine**	NA	NA	-0.23 (-0.54 to 0.07)	NA	-0.28 (-0.57 to 0.01)	-0.22 (-0.80 to 0.37)	-0.07 (-0.66 to 0.52)	NA
-0.33 (-1.38 to 0.71)	-0.08 (-0.75 to 0.60)	-0.02 (-0.71 to 0.68)	**Zotepine**	NA	NA	NA	NA	NA	NA	NA
-0.46 (-1.29 to 0.38)	-0.20 (-0.56 to 0.16)	-0.14 (-0.53 to 0.25)	-0.12 (-0.89 to 0.64)	**Ziprasidone**	NA	NA	NA	-0.07 (-0.52 to 0.38)	NA	NA
-0.47 (-1.28 to 0.35)	-0.21 (-0.42 to 0.00)	-0.15 (-0.37 to 0.07)	-0.13 (-0.84 to 0.57)	-0.01 (-0.42 to 0.40)	**Risperidone**	0.02 (-0.84 to 0.88)	-0.07 (-0.44 to 0.30)	NA	-0.06 (-0.75 to 0.64)	-0.32 (-0.78 to 0.13)
-0.60 (-1.54 to 0.34)	-0.35 (-0.88 to 0.18)	-0.28 (-0.82 to 0.25)	-0.27 (-1.13 to 0.59)	-0.15 (-0.78 to 0.48)	-0.13 (-0.67 to 0.40)	**Fluphenazine**	0.09 (-0.58 to 0.77)	NA	-0.15 (-1.02 to 0.73)	NA
-0.59 (-1.40 to 0.22)	**-0.33 (-0.55 to -0.12)**	**-0.27 (-0.48 to -0.06)**	-0.26 (-0.97 to 0.45)	-0.13 (-0.54 to 0.27)	-0.12 (-0.36 to 0.12)	0.01 (-0.50 to 0.52)	**Haloperidol**	NA	0.14 (-0.31 to 0.60)	NA
-0.70 (-1.46 to 0.06)	**-0.45 (-0.68 to -0.22)**	**-0.38 (-0.64 to -0.13)**	-0.37 (-1.08 to 0.35)	-0.25 (-0.60 to 0.11)	-0.23 (-0.53 to 0.06)	-0.10 (-0.66 to 0.46)	-0.11 (-0.40 to 0.17)	**Chlorpromazine**	-0.22 (-0.54 to 0.11)	NA
-0.71 (-1.52 to 0.09)	**-0.46 (-0.73 to -0.18)**	**-0.39 (-0.68 to -0.11)**	-0.38 (-1.11 to 0.35)	-0.26 (-0.67 to 0.16)	-0.24 (-0.55 to 0.06)	-0.11 (-0.65 to 0.43)	-0.12 (-0.41 to 0.16)	-0.01 (-0.27 to 0.25)	**Quetiapine**	NA
-0.79 (-1.72 to 0.15)	**-0.53 (-1.03 to -0.03)**	-0.47 (-0.97 to 0.03)	-0.45 (-1.30 to 0.39)	-0.33 (-0.94 to 0.28)	-0.32 (-0.78 to 0.13)	-0.19 (-0.89 to 0.51)	-0.20 (-0.71 to 0.32)	-0.09 (-0.63 to 0.45)	-0.08 (-0.62 to 0.47)	**Sertindole**

Treatments are presented in order of efficacy ranking. Results of the network meta-analysis are reported in the left lower half and results of pairwise meta-analyses in the right upper half. Each cell provides the effect estimate and the corresponding 95% credible interval (95% CI) of a comparison (left lower half: treatment in column versus treatment in row; right upper half: treatment in row versus treatment in column). The type of effect size measure is standardized mean difference (SMD). Bold results indicate 95% CI excluding no effect. NA=not available.

11.5 Excluding studies that did not use operationalized criteria to diagnose schizophrenia

Number of studies: k = 44

Number of pairwise comparisons: m = 56

Number of treatments: n = 12

Number of designs: d = 21

Network plot

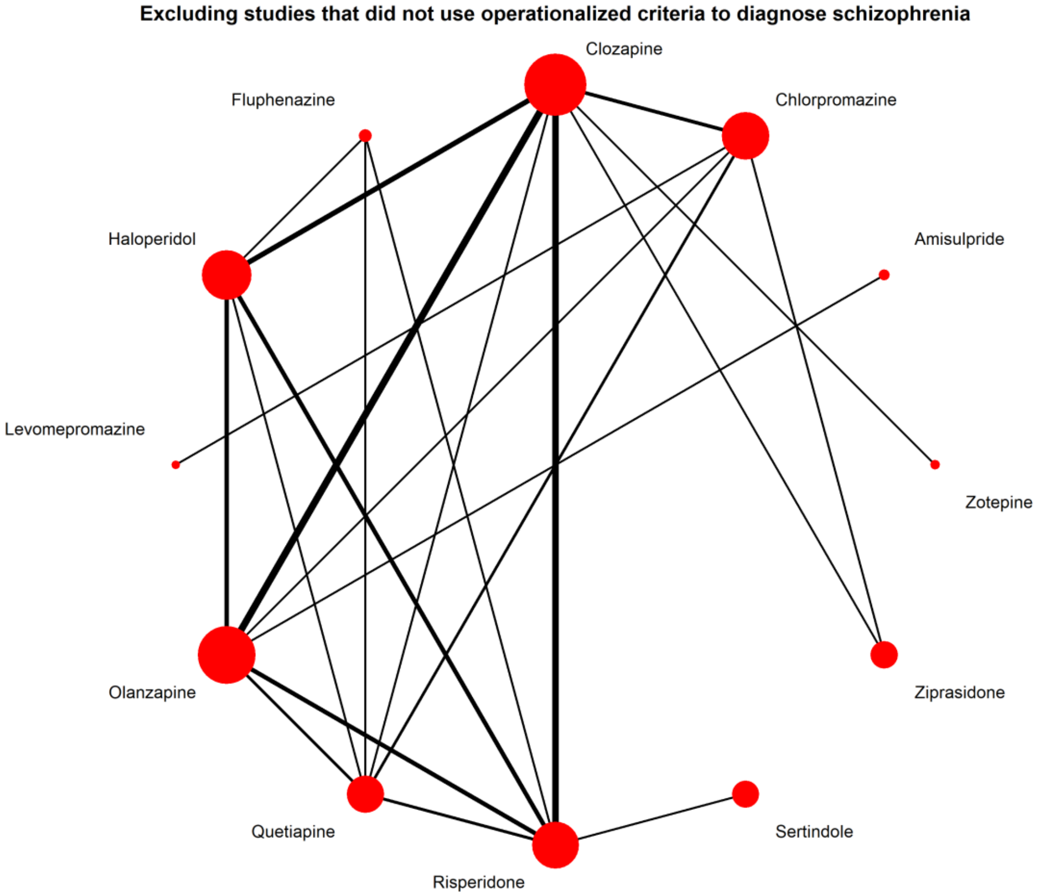

Lines link treatments with direct comparisons in trials; thickness of lines corresponds to the number of trials evaluating the comparison; size of the nodes corresponds to the number of participants assigned to the treatment.

τ	P-value	Inconsistency loop (%)
0.1848	< 0.0001	11.11

Forest-plot of results of network meta-analysis for antipsychotic drugs versus clozapine

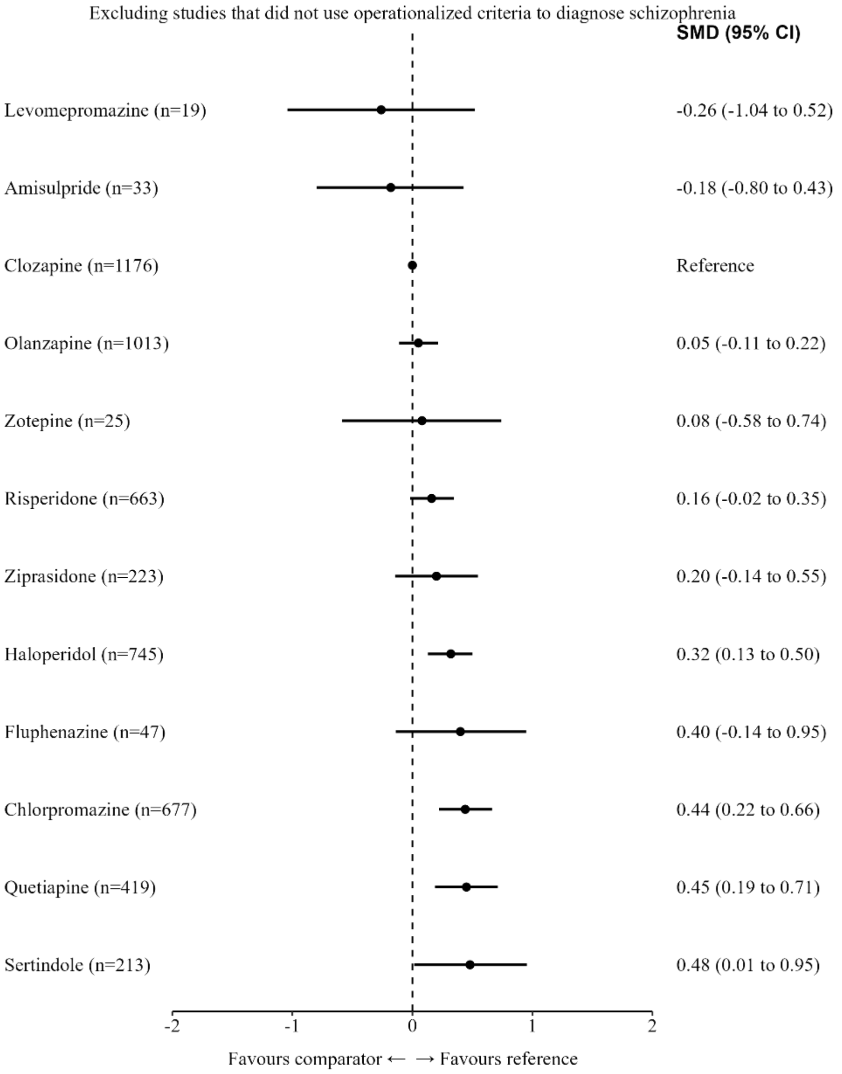

Effect sizes, measured as standardized mean difference (SMD), are from the network meta-analysis. Order of treatments is according to the mean effect size. Reference is clozapine. The direction of the effect is indicated below the x-axis.

League table

**Levomepromazine**	NA	NA	NA	NA	NA	NA	NA	NA	NA	-0.70 (-1.45 to 0.05)	NA
-0.08 (-1.06 to 0.91)	**Amisulpride**	NA	-0.24 (-0.83 to 0.35)	NA	NA	NA	NA	NA	NA	NA	NA
-0.26 (-1.04 to 0.52)	-0.18 (-0.80 to 0.43)	**Clozapine**	-0.01 (-0.22 to 0.19)	-0.08 (-0.74 to 0.58)	-0.08 (-0.30 to 0.15)	0.02 (-0.47 to 0.51)	-0.18 (-0.45 to 0.08)	NA	NA	**-0.78 (-1.08 to -0.47)**	**-1.01 (-1.88 to -0.14)**
-0.31 (-1.10 to 0.48)	-0.24 (-0.83 to 0.35)	-0.05 (-0.22 to 0.11)	**Olanzapine**	NA	-0.23 (-0.53 to 0.06)	NA	-0.28 (-0.56 to 0.00)	NA	NA	-0.22 (-0.78 to 0.35)	-0.07 (-0.65 to 0.52)
-0.34 (-1.36 to 0.69)	-0.26 (-1.16 to 0.64)	-0.08 (-0.74 to 0.58)	-0.03 (-0.71 to 0.66)	**Zotepine**	NA	NA	NA	NA	NA	NA	NA
-0.42 (-1.22 to 0.37)	-0.35 (-0.97 to 0.27)	-0.16 (-0.35 to 0.02)	-0.11 (-0.31 to 0.09)	-0.09 (-0.77 to 0.60)	**Risperidone**	NA	-0.15 (-0.49 to 0.19)	0.02 (-0.83 to 0.87)	-0.32 (-0.75 to 0.11)	NA	-0.06 (-0.74 to 0.63)
-0.46 (-1.28 to 0.36)	-0.39 (-1.08 to 0.31)	-0.20 (-0.55 to 0.14)	-0.15 (-0.52 to 0.22)	-0.12 (-0.87 to 0.62)	-0.04 (-0.42 to 0.35)	**Ziprasidone**	NA	NA	NA	-0.07 (-0.50 to 0.36)	NA
-0.58 (-1.37 to 0.22)	-0.50 (-1.12 to 0.12)	**-0.32 (-0.50 to -0.13)**	**-0.26 (-0.46 to -0.07)**	-0.24 (-0.92 to 0.45)	-0.15 (-0.37 to 0.06)	-0.11 (-0.49 to 0.27)	**Haloperidol**	-0.27 (-1.01 to 0.46)	NA	NA	0.14 (-0.29 to 0.57)
-0.67 (-1.60 to 0.27)	-0.59 (-1.39 to 0.22)	-0.40 (-0.95 to 0.14)	-0.35 (-0.90 to 0.19)	-0.33 (-1.18 to 0.53)	-0.24 (-0.79 to 0.30)	-0.20 (-0.84 to 0.43)	-0.09 (-0.62 to 0.44)	**Fluphenazine**	NA	NA	-0.15 (-1.01 to 0.72)
-0.74 (-1.65 to 0.16)	-0.67 (-1.43 to 0.09)	**-0.48 (-0.95 to -0.01)**	-0.43 (-0.91 to 0.05)	-0.41 (-1.22 to 0.41)	-0.32 (-0.75 to 0.11)	-0.28 (-0.86 to 0.30)	-0.17 (-0.65 to 0.31)	-0.08 (-0.77 to 0.62)	**Sertindole**	NA	NA
-0.70 (-1.45 to 0.05)	-0.63 (-1.27 to 0.01)	**-0.44 (-0.66 to -0.22)**	**-0.39 (-0.64 to -0.14)**	-0.36 (-1.06 to 0.33)	**-0.28 (-0.55 to -0.01)**	-0.24 (-0.58 to 0.09)	-0.13 (-0.39 to 0.14)	-0.04 (-0.60 to 0.53)	0.04 (-0.47 to 0.55)	**Chlorpromazine**	-0.22 (-0.53 to 0.10)
-0.71 (-1.50 to 0.08)	-0.63 (-1.28 to 0.01)	**-0.45 (-0.71 to -0.19)**	**-0.40 (-0.67 to -0.13)**	-0.37 (-1.08 to 0.34)	-0.29 (-0.58 to 0.00)	-0.25 (-0.64 to 0.15)	-0.13 (-0.40 to 0.13)	-0.04 (-0.60 to 0.51)	0.03 (-0.49 to 0.56)	-0.01 (-0.26 to 0.24)	**Quetiapine**

Treatments are presented in order of efficacy ranking. Results of the network meta-analysis are reported in the left lower half and results of pairwise meta-analyses in the right upper half. Each cell provides the effect estimate and the corresponding 95% credible interval (95% CI) of a comparison (left lower half: treatment in column versus treatment in row; right upper half: treatment in row versus treatment in column). The type of effect size measure is standardized mean difference (SMD). Bold results indicate 95% CI excluding no effect. NA=not available.

11.6 Excluding studies at high risk of bias

Number of studies: k = 23

Number of pairwise comparisons: m = 28

Number of treatments: n = 10

Number of designs: d = 15

Network plot

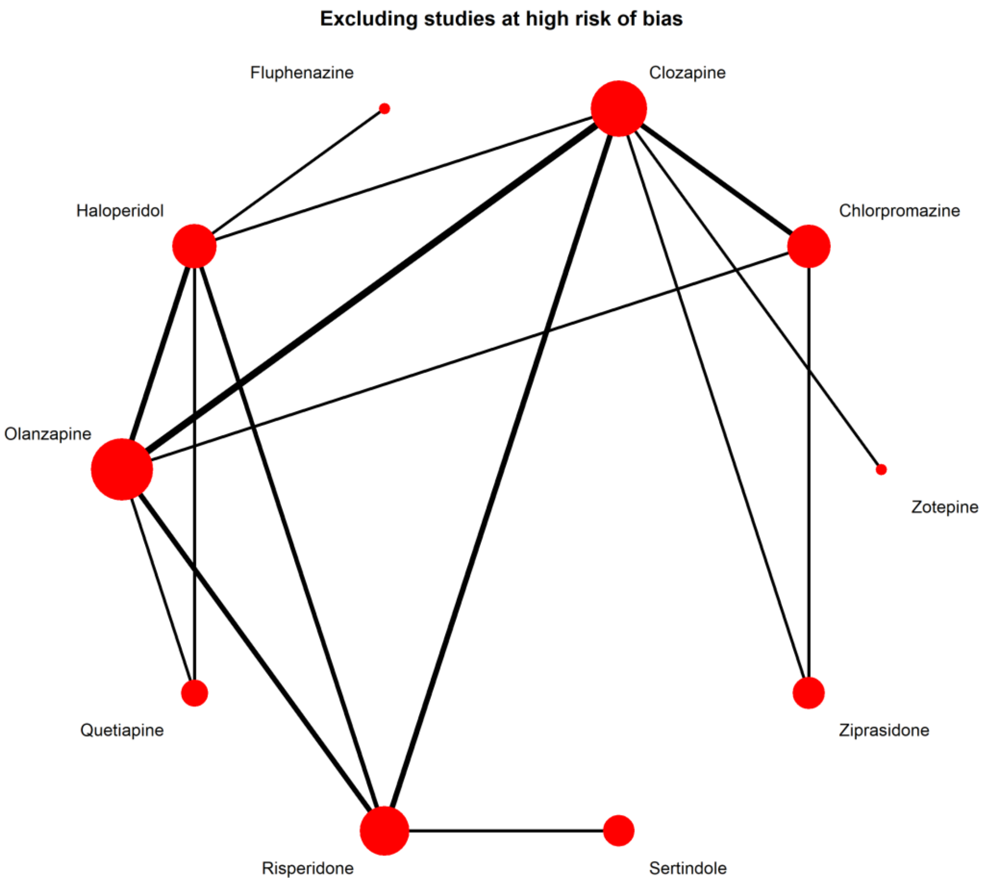

Lines link treatments with direct comparisons in trials; thickness of lines corresponds to the number of trials evaluating the comparison; size of the nodes corresponds to the number of participants assigned to the treatment.

τ	P-value	Inconsistency loop (%)
0.1655	0.0093	25.0

Forest-plot of results of network meta-analysis for antipsychotic drugs versus clozapine

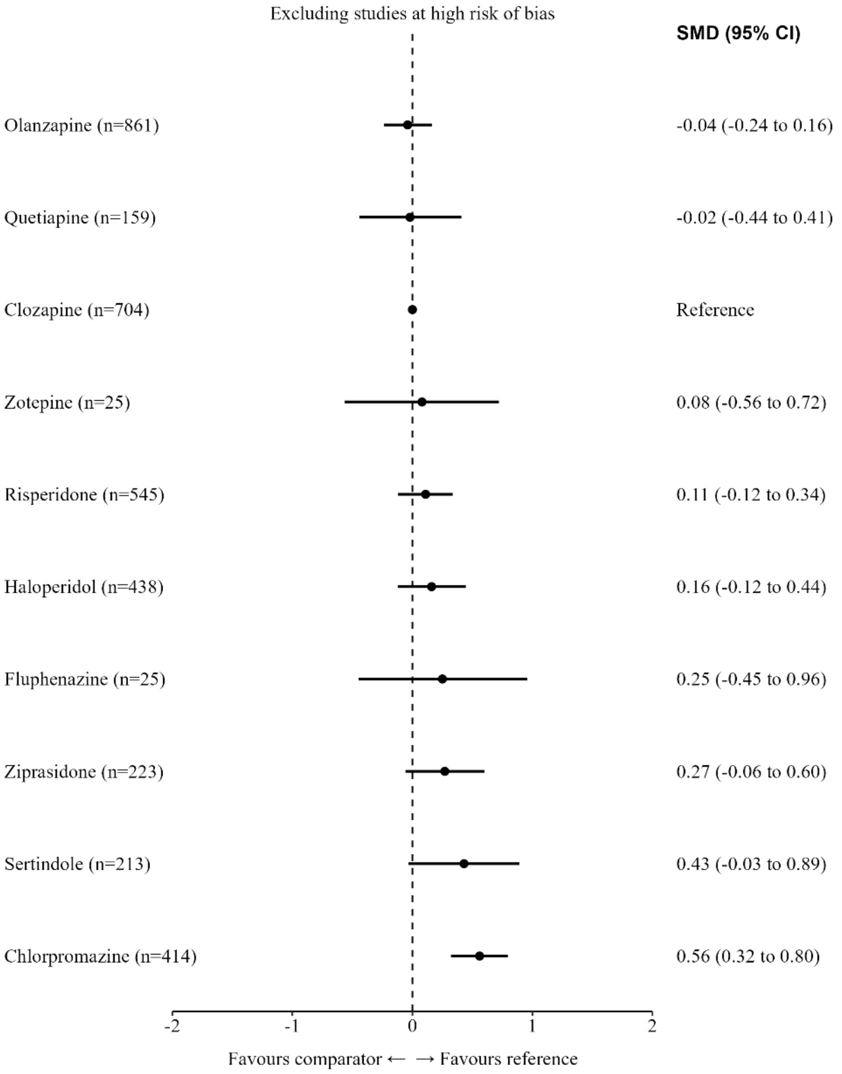

Effect sizes, measured as standardized mean difference (SMD), are from the network meta-analysis. Order of treatments is according to the mean effect size. Reference is clozapine. The direction of the effect is indicated below the x-axis.

League table

**Olanzapine**	-0.11 (-0.36 to 0.14)	0.09 (-0.61 to 0.79)	NA	-0.22 (-0.51 to 0.07)	-0.24 (-0.51 to 0.04)	NA	NA	NA	-0.22 (-0.76 to 0.33)
-0.04 (-0.24 to 0.16)	**Clozapine**	NA	-0.08 (-0.72 to 0.56)	-0.04 (-0.32 to 0.24)	0.12 (-0.44 to 0.67)	NA	0.02 (-0.44 to 0.48)	NA	**-0.78 (-1.07 to -0.49)**
-0.02 (-0.41 to 0.37)	0.02 (-0.41 to 0.44)	**Quetiapine**	NA	NA	-0.14 (-0.54 to 0.26)	NA	NA	NA	NA
-0.11 (-0.79 to 0.56)	-0.08 (-0.72 to 0.56)	-0.10 (-0.87 to 0.67)	**Zotepine**	NA	NA	NA	NA	NA	NA
-0.14 (-0.37 to 0.08)	-0.11 (-0.34 to 0.12)	-0.13 (-0.55 to 0.30)	-0.03 (-0.71 to 0.65)	**Risperidone**	-0.14 (-0.55 to 0.27)	NA	NA	-0.32 (-0.72 to 0.08)	NA
-0.20 (-0.43 to 0.04)	-0.16 (-0.44 to 0.12)	-0.18 (-0.53 to 0.17)	-0.08 (-0.79 to 0.62)	-0.05 (-0.34 to 0.23)	**Haloperidol**	-0.09 (-0.74 to 0.55)	NA	NA	NA
-0.29 (-0.97 to 0.39)	-0.25 (-0.96 to 0.45)	-0.27 (-1.00 to 0.46)	-0.18 (-1.13 to 0.78)	-0.15 (-0.85 to 0.56)	-0.09 (-0.74 to 0.55)	**Fluphenazine**	NA	NA	NA
-0.31 (-0.68 to 0.07)	-0.27 (-0.60 to 0.06)	-0.29 (-0.82 to 0.24)	-0.19 (-0.92 to 0.53)	-0.16 (-0.56 to 0.23)	-0.11 (-0.54 to 0.32)	-0.02 (-0.79 to 0.75)	**Ziprasidone**	NA	-0.07 (-0.47 to 0.32)
-0.46 (-0.92 to 0.00)	-0.43 (-0.89 to 0.03)	-0.45 (-1.03 to 0.14)	-0.35 (-1.14 to 0.44)	-0.32 (-0.72 to 0.08)	-0.27 (-0.76 to 0.22)	-0.17 (-0.98 to 0.63)	-0.16 (-0.72 to 0.41)	**Sertindole**	NA
**-0.59 (-0.88 to -0.31)**	**-0.56 (-0.80 to -0.32)**	**-0.58 (-1.05 to -0.10)**	-0.48 (-1.16 to 0.21)	**-0.45 (-0.77 to -0.13)**	**-0.40 (-0.75 to -0.04)**	-0.30 (-1.04 to 0.43)	-0.29 (-0.60 to 0.03)	-0.13 (-0.64 to 0.38)	**Chlorpromazine**

Treatments are presented in order of efficacy ranking. Results of the network meta-analysis are reported in the left lower half and results of pairwise meta-analyses in the right upper half. Each cell provides the effect estimate and the corresponding 95% credible interval (95% CI) of a comparison (left lower half: treatment in column versus treatment in row; right upper half: treatment in row versus treatment in column). The type of effect size measure is standardized mean difference (SMD). Bold results indicate 95% CI excluding no effect. NA=not available.

11.7 Excluding studies that only included children and/or adolescents

Number of studies: k = 40

Number of pairwise comparisons: m = 52

Number of treatments: n = 12

Number of designs: d = 21

Network plot

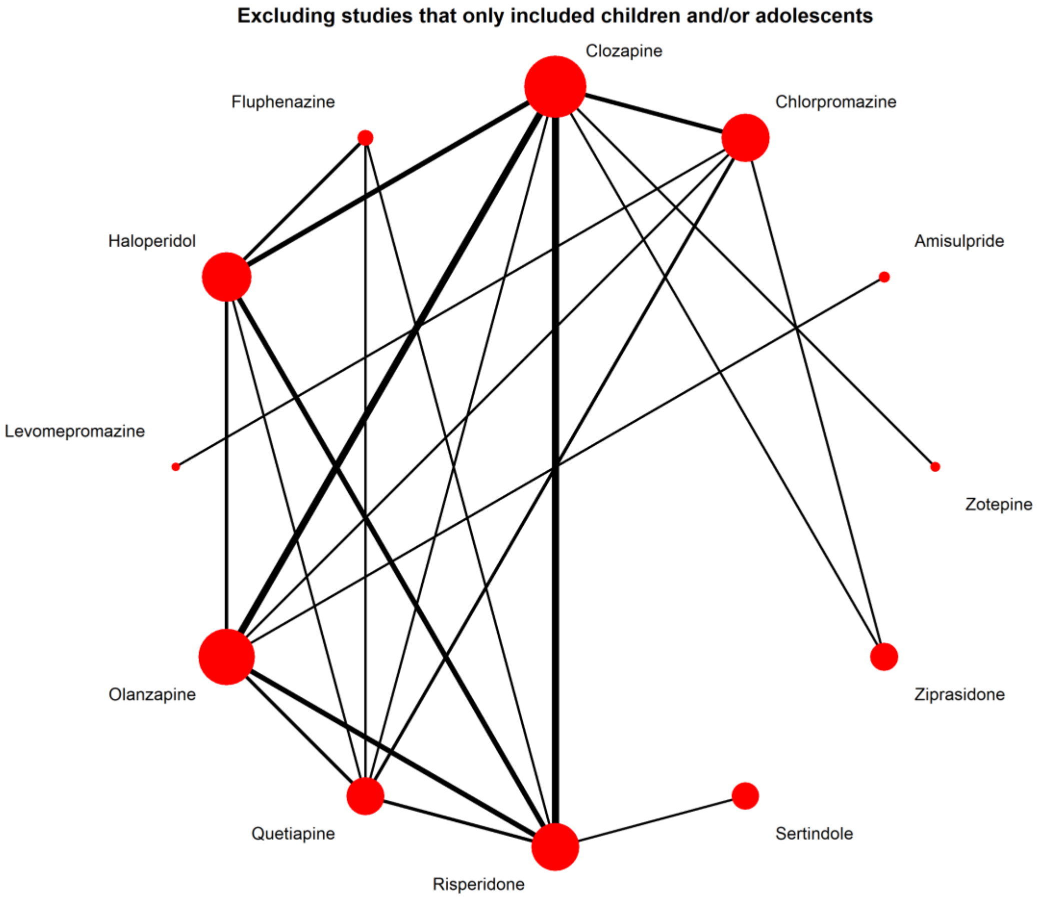

Lines link treatments with direct comparisons in trials; thickness of lines corresponds to the number of trials evaluating the comparison; size of the nodes corresponds to the number of participants assigned to the treatment.

τ	P-value	Inconsistency loop (%)
0.1874	< 0.0001	16.67

Forest-plot of results of network meta-analysis for antipsychotic drugs versus clozapine

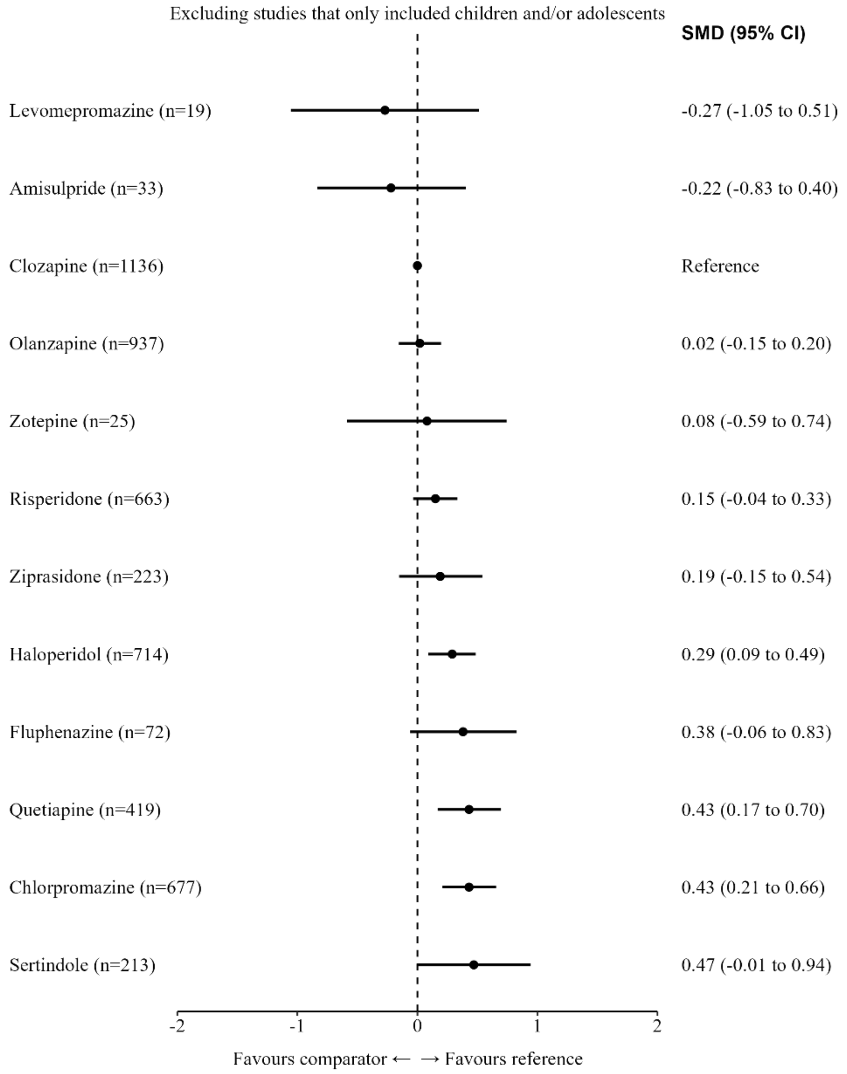

Effect sizes, measured as standardized mean difference (SMD), are from the network meta-analysis. Order of treatments is according to the mean effect size. Reference is clozapine. The direction of the effect is indicated below the x-axis.

League table

**Amisulpride**	NA	NA	-0.24 (-0.83 to 0.36)	NA	NA	NA	NA	NA	NA	NA	NA
0.05 (-0.94 to 1.04)	**Levomepromazine**	NA	NA	NA	NA	NA	NA	NA	NA	NA	-0.70 (-1.45 to 0.05)
-0.22 (-0.83 to 0.40)	-0.27 (-1.05 to 0.51)	**Clozapine**	0.03 (-0.19 to 0.25)	-0.08 (-0.74 to 0.59)	-0.08 (-0.30 to 0.15)	0.02 (-0.47 to 0.51)	-0.13 (-0.41 to 0.14)	NA	NA	**-1.01 (-1.89 to -0.13)**	**-0.78 (-1.08 to -0.47)**
-0.24 (-0.83 to 0.36)	-0.29 (-1.08 to 0.50)	-0.02 (-0.20 to 0.15)	**Olanzapine**	NA	-0.23 (-0.53 to 0.06)	NA	-0.30 (-0.64 to 0.04)	NA	NA	-0.07 (-0.65 to 0.52)	-0.22 (-0.79 to 0.36)
-0.29 (-1.20 to 0.61)	-0.35 (-1.38 to 0.68)	-0.08 (-0.74 to 0.59)	-0.06 (-0.75 to 0.63)	**Zotepine**	NA	NA	NA	NA	NA	NA	NA
-0.36 (-0.99 to 0.26)	-0.42 (-1.22 to 0.38)	-0.15 (-0.33 to 0.04)	-0.13 (-0.34 to 0.08)	-0.07 (-0.76 to 0.62)	**Risperidone**	NA	-0.15 (-0.49 to 0.19)	0.02 (-0.83 to 0.87)	-0.32 (-0.76 to 0.12)	-0.06 (-0.74 to 0.63)	NA
-0.41 (-1.11 to 0.29)	-0.47 (-1.29 to 0.36)	-0.19 (-0.54 to 0.15)	-0.17 (-0.55 to 0.20)	-0.12 (-0.87 to 0.63)	-0.05 (-0.43 to 0.34)	**Ziprasidone**	NA	NA	NA	NA	-0.07 (-0.50 to 0.36)
-0.50 (-1.14 to 0.13)	-0.56 (-1.36 to 0.24)	**-0.29 (-0.49 to -0.09)**	**-0.27 (-0.48 to -0.05)**	-0.21 (-0.90 to 0.48)	-0.14 (-0.36 to 0.08)	-0.09 (-0.48 to 0.29)	**Haloperidol**	-0.17 (-0.67 to 0.32)	NA	0.14 (-0.29 to 0.58)	NA
-0.60 (-1.34 to 0.15)	-0.65 (-1.54 to 0.24)	-0.38 (-0.83 to 0.06)	-0.36 (-0.81 to 0.09)	-0.30 (-1.10 to 0.50)	-0.23 (-0.68 to 0.21)	-0.19 (-0.74 to 0.37)	-0.09 (-0.51 to 0.32)	**Fluphenazine**	NA	-0.15 (-1.01 to 0.72)	NA
-0.69 (-1.45 to 0.08)	-0.74 (-1.65 to 0.17)	-0.47 (-0.94 to 0.01)	-0.45 (-0.93 to 0.04)	-0.39 (-1.21 to 0.43)	-0.32 (-0.76 to 0.12)	-0.27 (-0.86 to 0.31)	-0.18 (-0.67 to 0.31)	-0.09 (-0.71 to 0.54)	**Sertindole**	NA	NA
-0.65 (-1.30 to 0.01)	-0.70 (-1.50 to 0.09)	**-0.43 (-0.70 to -0.17)**	**-0.41 (-0.69 to -0.13)**	-0.35 (-1.07 to 0.36)	-0.28 (-0.58 to 0.01)	-0.24 (-0.64 to 0.16)	-0.14 (-0.42 to 0.13)	-0.05 (-0.52 to 0.42)	0.04 (-0.49 to 0.56)	**Quetiapine**	0.22 (-0.10 to 0.53)
-0.65 (-1.29 to 0.00)	-0.70 (-1.45 to 0.05)	**-0.43 (-0.66 to -0.21)**	**-0.41 (-0.67 to -0.16)**	-0.35 (-1.06 to 0.35)	**-0.28 (-0.56 to -0.01)**	-0.24 (-0.58 to 0.10)	-0.14 (-0.41 to 0.13)	-0.05 (-0.52 to 0.43)	0.04 (-0.48 to 0.55)	0.00 (-0.25 to 0.25)	**Chlorpromazine**

Treatments are presented in order of efficacy ranking. Results of the network meta-analysis are reported in the left lower half and results of pairwise meta-analyses in the right upper half. Each cell provides the effect estimate and the corresponding 95% credible interval (95% CI) of a comparison (left lower half: treatment in column versus treatment in row; right upper half: treatment in row versus treatment in column). The type of effect size measure is standardized mean difference (SMD). Bold results indicate 95% CI excluding no effect. NA=not available.

11.8 Excluding low clozapine doses (<400 mg)

Number of studies: k = 32

Number of pairwise comparisons: m = 39

Number of treatments: n = 11

Number of designs: d = 18

Network plot

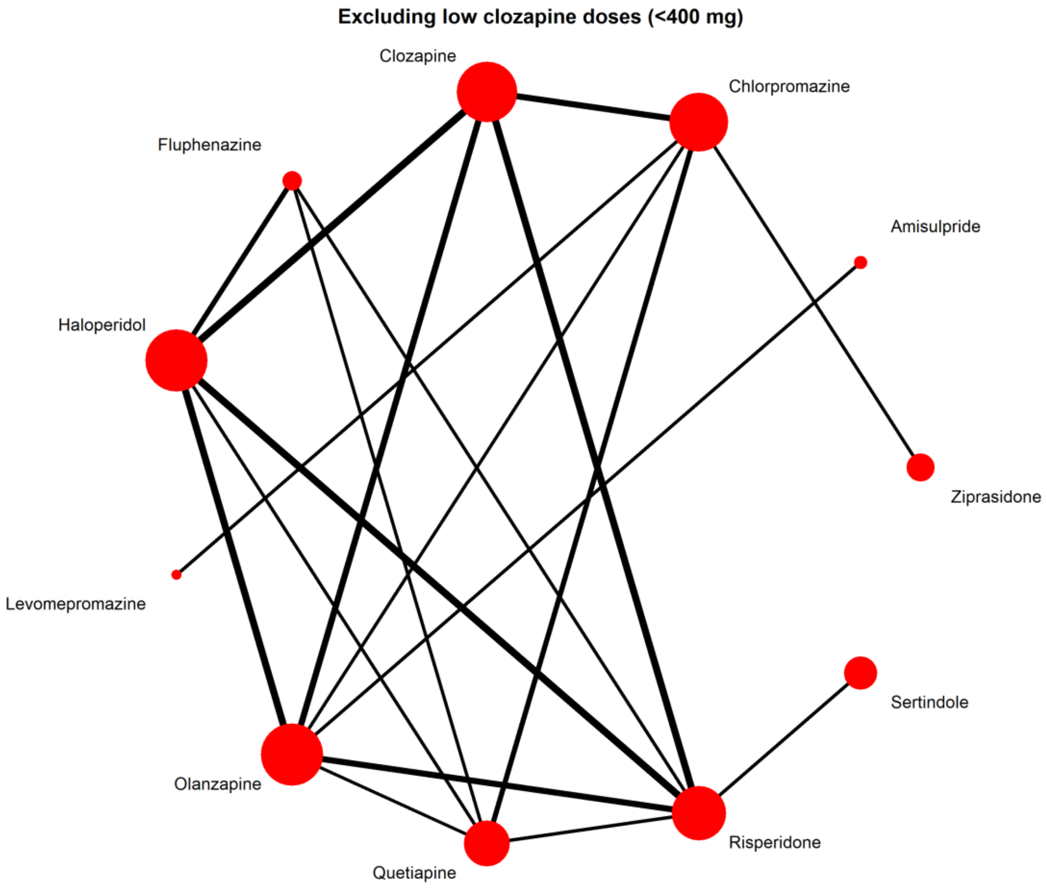

Lines link treatments with direct comparisons in trials; thickness of lines corresponds to the number of trials evaluating the comparison; size of the nodes corresponds to the number of participants assigned to the treatment.

τ	P-value	Inconsistency loop (%)
0.1786	0.0003	26.67

Forest-plot of results of network meta-analysis for antipsychotic drugs versus clozapine

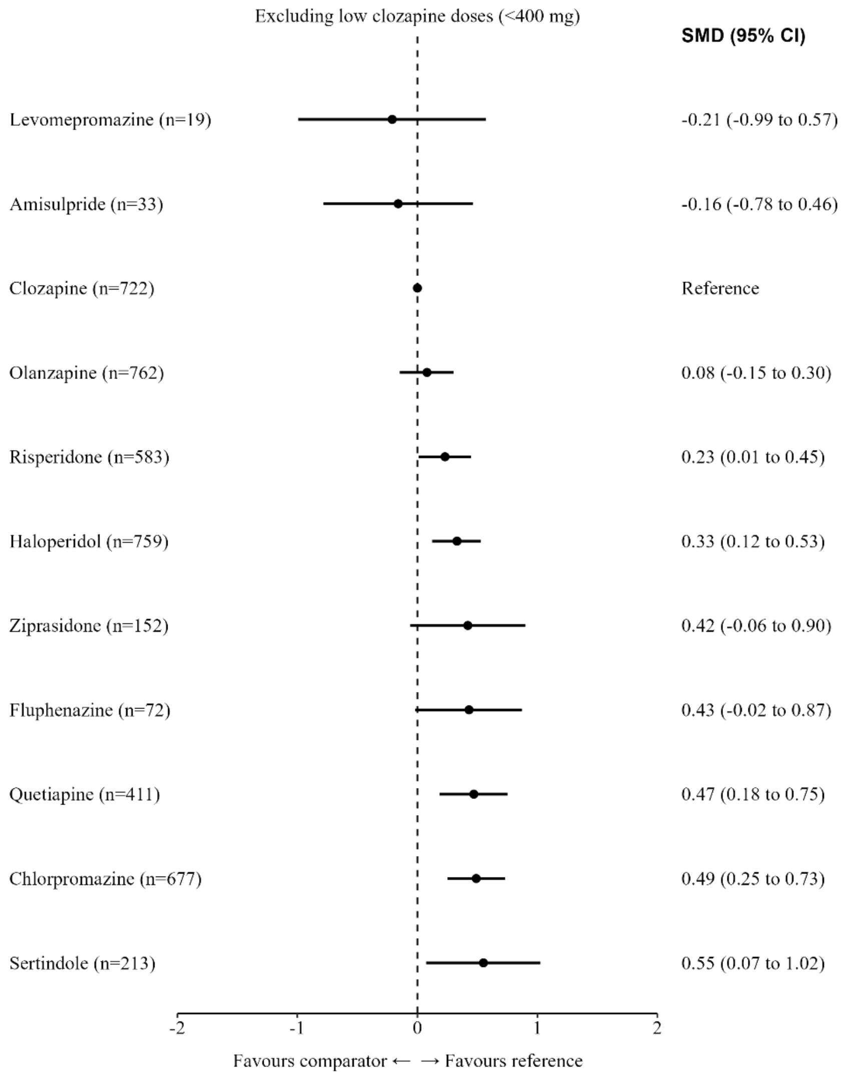

Effect sizes, measured as standardized mean difference (SMD), are from the network meta-analysis. Order of treatments is according to the mean effect size. Reference is clozapine. The direction of the effect is indicated below the x-axis.

League table

**Amisulpride**	NA	NA	-0.24 (-0.82 to 0.35)	NA	NA	NA	NA	NA	NA	NA
0.05 (-0.93 to 1.03)	**Levomepromazine**	NA	NA	NA	NA	NA	NA	NA	-0.70 (-1.45 to 0.04)	NA
-0.16 (-0.78 to 0.46)	-0.21 (-0.99 to 0.57)	**Clozapine**	0.15 (-0.22 to 0.53)	-0.13 (-0.41 to 0.15)	-0.14 (-0.40 to 0.13)	NA	NA	NA	**-0.78 (-1.07 to -0.48)**	NA
-0.24 (-0.82 to 0.35)	-0.29 (-1.08 to 0.50)	-0.08 (-0.30 to 0.15)	**Olanzapine**	-0.22 (-0.52 to 0.09)	**-0.28 (-0.55 to -0.01)**	NA	NA	0.09 (-0.62 to 0.81)	-0.22 (-0.78 to 0.34)	NA
-0.39 (-1.01 to 0.24)	-0.44 (-1.24 to 0.36)	**-0.23 (-0.45 to -0.01)**	-0.15 (-0.38 to 0.07)	**Risperidone**	-0.15 (-0.48 to 0.19)	NA	0.02 (-0.82 to 0.87)	-0.12 (-0.98 to 0.74)	NA	-0.32 (-0.74 to 0.10)
-0.49 (-1.10 to 0.13)	-0.54 (-1.33 to 0.25)	**-0.33 (-0.53 to -0.12)**	**-0.25 (-0.46 to -0.04)**	-0.10 (-0.32 to 0.13)	**Haloperidol**	NA	-0.17 (-0.66 to 0.31)	0.14 (-0.28 to 0.56)	NA	NA
-0.58 (-1.35 to 0.19)	-0.63 (-1.48 to 0.22)	-0.42 (-0.90 to 0.06)	-0.34 (-0.84 to 0.16)	-0.19 (-0.70 to 0.32)	-0.09 (-0.59 to 0.40)	**Ziprasidone**	NA	NA	-0.07 (-0.49 to 0.34)	NA
-0.59 (-1.32 to 0.15)	-0.64 (-1.52 to 0.24)	-0.43 (-0.87 to 0.02)	-0.35 (-0.80 to 0.10)	-0.20 (-0.64 to 0.25)	-0.10 (-0.51 to 0.31)	-0.01 (-0.64 to 0.62)	**Fluphenazine**	-0.15 (-1.01 to 0.71)	NA	NA
-0.63 (-1.28 to 0.02)	-0.68 (-1.46 to 0.11)	**-0.47 (-0.75 to -0.18)**	**-0.39 (-0.68 to -0.10)**	-0.24 (-0.55 to 0.07)	-0.14 (-0.42 to 0.13)	-0.05 (-0.53 to 0.44)	-0.04 (-0.51 to 0.43)	**Quetiapine**	0.22 (-0.09 to 0.52)	NA
**-0.65 (-1.30 to -0.01)**	-0.70 (-1.45 to 0.04)	**-0.49 (-0.73 to -0.25)**	**-0.41 (-0.69 to -0.14)**	-0.26 (-0.56 to 0.03)	-0.17 (-0.44 to 0.10)	-0.07 (-0.49 to 0.34)	-0.06 (-0.54 to 0.41)	-0.02 (-0.28 to 0.23)	**Chlorpromazine**	NA
-0.71 (-1.46 to 0.05)	-0.76 (-1.66 to 0.14)	**-0.55 (-1.02 to -0.07)**	-0.47 (-0.95 to 0.01)	-0.32 (-0.74 to 0.10)	-0.22 (-0.70 to 0.25)	-0.13 (-0.79 to 0.53)	-0.12 (-0.74 to 0.49)	-0.08 (-0.61 to 0.44)	-0.06 (-0.57 to 0.46)	**Sertindole**

Treatments are presented in order of efficacy ranking. Results of the network meta-analysis are reported in the left lower half and results of pairwise meta-analyses in the right upper half. Each cell provides the effect estimate and the corresponding 95% credible interval (95% CI) of a comparison (left lower half: treatment in column versus treatment in row; right upper half: treatment in row versus treatment in column). The type of effect size measure is standardized mean difference (SMD). Bold results indicate 95% CI excluding no effect. NA=not available.

11.9 Excluding studies from clozapine's manufacturer

Number of studies: k = 41

Number of pairwise comparisons: m = 53

Number of treatments: n = 12

Number of designs: d = 21

Network plot

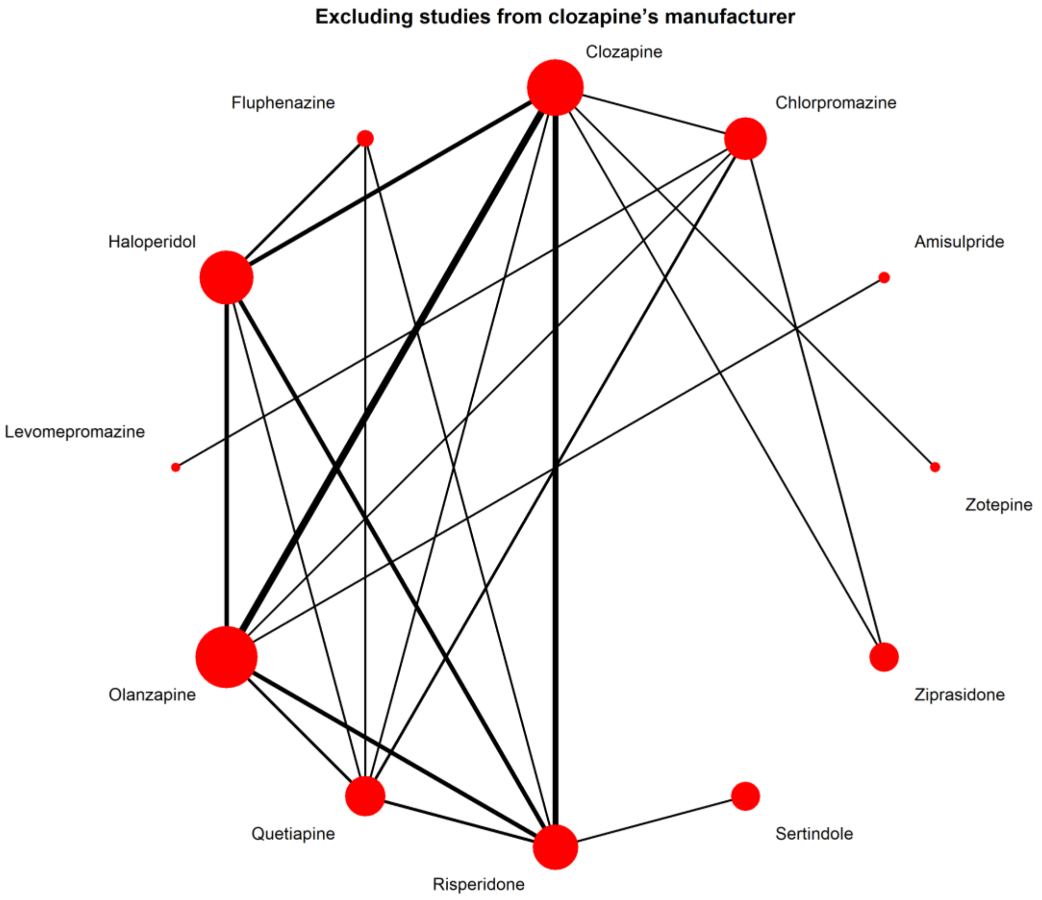

Lines link treatments with direct comparisons in trials; thickness of lines corresponds to the number of trials evaluating the comparison; size of the nodes corresponds to the number of participants assigned to the treatment.

τ	P-value	Inconsistency loop (%)
**0.0768**	**0.0804**	**16.67**

Forest-plot of results of network meta-analysis for antipsychotic drugs versus clozapine

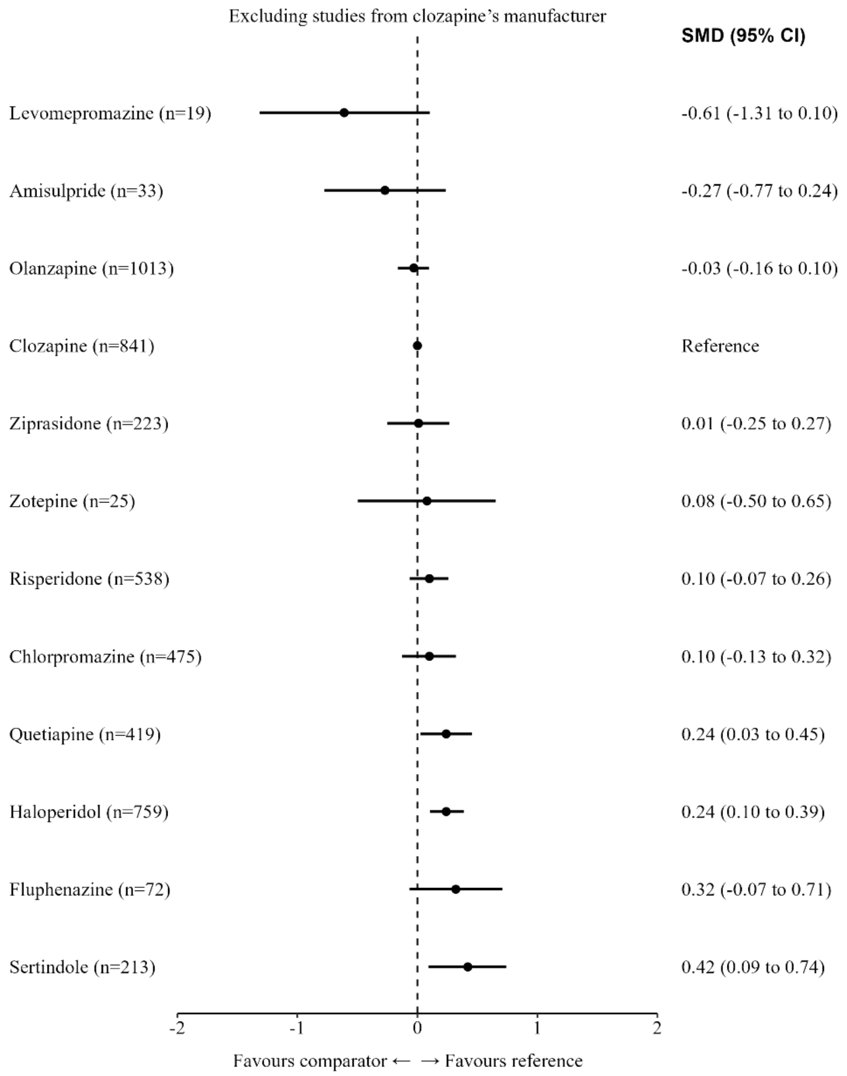

Effect sizes, measured as standardized mean difference (SMD), are from the network meta-analysis. Order of treatments is according to the mean effect size. Reference is clozapine. The direction of the effect is indicated below the x-axis.

League table

**Levomepromazine**	NA	NA	NA	NA	NA	**-0.70 (-1.37 to -0.03)**	NA	NA	NA	NA	NA
-0.34 (-1.20 to 0.52)	**Amisulpride**	-0.24 (-0.73 to 0.25)	NA	NA	NA	NA	NA	NA	NA	NA	NA
-0.57 (-1.28 to 0.14)	-0.24 (-0.73 to 0.25)	**Olanzapine**	-0.02 (-0.19 to 0.14)	NA	NA	-0.22 (-0.68 to 0.25)	**-0.23 (-0.44 to -0.02)**	-0.05 (-0.58 to 0.48)	**-0.27 (-0.46 to -0.08)**	NA	NA
-0.61 (-1.31 to 0.10)	-0.27 (-0.77 to 0.24)	-0.03 (-0.16 to 0.10)	**Clozapine**	0.02 (-0.34 to 0.38)	-0.08 (-0.65 to 0.50)	**-0.76 (-1.42 to -0.10)**	0.04 (-0.19 to 0.26)	**-1.01 (-1.82 to -0.20)**	-0.18 (-0.37 to 0.01)	NA	NA
-0.61 (-1.32 to 0.10)	-0.28 (-0.84 to 0.28)	-0.04 (-0.31 to 0.23)	-0.01 (-0.27 to 0.25)	**Ziprasidone**	NA	-0.07 (-0.34 to 0.20)	NA	NA	NA	NA	NA
-0.68 (-1.60 to 0.23)	-0.35 (-1.11 to 0.42)	-0.11 (-0.70 to 0.48)	-0.08 (-0.65 to 0.50)	-0.07 (-0.70 to 0.56)	**Zotepine**	NA	NA	NA	NA	NA	NA
**-0.70 (-1.37 to -0.03)**	-0.37 (-0.90 to 0.17)	-0.13 (-0.36 to 0.10)	-0.10 (-0.32 to 0.13)	-0.09 (-0.32 to 0.14)	-0.02 (-0.64 to 0.60)	**Chlorpromazine**	NA	**-0.22 (-0.42 to -0.01)**	NA	NA	NA
-0.70 (-1.42 to 0.02)	-0.37 (-0.88 to 0.15)	-0.13 (-0.29 to 0.03)	-0.10 (-0.26 to 0.07)	-0.09 (-0.38 to 0.20)	-0.02 (-0.62 to 0.58)	-0.00 (-0.25 to 0.25)	**Risperidone**	-0.06 (-0.70 to 0.58)	-0.14 (-0.43 to 0.15)	0.02 (-0.76 to 0.81)	**-0.32 (-0.60 to -0.04)**
**-0.85 (-1.54 to -0.15)**	-0.51 (-1.04 to 0.02)	**-0.27 (-0.49 to -0.06)**	**-0.24 (-0.45 to -0.03)**	-0.23 (-0.50 to 0.04)	-0.16 (-0.78 to 0.45)	-0.14 (-0.33 to 0.04)	-0.14 (-0.38 to 0.09)	**Quetiapine**	-0.14 (-0.42 to 0.14)	0.15 (-0.65 to 0.95)	NA
**-0.85 (-1.56 to -0.14)**	**-0.51 (-1.02 to -0.01)**	**-0.28 (-0.42 to -0.14)**	**-0.24 (-0.39 to -0.10)**	-0.24 (-0.51 to 0.04)	-0.17 (-0.76 to 0.42)	-0.15 (-0.38 to 0.08)	-0.15 (-0.32 to 0.02)	-0.00 (-0.21 to 0.20)	**Haloperidol**	-0.17 (-0.60 to 0.26)	NA
**-0.93 (-1.72 to -0.13)**	-0.59 (-1.21 to 0.03)	-0.35 (-0.74 to 0.03)	-0.32 (-0.71 to 0.07)	-0.31 (-0.76 to 0.14)	-0.24 (-0.94 to 0.45)	-0.22 (-0.64 to 0.20)	-0.22 (-0.62 to 0.17)	-0.08 (-0.48 to 0.32)	-0.08 (-0.44 to 0.29)	**Fluphenazine**	NA
**-1.02 (-1.79 to -0.25)**	**-0.69 (-1.27 to -0.10)**	**-0.45 (-0.77 to -0.13)**	**-0.42 (-0.74 to -0.09)**	-0.41 (-0.81 to 0.00)	-0.34 (-1.00 to 0.32)	-0.32 (-0.70 to 0.06)	**-0.32 (-0.60 to -0.04)**	-0.18 (-0.55 to 0.19)	-0.17 (-0.50 to 0.16)	-0.10 (-0.58 to 0.39)	**Sertindole**

Treatments are presented in order of efficacy ranking. Results of the network meta-analysis are reported in the left lower half and results of pairwise meta-analyses in the right upper half. Each cell provides the effect estimate and the corresponding 95% credible interval (95% CI) of a comparison (left lower half: treatment in column versus treatment in row; right upper half: treatment in row versus treatment in column). The type of effect size measure is standardized mean difference (SMD). Bold results indicate 95% CI excluding no effect. NA=not available.

11.10 Excluding studies from olanzapine's manufacturer

Number of studies: k = 35

Number of pairwise comparisons: m = 42

Number of treatments: n = 12

Number of designs: d = 19

Network plot

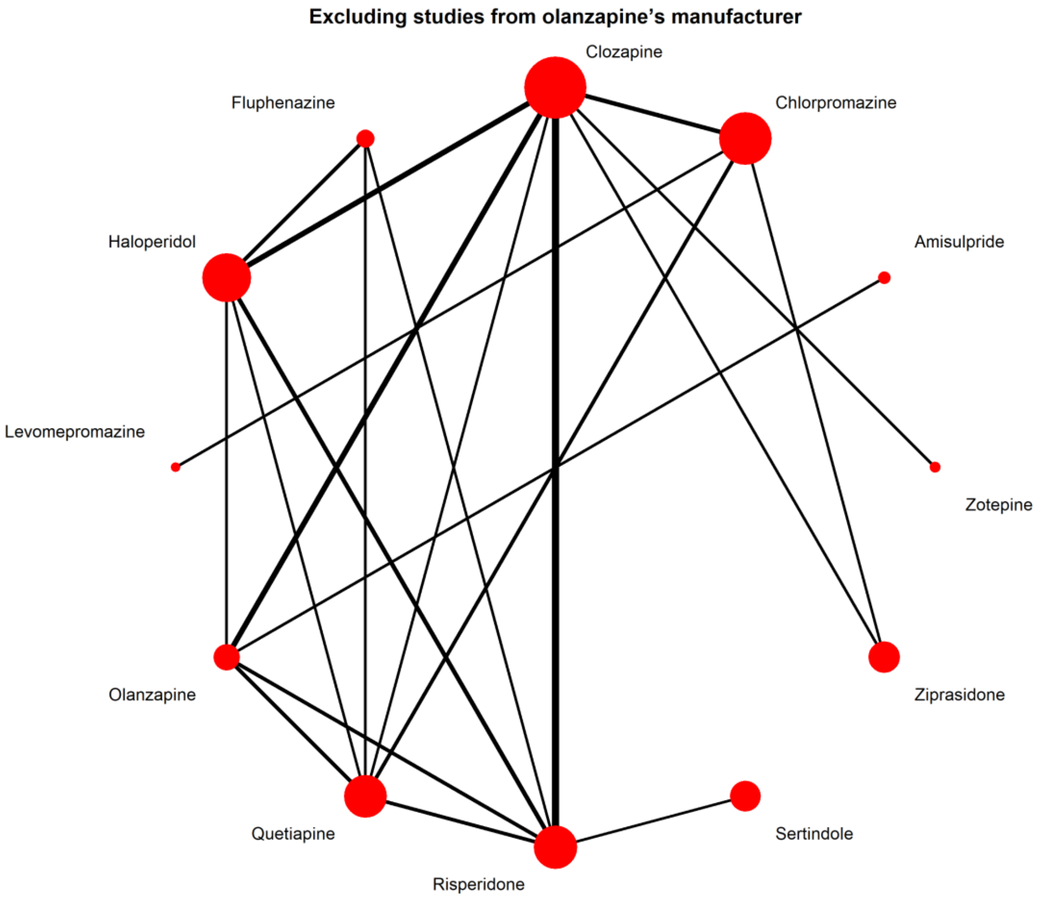

Lines link treatments with direct comparisons in trials; thickness of lines corresponds to the number of trials evaluating the comparison; size of the nodes corresponds to the number of participants assigned to the treatment.

τ	P-value	Inconsistency loop (%)
0.2106	0.0004	11.76

Forest-plot of results of network meta-analysis for antipsychotic drugs versus clozapine

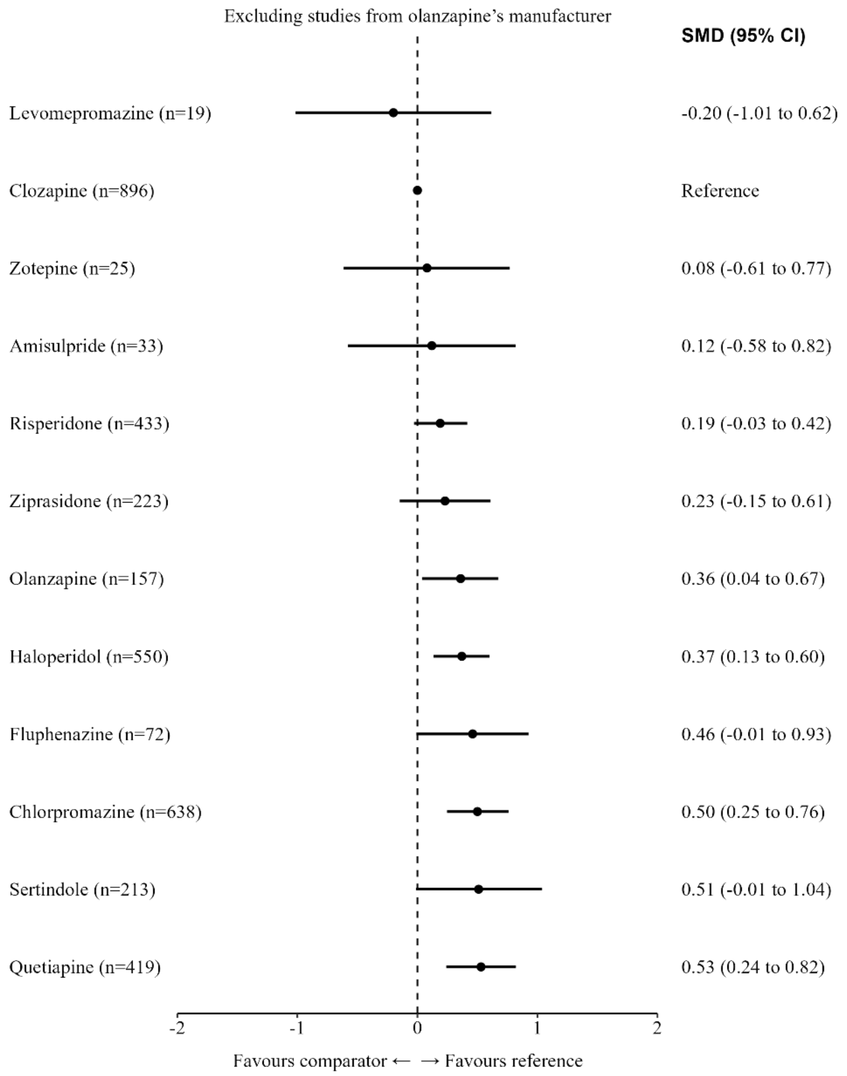

Effect sizes, measured as standardized mean difference (SMD), are from the network meta-analysis. Order of treatments is according to the mean effect size. Reference is clozapine. The direction of the effect is indicated below the x-axis.

League table

**Levomepromazine**	NA	NA	NA	NA	NA	NA	NA	NA	NA	-0.70 (-1.48 to 0.07)	NA
-0.20 (-1.01 to 0.62)	**Clozapine**	-0.08 (-0.77 to 0.61)	NA	-0.13 (-0.40 to 0.13)	0.02 (-0.50 to 0.55)	-0.43 (-0.88 to 0.01)	-0.27 (-0.58 to 0.05)	NA	NA	**-0.77 (-1.10 to -0.45)**	**-1.01 (-1.91 to -0.11)**
-0.28 (-1.35 to 0.79)	-0.08 (-0.77 to 0.61)	**Zotepine**	NA	NA	NA	NA	NA	NA	NA	NA	NA
-0.32 (-1.38 to 0.75)	-0.12 (-0.82 to 0.58)	-0.04 (-1.03 to 0.94)	**Amisulpride**	NA	NA	-0.24 (-0.86 to 0.39)	NA	NA	NA	NA	NA
-0.39 (-1.23 to 0.45)	-0.19 (-0.42 to 0.03)	-0.12 (-0.84 to 0.61)	-0.07 (-0.79 to 0.64)	**Risperidone**	NA	0.25 (-0.40 to 0.90)	-0.17 (-0.61 to 0.27)	0.02 (-0.85 to 0.89)	-0.32 (-0.80 to 0.16)	NA	-0.05 (-0.75 to 0.64)
-0.43 (-1.29 to 0.43)	-0.23 (-0.61 to 0.15)	-0.15 (-0.94 to 0.64)	-0.11 (-0.90 to 0.68)	-0.04 (-0.47 to 0.39)	**Ziprasidone**	NA	NA	NA	NA	-0.07 (-0.54 to 0.40)	NA
-0.56 (-1.42 to 0.31)	**-0.36 (-0.67 to -0.04)**	-0.28 (-1.04 to 0.48)	-0.24 (-0.86 to 0.39)	-0.16 (-0.51 to 0.19)	-0.13 (-0.61 to 0.36)	**Olanzapine**	-0.02 (-0.66 to 0.63)	NA	NA	NA	-0.07 (-0.67 to 0.53)
-0.57 (-1.40 to 0.27)	**-0.37 (-0.60 to -0.13)**	-0.29 (-1.02 to 0.44)	-0.25 (-0.96 to 0.46)	-0.17 (-0.44 to 0.09)	-0.14 (-0.57 to 0.29)	-0.01 (-0.35 to 0.33)	**Haloperidol**	-0.17 (-0.69 to 0.34)	NA	NA	0.14 (-0.33 to 0.62)
-0.66 (-1.58 to 0.27)	-0.46 (-0.93 to 0.01)	-0.38 (-1.22 to 0.45)	-0.34 (-1.16 to 0.48)	-0.27 (-0.74 to 0.21)	-0.23 (-0.82 to 0.36)	-0.10 (-0.63 to 0.43)	-0.09 (-0.52 to 0.34)	**Fluphenazine**	NA	NA	-0.15 (-1.03 to 0.74)
-0.71 (-1.68 to 0.25)	-0.51 (-1.04 to 0.01)	-0.44 (-1.30 to 0.43)	-0.39 (-1.25 to 0.46)	-0.32 (-0.80 to 0.16)	-0.28 (-0.93 to 0.36)	-0.16 (-0.75 to 0.43)	-0.15 (-0.69 to 0.40)	-0.06 (-0.73 to 0.62)	**Sertindole**	NA	NA
-0.70 (-1.48 to 0.07)	**-0.50 (-0.76 to -0.25)**	-0.43 (-1.16 to 0.31)	-0.38 (-1.11 to 0.35)	-0.31 (-0.63 to 0.01)	-0.27 (-0.64 to 0.10)	-0.15 (-0.53 to 0.24)	-0.14 (-0.45 to 0.18)	-0.04 (-0.55 to 0.46)	0.01 (-0.57 to 0.59)	**Chlorpromazine**	-0.22 (-0.56 to 0.13)
-0.73 (-1.55 to 0.09)	**-0.53 (-0.82 to -0.24)**	-0.45 (-1.20 to 0.30)	-0.41 (-1.13 to 0.31)	**-0.34 (-0.67 to -0.01)**	-0.30 (-0.73 to 0.13)	-0.17 (-0.55 to 0.20)	-0.16 (-0.46 to 0.14)	-0.07 (-0.56 to 0.42)	-0.02 (-0.60 to 0.56)	-0.03 (-0.31 to 0.25)	**Quetiapine**

Treatments are presented in order of efficacy ranking. Results of the network meta-analysis are reported in the left lower half and results of pairwise meta-analyses in the right upper half. Each cell provides the effect estimate and the corresponding 95% credible interval (95% CI) of a comparison (left lower half: treatment in column versus treatment in row; right upper half: treatment in row versus treatment in column). The type of effect size measure is standardized mean difference (SMD). Bold results indicate 95% CI excluding no effect. NA=not available.

11.11 Excluding off-label dose olanzapine (>20mg/d)

Number of studies: k = 37

Number of pairwise comparisons: m = 39

Number of treatments: n = 12

Number of designs: d = 18

Network plot

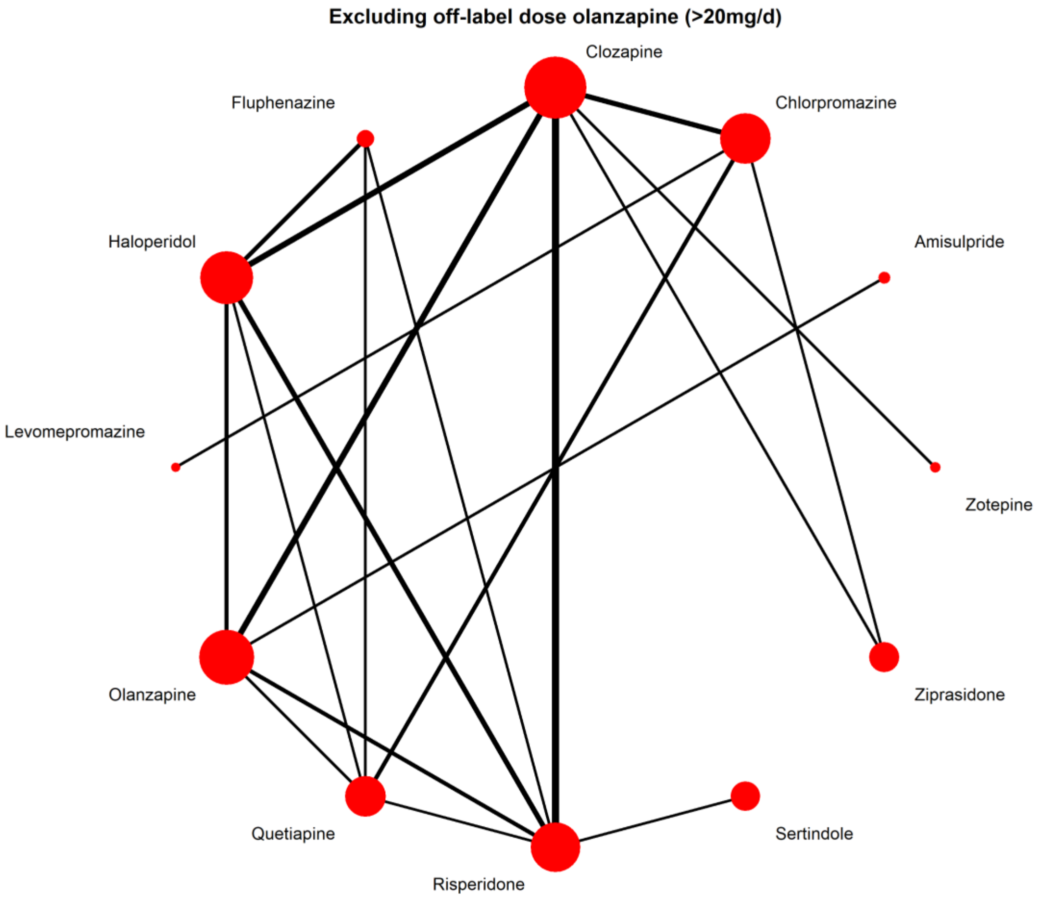

Lines link treatments with direct comparisons in trials; thickness of lines corresponds to the number of trials evaluating the comparison; size of the nodes corresponds to the number of participants assigned to the treatment.

τ	P-value	Inconsistency loop (%)
0.1874	0.0001	12.5

Forest-plot of results of network meta-analysis for antipsychotic drugs versus clozapine

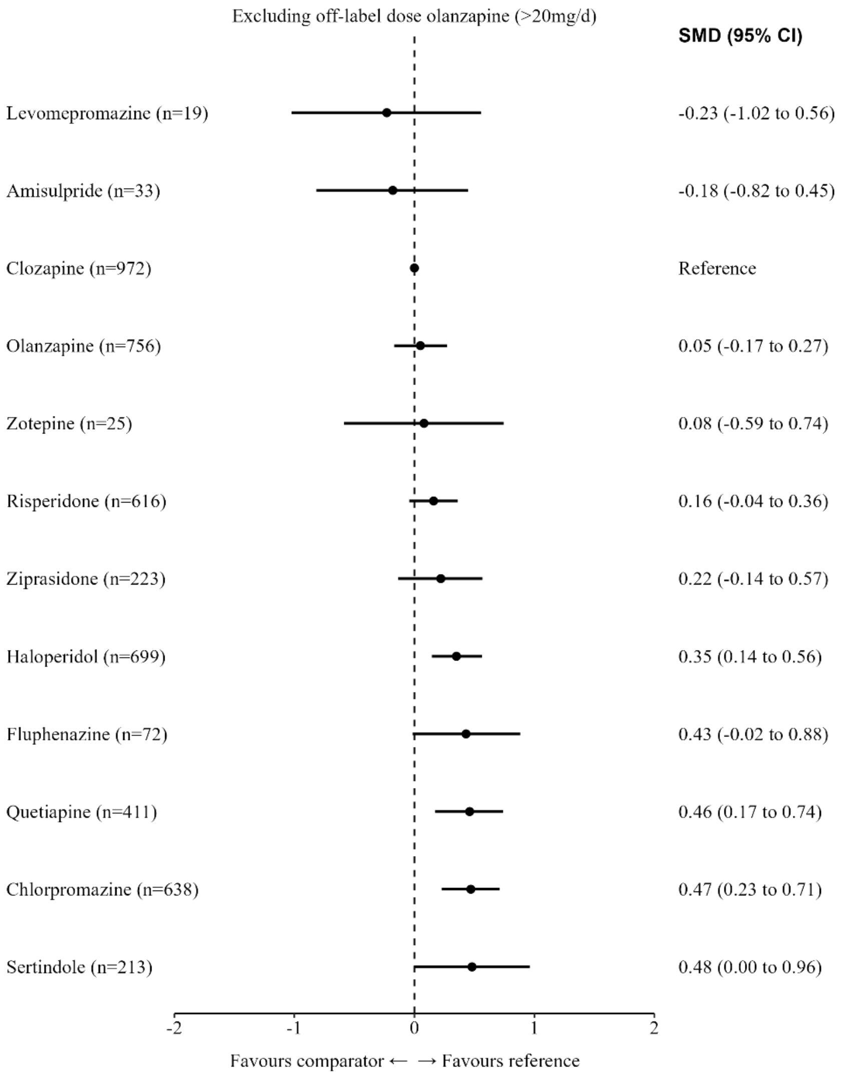

Effect sizes, measured as standardized mean difference (SMD), are from the network meta-analysis. Order of treatments is according to the mean effect size. Reference is clozapine. The direction of the effect is indicated below the x-axis.

League table

**Amisulpride**	NA	NA	-0.24 (-0.83 to 0.36)	NA	NA	NA	NA	NA	NA	NA	NA
0.05 (-0.96 to 1.05)	**Levomepromazine**	NA	NA	NA	NA	NA	NA	NA	NA	NA	-0.70 (-1.45 to 0.05)
-0.18 (-0.82 to 0.45)	-0.23 (-1.02 to 0.56)	**Clozapine**	-0.06 (-0.38 to 0.26)	-0.08 (-0.74 to 0.59)	-0.07 (-0.33 to 0.19)	0.02 (-0.47 to 0.51)	-0.26 (-0.56 to 0.03)	NA	NA	NA	**-0.78 (-1.08 to -0.47)**
-0.24 (-0.83 to 0.36)	-0.29 (-1.10 to 0.53)	-0.05 (-0.27 to 0.17)	**Olanzapine**	NA	-0.21 (-0.58 to 0.16)	NA	-0.35 (-0.72 to 0.03)	NA	NA	0.09 (-0.63 to 0.82)	NA
-0.26 (-1.18 to 0.66)	-0.31 (-1.34 to 0.72)	-0.08 (-0.74 to 0.59)	-0.03 (-0.73 to 0.67)	**Zotepine**	NA	NA	NA	NA	NA	NA	NA
-0.34 (-0.98 to 0.30)	-0.39 (-1.20 to 0.42)	-0.16 (-0.36 to 0.04)	-0.11 (-0.35 to 0.13)	-0.08 (-0.78 to 0.61)	**Risperidone**	NA	-0.16 (-0.59 to 0.26)	0.02 (-0.83 to 0.87)	-0.32 (-0.76 to 0.12)	-0.12 (-0.99 to 0.74)	NA
-0.40 (-1.12 to 0.32)	-0.45 (-1.27 to 0.38)	-0.22 (-0.57 to 0.14)	-0.16 (-0.57 to 0.24)	-0.14 (-0.89 to 0.62)	-0.06 (-0.46 to 0.34)	**Ziprasidone**	NA	NA	NA	NA	-0.07 (-0.50 to 0.36)
-0.54 (-1.18 to 0.10)	-0.59 (-1.39 to 0.22)	**-0.35 (-0.56 to -0.14)**	**-0.30 (-0.54 to -0.06)**	-0.28 (-0.97 to 0.42)	-0.19 (-0.43 to 0.04)	-0.14 (-0.53 to 0.26)	**Haloperidol**	-0.17 (-0.67 to 0.32)	NA	0.14 (-0.29 to 0.58)	NA
-0.62 (-1.37 to 0.14)	-0.67 (-1.56 to 0.23)	-0.43 (-0.88 to 0.02)	-0.38 (-0.85 to 0.08)	-0.35 (-1.16 to 0.45)	-0.27 (-0.73 to 0.18)	-0.22 (-0.78 to 0.34)	-0.08 (-0.50 to 0.34)	**Fluphenazine**	NA	-0.15 (-1.01 to 0.72)	NA
-0.66 (-1.44 to 0.11)	-0.71 (-1.63 to 0.21)	-0.48 (-0.96 to 0.00)	-0.43 (-0.93 to 0.07)	-0.40 (-1.22 to 0.42)	-0.32 (-0.76 to 0.12)	-0.26 (-0.86 to 0.33)	-0.13 (-0.62 to 0.37)	-0.05 (-0.68 to 0.58)	**Sertindole**	NA	NA
-0.64 (-1.31 to 0.03)	-0.69 (-1.49 to 0.11)	**-0.46 (-0.74 to -0.17)**	**-0.40 (-0.72 to -0.09)**	-0.38 (-1.10 to 0.35)	-0.30 (-0.61 to 0.02)	-0.24 (-0.65 to 0.16)	-0.10 (-0.39 to 0.18)	-0.02 (-0.50 to 0.45)	0.02 (-0.52 to 0.56)	**Quetiapine**	0.22 (-0.10 to 0.53)
-0.65 (-1.32 to 0.02)	-0.70 (-1.45 to 0.05)	**-0.47 (-0.71 to -0.23)**	**-0.42 (-0.72 to -0.11)**	-0.39 (-1.10 to 0.32)	**-0.31 (-0.61 to -0.01)**	-0.25 (-0.59 to 0.09)	-0.11 (-0.40 to 0.18)	-0.04 (-0.52 to 0.45)	0.01 (-0.52 to 0.54)	-0.01 (-0.27 to 0.25)	**Chlorpromazine**

Treatments are presented in order of efficacy ranking. Results of the network meta-analysis are reported in the left lower half and results of pairwise meta-analyses in the right upper half. Each cell provides the effect estimate and the corresponding 95% credible interval (95% CI) of a comparison (left lower half: treatment in column versus treatment in row; right upper half: treatment in row versus treatment in column). The type of effect size measure is standardized mean difference (SMD). Bold results indicate 95% CI excluding no effect. NA=not available.

11.12 A most extreme sensitivity analysis including only situations in which clozapine may be most superior

In this sensitivity analysis, open-label studies, studies in which clozapine doses below 400mg/day, studies with intolerant patients and studies with low and intermediate cut-off stringency of criteria for treatment resistance were excluded.

Number of studies: k = 10

Number of pairwise comparisons: m = 12

Number of treatments: n = 10

Number of designs: d = 9

Network plot

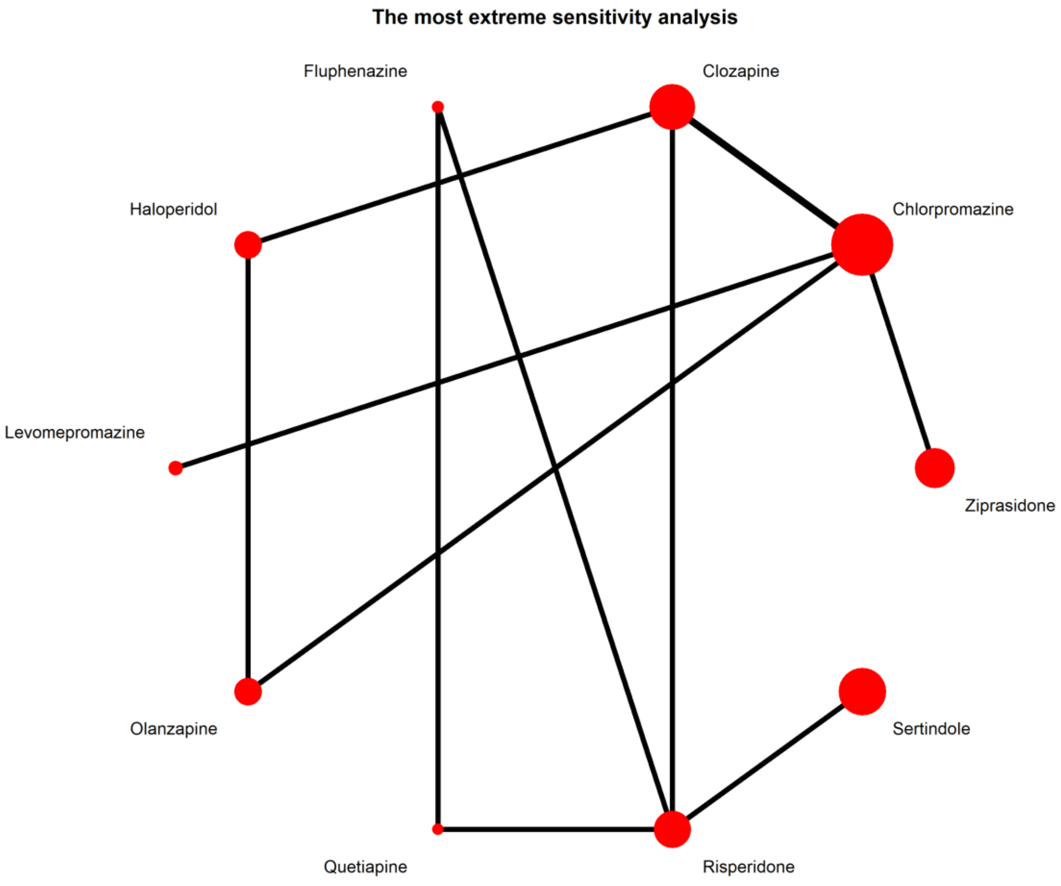

Lines link treatments with direct comparisons in trials; thickness of lines corresponds to the number of trials evaluating the comparison; size of the nodes corresponds to the number of participants assigned to the treatment.

τ	P-value	Inconsistency loop (%)
0	0.2145	0

Forest-plot of results of network meta-analysis for antipsychotic drugs versus clozapine

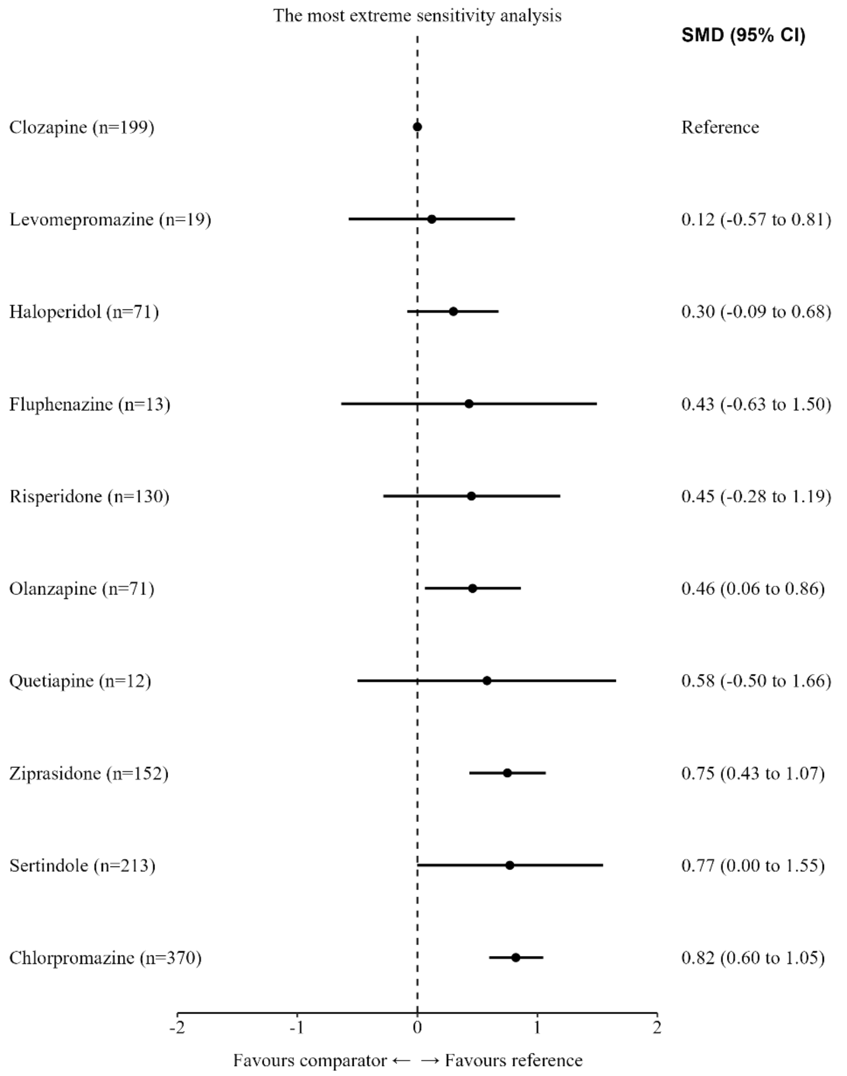

Effect sizes, measured as standardized mean difference (SMD), are from the network meta-analysis. Order of treatments is according to the mean effect size. Reference is clozapine. The direction of the effect is indicated below the x-axis.

League table

**Clozapine**	NA	-0.14 (-0.59 to 0.31)	-0.45 (-1.19 to 0.28)	NA	NA	NA	NA	NA	**-0.86 (-1.10 to -0.63)**
-0.12 (-0.81 to 0.57)	**Levomepromazine**	NA	NA	NA	NA	NA	NA	NA	**-0.70 (-1.36 to -0.05)**
-0.30 (-0.68 to 0.09)	-0.18 (-0.94 to 0.59)	**Haloperidol**	NA	NA	0.02 (-0.48 to 0.51)	NA	NA	NA	NA
-0.45 (-1.19 to 0.28)	-0.33 (-1.35 to 0.68)	-0.16 (-0.99 to 0.67)	**Risperidone**	0.02 (-0.75 to 0.79)	NA	-0.12 (-0.91 to 0.66)	NA	**-0.32 (-0.56 to -0.08)**	NA
-0.43 (-1.50 to 0.63)	-0.31 (-1.58 to 0.96)	-0.14 (-1.27 to 1.00)	0.02 (-0.75 to 0.79)	**Fluphenazine**	NA	-0.15 (-0.93 to 0.64)	NA	NA	NA
**-0.46 (-0.86 to -0.06)**	-0.34 (-1.10 to 0.41)	-0.17 (-0.57 to 0.23)	-0.01 (-0.85 to 0.83)	-0.03 (-1.17 to 1.11)	**Olanzapine**	NA	NA	NA	-0.22 (-0.65 to 0.22)
-0.58 (-1.66 to 0.50)	-0.46 (-1.74 to 0.82)	-0.28 (-1.42 to 0.86)	-0.12 (-0.91 to 0.66)	-0.15 (-0.93 to 0.64)	-0.12 (-1.26 to 1.03)	**Quetiapine**	NA	NA	NA
**-0.75 (-1.07 to -0.43)**	-0.63 (-1.32 to 0.06)	-0.46 (-0.92 to 0.01)	-0.30 (-1.10 to 0.50)	-0.32 (-1.43 to 0.79)	-0.29 (-0.72 to 0.15)	-0.17 (-1.30 to 0.95)	**Ziprasidone**	NA	-0.07 (-0.29 to 0.15)
-0.77 (-1.55 to 0.00)	-0.65 (-1.69 to 0.39)	-0.48 (-1.34 to 0.38)	**-0.32 (-0.56 to -0.08)**	-0.34 (-1.15 to 0.46)	-0.31 (-1.18 to 0.56)	-0.20 (-1.02 to 0.62)	-0.02 (-0.86 to 0.82)	**Sertindole**	NA
**-0.82 (-1.05 to -0.60)**	**-0.70 (-1.36 to -0.05)**	**-0.53 (-0.93 to -0.12)**	-0.37 (-1.14 to 0.40)	-0.39 (-1.48 to 0.70)	-0.36 (-0.73 to 0.01)	-0.24 (-1.35 to 0.86)	-0.07 (-0.29 to 0.15)	-0.05 (-0.86 to 0.76)	**Chlorpromazine**

Treatments are presented in order of efficacy ranking. Results of the network meta-analysis are reported in the left lower half and results of pairwise meta-analyses in the right upper half. Each cell provides the effect estimate and the corresponding 95% credible interval (95% CI) of a comparison (left lower half: treatment in column versus treatment in row; right upper half: treatment in row versus treatment in column). The type of effect size measure is standardized mean difference (SMD). Bold results indicate 95% CI excluding no effect. NA=not available.

12 Results of the subgroup analyses of the primary outcome

12.1 The criteria of treatment-resistant definitions

12.1.1 The criteria of treatment-resistant definitions

The criteria are a modified version of the previous network meta-analysis from Samara et al.[81] Additionally, we did a subgroup analysis using Samara's criteria and presented the results in Appendix 12.1.2.

a Low cut-off

Definition

Non-response or intolerant to antipsychotics without a specification and studies that do not meet intermediate or high cut-off criteria.

Number of studies: k = 19

Number of pairwise comparisons: m = 29

Number of treatments: n = 10

Number of designs: d = 13

Network plot

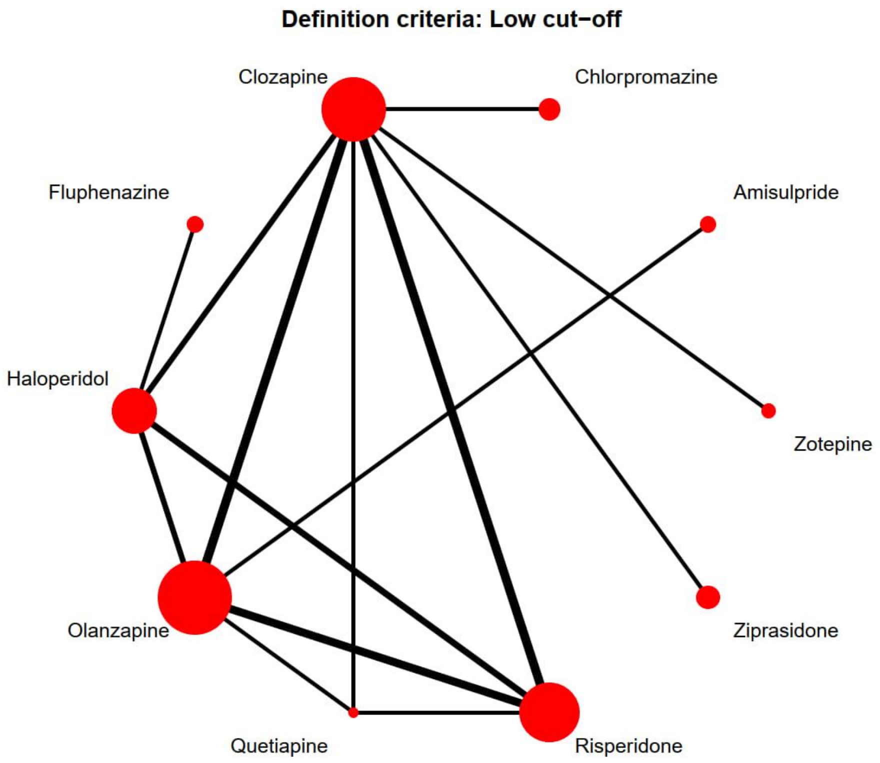

Lines link treatments with direct comparisons in trials; thickness of lines corresponds to the number of trials evaluating the comparison; size of the nodes corresponds to the number of participants assigned to the treatment.

τ	P-value	Inconsistency loop (%)
**0.1235**	**0.1370**	**11.11**

Forest-plot of results of network meta-analysis for antipsychotic drugs versus clozapine

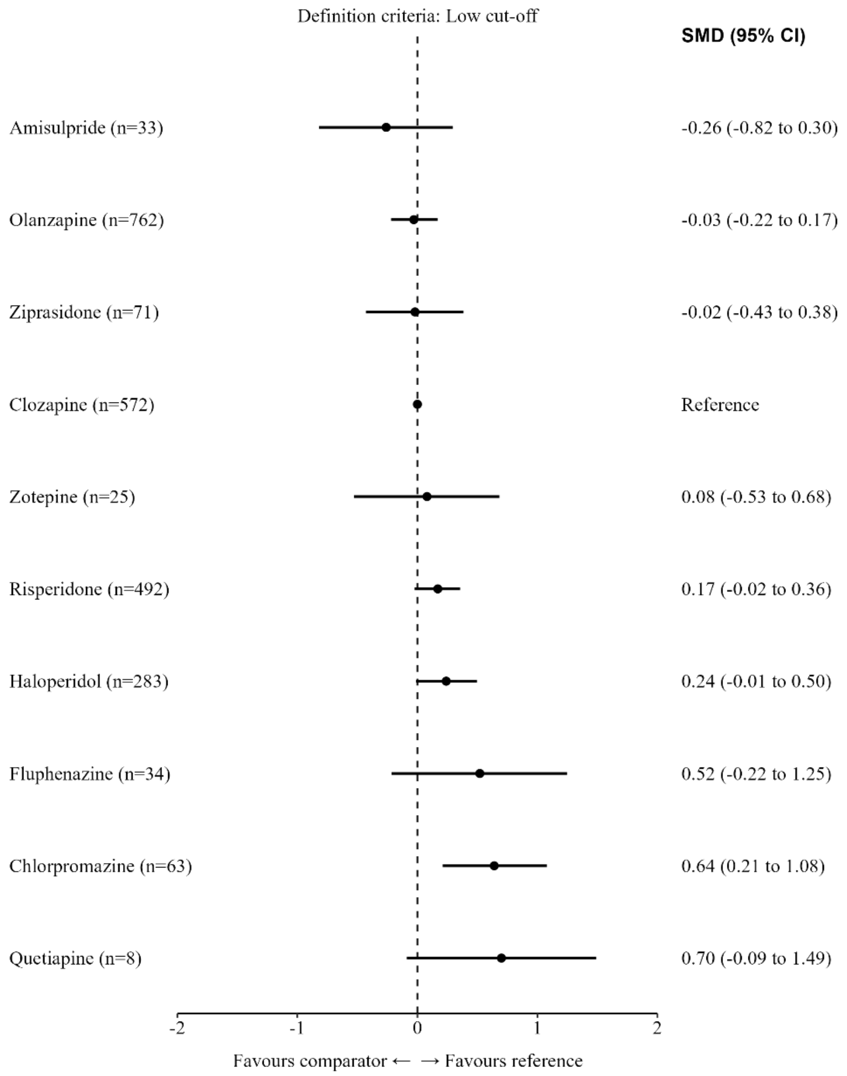

Effect sizes, measured as standardized mean difference (SMD), are from the network meta-analysis. Order of treatments is according to the mean effect size. Reference is clozapine. The direction of the effect is indicated below the x-axis.

League table

**Amisulpride**	-0.24 (-0.76 to 0.29)	NA	NA	NA	NA	NA	NA	NA	NA
-0.24 (-0.76 to 0.29)	**Olanzapine**	-0.03 (-0.27 to 0.20)	NA	NA	-0.23 (-0.48 to 0.01)	**-0.29 (-0.55 to -0.03)**	NA	-0.38 (-1.34 to 0.59)	NA
-0.26 (-0.82 to 0.30)	-0.03 (-0.22 to 0.17)	**Clozapine**	0.02 (-0.38 to 0.43)	-0.08 (-0.68 to 0.53)	-0.11 (-0.34 to 0.11)	-0.09 (-0.54 to 0.35)	NA	**-1.01 (-1.84 to -0.18)**	**-0.64 (-1.08 to -0.21)**
-0.24 (-0.93 to 0.45)	-0.00 (-0.45 to 0.45)	0.02 (-0.38 to 0.43)	**Ziprasidone**	NA	NA	NA	NA	NA	NA
-0.34 (-1.16 to 0.48)	-0.10 (-0.74 to 0.53)	-0.08 (-0.68 to 0.53)	-0.10 (-0.83 to 0.63)	**Zotepine**	NA	NA	NA	NA	NA
-0.43 (-0.99 to 0.13)	-0.19 (-0.38 to 0.00)	-0.17 (-0.36 to 0.02)	-0.19 (-0.64 to 0.26)	-0.09 (-0.72 to 0.55)	**Risperidone**	-0.07 (-0.40 to 0.26)	NA	0.06 (-1.03 to 1.14)	NA
-0.50 (-1.07 to 0.06)	**-0.27 (-0.49 to -0.05)**	-0.24 (-0.50 to 0.01)	-0.26 (-0.74 to 0.21)	-0.16 (-0.82 to 0.49)	-0.08 (-0.32 to 0.16)	**Haloperidol**	-0.27 (-0.96 to 0.41)	NA	NA
-0.78 (-1.67 to 0.11)	-0.54 (-1.26 to 0.18)	-0.52 (-1.25 to 0.22)	-0.54 (-1.37 to 0.30)	-0.44 (-1.39 to 0.51)	-0.35 (-1.08 to 0.38)	-0.27 (-0.96 to 0.41)	**Fluphenazine**	NA	NA
**-0.96 (-1.92 to -0.01)**	-0.73 (-1.52 to 0.07)	-0.70 (-1.49 to 0.09)	-0.72 (-1.61 to 0.16)	-0.62 (-1.62 to 0.37)	-0.53 (-1.33 to 0.26)	-0.46 (-1.28 to 0.36)	-0.19 (-1.25 to 0.88)	**Quetiapine**	NA
**-0.91 (-1.61 to -0.20)**	**-0.67 (-1.14 to -0.20)**	**-0.64 (-1.08 to -0.21)**	**-0.67 (-1.26 to -0.07)**	-0.57 (-1.31 to 0.18)	-0.48 (-0.95 to 0.00)	-0.40 (-0.90 to 0.10)	-0.13 (-0.98 to 0.72)	0.06 (-0.84 to 0.96)	**Chlorpromazine**

Treatments are presented in order of efficacy ranking. Results of the network meta-analysis are reported in the left lower half and results of pairwise meta-analyses in the right upper half. Each cell provides the effect estimate and the corresponding 95% credible interval (95% CI) of a comparison (left lower half: treatment in column versus treatment in row; right upper half: treatment in row versus treatment in column). The type of effect size measure is standardized mean difference (SMD). Bold results indicate 95% CI excluding no effect. NA=not available.

b Intermediate cut-off

Definition

Failure of response to at least 2 trials with antipsychotics at dosage in the therapeutic range and adequate duration and persistent at least moderate symptoms assessed with standardized rating scales.

Number of studies: k = 15

Number of pairwise comparisons: m = 15

Number of treatments: n = 7

Number of designs: d = 9

Network plot

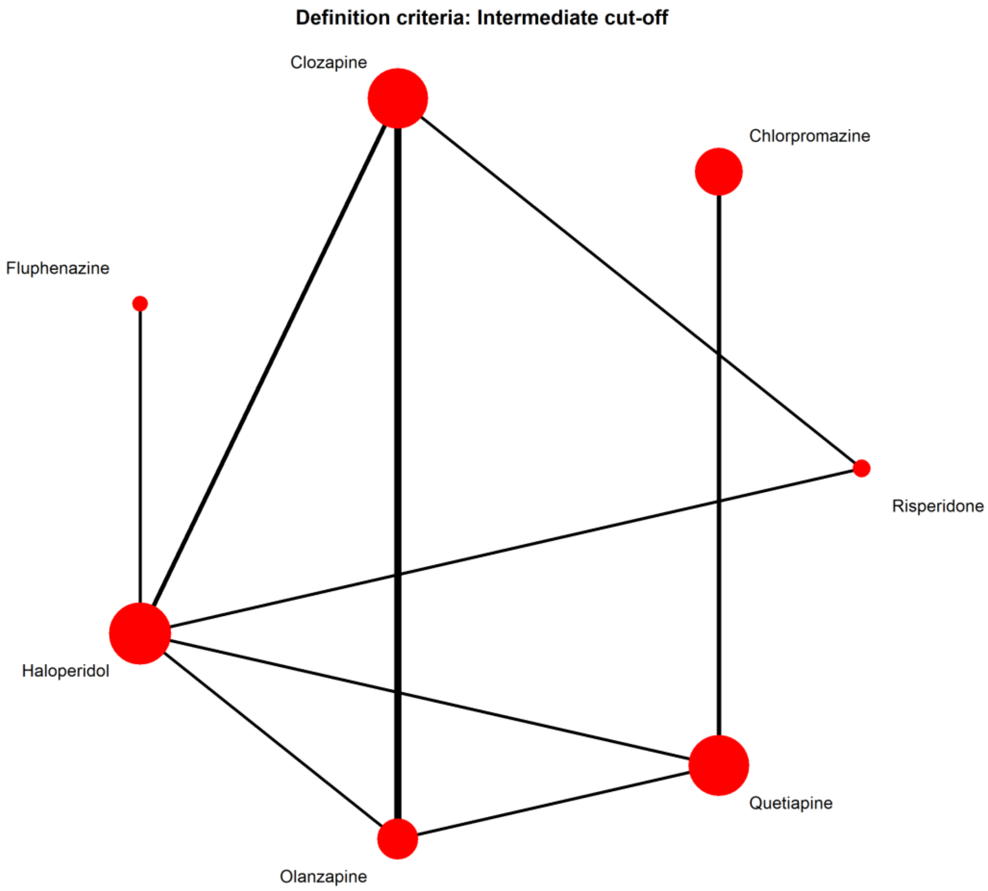

Lines link treatments with direct comparisons in trials; thickness of lines corresponds to the number of trials evaluating the comparison; size of the nodes corresponds to the number of participants assigned to the treatment.

τ	P-value	Inconsistency loop (%)
0	0.5108	0

<u>Forest-plot of results of network meta-analysis for antipsychotic drugs versus clozapine</u>

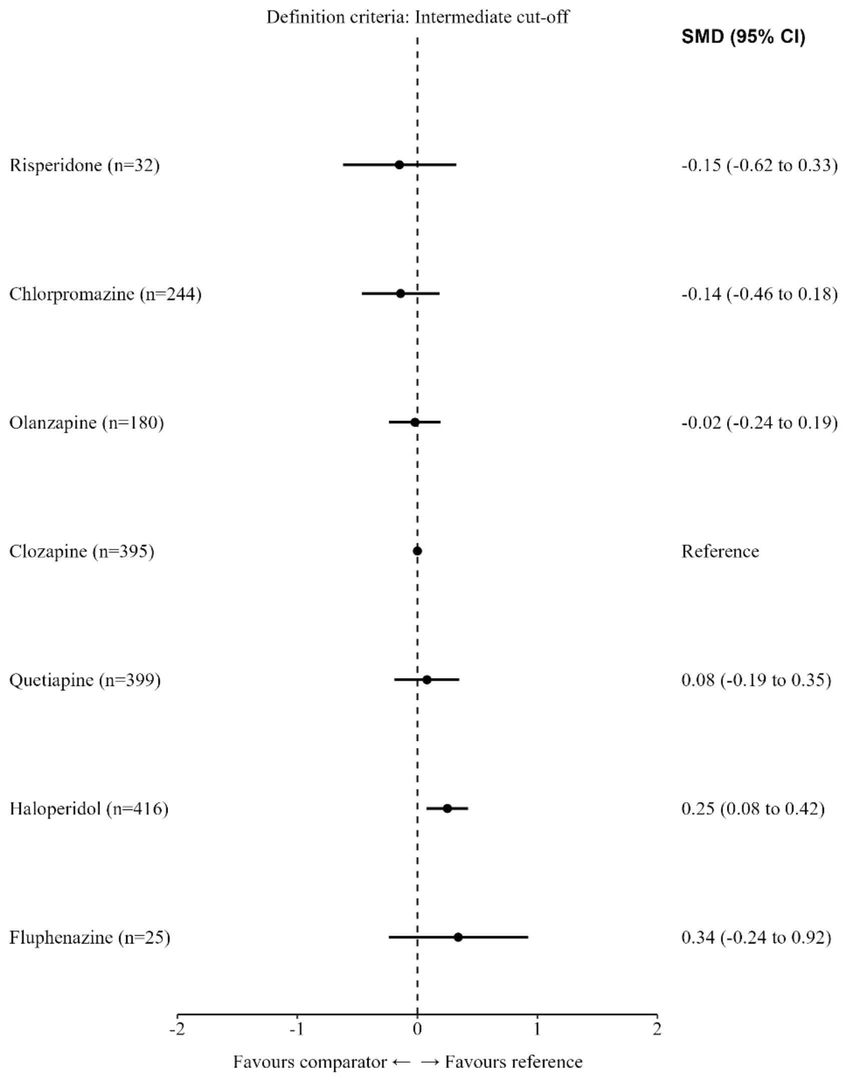

Effect sizes, measured as standardized mean difference (SMD), are from the network meta-analysis. Order of treatments is according to the mean effect size. Reference is clozapine. The direction of the effect is indicated below the x-axis.

League table

**Chlorpromazine**	NA	NA	NA	**-0.22 (-0.40 to -0.04)**	NA	NA
0.01 (-0.55 to 0.57)	**Risperidone**	NA	-0.03 (-0.58 to 0.52)	NA	NA	-0.72 (-1.62 to 0.19)
-0.12 (-0.48 to 0.24)	-0.13 (-0.64 to 0.39)	**Olanzapine**	-0.01 (-0.25 to 0.22)	0.09 (-0.53 to 0.72)	NA	-0.73 (-1.55 to 0.10)
-0.14 (-0.46 to 0.18)	-0.15 (-0.62 to 0.33)	-0.02 (-0.24 to 0.19)	**Clozapine**	NA	NA	**-0.23 (-0.41 to -0.04)**
**-0.22 (-0.40 to -0.04)**	-0.23 (-0.76 to 0.31)	-0.10 (-0.41 to 0.21)	-0.08 (-0.35 to 0.19)	**Quetiapine**	NA	-0.14 (-0.38 to 0.09)
-0.48 (-1.10 to 0.14)	-0.49 (-1.23 to 0.25)	-0.36 (-0.97 to 0.25)	-0.34 (-0.92 to 0.24)	-0.26 (-0.86 to 0.33)	**Fluphenazine**	0.09 (-0.46 to 0.65)
**-0.39 (-0.67 to -0.10)**	-0.40 (-0.88 to 0.09)	**-0.27 (-0.53 to -0.01)**	**-0.25 (-0.42 to -0.08)**	-0.17 (-0.39 to 0.05)	0.09 (-0.46 to 0.65)	**Haloperidol**

Treatments are presented in order of efficacy ranking. Results of the network meta-analysis are reported in the left lower half and results of pairwise meta-analyses in the right upper half. Each cell provides the effect estimate and the corresponding 95% credible interval (95% CI) of a comparison (left lower half: treatment in column versus treatment in row; right upper half: treatment in row versus treatment in column). The type of effect size measure is standardized mean difference (SMD). Bold results indicate 95% CI excluding no effect. NA=not available.

c High cut-off

Definition

Failure of response to at least two antipsychotic drug trials at dosage in the therapeutic range and adequate duration and at least one of them was prospective, also persistent at least moderate symptoms assessed with standardized rating scales at an assessment at the end of the prospective trial.

Number of studies: k = 11

Number of pairwise comparisons: m = 13

Number of treatments: n = 10

Number of designs: d = 9

Network plot

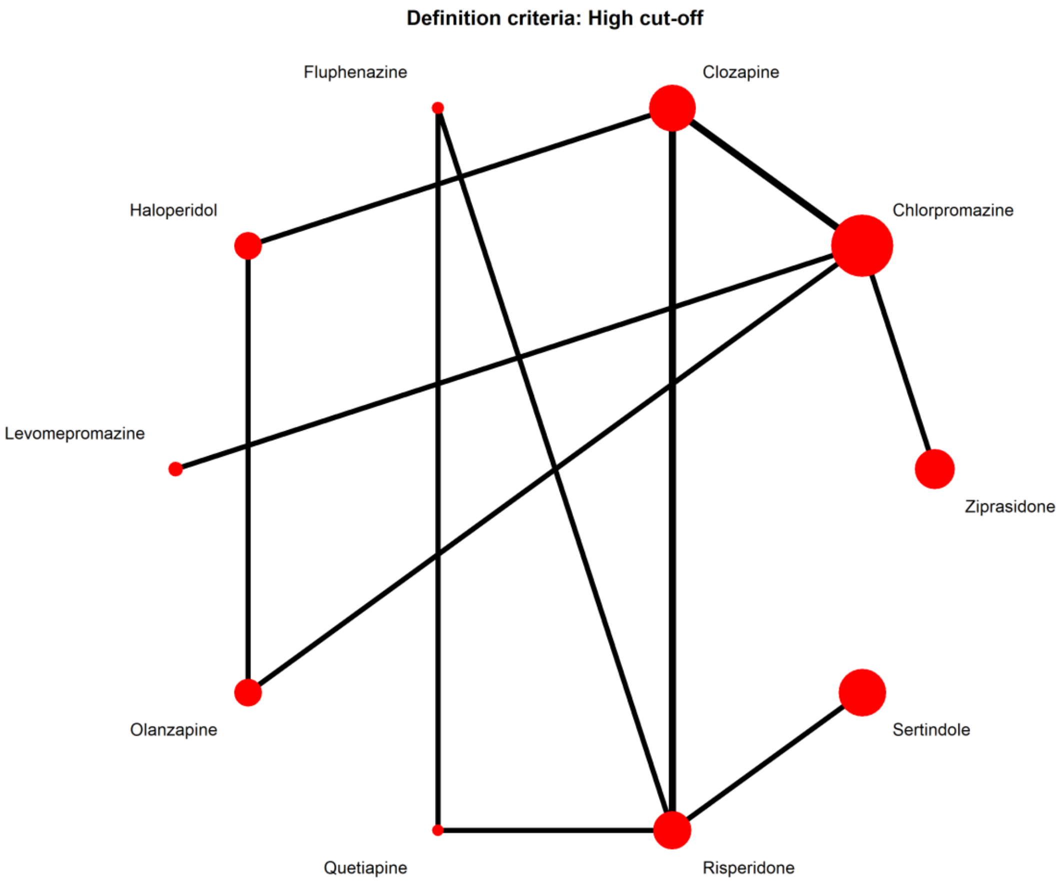

Lines link treatments with direct comparisons in trials; thickness of lines corresponds to the number of trials evaluating the comparison; size of the nodes corresponds to the number of participants assigned to the treatment.

τ	P-value	Inconsistency loop (%)
0.2199	0.4439	0

Forest-plot of results of network meta-analysis for antipsychotic drugs versus clozapine

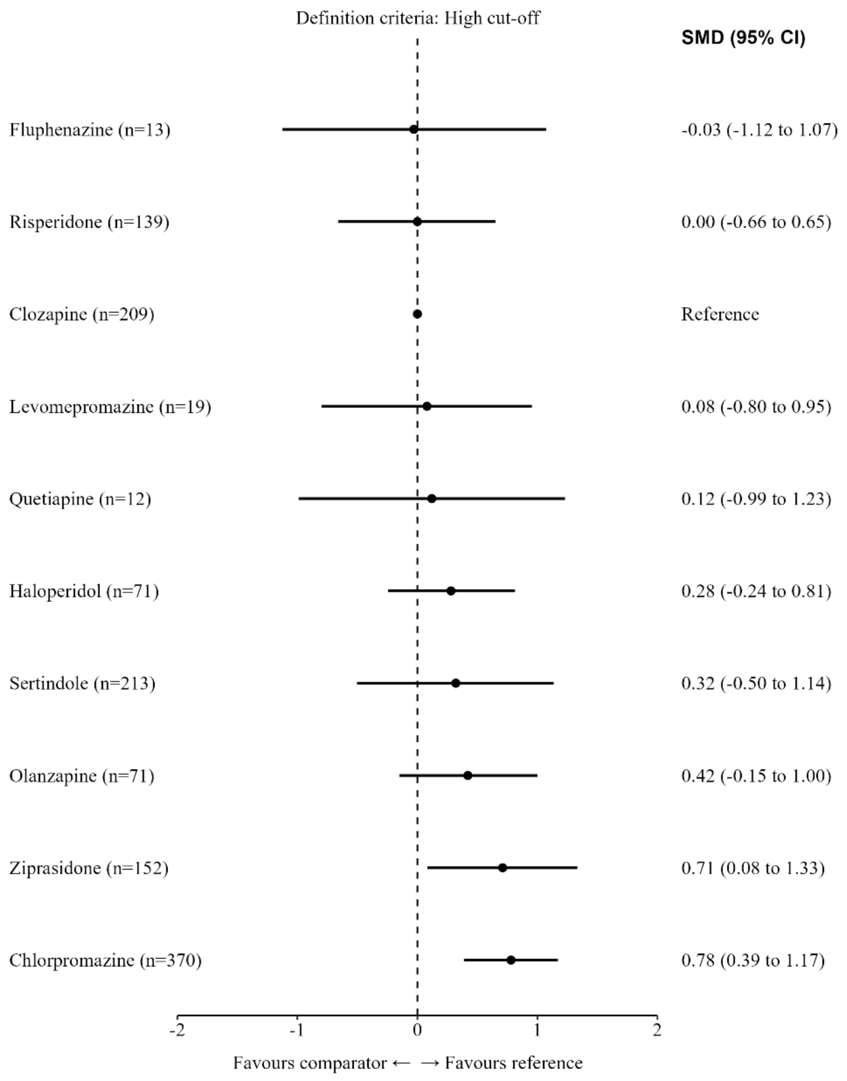

Effect sizes, measured as standardized mean difference (SMD), are from the network meta-analysis. Order of treatments is according to the mean effect size. Reference is clozapine. The direction of the effect is indicated below the x-axis.

League table

**Clozapine**	0.00 (-0.65 to 0.66)	NA	NA	NA	-0.14 (-0.77 to 0.49)	NA	NA	NA	**-0.84 (-1.26 to -0.42)**
0.00 (-0.65 to 0.66)	**Risperidone**	0.02 (-0.86 to 0.90)	NA	-0.12 (-1.02 to 0.77)	NA	-0.32 (-0.81 to 0.17)	NA	NA	NA
0.03 (-1.07 to 1.12)	0.02 (-0.86 to 0.90)	**Fluphenazine**	NA	-0.15 (-1.04 to 0.75)	NA	NA	NA	NA	NA
-0.08 (-0.95 to 0.80)	-0.08 (-1.18 to 1.01)	-0.10 (-1.51 to 1.30)	**Levomepromazine**	NA	NA	NA	NA	NA	-0.70 (-1.49 to 0.08)
-0.12 (-1.23 to 0.99)	-0.12 (-1.02 to 0.77)	-0.15 (-1.04 to 0.75)	-0.04 (-1.46 to 1.37)	**Quetiapine**	NA	NA	NA	NA	NA
-0.28 (-0.81 to 0.24)	-0.29 (-1.13 to 0.55)	-0.31 (-1.53 to 0.91)	-0.21 (-1.18 to 0.77)	-0.16 (-1.39 to 1.07)	**Haloperidol**	NA	0.02 (-0.64 to 0.68)	NA	NA
-0.32 (-1.14 to 0.50)	-0.32 (-0.81 to 0.17)	-0.34 (-1.35 to 0.67)	-0.24 (-1.44 to 0.96)	-0.20 (-1.22 to 0.83)	-0.03 (-1.01 to 0.94)	**Sertindole**	NA	NA	NA
-0.42 (-1.00 to 0.15)	-0.43 (-1.30 to 0.44)	-0.45 (-1.69 to 0.79)	-0.35 (-1.29 to 0.60)	-0.30 (-1.55 to 0.95)	-0.14 (-0.68 to 0.40)	-0.11 (-1.11 to 0.89)	**Olanzapine**	NA	-0.22 (-0.83 to 0.40)
**-0.71 (-1.33 to -0.08)**	-0.71 (-1.62 to 0.19)	-0.73 (-2.00 to 0.53)	-0.63 (-1.55 to 0.29)	-0.59 (-1.86 to 0.68)	-0.42 (-1.18 to 0.33)	-0.39 (-1.42 to 0.64)	-0.28 (-1.00 to 0.43)	**Ziprasidone**	-0.07 (-0.56 to 0.41)
**-0.78 (-1.17 to -0.39)**	**-0.78 (-1.55 to -0.02)**	-0.81 (-1.97 to 0.36)	-0.70 (-1.49 to 0.08)	-0.66 (-1.84 to 0.52)	-0.49 (-1.07 to 0.08)	-0.46 (-1.37 to 0.44)	-0.35 (-0.88 to 0.17)	-0.07 (-0.56 to 0.41)	**Chlorpromazine**

Treatments are presented in order of efficacy ranking. Results of the network meta-analysis are reported in the left lower half and results of pairwise meta-analyses in the right upper half. Each cell provides the effect estimate and the corresponding 95% credible interval (95% CI) of a comparison (left lower half: treatment in column versus treatment in row; right upper half: treatment in row versus treatment in column). The type of effect size measure is standardized mean difference (SMD). Bold results indicate 95% CI excluding no effect. NA=not available.

12.1.2 The criteria from Samara et al[81]

a No response to 1 previous antipsychotic

Number of studies: k = 11

Number of pairwise comparisons: m = 21

Number of treatments: n = 7

Number of designs: d = 9

Network plot

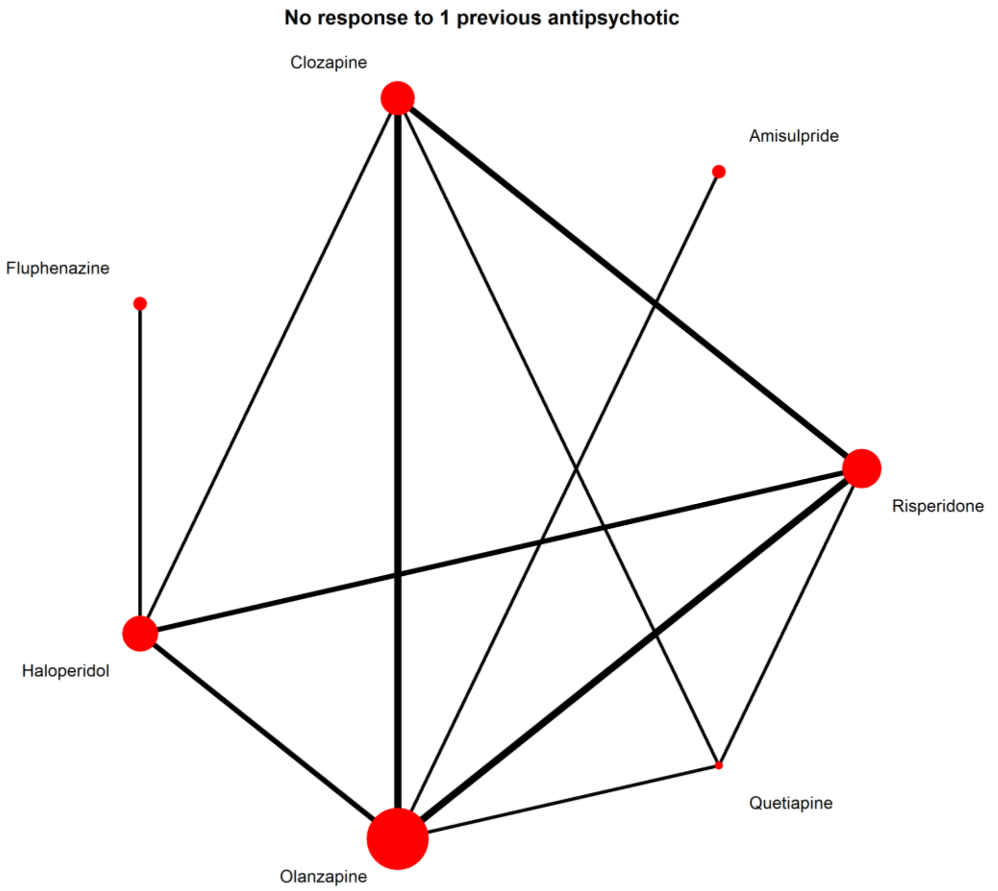

Lines link treatments with direct comparisons in trials; thickness of lines corresponds to the number of trials evaluating the comparison; size of the nodes corresponds to the number of participants assigned to the treatment.

τ	P-value	Inconsistency loop (%)
0.1111	0.1312	22.22

<u>Forest-plot of results of network meta-analysis for antipsychotic drugs versus clozapine</u>

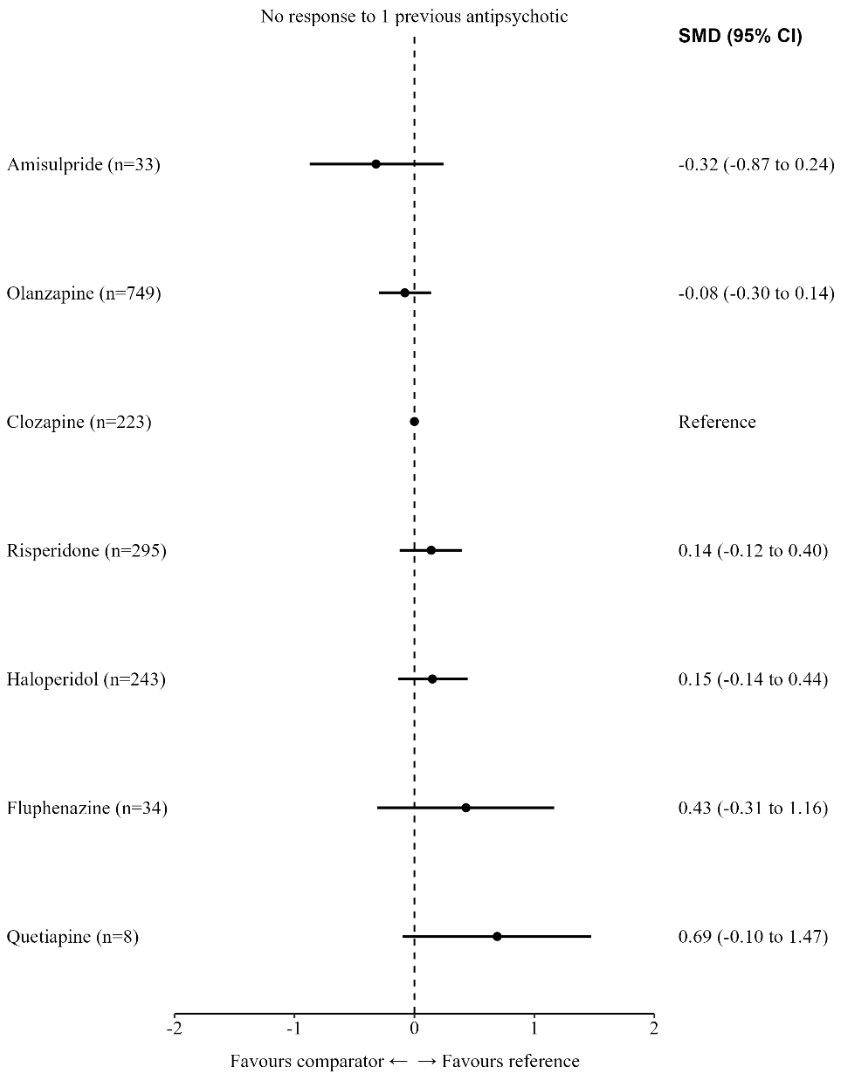

Effect sizes, measured as standardized mean difference (SMD), are from the network meta-analysis. Order of treatments is according to the mean effect size. Reference is clozapine. The direction of the effect is indicated below the x-axis.

League table

**Amisulpride**	-0.24 (-0.75 to 0.28)	NA	NA	NA	NA	NA
-0.24 (-0.75 to 0.28)	**Olanzapine**	-0.07 (-0.31 to 0.17)	-0.23 (-0.47 to 0.00)	**-0.29 (-0.54 to -0.04)**	NA	-0.38 (-1.34 to 0.58)
-0.32 (-0.87 to 0.24)	-0.08 (-0.30 to 0.14)	**Clozapine**	-0.04 (-0.39 to 0.31)	0.12 (-0.38 to 0.61)	NA	**-1.01 (-1.83 to -0.19)**
-0.45 (-1.01 to 0.10)	**-0.21 (-0.42 to -0.01)**	-0.14 (-0.40 to 0.12)	**Risperidone**	-0.03 (-0.42 to 0.36)	NA	0.06 (-1.02 to 1.14)
-0.47 (-1.03 to 0.09)	**-0.23 (-0.46 to -0.01)**	-0.15 (-0.44 to 0.14)	-0.02 (-0.29 to 0.25)	**Haloperidol**	-0.27 (-0.95 to 0.40)	NA
-0.74 (-1.62 to 0.14)	-0.51 (-1.22 to 0.21)	-0.43 (-1.16 to 0.31)	-0.29 (-1.02 to 0.44)	-0.27 (-0.95 to 0.40)	**Fluphenazine**	NA
**-1.00 (-1.95 to -0.06)**	-0.77 (-1.56 to 0.03)	-0.69 (-1.47 to 0.10)	-0.55 (-1.36 to 0.25)	-0.53 (-1.35 to 0.29)	-0.26 (-1.32 to 0.80)	**Quetiapine**

Treatments are presented in order of efficacy ranking. Results of the network meta-analysis are reported in the left lower half and results of pairwise meta-analyses in the right upper half. Each cell provides the effect estimate and the corresponding 95% credible interval (95% CI) of a comparison (left lower half: treatment in column versus treatment in row; right upper half: treatment in row versus treatment in column). The type of effect size measure is standardized mean difference (SMD). Bold results indicate 95% CI excluding no effect. NA=not available.

b No response to at least 2 retrospective periods of antipsychotic treatment

Number of studies: k = 20

Number of pairwise comparisons: m = 20

Number of treatments: n = 9

Number of designs: d = 11

Network plot

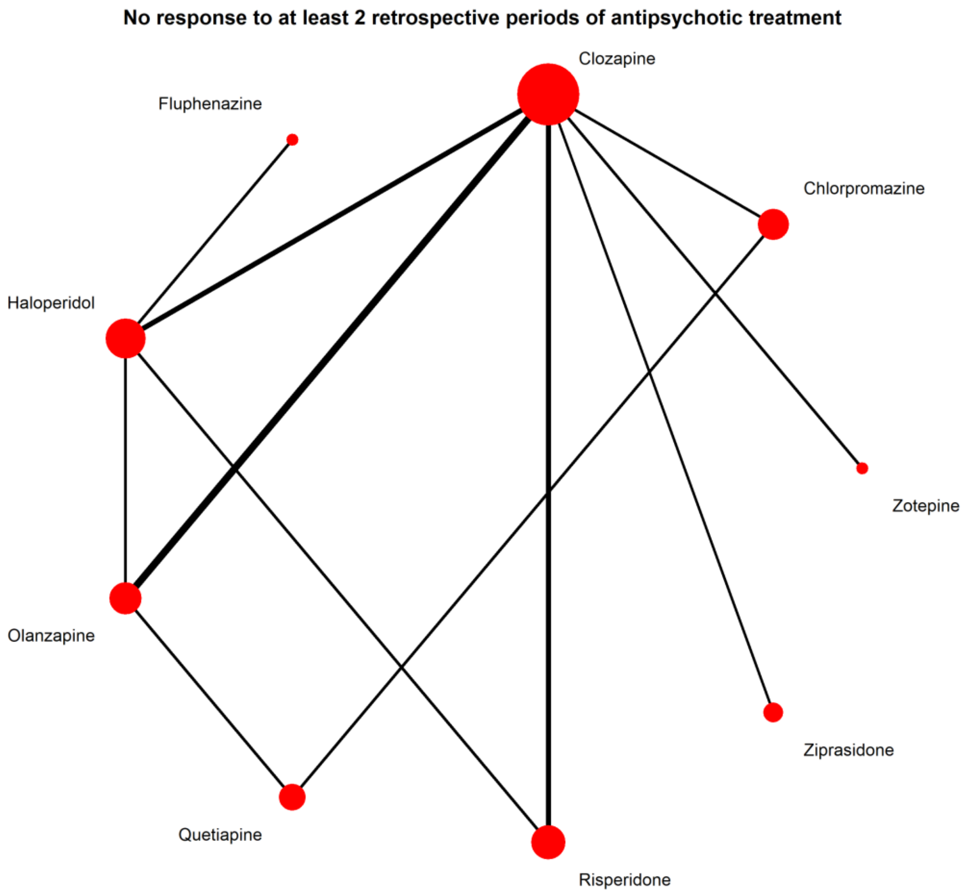

Lines link treatments with direct comparisons in trials; thickness of lines corresponds to the number of trials evaluating the comparison; size of the nodes corresponds to the number of participants assigned to the treatment.

τ	P-value	Inconsistency loop (%)
0.1506	0.2017	0.375

Forest-plot of results of network meta-analysis for antipsychotic drugs versus clozapine

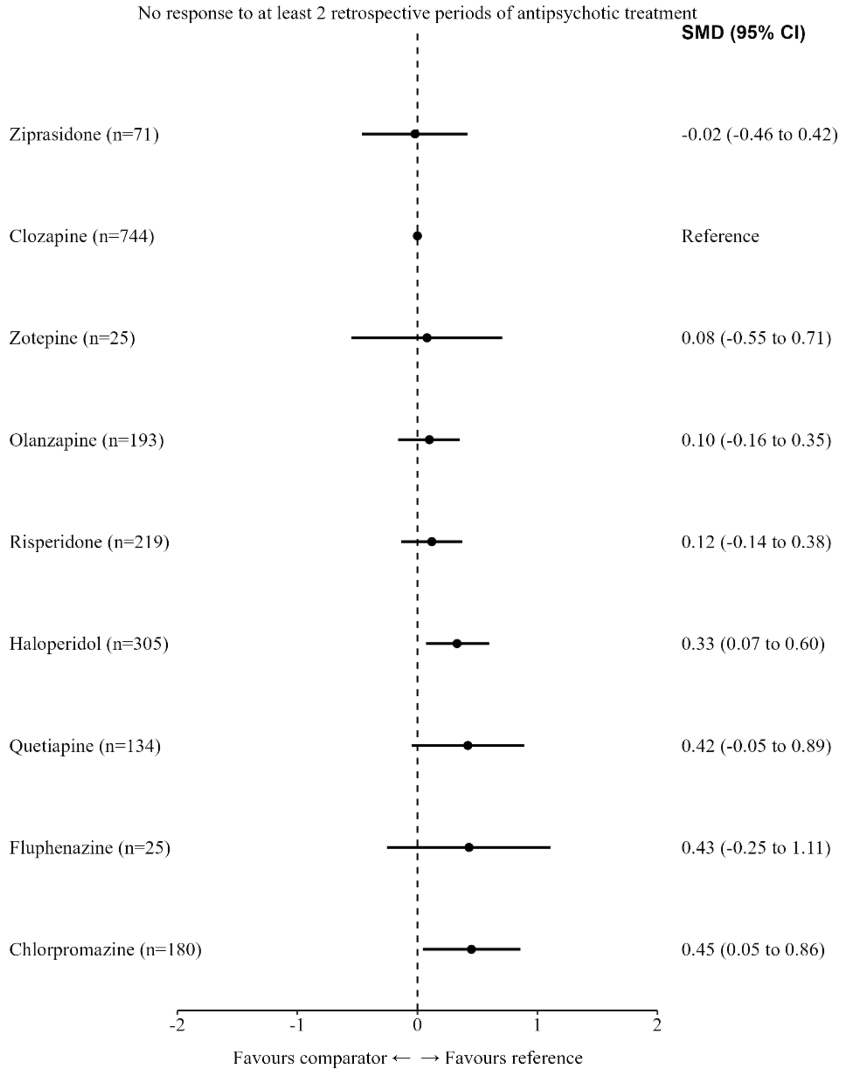

Effect sizes, measured as standardized mean difference (SMD), are from the network meta-analysis. Order of treatments is according to the mean effect size. Reference is clozapine. The direction of the effect is indicated below the x-axis.

League table

**Clozapine**	0.02 (-0.42 to 0.46)	-0.08 (-0.35 to 0.20)	-0.08 (-0.71 to 0.55)	-0.11 (-0.39 to 0.17)	-0.29 (-0.59 to 0.01)	NA	NA	**-0.64 (-1.11 to -0.18)**
0.02 (-0.42 to 0.46)	**Ziprasidone**	NA	NA	NA	NA	NA	NA	NA
-0.10 (-0.35 to 0.16)	-0.12 (-0.63 to 0.39)	**Olanzapine**	NA	NA	-0.73 (-1.61 to 0.15)	NA	0.09 (-0.59 to 0.78)	NA
-0.08 (-0.71 to 0.55)	-0.10 (-0.87 to 0.67)	0.02 (-0.66 to 0.70)	**Zotepine**	NA	NA	NA	NA	NA
-0.12 (-0.38 to 0.14)	-0.14 (-0.65 to 0.37)	-0.02 (-0.38 to 0.33)	-0.04 (-0.72 to 0.64)	**Risperidone**	-0.16 (-0.76 to 0.43)	NA	NA	NA
**-0.33 (-0.60 to -0.07)**	-0.36 (-0.87 to 0.16)	-0.24 (-0.59 to 0.11)	-0.26 (-0.94 to 0.42)	-0.21 (-0.55 to 0.12)	**Haloperidol**	-0.09 (-0.72 to 0.54)	NA	NA
-0.43 (-1.11 to 0.25)	-0.45 (-1.26 to 0.36)	-0.33 (-1.05 to 0.39)	-0.35 (-1.28 to 0.58)	-0.31 (-1.02 to 0.40)	-0.09 (-0.72 to 0.54)	**Fluphenazine**	NA	NA
-0.42 (-0.89 to 0.05)	-0.44 (-1.09 to 0.20)	-0.33 (-0.80 to 0.15)	-0.34 (-1.13 to 0.44)	-0.30 (-0.83 to 0.23)	-0.09 (-0.62 to 0.45)	0.01 (-0.82 to 0.83)	**Quetiapine**	0.11 (-0.29 to 0.50)
**-0.45 (-0.86 to -0.05)**	-0.47 (-1.07 to 0.12)	-0.36 (-0.80 to 0.09)	-0.37 (-1.12 to 0.37)	-0.33 (-0.81 to 0.15)	-0.12 (-0.60 to 0.36)	-0.02 (-0.82 to 0.77)	-0.03 (-0.39 to 0.33)	**Chlorpromazine**

Treatments are presented in order of efficacy ranking. Results of the network meta-analysis are reported in the left lower half and results of pairwise meta-analyses in the right upper half. Each cell provides the effect estimate and the corresponding 95% credible interval (95% CI) of a comparison (left lower half: treatment in column versus treatment in row; right upper half: treatment in row versus treatment in column). The type of effect size measure is standardized mean difference (SMD). Bold results indicate 95% CI excluding no effect. NA=not available.

c No response to a combination of retrospective and prospective criteria for treatment resistance

Number of studies: k = 14

Number of pairwise comparisons: m = 16

Number of treatments: n = 10

Number of designs: d = 12

Network plot

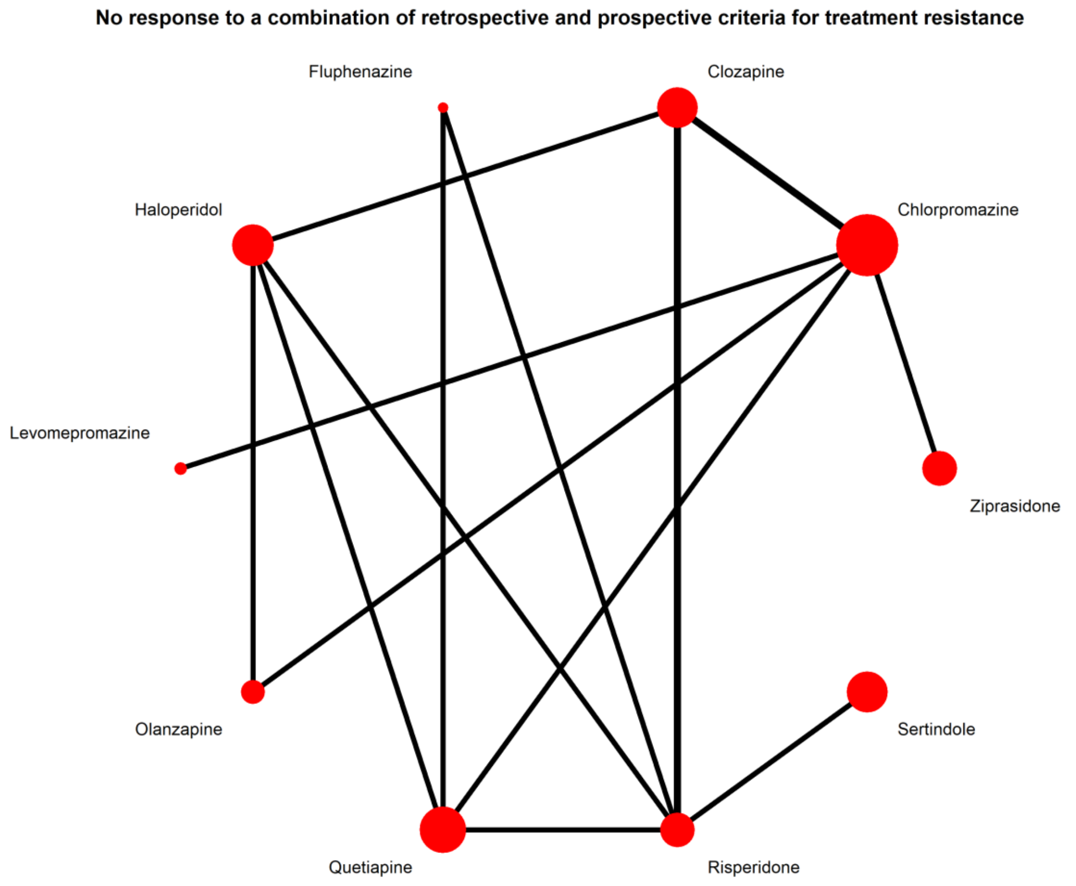

Lines link treatments with direct comparisons in trials; thickness of lines corresponds to the number of trials evaluating the comparison; size of the nodes corresponds to the number of participants assigned to the treatment.

τ	P-value	Inconsistency loop (%)
0.38	0.2853	0

Forest-plot of results of network meta-analysis for antipsychotic drugs versus clozapine

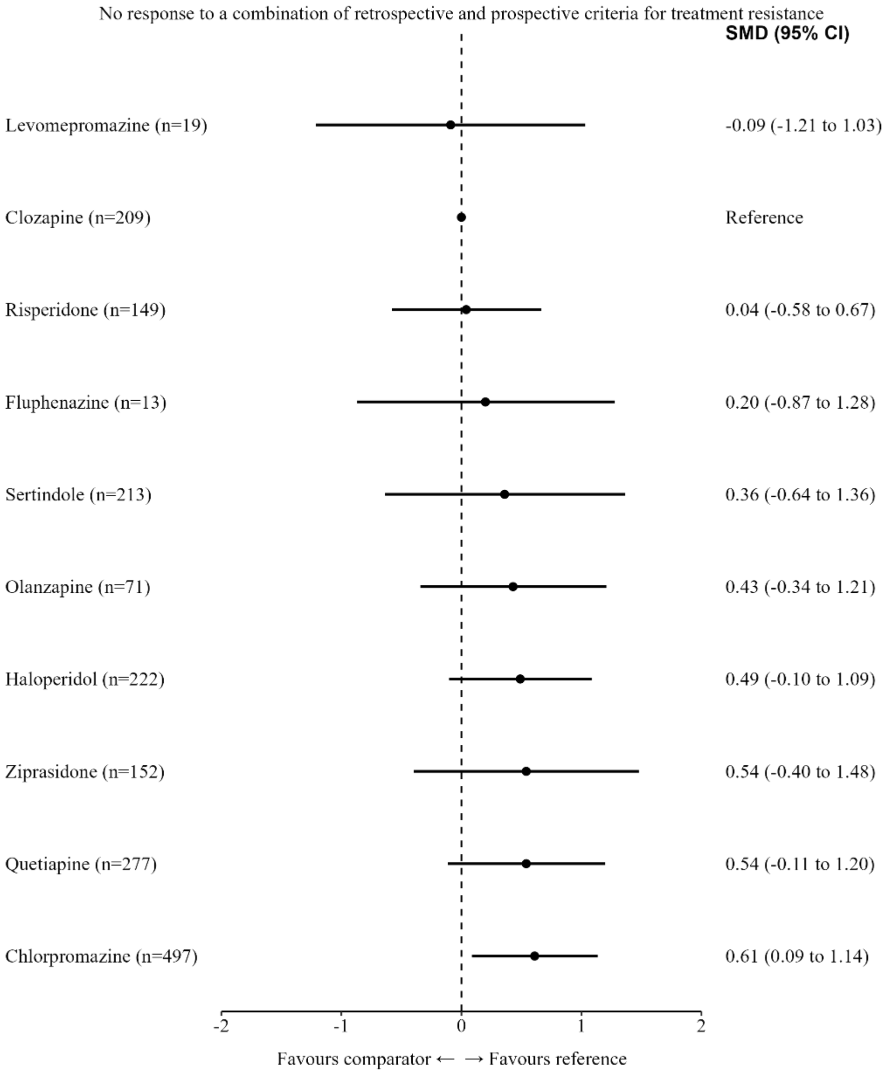

Effect sizes, measured as standardized mean difference (SMD), are from the network meta-analysis. Order of treatments is according to the mean effect size. Reference is clozapine. The direction of the effect is indicated below the x-axis.

League table

**Clozapine**	NA	0.03 (-0.75 to 0.82)	NA	NA	NA	-0.14 (-1.01 to 0.73)	NA	NA	**-0.83 (-1.45 to -0.22)**
0.09 (-1.03 to 1.21)	**Levomepromazine**	NA	NA	NA	NA	NA	NA	NA	-0.70 (-1.69 to 0.29)
-0.04 (-0.67 to 0.58)	-0.13 (-1.36 to 1.09)	**Risperidone**	0.02 (-1.05 to 1.09)	-0.32 (-1.10 to 0.46)	NA	-0.72 (-1.89 to 0.45)	NA	-0.12 (-1.21 to 0.96)	NA
-0.20 (-1.28 to 0.87)	-0.29 (-1.77 to 1.18)	-0.16 (-1.15 to 0.83)	**Fluphenazine**	NA	NA	NA	NA	-0.15 (-1.23 to 0.94)	NA
-0.36 (-1.36 to 0.64)	-0.45 (-1.91 to 1.00)	-0.32 (-1.10 to 0.46)	-0.16 (-1.42 to 1.10)	**Sertindole**	NA	NA	NA	NA	NA
-0.43 (-1.21 to 0.34)	-0.52 (-1.73 to 0.69)	-0.39 (-1.27 to 0.49)	-0.23 (-1.44 to 0.98)	-0.07 (-1.25 to 1.11)	**Olanzapine**	-0.02 (-0.91 to 0.88)	NA	NA	-0.22 (-1.08 to 0.65)
-0.49 (-1.09 to 0.10)	-0.58 (-1.76 to 0.59)	-0.45 (-1.13 to 0.23)	-0.29 (-1.36 to 0.79)	-0.13 (-1.17 to 0.91)	-0.06 (-0.76 to 0.64)	**Haloperidol**	NA	0.14 (-0.64 to 0.92)	NA
-0.54 (-1.48 to 0.40)	-0.63 (-1.89 to 0.63)	-0.50 (-1.56 to 0.56)	-0.34 (-1.67 to 1.00)	-0.18 (-1.49 to 1.14)	-0.11 (-1.15 to 0.93)	-0.05 (-1.05 to 0.95)	**Ziprasidone**	NA	-0.07 (-0.85 to 0.71)
-0.54 (-1.20 to 0.11)	-0.63 (-1.79 to 0.53)	-0.50 (-1.20 to 0.20)	-0.34 (-1.33 to 0.66)	-0.18 (-1.23 to 0.87)	-0.11 (-0.91 to 0.69)	-0.05 (-0.64 to 0.54)	-0.00 (-0.99 to 0.98)	**Quetiapine**	0.32 (-0.46 to 1.11)
**-0.61 (-1.14 to -0.09)**	-0.70 (-1.69 to 0.29)	-0.57 (-1.29 to 0.15)	-0.41 (-1.50 to 0.68)	-0.25 (-1.31 to 0.81)	-0.18 (-0.87 to 0.51)	-0.12 (-0.75 to 0.51)	-0.07 (-0.85 to 0.71)	-0.07 (-0.67 to 0.53)	**Chlorpromazine**

Treatments are presented in order of efficacy ranking. Results of the network meta-analysis are reported in the left lower half and results of pairwise meta-analyses in the right upper half. Each cell provides the effect estimate and the corresponding 95% credible interval (95% CI) of a comparison (left lower half: treatment in column versus treatment in row; right upper half: treatment in row versus treatment in column). The type of effect size measure is standardized mean difference (SMD). Bold results indicate 95% CI excluding no effect. NA=not available

12.2 Mean age

a More than 18 years old

Number of studies: k = 41

Number of pairwise comparisons: m = 53

Number of treatments: n = 12

Number of designs: d = 21

Network plot

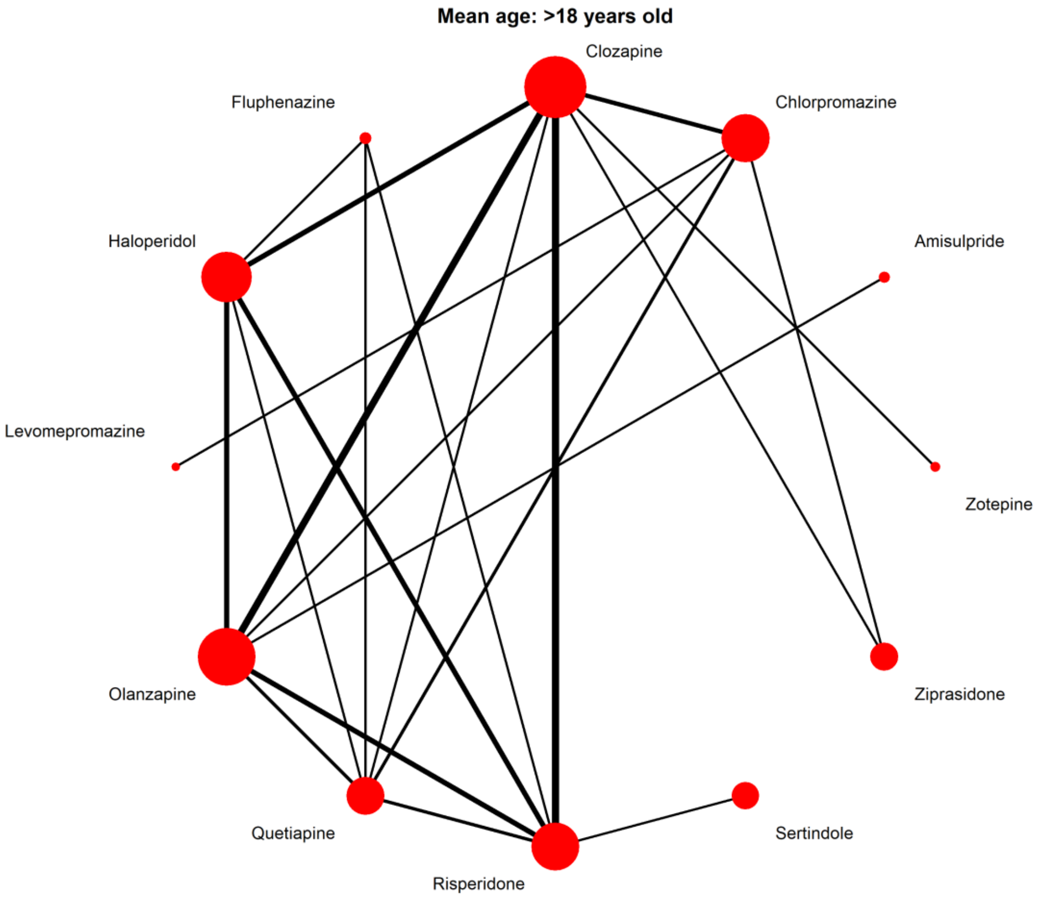

Lines link treatments with direct comparisons in trials; thickness of lines corresponds to the number of trials evaluating the comparison; size of the nodes corresponds to the number of participants assigned to the treatment.

τ	P-value	Inconsistency loop (%)
0.1882	< 0.0001	11.11

Forest-plot of results of network meta-analysis for antipsychotic drugs versus clozapine

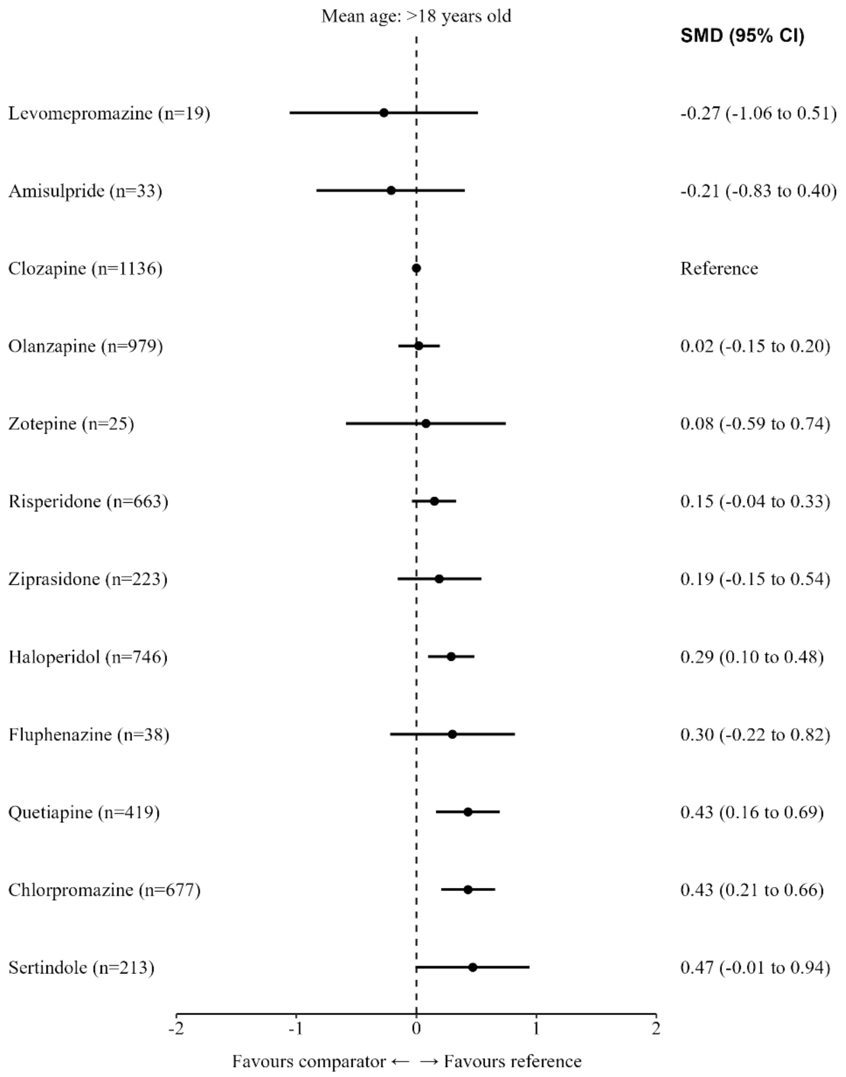

Effect sizes, measured as standardized mean difference (SMD), are from the network meta-analysis. Order of treatments is according to the mean effect size. Reference is clozapine. The direction of the effect is indicated below the x-axis.

League table

**Amisulpride**	NA	NA	-0.24 (-0.83 to 0.36)	NA	NA	NA	NA	NA	NA	NA	NA
0.06 (-0.93 to 1.05)	**Levomepromazine**	NA	NA	NA	NA	NA	NA	NA	NA	NA	-0.70 (-1.45 to 0.05)
-0.21 (-0.83 to 0.40)	-0.27 (-1.06 to 0.51)	**Clozapine**	0.03 (-0.19 to 0.25)	-0.08 (-0.74 to 0.59)	-0.08 (-0.30 to 0.15)	0.02 (-0.47 to 0.52)	NA	-0.13 (-0.41 to 0.14)	NA	**-1.01 (-1.89 to -0.13)**	**-0.78 (-1.08 to -0.47)**
-0.24 (-0.83 to 0.36)	-0.29 (-1.09 to 0.50)	-0.02 (-0.20 to 0.15)	**Olanzapine**	NA	-0.23 (-0.53 to 0.07)	NA	NA	-0.28 (-0.56 to 0.00)	NA	-0.07 (-0.65 to 0.52)	-0.22 (-0.79 to 0.36)
-0.29 (-1.20 to 0.62)	-0.35 (-1.38 to 0.68)	-0.08 (-0.74 to 0.59)	-0.06 (-0.74 to 0.63)	**Zotepine**	NA	NA	NA	NA	NA	NA	NA
-0.36 (-0.99 to 0.27)	-0.42 (-1.22 to 0.38)	-0.15 (-0.33 to 0.04)	-0.12 (-0.33 to 0.08)	-0.07 (-0.76 to 0.62)	**Risperidone**	NA	0.02 (-0.83 to 0.87)	-0.15 (-0.49 to 0.19)	-0.32 (-0.76 to 0.12)	-0.06 (-0.74 to 0.63)	NA
-0.41 (-1.11 to 0.30)	-0.47 (-1.29 to 0.36)	-0.19 (-0.54 to 0.15)	-0.17 (-0.55 to 0.21)	-0.12 (-0.87 to 0.64)	-0.05 (-0.43 to 0.34)	**Ziprasidone**	NA	NA	NA	NA	-0.07 (-0.50 to 0.36)
-0.52 (-1.31 to 0.28)	-0.57 (-1.50 to 0.36)	-0.30 (-0.82 to 0.22)	-0.28 (-0.80 to 0.25)	-0.22 (-1.07 to 0.62)	-0.15 (-0.68 to 0.37)	-0.11 (-0.72 to 0.51)	**Fluphenazine**	0.09 (-0.57 to 0.76)	NA	-0.15 (-1.01 to 0.72)	NA
-0.50 (-1.13 to 0.12)	-0.56 (-1.36 to 0.24)	**-0.29 (-0.48 to -0.10)**	**-0.27 (-0.47 to -0.07)**	-0.21 (-0.90 to 0.48)	-0.14 (-0.36 to 0.07)	-0.10 (-0.48 to 0.29)	0.01 (-0.49 to 0.52)	**Haloperidol**	NA	0.14 (-0.29 to 0.58)	NA
-0.68 (-1.45 to 0.08)	-0.74 (-1.65 to 0.17)	-0.47 (-0.94 to 0.01)	-0.44 (-0.93 to 0.04)	-0.39 (-1.21 to 0.43)	-0.32 (-0.76 to 0.12)	-0.27 (-0.86 to 0.31)	-0.17 (-0.85 to 0.51)	-0.18 (-0.67 to 0.31)	**Sertindole**	NA	NA
-0.64 (-1.30 to 0.01)	-0.70 (-1.49 to 0.09)	**-0.43 (-0.69 to -0.16)**	**-0.41 (-0.68 to -0.13)**	-0.35 (-1.07 to 0.37)	-0.28 (-0.57 to 0.01)	-0.23 (-0.63 to 0.16)	-0.13 (-0.66 to 0.41)	-0.14 (-0.41 to 0.13)	0.04 (-0.49 to 0.57)	**Quetiapine**	0.22 (-0.10 to 0.53)
-0.64 (-1.29 to 0.00)	-0.70 (-1.45 to 0.05)	**-0.43 (-0.66 to -0.21)**	**-0.41 (-0.66 to -0.15)**	-0.35 (-1.06 to 0.35)	**-0.28 (-0.56 to -0.01)**	-0.24 (-0.58 to 0.10)	-0.13 (-0.67 to 0.42)	-0.14 (-0.41 to 0.13)	0.04 (-0.48 to 0.55)	-0.00 (-0.25 to 0.25)	**Chlorpromazine**

Treatments are presented in order of efficacy ranking. Results of the network meta-analysis are reported in the left lower half and results of pairwise meta-analyses in the right upper half. Each cell provides the effect estimate and the corresponding 95% credible interval (95% CI) of a comparison (left lower half: treatment in column versus treatment in row; right upper half: treatment in row versus treatment in column). The type of effect size measure is standardized mean difference (SMD). Bold results indicate 95% CI excluding no effect. NA=not available.

b Less than 18 years old

No closed loops

12.3 Dose of the antipsychotics in chlorpromazine-equivalents

For the purpose of this analysis the median number of dose in chlorpromazine-equivalents was used.

a High dose

Number of studies: k = 26

Number of pairwise comparisons: m = 31

Number of treatments: n = 10

Number of designs: d = 14

Network plot

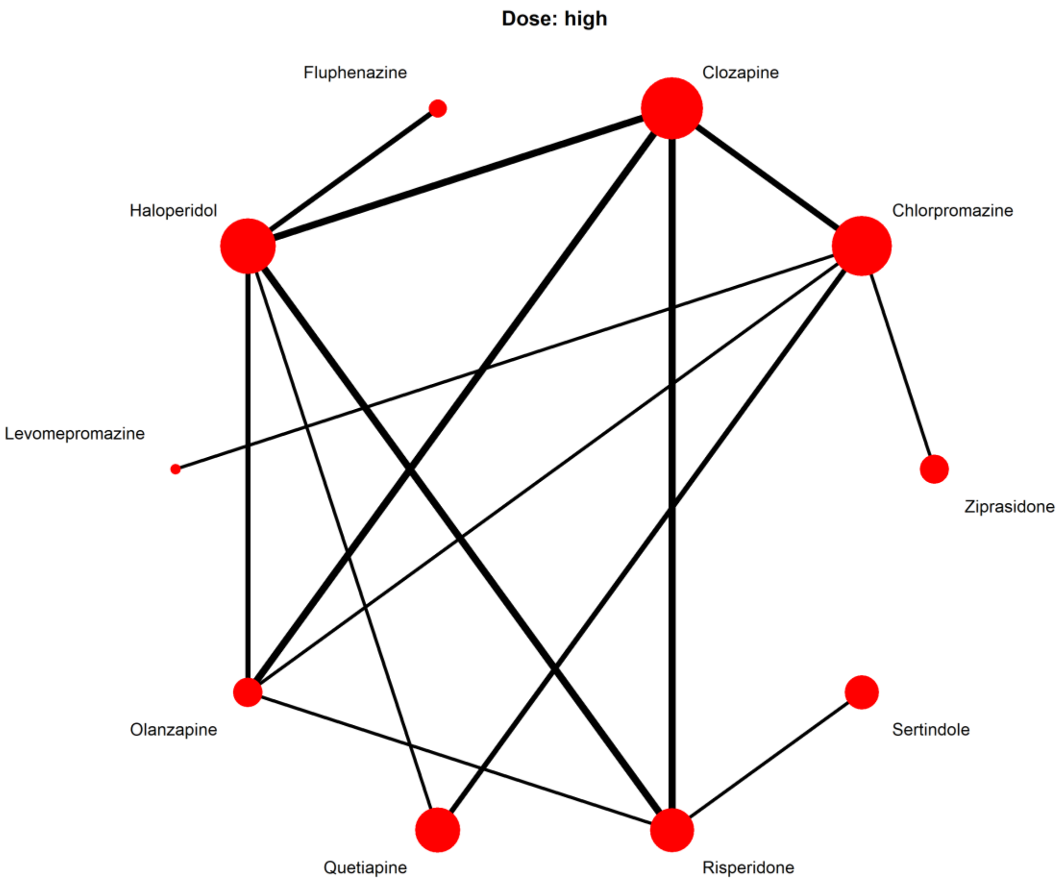

Lines link treatments with direct comparisons in trials; thickness of lines corresponds to the number of trials evaluating the comparison; size of the nodes corresponds to the number of participants assigned to the treatment.

τ	P-value	Inconsistency loop (%)
0.2378	< 0.0001	30.0

Forest-plot of results of network meta-analysis for antipsychotic drugs versus clozapine

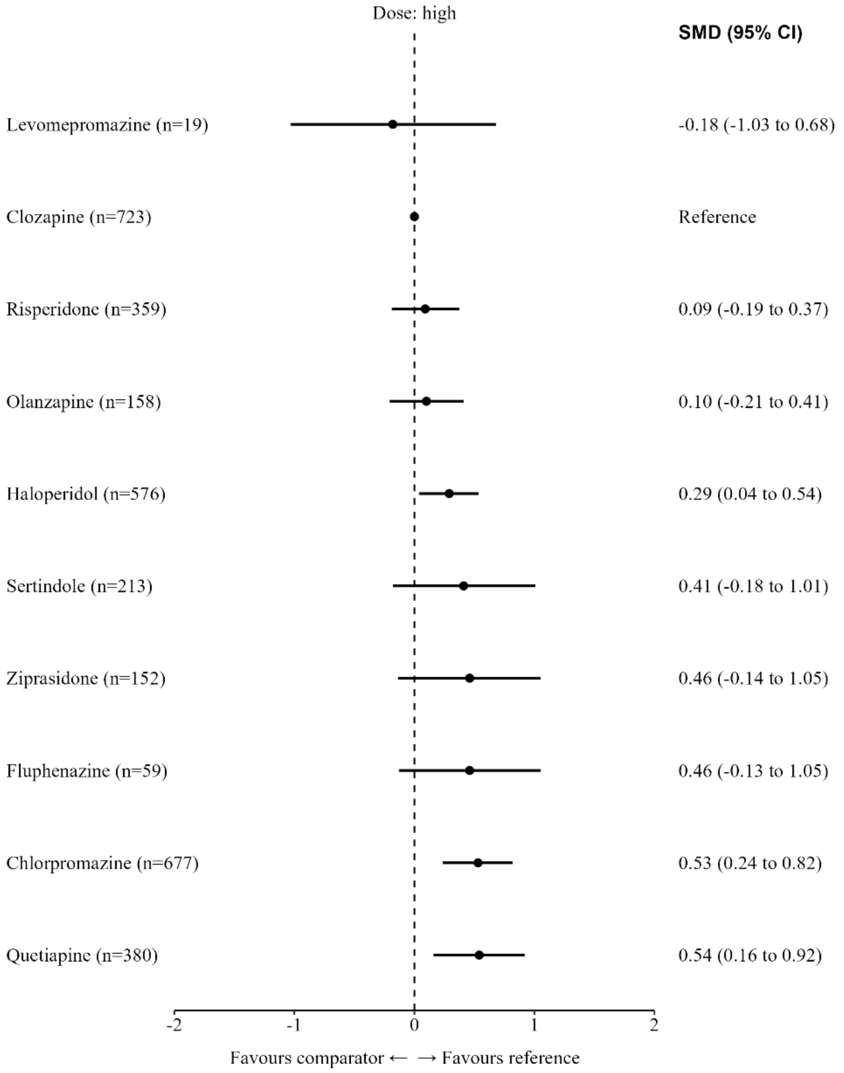

Effect sizes, measured as standardized mean difference (SMD), are from the network meta-analysis. Order of treatments is according to the mean effect size. Reference is clozapine. The direction of the effect is indicated below the x-axis.

League table

**Clozapine**	NA	0.01 (-0.33 to 0.34)	0.07 (-0.33 to 0.46)	-0.13 (-0.45 to 0.19)	NA	NA	NA	**-0.77 (-1.12 to -0.42)**	NA
0.18 (-0.68 to 1.03)	**Levomepromazine**	NA	NA	NA	NA	NA	NA	-0.70 (-1.51 to 0.10)	NA
-0.09 (-0.37 to 0.19)	-0.27 (-1.16 to 0.62)	**Risperidone**	0.24 (-0.40 to 0.88)	-0.15 (-0.53 to 0.22)	-0.32 (-0.84 to 0.20)	NA	NA	NA	NA
-0.10 (-0.41 to 0.21)	-0.28 (-1.16 to 0.61)	-0.01 (-0.38 to 0.37)	**Olanzapine**	-0.20 (-0.67 to 0.27)	NA	NA	NA	-0.22 (-0.85 to 0.42)	NA
**-0.29 (-0.54 to -0.04)**	-0.46 (-1.33 to 0.41)	-0.19 (-0.49 to 0.10)	-0.19 (-0.52 to 0.15)	**Haloperidol**	NA	NA	-0.18 (-0.71 to 0.36)	NA	0.14 (-0.38 to 0.66)
-0.41 (-1.01 to 0.18)	-0.59 (-1.62 to 0.44)	-0.32 (-0.84 to 0.20)	-0.31 (-0.95 to 0.33)	-0.13 (-0.73 to 0.47)	**Sertindole**	NA	NA	NA	NA
-0.46 (-1.05 to 0.14)	-0.63 (-1.59 to 0.32)	-0.36 (-1.01 to 0.28)	-0.35 (-0.99 to 0.28)	-0.17 (-0.79 to 0.45)	-0.04 (-0.87 to 0.79)	**Ziprasidone**	NA	-0.07 (-0.59 to 0.45)	NA
-0.46 (-1.05 to 0.13)	-0.64 (-1.66 to 0.39)	-0.37 (-0.98 to 0.24)	-0.36 (-0.99 to 0.27)	-0.18 (-0.71 to 0.36)	-0.05 (-0.85 to 0.76)	-0.01 (-0.82 to 0.81)	**Fluphenazine**	NA	NA
**-0.53 (-0.82 to -0.24)**	-0.70 (-1.51 to 0.10)	**-0.43 (-0.82 to -0.05)**	**-0.43 (-0.79 to -0.06)**	-0.24 (-0.58 to 0.10)	-0.11 (-0.76 to 0.54)	-0.07 (-0.59 to 0.45)	-0.06 (-0.70 to 0.57)	**Chlorpromazine**	-0.21 (-0.59 to 0.16)
**-0.54 (-0.92 to -0.16)**	-0.71 (-1.58 to 0.15)	-0.45 (-0.89 to 0.00)	-0.44 (-0.88 to 0.00)	-0.25 (-0.63 to 0.13)	-0.13 (-0.81 to 0.56)	-0.08 (-0.69 to 0.53)	-0.08 (-0.73 to 0.58)	-0.01 (-0.34 to 0.31)	**Quetiapine**

Treatments are presented in order of efficacy ranking. Results of the network meta-analysis are reported in the left lower half and results of pairwise meta-analyses in the right upper half. Each cell provides the effect estimate and the corresponding 95% credible interval (95% CI) of a comparison (left lower half: treatment in column versus treatment in row; right upper half: treatment in row versus treatment in column). The type of effect size measure is standardized mean difference (SMD). Bold results indicate 95% CI excluding no effect. NA=not available.

b Low dose

Number of studies: k = 19

Number of pairwise comparisons: m = 26

Number of treatments: n = 9

Number of designs: d = 11

Network plot

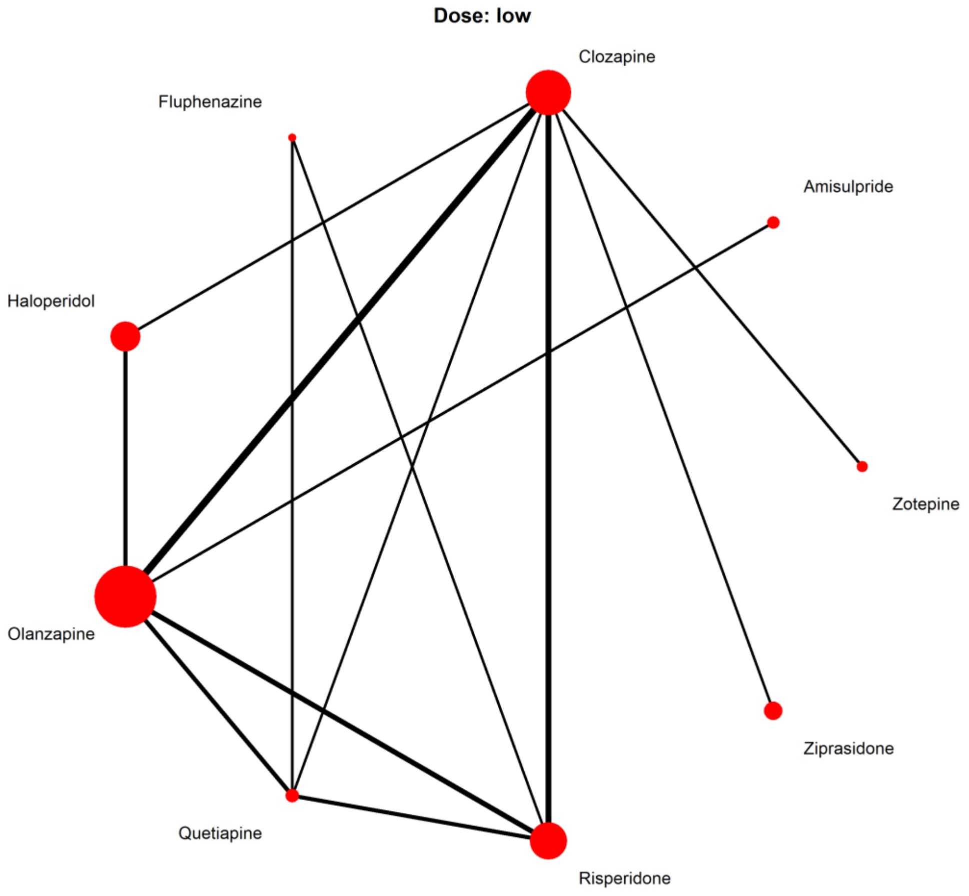

Lines link treatments with direct comparisons in trials; thickness of lines corresponds to the number of trials evaluating the comparison; size of the nodes corresponds to the number of participants assigned to the treatment.

τ	P-value	Inconsistency loop (%)
0.1233	0.0817	10.0

Forest-plot of results of network meta-analysis for antipsychotic drugs versus clozapine

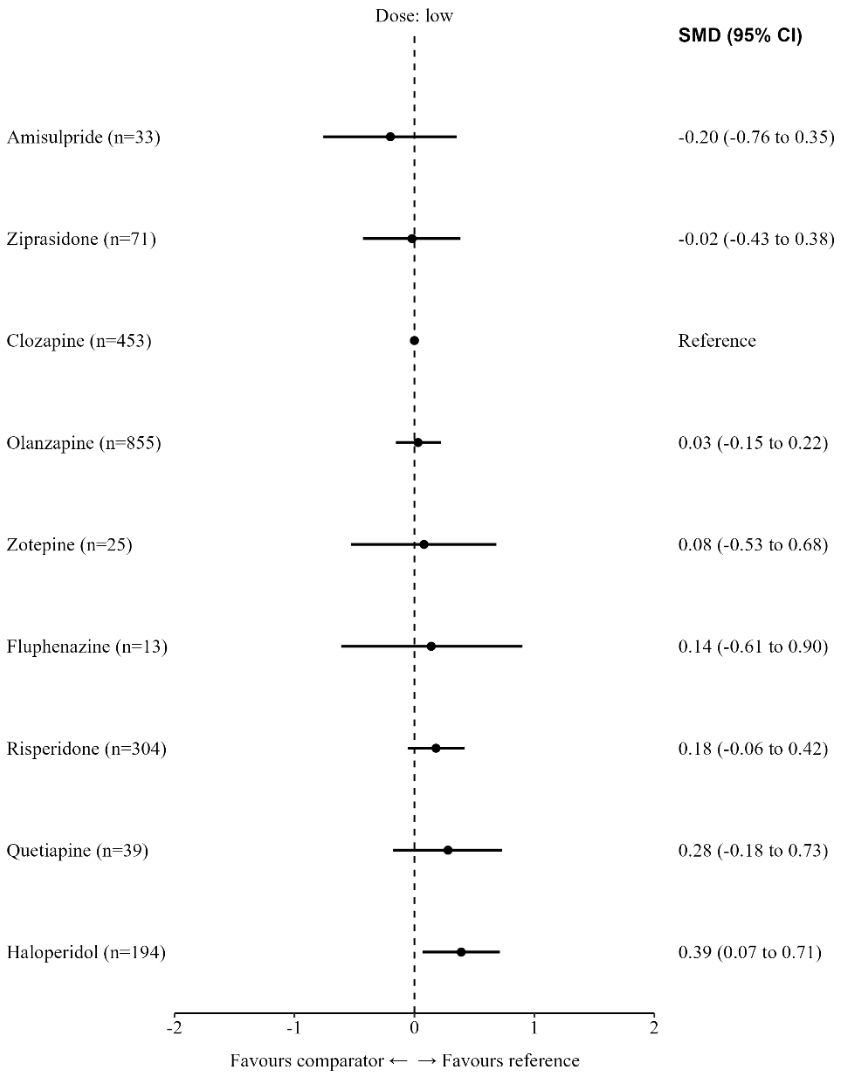

Effect sizes, measured as standardized mean difference (SMD), are from the network meta-analysis. Order of treatments is according to the mean effect size. Reference is clozapine. The direction of the effect is indicated below the x-axis.

League table

**Amisulpride**	NA	NA	-0.24 (-0.76 to 0.29)	NA	NA	NA	NA	NA
-0.20 (-0.76 to 0.35)	**Clozapine**	0.02 (-0.38 to 0.43)	-0.03 (-0.25 to 0.18)	-0.08 (-0.68 to 0.53)	NA	-0.13 (-0.46 to 0.19)	**-1.01 (-1.84 to -0.18)**	-0.78 (-1.70 to 0.14)
-0.18 (-0.87 to 0.51)	0.02 (-0.38 to 0.43)	**Ziprasidone**	NA	NA	NA	NA	NA	NA
-0.24 (-0.76 to 0.29)	-0.03 (-0.22 to 0.15)	-0.06 (-0.50 to 0.39)	**Olanzapine**	NA	NA	-0.23 (-0.51 to 0.05)	-0.06 (-0.61 to 0.49)	**-0.32 (-0.61 to -0.03)**
-0.28 (-1.10 to 0.54)	-0.08 (-0.68 to 0.53)	-0.10 (-0.83 to 0.63)	-0.04 (-0.68 to 0.59)	**Zotepine**	NA	NA	NA	NA
-0.35 (-1.26 to 0.57)	-0.14 (-0.90 to 0.61)	-0.17 (-1.03 to 0.69)	-0.11 (-0.86 to 0.64)	-0.07 (-1.03 to 0.90)	**Fluphenazine**	-0.02 (-0.83 to 0.78)	-0.15 (-0.97 to 0.67)	NA
-0.38 (-0.96 to 0.19)	-0.18 (-0.42 to 0.06)	-0.20 (-0.67 to 0.27)	-0.15 (-0.37 to 0.08)	-0.10 (-0.75 to 0.55)	-0.04 (-0.77 to 0.70)	**Risperidone**	-0.06 (-0.71 to 0.60)	NA
-0.48 (-1.16 to 0.21)	-0.28 (-0.73 to 0.18)	-0.30 (-0.91 to 0.31)	-0.24 (-0.68 to 0.20)	-0.20 (-0.96 to 0.56)	-0.13 (-0.87 to 0.61)	-0.09 (-0.55 to 0.36)	**Quetiapine**	NA
-0.59 (-1.18 to 0.00)	**-0.39 (-0.71 to -0.07)**	-0.41 (-0.93 to 0.11)	**-0.36 (-0.63 to -0.08)**	-0.31 (-1.00 to 0.37)	-0.25 (-1.04 to 0.55)	-0.21 (-0.56 to 0.14)	-0.11 (-0.63 to 0.40)	**Haloperidol**

Treatments are presented in order of efficacy ranking. Results of the network meta-analysis are reported in the left lower half and results of pairwise meta-analyses in the right upper half. Each cell provides the effect estimate and the corresponding 95% credible interval (95% CI) of a comparison (left lower half: treatment in column versus treatment in row; right upper half: treatment in row versus treatment in column). The type of effect size measure is standardized mean difference (SMD). Bold results indicate 95% CI excluding no effect. NA=not available.

12.4 Publication year

For the purpose of this analysis the median number of publication year was used.

a Early

Number of studies: k = 23

Number of pairwise comparisons: m = 23

Number of treatments: n = 8

Number of designs: d = 11

Network plot

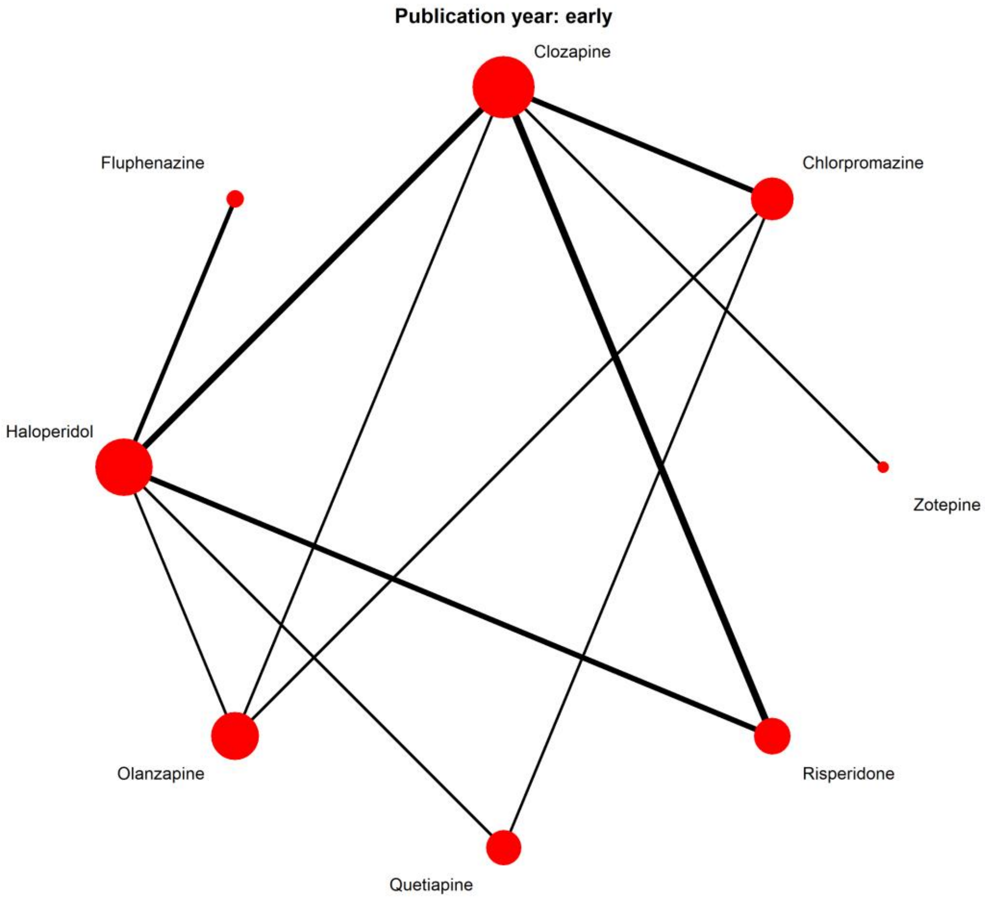

Lines link treatments with direct comparisons in trials; thickness of lines corresponds to the number of trials evaluating the comparison; size of the nodes corresponds to the number of participants assigned to the treatment.

τ	P-value	Inconsistency loop (%)
0.1966	0.0136	33.33

Forest-plot of results of network meta-analysis for antipsychotic drugs versus clozapine

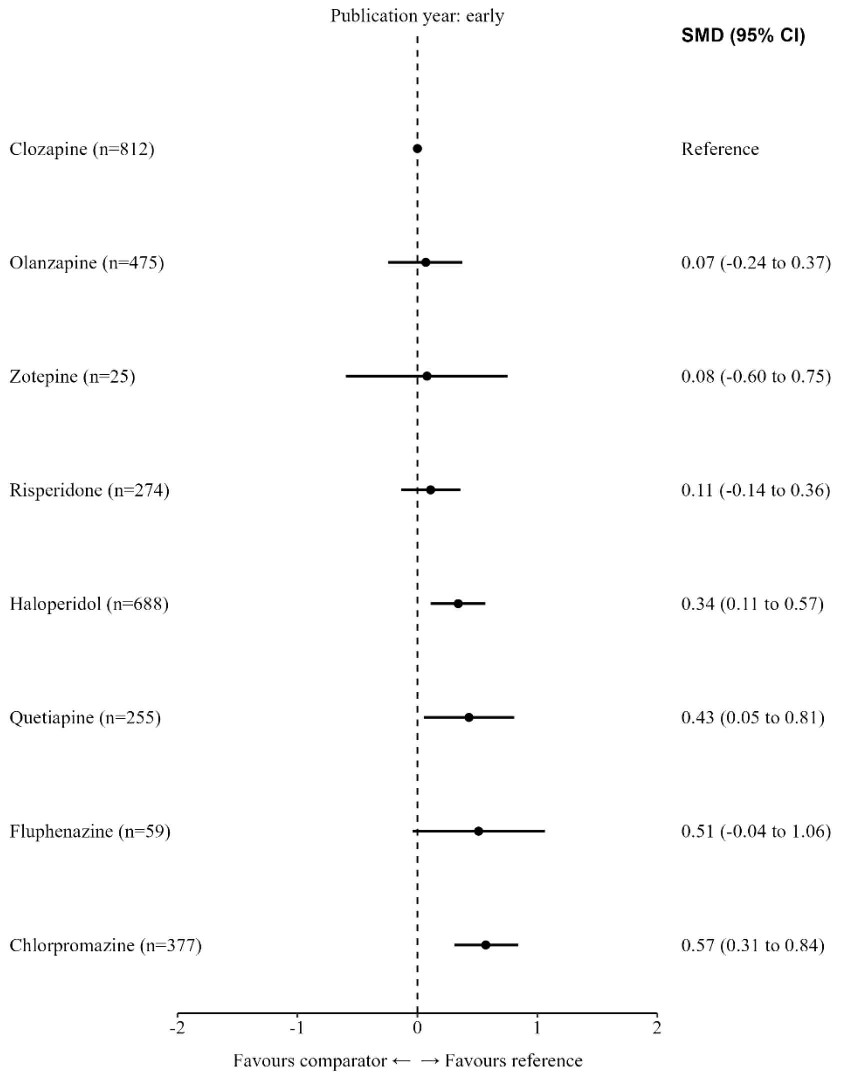

Effect sizes, measured as standardized mean difference (SMD), are from the network meta-analysis. Order of treatments is according to the mean effect size. Reference is clozapine. The direction of the effect is indicated below the x-axis.

League table

**Clozapine**	0.14 (-0.34 to 0.63)	-0.08 (-0.37 to 0.20)	-0.08 (-0.75 to 0.60)	-0.26 (-0.57 to 0.04)	NA	NA	**-0.77 (-1.09 to -0.46)**
-0.07 (-0.37 to 0.24)	**Olanzapine**	NA	NA	-0.27 (-0.70 to 0.16)	NA	NA	-0.22 (-0.80 to 0.37)
-0.11 (-0.36 to 0.14)	-0.05 (-0.42 to 0.33)	**Risperidone**	NA	-0.16 (-0.59 to 0.27)	NA	NA	NA
-0.08 (-0.75 to 0.60)	-0.01 (-0.76 to 0.73)	0.03 (-0.69 to 0.75)	**Zotepine**	NA	NA	NA	NA
**-0.34 (-0.57 to -0.11)**	-0.27 (-0.58 to 0.04)	-0.23 (-0.51 to 0.06)	-0.26 (-0.97 to 0.45)	**Haloperidol**	0.14 (-0.31 to 0.59)	-0.17 (-0.68 to 0.33)	NA
**-0.43 (-0.81 to -0.05)**	-0.37 (-0.80 to 0.07)	-0.32 (-0.75 to 0.11)	-0.35 (-1.13 to 0.42)	-0.09 (-0.45 to 0.26)	**Quetiapine**	NA	0.11 (-0.36 to 0.57)
-0.51 (-1.06 to 0.04)	-0.45 (-1.04 to 0.14)	-0.40 (-0.98 to 0.18)	-0.43 (-1.31 to 0.44)	-0.17 (-0.68 to 0.33)	-0.08 (-0.70 to 0.53)	**Fluphenazine**	NA
**-0.57 (-0.84 to -0.31)**	**-0.51 (-0.86 to -0.16)**	**-0.46 (-0.82 to -0.11)**	-0.50 (-1.22 to 0.23)	-0.24 (-0.55 to 0.08)	-0.14 (-0.50 to 0.22)	-0.06 (-0.65 to 0.53)	**Chlorpromazine**

Treatments are presented in order of efficacy ranking. Results of the network meta-analysis are reported in the left lower half and results of pairwise meta-analyses in the right upper half. Each cell provides the effect estimate and the corresponding 95% credible interval (95% CI) of a comparison (left lower half: treatment in column versus treatment in row; right upper half: treatment in row versus treatment in column). The type of effect size measure is standardized mean difference (SMD). Bold results indicate 95% CI excluding no effect. NA=not available.

b Late

Number of studies: k = 22

Number of pairwise comparisons: m = 34

Number of treatments: n = 11

Number of designs: d = 14

Network plot

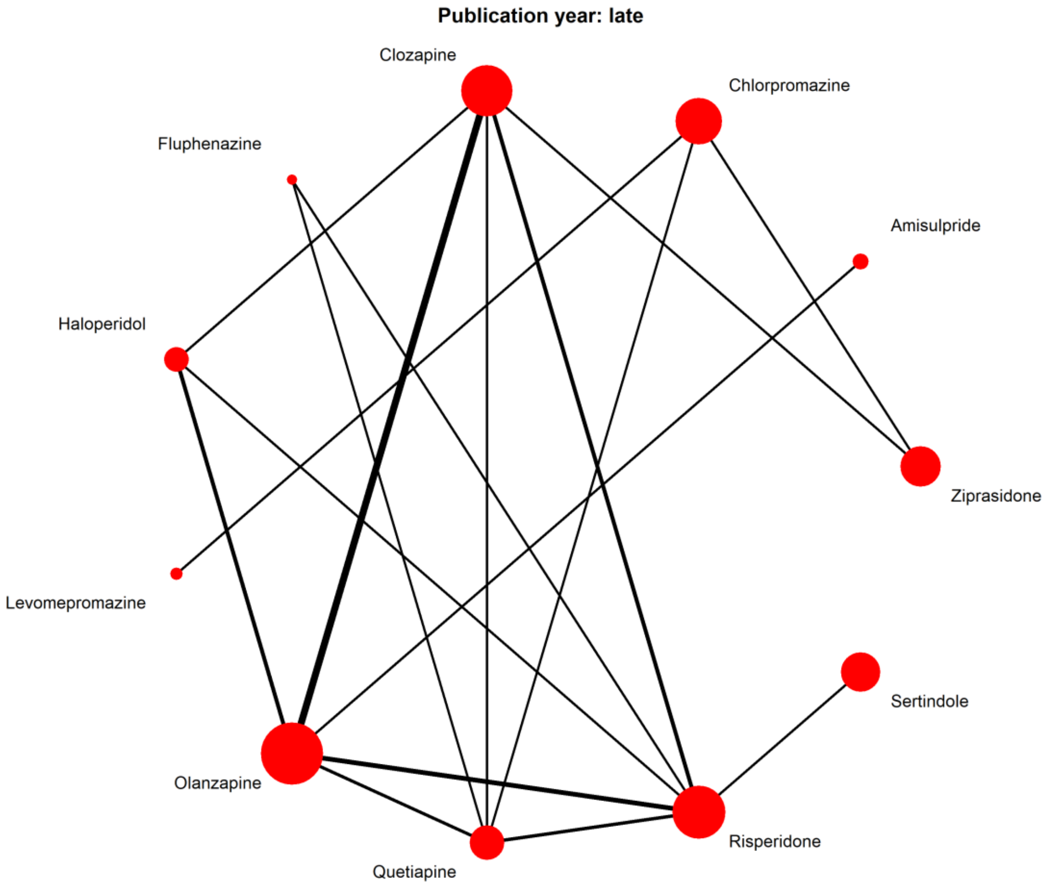

Lines link treatments with direct comparisons in trials; thickness of lines corresponds to the number of trials evaluating the comparison; size of the nodes corresponds to the number of participants assigned to the treatment.

τ	P-value	Inconsistency loop (%)
0.1125	0.0935	14.29

Forest-plot of results of network meta-analysis for antipsychotic drugs versus clozapine

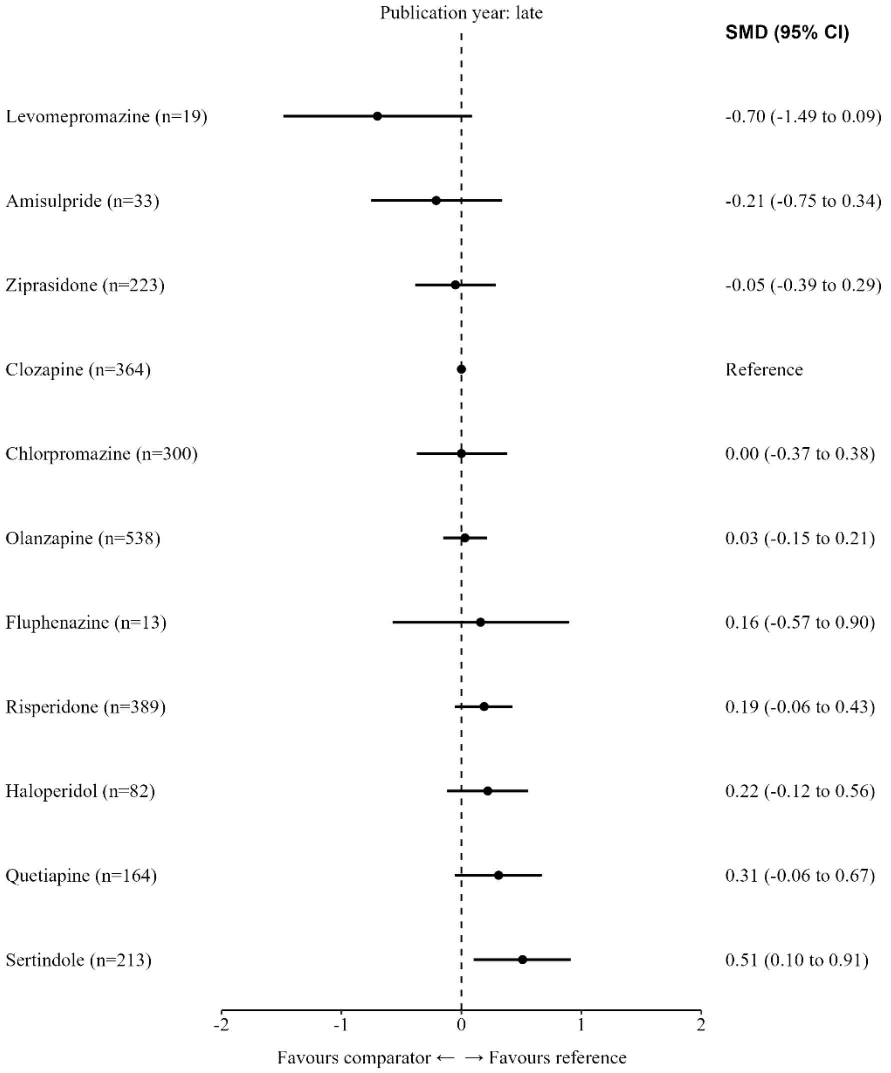

Effect sizes, measured as standardized mean difference (SMD), are from the network meta-analysis. Order of treatments is according to the mean effect size. Reference is clozapine. The direction of the effect is indicated below the x-axis.

League table

**Levomepromazine**	NA	NA	NA	**-0.70 (-1.39 to -0.01)**	NA	NA	NA	NA	NA	NA
-0.49 (-1.44 to 0.45)	**Amisulpride**	NA	NA	NA	-0.24 (-0.75 to 0.28)	NA	NA	NA	NA	NA
-0.65 (-1.40 to 0.10)	-0.16 (-0.79 to 0.47)	**Ziprasidone**	-0.02 (-0.42 to 0.37)	-0.07 (-0.39 to 0.24)	NA	NA	NA	NA	NA	NA
-0.70 (-1.49 to 0.09)	-0.21 (-0.75 to 0.34)	-0.05 (-0.39 to 0.29)	**Clozapine**	NA	-0.03 (-0.23 to 0.17)	NA	-0.02 (-0.37 to 0.33)	0.12 (-0.38 to 0.62)	**-1.01 (-1.83 to -0.19)**	NA
**-0.70 (-1.39 to -0.01)**	-0.21 (-0.86 to 0.44)	-0.05 (-0.34 to 0.23)	-0.00 (-0.38 to 0.37)	**Chlorpromazine**	NA	NA	NA	NA	-0.32 (-0.65 to 0.01)	NA
-0.73 (-1.52 to 0.07)	-0.24 (-0.75 to 0.28)	-0.08 (-0.44 to 0.28)	-0.03 (-0.21 to 0.15)	-0.03 (-0.42 to 0.36)	**Olanzapine**	NA	-0.23 (-0.47 to 0.00)	-0.29 (-0.62 to 0.05)	-0.06 (-0.60 to 0.49)	NA
-0.86 (-1.89 to 0.17)	-0.37 (-1.26 to 0.53)	-0.21 (-0.98 to 0.56)	-0.16 (-0.90 to 0.57)	-0.16 (-0.92 to 0.60)	-0.13 (-0.86 to 0.60)	**Fluphenazine**	-0.02 (-0.82 to 0.78)	NA	-0.15 (-0.96 to 0.67)	NA
**-0.88 (-1.69 to -0.08)**	-0.39 (-0.95 to 0.16)	-0.24 (-0.63 to 0.16)	-0.19 (-0.43 to 0.06)	-0.18 (-0.60 to 0.23)	-0.16 (-0.37 to 0.05)	-0.02 (-0.75 to 0.70)	**Risperidone**	-0.12 (-0.62 to 0.37)	-0.06 (-0.71 to 0.59)	-0.32 (-0.64 to 0.00)
**-0.92 (-1.76 to -0.07)**	-0.43 (-1.03 to 0.18)	-0.27 (-0.73 to 0.20)	-0.22 (-0.56 to 0.12)	-0.21 (-0.70 to 0.27)	-0.19 (-0.50 to 0.12)	-0.06 (-0.84 to 0.73)	-0.03 (-0.38 to 0.31)	**Haloperidol**	NA	NA
**-1.01 (-1.76 to -0.25)**	-0.51 (-1.14 to 0.12)	-0.36 (-0.72 to 0.01)	-0.31 (-0.67 to 0.06)	-0.30 (-0.60 to 0.00)	-0.28 (-0.64 to 0.09)	-0.15 (-0.87 to 0.58)	-0.12 (-0.51 to 0.27)	-0.09 (-0.56 to 0.38)	**Quetiapine**	NA
**-1.20 (-2.07 to -0.34)**	**-0.71 (-1.36 to -0.07)**	**-0.56 (-1.06 to -0.05)**	**-0.51 (-0.91 to -0.10)**	-0.50 (-1.03 to 0.02)	**-0.48 (-0.86 to -0.09)**	-0.34 (-1.13 to 0.45)	-0.32 (-0.64 to 0.00)	-0.29 (-0.76 to 0.19)	-0.20 (-0.70 to 0.31)	**Sertindole**

Treatments are presented in order of efficacy ranking. Results of the network meta-analysis are reported in the left lower half and results of pairwise meta-analyses in the right upper half. Each cell provides the effect estimate and the corresponding 95% credible interval (95% CI) of a comparison (left lower half: treatment in column versus treatment in row; right upper half: treatment in row versus treatment in column). The type of effect size measure is standardized mean difference (SMD). Bold results indicate 95% CI excluding no effect. NA=not available.

12.5 Baseline severity

For the purpose of this analysis the median baseline severity of each scale was used.

a High

Number of studies: k = 21

Number of pairwise comparisons: m = 23

Number of treatments: n = 10

Number of designs: d = 12

Network plot

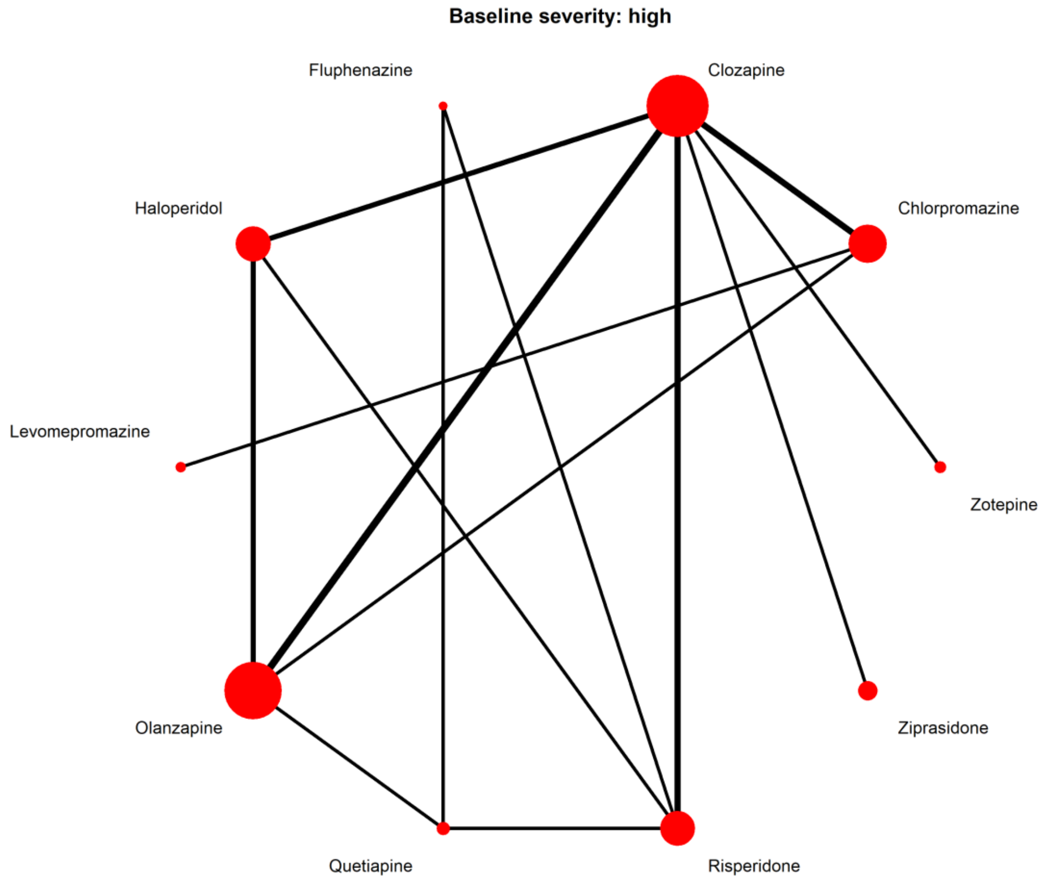

Lines link treatments with direct comparisons in trials; thickness of lines corresponds to the number of trials evaluating the comparison; size of the nodes corresponds to the number of participants assigned to the treatment.

τ	P-value	Inconsistency loop (%)
0.0986	0.1524	27.27

Forest-plot of results of network meta-analysis for antipsychotic drugs versus clozapine

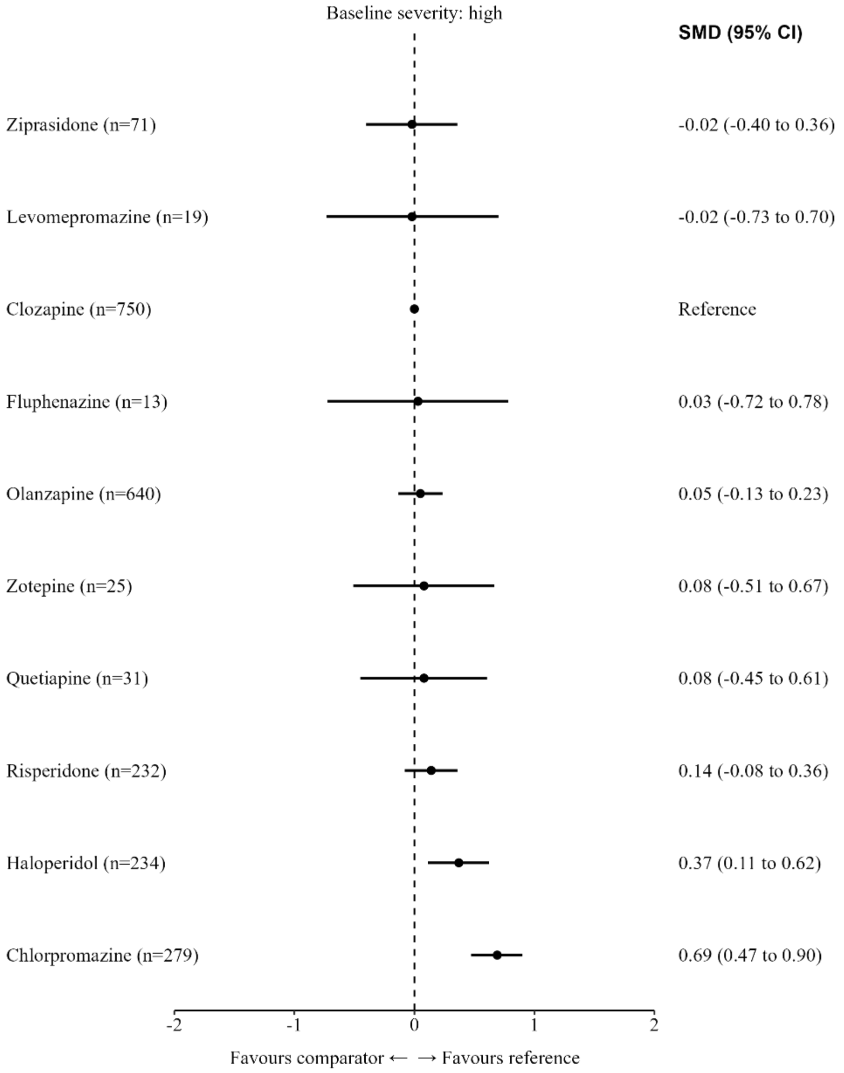

Effect sizes, measured as standardized mean difference (SMD), are from the network meta-analysis. Order of treatments is according to the mean effect size. Reference is clozapine. The direction of the effect is indicated below the x-axis.

League table

**Clozapine**	0.02 (-0.36 to 0.40)	NA	NA	0.05 (-0.16 to 0.27)	NA	-0.08 (-0.67 to 0.51)	-0.14 (-0.38 to 0.10)	-0.47 (-1.05 to 0.10)	**-0.79 (-1.02 to -0.55)**
0.02 (-0.36 to 0.40)	**Ziprasidone**	NA	NA	NA	NA	NA	NA	NA	NA
0.02 (-0.70 to 0.73)	-0.01 (-0.82 to 0.80)	**Levomepromazine**	NA	NA	NA	NA	NA	NA	**-0.70 (-1.39 to -0.02)**
-0.03 (-0.78 to 0.72)	-0.05 (-0.89 to 0.79)	-0.04 (-1.08 to 0.99)	**Fluphenazine**	NA	-0.15 (-0.96 to 0.66)	NA	-0.02 (-0.81 to 0.77)	NA	NA
-0.05 (-0.23 to 0.13)	-0.07 (-0.50 to 0.35)	-0.07 (-0.80 to 0.66)	-0.02 (-0.78 to 0.73)	**Olanzapine**	0.09 (-0.56 to 0.74)	NA	NA	**-0.31 (-0.56 to -0.06)**	-0.22 (-0.69 to 0.26)
-0.08 (-0.61 to 0.45)	-0.10 (-0.75 to 0.55)	-0.09 (-0.98 to 0.79)	-0.05 (-0.79 to 0.69)	-0.03 (-0.54 to 0.49)	**Quetiapine**	NA	0.12 (-0.68 to 0.93)	NA	NA
-0.08 (-0.67 to 0.51)	-0.10 (-0.80 to 0.60)	-0.09 (-1.02 to 0.83)	-0.05 (-1.00 to 0.90)	-0.03 (-0.64 to 0.59)	-0.00 (-0.79 to 0.79)	**Zotepine**	NA	NA	NA
-0.14 (-0.36 to 0.08)	-0.16 (-0.60 to 0.28)	-0.16 (-0.90 to 0.59)	-0.11 (-0.85 to 0.62)	-0.09 (-0.36 to 0.18)	-0.06 (-0.59 to 0.47)	-0.06 (-0.69 to 0.57)	**Risperidone**	-0.16 (-0.71 to 0.39)	NA
**-0.37 (-0.62 to -0.11)**	-0.39 (-0.85 to 0.07)	-0.38 (-1.14 to 0.37)	-0.34 (-1.11 to 0.44)	**-0.32 (-0.54 to -0.09)**	-0.29 (-0.84 to 0.26)	-0.29 (-0.93 to 0.35)	-0.23 (-0.53 to 0.07)	**Haloperidol**	NA
**-0.69 (-0.90 to -0.47)**	**-0.71 (-1.14 to -0.27)**	**-0.70 (-1.39 to -0.02)**	-0.66 (-1.44 to 0.12)	**-0.63 (-0.89 to -0.38)**	**-0.61 (-1.17 to -0.05)**	-0.61 (-1.23 to 0.02)	**-0.55 (-0.85 to -0.24)**	-0.32 (-0.64 to 0.00)	**Chlorpromazine**

Treatments are presented in order of efficacy ranking. Results of the network meta-analysis are reported in the left lower half and results of pairwise meta-analyses in the right upper half. Each cell provides the effect estimate and the corresponding 95% credible interval (95% CI) of a comparison (left lower half: treatment in column versus treatment in row; right upper half: treatment in row versus treatment in column). The type of effect size measure is standardized mean difference (SMD). Bold results indicate 95% CI excluding no effect. NA=not available.

b Low

Number of studies: k = 19

Number of pairwise comparisons: m = 26

Number of treatments: n = 8

Number of designs: d = 11

Network plot

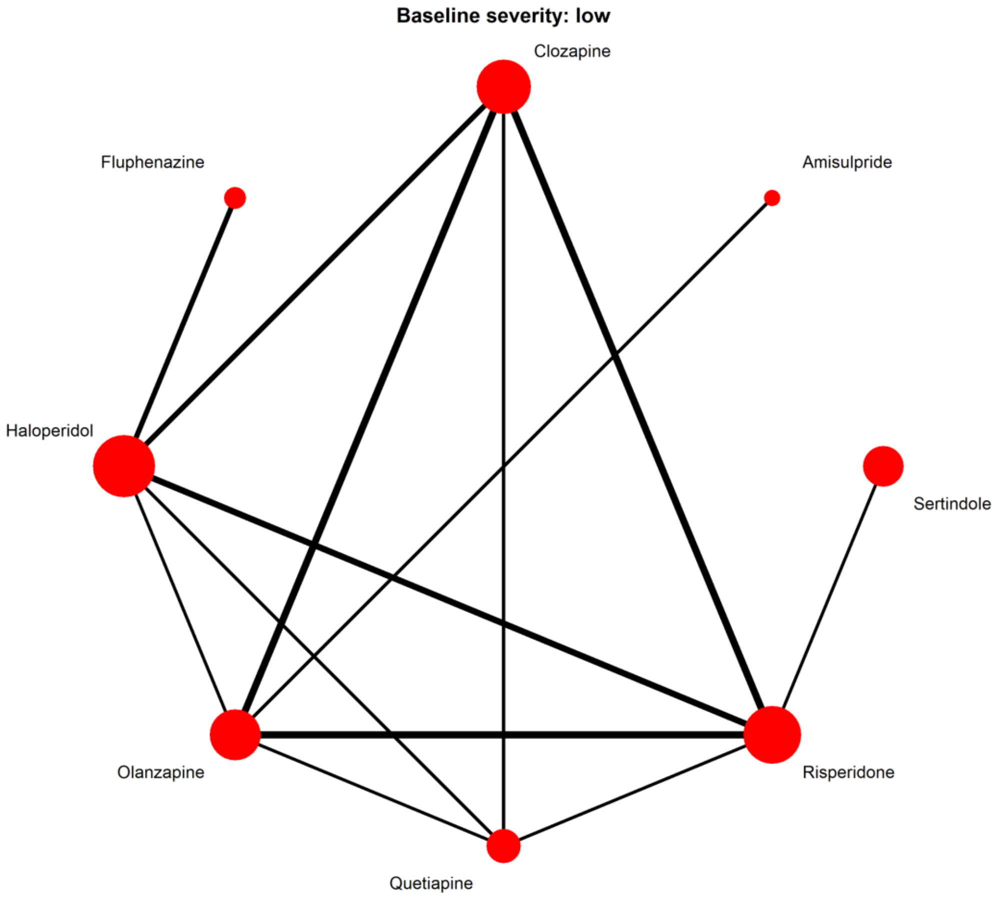

Lines link treatments with direct comparisons in trials; thickness of lines corresponds to the number of trials evaluating the comparison; size of the nodes corresponds to the number of participants assigned to the treatment.

τ	P-value	Inconsistency loop (%)
0.0925	0.1893	20.0

Forest-plot of results of network meta-analysis for antipsychotic drugs versus clozapine

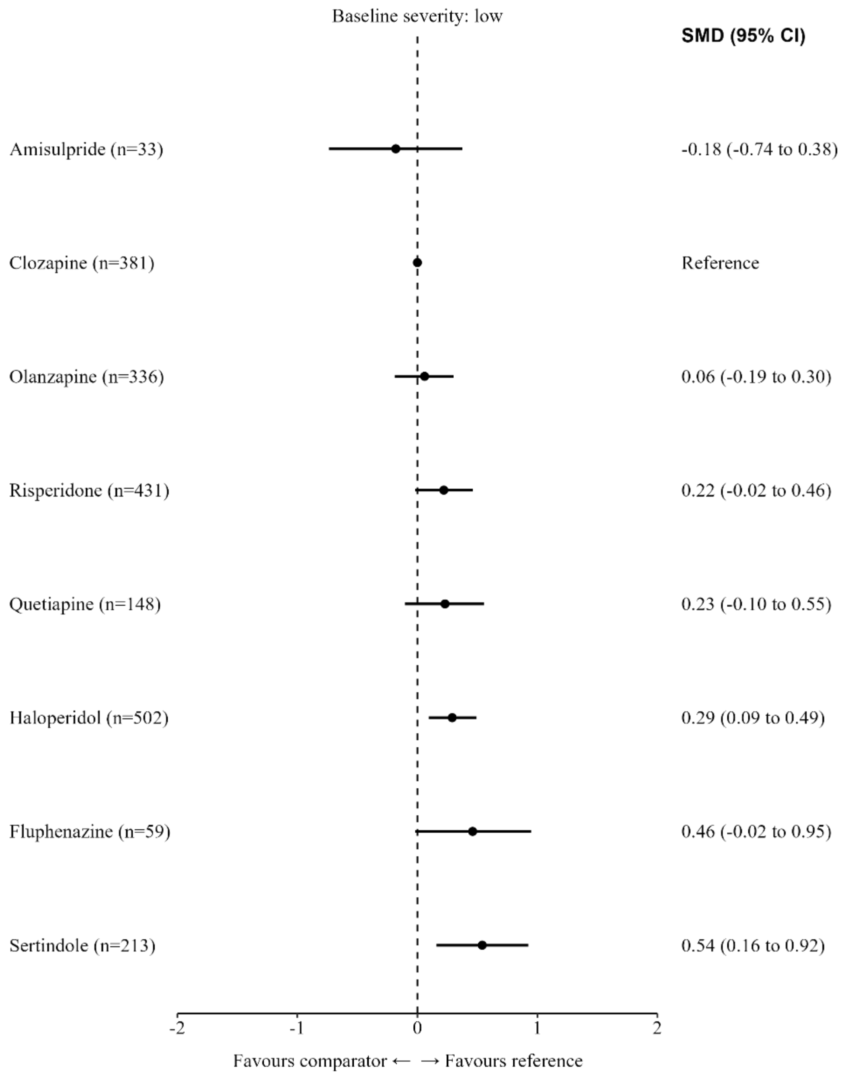

Effect sizes, measured as standardized mean difference (SMD), are from the network meta-analysis. Order of treatments is according to the mean effect size. Reference is clozapine. The direction of the effect is indicated below the x-axis.

League table

**Amisulpride**	NA	-0.24 (-0.74 to 0.26)	NA	NA	NA	NA	NA
-0.18 (-0.74 to 0.38)	**Clozapine**	-0.28 (-0.63 to 0.07)	**-1.01 (-1.82 to -0.20)**	-0.18 (-0.55 to 0.20)	-0.21 (-0.44 to 0.02)	NA	NA
-0.24 (-0.74 to 0.26)	-0.06 (-0.30 to 0.19)	**Olanzapine**	-0.38 (-1.33 to 0.57)	**-0.23 (-0.45 to -0.01)**	-0.36 (-0.85 to 0.12)	NA	NA
-0.41 (-1.03 to 0.21)	-0.23 (-0.55 to 0.10)	-0.17 (-0.54 to 0.20)	**Quetiapine**	-0.06 (-1.13 to 1.01)	-0.14 (-0.44 to 0.15)	NA	NA
-0.40 (-0.94 to 0.14)	-0.22 (-0.46 to 0.02)	-0.17 (-0.37 to 0.04)	0.00 (-0.36 to 0.37)	**Risperidone**	-0.13 (-0.48 to 0.22)	NA	**-0.32 (-0.62 to -0.02)**
-0.47 (-1.04 to 0.09)	**-0.29 (-0.49 to -0.09)**	-0.24 (-0.50 to 0.03)	-0.07 (-0.35 to 0.21)	-0.07 (-0.32 to 0.18)	**Haloperidol**	-0.17 (-0.61 to 0.27)	NA
-0.65 (-1.36 to 0.07)	-0.46 (-0.95 to 0.02)	-0.41 (-0.92 to 0.10)	-0.24 (-0.76 to 0.28)	-0.24 (-0.75 to 0.26)	-0.17 (-0.61 to 0.27)	**Fluphenazine**	NA
**-0.72 (-1.34 to -0.11)**	**-0.54 (-0.92 to -0.16)**	**-0.49 (-0.85 to -0.13)**	-0.32 (-0.78 to 0.15)	**-0.32 (-0.62 to -0.02)**	-0.25 (-0.64 to 0.14)	-0.08 (-0.66 to 0.51)	**Sertindole**

Treatments are presented in order of efficacy ranking. Results of the network meta-analysis are reported in the left lower half and results of pairwise meta-analyses in the right upper half. Each cell provides the effect estimate and the corresponding 95% credible interval (95% CI) of a comparison (left lower half: treatment in column versus treatment in row; right upper half: treatment in row versus treatment in column). The type of effect size measure is standardized mean difference (SMD). Bold results indicate 95% CI excluding no effect. NA=not available.

12.6 Study duration

For the purpose of this analysis the median number of study duration was used.

a Short

Number of studies: k = 21

Number of pairwise comparisons: m = 21

Number of treatments: n = 9

Number of designs: d = 13

Network plot

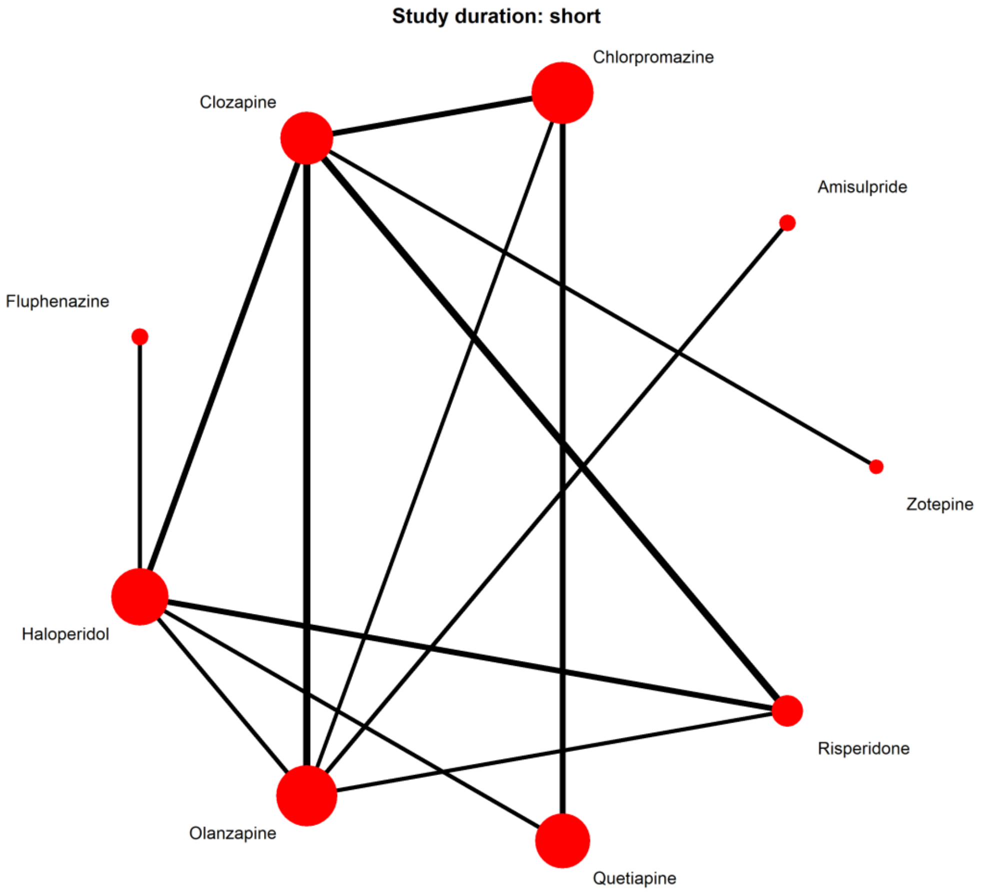

Lines link treatments with direct comparisons in trials; thickness of lines corresponds to the number of trials evaluating the comparison; size of the nodes corresponds to the number of participants assigned to the treatment.

τ	P-value	Inconsistency loop (%)
0.2106	0.0370	30.0

Forest-plot of results of network meta-analysis for antipsychotic drugs versus clozapine

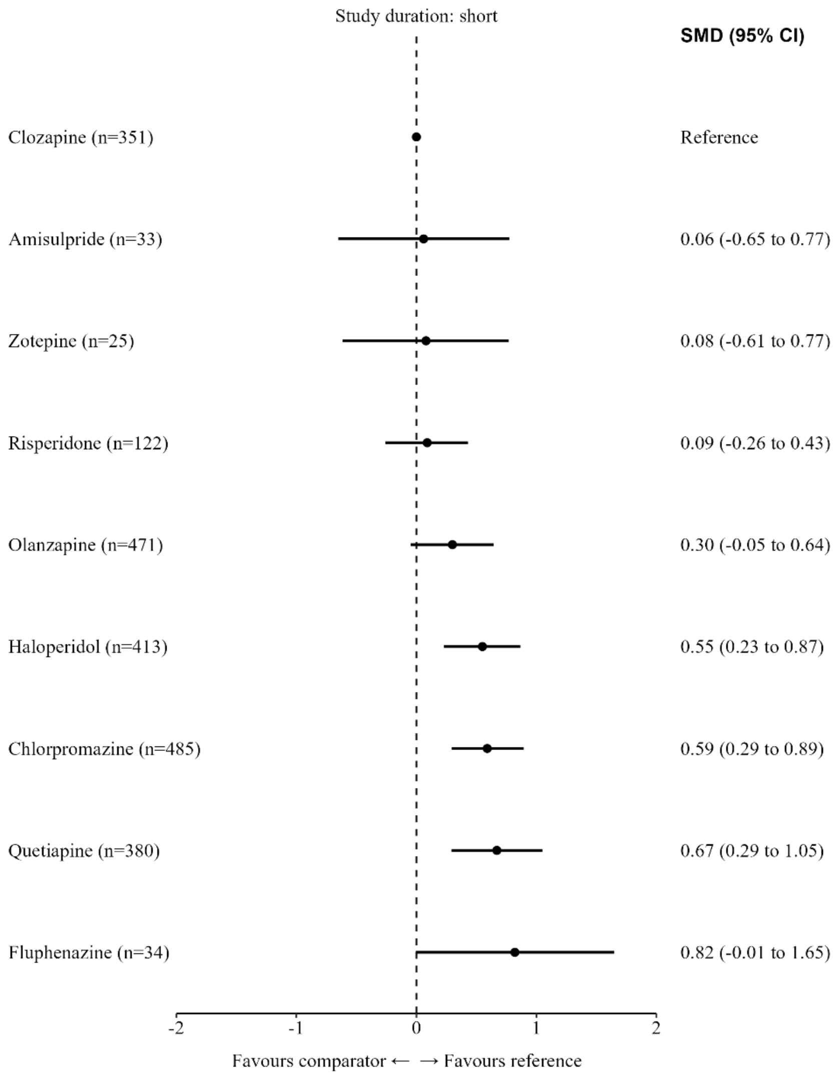

Effect sizes, measured as standardized mean difference (SMD), are from the network meta-analysis. Order of treatments is according to the mean effect size. Reference is clozapine. The direction of the effect is indicated below the x-axis.

League table

**Clozapine**	0.10 (-0.33 to 0.54)	NA	-0.08 (-0.77 to 0.61)	-0.46 (-1.06 to 0.15)	-0.32 (-0.84 to 0.20)	**-0.78 (-1.14 to -0.41)**	NA	NA
-0.09 (-0.43 to 0.26)	**Risperidone**	NA	NA	0.15 (-0.66 to 0.96)	-0.33 (-0.88 to 0.22)	NA	NA	NA
-0.06 (-0.77 to 0.65)	0.02 (-0.72 to 0.77)	**Amisulpride**	NA	-0.24 (-0.86 to 0.39)	NA	NA	NA	NA
-0.08 (-0.77 to 0.61)	0.01 (-0.77 to 0.78)	-0.02 (-1.01 to 0.98)	**Zotepine**	NA	NA	NA	NA	NA
-0.30 (-0.64 to 0.05)	-0.21 (-0.62 to 0.19)	-0.24 (-0.86 to 0.39)	-0.22 (-0.99 to 0.55)	**Olanzapine**	-0.27 (-0.72 to 0.18)	-0.22 (-0.82 to 0.39)	NA	NA
**-0.55 (-0.87 to -0.23)**	**-0.46 (-0.83 to -0.09)**	-0.49 (-1.19 to 0.22)	-0.47 (-1.23 to 0.29)	-0.25 (-0.58 to 0.08)	**Haloperidol**	NA	0.14 (-0.33 to 0.62)	-0.27 (-1.04 to 0.49)
**-0.59 (-0.89 to -0.29)**	**-0.51 (-0.93 to -0.09)**	-0.53 (-1.25 to 0.19)	-0.52 (-1.27 to 0.24)	-0.30 (-0.66 to 0.07)	-0.05 (-0.40 to 0.31)	**Chlorpromazine**	-0.22 (-0.56 to 0.13)	NA
**-0.67 (-1.05 to -0.29)**	**-0.58 (-1.04 to -0.13)**	-0.61 (-1.36 to 0.14)	-0.59 (-1.38 to 0.20)	-0.37 (-0.79 to 0.05)	-0.12 (-0.48 to 0.24)	-0.08 (-0.38 to 0.23)	**Quetiapine**	NA
-0.82 (-1.65 to 0.01)	-0.74 (-1.58 to 0.11)	-0.76 (-1.80 to 0.28)	-0.74 (-1.82 to 0.34)	-0.52 (-1.36 to 0.31)	-0.27 (-1.04 to 0.49)	-0.23 (-1.07 to 0.61)	-0.15 (-0.99 to 0.69)	**Fluphenazine**

Treatments are presented in order of efficacy ranking. Results of the network meta-analysis are reported in the left lower half and results of pairwise meta-analyses in the right upper half. Each cell provides the effect estimate and the corresponding 95% credible interval (95% CI) of a comparison (left lower half: treatment in column versus treatment in row; right upper half: treatment in row versus treatment in column). The type of effect size measure is standardized mean difference (SMD). Bold results indicate 95% CI excluding no effect. NA=not available.

b Long

Number of studies: k = 24

Number of pairwise comparisons: m = 36

Number of treatments: n = 10

Number of designs: d = 16

Network plot

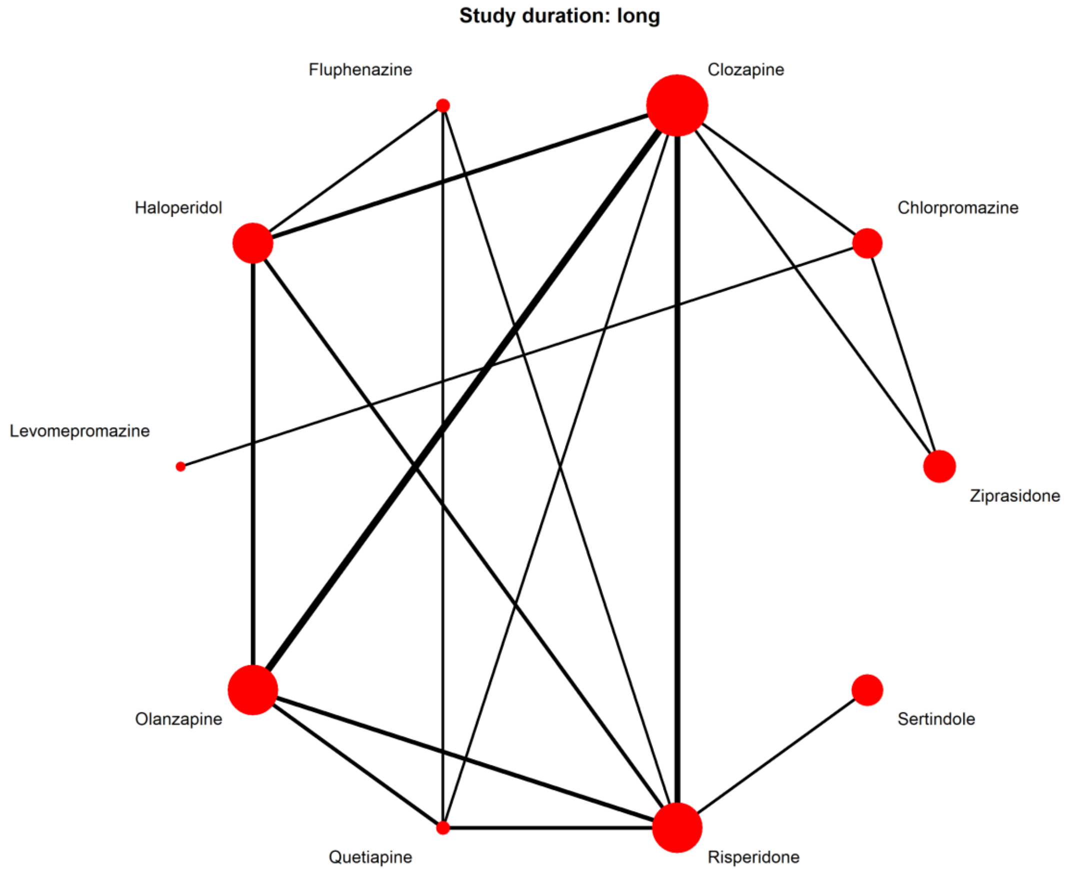

Lines link treatments with direct comparisons in trials; thickness of lines corresponds to the number of trials evaluating the comparison; size of the nodes corresponds to the number of participants assigned to the treatment.

τ	P-value	Inconsistency loop (%)
0.0893	0.1223	26.67

Forest-plot of results of network meta-analysis for antipsychotic drugs versus clozapine

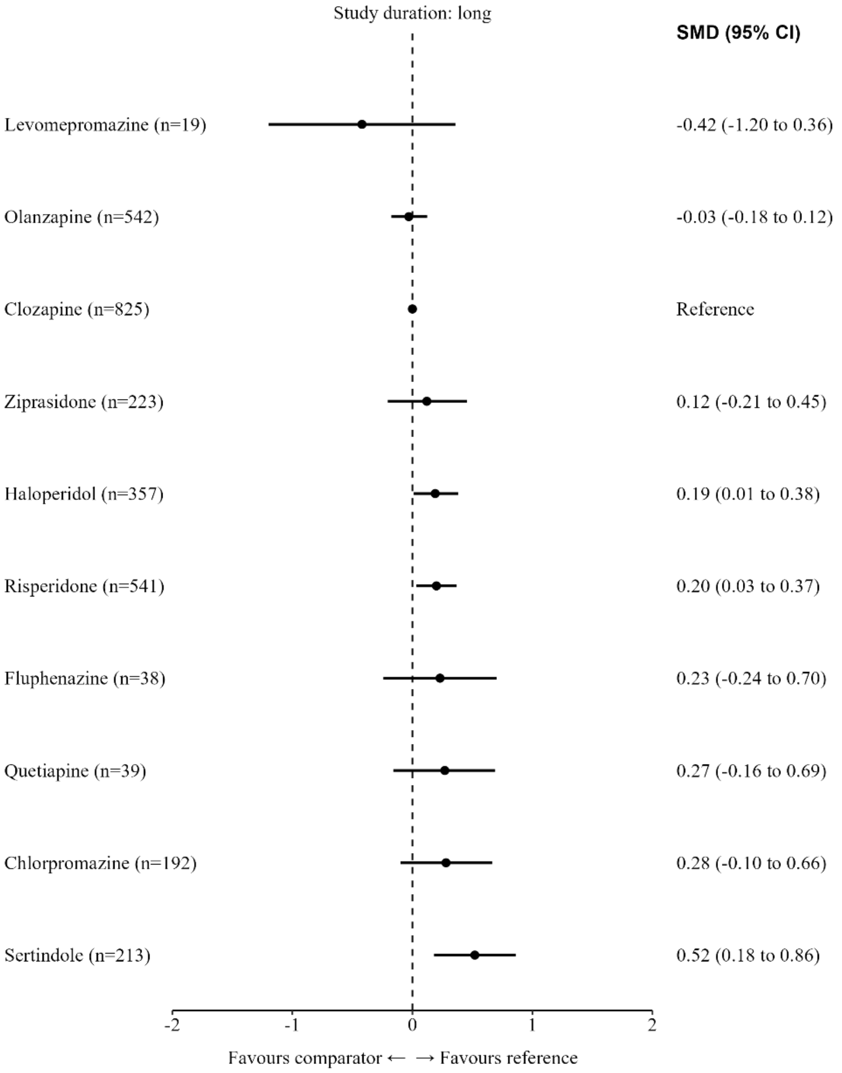

Effect sizes, measured as standardized mean difference (SMD), are from the network meta-analysis. Order of treatments is according to the mean effect size. Reference is clozapine. The direction of the effect is indicated below the x-axis.

League table

**Levomepromazine**	NA	NA	NA	NA	NA	NA	NA	**-0.70 (-1.38 to -0.02)**	NA
-0.39 (-1.19 to 0.40)	**Olanzapine**	-0.06 (-0.24 to 0.11)	NA	-0.28 (-0.61 to 0.05)	**-0.24 (-0.47 to -0.01)**	NA	-0.06 (-0.59 to 0.48)	NA	NA
-0.42 (-1.20 to 0.36)	-0.03 (-0.18 to 0.12)	**Clozapine**	0.02 (-0.35 to 0.39)	-0.16 (-0.38 to 0.06)	-0.17 (-0.38 to 0.05)	NA	**-1.01 (-1.82 to -0.20)**	**-0.76 (-1.42 to -0.09)**	NA
-0.54 (-1.27 to 0.18)	-0.15 (-0.51 to 0.21)	-0.12 (-0.45 to 0.21)	**Ziprasidone**	NA	NA	NA	NA	-0.07 (-0.36 to 0.21)	NA
-0.62 (-1.42 to 0.19)	**-0.22 (-0.43 to -0.01)**	**-0.19 (-0.38 to -0.01)**	-0.07 (-0.45 to 0.31)	**Haloperidol**	0.03 (-0.35 to 0.41)	-0.09 (-0.67 to 0.49)	NA	NA	NA
-0.62 (-1.42 to 0.18)	**-0.23 (-0.40 to -0.05)**	**-0.20 (-0.37 to -0.03)**	-0.07 (-0.45 to 0.30)	-0.00 (-0.22 to 0.22)	**Risperidone**	0.02 (-0.77 to 0.81)	-0.06 (-0.70 to 0.59)	NA	**-0.32 (-0.62 to -0.03)**
-0.65 (-1.56 to 0.26)	-0.26 (-0.73 to 0.22)	-0.23 (-0.70 to 0.24)	-0.11 (-0.68 to 0.47)	-0.04 (-0.49 to 0.42)	-0.03 (-0.50 to 0.44)	**Fluphenazine**	-0.15 (-0.95 to 0.66)	NA	NA
-0.69 (-1.57 to 0.20)	-0.29 (-0.71 to 0.13)	-0.27 (-0.69 to 0.16)	-0.14 (-0.68 to 0.40)	-0.07 (-0.51 to 0.37)	-0.07 (-0.49 to 0.36)	-0.04 (-0.60 to 0.53)	**Quetiapine**	NA	NA
**-0.70 (-1.38 to -0.02)**	-0.31 (-0.72 to 0.10)	-0.28 (-0.66 to 0.10)	-0.16 (-0.42 to 0.11)	-0.09 (-0.51 to 0.34)	-0.08 (-0.50 to 0.34)	-0.05 (-0.66 to 0.56)	-0.02 (-0.59 to 0.55)	**Chlorpromazine**	NA
**-0.94 (-1.79 to -0.09)**	**-0.55 (-0.89 to -0.20)**	**-0.52 (-0.86 to -0.18)**	-0.40 (-0.87 to 0.08)	-0.33 (-0.69 to 0.04)	**-0.32 (-0.62 to -0.03)**	-0.29 (-0.85 to 0.27)	-0.25 (-0.77 to 0.26)	-0.24 (-0.75 to 0.27)	**Sertindole**

Treatments are presented in order of efficacy ranking. Results of the network meta-analysis are reported in the left lower half and results of pairwise meta-analyses in the right upper half. Each cell provides the effect estimate and the corresponding 95% credible interval (95% CI) of a comparison (left lower half: treatment in column versus treatment in row; right upper half: treatment in row versus treatment in column). The type of effect size measure is standardized mean difference (SMD). Bold results indicate 95% CI excluding no effect. NA=not available.

13 Risk of bias Assessment

	Risk of bias domains					
Study	D1	D2	D3	D4	D5	Overall
Altamura 2002	+	+	X	+	+	X
AstraZeneca 5077IL/0031	+	+	X	+	+	X
AstraZeneca 5077IL/0054	+	+	X	+	+	X
Azorin 2001	+	+	-	+	+	-
Bitter 2004	+	+	-	+	+	-
Bondolfi 1998	+	+	-	+	+	-
Breier 1999	-	+	X	+	+	X
Breier 1999a	+	+	-	+	+	-
Buchanan 1998	-	+	X	+	+	X
Buchanan 2005	-	+	-	+	+	-
Chen 2012	-	-	-	+	+	-
Chowdhury 1999	-	-	-	-	+	X
Claus 1992	+	+	X	+	+	X
Conley 1998	+	+	-	+	+	-
Conley 2003	-	+	X	+	+	X
Conley 2005	+	+	X	+	+	X
Emsley 2000	+	+	-	+	+	-
Hall 1968	-	+	-	+	+	-
Hong 1997	-	+	-	+	+	-
Honigfeld 1984b	+	+	-	+	+	-
Kahn 2018	+	X	-	+	+	X
Kane 1988	+	+	-	+	+	-
Kane 2001	+	X	X	+	+	X
Kane 2006	+	+	-	+	+	-
Kane 2010b	+	+	-	+	+	-
Kinon 1993b	-	-	X	+	+	X
Kinon 2009	+	+	-	+	+	-

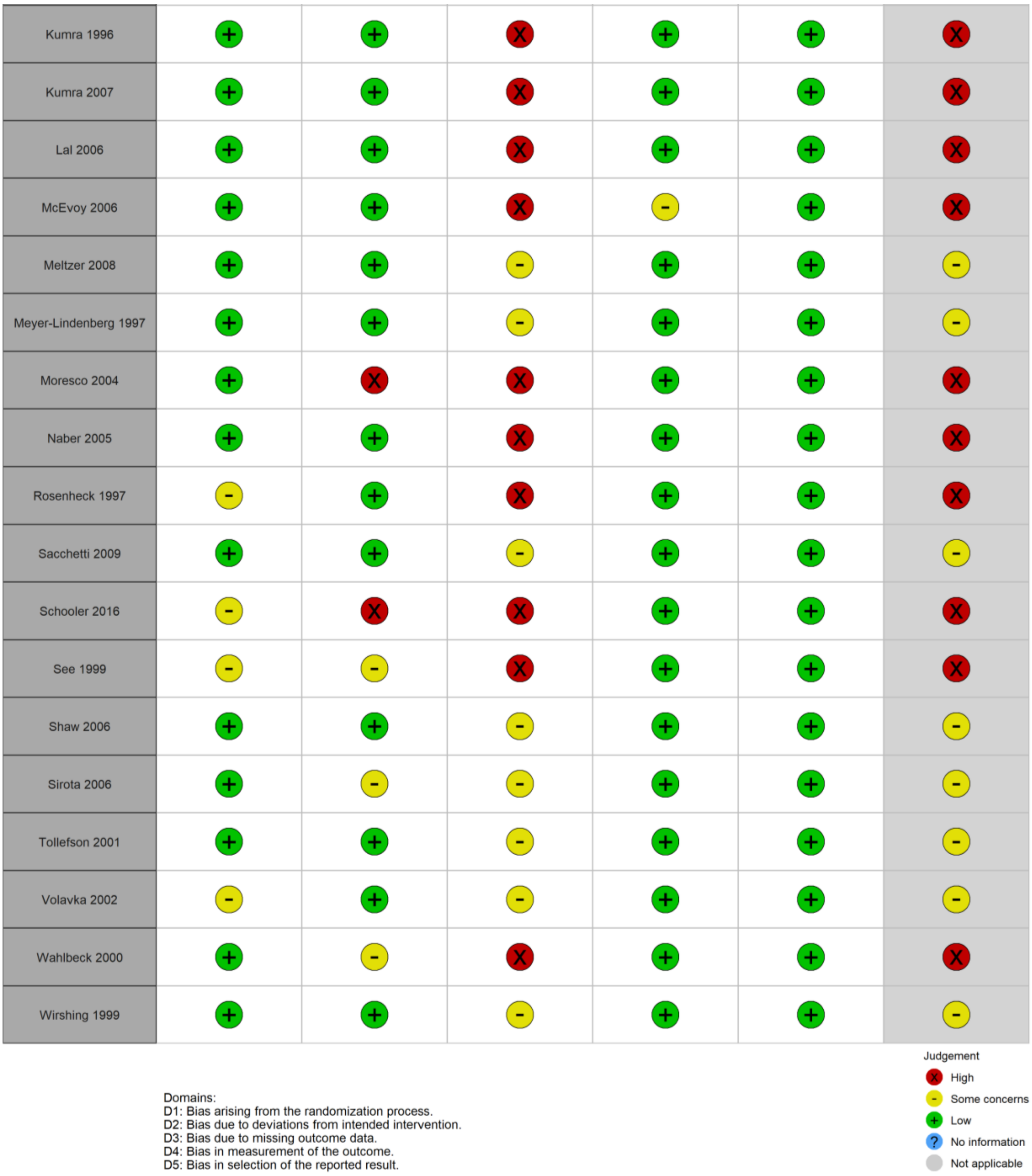
Kumra 1996
Kumra 2007
Lal 2006
McEvoy 2006
Meltzer 2008
Meyer-Lindenberg 1997
Moresco 2004
Naber 2005
Rosenheck 1997
Sacchetti 2009
Schooler 2016
See 1999
Shaw 2006
Sirota 2006
Tollefson 2001
Volavka 2002
Wahlbeck 2000
Wirshing 1999
Domains:
D1: Bias arising from the randomization process.
D2: Bias due to deviations from intended intervention.
D3: Bias due to missing outcome data.
D4: Bias in measurement of the outcome.
D5: Bias in selection of the reported result.
Judgement
High
Some concerns
Low
No information
Not applicable

14 Investigation of the small-study effect and publication bias

We investigated the presence of the small-study effect for the primary outcome of overall symptoms of schizophrenia. We used funnel plots for pairwise meta-analysis including 10 or more studies and comparison-adjusted funnel plots for network meta-analysis. In our study only one comparison, clozapine versus olanzapine, included 10 studies.

In comparison-adjusted funnel plots, comparisons have been directed according to the oldness and the effectiveness of the treatments: namely, all comparisons are "older interventions versus newer interventions" and "more efficacious interventions versus less efficacious interventions," respectively. Each comparison is represented by a different colored symbol in the graph. The x-axis shows the difference between the study effect and the summary effect for each comparison, while the y-axis depicts the standard error of the observed effect size. Potential asymmetry would indicate a form of small-study effects depending on the defined direction, while symmetry in the funnel plot suggests lack of evidence of small-study effects. Egger's tests for funnel plot asymmetry results are also presented.

Comparison-adjusted funnel plots suggest no clear indication of small-study effects in general. However, small-study effects could be suspected of clozapine-olanzapine studies according to the funnel plot for pairwise meta-analysis.

Funnel plot of pairwise meta-analysis for clozapine and olanzapine

Potential missing studies on the right side of the figure would indicate that small studies are likely to provide larger effects in clozapine. The Egger's test shows the asymmetry is significant ($p < 0.05$).

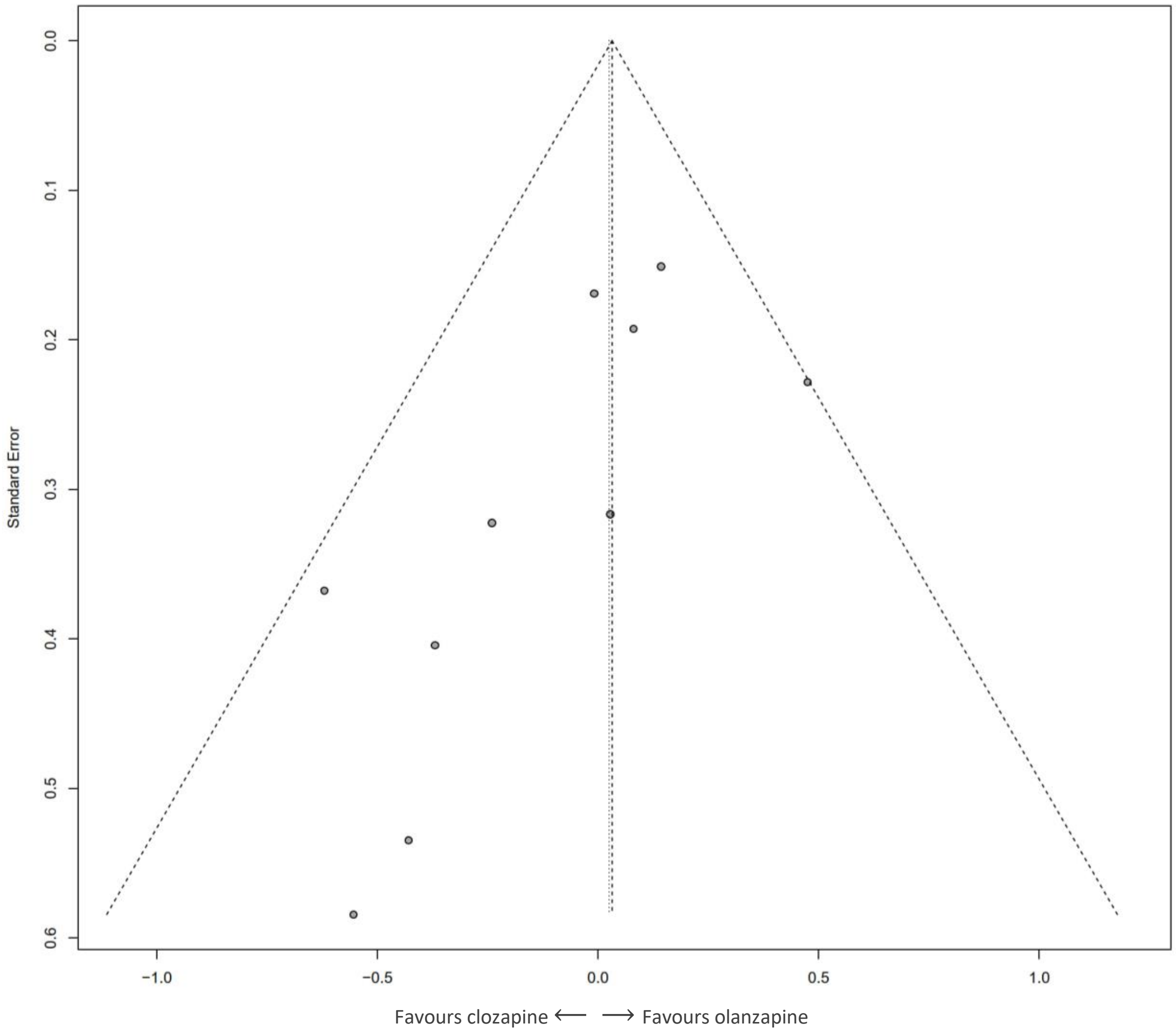

Egger's test for funnel plot asymmetry

t	df	p-value
-2.51	8	0.0364

Comparison-adjusted funnel plot for the primary outcome according to oldness of the treatment

Potential missing studies on the left side of the figure would indicate that small studies are likely to provide larger effects for the newer interventions. However, the Egger's test shows the asymmetry is not significant.

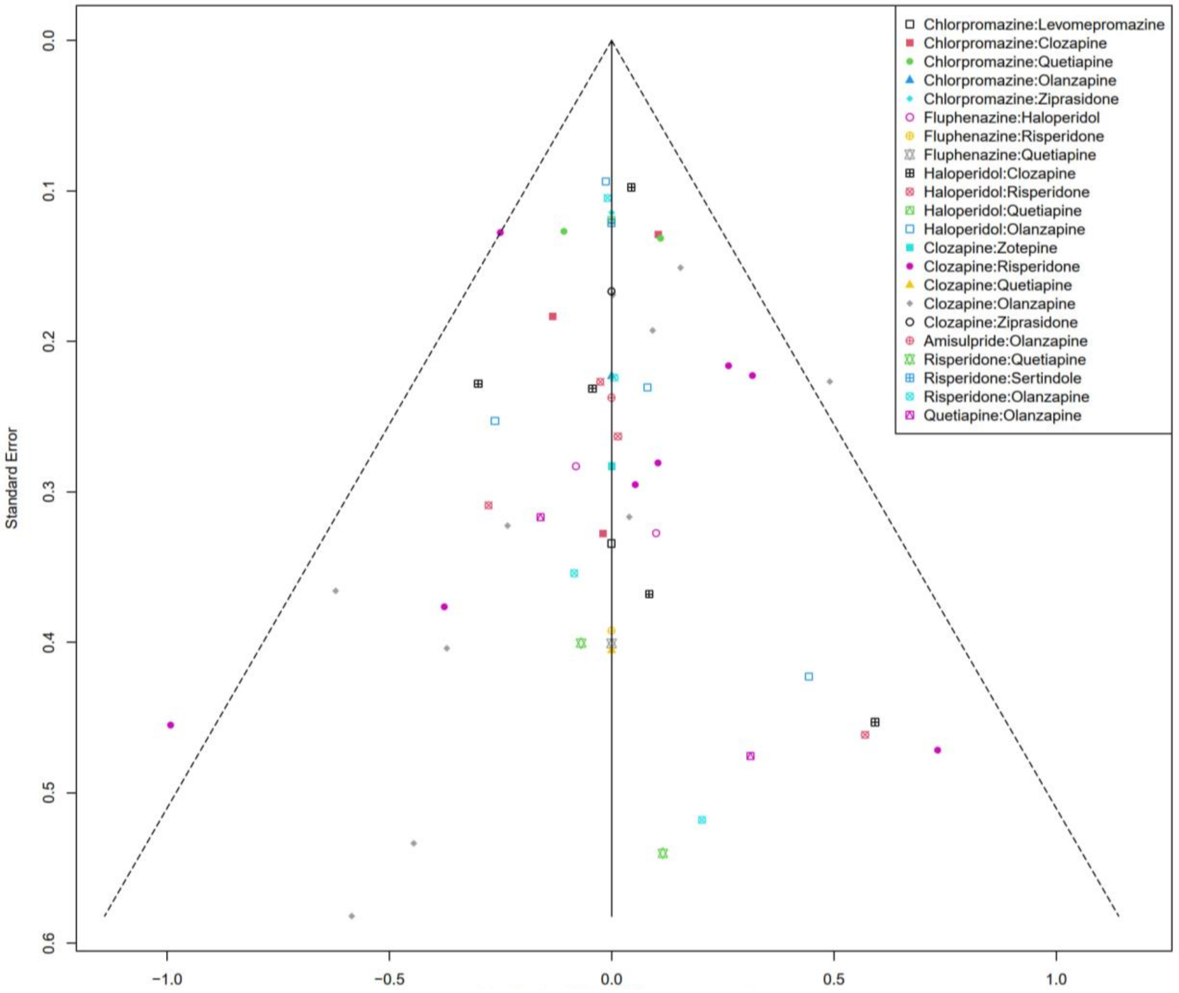

Older treatment more efficacious than comparison average ⟵ ⟶ Newer treatment more efficacious than comparison average

Egger's test for funnel plot asymmetry

t	df	p-value
-0.25	55	0.8049

Comparison-adjusted funnel plot for the primary outcome according to effectiveness of the treatment

Potential missing studies on the right side of the figure would indicate that small studies are likely to provide larger effects for the more efficacious interventions. However, the Egger's test shows the asymmetry is not significant.

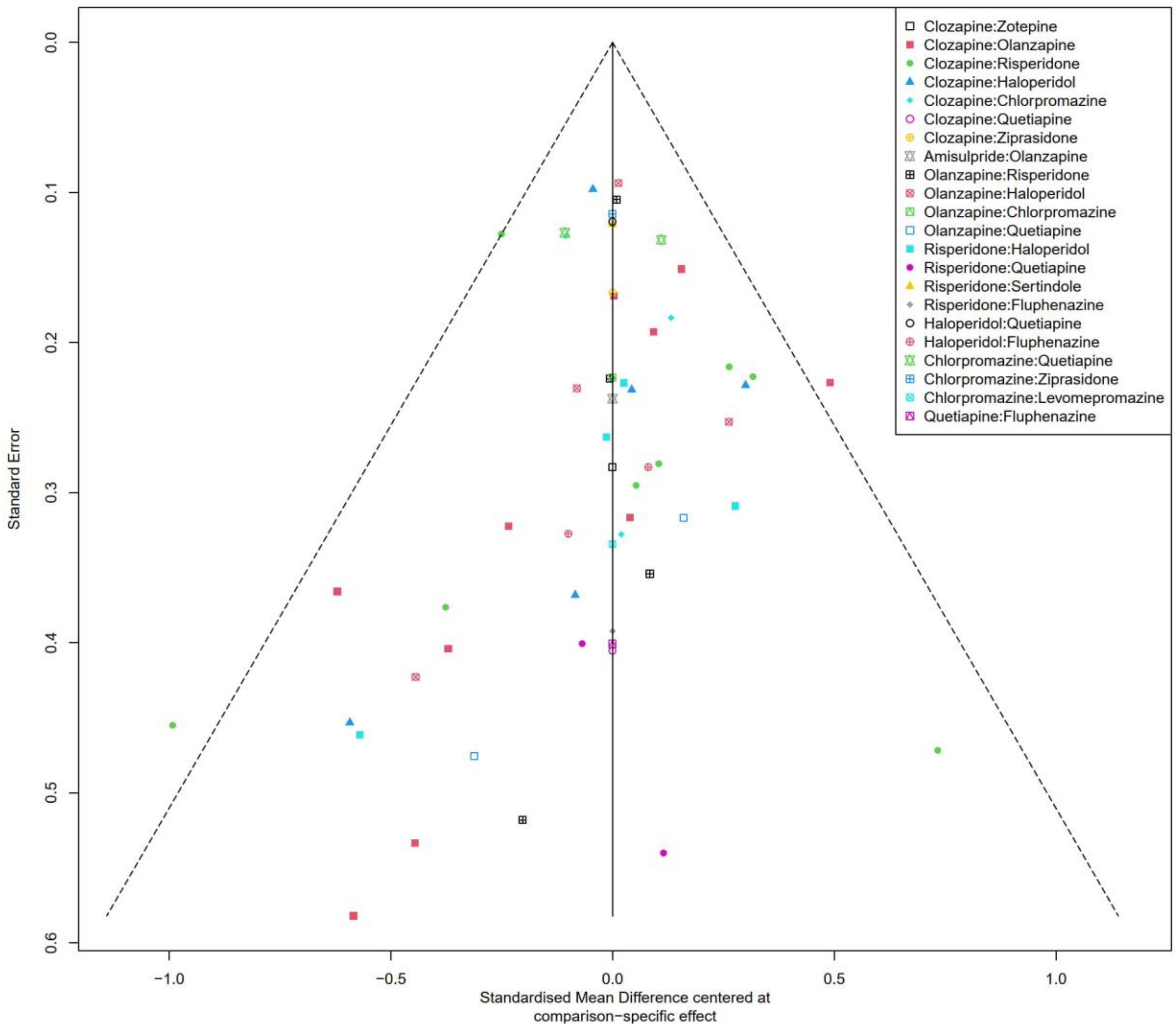

Treatment higher in the total ranking appears more efficacious than comparison average ⟵ ⟶ Treatment lower in the total ranking appears more efficacious than comparison average

Egger's test for funnel plot asymmetry

t	df	p-value
-1.02	55	0.3120

15 Evaluating the confidence in NMA: CINeMA

We evaluated the confidence in evidence of network meta-analytic estimates for the primary outcome with Confidence in Network Meta-Analysis (CINeMA) framework[81] and the official online tool (https://cinema.ispm.unibe.ch/). The CINeMA framework considers six domains that affect the level of confidence in the NMA results: within-study bias, reporting bias, indirectness, imprecision, heterogeneity, and incoherence. These domains are evaluated for each comparison, which subsequently determine an overall confidence in evidence for a comparison. The tool estimates the contribution of each study to the effect size of a comparison using original data and then calculates a contribution matrix. Therefore, some settings and judgements need to be given, which we report in the following.

Domain 1: Within-study bias

We assessed within-study bias using the overall risk of bias rating from the Cochrane's RoB 2.0. Within-study bias for each comparison was classified into "no concerns," "some concerns," and "major concerns" based on the average overall risk of bias according to the contribution matrix.

Domain 2: Reporting bias

We accessed reporting bias using ROB-MEN (Risk of Bias due to Missing Evidence in Network meta-analysis).[83] ROB-MEN, as part of the CINeMA framework, is a web application (https://cinema.ispm.unibe.ch/rob-men/) that simplifies the evaluation of risk of bias due to missing evidence in the estimates from network meta-analysis. The tool has two core parts: the Pairwise Comparisons table and the ROB-MEN table. After the assessment within both tables, a level of "low risk," "some concerns," or "high risk" for the bias due to missing evidence is assigned to each comparison as the final output. Judgements were given based on the recommendations in the guideline[83] and were reported in Appendix 16 ROB-MEN.

Domain 3: Indirectness

For indirectness, it should be judged whether study populations, interventions, outcomes, and study settings are representative of the ones relevant for the research questions. In the cases of the studies included in the present review, the inclusion criteria led to a selection of studies in which these characteristics are directly relevant for the research question, so no indirectness is identified. Details are provided below.

Population: We included only studies in participants with a treatment-resistant form of schizophrenia, schizoaffective disorder, or schizophreniform disorder. Therefore, we consider the study populations as directly relevant for the research question.

Intervention: All investigated interventions were licensed antipsychotics or placebo and thus directly relevant for the research question.

Outcome: Only validated scales, measuring overall symptom of schizophrenia, were used for the primary outcome and are thus relevant for the research question.

Setting: We included studies conducted with out-patients, in-patients, or both. The baseline severity of patients with treatment-resistant schizophrenia is varied. In some studies, patients had symptoms and required rehospitalizations, but in other studies patients were not so symptomatic and were treated as outpatients. Therefore, all these settings can be relevant to the research question.

Domain 4: Imprecision

This domain requires setting thresholds for clinically important differences between the interventions. We considered standardized mean differences (SMDs) outside the range -0.1 to +0.1 as clinically important.

Domain 5: Heterogeneity

We considered standardized mean differences (SMDs) outside the range -0.1 to +0.1 as clinically important. Heterogeneity was evaluated based on the relation of both the 95% confidence intervals and the 95% prediction intervals, with the clinically meaningful threshold defined above in the domain of imprecision according to CINeMA documentation.[82] Comparisons could be classified according to heterogeneity evaluation as "no concerns," "some concerns," and "major concerns."

Domain 6: Incoherence

We considered standardized mean differences (SMDs) outside the range -0.1 to +0.1 as clinically important. Incoherence was evaluated using a design-by-treatment test (for comparisons with only direct or indirect evidence) and the SIDE approach (when both direct and indirect evidence was available) according to CINeMA documentation.[82] Comparisons could be classified according to incoherence evaluation as "no concerns," "some concerns," and "major concerns."

Overall confidence

In CINeMA, judgments for each domain can be summarized in an overall judgment on the confidence in the NMA estimate for each comparison. The level of confidence in the estimate can be classified as "very low," "low," "moderate," "and "high." The meaning of these four levels can be interpreted as follows:

- High quality: Further research is very unlikely to change our confidence in the estimate of effect.

- Moderate quality: Further research is likely to have an important impact on our confidence in the estimate of effect and may change the estimate.

- Low quality: Further research is very likely to have an important impact on our confidence in the estimate of effect and is likely to change the estimate.

- Very low quality: We are very uncertain about the estimate.

The CINeMA-guidance document[82] suggests starting at the first level (i.e., high) for each comparison and to downgrade for one level for a rating of "some concerns," and by two levels for a rating of "major concerns." In the case that several domains are at the level of some concerns or major concerns, it is recommended to consider judgements on different domains jointly rather than in isolation. The reason is that domains are interconnected and downgrading more than once for related concerns should be avoided. The following examples are given in the guidance document: Indirectness includes considerations on intransitivity, which manifests itself in the data as statistical incoherence. Heterogeneity will increase imprecision in treatment effects and may be related to variability in within-study bias or the presence of reporting bias.) Based on these recommendations, we used the following approach that was reported in two previous network meta-analysis studies[84,85] to reach an overall level of confidence for each comparison:

- One judgement of "some concerns" leads to downgrading by one level.

- One judgement of "major concerns" leads to downgrading by two levels.

- Two judgements of "some concerns" could be interconnected and do not justify downgrading more than by one level.

- One judgement of "major concerns" and up to two judgements of "some concerns" or one additional judgement of "major concerns" could be interconnected and do not justify downgrading by more than two levels.

- Two judgements of "major concerns" and any additional judgements of "some concerns" or "major concerns" (or more than four judgements of some concerns) lead to downgrading by three levels.

Confidence in the estimates for the primary outcomes

Comparison	Number of Studies	Within-study bias	Reporting bias	Indirectness	Imprecision	Heterogeneity	Incoherence	Confidence rating	Reason(s) for downgrading
				Mixed evidence					
Amisulpride vs Olanzapine	1	Major concerns	Low risk	No concerns	Major concerns	No concerns	Major concerns	Very low	
Chlorpromazine vs Clozapine	3	Some concerns	Low risk	No concerns	No concerns	No concerns	Major concerns	Low	
Chlorpromazine vs Levomepromazine	1	Major concerns	Low risk	No concerns	Some concerns	Some concerns	Major concerns	Very low	
Chlorpromazine vs Olanzapine	1	Some concerns	Low risk	No concerns	No concerns	Some concerns	No concerns	Moderate	
Chlorpromazine vs Quetiapine	2	Major concerns	Low risk	No concerns	Major concerns	No concerns	Major concerns	Very low	
Chlorpromazine vs Ziprasidone	1	Some concerns	Low risk	No concerns	Some concerns	Some concerns	No concerns	Low	
Clozapine vs Haloperidol	5	Major concerns	Low risk	No concerns	No concerns	Some concerns	No concerns	Low	
Clozapine vs Olanzapine	10	Some concerns	Low risk	No concerns	Major concerns	No concerns	No concerns	Low	
Clozapine vs Quetiapine	1	Some concerns	Low risk	No concerns	No concerns	No concerns	No concerns	Moderate	
Clozapine vs Risperidone	8	Some concerns	Low risk	No concerns	Some concerns	Some concerns	No concerns	Low	
Clozapine vs Ziprasidone	1	Some concerns	Low risk	No concerns	Major concerns	No concerns	No concerns	Low	
Clozapine vs Zotepine	1	Some concerns	Low risk	No concerns	Major concerns	No concerns	Major concerns	Very low	
Fluphenazine vs Haloperidol	2	Major concerns	Low risk	No concerns	Major concerns	No concerns	No concerns	Low	
Fluphenazine vs Quetiapine	1	Major concerns	Low risk	No concerns	Major concerns	No concerns	No concerns	Low	
Fluphenazine vs Risperidone	1	Major concerns	Low risk	No concerns	Major concerns	No concerns	No concerns	Low	
Haloperidol vs Olanzapine	4	Some concerns	Low risk	No concerns	No concerns	Major concerns	No concerns	Low	
Haloperidol vs Quetiapine	1	Some concerns	Low risk	No concerns	Major concerns	No concerns	Some concerns	Low	
Haloperidol vs Risperidone	4	Some concerns	Some concerns	No concerns	Some concerns	Some concerns	No concerns	Low	
Olanzapine vs Quetiapine	2	Some concerns	Low risk	No concerns	No concerns	Some concerns	No concerns	Moderate	
Olanzapine vs Risperidone	4	Some concerns	Low risk	No concerns	Some concerns	Some concerns	No concerns	Low	
Quetiapine vs Risperidone	2	Some concerns	Low risk	No concerns	Some concerns	Some concerns	No concerns	Low	
Risperidone vs Sertindole	1	Some concerns	Low risk	No concerns	Some concerns	Some concerns	Major concerns	Very low	
				Indirect evidence					
Amisulpride vs Chlorpromazine	--	Major concerns	Low risk	No concerns	Some concerns	Some concerns	Major concerns	Very low	
Amisulpride vs Clozapine	--	Major concerns	Low risk	No concerns	Major concerns	No concerns	Major concerns	Very low	
Amisulpride vs Fluphenazine	--	Major concerns	Low risk	No concerns	Major concerns	No concerns	Major concerns	Very low	
Amisulpride vs Haloperidol	--	Major concerns	Low risk	No concerns	Major concerns	No concerns	Major concerns	Very low	
Amisulpride vs Levomepromazine	--	Major concerns	Low risk	No concerns	Major concerns	No concerns	Major concerns	Very low	
Amisulpride vs Quetiapine	--	Major concerns	Low risk	No concerns	Some concerns	Some concerns	Major concerns	Very low	
Amisulpride vs Risperidone	--	Major concerns	Low risk	No concerns	Major concerns	No concerns	Major concerns	Very low	
Amisulpride vs Sertindole	--	Some concerns	Low risk	No concerns	Some concerns	Some concerns	Major concerns	Very low	
Amisulpride vs Ziprasidone	--	Some concerns	Low risk	No concerns	Major concerns	No concerns	Major concerns	Very low	
Amisulpride vs Zotepine	--	Some concerns	Low risk	No concerns	Major concerns	No concerns	Major concerns	Very low	
Chlorpromazine vs Fluphenazine	--	Major concerns	Low risk	No concerns	Major concerns	No concerns	Major concerns	Very low	
Chlorpromazine vs Haloperidol	--	Some concerns	Low risk	No concerns	Major concerns	No concerns	Major concerns	Very low	
Chlorpromazine vs Risperidone	--	Some concerns	Low risk	No concerns	No concerns	Major concerns	Major concerns	Very low	
Chlorpromazine vs Sertindole	--	Some concerns	Low risk	No concerns	Major concerns	No concerns	Major concerns	Very low	
Chlorpromazine vs Zotepine	--	Some concerns	Low risk	No concerns	Major concerns	No concerns	Major concerns	Very low	
Clozapine vs Fluphenazine	--	Major concerns	Low risk	No concerns	Some concerns	Some concerns	Major concerns	Very low	
Clozapine vs Levomepromazine	--	Major concerns	Low risk	No concerns	Major concerns	No concerns	Major concerns	Very low	
Clozapine vs Sertindole	--	Some concerns	Low risk	No concerns	No concerns	Major concerns	Major concerns	Very low	
Fluphenazine vs Levomepromazine	--	Major concerns	Low risk	No concerns	Major concerns	No concerns	Major concerns	Very low	
Fluphenazine vs Olanzapine	--	Some concerns	Low risk	No concerns	Some concerns	Some concerns	Major concerns	Very low	
Fluphenazine vs Sertindole	--	Some concerns	Low risk	No concerns	Major concerns	No concerns	Major concerns	Very low	
Fluphenazine vs Ziprasidone	--	Some concerns	Low risk	No concerns	Major concerns	No concerns	Major concerns	Very low	
Fluphenazine vs Zotepine	--	Some concerns	Low risk	No concerns	Major concerns	No concerns	Major concerns	Very low	
Haloperidol vs Levomepromazine	--	Major concerns	Low risk	No concerns	Major concerns	No concerns	Major concerns	Very low	
Haloperidol vs Sertindole	--	Some concerns	Low risk	No concerns	Major concerns	No concerns	Major concerns	Very low	
Haloperidol vs Ziprasidone	--	Some concerns	Low risk	No concerns	Major concerns	No concerns	Major concerns	Very low	
Haloperidol vs Zotepine	--	Some concerns	Low risk	No concerns	Major concerns	No concerns	Major concerns	Very low	
Levomepromazine vs Olanzapine	--	Major concerns	Low risk	No concerns	Major concerns	No concerns	Major concerns	Very low	
Levomepromazine vs Quetiapine	--	Major concerns	Low risk	No concerns	Some concerns	Some concerns	Major concerns	Very low	

Levomepromazine vs Risperidone	--	Major concerns	Low risk	No concerns	Major concerns	No concerns	Major concerns	Very low	
Levomepromazine vs Sertindole	--	Some concerns	Low risk	No concerns	Major concerns	No concerns	Major concerns	Very low	
Levomepromazine vs Ziprasidone	--	Some concerns	Low risk	No concerns	Major concerns	No concerns	Major concerns	Very low	
Levomepromazine vs Zotepine	--	Some concerns	Low risk	No concerns	Major concerns	No concerns	Major concerns	Very low	
Olanzapine vs Sertindole	--	Some concerns	Low risk	No concerns	Some concerns	Some concerns	Major concerns	Very low	
Olanzapine vs Ziprasidone	--	Some concerns	Low risk	No concerns	Major concerns	No concerns	Major concerns	Very low	
Olanzapine vs Zotepine	--	Some concerns	Low risk	No concerns	Major concerns	No concerns	Major concerns	Very low	
Quetiapine vs Sertindole	--	Some concerns	Low risk	No concerns	Major concerns	No concerns	Major concerns	Very low	
Quetiapine vs Ziprasidone	--	Some concerns	Low risk	No concerns	Major concerns	No concerns	Major concerns	Very low	
Quetiapine vs Zotepine	--	Some concerns	Low risk	No concerns	Major concerns	No concerns	Major concerns	Very low	
Risperidone vs Ziprasidone	--	Some concerns	Low risk	No concerns	Major concerns	No concerns	Major concerns	Very low	
Risperidone vs Zotepine	--	Some concerns	Low risk	No concerns	Major concerns	No concerns	Major concerns	Very low	
Sertindole vs Ziprasidone	--	Some concerns	Low risk	No concerns	Major concerns	No concerns	Major concerns	Very low	
Sertindole vs Zotepine	--	Some concerns	Low risk	No concerns	Major concerns	No concerns	Major concerns	Very low	
Ziprasidone vs Zotepine	--	Some concerns	Low risk	No concerns	Major concerns	No concerns	Major concerns	Very low	

16 ROB-MEN

In the Pairwise Comparisons table, judgements need to be given in two columns: within-study assessment of bias and across-study assessment of bias. Then the overall bias of this table is given automatically. For the ROB-MEN Table, evaluation of contribution from evidence with suspected bias and evaluation of small-study effects require judgements, and overall risk of bias is also automatically given. We report the details in the following.

Within-study assessment of bias

No bias detected was given if no study was suspected of selective non-reporting or under-reporting of results for a specific comparison. Otherwise, we assessed each study for the presence of selective non-reporting of results. Then, we assessed the potential impact of the missing results across all studies using two signaling questions[83] to reach an overall judgement of *no bias detected* or *suspected bias favoring X* for each comparison.

We found three comparisons to be potentially biased because the extra studies did not report the full results and were sponsored by the company manufacturing the drug favored by the bias. For the rest comparisons, the extra studies were either small-studies that were not sufficient to have a notable effect on the overall result or studies that used other primary outcomes instead of overall symptoms.

Across-study assessment of bias

For this situation, it should be evaluated whether there are studies undertaken but not published (publication bias) for each comparison. We assigned *no bias detected* for risk of publication bias to all comparisons because (i) we hypothesized that studies for patients with treatment-resistant schizophrenia are hard to conduct; therefore, there should not be more studies that are not published, especially after the comprehensive search for both published and unpublished studies; (ii) two comparison-adjusted funnel plots and their Egger's test results did not indicate clear evidence for missing small-studies.

Overall bias of Pairwise Comparisons Table

	Pairwise comparison	Number of studies in each comparison		Within-study assessment of bias	Across-study assessment of bias	Overall judgement
		Reporting this outcome (sample size)	Total identified in the SR (total sample size)	set all to "No bias"	set all to "No bias"	calculate overall judgement
**Group A: observed for this outcome**						
1	Amisulpride:Olanzapine	1 (72)	1 (72)	No bias detected	No bias detected	No bias detected
2	Chlorpromazine:Clozapine	3 (430)	3 (430)	No bias detected	No bias detected	No bias detected
3	Chlorpromazine:Levomepromazine	1 (38)	1 (38)	No bias detected	No bias detected	No bias detected
4	Chlorpromazine:Olanzapine	1 (81)	1 (81)	No bias detected	No bias detected	No bias detected
5	Chlorpromazine:Placebo	1 (40)	4 (108)	No bias detected	No bias detected	No bias detected
6	Chlorpromazine:Quetiapine	2 (484)	2 (484)	No bias detected	No bias detected	No bias detected
7	Chlorpromazine:Sulpiride	1 (75)	1 (75)	No bias detected	No bias detected	No bias detected
8	Chlorpromazine:Thioridazine	1 (40)	1 (40)	No bias detected	No bias detected	No bias detected
9	Chlorpromazine:Trifluoperazine	1 (40)	1 (40)	No bias detected	No bias detected	No bias detected
10	Chlorpromazine:Ziprasidone	1 (306)	1 (306)	No bias detected	No bias detected	No bias detected
11	Clozapine:Haloperidol	5 (630)	6 (630)	No bias detected	No bias detected	No bias detected
12	Clozapine:Olanzapine	10 (678)	13 (678)	No bias detected	No bias detected	No bias detected
13	Clozapine:Quetiapine	1 (41)	2 (41)	Suspected bias favouring Clozapine	No bias detected	Suspected bias favouring Clozapine
14	Clozapine:Risperidone	8 (601)	10 (621)	No bias detected	No bias detected	No bias detected

15	Clozapine:Ziprasidone	1 (144)	1 (144)	No bias detected	No bias detected	No bias detected
16	Clozapine:Zotepine	1 (50)	1 (50)	No bias detected	No bias detected	No bias detected
17	Fluphenazine:Haloperidol	2 (97)	2 (97)	No bias detected	No bias detected	No bias detected
18	Fluphenazine:Quetiapine	1 (25)	1 (25)	No bias detected	No bias detected	No bias detected
19	Fluphenazine:Risperidone	1 (26)	1 (26)	No bias detected	No bias detected	No bias detected
20	Haloperidol:Olanzapine	4 (679)	5 (717)	No bias detected	No bias detected	No bias detected
21	Haloperidol:Quetiapine	1 (281)	1 (281)	No bias detected	No bias detected	No bias detected
22	Haloperidol:Risperidone	4 (198)	6 (198)	Suspected bias favouring Risperidone	No bias detected	Suspected bias favouring Risperidone
23	Olanzapine:Quetiapine	2 (58)	3 (83)	No bias detected	No bias detected	No bias detected
24	Olanzapine:Risperidone	4 (496)	6 (770)	No bias detected	No bias detected	No bias detected
25	Placebo:Thioridazine	1 (40)	1 (40)	No bias detected	No bias detected	No bias detected
26	Placebo:Trifluoperazine	1 (40)	2 (90)	No bias detected	No bias detected	No bias detected
27	Quetiapine:Risperidone	2 (39)	3 (61)	No bias detected	No bias detected	No bias detected
28	Risperidone:Sertindole	1 (315)	1 (315)	No bias detected	No bias detected	No bias detected
29	Thioridazine:Trifluoperazine	1 (40)	1 (40)	No bias detected	No bias detected	No bias detected
**Group B: observed for other outcomes**						
30	Chlorpromazine:Haloperidol	NA (NA)	2 (47)	No bias detected	No bias detected	No bias detected
31	Chlorpromazine:Risperidone	NA (NA)	1 (27)	No bias detected	No bias detected	No bias detected
32	Haloperidol:Levomepromazine	NA (NA)	1 (39)	No bias detected	No bias detected	No bias detected
33	Haloperidol:Placebo	NA (NA)	3 (57)	Suspected bias favouring Haloperidol	No bias detected	Suspected bias favouring Haloperidol
**Group C: unobserved**						
34	Amisulpride:Chlorpromazine	NA (NA)	NA (NA)		No bias detected	No bias detected
35	Amisulpride:Clozapine	NA (NA)	NA (NA)		No bias detected	No bias detected
36	Amisulpride:Fluphenazine	NA (NA)	NA (NA)		No bias detected	No bias detected
37	Amisulpride:Haloperidol	NA (NA)	NA (NA)		No bias detected	No bias detected
38	Amisulpride:Levomepromazine	NA (NA)	NA (NA)		No bias detected	No bias detected
39	Amisulpride:Placebo	NA (NA)	NA (NA)		No bias detected	No bias detected
40	Amisulpride:Quetiapine	NA (NA)	NA (NA)		No bias detected	No bias detected
41	Amisulpride:Risperidone	NA (NA)	NA (NA)		No bias detected	No bias detected
42	Amisulpride:Sertindole	NA (NA)	NA (NA)		No bias detected	No bias detected
43	Amisulpride:Sulpiride	NA (NA)	NA (NA)		No bias detected	No bias detected
44	Amisulpride:Thioridazine	NA (NA)	NA (NA)		No bias detected	No bias detected
45	Amisulpride:Trifluoperazine	NA (NA)	NA (NA)		No bias detected	No bias detected
46	Amisulpride:Ziprasidone	NA (NA)	NA (NA)		No bias detected	No bias detected
47	Amisulpride:Zotepine	NA (NA)	NA (NA)		No bias detected	No bias detected
48	Chlorpromazine:Fluphenazine	NA (NA)	NA (NA)		No bias detected	No bias detected
49	Chlorpromazine:Sertindole	NA (NA)	NA (NA)		No bias detected	No bias detected
50	Chlorpromazine:Zotepine	NA (NA)	NA (NA)		No bias detected	No bias detected
51	Clozapine:Fluphenazine	NA (NA)	NA (NA)		No bias detected	No bias detected
52	Clozapine:Levomepromazine	NA (NA)	NA (NA)		No bias detected	No bias detected
53	Clozapine:Placebo	NA (NA)	NA (NA)		No bias detected	No bias detected
54	Clozapine:Sertindole	NA (NA)	NA (NA)		No bias detected	No bias detected
55	Clozapine:Sulpiride	NA (NA)	NA (NA)		No bias detected	No bias detected
56	Clozapine:Thioridazine	NA (NA)	NA (NA)		No bias detected	No bias detected
57	Clozapine:Trifluoperazine	NA (NA)	NA (NA)		No bias detected	No bias detected
58	Fluphenazine:Levomepromazine	NA (NA)	NA (NA)		No bias detected	No bias detected
59	Fluphenazine:Olanzapine	NA (NA)	NA (NA)		No bias detected	No bias detected
60	Fluphenazine:Placebo	NA (NA)	NA (NA)		No bias detected	No bias detected
61	Fluphenazine:Sertindole	NA (NA)	NA (NA)		No bias detected	No bias detected
62	Fluphenazine:Sulpiride	NA (NA)	NA (NA)		No bias detected	No bias detected
63	Fluphenazine:Thioridazine	NA (NA)	NA (NA)		No bias detected	No bias detected

64	Fluphenazine:Trifluoperazine	NA (NA)	NA (NA)	No bias detected	No bias detected
65	Fluphenazine:Ziprasidone	NA (NA)	NA (NA)	No bias detected	No bias detected
66	Fluphenazine:Zotepine	NA (NA)	NA (NA)	No bias detected	No bias detected
67	Haloperidol:Sertindole	NA (NA)	NA (NA)	No bias detected	No bias detected
68	Haloperidol:Sulpiride	NA (NA)	NA (NA)	No bias detected	No bias detected
69	Haloperidol:Thioridazine	NA (NA)	NA (NA)	No bias detected	No bias detected
70	Haloperidol:Trifluoperazine	NA (NA)	NA (NA)	No bias detected	No bias detected
71	Haloperidol:Ziprasidone	NA (NA)	NA (NA)	No bias detected	No bias detected
72	Haloperidol:Zotepine	NA (NA)	NA (NA)	No bias detected	No bias detected
73	Levomepromazine:Olanzapine	NA (NA)	NA (NA)	No bias detected	No bias detected
74	Levomepromazine:Placebo	NA (NA)	NA (NA)	No bias detected	No bias detected
75	Levomepromazine:Quetiapine	NA (NA)	NA (NA)	No bias detected	No bias detected
76	Levomepromazine:Risperidone	NA (NA)	NA (NA)	No bias detected	No bias detected
77	Levomepromazine:Sertindole	NA (NA)	NA (NA)	No bias detected	No bias detected
78	Levomepromazine:Sulpiride	NA (NA)	NA (NA)	No bias detected	No bias detected
79	Levomepromazine:Thioridazine	NA (NA)	NA (NA)	No bias detected	No bias detected
80	Levomepromazine:Trifluoperazine	NA (NA)	NA (NA)	No bias detected	No bias detected
81	Levomepromazine:Ziprasidone	NA (NA)	NA (NA)	No bias detected	No bias detected
82	Levomepromazine:Zotepine	NA (NA)	NA (NA)	No bias detected	No bias detected
83	Olanzapine:Placebo	NA (NA)	NA (NA)	No bias detected	No bias detected
84	Olanzapine:Sertindole	NA (NA)	NA (NA)	No bias detected	No bias detected
85	Olanzapine:Sulpiride	NA (NA)	NA (NA)	No bias detected	No bias detected
86	Olanzapine:Thioridazine	NA (NA)	NA (NA)	No bias detected	No bias detected
87	Olanzapine:Trifluoperazine	NA (NA)	NA (NA)	No bias detected	No bias detected
88	Olanzapine:Ziprasidone	NA (NA)	NA (NA)	No bias detected	No bias detected
89	Olanzapine:Zotepine	NA (NA)	NA (NA)	No bias detected	No bias detected
90	Placebo:Quetiapine	NA (NA)	NA (NA)	No bias detected	No bias detected
91	Placebo:Risperidone	NA (NA)	NA (NA)	No bias detected	No bias detected
92	Placebo:Sertindole	NA (NA)	NA (NA)	No bias detected	No bias detected
93	Placebo:Sulpiride	NA (NA)	NA (NA)	No bias detected	No bias detected
94	Placebo:Ziprasidone	NA (NA)	NA (NA)	No bias detected	No bias detected
95	Placebo:Zotepine	NA (NA)	NA (NA)	No bias detected	No bias detected
96	Quetiapine:Sertindole	NA (NA)	NA (NA)	No bias detected	No bias detected
97	Quetiapine:Sulpiride	NA (NA)	NA (NA)	No bias detected	No bias detected
98	Quetiapine:Thioridazine	NA (NA)	NA (NA)	No bias detected	No bias detected
99	Quetiapine:Trifluoperazine	NA (NA)	NA (NA)	No bias detected	No bias detected
100	Quetiapine:Ziprasidone	NA (NA)	NA (NA)	No bias detected	No bias detected
101	Quetiapine:Zotepine	NA (NA)	NA (NA)	No bias detected	No bias detected
102	Risperidone:Sulpiride	NA (NA)	NA (NA)	No bias detected	No bias detected
103	Risperidone:Thioridazine	NA (NA)	NA (NA)	No bias detected	No bias detected
104	Risperidone:Trifluoperazine	NA (NA)	NA (NA)	No bias detected	No bias detected
105	Risperidone:Ziprasidone	NA (NA)	NA (NA)	No bias detected	No bias detected
106	Risperidone:Zotepine	NA (NA)	NA (NA)	No bias detected	No bias detected
107	Sertindole:Sulpiride	NA (NA)	NA (NA)	No bias detected	No bias detected
108	Sertindole:Thioridazine	NA (NA)	NA (NA)	No bias detected	No bias detected
109	Sertindole:Trifluoperazine	NA (NA)	NA (NA)	No bias detected	No bias detected
110	Sertindole:Ziprasidone	NA (NA)	NA (NA)	No bias detected	No bias detected
111	Sertindole:Zotepine	NA (NA)	NA (NA)	No bias detected	No bias detected
112	Sulpiride:Thioridazine	NA (NA)	NA (NA)	No bias detected	No bias detected
113	Sulpiride:Trifluoperazine	NA (NA)	NA (NA)	No bias detected	No bias detected
114	Sulpiride:Ziprasidone	NA (NA)	NA (NA)	No bias detected	No bias detected

115	Sulpiride:Zotepine	NA (NA)	NA (NA)	No bias detected	No bias detected
116	Thioridazine:Ziprasidone	NA (NA)	NA (NA)	No bias detected	No bias detected
117	Thioridazine:Zotepine	NA (NA)	NA (NA)	No bias detected	No bias detected
118	Trifluoperazine:Ziprasidone	NA (NA)	NA (NA)	No bias detected	No bias detected
119	Trifluoperazine:Zotepine	NA (NA)	NA (NA)	No bias detected	No bias detected
120	Ziprasidone:Zotepine	NA (NA)	NA (NA)	No bias detected	No bias detected

Showing 1 to 120 of 120 entries

Evaluation of contribution from evidence with suspected bias

The possible levels for contribution are *no substantial contribution from bias*, *substantial contribution from bias balanced*, and *substantial contribution from bias favoring X*. We considered the contribution from biased evidence as substantially in favor of one treatment if the relative difference between treatments was at least 15%.

Evaluation of small-study effects

To assess the presence of small-study effects, we compared the obtained adjusted-estimates (column 6 of the ROB-MAN table: NMR treatment effect at the smallest observed variance) with the original estimates (column 5 of the ROB-MAN table: NMA treatment effect) by looking at the overlap of their corresponding confidence intervals. A lack of overlap between the two intervals indicates that effect estimates differ between smaller and larger studies. No small-study effect was identified among all comparisons.

Overall risk of bias

	NMA estimate	% contribution of evidence from pairwise comparisons with suspected bias		Evaluation of contribution from evidence with suspected bias	Bias assessment for indirect evidence	NMA treatment effect	NMR treatment effect at the smallest observed variance	Evaluation of small-study effects	Overall risk of bias
		Favouring first treatment	Favouring second treatment	set all to "No substantial contribution"				set all to "No evidence"	calculate overall RoB
mixed/only direct									
1	Amisulpride:Olanzapine	0	0	No substantial contribution from bias	No bias detected	0.23 (-0.42,0.86)	0.3 (-11.2,12.53)	No evidence of small-study effects	Low risk
2	Chlorpromazine:Clozapine	0	1	No substantial contribution from bias	No bias detected	-0.44 (-0.68,-0.19)	-0.52 (-0.91,-0.13)	No evidence of small-study effects	Low risk
3	Chlorpromazine:Levomepromazine	0	0	No substantial contribution from bias	No bias detected	-0.69 (-1.45,0.07)	2.14 (-30.61,18.33)	No evidence of small-study effects	Low risk
4	Chlorpromazine:Olanzapine	0	0	No substantial contribution from bias	No bias detected	-0.38 (-0.64,-0.1)	-0.5 (-0.93,-0.08)	No evidence of small-study effects	Low risk
5	Chlorpromazine:Placebo	0	0	No substantial contribution from bias	No bias detected	0.52 (-0.18,1.22)	2.75 (-12.97,16.74)	No evidence of small-study effects	Low risk
6	Chlorpromazine:Quetiapine	0	0	No substantial contribution from bias	No bias detected	-0.01 (-0.29,0.26)	0.06 (-0.32,0.44)	No evidence of small-study effects	Low risk
7	Chlorpromazine:Sulpiride	0	0	No substantial contribution from bias	No bias detected	-0.44 (-1.07,0.18)	4.84 (-20.02,20.36)	No evidence of small-study effects	Low risk
8	Chlorpromazine:Thioridazine	0	0	No substantial contribution from bias	No bias detected	-0.09 (-0.79,0.62)	-7.49 (-33.25,6.5)	No evidence of small-study effects	Low risk
9	Chlorpromazine:Trifluoperazine	0	0	No substantial contribution from bias	No bias detected	-0.21 (-0.96,0.53)	-0.56 (-12.07,16.99)	No evidence of small-study effects	Low risk
10	Chlorpromazine:Ziprasidone	0	0	No substantial contribution from bias	No bias detected	-0.24 (-0.62,0.15)	0.14 (-0.63,0.97)	No evidence of small-study effects	Low risk
11	Clozapine:Haloperidol	1	0	No substantial contribution from bias	No bias detected	0.32 (0.12,0.52)	0.3 (0,0.57)	No evidence of small-study effects	Low risk
12	Clozapine:Olanzapine	0	0	No substantial contribution from bias	No bias detected	0.06 (-0.11,0.24)	0.02 (-0.23,0.26)	No evidence of small-study effects	Low risk
13	Clozapine:Quetiapine	6	0	No substantial contribution from bias	Suspected bias favouring Clozapine	0.42 (0.14,0.71)	0.58 (0.17,0.98)	No evidence of small-study effects	Low risk
14	Clozapine:Risperidone	0	8	No substantial contribution from bias	No bias detected	0.19 (0,0.38)	0.27 (-0.01,0.55)	No evidence of small-study effects	Low risk
15	Clozapine:Ziprasidone	0	0	No substantial contribution from bias	No bias detected	0.2 (-0.2,0.59)	0.67 (-0.18,1.53)	No evidence of small-study effects	Low risk
16	Clozapine:Zotepine	0	0	No substantial contribution from bias	No bias detected	0.07 (-0.63,0.76)	19.23 (2.56,38.72)	No evidence of small-study effects	Low risk

17	Fluphenazine:Haloperidol	0	0	No substantial contribution from bias	No bias detected	-0.1 (-0.52,0.32)	-0.3 (-1.2,0.48)	No evidence of small-study effects	Low risk
18	Fluphenazine:Quetiapine	0	0	No substantial contribution from bias	No bias detected	0.01 (-0.48,0.49)	-0.01 (-0.98,0.85)	No evidence of small-study effects	Low risk
19	Fluphenazine:Risperidone	0	12	No substantial contribution from bias	No bias detected	-0.22 (-0.67,0.23)	-0.32 (-1.3,0.53)	No evidence of small-study effects	Low risk
20	Haloperidol:Olanzapine	0	0	No substantial contribution from bias	No bias detected	-0.26 (-0.46,-0.05)	-0.28 (-0.57,0.01)	No evidence of small-study effects	Low risk
21	Haloperidol:Quetiapine	0	0	No substantial contribution from bias	No bias detected	0.11 (-0.2,0.4)	0.29 (-0.12,0.69)	No evidence of small-study effects	Low risk
22	Haloperidol:Risperidone	0	33	Substantial contribution from bias favouring Risperidor	Suspected bias favouring Risperidone	-0.13 (-0.36,0.1)	-0.03 (-0.37,0.33)	No evidence of small-study effects	Some concerns
23	Olanzapine:Quetiapine	0	0	No substantial contribution from bias	No bias detected	0.37 (0.06,0.65)	0.56 (0.13,0.99)	No evidence of small-study effects	Low risk
24	Olanzapine:Risperidone	0	8	No substantial contribution from bias	No bias detected	0.13 (-0.08,0.33)	0.25 (-0.05,0.56)	No evidence of small-study effects	Low risk
25	Placebo:Thioridazine	0	0	No substantial contribution from bias	No bias detected	-0.61 (-1.35,0.15)	-10.53 (-35.1,3.13)	No evidence of small-study effects	Low risk
26	Placebo:Trifluoperazine	0	0	No substantial contribution from bias	No bias detected	-0.73 (-1.51,0.07)	-1.89 (-19.76,14.98)	No evidence of small-study effects	Low risk
27	Quetiapine:Risperidone	0	9	No substantial contribution from bias	No bias detected	-0.23 (-0.54,0.09)	-0.31 (-0.77,0.16)	No evidence of small-study effects	Low risk
28	Risperidone:Sertindole	0	0	No substantial contribution from bias	No bias detected	0.32 (-0.18,0.82)	-0.11 (-13.58,16.37)	No evidence of small-study effects	Low risk
29	Thioridazine:Trifluoperazine	0	0	No substantial contribution from bias	No bias detected	-0.12 (-0.89,0.65)	9.3 (-10.26,43.24)	No evidence of small-study effects	Low risk
**indirect**									
30	Chlorpromazine:Haloperidol	0	0	No substantial contribution from bias	No bias detected	-0.12 (-0.4,0.18)	-0.22 (-0.66,0.19)	No evidence of small-study effects	Low risk
31	Chlorpromazine:Risperidone	0	5	No substantial contribution from bias	No bias detected	-0.25 (-0.54,0.05)	-0.25 (-0.72,0.21)	No evidence of small-study effects	Low risk
32	Haloperidol:Levomepromazine	0	0	No substantial contribution from bias	No bias detected	-0.57 (-1.39,0.25)	2.34 (-30.34,18.53)	No evidence of small-study effects	Low risk
33	Haloperidol:Placebo	0	0	No substantial contribution from bias	Suspected bias favouring Haloperidol	0.64 (-0.14,1.4)	3.04 (-12.76,16.94)	No evidence of small-study effects	Low risk
34	Amisulpride:Chlorpromazine	0	0	No substantial contribution from bias	No bias detected	0.61 (-0.11,1.3)	0.8 (-10.78,13.15)	No evidence of small-study effects	Low risk
35	Amisulpride:Clozapine	0	0	No substantial contribution from bias	No bias detected	0.17 (-0.51,0.83)	0.28 (-11.21,12.52)	No evidence of small-study effects	Low risk
36	Amisulpride:Fluphenazine	0	0	No substantial contribution from bias	No bias detected	0.59 (-0.22,1.36)	0.87 (-10.6,13.16)	No evidence of small-study effects	Low risk
37	Amisulpride:Haloperidol	0	0	No substantial contribution from bias	No bias detected	0.49 (-0.2,1.16)	0.59 (-10.93,12.81)	No evidence of small-study effects	Low risk
38	Amisulpride:Levomepromazine	0	0	No substantial contribution from bias	No bias detected	-0.09 (-1.12,0.97)	0.12 (-17.17,20.63)	No evidence of small-study effects	Low risk
39	Amisulpride:Placebo	0	0	No substantial contribution from bias	No bias detected	1.13 (0.13,2.13)	2.17 (-17.17,21.78)	No evidence of small-study effects	Low risk
40	Amisulpride:Quetiapine	0	0	No substantial contribution from bias	No bias detected	0.6 (-0.13,1.3)	0.86 (-10.66,13.21)	No evidence of small-study effects	Low risk
41	Amisulpride:Risperidone	0	6	No substantial contribution from bias	No bias detected	0.36 (-0.33,1.04)	0.53 (-10.96,12.78)	No evidence of small-study effects	Low risk
42	Amisulpride:Sertindole	0	0	No substantial contribution from bias	No bias detected	0.68 (-0.18,1.52)	-1.92 (-16.62,29.98)	No evidence of small-study effects	Low risk
43	Amisulpride:Sulpiride	0	0	No substantial contribution from bias	No bias detected	0.17 (-0.81,1.08)	8.34 (-27.19,22.88)	No evidence of small-study effects	Low risk
44	Amisulpride:Thioridazine	0	0	No substantial contribution from bias	No bias detected	0.52 (-0.48,1.49)	-10.29 (-32.01,14.26)	No evidence of small-study effects	Low risk
45	Amisulpride:Trifluoperazine	0	0	No substantial contribution from bias	No bias detected	0.4 (-0.63,1.41)	-1.69 (-17.79,28.55)	No evidence of small-study effects	Low risk
46	Amisulpride:Ziprasidone	0	0	No substantial contribution from bias	No bias detected	0.37 (-0.42,1.14)	0.92 (-10.64,13.24)	No evidence of small-study effects	Low risk
47	Amisulpride:Zotepine	0	0	No substantial contribution from bias	No bias detected	0.24 (-0.74,1.18)	16.09 (-6.22,50.2)	No evidence of small-study effects	Low risk
48	Chlorpromazine:Fluphenazine	0	0	No substantial contribution from bias	No bias detected	-0.02 (-0.51,0.47)	0.07 (-0.8,1.06)	No evidence of small-study effects	Low risk
49	Chlorpromazine:Sertindole	0	0	No substantial contribution from bias	No bias detected	0.07 (-0.5,0.66)	-0.33 (-13.85,16.16)	No evidence of small-study effects	Low risk
50	Chlorpromazine:Zotepine	0	0	No substantial contribution from bias	No bias detected	-0.37 (-1.1,0.37)	18.7 (2.02,38.31)	No evidence of small-study effects	Low risk
51	Clozapine:Fluphenazine	1	0	No substantial contribution from bias	No bias detected	0.41 (-0.03,0.86)	0.59 (-0.24,1.55)	No evidence of small-study effects	Low risk
52	Clozapine:Levomepromazine	1	0	No substantial contribution from bias	No bias detected	-0.25 (-1.04,0.54)	2.59 (-30.05,18.85)	No evidence of small-study effects	Low risk
53	Clozapine:Placebo	1	0	No substantial contribution from bias	No bias detected	0.96 (0.2,1.7)	3.32 (-12.44,17.2)	No evidence of small-study effects	Low risk
54	Clozapine:Sertindole	0	0	No substantial contribution from bias	No bias detected	0.51 (-0.02,1.05)	0.3 (-13.31,16.66)	No evidence of small-study effects	Low risk
55	Clozapine:Sulpiride	1	0	No substantial contribution from bias	No bias detected	0 (-0.68,0.66)	5.35 (-19.55,20.88)	No evidence of small-study effects	Low risk
56	Clozapine:Thioridazine	1	0	No substantial contribution from bias	No bias detected	0.35 (-0.4,1.09)	-6.98 (-32.74,6.98)	No evidence of small-study effects	Low risk
57	Clozapine:Trifluoperazine	1	0	No substantial contribution from bias	No bias detected	0.23 (-0.56,1)	-0.04 (-11.59,17.5)	No evidence of small-study effects	Low risk
58	Fluphenazine:Levomepromazine	0	0	No substantial contribution from bias	No bias detected	-0.66 (-1.58,0.24)	2.11 (-30.78,18.3)	No evidence of small-study effects	Low risk
59	Fluphenazine:Olanzapine	0	0	No substantial contribution from bias	No bias detected	-0.36 (-0.8,0.1)	-0.58 (-1.52,0.23)	No evidence of small-study effects	Low risk
60	Fluphenazine:Placebo	0	0	No substantial contribution from bias	No bias detected	0.54 (-0.32,1.39)	2.8 (-13.06,16.64)	No evidence of small-study effects	Low risk
61	Fluphenazine:Sertindole	0	0	No substantial contribution from bias	No bias detected	0.09 (-0.57,0.78)	-0.93 (-14.02,16.02)	No evidence of small-study effects	Low risk
62	Fluphenazine:Sulpiride	0	0	No substantial contribution from bias	No bias detected	-0.42 (-1.22,0.36)	4.73 (-20.04,20.11)	No evidence of small-study effects	Low risk
63	Fluphenazine:Thioridazine	0	0	No substantial contribution from bias	No bias detected	-0.07 (-0.92,0.81)	-7.68 (-33.21,6.42)	No evidence of small-study effects	Low risk
64	Fluphenazine:Trifluoperazine	0	0	No substantial contribution from bias	No bias detected	-0.17 (-1.09,0.69)	-0.71 (-12.18,16.87)	No evidence of small-study effects	Low risk
65	Fluphenazine:Ziprasidone	0	0	No substantial contribution from bias	No bias detected	-0.21 (-0.81,0.37)	0.07 (-1.13,1.2)	No evidence of small-study effects	Low risk
66	Fluphenazine:Zotepine	0	0	No substantial contribution from bias	No bias detected	-0.35 (-1.16,0.47)	18.7 (1.93,38.13)	No evidence of small-study effects	Low risk
67	Haloperidol:Sertindole	0	0	No substantial contribution from bias	No bias detected	0.19 (-0.35,0.74)	-0.11 (-13.58,16.37)	No evidence of small-study effects	Low risk
68	Haloperidol:Sulpiride	0	0	No substantial contribution from bias	No bias detected	-0.32 (-1.02,0.36)	5.07 (-19.88,20.55)	No evidence of small-study effects	Low risk
69	Haloperidol:Thioridazine	0	0	No substantial contribution from bias	No bias detected	0.03 (-0.74,0.79)	-7.27 (-33.01,6.69)	No evidence of small-study effects	Low risk
70	Haloperidol:Trifluoperazine	0	0	No substantial contribution from bias	No bias detected	-0.08 (-0.89,0.7)	-0.33 (-11.87,17.17)	No evidence of small-study effects	Low risk
71	Haloperidol:Ziprasidone	0	0	No substantial contribution from bias	No bias detected	-0.11 (-0.56,0.3)	0.37 (-0.47,1.25)	No evidence of small-study effects	Low risk
72	Haloperidol:Zotepine	0	0	No substantial contribution from bias	No bias detected	-0.25 (-0.97,0.48)	18.9 (2.27,38.42)	No evidence of small-study effects	Low risk

73	Levomepromazine:Olanzapine	0	0	No substantial contribution from bias	No bias detected	0.31 (-0.5,1.12)	-2.63 (-18.83,30.07)	No evidence of small-study effects	Low risk
74	Levomepromazine:Placebo	0	0	No substantial contribution from bias	No bias detected	1.21 (0.18,2.24)	3.11 (-29.49,38.91)	No evidence of small-study effects	Low risk
75	Levomepromazine:Quetiapine	0	0	No substantial contribution from bias	No bias detected	0.67 (-0.14,1.49)	-2.02 (-18.23,30.62)	No evidence of small-study effects	Low risk
76	Levomepromazine:Risperidone	0	4	No substantial contribution from bias	No bias detected	0.44 (-0.38,1.26)	-2.33 (-18.52,30.33)	No evidence of small-study effects	Low risk
77	Levomepromazine:Sertindole	0	0	No substantial contribution from bias	No bias detected	0.76 (-0.19,1.71)	-1.48 (-27.81,47.5)	No evidence of small-study effects	Low risk
78	Levomepromazine:Sulpiride	0	0	No substantial contribution from bias	No bias detected	0.26 (-0.75,1.22)	2.49 (-28.06,35.97)	No evidence of small-study effects	Low risk
79	Levomepromazine:Thioridazine	0	0	No substantial contribution from bias	No bias detected	0.6 (-0.43,1.63)	-11.66 (-44.06,31.09)	No evidence of small-study effects	Low risk
80	Levomepromazine:Trifluoperazine	0	0	No substantial contribution from bias	No bias detected	0.49 (-0.61,1.54)	-2.32 (-29.18,46.82)	No evidence of small-study effects	Low risk
81	Levomepromazine:Ziprasidone	0	0	No substantial contribution from bias	No bias detected	0.45 (-0.4,1.29)	-1.82 (-18.2,30.58)	No evidence of small-study effects	Low risk
82	Levomepromazine:Zotepine	0	0	No substantial contribution from bias	No bias detected	0.32 (-0.74,1.36)	15.02 (-0.76,68.05)	No evidence of small-study effects	Low risk
83	Olanzapine:Placebo	0	0	No substantial contribution from bias	No bias detected	0.9 (0.13,1.65)	3.3 (-12.47,17.17)	No evidence of small-study effects	Low risk
84	Olanzapine:Sertindole	0	0	No substantial contribution from bias	No bias detected	0.45 (-0.1,0.98)	0.27 (-13.31,16.64)	No evidence of small-study effects	Low risk
85	Olanzapine:Sulpiride	0	0	No substantial contribution from bias	No bias detected	-0.06 (-0.76,0.6)	5.34 (-19.58,20.82)	No evidence of small-study effects	Low risk
86	Olanzapine:Thioridazine	0	0	No substantial contribution from bias	No bias detected	0.29 (-0.47,1.04)	-7 (-32.74,6.96)	No evidence of small-study effects	Low risk
87	Olanzapine:Trifluoperazine	0	0	No substantial contribution from bias	No bias detected	0.17 (-0.63,0.95)	-0.06 (-11.61,17.46)	No evidence of small-study effects	Low risk
88	Olanzapine:Ziprasidone	0	0	No substantial contribution from bias	No bias detected	0.14 (-0.29,0.55)	0.65 (-0.21,1.52)	No evidence of small-study effects	Low risk
89	Olanzapine:Zotepine	0	0	No substantial contribution from bias	No bias detected	0.01 (-0.71,0.71)	19.2 (2.55,38.66)	No evidence of small-study effects	Low risk
90	Placebo:Quetiapine	0	0	No substantial contribution from bias	No bias detected	-0.54 (-1.29,0.22)	-2.66 (-16.66,13.01)	No evidence of small-study effects	Low risk
91	Placebo:Risperidone	0	3	No substantial contribution from bias	No bias detected	-0.77 (-1.53,0.01)	-3.07 (-16.93,12.68)	No evidence of small-study effects	Low risk
92	Placebo:Sertindole	0	0	No substantial contribution from bias	No bias detected	-0.45 (-1.35,0.48)	-3.11 (-11.77,9.66)	No evidence of small-study effects	Low risk
93	Placebo:Sulpiride	0	0	No substantial contribution from bias	No bias detected	-0.96 (-1.89,-0.02)	3.06 (-33.35,28.42)	No evidence of small-study effects	Low risk
94	Placebo:Ziprasidone	0	0	No substantial contribution from bias	No bias detected	-0.75 (-1.56,0.03)	-2.47 (-16.59,13.15)	No evidence of small-study effects	Low risk
95	Placebo:Zotepine	0	0	No substantial contribution from bias	No bias detected	-0.88 (-1.91,0.13)	16.29 (-4.89,42.48)	No evidence of small-study effects	Low risk
96	Quetiapine:Sertindole	0	0	No substantial contribution from bias	No bias detected	0.08 (-0.5,0.68)	-0.44 (-13.91,16.13)	No evidence of small-study effects	Low risk
97	Quetiapine:Sulpiride	0	0	No substantial contribution from bias	No bias detected	-0.42 (-1.12,0.24)	4.79 (-20.11,20.23)	No evidence of small-study effects	Low risk
98	Quetiapine:Thioridazine	0	0	No substantial contribution from bias	No bias detected	-0.07 (-0.82,0.68)	-7.55 (-33.35,6.44)	No evidence of small-study effects	Low risk
99	Quetiapine:Trifluoperazine	0	0	No substantial contribution from bias	No bias detected	-0.19 (-0.99,0.59)	-0.64 (-12.15,16.91)	No evidence of small-study effects	Low risk
100	Quetiapine:Ziprasidone	0	0	No substantial contribution from bias	No bias detected	-0.22 (-0.67,0.22)	0.08 (-0.75,0.93)	No evidence of small-study effects	Low risk
101	Quetiapine:Zotepine	0	0	No substantial contribution from bias	No bias detected	-0.36 (-1.11,0.4)	18.63 (1.96,38.21)	No evidence of small-study effects	Low risk
102	Risperidone:Sulpiride	4	0	No substantial contribution from bias	No bias detected	-0.19 (-0.88,0.48)	5.12 (-19.85,20.55)	No evidence of small-study effects	Low risk
103	Risperidone:Thioridazine	3	0	No substantial contribution from bias	No bias detected	0.16 (-0.61,0.93)	-7.26 (-33.02,6.66)	No evidence of small-study effects	Low risk
104	Risperidone:Trifluoperazine	3	0	No substantial contribution from bias	No bias detected	0.04 (-0.76,0.83)	-0.33 (-11.85,17.24)	No evidence of small-study effects	Low risk
105	Risperidone:Ziprasidone	4	0	No substantial contribution from bias	No bias detected	0.01 (-0.42,0.44)	0.4 (-0.47,1.28)	No evidence of small-study effects	Low risk
106	Risperidone:Zotepine	5	0	No substantial contribution from bias	No bias detected	-0.12 (-0.84,0.6)	18.94 (2.28,38.44)	No evidence of small-study effects	Low risk
107	Sertindole:Sulpiride	0	0	No substantial contribution from bias	No bias detected	-0.51 (-1.37,0.34)	2.42 (-27.3,28.68)	No evidence of small-study effects	Low risk
108	Sertindole:Thioridazine	0	0	No substantial contribution from bias	No bias detected	-0.16 (-1.07,0.74)	-10.47 (-27.35,6.25)	No evidence of small-study effects	Low risk
109	Sertindole:Trifluoperazine	0	0	No substantial contribution from bias	No bias detected	-0.27 (-1.23,0.65)	-2.42 (-17.33,20.82)	No evidence of small-study effects	Low risk
110	Sertindole:Ziprasidone	0	0	No substantial contribution from bias	No bias detected	-0.31 (-0.97,0.34)	0.44 (-16.1,14.03)	No evidence of small-study effects	Low risk
111	Sertindole:Zotepine	0	0	No substantial contribution from bias	No bias detected	-0.44 (-1.31,0.44)	16.69 (0.81,44.14)	No evidence of small-study effects	Low risk
112	Sulpiride:Thioridazine	0	0	No substantial contribution from bias	No bias detected	0.35 (-0.59,1.28)	-4.08 (-51.87,16.98)	No evidence of small-study effects	Low risk
113	Sulpiride:Trifluoperazine	0	0	No substantial contribution from bias	No bias detected	0.23 (-0.73,1.21)	-3.22 (-27.25,21.37)	No evidence of small-study effects	Low risk
114	Sulpiride:Ziprasidone	0	0	No substantial contribution from bias	No bias detected	0.2 (-0.52,0.95)	-4.77 (-20.06,20.08)	No evidence of small-study effects	Low risk
115	Sulpiride:Zotepine	0	0	No substantial contribution from bias	No bias detected	0.07 (-0.88,1.03)	17.35 (-4.05,38.2)	No evidence of small-study effects	Low risk
116	Thioridazine:Ziprasidone	0	0	No substantial contribution from bias	No bias detected	-0.15 (-0.95,0.65)	7.59 (-6.32,33.5)	No evidence of small-study effects	Low risk
117	Thioridazine:Zotepine	0	0	No substantial contribution from bias	No bias detected	-0.28 (-1.29,0.73)	23.59 (0.77,69.11)	No evidence of small-study effects	Low risk
118	Trifluoperazine:Ziprasidone	0	0	No substantial contribution from bias	No bias detected	-0.03 (-0.84,0.81)	0.7 (-16.84,12.34)	No evidence of small-study effects	Low risk
119	Trifluoperazine:Zotepine	0	0	No substantial contribution from bias	No bias detected	-0.16 (-1.2,0.88)	19.45 (-6,43.77)	No evidence of small-study effects	Low risk
120	Ziprasidone:Zotepine	0	0	No substantial contribution from bias	No bias detected	-0.13 (-0.92,0.66)	18.43 (1.83,38.15)	No evidence of small-study effects	Low risk

Showing 1 to 120 of 120 entries

17 References

1 Lefebvre C, Glanville J, Briscoe S, et al. Searching for and selecting studies, 2019.

2 Shokraneh F, Adams CE. Study-based registers reduce waste in systematic reviewing: discussion and case report. *Syst Rev* 2019; **8:** 129. https://doi.org/10.1186/s13643-019-1035-3.

3 Shokraneh F, Adams CE. Study-based registers of randomized controlled trials: Starting a systematic review with data extraction or meta-analysis. *Bioimpacts* 2017; **7:** 209–17. https://doi.org/10.15171/bi.2017.25.

4 Shokraneh F, Adams CE. Cochrane Schizophrenia Group's Study-Based Register of Randomized Controlled Trials: Development and Content Analysis. *Schizophr Bull Open* 2020; **1:** sgaa061. https://doi.org/10.1093/schizbullopen/sgaa061.

5 Shokraneh F, Adams CE. Classification of all pharmacological interventions tested in trials relevant to people with schizophrenia: A study-based analysis. *Health Info Libr J* 2021. https://doi.org/10.1111/hir.12366.

6 Schlosberg A, Shadmi M. A comparative controlled study of two long-acting phenothiazines. pipotiazine palmitate and fluphenazine decanoate. *Current Therapeutic Research, Clinical and Experimental* 1978; **23:** 642–54.

7 Teja JS, Grey WH, Clums JM, Warren C. Tranquilizers or anti-depressants for chronic schizophrenics: a long term study. *Aust N Z J Psychiatry* 1975; **9:** 241–47.

8 Bozzatello P, Bellino S, Mancini I, Sandei L, Zanalda E, Rocca P. Effects on Satisfaction and Service Engagement of Paliperidone Palmitate Compared with Oral Paliperidone in Patients with Schizophrenia. An Open Label Randomized Controlled Trial. *Clinical drug investigation* 2019; **39:** 169–78.

9 Ahlfors UG, Dencker SJ, Gravem A, Remvig J. Clopenthixol decanoate and perphenazine enanthate in schizophrenic patients. A double-blind Nordic multicentre trial. *Acta Psychiatrica Scandinavica Supplementum* 1980; **279:** 77–91.

10 Altamura AC, Velona I, Curreli R, Mundo E, Bravi D. Is olanzapine better than haloperidol in resistant schizophrenia? A double-blind study in partial responders. *International Journal of Psychiatry in Clinical Practice* 2002; **6:** 107–11.

11 Alvarez E, Ciudad A, Olivares JM, Bousono M, Gomez JC. A randomized, 1-year follow-up study of olanzapine and risperidone in the treatment of negative symptoms in outpatients with schizophrenia. *Journal of Clinical Psychopharmacology* 2006; **26:** 238–49.

12 AstraZeneca. A multicenter, double-blind, randomized, comparison of quetiapine (seroquel) and chlorpromazine in the treatment of subjects with treatment-resistant schizophrenia. *http://www.Clinicalstudyresults.org/* 2005.

13 AstraZeneca. A multicenter, double-blind, randomized, comparison of quetiapine (seroquel) and chlorpromazine in the treatment of subjects with treatment-resistant schizophrenia. *http://www.Clinicalstudyresults.org/* 2005.

14 Azorin JM, Spiegel R, Remington G, et al. A double-blind comparative study of clozapine and risperidone in the management of severe chronic schizophrenia. *The American Journal of Psychiatry* 2001; **158:** 1305–13.

15 Bitter I, Dossenbach MRK, Brook S, et al. Olanzapine versus clozapine in treatment-resistant or treatment-intolerant schizophrenia. *Progress in Neuro-Psychopharmacology and Biological Psychiatry* 2004; **28:** 173–80.

16 Bondolfi G, Dufour H, Patris M, et al. Risperidone versus clozapine in treatment-resistant chronic schizophrenia. a randomized double-blind study. *American Journal of Psychiatry* 1998; **155:** 499–504.

17 Breier AF, Malhotra AK, Su TP, et al. Clozapine and risperidone in chronic schizophrenia. effects on symptoms, parkinsonian side effects, and neuroendocrine response. *American Journal of Psychiatry* 1999; **156:** 294–98.
18 Breier A, Hamilton SH, David S. Comparative efficacy of olanzapine and haloperidol for patients with treatment resistant schizophrenia. *Schizophrenia Research* 1999; **36:** 271.
19 Browne FW, Cooper SJ, Wilson R, King DJ. Serum haloperidol levels and clinical response in chronic, treatment resistant schizophrenic patients. *Journal of Psychopharmacology* 1988; **2:** 94–103.
20 Buchanan RW, Breier A, Kirkpatrick B, Ball P, Carpenter, W. T., Jr. Positive and negative symptom response to clozapine in schizophrenic patients with and without the deficit syndrome. *American Journal of Psychiatry* 1998; **155:** 751–60.
21 Buchanan RW, Ball MP, Weiner E, et al. Olanzapine treatment of residual positive and negative symptoms. *American Journal of Psychiatry* 2005; **162:** 124–29.
22 Chen JJ, Chan HY, Chen CH, Gau SSF, Hwu HG. Risperidone and olanzapine versus another first generation antipsychotic in patients with schizophrenia inadequately responsive to first generation antipsychotics. *PharmacoPsychiatry* 2012; **45:** 64–71.
23 Chowdhury AN, Mukherjee A, Ghosh K, Chowdhury S, Das SK. Horizon of a new hope. recovery of schizophrenia in India. *International Medical Journal* 1999; **6:** 181–85.
24 Claus A, Bollen J, De CH, et al. Risperidone versus haloperidol in the treatment of chronic schizophrenic inpatients. a multicentre double-blind comparative study. *Acta Psychiatrica Scandinavica* 1992; **85:** 295–305.
25 Conley RR, Tamminga CA, Bartko JJ, et al. Olanzapine compared with chlorpromazine in treatment-resistant schizophrenia. *American Journal of Psychiatry* 1998; **155:** 914–20.
26 Conley RR, Kelly DL, Richardson CM, Tamminga CA, Carpenter, W. T., Jr. The efficacy of high-dose olanzapine versus clozapine in treatment-resistant schizophrenia. a double-blind, crossover study. *Journal of Clinical Psychopharmacology* 2003; **23:** 668–71.
27 Conley RR, Kelly DL, Nelson MW, et al. Risperidone, quetiapine, and fluphenazine in the treatment of patients with therapy-refractory schizophrenia. *Clinical Neuropharmacology* 2005; **28:** 163–68.
28 Daniel DG, Goldberg TE, Weinberger DR, et al. Different side effect profiles of risperidone and clozapine in 20 outpatients with schizophrenia or schizoaffective disorder. a pilot study. *American Journal of Psychiatry* 1996; **153:** 417–19.
29 Dean EF, Buker S. Schizophrenia treated with and without chlorpromazine. *Rocky Mountain Medical Journal* 1958; **55:** 47–50.
30 Emsley RA, Raniwalla J, Bailey PJ, Jones-Am. A comparison of the effects of quetiapine ('seroquel') and haloperidol in schizophrenic patients with a history of and a demonstrated, partial response to conventional antipsychotic treatment. *International Clinical Psychopharmacology* 2000; **15:** 121–31.
31 Hall WB, Vestre ND, Schiele BC, Zimmermann R. A controlled comparison of haloperidol and fluphenazine in chronic treatment-resistant schizophrenics. *Diseases of the Nervous System* 1968; **29:** 405–08.
32 Hong CJ, Chen JY, Chiu HJ, Sim CB. A double-blind comparative study of clozapine versus chlorpromazine on Chinese patients with treatment-refractory schizophrenia. *International Clinical Psychopharmacology* 1997; **12:** 123–30.
33 Honigfeld G, Patin J, Singer J. Clozapine. antipsychotic activity in treatment-resistant schizophrenics. *Advances in Therapy* 1984; **1:** 77–97.
34 Howard JS. Haloperidol for chronically hospitalized psychotics. a double blind comparison with thiothixene and placebo: a follow up open evaluation. *Diseases of the Nervous System* 1974; **35:** 458–63.

35 Kahn RS, van Winter RI, Leucht S, et al. Amisulpride and olanzapine followed by open-label treatment with clozapine in first-episode schizophrenia and schizophreniform disorder (OPTiMiSE). a three-phase switching study. *Lancet Psychiatry* 2018; **5:** 797–807.
36 Kane JM, Honigfeld G, Singer J, Meltzer H. Clozapine in treatment-resistant schizophrenics. *Psychopharmacology Bulletin* 1988; **24:** 62–67.
37 Kane JM, Marder SR, Schooler NR, et al. Clozapine and haloperidol in moderately refractory schizophrenia. a 6-month randomized double-blind comparison. *Archives of General Psychiatry* 2001; **58:** 965–72.
38 Kane JM, Khanna S, Rajadhyaksha S, Giller E. Efficacy and tolerability of ziprasidone in patients with treatment-resistant schizophrenia. *International Clinical Psychopharmacology* 2006; **21:** 21–28.
39 Kane JM, Meltzer HY, Carson, W. H., Jr., McQuade RD, Marcus RN, Sanchez R. Aripiprazole for treatment-resistant schizophrenia. results of a multicenter, randomized, double-blind, comparison study versus perphenazine. *Journal of Clinical Psychiatry* 2007; **68:** 213–23.
40 Kane JM, Potkin SG, Daniel DG, Buckley PF. A double-blind, randomized study comparing the efficacy and safety of sertindole and risperidone in patients with treatment-resistant schizophrenia. *Journal of Clinical Psychiatry* 2011; **72:** 194–204.
41 Kane JM, Kinon B, Johns C. Alternative strategies for treating neuroleptic non responsive patients. *Schizophrenia Research* 1993; **9:** 240.
42 Kinon BJ, Chen L, Ascher-Svanum H, et al. Early response to antipsychotic drug therapy as a clinical marker of subsequent response in the treatment of schizophrenia. *Neuropsychopharmacology* 2010; **35:** 581–90.
43 Kumra S, Frazier JA, Jacobsen LK, et al. Childhood-onset schizophrenia. A double-blind clozapine-haloperidol comparison. *Archives of General Psychiatry* 1996; **53:** 1090–97.
44 Kumra S, Kranzler H, Gerbino-Rosen G, et al. Clozapine and "high-dose" olanzapine in refractory early-onset schizophrenia. a 12-week randomized and double-blind comparison. *Biological Psychiatry* 2008; **63:** 524–29.
45 Lal S, Thavundayil JX, Nair NPV, et al. Levomepromazine versus chlorpromazine in treatment-resistant schizophrenia. a double-blind randomized trial. *Journal of Psychiatry and Neuroscience* 2006; **31:** 271–79.
46 Marjerrison G, Irvine D, Stewart CN, Williams R, Matheu H, Demay M. Withdrawal of long term phenothiazines from chronically hospitalized psychiatric patients. *Canadian Psychiatric Association Journal* 1964; **60:** 290–98.
47 McCreadie RG, MacDonald IM. High dosage haloperidol in chronic schizophrenia. *British Journal of Psychiatry* 1977; **131:** 310–16.
48 McEvoy JP, Lieberman JA, Stroup TS, et al. Effectiveness of clozapine versus olanzapine, quetiapine, and risperidone in patients with chronic schizophrenia who did not respond to prior atypical antipsychotic treatment. *American Journal of Psychiatry* 2006; **163:** 600–10.
49 Meltzer HY, Bobo WV, Roy A, et al. A randomized, double-blind comparison of clozapine and high-dose olanzapine in treatment-resistant patients with schizophrenia. *Journal of Clinical Psychiatry* 2008; **69:** 274–85.
50 Mercer G, Finlayson A, Johnstone EC, Murray C, Owens DGC. A study of enhanced management in patients with treatment-resistant schizophrenia. *Journal of Psychopharmacology* 1997; **11:** 349–56.
51 Meyer-Lindenberg A, Gruppe, H., Bauer U, Lis S, Krieger S, Gallhofer B. Improvement of cognitive function in schizophrenic patients receiving clozapine or zotepine. results from a double-blind study. *PharmacoPsychiatry* 1997; **30:** 35–42.

52 Moresco RM, Cavallaro R, Messa C, et al. Cerebral D2 and 5-HT2 receptor occupancy in schizophrenic patients treated with olanzapine or clozapine. *Journal of Psychopharmacology* 2004; **18:** 355–65.
53 Naber D, Riedel M, Klimke A, et al. Randomized double blind comparison of olanzapine vs clozapine on subjective well-being and clinical outcome in patients with schizophrenia. *Acta Psychiatrica Scandinavica* 2005; **111:** 106–15.
54 Rosenheck R, Cramer J, Xu W, et al. A comparison of clozapine and haloperidol in hospitalized patients with refractory schizophrenia. *New England Journal of Medicine* 1997; **337:** 809–15.
55 Sacchetti E, Galluzzo A, Valsecchi P, Romeo F, Gorini B, Warrington L. Ziprasidone vs clozapine in schizophrenia patients refractory to multiple antipsychotic treatments. the MOZART study. *Schizophrenia Research* 2009; **113:** 112–21.
56 Schiele BC, Vestre ND, Stein KE. A comparison of thioridazine, trifluoperazine, chlorpromazine, and placebo. a double-blind controlled study on the treatment of chronic hospitalized, schizophrenic patients. *Journal of Clinical and Experimental Psychopathology* 1961; **22:** 151–62.
57 Schooler NR, Marder SR, Chengappa KN, et al. Clozapine and risperidone in moderately refractory schizophrenia. a 6-month randomized double-blind comparison. *Journal of Clinical Psychiatry* 2016; **77:** 628–34.
58 See RE, Fido AA, Maurice M, Ibrahim MM, Salama GM. Risperidone induced increase of plasma norepinephrine is not correlated with symptom improvement in chronic schizophrenia. *Biological Psychiatry* 1999; **45:** 1653–56.
59 Shalev A, Hermesh H, Rothberg J, Munitz H. Poor neuroleptic response in acutely exacerbated schizophrenic patients. *Acta Psychiatrica Scandinavica* 1993; **87:** 86–91.
60 Shaw P, Sporn A, Gogtay N, et al. Childhood-onset schizophrenia. a double-blind, randomized clozapine-olanzapine comparison. *Archives of General Psychiatry* 2006; **63:** 721–30.
61 Sirota P, Pannet I, Koren a, Tchernichovsky E. Quetiapine versus olanzapine for the treatment of negative symptoms in patients with schizophrenia. *Human Psychopharmacology* 2006; **21:** 227–34.
62 Smith RC, Infante M, Singh A, Khandat A. The effects of olanzapine on neurocognitive functioning in medication-refractory schizophrenia. *International Journal of Neuropsychopharmacology* 2001; **4:** 239–50.
63 Suzuki T, Uchida H, Watanabe K, et al. How effective is it to sequentially switch among olanzapine, quetiapine and risperidone? - A randomized, open-label study of algorithm-based antipsychotic treatment to patients with symptomatic schizophrenia in the real-world clinical setting. *Psychopharmacology* 2007; **195:** 285–95.
64 Tollefson GD, Birkett MA, Kiesler GM, Wood AJ, Lilly, Resistant Schizophrenia Study Group. Double-blind comparison of olanzapine versus clozapine in schizophrenic patients clinically eligible for treatment with clozapine. *Biological Psychiatry* 2001; **49:** 52–63.
65 Toru M, Shimazono Y, Miyasaka M, Kokubo T, Mori Y, Nasu T. A double-blind comparison of sulpiride with chlorpromazine in chronic schizophrenia. *Journal of Clinical Pharmacology and New Drugs* 1972; **12:** 221–29.
66 Volavka J, Czobor P, Sheitman B, et al. Clozapine, olanzapine, risperidone, and haloperidol in the treatment of patients with chronic schizophrenia and schizoaffective disorder. *American Journal of Psychiatry* 2002; **159:** 255–62.
67 Wahlbeck K, Cheine M, Tuisku K, Ahokas A, Joffe G, Rimon R. Risperidone versus clozapine in treatment-resistant schizophrenia. a randomized pilot study. *Progress in Neuro-Psychopharmacology and Biological Psychiatry* 2000; **24:** 911–22.
68 Wirshing DA, Marshall, B. D., Jr., Green MF, Mintz J, Marder SR, Wirshing WC. Risperidone in treatment-refractory schizophrenia. *American Journal of Psychiatry* 1999; **156:** 1374–79.

69 Addington J, Addington D. Neurocognitive functioning in schizophrenia. a trial of risperidone versus haloperidol. *Canadian Journal of Psychiatry [Revue Canadienne de Psychiatrie]* 1997; **42:** 983.
70 Byerly M, Weber M. Clozapine versus quetiapine for schizophrenia. *Stanley Foundation Research Programs* 1999.
71 Estrella MH, Soria FL, Gonzalez CJC, Butron MAL, Torres JA, Escareño RR. Cost-effectiveness of clozapine vs respiridone for treatment-resistant schizophrenic patients. *Proceedings of the 10th World Congress of Psychiatry; 1996 Aug 23-28; Madrid, Spain* 1996.
72 Nct. Clozapine vs olanzapine. an effectiveness study. *https://ClinicalTrials.gov/* 2005.
73 Hamilton M, Smith ALG, Lapidus HR, Cadogan EP. A controlled trial of thiopropazate dihydrochloride (dartalan), chlorpromazine and occupational therapy in chronic schizophrenics. *Journal of Mental Science* 1960; **106:** 40–55.
74 Heres S, Cordes J, Feyerabend S, et al. Changing the Antipsychotic in Early Nonimprovers to Amisulpride or Olanzapine: Randomized, Double-Blind Trial in Patients With Schizophrenia. *Schizophrenia Bulletin* 2022; **48:** 1273–83. https://doi.org/10.1093/schbul/sbac068.
75 Heylen S, Gelders Y, Bussche GV. Risperidone versus haloperidol in chronic psychotic patients. an 8 week multicenter double-blind comparative trial. *Psychopharmacology* 1988; **96:** 238.
76 Mergl RP, Gallinat J, Riedel M, Schröter A, Tigges P, Hegerl U. Digitised hand movement analysis of EPMS. *Proceedings of the 11th World Congress of Psychiatry; 1999 Aug 6-11; Hamburg, Germany* 1999; **2:** 164.
77 Oliemeulen EAP, Jogems-Kosterman BJM, Van, Hoof J. J. M. Is olanzapine a substitute for clozapine? *Schizophrenia Research* 1999; **36:** 146–47.
78 Salganik I, Modai I, Bercovici BR, Kutzuk D, Weizman A. Clozapine vs haloperidol therapy in elderly chronic schizophrenic inpatients. preliminary results. A double blind, cross over randomized study. *International Journal of Geriatric Psychopharmacology* 1998; **1:** 185–87.
79 Chaimani A, Caldwell DM, Li T, Higgins JPT, Salanti G. Undertaking network meta-analyses, 2019.
80 Leucht S, Rothe P, Davis JM, Engel RR. Equipercentile linking of the BPRS and the PANSS. *Eur Neuropsychopharmacol* 2013; **23:** 956–59. https://doi.org/10.1016/j.euroneuro.2012.11.004.
81 Samara MT, Dold M, Gianatsi M, et al. Efficacy, Acceptability, and Tolerability of Antipsychotics in Treatment-Resistant Schizophrenia: A Network Meta-analysis. *JAMA psychiatry* 2016; **73:** 199–210. https://doi.org/10.1001/jamapsychiatry.2015.2955.
82 Nikolakopoulou A, Higgins JPT, Papakonstantinou T, et al. CINeMA: An approach for assessing confidence in the results of a network meta-analysis. *PLoS Med* 2020; **17:** e1003082. https://doi.org/10.1371/journal.pmed.1003082.
83 Chiocchia V, Nikolakopoulou A, Higgins JPT, et al. ROB-MEN: a tool to assess risk of bias due to missing evidence in network meta-analysis. *BMC Medicine* 2021; **19:** 304. https://doi.org/10.1186/s12916-021-02166-3.
84 Schneider-Thoma J, Chalkou K, Dörries C, et al. Comparative efficacy and tolerability of 32 oral and long-acting injectable antipsychotics for the maintenance treatment of adults with schizophrenia: a systematic review and network meta-analysis. *Lancet* 2022; **399:** 824–36. https://doi.org/10.1016/S0140-6736(21)01997-8.
85 Bighelli I, Rodolico A, García-Mieres H, et al. Psychosocial and psychological interventions for relapse prevention in schizophrenia: a systematic review and network meta-analysis. *Lancet Psychiatry* 2021; **8:** 969–80. https://doi.org/10.1016/S2215-0366(21)00243-1.
